# Sigmatropic [1,5] Carbon Shift of Transient C3 Ammonium Enolates

**DOI:** 10.1002/anie.202204378

**Published:** 2022-06-10

**Authors:** Garrit Wicker, Rundong Zhou, Roland Schoch, Jan Paradies

**Affiliations:** ^1^ Department of Chemistry Paderborn University Warburger Strasse 100 33098 Paderborn Germany

**Keywords:** Ammonium Enolate, Asymmetric, Density Functional Calculations, Sigmatropic Rearrangement, Tetrahydroquinoline

## Abstract

The stereospecific sigmatropic [1,5] carbon shift of C3 ammonium enolates is discovered. According to mechanistic, kinetic and computational experiments, this new rearrangement proceeds via the catalytic generation of a transient C3 ammonium enolate by intramolecular aza‐Michael addition. This intermediate rapidly undergoes [1,5] sigmatropic carbon migration to furnish the respective tetrahydroquinoline‐4‐ones with excellent diastereoselectivities of d.r. >99 : 1 and in 61–98 % yield.

## Graphical abstract

The discovery of a new [1,5] sigmatropic rearrangement is described. With the aid of a borane‐based Lewis acid, the transiently generated C3 ammonium enolates undergo a sigmatropic [1,5] shift of benzylic, allylic and propargylic groups to provide access to *trans*‐configured tetrahydroquinoline‐4‐ones with diastereoselectivity of >99 : 1. The reaction is investigated in kinetic, mechanistic and computational experiments.

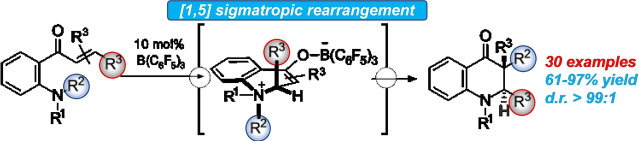

Rearrangement reactions are one of the most useful tools for construction of organic scaffolds.^[1]^ In fact, [1,2]^[2]^ and [3,3]^[3]^ sigmatropic rearrangements are typical reactions used to achieve molecular complexity with high stereocontrol. Observations of sigmatropic shifts of hydrogen, silicon or acyl groups across extended π‐systems, such as cyclopentadiene, indenyl^[4]^ or quinone[^4d^, ^5^] structures, have provided a basis for in‐depth understanding of organic chemistry^[6]^ in addition to the synthetic benefits. Electrophilic nitrogen‐ylide rearrangements, like the [1,2] and [2,3] Stevens^[7]^ and Sommelet–Hauser^[8]^ rearrangements, offer stereoselective access to synthetically useful amino building blocks through nitrogen to carbon chirality transfer.^[9]^ The [1,2] Stevens progresses by homolytic C−N bond dissociation with formation of a caged radical pair, while the [2,3] Stevens and the Sommelet–Hauser rearrangements proceed by a concerted mechanism (Scheme 1a and b).

**Scheme 1:**
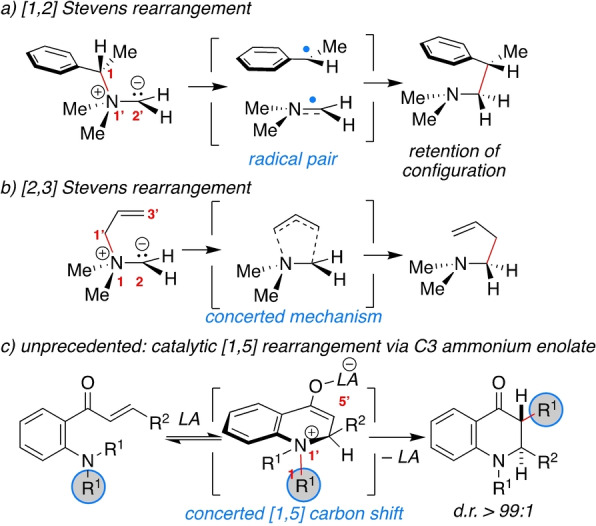
a) [1,2] and b) [2,3] Stevens rearrangement; c) sigmatropic [1,5] carbon shift of C3 ammonium enolates.

Both rearrangements require, first, quaternization of the nitrogen atom and, as a second step, formation of the ylide by deprotonation. There have been only a few reported examples of catalytic creation of the ylide intermediate.^[10]^ And although C3 ammonium enolates^[11]^ are well‐known intermediates in aza‐Michael and Morita–Baylis–Hillman reactions,^[12]^ cyclic C3 ammonium enolates have yet to be considerd as precursors for sigmatropic [1,5] rearrangements (Scheme 1c).

We had observed during our recent investigations of redox isomerization^[13]^ an unsusal migration of a benzylic fragment (Scheme 2, top).

**Scheme 2:**
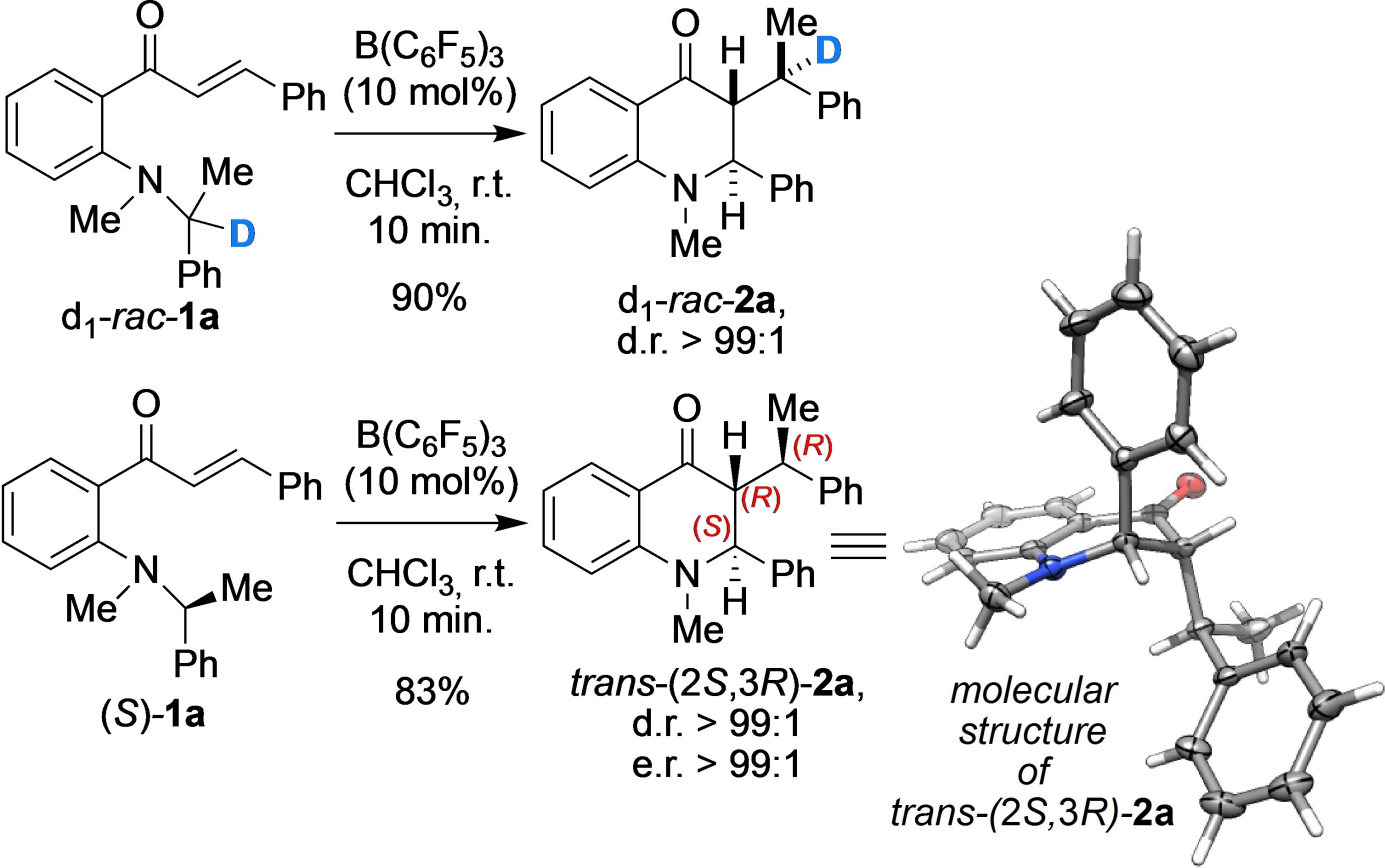
Lewis acid‐induced benzyl migration.^[14]^

Unexpectedly, the reaction of the amino chalcone *d_1_*‐*rac*‐**1 a** with B(C_6_F_5_)_3_ ^[15]^ did not furnish the corresponding deuteride shift product, but rather the carbon migration product d_1_‐*rac*‐**2 a** as a single diastereomer in 90 % yield. Remarkably, when the reaction was performed with the enantiopure (*S*)‐phenylethyl derivative (*S*)‐**1 a**, only the *trans*‐**2 a** was obtained as a single enantiomer (e.r. 99 : 1) in 83 % yield (Scheme 2, bottom). Crystal structure analysis^[16]^ of the reaction product permitted indirect assignment of the absolute configuration to that of *trans‐*(2*S*,3*R*)‐(*S*)‐phenylethyltetrahydroquinolone **2 a**, which indicates that the reaction proceeds under excellent stereocontrol with complete chirality transfer and retention of configuration of the migrating carbon center. Careful inspection of the reaction by ^1^H NMR spectroscopy did not provide deeper insight into the reaction mechanism, because only the starting materials and *trans*‐**2 a** were detectable. However, the ^11^B NMR featured resonances at *δ*=−1.8 ppm and −5.8 ppm, accounting for tetragonal B−O adducts. We anticipated that the reaction might proceed by a Lewis acid‐induced intramolecular aza‐Michael reaction setting the stage for a so far unprecedented [1,5] carbon shift of a transiently generated C3 ammonium enolate. To support that such a process was at work, we isolated and characterized the C3 ammonium enolate **4** as the reaction product of 4‐*N*,*N*‐(dimethylamino)chalcone (**3**) with 1.0 equiv B(C_6_F_5_)_3_ (Scheme 3).

**Scheme 3:**
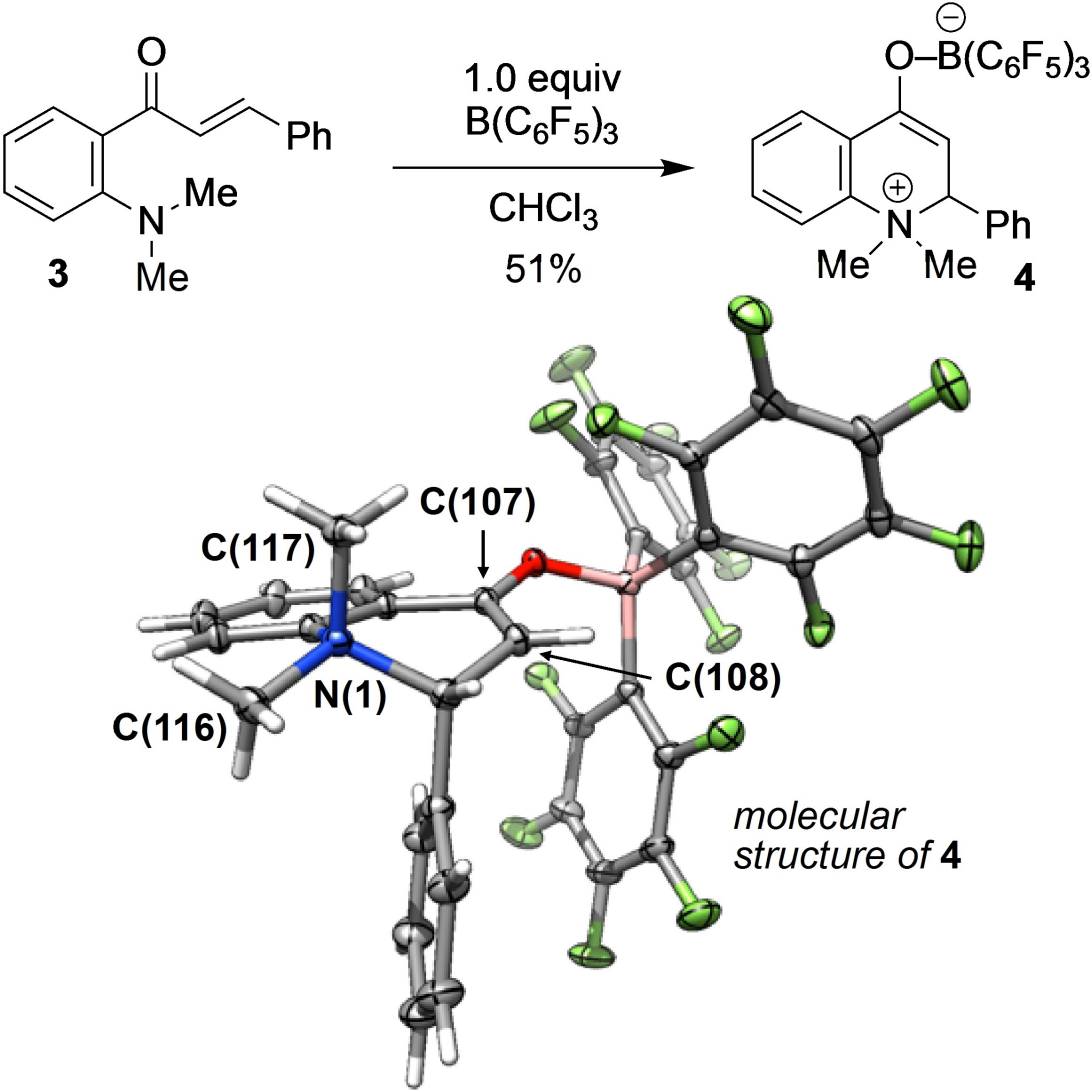
Reaction of **3** with B(C_6_F_5_)_3_; molecular structure of **4**; selected bond lengths: N(1)−C(116) 1.5080(12) Å; N(1)−C(117) 1.5138(12) Å; C(107)−C(108) 1.3435(13) Å.

The bond length between C(107)−C(108) of 1.3435(13) Å is in agreement with a benzylic enolate double bond (1.3565(11) Å).^[17]^ Notably, the nitrogen atom is quaternized, with one methyl group in equatorial position and the second axial and antiperiplanar to the phenyl substituent. The axial N(1)−C(117) bond is slightly elongated compared to the equatorial N(1)−C(116) bond, suggesting electronic interaction with the aromatic system. Since the conjugate addition is of utmost importance for the overall reaction, we investigated substituent effects at the Michael acceptor using 4‐methoxybenzyl (PMB) as migrating group (Scheme 4). To our delight, electron‐rich and electron‐deficient substituents at the olefin were very well tolerated. However, we found that the reaction rate is sensitive to electronic modifications. The Hammett analysis of **1 b**–**d** displays a positive slope of *ρ*=1.889±0.13, which implies that the negative charge arising from nucleophilic attack at the Michael position in the rate‐determining step needs to be stabilized. The tetrahydroquinolinone bearing a quaternary stereocenter **2 e** was obtained in 88 % yield as a single diastereomer. Also, double substitution at the Michael position was tolerated such that **2 f** was produced in 65 % yield. Bulky tert‐butyl substituents (**2 g**) as well as heterocyclic ones, such as furanyl (**2 h**), thiophenyl (**2 i**) or pyridyl (**2 j**), were well tolerated. The molecular structure of **2 j** justifies our assignment of it to the *trans* diastereomer. Next, we studied the impact of the nitrogen substituents on the rearrangement reaction (Scheme 5).

**Scheme 4:**
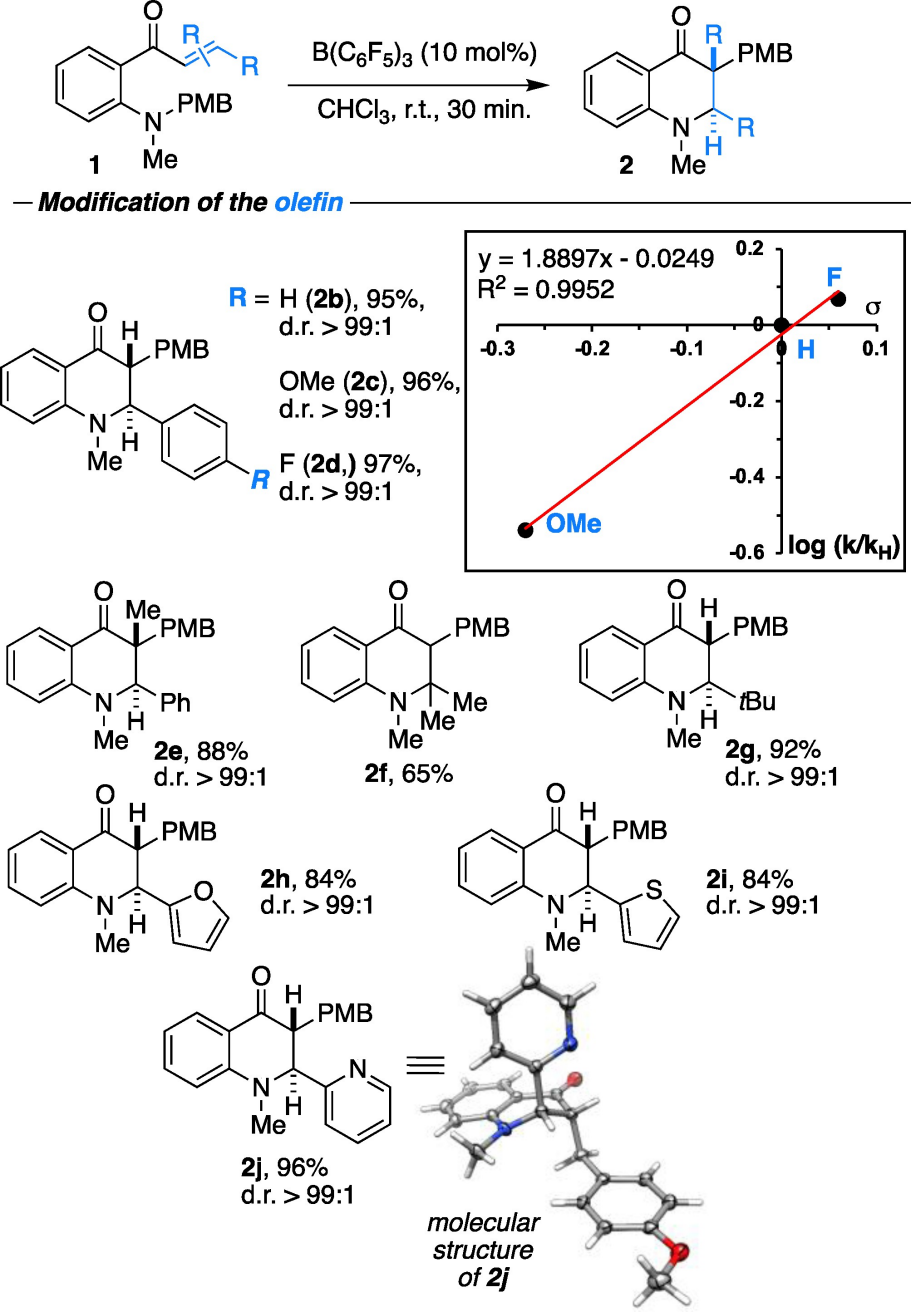
Modifications at the olefin in the sigmatropic [1,5] carbon rearrangement; B(2,4,6‐F_3_‐C_6_H_2_)_3_ (10 mol %) was used as catalyst in the Hammett analysis.

**Scheme 5:**
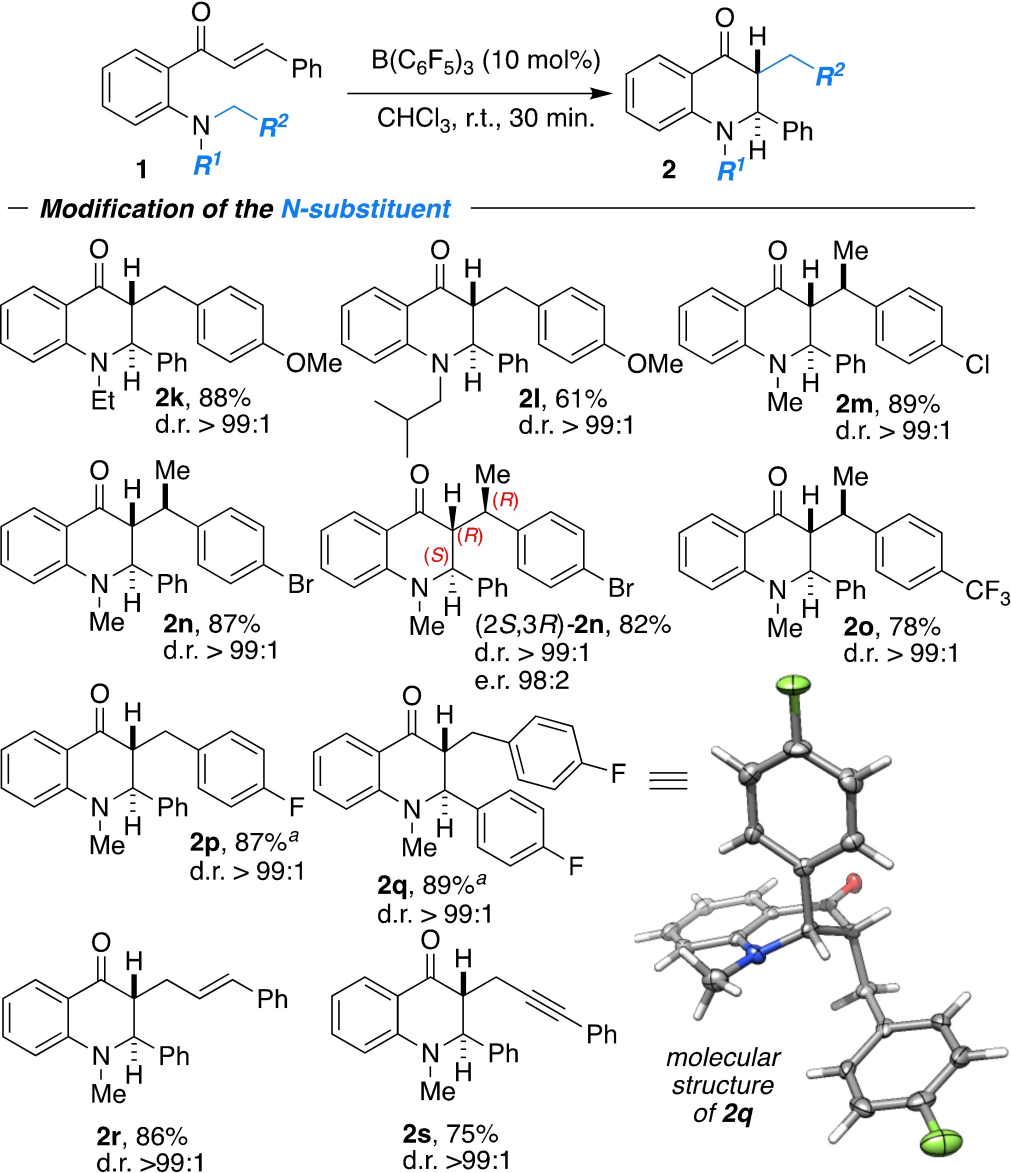
Scope of the nitrogen substituent in the sigmatropic [1,5] carbon rearrangement; [a] performed at 60 °C.

Larger substituents on the nitrogen atom, like ethyl (**2 k**) or isobutyl (**2 l**), did not suppress the reaction, but slightly diminished yields were obtained, with preservation of the excellent diastereoselectivity of >99 : 1. Thereafter, we investigated the scope of the migrating group attached to the nitrogen atom. The phenylethyl derivatives **2 m**–**o** were obtained as single diastereomers in 78–89 % yield. The optical purity of (*S*)‐**1 n** (96 % ee) was quantitatively transferred to the three stereocenters of (2*S*,3*R*)‐**2 n**. The fluoro derivatives **1 p** and **1 q** required elevated temperatures of 60 °C, probably due to the reduced nucleophilicity of the nitrogen atom. However, both products **2 p** and **2 q** were obtained without depletion of the perfect diastereoselectivity at 87 % and 89 % yield, respectively. We successfully expanded the scope of the migrating groups to the cinnamyl and propargyl derivatives **1 r** and **1 s**, which cleanly underwent migration. The products **2 r** and **2 s** were obtained in 86 % and 75 % yield as single diastereomers (d.r. >99 : 1). The impact of various substituents on the aromatic backbone was probed (Scheme 6).

**Scheme 6:**
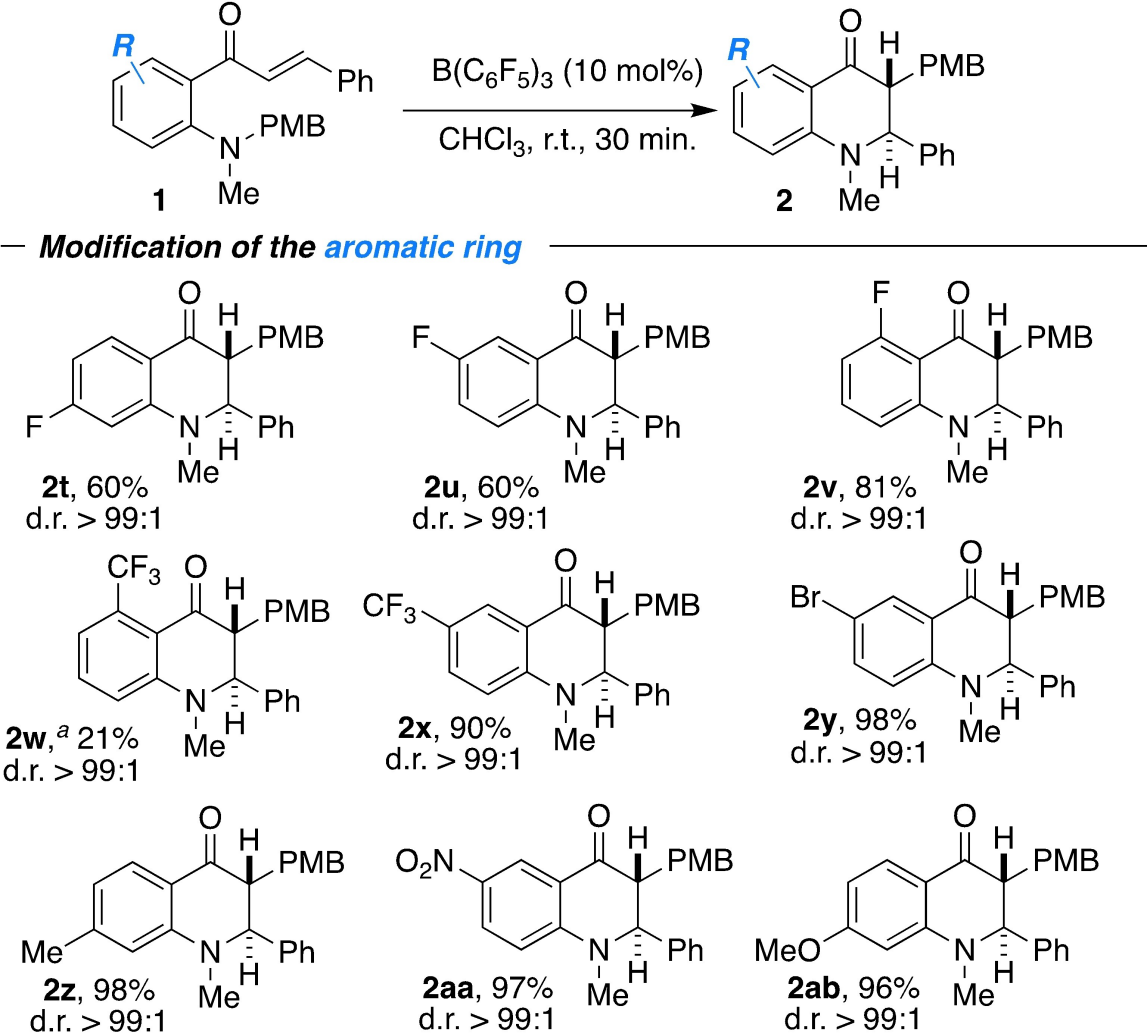
Modification of the aromatic ring; [a] the reaction was performed for 18 h.

The products **2 t**–**2 v** were obtained in 60 % to 81 % yields. The *ortho*‐CF_3_ substituted derivative **2 w** was obtained in 21 % yield, probably as a result of steric crowding of the carbonyl group. Electronic effects seem to play a minor role because the *meta*‐CF_3_ substituted **1 x** was converted into **2 x** in 90 % yield, although the N‐atom should be significantly less nucleophilic. Furthermore, bromo, methyl, nitro and methoxy groups were well tolerated (**2 y**–**2 ab**). All products were produced with an excellent diastereoselectivity of >99 : 1.

Finally, we investigated the reaction of (*S*)‐**1 a** in detail by density functional theory (DFT) on the PW6B95/def2‐QZVPP//PBEh‐3c/def2‐mSVP^[18]^ level including dispersion correction D3BJ and the CPCM(CHCl_3_) solvent model as implemented in the ORCA package^[19]^ after preoptimizations with GFN2‐xTB.^[20]^ Transition states were located by the growing string method^[21]^ in combination with GFN2‐xTB and then further optimized by DFT‐level calculations. The binding of B(C_6_F_5_)_3_ to (*S*)‐**1 a** is endergonic by 6.0 kcal mol^−1^ and induces the change from the *s‐cis* to the *s‐trans* conformer. Both conformations provide a perfect setup for the aza‐Michael addition (Figure 1, see Supporting Information for details). The addition of the nitrogen atom to the α,β‐unsaturated carbonyl fragment is the rate‐ and stereo‐discriminating step. The barrier of the *Si*‐face attack (TS_1_) is 2.5 kcal mol^−1^ lower in free energy than the *Re*‐face attack (TS_1′_).

**Figure 1:**
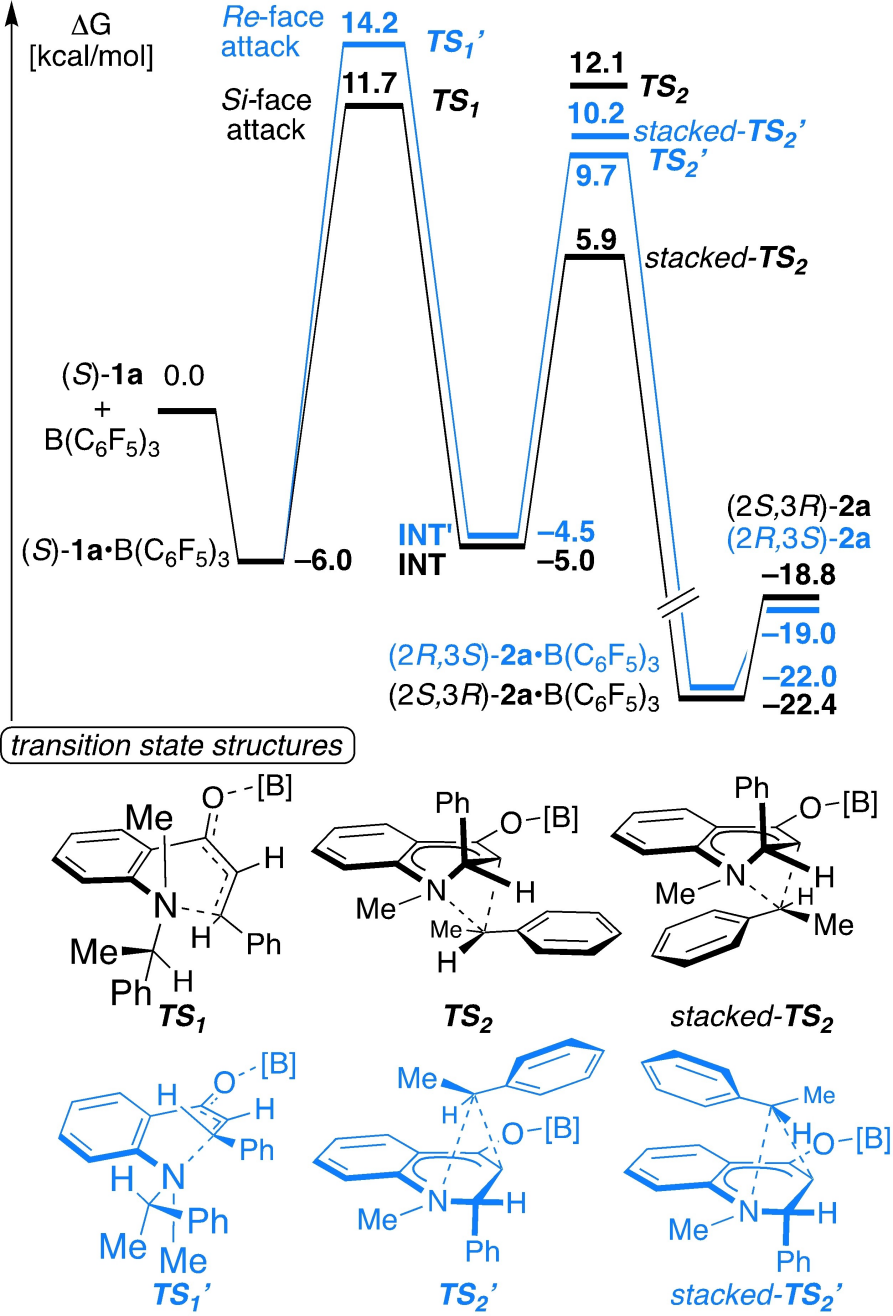
Calculated free reaction energies in kcal mol^−1^ and transition state geometries ([B]=B(C_6_F_5_)_3_).

The diastereomeric C3 ammonium enolates INT and INT′ are nearly equal in free energy. For each of the diastereomeric transition states of the [1,5] carbon shift, two low‐energy conformers were identified, which differ by the orientation of the phenylethyl group (Figure 1, bottom: TS_2_, TS_2′_ and *stacked*‐TS_2_, *stacked*‐TS_2′_). For both C3 ammonium enolate intermediates (INT and INT′), the lowest barriers for the [1,5] carbon shift via *stacked*‐TS_2_ and *stacked*‐TS_2′_ are lower than those for aza‐Michael addition, so it is reasonable that these intermediates were not detectable by NMR spectroscopy. The *stacked*‐TS_2_ benefits energetically from both the stabilizing C_6_H_5_‐C_6_F_5_ and quinoline‐migrating group π‐π interactions (see NCI plots^[22]^ in Supporting Information, Figure S5 and S6). The higher barrier of TS_2_ by 6.2 kcal mol^−1^ may be attributed to the reduced π‐π interaction of the migrating group with the tetrahydroquinoline. A comparable ΔΔG* of 6.4 kcal mol^−1^ was computed for the [1,5] shift of **1 b** (Figure S7 and S8).] The stabilizing C_6_H_5_−C_6_F_5_ interaction is absent in both TS_2′_ transition states, which may account for the higher barrier of approx. 15–17 kcal mol^−1^. However, both of these interactions are not crucial for the sigmatropic shift, because the aliphatic derivatives **1 f** and **1 g** (see Scheme 4) and the substituted benzoids **1 t**–**1 x** (see Scheme 6) were cleanly converted into the corresponding tetrahydroquinolinones. The formation of (2*S*,3*R*)‐**2 a**⋅B(C_6_F_5_)_3_ is exergonic by 22.4 kcal mol^−1^, and it is more stable than the corresponding free species. This is in agreement with the ^11^B NMR experiments, which confirmed that only tetragonal B−O adducts were present. The transition state *stacked‐*TS_2_ was studied in more detail by reoptimization with the range‐separated hybrid functional ωB97X‐D3BJ/def2‐TZVP.^[23]^ The electronic structure of the transition state in the [1,2] Stevens rearrangement has been the object of some debate and can be best described as singlet biradical.^[24]^ Unrestricted DFT calculations converged to the closed shell determinant (⟨S^2^⟩=0.000, Δ*E*_UKS_‐_RKS_=0.75 kcal mol^−1^), so *stacked*‐TS_2_ can be classified as a closed‐shell species. This is supported by a complete active space self‐consistent field (CASSCF) (2,2)/def2‐TZVP calculation which shows only minor LUMO population of 0.073.^[25]^ This result is corroborated by the observation that the reaction rate was not influenced when the reaction was performed in the presence of 1.0 equiv of TEMPO (see Supporting Information). The localized orbital analysis reveals double bond character for both the N‐aryl (aniline) and for the enolate fragment (Figure 2, top).

**Figure 2:**
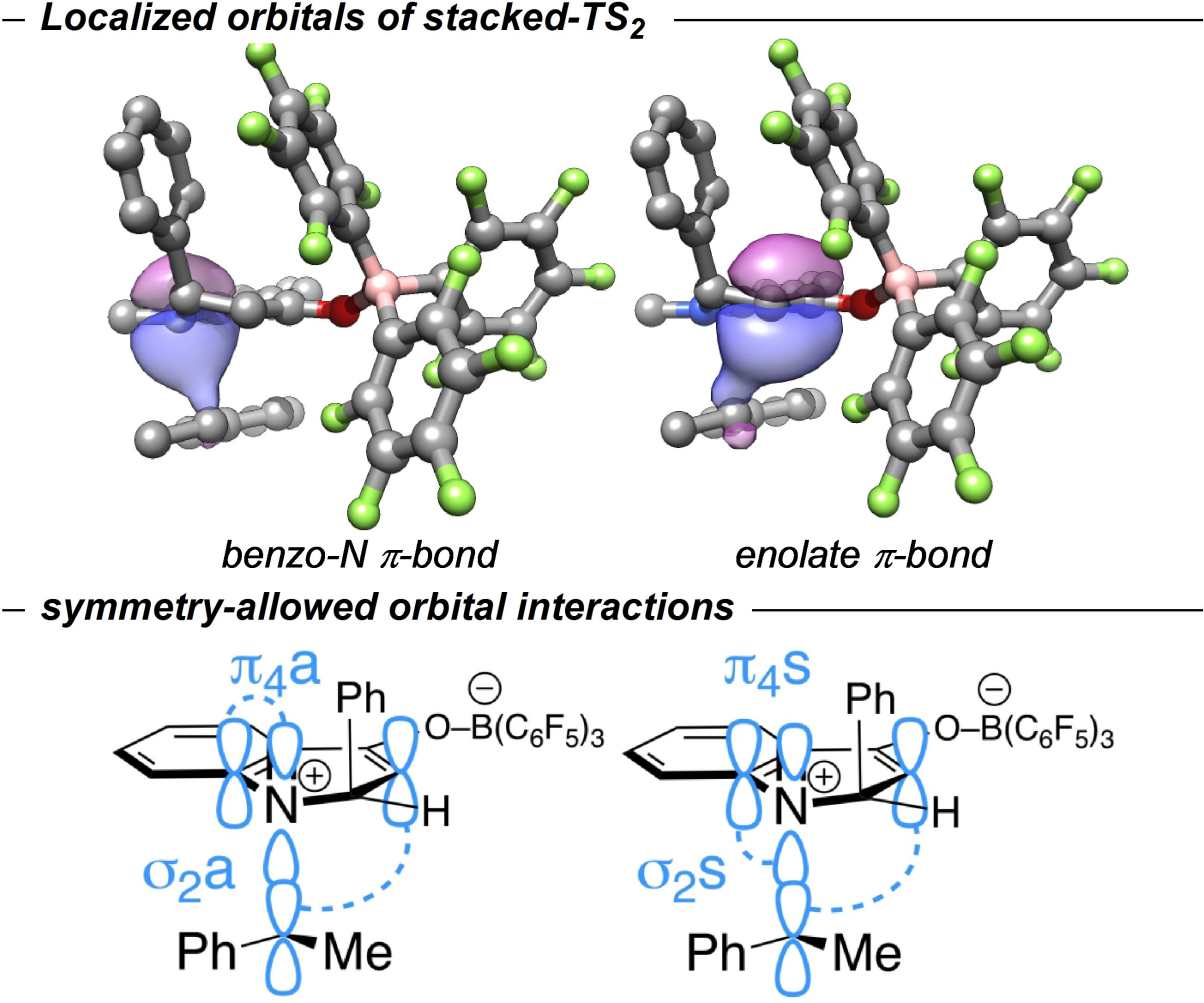
Localized orbitals of the benzo‐N and of the enolate π‐bond (UKS‐ ωB97X‐D3BJ/def2‐TZVP) of the transition state structure *stacked*‐TS_2_ (top); orbital analysis of Woodward–Hoffmann symmetry‐allowed processes (bottom).

The distances of the N atom and enolate C atom to the benzyl C atom deviate only marginally, by 0.08 Å (*r*_N‐benzyl_=2.87 Å, *r*_enolate‐benzyl_=2.95 Å). This indicates a concerted migration of the benzyl group in a slightly asynchronous transition state. The reaction can therefore be classified as a sigmatropic [1,5] rearrangement of a transiently formed C3 ammonium enolate. This is in accordance with Woodward–Hoffmann rules, which prescribe that it proceed via a π_4_s+σ_2_s or π_4_a+σ_2_a process (Figure 2, bottom).

In summary, we discovered a new stereospecific [1,5] sigmatropic rearrangement of transiently generated C3 ammonium enolates. In contrast to other electrophilic rearrangements, the reactive species is catalytically generated through an intramolecular aza‐Michael reaction. Kinetic, mechanistic and computational experiments all indicate that the rate‐determining step is the initial formation of the zwitterionic C3 ammonium enolate. The symmetry‐allowed [1,5] shift proceeds with retention of configuration of the migrating carbon atom. The tetrahydroquinoline‐4‐ones were obtained in high yields and with excellent diastereoselectivity (d.r. >99 : 1).

## Conflict of interest

The authors declare no conflict of interest.

## Supporting information

As a service to our authors and readers, this journal provides supporting information supplied by the authors. Such materials are peer reviewed and may be re‐organized for online delivery, but are not copy‐edited or typeset. Technical support issues arising from supporting information (other than missing files) should be addressed to the authors.

Supporting Information

## Data Availability

The data that support the findings of this study are available in the Supporting Information of this article.

## References

[1a] C. M. Rojas , Molecular Rearrangements in Organic Synthesis, Wiley, Hoboken, 2015;

[1b] C. L. Hugelshofer , T. Magauer , Nat. Prod. Rep. 2017, 34, 228–234; 28180228 10.1039/c7np00005g

[1c] H. Wu , Q. Wang , J. Zhu , Eur. J. Org. Chem. 2019, 1964–1980.

[2a] Z.-L. Song , C.-A. Fan , Y.-Q. Tu , Chem. Rev. 2011, 111, 7523–7556; 21851053 10.1021/cr200055g

[2b] B. Delayre , Q. Wang , J. Zhu , ACS Cent. Sci. 2021, 7, 559–569. 34056086 10.1021/acscentsci.1c00075 PMC8155462

[3a] U. Nubbemeyer , Synthesis 2003, 961–1008;

[3b] E. A. Ilardi , C. E. Stivala , A. Zakarian , Chem. Soc. Rev. 2009, 38, 3133–3148; 19847347 10.1039/b901177n PMC4103198

[3c] W. Zhang , B. Nay , Eur. J. Org. Chem. 2020, 3517–3525.

[4a] L. L. Miller , R. Greisinger , R. F. Boyer , J. Am. Chem. Soc. 1969, 91, 1578–1580;

[4b] D. J. Field , D. W. Jones , G. Kneen , Chem. Commun. 1976, 873–874;

[4c] W. R. Dolbier , K. E. Anapolle , L. McCullagh , K. Matsui , J. M. Riemann , D. Rolison , J. Org. Chem. 1979, 44, 2845–2849;

[4d] D. J. Field , D. W. Jones , J. Chem. Soc. Perkin Trans. 1 1980, 714–721;

[4e] M. Stradiotto , M. A. Brook , M. J. McGlinchey , Perkin Trans. 1 2000, 611–618;

[4f] S. Yamabe , N. Tsuchida , S. Yamazaki , J. Chem. Theory Comput. 2005, 1, 944–952. 26641910 10.1021/ct0500646

[5a] S. C. Cooper , P. G. Sammes , Chem. Commun. 1980, 633–634;

[5b] P. Loeliger , H. Mayer , Helv. Chim. Acta 1980, 63, 1604–1608.

[6a] F.-G. Klärner , Topics in Stereochemistry, Wiley, New York, 1984, pp. 1–42;

[6b] F. Jensen , J. Am. Chem. Soc. 1989, 111, 4643–4647;

[6c] A. Kless , M. Nendel , S. Wilsey , K. N. Houk , J. Am. Chem. Soc. 1999, 121, 4524–4525;

[6d] I. V. Alabugin , M. Manoharan , B. Breiner , F. D. Lewis , J. Am. Chem. Soc. 2003, 125, 9329–9342; 12889962 10.1021/ja035729x

[6e] H. Goossens , J. M. Winne , S. Wouters , L. Hermosilla , P. J. De Clercq , M. Waroquier , V. Van Speybroeck , S. Catak , J. Org. Chem. 2015, 80, 2609–2620. 25615563 10.1021/jo5027639

[7a] T. S. Stevens , J. Chem. Soc. 1930, 2107–2119;

[7b] T. S. Stevens , W. W. Snedden , E. T. Stiller , T. Thomson , J. Chem. Soc. 1930, 2119–2125;

[7c] T. Thomson , T. S. Stevens , J. Chem. Soc. 1932, 1932–1940.

[8a] M. C. R. Sommelet , Hebd. Seances Acad. Sci. 1937, 205, 56;

[8b] C. R. Hauser , S. W. Kantor , J. Am. Chem. Soc. 1951, 73, 1437–1441;

[8c] S. W. Kantor , C. R. Hauser , J. Am. Chem. Soc. 1951, 73, 4122–4131.

[9a] K. W. Glaeske , F. G. West , Org. Lett. 1999, 1, 31–34;

[9b] J. A. Workman , N. P. Garrido , J. Sançon , E. Roberts , H. P. Wessel , J. B. Sweeney , J. Am. Chem. Soc. 2005, 127, 1066–1067; 15669822 10.1021/ja043768i

[9c] E. Tayama , S. Nanbara , T. Nakai , Chem. Lett. 2006, 35, 478–479;

[9d] J. A. Vanecko , H. Wan , F. G. West , Tetrahedron 2006, 62, 1043–1062;

[9e] E. Tayama , H. Kimura , Angew. Chem. Int. Ed. 2007, 46, 8869–8871; 10.1002/anie.200703832 17943952

[9f] J. B. Sweeney , Chem. Soc. Rev. 2009, 38, 1027–1038; 19421580 10.1039/b604828p

[9g] S. Bhakat , J. Chem. Pharm. Res. 2011, 115–121;

[9h] J. Clayden , M. Donnard , J. Lefranc , D. J. Tetlow , Chem. Commun. 2011, 47, 4624–4639; 10.1039/c1cc00049g 21380444

[9i] G. Lahm , J. C. O. Pacheco , T. Opatz , Synthesis 2014, 46, 2413–2421;

[9j] E. Tayama , K. Watanabe , Y. Matano , Eur. J. Org. Chem. 2016, 3631–3641;

[9k] H. Cho , H. Jeon , J. E. Shin , S. Lee , S. Park , S. Kim , Chem. Eur. J. 2019, 25, 2447–2451; 30569571 10.1002/chem.201804965

[9l] E. Tayama , Heterocycles 2016, 92, 793–828;

[9m] D. Baidilov , Synthesis 2020, 52, 21–26.

[10a] M. P. Doyle , W. H. Tamblyn , V. Bagheri , J. Org. Chem. 1981, 46, 5094–5102;

[10b] A. Soheili , U. K. Tambar , J. Am. Chem. Soc. 2011, 133, 12956–12959; 21793509 10.1021/ja204717b

[10c] S. S. M. Spoehrle , T. H. West , J. E. Taylor , A. M. Z. Slawin , A. D. Smith , J. Am. Chem. Soc. 2017, 139, 11895–11902; 28763205 10.1021/jacs.7b05619 PMC5579534

[10d] S. Xi , J. Dong , H. Chen , Q. Dong , J. Yang , Q. Tan , C. Zhang , Y. Lan , M. Zhang , Sci. Adv. 2021, 7, eabd5290. 33514546 10.1126/sciadv.abd5290 PMC7846163

[11a] M. J. Gaunt , C. C. C. Johansson , Chem. Rev. 2007, 107, 5596–5605; 18072805 10.1021/cr0683764

[11b] L. C. Morrill , A. D. Smith , Chem. Soc. Rev. 2014, 43, 6214–6226. 24867308 10.1039/c4cs00042k

[12] For recent applications of aza-Michael additions see:

[12a] T. Kang , L. Hou , S. Ruan , W. Cao , X. Liu , X. Feng , Nat. Commun. 2020, 11, 3869; 32747706 10.1038/s41467-020-17681-9 PMC7398931

[12b] P. Sharma , R. Gupta , R. K. Bansal , Beilstein J. Org. Chem. 2021, 17, 2585–2610; 34760026 10.3762/bjoc.17.173 PMC8551878

[12c] Y.-X. Song , D.-M. Du , Adv. Synth. Catal. 2021, 363, 4667–4694;

[12d] S. Wang , R. Guillot , J.-F. Carpentier , Y. Sarazin , C. Bour , V. Gandon , D. Lebœuf , Angew. Chem. Int. Ed. 2020, 59, 1134–1138; 10.1002/anie.201911761 31661585

[12e] S. Arava , S. K. Santra , G. K. Pathe , R. Kapanaiah , A. M. Szpilman , Angew. Chem. Int. Ed. 2020, 59, 15171–15175; 10.1002/anie.202005286 32394609

[12f] Z.-H. Yang , P. Chen , Z.-C. Chen , Z. Chen , W. Du , Y.-C. Chen , Angew. Chem. Int. Ed. 2021, 60, 13913–13917; 10.1002/anie.202102842 33829638

[12g] L. Zhang , W. Bao , Y. Liang , W. Pan , D. Li , L. Kong , Z.-X. Wang , B. Peng , Angew. Chem. Int. Ed. 2021, 60, 11414–11422; 10.1002/anie.202100497 33644970

[13a] S. Tamke , Z.-W. Qu , N. A. Sitte , U. Flörke , S. Grimme , J. Paradies , Angew. Chem. Int. Ed. 2016, 55, 4336–4339; 10.1002/anie.201511921 26939012

[13b] A. F. G. Maier , S. Tussing , T. Schneider , U. Flörke , Z.-W. Qu , S. Grimme , J. Paradies , Angew. Chem. Int. Ed. 2016, 55, 12219–12223; 10.1002/anie.201606426 27594431

[13c] A. F. G. Maier , S. Tussing , H. Zhu , G. Wicker , P. Tzvetkova , U. Flörke , C. G. Daniliuc , S. Grimme , J. Paradies , Chem. Eur. J. 2018, 24, 16287–16291; 30230618 10.1002/chem.201804777

[13d] G. Wicker , R. Schoch , J. Paradies , Org. Lett. 2021, 23, 3626–3630; 33843243 10.1021/acs.orglett.1c01018

[13e] R. Zhou , J. Paradies , Eur. J. Org. Chem. 2021, 6334–6339.

[14] E. F. Pettersen , T. D. Goddard , C. C. Huang , G. S. Couch , D. M. Greenblatt , E. C. Meng , T. E. Ferrin , J. Comput. Chem. 2004, 25, 1605–1612. 15264254 10.1002/jcc.20084

[15] For a recent review see:

[15a] J. L. Carden , A. Dasgupta , R. L. Melen , Chem. Soc. Rev. 2020, 49, 1706–1725; for a recent application see:32100762 10.1039/c9cs00769e

[15b] W. Li , T. Werner , Org. Lett. 2017, 19, 2568–2571; refs. [13c] and [13d]. 28492083 10.1021/acs.orglett.7b00720

[16] Deposition Numbers 2127715 (for (2*S*,3*R*)-**2a**), 2127718 (for (*rac*)-**2a**), 2127717 (for **3**), 2127716 (for **2j**), and 2127719 (for **2q**) contains the supplementary crystallographic data for this paper. These data are provided free of charge by the joint Cambridge Crystallographic Data Centre and Fachinformationszentrum Karlsruhe Access Structures service.

[17] M. A. Nichols , C. M. Leposa , A. D. Hunter , M. Zeller , J. Chem. Crystallogr. 2007, 37, 825–829.

[18a] F. Weigend , R. Ahlrichs , Phys. Chem. Chem. Phys. 2005, 7, 3297–3305; 16240044 10.1039/b508541a

[18b] F. Weigend , Phys. Chem. Chem. Phys. 2006, 8, 1057–1065; 16633586 10.1039/b515623h

[18c] A. Hellweg , C. Hättig , S. Höfener , W. Klopper , Theor. Chem. Acc. 2007, 117, 587–597;

[18d] F. Weigend , J. Comput. Chem. 2008, 29, 167–175; 17568435 10.1002/jcc.20702

[18e] A. V. Marenich , C. J. Cramer , D. G. Truhlar , J. Phys. Chem. B 2009, 113, 6378–6396; 19366259 10.1021/jp810292n

[18f] S. Grimme , J. Antony , S. Ehrlich , H. Krieg , J. Chem. Phys. 2010, 132, 154104; 20423165 10.1063/1.3382344

[18g] S. Grimme , S. Ehrlich , L. Goerigk , J. Comput. Chem. 2011, 32, 1456–1465; 21370243 10.1002/jcc.21759

[18h] H. Kruse , S. Grimme , J. Chem. Phys. 2012, 136, 154101; 22519309 10.1063/1.3700154

[18i] S. Grimme , J. G. Brandenburg , C. Bannwarth , A. Hansen , J. Chem. Phys. 2015, 143, 054107. 26254642 10.1063/1.4927476

[19] F. Neese , WIREs Comput. Mol. Sci. 2018, 8, e1327.

[20] C. Bannwarth , E. Caldeweyher , S. Ehlert , A. Hansen , P. Pracht , J. Seibert , S. Spicher , S. Grimme , WIREs Comput. Mol. Sci. 2021, 11, e1493.

[21] P. M. Zimmerman , J. Chem. Phys. 2013, 138, 184102. 23676024 10.1063/1.4804162

[22] T. Lu , F. Chen , J. Comput. Chem. 2012, 33, 580–592. 22162017 10.1002/jcc.22885

[23] J.-D. Chai , M. Head-Gordon , J. Chem. Phys. 2008, 128, 084106. 18315032 10.1063/1.2834918

[24a] G. Ghigo , S. Cagnina , A. Maranzana , G. Tonachini , J. Org. Chem. 2010, 75, 3608–3617; 20459136 10.1021/jo100367z

[24b] R. W. Jemison , D. G. Morris , Chem. Commun. 1969, 1226–1227.

[25] R. Ponce Ortiz , J. Casado , V. Hernández , J. T. López Navarrete , P. M. Viruela , E. Ortí , K. Takimiya , T. Otsubo , Angew. Chem. Int. Ed. 2007, 46, 9057–9061; 10.1002/anie.200703244 17960741

