# Supplementary material for: Sigmatropic [1,5] Carbon Shift of Transient C3 Ammonium Enolates
Source: Angew Chem Int Ed Engl. 2022 Jun 10;61(31):e202204378. doi: 10.1002/anie.202204378 (PMC9401041; doi:10.1002/anie.202204378)

Angewandte Chemie
Eine Zeitschrift der Gesellschaft Deutscher Chemiker

Supporting Information

Sigmatropic [1,5] Carbon Shift of Transient C3 Ammonium Enolates

*G. Wicker, R. Zhou, R. Schoch, J. Paradies**

Content

1 General information 4

1.1 Synthesis and techniques 4

1.2 Reagents and materials 4

1.3 Characterization 4

2 Cyclization reactions 7

2.1 Substrate synthesis 7

2.1.1 Syntheses of bromides for nucleophilic substitution 7

2.1.2 General procedure for the synthesis of primary amines by nucleophilic substitution (GP 1) 8

2.1.3 Synthesis of primary amines by nucleophilic aromatic substitution (GP 2) 10

2.1.4 General procedure for the synthesis of *N,N*-dialkylated 2'-aminoacetophenones via nucleophilic substitution (GP 3) 11

2.1.5 General procedure for the synthesis of *N,N*-dialkylated 2'-aminoacetophenones via nucleophilic aromatic substitution (GP 4) 14

2.1.6 General procedure for aldol condensations (GP 5) 22

2.2 Cyclization experiments 44

2.2.1 General Procedure for NMR-scale experiments (GP 6) 44

2.2.2 Lewis acid screening 45

2.2.3 General procedure for table run experiments (GP 7) 46

2.2.4 Representative procedure for 1 mmol scale 65

2.3 References 66

3 NMR Spectra 67

3.1 NMR spectra of precursors 67

3.2 NMR spectra of starting materials 91

3.3 NMR spectra of cyclization products 122

4 UV/Vis and luminescence spectra 144

5 Mechanistic studies and kinetic analysis 164

5.1.1 Isolation of ((1,1-dimethyl-2-phenyl-1,2,3,4-tetrahydroquinolin-1-ium-4-yl)oxy)tris(perfluorophenyl)borate (3) 164

5.1.2 General procedure for kinetic measurements 164

5.2 Control experiments 175

5.2.1 NMR scale experiment with radical scavenger 175

6 Crystallographic Data 176

7 Computational chemistry 186

1 General information

1.1 Synthesis and techniques

All preparations concerning air or moisture sensitive compounds were carried out in oven dried glassware under an atmosphere of inert gas (Argon 5.0, *Air Liquide*) employing both SCHLENK line techniques and an inert atmosphere glovebox manufactured by *Glovebox Systems*. Experiments conducted at elevated temperatures were carried out using an oil bath. For NMR scale experiments Teflon cap sealed J.YOUNG NMR tubes were used. Table run experiments were carried out in fresh crimp seal glass vials. Deuterated solvents were degassed by 3 freeze-pump-thaw cycles and stored over 3 Å molecular sieves. MeCN and MeOH were bought as HPLC-grade (≥ 99% purity) and used without further purification. Chloroform, pentane, and dichloromethane were bought as HPLC grade (≥ 99% purity), dispensed into STRAUS flasks equipped with YOUNG type Teflon stop-cocks, degassed by 3 freeze-pump-thaw cycles, and stored over 3 Å resp. 4 Å molecular sieves. Molecular sieves were activated at 280 °C under vacuum and stored under inert atmosphere. The following abbreviations for solvents were used: Ethyl acetate (EA), cyclohexane (CH), dichloromethane (DCM), *N,N*-dimethly formamide (DMF).

1.2 Reagents and materials

All commercially available chemicals were purchased from *ABCR*, *Acros*, *Alfa Aesar*, *Merck, Sigma Aldrich,* and *TCI Chemicals* and used without further purification unless stated otherwise. $B(C_6F_5)_3$ was purchased from BOULDER SCIENTIFIC COMPANY and used as received. Technical grade solvents for flash chromatography were distilled under reduced pressure prior to use. Silica gel for flash chromatography was purchased from *Merck* (Silica gel 60, 0.015-0.040 nm). Automated flash chromatography was performed on a *Büchi* Pure C810 Flash system using prepacked columns (*Büchi* FlashPure Ecoflex and FlashPure ID).

1.3 Characterization

Nuclear magnetic resonance spectroscopy (NMR)

NMR-spectra were recorded on a *Bruker* AV 300 (300 MHz), a *Bruker* AV 500 (500 MHz) or a *Bruker* Ascend 700 (700 MHz) as solutions. Chemical shifts are expressed in parts per million [ppm, δ] downfield from tetramethyl silane (TMS) and are referenced to the residual solvent signals of $CDCl_3$ (7.26 ppm for ^{1}H-NMR, 77.16 ppm for ^{13}C-NMR) and CD_2Cl_2 (5.32 ppm for ^{1}H-NMR and 53.84 ppm for ^{13}C-

NMR). ^{11}B-NMR, $^{1}H^{15}N$-HMBC-NMR and ^{19}F-NMR spectra are referenced to $BF_3{\cdot}OEt_2$, $NH_{3(l)}$ and $CFCl_3$, respectively. Data are reported as follows: chemical shift, multiplicity (s = singlet, d = doublet, dd = doublet of a doublet, ddd, = doublet of a doublet of a doublet, t = triplet, tt = triplet of a triplet, quart = quartet, m = multiplet, *etc.*), absolute values of coupling constants (*J*) [Hz] and integration. Exact assignment of signals was done under consideration of $^{1}H,^{1}H$-COSY, $^{1}H,^{13}C$-HSQC, $^{1}H,^{13}C$-HMBC, ^{1}H, ^{15}N-HMBC, DEPT135 and DEPTQ spectra. The following abbreviations were used: CH_3 = primary (RCH_3), CH_2 = secondary (R_2CH_2), CH = tertiary (R_3CH), C_q = quaternary (R_4C), H_{Ar} = aromatic hydrogen, H_{Alk} = alkylic hydrogen, H_{Ph} = phenylic hydrogen.

Mass spectrometry

All mass spectra were recorded on *Waters* Synapt 2G mass-spectrometer (electrospray-ionization – ESI) with a quadrupole-TOF analyzer. The molecular fragments are addressed as the ratio between mass and charge (m/z), the intensities as a percentaged value relative to the intensity of the base signal (100%). The abbreviation [M^+] refers to the molecule-ion.

Thin layer chromatography (TLC)

TLC was run on silica coated aluminum plates with fluorescence indicator (*Merck* Kieselgel 60 F254) and were analyzed by fluorescence.

X-ray single crystal structure analysis

The presented X-ray single crystal data were recorded on a *Bruker Venture D8* diffractometer. In case of *(2S,3R)*-**2a** a Cu K_α µ-source (λ=1.54178 Å) was applied, whereas for the other crystals a Mo K_α µ-source (λ=0.71073 Å) was used. Both radiations were monochromized by *Incoatec* multilayer Montel optics and a Photon III area detector was applied for data acquisition. All crystals were kept at 120 K during measurement.

Data processing was carried out using the *Bruker* APEX III software package: This includes SAINT for data integration and SADABS for a multi-scan absorption correction. Structure solution was obtained by direct methods and the refinement of the structures using full-matrix least squares method based on F^2 were achieved in SHELX.[SI1] All non-hydrogen-atoms were refined anisotropically and the hydrogen atom positions were refined at idealized positions riding on the carbon atoms with isotropic displacement parameters $U_{iso}(H)$=1.2 $U_{eq}(C)$ resp. 1.5 $U_{eq}(-CH_3)$ and C-H

bond lengths of 0.93-0.96 Å. All CH_3 hydrogen atoms were allowed to rotate but not to tip.

Crystallographic data have been deposited at the Cambridge Crystallographic Data Centre assigned to the deposition numbers 2127715-2127719. Copies are available free of charge via www.ccdc.cam.ac.uk.

UV/Vis-spectroscopy

UV/Vis-spectra were recorded on an *Agilent Technologies* Cary 50 UV-Vis-spectrometer from solutions of the respective compound in DCM (10^{-5} M). Spectra were plotted with ORIGINLAB OrignPro 2018.

Luminescence spectroscopy

Luminescence spectra were recorded on a *JASCO* FP-8300 fluorescence-spectrometer from solutions of the respective compound in DCM (10^{-5} M). Spectra were plotted with ORIGINLAB OriginPro 2018.

High performance liquid chromatography (HPLC)

For analytical HPLC at chiral stationary phase for determination of enantiomeric excess (ee) a *Varian* 920-LC was used with the following capillary columns: *Chiralcel* OJ-J, *Chiralcel* IA.

.

2 Cyclization reactions

2.1 Substrate synthesis

2.1.1 Syntheses of bromides for nucleophilic substitution

2.1.1.1 1-(1-Bromoethyl-1-*d*)benzene (**S1a**)

S1a was synthesized according to a modified literature procedure.[SI2] In a SCHLENK flask, acetophenone (1.17 ml, 10.0 mmol, 1.00 equiv.) was dissolved in EtOH (2.7 ml, 7.41 M) and cooled to 0 °C. $NaBD_4$ (134 mg, 3.20 mmol, 0.320 equiv.) was added slowly and the reaction mixture was stirred at 0 °C for 4.5 hours. Then, 30 ml saturated aqueous NH_4Cl solution were added, the aqueous phase was extracted thrice with 20 ml EA and the combined organic layers were dried over $MgSO_4$. Volatiles were removed under reduced pressure, the crude 1-Phenylethan-1-*d*-1-ol was obtained as a colorless oil (1.09 g, 8.87 mmol, 89%) and used without further purification.

1-Phenylethan-1-*d*-1-ol (1.09 g, 8.87 mmol, 1.00 equiv.) was stirred in a round bottom flask at 0 °C. Acetyl bromide (1.31 ml, 17.8 mmol, 2.00 equiv.) was added slowly and the reaction mixture was stirred overnight while heating up to room temperature. Then, 5 ml water were added, the aqueous phase was extracted thrice with 10 ml DCM and the combined organic layers were dried over $MgSO_4$. Volatiles were removed under reduced pressure, the product was obtained as slightly yellow oil (1.34 g, 7.20 mmol, 81%, 98.3% deuterium incorporation) and used without further purification.

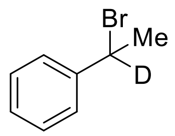

^{1}H-NMR: (500 MHz, 303 K, $CDCl_3$): δ = 7.46 – 7.43 (m, 2H, H_{Ar}), 7.37 – 7.33 (m, 2H, H_{Ar}), 7.31 – 7.27 (m, 1H, H_{Ar}), 2.05 (t, $^3J_{HD}$ = 0.9 Hz, 3H, CH_3).

2.1.1.2 1-(1-Bromoethyl)-4-chlorobenzene (**S1b**)

According to a modified literature procedure[SI3], 1-(4-chlorophenyl)ethan-1-ol (0.670 ml, 5.00 mmol, 1.00 equiv.) was dissolved in DCM (0.27 M), cooled to 0 °C and PBr_3 (0.14 ml, 3.35 mmol, 0.667 equiv.) was added. The reaction mixture was stirred at room temperature for 1 hour before 10 ml water were added. The organic layer was separated and filtrated through pad of SiO_2. The filtrate was dried over $MgSO_4$, and volatiles were removed under reduced pressure. The crude product was obtained as colorless oil (880 mg, 4.01 mmol, 80%) and used without further purification.

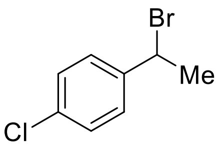

^{1}H-NMR: (700 MHz, 298 K, $CDCl_3$): δ = 7.38 – 7.36 (m, 2H, H_{Ar}), 7.32 – 7.30 (m, 2H, H_{Ar}), 5.17 (q, $^3J_{HH}$ = 6.9 Hz, 1H, C*H*CH_3), 2.03 (d, $^3J_{HH}$ = 6.9 Hz, 3H, CHCH_3).

NMR spectroscopic data is in good agreement with previously reported chemical shifts and signal patterns.[SI4]

2.1.1.3 1-(1-Bromoethyl)-4-(trifluoromethyl)benzene (**S1c**)

According to a modified literature procedure[SI3], 1-(4-(trifluoromethyl)phenyl)ethan-1-ol (2.03 ml, 13.2 mmol, 1.00 equiv.) was dissolved in DCM (0.27 M) and cooled to 0 °C. After addition of PBr_3 (0.38 ml, 8.81 mmol, 0.667 equiv.), the reaction mixture was warmed to room temperature and stirred for one hour. Then, 25 ml water and 25 ml of aqueous saturated $NaHCO_3$ solution were added. The organic phase was separated, dried over $MgSO_4$ and volatiles were removed under reduced pressure. The crude product was obtained as colorless liquid (2.09 g, 8.26 mmol, 63%) and used without further purification.

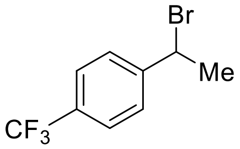

^{1}H-NMR: (700 MHz, 298 K, $CDCl_3$): δ = 7.62 – 7.59 (m, 2H, H_{Ar}), 7.57 – 7.53 (m, 2H, H_{Ar}), 5.20 (q, $^3J_{HH}$ = 6.9 Hz, 1H, C*H*CH_3), 2.05 (d, $^3J_{HH}$ = 7.0 Hz, 3H, CHCH_3).

NMR spectroscopic data is in good agreement with previously reported chemical shifts and signal patterns.[SI3]

2.1.2 General procedure for the synthesis of primary amines by nucleophilic substitution (GP 1)

A crimp seal glass vial was charged with 2’-aminoacetophenone (1.00 equiv.), the corresponding bromide (0.80 equiv.), K_2CO_3 (1.20 equiv.) and MeCN (1 M). The reaction mixture was stirred at 90 °C overnight, cooled to room temperature diluted with EA and solids were filtered off. Volatiles were removed under reduced pressure and the crude product was purified by flash chromatography (SiO_2, mixtures of EA and CH).

2.1.2.1 1-(2-((1-phenylethyl)amino)phenyl)ethan-1-one (*rac*-**S2a**)

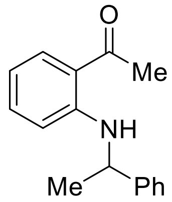

GP 1 starting from 2’-aminoacetophenone (5.00 mmol) and (1-bromoethyl)benzene, CH/EA 25:1, yellow oil (527 mg, 2.20 mmol, 55%).

^{1}H-NMR: (700 MHz, 298 K, $CDCl_3$): δ = 9.36 (bs, 1H, N*H*), 7.76 – 7.74 (m, 1H, H_{Ar}), 7.35 – 7.29 (m, 4H, H_{Ar}), 7.24 – 7.21 (m, 1H, H_{Ar}),

7.20 – 7.17 (m, 1H, H_{Ar}), 6.57 – 6.54 (m, 1H, H_{Ar}), 6.50 – 6.47 (m, 1H, H_{Ar}), 4.62 – 4.57 (m, 1H, C*H*(CH_3)), 2.62 (s, 3H, CO(CH_3)), 1.59 (d, $^3J_{HH}$ = 6.8 Hz, 3H, CH(CH_3)); **^{13}C-NMR** (176 MHz, 298 K, $CDCl_3$): δ = 201.2 (C_q), 150.1 (C_q), 144.9 (C_q), 134.5 (CH), 132.7 (CH), 128.8 (CH), 127.1 (CH), 125.9 (CH), 117.8 (C_q), 114.4 (CH), 113.4 (CH), 52.9 (CH), 28.2 (CH_3), 25.2 (CH_3); **^{15}N-NMR** (71 MHz, 298 K, $CDCl_3$): δ = 90.1.

2.1.2.2 1-(2-((1-Phenylethyl-1-*d*)amino)phenyl)ethan-1-one (*rac*-d_1-**S2a**)

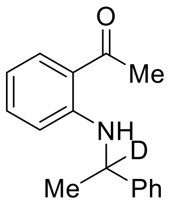

GP 1 starting from 2'-aminoacetophenone (2.00 mmol) and (1-bromoethyl-1-*d*)benzene (**S1a**), CH/EA 25:1, yellow oil (257 mg, 1.07 mmol, 67%). The product was directly used in **GP 3**.

^{1}H-NMR: (700 MHz, 298 K, $CDCl_3$): δ = 9.34 (bs, 1H, N*H*), 7.77 – 7.72 (m, 1H, H_{Ar}), 7.35 – 7.28 (m, 4H, H_{Ar}), 7.24 – 7.16 (m, 2H, H_{Ar}), 6.57 – 6.53 (m, 1H, H_{Ar}), 6.50 – 6.47 (m, 1H, H_{Ar}), 2.62 (s, 3H, COCH_3), 1.58 (s, 3H, CDCH_3).

2.1.2.3 1-(2-((1-(4-Chlorophenyl)ethyl)amino)phenyl)ethan-1-one (***rac*-S2b**)

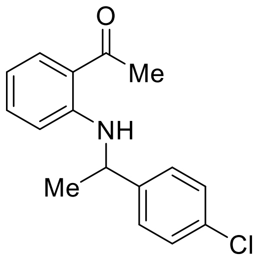

GP 1 starting from 2'-aminoacetophenone (5.00 mmol) and **S1b**, CH/EA 25:1, yellow oil (702 mg, 2.56 mmol, 64%).

^{1}H-NMR: (700 MHz, 298 K, $CDCl_3$): δ = 9.36 (bd, $^3J_{HH}$ = 5.0 Hz 1H, N*H*), 7.80 – 7.74 (m, 1H, H_{Ar}), 7.29 – 7.24 (m, 4H, H_{Ar}, overlap with solvent signal), 7.21 – 7.17 (m, 1H, H_{Ar}), 6.60 – 6.55 (m, 1H, H_{Ar}), 6.42 – 6.38 (m, 1H, H_{Ar}), 4.61 – 4.54 (m, 1H, C*H*CH_3), 2.62 (s, 3H, COCH_3), 1.57 (d, $^3J_{HH}$ = 6.8 Hz, CHCH_3); **^{13}C-NMR** (176 MHz, 298 K, $CDCl_3$): δ = 201.3 (C_q), 150.0 (C_q), 143. 6 (C_q), 135.0 (CH), 132.8 (CH), 132.6 (C_q), 129.0 (CH), 127.3 (CH), 117.9 (C_q), 114.7 (CH), 113.2 (CH), 52.3 (CH), 28.2 (CH_3, CO*C*H_3), 25.1 (CH_3, CHCH_3); **^{15}N-NMR** (71 MHz, 298 K, $CDCl_3$): δ = 89.4; **HRMS** (ESI+, MeOH) *m/z* $[M+Na]^+$ ($C_{16}H_{16}NONaCl$)calc. 296.0818, found 296.0818.

2.1.2.4 1-(2-((1-(4-(trifluoromethyl)phenyl)ethyl)amino)phenyl)ethan-1-one (*rac*-**S2c**)

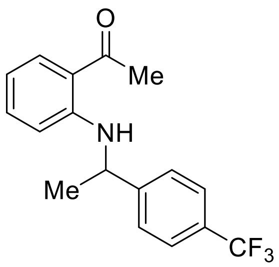

GP 1 starting from 2'-aminoacetophenone (5.00 mmol) and **S1c**, CH/EA 25:1, yellow solid (691 mg, 2.25 mmol, 56%).

^{1}H-NMR: (700 MHz, 298 K, $CDCl_3$): δ = 9.36 (bd, $^3J_{HH}$ = 5.1 Hz 1H, N*H*), 7.80 – 7.75 (m, 1H, H_{Ar}), 7.59 – 7.54 (m, 2H, H_{Ar}), 7.47 – 7.42 (m, 1H, H_{Ar}), 7.21 – 7.17 (m, 1H, H_{Ar}), 6.62 – 6.56 (m, 1H, H_{Ar}), 6.39 – 6.36 (m, 1H, H_{Ar}), 4.67 – 4.62 (m, 1H, C*H*CH_3), 2.63 (s, 3H, COCH_3), 1.60 (d, $^3J_{HH}$ = 6.8 Hz, 3H, CHCH_3); **^{13}C-NMR** (176 MHz, 298 K, $CDCl_3$):

δ = 201.4 (C_q), 149.9 (C_q), 149.2 (C_q), 135.1 (CH), 132.9 (CH), 129.4 (q, $^2J_{CF}$ = 32.3 Hz, C_q), 126.3 (CH), 125.9 (q, $^3J_{CF}$ = 3.71 Hz, CH), 124.3 (q, $^1J_{CF}$ = 271.9 Hz, CF_3), 118.0 (C_q), 114.9 (CH), 113.1 (CH), 52.6 (CH), 28.2 (CH_3, CO*C*H_3), 25.0 (CH_3, CH*C*H_3); **^{19}F-NMR** (282 MHz, 298 K, $CDCl_3$): δ = −62.4 (s, 3F); **^{15}N-NMR** (71 MHz, 298 K, $CDCl_3$): δ = 88.6; **HRMS** (ESI+, MeCN) *m/z* $[M+H]^+$ ($C_{17}H_{17}NOF_3$)calc. 308.1262, found 308.1255.

2.1.2.5 Synthesis of 1-(2-(methylamino)phenyl)ethan-1-one (**S2d**)

According to a modified literature procedure[SI5], a crimp seal glass vial was charged with 2'-aminoacetophenone (2.41 ml, 20.0 mmol, 1.00 equiv.), iodomethane (1.25 ml, 20 mmol, 1.00 equiv.), K_2CO_3 (2.76 mg, 20.0 mmol, 1.00 equiv.) and DMF (12.0 ml, 1.67 M). The reaction mixture was stirred at room temperature for 3 days. 40 ml water were added, and the aqueous phase was extracted four times with 40 ml EA. The organic phase was dried over Na_2SO_4, volatiles were removed under reduced pressure and the crude product was purified by flash chromatography (SiO_2, 40:1 CH/EA). **S2d** was obtained as yellow solid (1.45 g, 9.69 mmol, 50%).

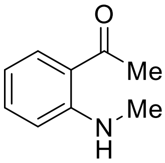

^{1}H-NMR: (700 MHz, 298 K, $CDCl_3$): δ = 8.80 (bs, 1H, N*H*), 7.76 – 7.73 (m, 1H, H_{Ar}), 7.41 – 7.36 (m, 1H, H_{Ar}), 6.71 – 6.68 (m, 1H, H_{Ar}), 6.62 – 6.57 (m, 1H, H_{Ar}), 2.81 (s, 3H, CH_3), 2.58 (s, 3H, CH_3).

NMR spectroscopic data is in good agreement with previously reported chemical shifts and signal patterns.[SI6]

2.1.3 Synthesis of primary amines by nucleophilic aromatic substitution (GP 2)

According to a modified literature procedure[SI7], a crimp seal glass vial was charged with 2'-fluoroacetophenone (1.00 equiv.), the primary amine (1.15 equiv.), K_2CO_3 (1.15 equiv.) and DMF (1 M). The reaction mixture was stirred at 140 °C for the indicated time and then, cooled to room temperature. The reaction mixture was washed with aqueous saturated NH_4Cl solution, and the aqueous phase was extracted thrice with EA. The organic phase was dried over Na_2SO_4, volatiles were removed under reduced pressure and the crude product was purified by flash chromatography (SiO_2, mixtures of CH/EA).

2.1.3.1 (*S*)-1-(2-((1-phenylethyl)amino)phenyl)ethan-1-one ((*S*)-**S2e**)

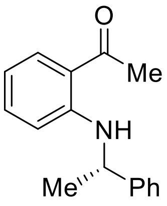

GP 2 starting from 2'-fluoroacetophenone (5.00 mmol) and (*S*)-1-phenylethan-1-amine, 3 d, CH/EA 40:1, yellow oil (662 mg, 2.77 mmol, 55%).

^{1}H-NMR: (700 MHz, 298 K, $CDCl_3$): δ = 9.35 (bd, $^3J_{HH}$ = 4.9 Hz 1H, N*H*), 7.76 – 7.74 (m, 1H, H_{Ar}), 7.34 – 7.29 (m, 4H, H_{Ar}), 7.23 – 7.20 (m, 1H, H_{Ar}), 7.19 – 7.17 (m, 1H, H_{Ar}), 6.56 – 6.53 (m, 1H, H_{Ar}), 6.48 – 6.46 (m, 1H, H_{Ar}), 4.62 – 4.57 (m, 1H, C*H*CH_3), 2.62 (s, 3H, COC*H*$_3$), 1.59 (d, $^3J_{HH}$ = 6.8 Hz, 3H, CHC*H*$_3$); **^{13}C-NMR** (176 MHz, 298 K, $CDCl_3$): δ = 201.2 (C_q), 150.2 (C_q), 145.0 (C_q), 135.0 (CH), 132.8 (CH), 128.8 (CH), 127.0 (CH), 125.9 (CH), 117.8 (C_q), 114.3 (CH), 113.3 (CH), 52.8 (CH, *C*HCH_3), 28.2 (CH_3, CO*C*H$_3$), 25.2 (CH_3, CH*C*H$_3$); **^{15}N-NMR** (71 MHz, 298 K, $CDCl_3$): δ = 90.5; **HRMS** (ESI+, MeCN) *m/z* $[M+Na]^+$ ($C_{16}H_{17}NONa$)calc. 262.1208, found 262.1198.

2.1.4 General procedure for the synthesis of *N,N*-dialkylated 2'-aminoacetophenones via nucleophilic substitution (GP 3)

A crimp seal glass vial was charged with the secondary amine (1.00 equiv.), methyl iodide (1.50 equiv.), K_2CO_3 (1.25 equiv.) and MeCN (1 M), and the reaction mixture was stirred at 90 °C for the indicated time. The reaction mixture was then diluted with EA, filtrated and volatiles were removed under reduced pressure. The crude product was purified by flash chromatography (SiO_2, mixtures of CH/EA).

2.1.4.1 1-(2-((1-(4-bromophenyl)ethyl)(methyl)amino)phenyl)ethan-1-one (*rac*-**S3a**)

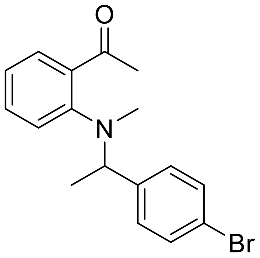

GP 2 starting from 2'-fluoroacetophenone (10 mmol) and 1-(4-bromophenyl)ethan-1-amine, 3 d, CH/EA 40:1, yellow oil (885 mg, 2.78 mmol, 28%). The product (2.78 mmol) was directly used in **GP 3**, 3 d, CH/EA 25:1, yellow oil (353 mg, 1.11 mmol, 11% over two steps).

^{1}H-NMR: (700 MHz, 298 K, $CDCl_3$): δ = 7.44 – 7.40 (m, 3H, H_{Ar}), 7.36 – 7.33 (m, 1H, H_{Ar}), 7.10 – 7.04 (m, 3H, H_{Ar}), 7.02 – 6.97 (m, 1H, H_{Ar}), 4.29 (bs, 1H, C*H*CH_3), 2.67 (s, 3H, COC*H*$_3$), 2.54 (s, 3H, NC*H*$_3$), 1.37 (d, $^3J_{HH}$ = 6.7 Hz, 3H, CHC*H*$_3$); **^{13}C-NMR** (176 MHz, 298 K, $CDCl_3$): δ = 204.7 (C_q), 150.6 (C_q), 140.7 (C_q), 137.0 (C_q), 131.5 (CH), 129.3 (CH), 128.8 (CH), 122.9 (CH), 121.9 (CH), 121.2 (C_q), 63.4 (CH, *C*HCH_3), 37.6 (CH_3, N*C*H$_3$), 30.4 (CH_3, CO*C*H$_3$), 18.8 (CH_3, CH*C*H$_3$) (One signal of an aromatic

carbon atom was not observed.); **^{15}N-NMR** (71 MHz, 298 K, $CDCl_3$): δ = 57.7; **HRMS** (ESI+, MeOH) *m/z* $[M+Na]^+$ ($C_{17}H_{18}NONaBr$) calc. 354.0469, found 354.0459.

2.1.4.2 (*S*)-1-(2-((1-(4-bromophenyl)ethyl)(methyl)amino)phenyl)ethan-1-one ((*S*)-**S3a**)

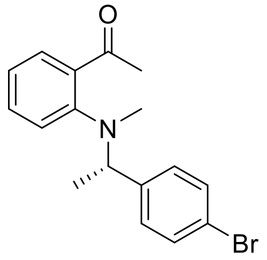

GP 2 starting from 2'-fluoroacetophenone (5 mmol) and (*S*)-1-(4-bromophenyl)ethan-1-amine, 3 d, CH/EA 40:1, yellow oil (624 mg, 1.96 mmol, 39%). The product (1.96 mmol) was directly used in **GP 3**, 3 d, CH/EA 25:1, yellow oil (413 mg, 1.24 mmol, 25% over two steps).

^{1}H-NMR: (700 MHz, 298 K, $CDCl_3$): δ = 7.43 – 7.40 (m, 3H, H_{Ar}), 7.36 – 7.33 (m, 1H, H_{Ar}), 7.09 – 7.05 (m, 3H, H_{Ar}), 7.01 – 6.98 (m, 1H, H_{Ar}), 4.28 (q, $^3J_{HH}$ = 6.8 Hz, 1H, C*H*CH_3), 2.66 (s, 3H, COC*H*$_3$), 2.53 (s, 3H, NC*H*$_3$), 1.37 (d, $^3J_{HH}$ = 6.8 Hz, 3H, CHC*H*$_3$); **^{13}C-NMR** (176 MHz, 298 K, $CDCl_3$): δ = 204.7 (C_q), 150.7 (C_q), 140.7 (C_q), 137.0 (C_q), 131.5 (CH), 129.3 (CH), 128.8 (CH), 122.9 (CH), 121.9 (CH), 121.1 (C_q), 63.4 (CH, *C*HCH_3), 37.6 (CH_3, N*C*H$_3$), 30.4 (CH_3, CO*C*H$_3$), 18.8 (CH_3, CH*C*H$_3$) (One signal of an aromatic carbon atom was not observed.); **^{15}N-NMR** (71 MHz, 298 K, $CDCl_3$): δ = 56.7; **HRMS** (ESI+, MeOH) *m/z* $[M+Na]^+$ ($C_{17}H_{18}NONaBr$) calc. 354.0469, found 354.0469.

2.1.4.3 1-(2-(Methyl(1-(4-(trifluoromethyl)phenyl)ethyl)amino)phenyl)ethan-1-one (*rac*-**S3b**)

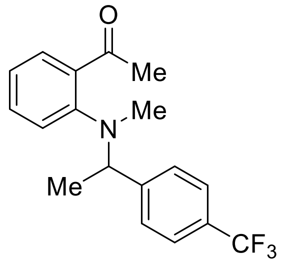

GP 3 starting from (*rac*-**S2c**) (2.25 mmol), 3 d, CH/EA 40:1, yellow oil (160 mg, 0.500 mmol, 22%)

^{1}H-NMR: (700 MHz, 298 K, $CDCl_3$): δ = 7.54 – 7.54 (m, 2H, H_{Ar}), 7.44 – 7.42 (m, 1H, H_{Ar}), 7.37 – 7.34 (m, 1H, H_{Ar}), 7.34 – 7.31 (m, 2H, H_{Ar}), 7.11 – 7.08 (m, 1H, H_{Ar}), 7.04 – 7.01 (m, 1H. H_{Ar}), 4.37 (q, $^3J_{HH}$ = 6.8 Hz, 1H, C*H*CH_3), 2.67 (s, 3H, CH_3, COC*H*$_3$), 2.55 (s, 3H, CH_3, NC*H*$_3$), 1.40 (d, $^3J_{HH}$ = 6.8 Hz, 3H, CHC*H*$_3$); **^{13}C-NMR** (176 MHz, 298 K, $CDCl_3$): δ = 204.6 (C_q), 150.5 (C_q), 146.0 (C_q), 137.1 (C_q), 131.5 (CH), 129.6 (q, $^2J_{CF}$ = 32.3 Hz, C_q), 128.8 (CH), 127.8 (CH), 125.4 (q, $^3J_{CF}$ = 3.8 Hz, CH), 124.3 (q, $^1J_{CF}$ = 271.0 Hz, CF_3), 123.5 (CH), 122.0 (CH), 63.6 (CH, *C*HCH_3), 38.0 (CH_3, N*C*H$_3$), 30.5 (CH_3, CO*C*H$_3$), 19.0 (CH_3, CH*C*H$_3$); **^{15}N-NMR** (71 MHz, 298 K, $CDCl_3$): δ = 56.0; **HRMS** (ESI+, MeCN) *m/z* $[M+H]^+$ ($C_{18}H_{19}NOF_3$) calc. 322.1419, found 322.1422.

2.1.4.4 1-(2-(Cinnamyl(methyl)amino)phenyl)ethan-1-one (**S3c**)

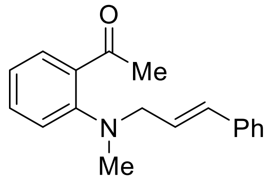

GP 3 starting from **S2d** (2.00 mmol, 1.00 equiv.) and cinnamyl bromide (1.60 mmol, 0.800 equiv.) in acetone, overnight, CH/EA 20:1, yellow oil (210 mg, 0.791 mmol, 40%).

^{1}H-NMR: (700 MHz, 298 K, $CDCl_3$): δ = 7.46 – 7.44 (m, 1H, H_{Ar}), 7.39 – 7.35 (m, 3H. H_{ar}), 7.33 – 7.30 (m, 2H, H_{Ar}), 7.26 – 7.20 (m, 1H, H_{Ar}), 7.09 – 7.06 (m, 1H, H_{Ar}), 7.00 – 6.97 (m, 1H, H_{Ar}), 6.54 (d, $^3J_{HH}$ = 15.8 Hz, 1H, NCH_2CHCH), 6.22 (dt, $^3J_{HH}$ = 15.8, 6.5 Hz, 1H, NCH_2CHCH), 3.79 (d, $^3J_{HH}$ = 6.4 Hz, 2H, NCH_2CHCH), 2.80 (s, 3H, $COCH_3$), 2.65 (s, 3H, NCH_3); **^{13}C-NMR** (176 MHz, 298 K, $CDCl_3$): δ = 204.0 (C_q), 151.3 (C_q), 136.8 (C_q), 134.0 (C_q), 133.3 (CH, NCH_2CHCH), 131.9 (CH), 129.6 (CH), 128.7 (CH), 127.8 (CH), 126.5 (CH), 125.8 (CH, NCH_2CHCH), 121.3 (CH), 119.0 (CH), 59.8 (CH_2), 41.1 (CH_3, $COCH_3$), 29.3 (CH_3, NCH_3) **^{15}N-NMR** (71 MHz, 298 K, $CDCl_3$): δ = 51.2; **HRMS** (ESI+, MeOH) *m/z* $[M+Na]^+$ ($C_{18}H_{19}NONa$) calc. 288.1364, found 288.1367.

2.1.4.5 1-(2-(Methyl(3-phenylprop-2-yn-1-yl)amino)phenyl)ethan-1-one (**S3d**)

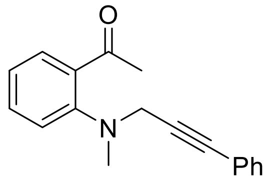

GP 3 starting from **S2d** (2.40 mmol, 1.00 equiv.) and (3-bromoprop-1-yn-1-yl)benzene (1.92 mmol, 0.800 equiv.) in acetone, CH/EA 20:1, yellow oil (236 mg, 0.896 mmol, 47%).

^{1}H-NMR: (700 MHz, 298 K, $CDCl_3$): δ = 7.57 – 7.55 (m, 1H, H_{Ar}), 7.52 – 7.47 (m, 2H. H_{ar}), 7.36 – 7.34 (m, 2H, H_{Ar}), 7.32 – 7.30 (m, 2H, H_{Ar}), 7.29 – 7.27 (m, 1H, H_{Ar}), 7.22 – 7.18 (m, 1H, H_{Ar}), 4.24 (s, 2H, NCH_2), 3.07 (s, 3H, NCH_3), 2.69 (s, 3H, $COCH_3$); **^{13}C-NMR** (176 MHz, 298 K, $CDCl_3$): δ = 203.5 (C_q), 147.5 (C_q), 134.6 (C_q), 132.3 (CH), 131.8 (CH), 129.8 (CH), 128.7 (CH), 128.5 (CH), 124.4 (CH), 122.5 (C_q), 87.3 (C_q), 82.8 (C_q), 48.0 (CH_2), 42.0 (CH_3, NCH_3), 29.5 (CH_3, $COCH_3$); **HRMS** (ESI+, MeOH) *m/z* $[M+H]^+$ ($C_{18}H_{18}NO$) calc. 264.1388, found 264.1370.

2.1.4.6 1-(2-((4-Fluorobenzyl)(methyl)amino)phenyl)ethan-1-one (**S3e**)

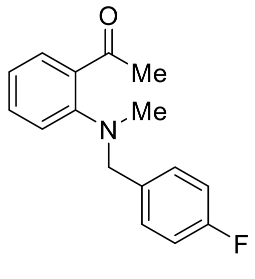

GP 3 starting from **S2d** (2.50 mmol, 1.00 equiv.) and 4-fluorobenzyl bromide (3.13 mmol, 1.25 equiv.), CH/EA 25:1, yellow oil (387 mg, 1.51 mmol, 60%)

^{1}H-NMR: (500 MHz, 303 K, $CDCl_3$): δ = 7.47 – 7.43 (m, 1H, H_{Ar}), 7.39 – 7.34 (m, 1H, H_{Ar}), 7.19 – 7.14 (m, 2H, H_{Ar}), 7.03 – 6.96 (m, 4H, H_{Ar}), 4.17 (s, 2H, CH_2), 2.66 (s, 3H, NCH_3), 2.65 (s, 3H, $COCH_3$); **^{13}C-NMR**

(126 MHz, 303 K, $CDCl_3$): δ = 203.9 (C_q), 162.3 (d, $^1J_{CF}$ = 245.5 Hz, C_q), 151.2 (C_q), 134.4 (C_q), 133.2 (d, $^4J_{CF}$ = 3.0 Hz, C_q), 131.9 (CH), 130.2 (d, $^3J_{CF}$ = 8.0 Hz, CH), 129.5 (CH), 121.6 (CH), 199.5 (CH), 115.4 (d, $^2J_{CF}$ = 21.2 Hz, CH)60.3 (CH_2), 41.8 (CH_3, NCH_3), 29.6 (CH_3, COCH_3); **^{19}F-NMR** (282 MHz, 298 K, $CDCl_3$): δ = −115.2 – 115.3 (m, 1F); **HRMS** (ESI+, MeOH) *m/z* $[M+Na]^+$ ($C_{16}H_{16}NOFNa$) calc. 280.1114, found 280.1118.

2.1.4.7 1-(2-(Dimethylamino)phenyl)ethan-1-one (**S3f**)

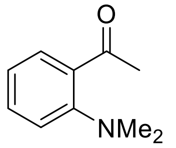

GP 3 starting from 2'-aminoacetophenone (676 mg, 5 mmol, 1.00 equiv.) and MeI (2.13 g, 15 mmol, 3.00 equiv.) and K_2CO_3 (1.73 g, 12.5 mmol, 2.5 equiv.). The product was obtained as yellow oil (780 mg, 4.77 mmol, 96%).

NMR spectroscopic data is in good agreement with previously reported chemical shifts and signal patterns.[SI8]

2.1.5 General procedure for the synthesis of *N,N*-dialkylated 2'-aminoacetophenones via nucleophilic aromatic substitution (GP 4)

According to a modified literature procedure[SI7], a crimp seal glass vial was charged with 2'-fluoroacetophenone (1.00 equiv.), the secondary amine (1.15 equiv.), K_2CO_3 (1.15 equiv.) and DMF (1 M). The reaction mixture was stirred at 140 °C for the indicated time and then, cooled to room temperature. The reaction mixture was washed with aqueous saturated NH_4Cl solution, and the aqueous phase was extracted thrice with EA. The organic phase was dried over Na_2SO_4, volatiles were removed under reduced pressure and the crude product was purified by flash chromatography (SiO_2, mixtures of CH/EA).

2.1.5.1 1-(2-((4-Methoxybenzyl)(methyl)amino)phenyl)ethan-1-one (**S3g**)

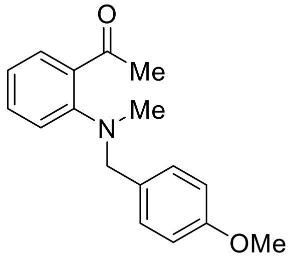

GP 4 starting from 2'-fluoroacetophenone (5 mmol) and 4-methoxy-*N*-methylbenzylamine, CH/EA 20:1, yellow oil (959 mg, 3.56 mmol, 71%).

^{1}H-NMR: (700 MHz, 298 K, $CDCl_3$): δ = 7.46 – 7.44 (m, 1H, H_{Ar}), 7.37 – 7.34 (m, 1H, H_{Ar}), 7.12 – 7.09 (m, 2H, H_{Ar}), 7.02 – 6.98 (m, 2H, H_{Ar}), 6.85 – 6.82 (m, 2H, H_{Ar}), 4.14 (s, 2H, CH_2), 3.79 (s, 3H, OCH_3), 2.66 (s, 3H, COCH_3), 2.65 (s, 3H, NCH_3); **^{13}C-NMR** (176 MHz, 298 K, $CDCl_3$): δ = 204.1 (C_q), 159.0 (C_q), 151.5 (C_q), 134.2 (C_q), 131.9 (CH), 129.8 (CH), 129.51 (C_q),

129.49 (CH), 121.3 (CH), 119.3 (CH), 113.9 (CH), 60.4 (CH_2), 55.4 (CH_3, O*C*H_3), 41.7 (CH_3, N*C*H_3), 29.5 (CH_3, CO*C*H_3); **^{15}N-NMR** (71 MHz, 298 K, $CDCl_3$): δ = 53.0; **HRMS** (ESI+, MeCN) *m/z* $[M+Na]^+$ ($C_{17}H_{19}NO_2Na$) calc. 292.1313, found 292.1312.

2.1.5.2 1-(2-(Ethyl(4-methoxybenzyl)amino)phenyl)ethan-1-one (**S3h**)

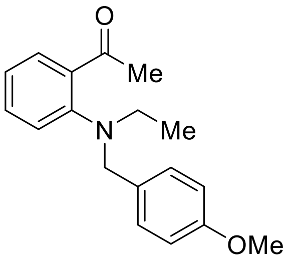

GP 4 starting from 2'-fluoroacetophenone (5 mmol) and 4-methoxy-*N*-ethylbenzylamine, CH/EA 25:1, yellow oil (757 mg, 2.67 mmol, 53%)

^{1}H-NMR: (700 MHz, 298 K, $CDCl_3$): δ = 7.44 – 7.41 (m, 1H, H_{Ar}), 7.36 – 7.33 (m, 1H, H_{Ar}), 7.11 – 7.09 (m, 2H, H_{Ar}), 7.04 – 7.01 (m, 2H, H_{Ar}), 6.83 – 6.80 (m, 2H, H_{Ar}), 4.15 (s, 2H, C*H_2*Ph), 3.79 (s, 3H, OC*H_3*), 3.02 (d, $^3J_{HH}$ = 7.1 Hz, 2H, C*H_2*CH_3), 2.68 (s, 3H, COC*H_3*), 1.00 (t, $^3J_{HH}$ = 7.0 Hz, 3H, CH_2C*H_3*); **^{13}C-NMR** (176 MHz, 298 K, $CDCl_3$): δ = 204.7 (C_q),158.9 (C_q), 149.9 (C_q), 136.4 (C_q), 131.4 (CH), 130.1 (CH), 129.7 (C_q), 129.3 (CH), 122.2 (CH), 121.5 (CH), 113.8 (CH), 57.2 (CH_2, C*H_2*Ph), 55.4 (CH_3, O*C*H_3), 47.3 (CH_2, N*C*H_2CH_3), 29.7 (CH_3, CO*C*H_3), 11.3 (CH_3, CH_2CH_3); **^{15}N-NMR** (71 MHz, 298 K, $CDCl_3$): δ = 62.6; **HRMS** (ESI+, MeCN) *m/z* $[M+Na]^+$ ($C_{18}H_{21}NO_2Na$) calc. 306.1470, found 306.1483.

2.1.5.3 1-(2-(Isobutyl(4-methoxybenzyl)amino)phenyl)ethan-1-one (**S3i**)

A round bottom flask was charged with 4-methoxybenzaldehyde (0.610 ml, 5.00 mmol, 1.00 equiv.), isobutyl amine (0.550 ml, 5.5 mmol, 1.10 equiv.) and 20 ml dichloroethane. A second round bottom flask charged with $NaBH_4$ (284 mg, 7.5 mmol, 1.5 equiv.) and 20 ml dichloroethane and was cooled to 0 °C, before acetic acid (1.29 ml, 22.5 mmol, 4.5 equiv.) was added. Then, the solution of 4-methoxybenzaldehyde and isobutyl amine was transferred to the cooled round bottom flask, and the reaction mixture was stirred at room temperature overnight. The reaction mixture was quenched by addition of 20 ml aqueous NaOH solution (1 M), the aqueous phase was extracted thrice with 20 ml DCM. The combined organic layers were washed with 20 ml water and 20 ml brine and dried over Na_2SO_4. Volatiles were removed under reduced pressure and the crude product (4-methoxy-*N*-isobutylbenzylamine) was used in the next step without further purification.

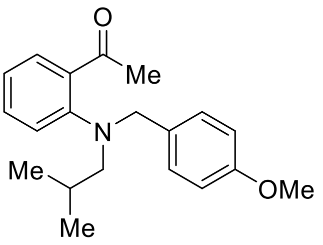

GP 4 starting from 2'-fluoroacetophenone (2.12 mmol) and 4-methoxy-*N*-isobutylbenzylamine, CH/EA 25:1, yellow oil (249 mg, 0.800 mmol, 16% over two steps)

^{1}H-NMR: (700 MHz, 298 K, $CDCl_3$): δ = 7.45 – 7.42 (m,1H, H_{Ar}), 7.33 – 7.29 (m, 1H, H_{Ar}), 6.99 – 6.96 (m, 3H, H_{Ar}), 6.96 – 6.93 (m, 1H, H_{Ar}), 6.79 (m, 2H, H_{Ar}), 4.18 (s, 2H, CH_2Ar), 3.77 (s, 3H, CH_3, OCH_3), 2.75 (d, $^3J_{HH}$ =6.9 Hz, 2H, $CH_2CH(CH_3)_2$), 2.67 (s, CH_3, $COCH_3$), 1.99 – 1.93 (m, 1H, $CH_2CH(CH_3)_2$), 0.85 (d, $^3J_{HH}$ = 6.6 Hz, 6H, $CH_2CH(CH_3)_2$); **^{13}C-NMR** (176 MHz, 298 K, $CDCl_3$): δ = 204.2 (C_q), 158.9 (C_q), 150.5 (C_q), 134.9 (C_q), 131.5 (CH), 130.0 (CH), 129.7 (CH), 129.2 (C_q), 121.2 (CH), 120.6 (CH), 113.7 (CH), 59.6 (CH_2, $CH_2CH(CH_3)_2$), 59.1 (CH_2, CH_2Ar), 55.3 (CH_3, OCH_3), 29.6 (CH_3, $COCH_3$), 26.2 (CH, $CH_2CH(CH_3)_2$), 21.0 (CH_3, $CH_2CH(CH_3)_2$); **^{15}N-NMR** (71 MHz, 298 K, $CDCl_3$): δ = 62.8; **HRMS** (ESI+, MeOH) *m/z* $[M+Na]^+$ ($C_{20}H_{25}NO_2Na$) calc. 334.1783, found 334.1790.

2.1.5.4 1-(2-((4-Methoxybenzyl)(methyl)amino)phenyl)propan-1-one (**S3j**)

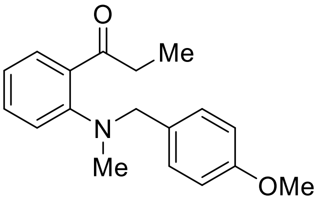

GP 4 starting from 2'-fluoropropiophenone (5 mmol) and 4-methoxy-*N*-methylbenzylamine, CH/EA 25:1, yellow oil (672 mg, 2.37 mmol, 47%).

^{1}H-NMR: (700 MHz, 298 K, $CDCl_3$): δ = 7.37 – 7.31 (m, 2H, H_{Ar}), 7.13 – 7.08 (m, 2H, H_{Ar}), 7.03 – 6.98 (m, 2H, H_{Ar}), 6.85 – 6.81 (m, 2H, H_{Ar}), 4.11 (s, 2H, CH_2Ar), 3.79 (s, 3H, OCH_3), 3.05 (q, $^3J_{HH}$ =7.3 Hz, 2H, $COCH_2CH_3$), 2.63 (s, 3H, NCH_3), 1.18 (t, $^3J_{HH}$ =7.3 Hz, 3H, $COCH_2CH_3$); **^{13}C-NMR** (176 MHz, 298 K, $CDCl_3$): δ = 208.3 (C_q), 159.0 (C_q), 151.0 (C_q), 134.8 (C_q), 131.3 (CH), 129.8 (CH), 129.6 (C_q), 129.0 (CH), 121.6 (CH), 119.4 (CH), 113.9 (CH), 60.5 (CH_2, CH_2Ar), 55.4 (CH_3, OCH_3), 41.6 (CH_3, NCH_3), 35.2 (CH_2, $COCH_2CH_3$), 9.0 (CH_3, $COCH_2CH_3$); **HRMS** (ESI+, MeOH) *m/z* $[M+Na]^+$ ($C_{18}H_{21}NO_2Na$) calc. 306.1470, found 306.1493.

2.1.5.5 Synthesis of 1-(2-fluoro-6-((4-methoxybenzyl)(methyl)amino)phenyl)ethan-1-one (**S3k**)

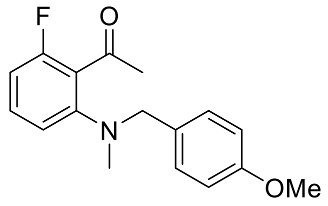

GP 1 method A starting from 2, 6-difluoroacetophenone (5.75 mmol) and 4-methoxy-*N*-methylbenzylamine, CH/ EA 20:1, yellow oil (731 mg, 2.54 mmol, 51%).

^{1}H-NMR (500 MHz, 303 K, $CDCl_3$): δ = 7.24-7.21 (m, 1H, H_{Ar}), 7.15-7.10 (m, 2H, H_{Ar}), 6.87-6.82 (m, 2H, H_{Ar}), 6.82-6.78 (m, 1H, H_{Ar}), 6.75-6.69 (m, 1H, H_{Ar}), 4.09 (s, 2H, NC*H*$_2$), 3.79 (s, 3H, OC*H*$_3$), 2.63 (s, 3H, NC*H*$_3$,) 2.59 (d, $^3J_{HH}$ = 1.1 Hz, 3H, COC*H*$_3$).

^{13}C-NMR (176 MHz, 298 K, $CDCl_3$): δ = 201.6 (C_q), 160.3-158.9 (d, $^1J_{CF}$ '= 247.8 Hz, C_q), 159.1 (C_q), , 152.0 (d, $^3J_{CF}$ = 6.4 Hz, C_q), 131.0 (d, $^3J_{CH}$ = 10.5 Hz, CH), 129.8 (CH), 129.6 (C_q), 124.0 (d, $^2J_{CF}$ = 17.3 Hz, C_q), 115.5 (d, $^4J_{CF}$ = 2.7 Hz, CH), 114.0 (CH), 109.1 (d, $^2J_{CF}$ = 22.2 Hz, CH), 60.6 (CH_2, N*C*H$_2$), 55.4 (CH_3, O*C*H$_3$), 41.4 (CH_3, N*C*H$_3$), 32.1 (CH_3, CO*C*H$_3$).

HRMS (EI) exact mass for $[M]^+$ ($C_{17}H_{18}FNO_2$) calc m/z 287.1317, found 287.1317.

2.1.5.6 Synthesis of 1-(4-fluoro-2-((4-methoxybenzyl)(methyl)amino)phenyl)ethan-1-one (**S3l**)

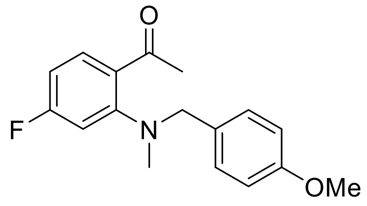

GP 1 method A starting from 2, 4-difluoroacetophenone (5.75 mmol) and 4-methoxy-*N*-methylbenzylamine, CH/ EA 20:1, yellow oil (843 mg, 2.92 mmol, 59%).

^{1}H-NMR (700 MHz, 298 K, $CDCl_3$): δ = 7.50-7.47 (m, 1H, H_{Ar}), 7.11-7.05 (m, 2H, H_{Ar}), 6.87-6.81 (m, 2H, H_{Ar}), 6.68-6.61 (m, 2H, H_{Ar}), 4.17 (s, 2H, NC*H*$_2$), 3.70 (s, 3H, OC*H*$_3$), 2.66 (s, 3H, NC*H*$_3$,) 2.62 (d, $^3J_{HH}$ = 1.1 Hz, 3H, COC*H*$_3$).

^{13}C-NMR (176 MHz, 298 K, $CDCl_3$): δ = 202.0 (C_q), 166.0-164.6 (d, $^1J_{CF}$ = 251.4 Hz, C_q), 159.2 (C_q), 153.8 (d, $^3J_{CF}$ = 9.6 Hz, C_q), 132.2 (d, $^3J_{CF}$ = 10.6 Hz, CH), 129.7 (CH), 129.2 (d, $^4J_{CF}$ = 2.7 Hz, CH), 128.9 (C_q), 114.1 (CH), 107.6 (d, $^2J_{CF}$ = 21.8 Hz, CH) 106.1 (d, $^2J_{CF}$ = 23.6 Hz, CH), 60.6 (CH_2, N*C*H$_2$), 55.4 (CH_3, O*C*H$_3$), 41.4 (CH_3, N*C*H$_3$), 32.1 (CH_3, CO*C*H$_3$).

HRMS (ESI) exact mass for $[M]^+$ ($C_{17}H_{18}FNO_2$) calc m/z 287.1317, found 287.1316.

2.1.5.7 Synthesis of 1-(5-fluoro-2-((4-methoxybenzyl)(methyl)amino)phenyl)ethan-1-one (**S3m**)

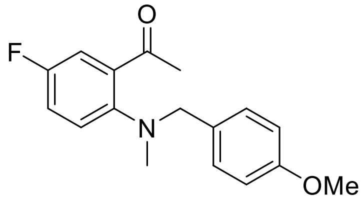

GP 1 method A starting from 2, 5-difluoroacetophenone (5.75 mmol) and 4-methoxy-*N*-methylbenzylamine, CH/ EA 20:1, orange oil (610 mg, 2.12 mmol, 42%).

^{1}H-NMR (700 MHz, 298 K, $CDCl_3$): δ = 7.14-7.13 (m, 1H, H_{Ar}), 7.11-7.08 (m, 2H, H_{Ar}), 7.08-7.04 (m, 1H, H_{Ar}), 7.02-6.98 (m, 1H, H_{Ar}), 6.85-6.81 (m, 2H, H_{Ar}), 4.04 (s, 2H, NCH_2), 3.79 (s, 3H, OCH_3), 2.67 (s, 3H, COCH_3) 2.61 (s, 3H, NCH_3).

^{13}C-NMR (176 MHz, 298 K, $CDCl_3$): δ = 203.0 (C_q), 159.1 (C_q), 158.9-157.5 (d, $^1J_{CF}$ = 242.9 Hz, C_q), 147.8 (d, $^4J_{CF}$ = 2.4 Hz, C_q), 136.7 (d, $^3J_{CF}$ = 5.8 Hz, C_q), 130.1 (CH), 129.3 (C_q), 121.8 (d, $^3J_{CF}$ = 7.6 Hz, CH), 118.3 (d, $^2J_{CF}$ = 22.3 Hz, CH), 115.7 (d, $^2J_{CF}$ = 23.5 Hz, CH), 113.9 (CH), 61.2 (CH_2, NCH_2), 55.4 (CH_3, OCH_3), 42.4 (CH_3, COCH_3), 29.7 (CH_3, NCH_3).

HRMS (EI) exact mass for $[M]^+$ ($C_{17}H_{18}FNO_2$) calc m/z 287.1317, found 287.1316.

2.1.5.8 Synthesis of 1-(2-((4-methoxybenzyl)(methyl)amino)-6-(trifluoromethyl)phenyl)ethan-1-one (**S3n**)

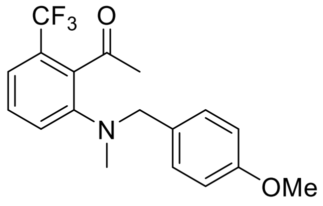

GP 1 method B starting from 2-fluoro-6-trifluoromethylacetophenone (1.65 mmol) and 4-methoxy-*N*-methylbenzylamine, CH/ EA 20:1, yellow oil (237 mg, 0.703 mmol, 24%).

^{1}H-NMR (700 MHz, 298 K, $CDCl_3$): δ = 7.49-7.37 (m, 3H, H_{Ar}), 7.21-7.12 (m, 2H, H_{Ar}), 6.91-6.83 (m, 2H, H_{Ar}), 3.95 (s, 2H, NCH_2), 3.82 (s, 3H, OCH_3), 2.60 (s, 3H, COCH_3) 2.57 (s, 3H, NCH_3).

^{13}C-NMR (176 MHz, 298 K, $CDCl_3$): δ = 204.1 (C_q), 159.2 (C_q), 151.3 (C_q), 138.3 (q, $^4J_{CF}$ = 1.9 Hz, C_q), 130.4 (CH), 129.7 (CH), 129.6 (C_q), 127.9 (q, $^2J_{CF}$ = 31.8 Hz, C_q), 126.1-121.4 (q, $^1J_{CF}$ = 274.0 Hz, C_q), 126.0 (CH), 122.0 (q, $^3J_{CF}$ = 4.9 Hz, C_q), 113.9 (CH), 61.8 (CH_2, NCH_2), 55.5 (CH_3, OCH_3), 42.0 (CH_3, COCH_3), 29.7 (q, $^5J_{CF}$ = 1.8 Hz, CH_3, NCH_3).

HRMS (ESI+, MeCN) exact mass for $[M+Na]^+$ ($C_{18}H_{18}F_3NO_2Na$): calc m/z 360.1187, found 360.1176

2.1.5.9 Synthesis of 1-(2-((4-methoxybenzyl)(methyl)amino)-5-(trifluoromethyl)phenyl)ethan-1-one (**S3o**)

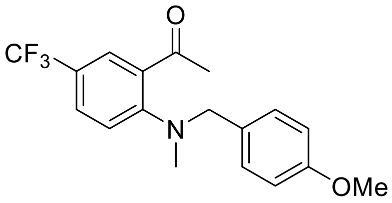

GP 1 method B starting from 2-fluoro-5-trifluoromethylacetophenone (5.00 mmol) and 4-methoxy-*N*-methylbenzylamine, CH/ EA 20:1, orange oil 1.21 g, 3.58 mmol, 72%).

^{1}H-NMR (500 MHz, 303 K, $CDCl_3$): δ = 7.72-7.70 (m, 1H, H_{Ar}), 7.56-7.54 (m, 1H, H_{Ar}), 7.10-7.05 (m, 2H, H_{Ar}), 7.02-6.98 (m, 1H, H_{Ar}), 6.87-6.82 (m, 2H, H_{Ar}), 4.28 (s, 2H, NCH_2), 3.79 (s, 3H, OCH_3), 2.75 (s, 3H, $COCH_3$) 2.63 (s, 3H, NCH_3).

^{13}C-NMR (176 MHz, 298 K, $CDCl_3$): δ = 202.0 (C_q), 159.3 (C_q), 153.4 (C_q), 138.3 (q, $^4J_{CF}$ = 1.9 Hz, C_q), 131.3 (CH), 129.4 (CH), 128.7 (q, $^3J_{CF}$ = 3.6 Hz, CH), 128.6 (C_q), 127.3 (q, $^3J_{CF}$ = 3.8 Hz, CH), 126.8-122.2 (q, $^1J_{CF}$ = 270.9 Hz, C_q), 121.5 (q, $^2J_{CF}$ = 33.2 Hz, C_q), 59.4 (CH_2, NCH_2), 55.5 (CH_3, OCH_3), 41.5 (CH_3, $COCH_3$), 29.0 (q, $^5J_{CF}$ = 1.8 Hz, CH_3, NCH_3).

HRMS (EI) exact mass for $[M]^+$ ($C_{18}H_{18}F_3NO_2$) calc m/z 337.1285, found 337.1287.

2.1.5.10 Synthesis of 2-((4-methoxybenzyl)(methyl)amino)benzaldehyde (**S3k**)

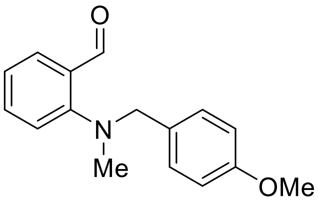

GP 4 starting from 2'-fluorobenzaldehyde (5 mmol) and 4-methoxy-*N*-methylbenzylamine, CH/EA 20:1, yellow oil (1.17 g, 4.57 mmol, 91%).

NMR spectroscopic data is in good agreement with previously reported chemical shifts and signal patterns.[SI8]

2.1.5.11 Synthesis of 1-(5-bromo-2-((4-methoxybenzyl)(methyl)amino)phenyl)ethan-1-one (**S3q**)

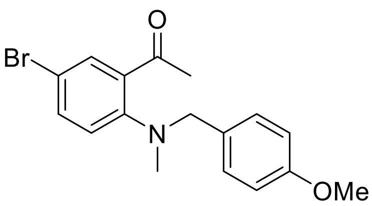

GP 1 method B starting from 5-bromo-2-fluoroacetophenone (5.00 mmol) and 4-methoxy-*N*-methylbenzylamine, CH/ EA 20:1, yellow oil 649 mg, 1.86 mmol, 37%).

^{1}H-NMR (500 MHz, 303 K, $CDCl_3$): δ = 7.56-7.53 (m, 1H, H_{Ar}), 7.44-7.40 (m, 1H, H_{Ar}), 7.10-7.05 (m, 2H, H_{Ar}), 6.87-6.81 (m, 3H, H_{Ar}), 4.12 (s, 2H, NCH_2), 3.79 (s, 3H, OCH_3), 2.64 (s, 3H, NCH_3) 2.63 (s, 3H, $COCH_3$).

^{13}C-NMR (176 MHz, 298 K, $CDCl_3$): δ = 202.4 (C_q), 159.2 (C_q), 150.4 (C_q), 135.4 (C_q), 134.5 (CH), 132.1 (CH), 129.8 (CH), 129.0 (C_q), 121.2 (CH), 114.0 (CH), 113.8 (C_q), 60.3 (CH_2, NCH_2), 55.4 (CH_3, OCH_3), 41.7 (CH_3, CH_3, NCH_3), 29.3 (CH_3, COCH_3).

HRMS (EI) exact mass for $[M]^+$ ($C_{17}H_{18}BrNO_2$) calc m/z 347.0515, found 347.0522.

2.1.5.12 Synthesis of 1-(2-((4-methoxybenzyl)(methyl)amino)-4-methylphenyl)ethan-1-one (**S3r**)

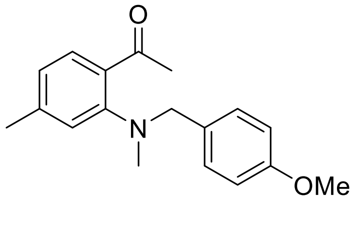

GP 1 method B starting from 2-fluoro-4-methylacetophenon (5.00 mmol) and 4-methoxy-*N*-methylbenzylamine, CH/ EA 20:1, yellow oil 630 mg, 2.22 mmol, 44%).

^{1}H-NMR (500 MHz, 303 K, $CDCl_3$): δ = 7.42-7.37 (m, 1H, H_{Ar}), 7.15-7.10 (m, 2H, H_{Ar}), 6.86-6.80 (m, 4H, H_{Ar}), 4.14 (s, 2H, NCH_2), 3.80 (s, 3H, OCH_3), 2.64 (s, 3H, COCH_3) 2.63 (s, CH_3, NCH_3), 2.33 (s, CH_3, CCH_3).

^{13}C-NMR (176 MHz, 298 K, $CDCl_3$): δ = 203.5 (C_q), 159.1 (C_q), 151.9 (C_q), 142.6 (C_q), 131.3 (C_q), 130.0 (CH), 129.9 (CH), 129.7 (C_q), 122.1 (CH), 119.9 (CH), 113.9 (CH), 60.4 (CH_2, NCH_2), 55.4 (CH_3, OCH_3), 41.7 (CH_3, NCH_3), 29.4 (CH_3, COCH_3), 21.9 (CH_3, CCH_3)

HRMS (ESI+, MeCN) exact mass for $[M+Na]^+$ ($C_{18}H_{21}NO_2Na$): calc m/z 306.1470, found 306.1466

2.1.5.13 Synthesis of 1-(2-((4-methoxybenzyl)(methyl)amino)-5-nitrophenyl)ethan-1-one (**S3s**)

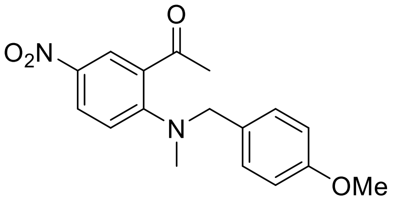

GP 1 method B starting from 2-fluoro-5-nitroacetophenone (5.00 mmol) and 4-methoxy-*N*-methylbenzylamine, CH/ EA 5:1, yellow oil 1.26 g, 4.01 mmol, 80%).

^{1}H-NMR (700 MHz, 298 K, $CDCl_3$): δ = 8.44-8.41 (m, 1H, H_{Ar}), 8.15-8.10 (m, 1H, H_{Ar}), 7.08-7.03 (m, 2H, H_{Ar}), 6.92-6.88 (m, 2H, H_{Ar}), 6.87-6.84 (m, 2H, H_{Ar}), 4.45 (s, 2H, NCH_2), 3.79 (s, 3H, OCH_3), 2.85 (s, 3H, NCH_3), 2.62 (s, CH_3, COCH_3).

^{13}C-NMR (176 MHz, 298 K, $CDCl_3$): δ = 199.4 (C_q), 159.4 (C_q), 154.7 (C_q), 138.1 (C_q), 128.8 (CH), 127.7 (C_q), 127.4 (CH), 127.3 (C_q), 127.0 (CH), 116.5 (CH), 114.5 (CH), 58.5 (CH_2, NCH_2), 55.5 (CH_3, OCH_3), 42.0 (CH_3, NCH_3), 28.2 (CH_3, COCH_3).

HRMS (ESI+, MeCN) exact mass for $[M+Na]^+$ ($C_{17}H_{18}N_2O_4Na$): calc m/z 337.1164, found 337.1152

2.1.5.14 Synthesis of 1-(4-methoxy-2-((4-methoxybenzyl)(methyl)amino)phenyl)ethan-1-one (**S3t**)

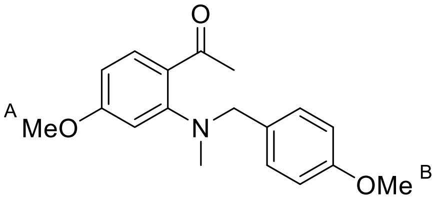

GP 1 method B starting from 2-fluoro-4-methoxyacetophenone (5.00 mmol) and 4-methoxy-*N*-methylbenzylamine, CH/ EA 10:1, yellow oil 1.18 g, 3.94 mmol, 79%).

^{1}H-NMR (700 MHz, 298 K, $CDCl_3$): δ = 7.55-7.53 (m, 1H, H_{Ar}), 7.13-7.09 (m, 2H, H_{Ar}), 6.86-6.81 (m, 2H, H_{Ar}), 6.52-6.50 (m, 1H, H_{Ar}), 6.48-6.47 (m, 1H, H_{Ar}), 4.17 (s, 2H, NCH_2), 3.80 (s, 3H, OCH_3^A), 3.79 (s, 3H, OCH_3^B), 2.65 (s, 3H, NCH_3), 2.63 (s, CH_3, $COCH_3$).

^{13}C-NMR (176 MHz, 298 K, $CDCl_3$): δ = 201.6 (C_q), 163.0 (C_q), 159.1 (C_q), 153.9 (C_q), 132.4 (CH), 129.8 (CH), 129.5 (C_q), 126.3 (C_q), 113.9 (CH), 105.7 (CH), 105.2 (CH), 60.3 (CH_2, NCH_2), 55.5 (CH_3, OCH_3^A), 55.5 (CH_3, OCH_3^B), 41.7 (CH_3, NCH_3), 29.1 (CH_3, $COCH_3$).

HRMS (EI) exact mass for $[M]^+$ ($C_{18}H_{21}NO_3$): calc m/z 299.1516, found 299.1506.

2.1.6 General procedure for aldol condensations (GP 5)

The ketone (1.00 equiv.), the corresponding aldehyde (1.10 equiv.) and NaOH (2.00 equiv.) were dissolved in MeOH (3.00 ml/mmol ketone) and stirred at room temperature or at 60 °C overnight. If the formation of a suspension was observed, the solid was filtered off, washed with MeOH, dried under reduced pressure, and used without further purification. Otherwise, water was added, and the resulting mixture was extracted thrice with DCM. The combined organic layers were dried over Na_2SO_4, and the solvent was removed under reduced pressure. The crude product was purified by flash chromatography (silica, mixtures of CH and EA).

2.1.6.1 (*E*)-1-(2-(Methyl(1-phenylethyl)amino)phenyl)-3-phenylprop-2-en-1-one (*rac*-**1a**)

GP 3 starting from *rac*-**S2a** (2.20 mmol), 3 d, CH/EA 40:1, yellow oil (260 mg, 1.03 mmol, 47%). The product (*rac*-1-(2-(methyl(1-phenylethyl)amino)phenyl)ethan-1-one) was directly used in the next step.

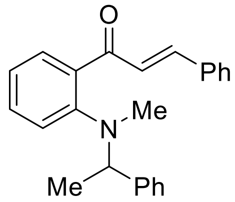

GP 5 starting from *rac*-1-(2-(methyl(1-phenylethyl)amino)phenyl)ethan-1-one (2.03 mmol) and benzaldehyde, room temperature, CH/EA 25:1, yellow oil (347 mg, 1.02 mmol, 99%)

^{1}H-NMR: (500 MHz, 303 K, CD_2Cl_2): δ = 7.65 – 7.58 (m, 3H, 2H$_{Ar}$, COCHC*H*), 7.52 – 7.49 (m, 1H, H$_{Ar}$), 7.43 – 7.38 (m, 4H, H$_{Ar}$), 7.36 (d, $^3J_{HH}$ =16.0 Hz, 7.19 – 7.17 (m, 5H, H$_{Ar}$), 7.11 – 7.05 (m, 2H, H$_{Ar}$), 4.45 (q, $^3J_{HH}$ =6.8 Hz, 1H, C*H*CH$_3$), 2.58 (s, 3H, NC*H*$_3$), 1.38 (d, $^3J_{HH}$ =6.8 Hz, 3H, CHC*H*$_3$); **^{13}C-NMR** (176 MHz, 298 K, $CDCl_3$): δ = 195.8 (C$_q$), 151.5 (C$_q$), 142.6 (CH, COCH*C*H), 141.4 (C$_q$), 135.2 (C$_q$), 135.0 (C$_q$), 131.6 (CH), 130.3 (CH), 130.1 (CH), 129.0 (CH), 128.5 (CH), 128.2 (CH), 127.5 (CH), 127.2 (CH), 127.2 (CH, COCHCH), 127.2 (CH, CO*C*HCH), 122.1 (CH), 121.0 (CH), 63.6 (CH, *C*HCH$_3$), 36.7 (CH$_3$, N*C*H$_3$), 18.1 (CH$_3$, CH*C*H$_3$); **^{15}N-NMR** (71 MHz, 298 K, $CDCl_3$): δ = 59.2; **HRMS** (ESI+, MeOH) *m/z* $[M+Na]^+$ ($C_{24}H_{23}NONa$) calc. 364.1677, found 364.1686.

HPLC (OJ-H, 15 °C, heptane/i-PrOH: 90/10, flow rate: 0.7 ml/min, 388 nm).

Chromatogram : GWI-421-1_channel5

System : LC_920

Acquired : 19.10.2020 14:39:49
Processed : 21.10.2020 15:32:02
Printed : 21.10.2020 15:32:44

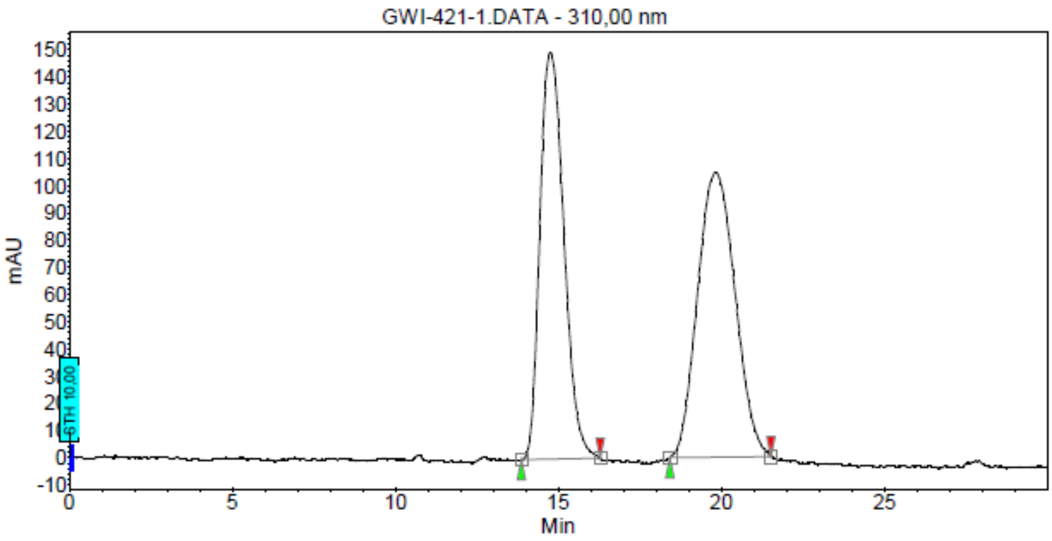

Peak results :

Index	Name	Time [Min]	Quantity [% Area]	Height [mAU]	Area [mAU.Min]	Area % [%]
1	UNKNOWN	14.75	47.71	149.5	127.6	47.712
2	UNKNOWN	19.80	52.29	104.9	139.8	52.288
Total			100.00	254.4	267.3	100.000

2.1.6.2 (*E*)-1-(2-(methyl(1-phenylethyl-1-*d*)amino)phenyl)-3-phenylprop-2-en-1-one (d_1-*rac*-**1a**)

GP 3 starting from d_1-*rac*-**S2a** (1.07 mmol), 5 d, CH/EA 40:1, yellow oil (116 mg, 0.456 mmol, 43%). The product (*rac*-1-(2-(methyl(1-phenylethyl-1-d)amino)phenyl)ethan-1-one) was directly used in the next step.

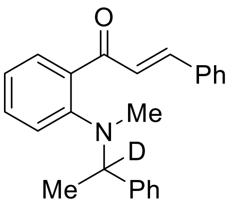

GP 5 starting from *rac*-1-(2-(methyl(1-phenylethyl-1-d)amino)phenyl)ethan-1-one (116 mg, 0.456 mmol, 1.00 equiv.) and benzaldehyde, room temperature, CH/EA 25:1, yellow oil (151 mg, 0.441 mmol, 97%)

^{1}H-NMR: (700 MHz, 298 K, CD_2Cl_2): δ = 7.61 – 7.55 (m, 3H, 2H_{Ar}, COCHC*H*), 7.49 – 7.46 (m, 1H, H_{Ar}), 7.41 – 7.36 (m, 4H, H_{Ar}), 7.34 (d, $^3J_{HH}$ =16.0 Hz, 1H, COC*H*CH), 7.17 – 7.12 (m, 5H, H_{Ar}), 7.07 – 7.04 (m, 1H, H_{Ar}), 7.04 – 7.01 (m, 1H, H_{Ar}), 2.55 (s, 3H, NCH_3), 1.36 (3H, CDCH_3); **^{13}C-NMR** (176 MHz, 298 K, $CDCl_3$): δ = 195.7 (C_q), 151.9 (C_q), 142.6 (CH, COCHCH), 141.9 (C_q), 135.6 (C_q), 135.4 (C_q), 131.7 (C_q),

130.5 (CH), 130.5 (CH), 130.2 (CH), 129.3 (CH), 128.7 (CH), 128.4 (CH), 127.8 (CH), 127.6 (CH), 127.4 (CH), 122.2 (CH), 121.3 (CH), 63.4 (C_q, *C*DCH3), 36.8 (CH_3, N*C*H3), 18.0 (CH_3, CD*C*H3); **^{15}N-NMR** (71 MHz, 298 K, $CDCl_3$): δ = 59.1; **HRMS** (ESI+, MeOH) *m/z* $[M+Na]^+$ ($C_{24}H_{22}DNONa$) calc. 365.1740, found 365.1738.

2.1.6.3 (*S,E*)-1-(2-(Methyl(1-phenylethyl)amino)phenyl)-3-phenylprop-2-en-1-one ((*S*)-**1a**)

GP 3 starting from (*S*)-**S2e** (1.07 mmol), 5 d, CH/EA 40:1, yellow oil (168 mg, 0.663 mmol, 53%). The product ((*S*)-1-(2-(methyl(1-phenylethyl)amino)phenyl)ethan-1-one) was directly used in the next step.

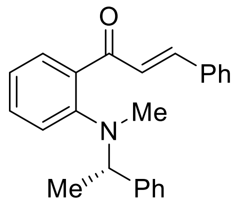

GP 5 starting from (*S*)-1-(2-(methyl(1-phenylethyl)amino)phenyl)ethan-1-one (168 mg, 0.663 mmol, 1.00 equiv.) and benzaldehyde, room temperature, CH/EA 40:1, yellow oil (226 mg, 0.662 mmol, 99%).

^{1}H-NMR: (700 MHz, 298 K, CD_2Cl_2): δ = 7.63 – 7.58 (m, 3H, 2H_{Ar}, COCHC*H*), 7.51 – 7.49 (m, 1H, H_{Ar}), 7.43 – 7.38 (m, 4H, H_{Ar}), 7.36 (d, $^3J_{HH}$ =16.0 Hz, 1H, COC*H*CH), 7.19 – 7.15 (m, 5H, H_{Ar}), 7.10 – 7.07 (m, 1H, H_{Ar}), 7.07 – 7.05 (m, 1H, H_{Ar}), 4.45 (q, $^3J_{HH}$ =6.8 Hz, 1H, C*H*CH3), 2.57 (s, 3H, NCH3), 1.38 (d, $^3J_{HH}$ =6.9 Hz, 3H, CHC*H*3); **^{13}C-NMR** (176 MHz, 298 K, $CDCl_3$): δ = 195.7 (C_q), 152.0 (C_q), 142.5 (CH, COCH*C*H), 142.0 (C_q), 135.6 (C_q), 135.5 (C_q), 131.7 (CH), 130.5 (CH), 129.3 (CH), 128.7 (CH), 128.5 (CH), 127.8 (CH), 127.6 (CH), 127.4 (CH), 122.2 (CH), 121.4 (CH), 63.8 (CH, C*H*CH3), 37.0 (CH_3, N*C*H3), 18.3 (CH_3, CH*C*H3); **^{15}N-NMR** (71 MHz, 298 K, $CDCl_3$): δ = 59.4; **HRMS** (ESI+, MeOH) *m/z* $[M+Na]^+$ ($C_{24}H_{23}NONa$) calc. 364.1677, found 364.1697.

HPLC (OJ-H, 15 °C, heptane/i-PrOH: 90/10, flow rate: 0.7 ml/min, 388 nm).

Chromatogram : GWI-442-1_channel5

System : LC_920

Acquired : 19.10.2020 15:37:48
Processed : 21.10.2020 15:35:15
Printed : 21.10.2020 15:35:46

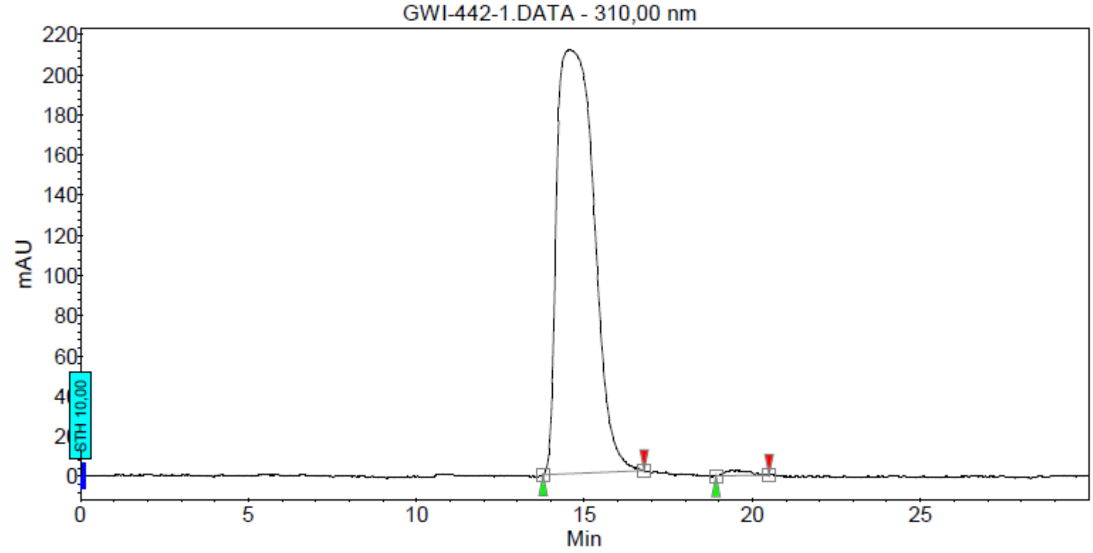

Peak results :

Index	Name	Time [Min]	Quantity [% Area]	Height [mAU]	Area [mAU.Min]	Area % [%]
1	UNKNOWN	14,57	99,18	211,2	269,7	99,176
2	UNKNOWN	19,40	0,82	2,7	2,2	0,824
Total			100,00	213,9	271,9	100,000

2.1.6.4 (*E*)-1-(2-((4-Methoxybenzyl)(methyl)amino)phenyl)-3-phenylprop-2-en-1-one (**1b**)

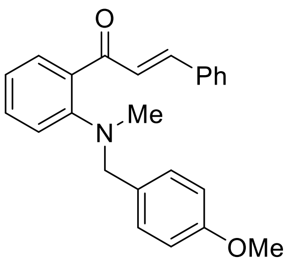

GP 5 starting from **S3f** (959 mg, 3.56 mmol, 1.00 equiv.) and benzaldehyde, room temperature, CH/EA 20:1, yellow solid (1.01 g, 2.81 mmol, 79%).

^{1}H-NMR: (700 MHz, 298 K, $CDCl_3$): δ = 7.69 (d, $^3J_{HH}$ =15.9 Hz, 1H, COCHC*H*), 7.59 – 7.55 (m, 3H, H_{Ar}), 7.45 – 7.39 (m, 5H, 4H_{Ar}, COC*H*CH), 7.12 – 7.09 (m, 2H, H_{Ar}), 7.09 – 7.06 (m, 1H, H_{Ar}), 7.06 – 7.02 (m, 1H, H_{Ar}), 6.74 – 6.70 (m, 2H, H_{Ar}), 4.18 (s, 2H, CH_2), 3.74 (s, 3H, OCH_3), 2.67 (s, 3H, NCH_3); **^{13}C-NMR** (176 MHz, 298 K, $CDCl_3$): δ = 195.4 (C_q), 158.9 (C_q), 152.0 (C_q), 142.6 (CH, COCH*C*H), 135.3 (C_q), 133.2 (C_q), 132.0 (CH), 130.5 (CH), 130.3 (CH), 129.72 (CH), 129.66 (C_q), 129.0 (CH), 128.5 (CH), 126.7 (CH, CO*C*HCH), 121.3 (CH),

119.0 (CH), 113.8 (CH); 60.2 (CH_2), 55.3 (CH_3, O*C*H_3), 41.8 (CH_3, N*C*H_3); **HRMS** (ESI+, MeCN) *m/z* $[M+Na]^+$ ($C_{24}H_{23}NO_2Na$) calc. 380.1626, found 380.1631.

2.1.6.5 (*E*)-1-(2-((4-Methoxybenzyl)(methyl)amino)phenyl)-3-(4-methoxyphenyl)prop-2-en-1-one (**1c**)

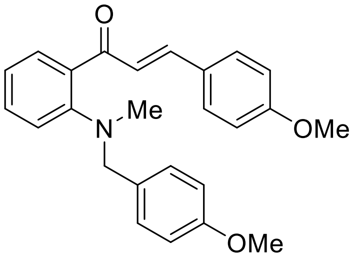

GP 5 starting from **S3g** (350 mg, 1.30 mmol, 1.00 equiv.) and 4-methoxybenzaldehyde, 60 °C, CH/EA 20:1, yellow solid (332 mg, 0.857 mmol, 66%).

¹H-NMR (700 MHz, 298 K, $CDCl_3$): δ = 7.63 (d, $^3J_{HH}$ = 15.9 Hz, 1H, COCHC*H*), 7.55 – 7.53 (m, 1H, H_{Ar}), 7.53 – 7.50 (m, 2H, H_{Ar}), 7.41 – 7.38 (m, 1H, H_{Ar}), 7.28 (d, $^3J_{HH}$ = 15.9 Hz, COC*H*CH), 7.13 – 7.10 (m, 2H, H_{Ar}), 7.07 – 7.05 (m, 1H, H_{Ar}), 7.04 – 7.01 (m, 1H, H_{Ar}), 6.93 -6.90 (m, 2H, H_{Ar}), 6.75 – 6.72 (m, 2H, H_{Ar}), 4.18 (s, 2H, CH_2), 3.86 (s, 3H, OCH_3), 3.75 (s, 3H, OCH_3), 2.67 (s, 3H, NCH_3); **¹³C-NMR** (176 MHz, 298 K, $CDCl_3$): δ = 195.6 (C_q), 161.5 (C_q), 158.9 (C_q), 151.9 (C_q), 142.8 (CH, COCH*C*H), 133.4 (C_q), 131.7 (CH), 130.4 (CH), 130.2 (CH), 129.8 (C_q), 129.7 (CH), 128.0 (C_q), 124.6 (CH, CO*C*HCH), 121.1 (CH), 118.9 (CH), 114.5 (CH), 113.8 (CH), 60.2 (CH_2), 55.6 (CH_3, O*C*H_3), 55.3 (CH_3, O*C*H_3), 41.6 (CH_3, N*C*H_3); **HRMS** (ESI+, MeCN) *m/z* $[M+Na]^+$ ($C_{25}H_{25}NO_3Na$) calc. 410.1732, found 410.1727.

2.1.6.6 (*E*)-1-(2-((4-Methoxybenzyl)(methyl)amino)phenyl)-3-(4-methoxyphenyl)prop-2-en-1-one (**1d**)

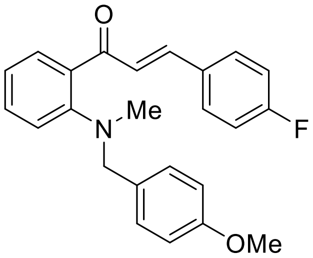

GP 5 starting from **S3g** (311 mg, 1.3 mmol, 1.00 equiv.) and 4-fluorobenzaldehyde, room temperature (Prior to purification the crude product was dissolved in 5 ml methanol, washed with saturated aqueous solution of $NaHSO_3$ (25 mL). The aqueous phase was diluted with 25 ml H_2O and extracted with 25 ml of a mixture of CH/EA (10% EA), then the organic phase was dried over Na_2SO_4, and volatiles were removed under reduced pressure.), CH/EA 20:1, yellow oil (313 mg, 0.834 mmol, 64%)

¹H-NMR (700 MHz, 298 K, $CDCl_3$): δ = 7.64 (d, $^3J_{HH}$ = 16.0 Hz, 1H, COCHC*H*), 7.57 – 7.52 (m, 3H, H_{Ar}), 7.43 – 7.40 (m, 1H, H_{Ar}), 7.34 (d, $^3J_{HH}$ = 15.9 Hz, COC*H*CH), 7.11 – 7.07 (m, 5H, H_{Ar}), 7.06 – 7.03 (m, 1H, H_{Ar}), 6.74 – 6.71 (m, 1H, H_{Ar}), 4.17 (s, 2H, CH_2), 3.75 (s, 3H, OCH_3), 2.67 (s, 3H, NCH_3); **¹³C-NMR** (176 MHz, 298 K, $CDCl_3$): δ = 195.1 (C_q), 164.0 (d, $^1J_{CF}$ = 251.2 Hz, C_q), 159.0 (C_q), 152.1 (C_q), 141.3 (CH,

COCHCH), 133.1 (C_q), 132.1 (CH), 131.6 (d, $^4J_{CF}$ = 3.2 Hz, C_q), 130.5 (CH), 130.3 (d, $^3J_{CF}$ = 8.5 Hz, CH), 129.7 (CH), 129.6 (C_q), 126.5 (CH), 124.6 (CH, CO*C*HCH), 121.3 (CH), 119.0 (CH), 116.1 (d, $^2J_{CF}$ = 21.9 Hz, CH), 113.8 (CH), 60.3 (CH_2), 55.3 (CH_3, O*C*H_3), 41.8 (CH_3, N*C*H_3); **HRMS** (ESI+, MeOH) *m/z* $[M+Na]^+$ ($C_{24}H_{22}NO_2FNa$) calc. 398.1532, found 398.1522.

2.1.6.7 (*E*)-1-(2-((4-methoxybenzyl)(methyl)amino)phenyl)-2-methyl-3-phenylprop-2-en-1-one (**1e**)

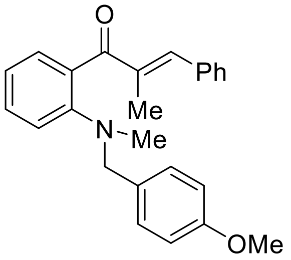

GP 5 starting from **S3j** (368 mg, 1.30 mmol, 1.00 equiv.) and benzaldehyde, 60 °C, CH/EA 25:1, yellow oil (479 mg, 1.29 mmol, 99%)

^{1}H-NMR (700 MHz, 298 K, $CDCl_3$): δ = 7.41 – 7.35 (m, 5H, H_{Ar}), 7.35 – 7.31 (m, 1H, H_{Ar}), 7.31 – 7.29 (m, 1H, H_{Ar}), 7.21 (s, 1H, COC(CH_3)C*H*), 7.12 – 7.08 (m, 2H, H_{Ar}), 7.08 – 7.06 (m, 1H, H_{Ar}), 7.03 – 7.00 (m, 1H, H_{Ar}), 6.75 – 6.73 (m, 2H, H_{Ar}), 4.12 (s, 2H, CH_2), 3.75 (s, 3H, OC*H*$_3$), 2.64 (bs, 3H, NC*H*$_3$), 2.22 (bs, 3H, COC(C*H*$_3$)); **^{13}C-NMR** (176 MHz, 298 K, $CDCl_3$): δ = 201.5 (C_q), 158.8 (C_q), 151.2 (C_q), 142.6 (CH, COC(CH_3)*C*H), 137.7 (C_q, CO*C*(CH_3)), 136.2 (C_q), 132.9 (C_q), 130.7 (CH), 130.3 (C_q), 130.0 (CH), 129.7 (CH), 128.7 (CH), 128.5 (CH), 121.0 (CH), 119.2 (CH), 113.8 (CH), 59.9 (CH_2), 55.3 (CH_3, O*C*H_3), 41.0 (CH_3, N*C*H_3), 13.6 (CH_3, COC(*C*H_3)); **HRMS** (ESI+, MeOH) *m/z* $[M+Na]^+$ ($C_{25}H_{25}NO_2Na$) calc. 394.1783, found 394.1792.

2.1.6.8 1-(2-((4-methoxybenzyl)(methyl)amino)phenyl)-3-methylbut-2-en-1-one (**1f**)

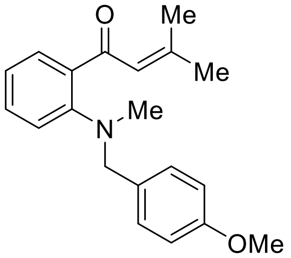

According to a modified literature procedure,[SI9] **S3k** (992 mg, 3.89 mmol, 1.00 equiv.) was dissolved in 20 ml THF and cooled to −78 °C. Then, 2-methylpropenyl magnesium bromide (0.5 M in THF, 11.7 ml, 5.83 mmol, 1.5 equiv.) was added. The reaction mixture was stirred at −78 °C for 3 hours and quenched with 10 ml saturated aqueous NH_4Cl solution. The aqueous layer was extracted thrice with 10 ml Et_2O. The organic layer was washed thrice with 10 ml H_2O and once with 10 ml brine, dried over Na_2SO_4 and volatiles were removed under reduced pressure. After purification by automated flash chromatography (SiO_2, CH/EA) the product (1-(2-((4-methoxybenzyl)(methyl)amino)phenyl)-3-methylbut-2-en-1-ol) was directly used in the next step.

The slightly yellow oil (1-(2-((4-methoxybenzyl)(methyl)amino)phenyl)-3-methylbut-2-en-1-ol, 915 mg, 2.94 mmol) was dissolved in DCM. After addition of MnO_2 (9 g), the reaction mixture was stirred under reflux for 18 hours. The reaction mixture was filtered through a plug of celite, and volatiles were removed under reduced pressure. Flash chromatography (SiO_2, CH/EA 25:1) afforded the product as yellow oil (96 mg, 0.310 mmol, 8% over two steps).

^{1}H-NMR (700 MHz, 298 K, $CDCl_3$): δ = 7.48 – 7.45 (m, 1H, H_{Ar}), 7.35 – 7.32 (m, 1H, H_{Ar}), 7.17 – 7.14 (m, 2H, H_{Ar}), 7.00 – 6.95 (m, 2H, H_{Ar}), 6.84 – 6.81 (m, 2H, H_{Ar}), 6.66 – 6.65 (m, 1H, COC*H*), 4.15 (bs, 2H, CH_2), 3.79 (s, 3H, OCH_3), 2.62 (bs, 3H, NCH_3), 2.22 (d, $^4J_{HH}$ = 0.8 Hz, CH_3^A), 1.96 (d, d, $^4J_{HH}$ = 0.6 Hz, CH_3^B); **^{13}C-NMR** (176 MHz, 298 K, $CDCl_3$): δ = 196.1 (C_q), 151.6 (C_q), 159.0 (C_q), 154.9 (C_q), 134.8 (C_q), 131.4 (CH), 130.2 (CH), 129.7 (CH), 125.4 (CH, CO*C*H), 121.0 (CH), 118.6 (CH), 113.9 (CH), 60.2 (CH_2), 55.5 (CH_3, OCH_3), 41.7 (CH_3, NCH_3), 28.1 (CH_3, CH_3^B), 21.2 (CH_3, CH_3^A); **HRMS** (ESI+, MeOH) *m/z* $[M+Na]^+$ ($C_{20}H_{23}NONa$) calc. 332.1626, found 332.1608.

2.1.6.9 (*E*)-1-(2-((4-Methoxybenzyl)(methyl)amino)phenyl)-4,4-dimethylpent-2-en-1-one (**1g**)

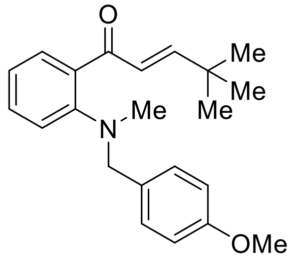

GP 5 starting from **S3g** (350 mg, 1.30 mmol, 1.00 equiv.) and pivalaldehyde, 60 °C, CH/EA 20:1, yellow oil (313 mg, 0.928 mmol, 71%).

^{1}H-NMR (700 MHz, 273 K, $CDCl_3$): δ = 7.47 – 7.45 (m, 1H, H_{Ar}), 7.38 – 7.35 (m, 1H, H_{Ar}), 7.12 – 7.09 (m, 2H, H_{Ar}), 7.01 – 6.98 (m, 2H, H_{Ar}), 6.96 (d, $^3J_{HH}$ = 15.9 Hz, 1H, COCHC*H*), 6.83 – 6.80 (m, 2H, H_{Ar}), 6.68 (d, $^3J_{HH}$ = 15.9 Hz, COC*H*CH), 4.11 (s, 2H, CH_2), 3.79 (s, 3H, OCH_3), 2.62 (s, 3H, NCH_3), 1.10 (s, 9H, C(CH_3)$_3$); **^{13}C-NMR** (176 MHz, 273 K, $CDCl_3$): δ = 196.9 (C_q), 158.9 (C_q), 158.0 (CH, COCH*C*H), 151.7 (C_q), 132.9 (C_q), 131.9 (CH), 130.5 (CH), 129.8 (CH), 129.7 (C_q), 125.1 (CH), 121.0 (CH), 118.8 (CH), 113.8 (CH), 60.42 (CH_2), 55.5 (CH_3, OCH_3), 41.2 (CH_3, NCH_3), 34.2 (C_q, *C*(CH_3)$_3$), 29.1 (CH_3, C(CH_3)$_3$); **^{15}N-NMR** (71 MHz, 273 K, $CDCl_3$): δ = 54.2; **HRMS** (ESI+, MeOH) *m/z* $[M+Na]^+$ ($C_{22}H_{27}NO_2Na$) calc. 360.1939, found 360.1945.

2.1.6.10 (*E*)-3-(furan-2-yl)-1-(2-((4-Methoxybenzyl)(methyl)amino)phenyl)prop-2-en-1-one (**1h**)

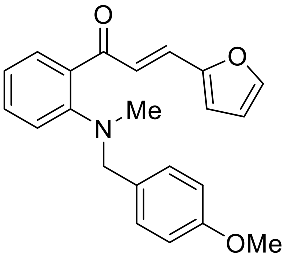

GP 5 starting from **S3g** (350 mg, 1.30 mmol, 1.00 equiv.) and furfural, room temperature, CH/EA 25:1, yellow oil (401 mg, 1.15 mmol, 89%).

^{1}H-NMR (700 MHz, 298 K, $CDCl_3$): δ = 7.56 – 7.52 (m, 2H, H_{Ar}), 7.45 (d, $^3J_{HH}$ = 15.7 Hz, 1H, COCHC*H*), 7.41 – 7.38 (m, 1H, H_{Ar}), 7.35 (d, $^3J_{HH}$ = 15.7 Hz, COC*H*CH), 7.18 – 7.15 (m, 2H, H_{Ar}), 7.08 – 7.05 (m, 1H, H_{Ar}), 7.04 – 7.00 (m, 1H, H_{Ar}), 6.77 – 6.74 (m, 2H, H_{Ar}), 6.77 – 6.74 (m, 2H, H_{Ar}), 6.66 – 6.64 (m, 1H, H_{Ar}), 6.52 – 6.50 (m, 1H, H_{Ar}), 4.18 (s, 2H, CH_2), 3.76 (s, 3H, OC*H*$_3$), 2.65 (s, 3H, NC*H*$_3$); **^{13}C-NMR** (176 MHz, 298 K, $CDCl_3$): δ = 194.9 (C_q), 158.9 (C_q), 152.10 (C_q), 142.05 (C_q), 144.7 (CH, COCH*C*H), 133.3 (C_q), 131.9 (CH), 130.4 (CH), 129.8 (C_q), 129.7 (CH), 128.8 (CH), 124.5 (CH, CO*C*HCH), 121.2 (CH), 118.9 (CH), 115.4 (CH), 113.7 (CH), 112.7 (CH), 60.2 (CH_2), 55.3 (CH_3, O*C*H$_3$), 41.9 (CH_3, N*C*H$_3$); **HRMS** (ESI+, MeOH) *m/z* $[M+Na]^+$ ($C_{22}H_{21}NO_3Na$) calc. 370.1422, found 370.1419.

2.1.6.11 (*E*)-1-(2-((4-Methoxybenzyl)(methyl)amino)phenyl)-3-(thiophen-2-yl)prop-2-en-1-one (**1i**)

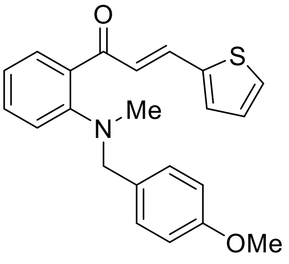

GP 5 starting from **S3g** (350 mg, 1.30 mmol, 1.00 equiv.) and 2-thiophenecarbaldehyde, 60 °C, CH/EA 25:1, yellow oil (419 mg, 1.15 mmol, 89%).

^{1}H-NMR (700 MHz, 298 K, $CDCl_3$): δ = 7.71 (d, $^3J_{HH}$ = 15.6 Hz, 1H, COCHC*H*), 7.56 – 7.53 (m, 1H, H_{Ar}), 7.42 – 7.38 (m, 2H, H_{Ar}), 7.30 – 7.28 (m, 1H, H_{Ar}), 7.24 (d, $^3J_{HH}$ = 15.6 Hz, COC*H*CH), 7.16 – 7.12 (m, 2H, H_{Ar}), 7.09 – 7.05 (m, 2H, H_{Ar}), 7.05 – 7.01 (m, 1H, H_{Ar}), 6.77 – 6.75 (m, 2H, H_{Ar}), 4.17 (s, 2H, CH_2), 3.75 (s, 3H, OC*H*$_3$), 2.67 (s, 3H, NC*H*$_3$); **^{13}C-NMR** (176 MHz, 298 K, $CDCl_3$): δ = 194.8 (C_q), 158.9 (C_q), 152.0 (C_q), 140.8 (C_q), 135.1 (CH, COCH*C*H), 133.2 (C_q), 131.9 (CH), 131.5 (CH), 130.4 (CH), 129.74 (CH), 129.70 (C_q), 128.41 (CH), 128.38 (CH), 125.7 (CH, CO*C*HCH), 121.3 (CH), 119.0 (CH), 113.9 (CH), 60.2 (CH_2), 55.3 (CH_3, O*C*H$_3$), 41.8 (CH_3); **^{15}N-NMR** (71 MHz, 298 K, $CDCl_3$): δ = 54.5; **HRMS** (ESI+, MeOH) *m/z* $[M+Na]^+$ ($C_{22}H_{21}NO_2SNa$) calc. 386.1191, found 386.1190.

2.1.6.12 (*E*)-1-(2-((4-Methoxybenzyl)(methyl)amino)phenyl)-3-(pyridin-2-yl)prop-2-en-1-one (**1j**)

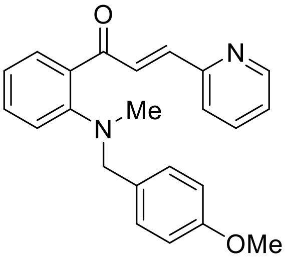

GP 5 starting from **S3g** (350 mg, 1.30 mmol, 1.00 equiv.) and 2-pyridinecarbaldehyde, room temperature, filtration, yellow solid (241 mg, 0.672 mmol, 52%).

^{1}H-NMR (700 MHz, 298 K, $CDCl_3$): δ = 8.71 – 8.68 (m, 2H, H_{Ar}), 7.91 (d, $^3J_{HH}$ = 15.6 Hz, 1H, COCHC*H*), 7.74 – 7.71 (m, 1H, H_{Ar}), 7.66 (d, $^3J_{HH}$ = 15.6 Hz, COC*H*CH), 7.18 – 7.15 (m, 2H, H_{Ar}), 7.58 – 7.56 (m, 1H, H_{Ar}), 7.47 – 7.44 (m, 1H, H_{Ar}), 7.43 – 7.39 (m, 1H, H_{Ar}), 7.30 – 7.27 (m, 1H, H_{Ar}), 7.19 – 7.16 (m, 2H, H_{Ar}), 7.10 – 7.06 (m, 1H, H_{Ar}), 7.05 – 7.01 (m, 1H, H_{Ar}), 6.70 – 6.67 (m, 2H, H_{Ar}), 4.19 (s, 2H, CH_2), 3.73 (s, 3H, OC*H*$_3$), 2.65 (s, 3H, NC*H*$_3$); **^{13}C-NMR** (176 MHz, 298 K, $CDCl_3$): δ = 195.4 (C_q), 158.9 (C_q), 153.9 (C_q), 152.3 (C_q), 150.2 (CH), 140.7 (CH, CO*C*HCH), 133.1 (C_q), 132.2 (CH), 129.8 (C_q), 130.54 (CH, COCHC*H*), 130.46 (CH), 129.9 (CH), 129.7 (C_q), 124.8 (CH), 124.1 (CH), 121.3 (CH), 118.9 (CH), 113.7 (CH), 60.2 (CH_2), 55.3 (CH_3, O*C*H$_3$), 42.1 (CH_3, N*C*H$_3$); **HRMS** (ESI+, MeOH) *m/z* $[M+Na]^+$ ($C_{23}H_{22}N_2O_2Na$) calc. 381.1579, found 381.1582.

2.1.6.13 (E)-1-(2-(ethyl(4-methoxybenzyl)amino)phenyl)-3-phenylprop-2-en-1-one (**1k**)

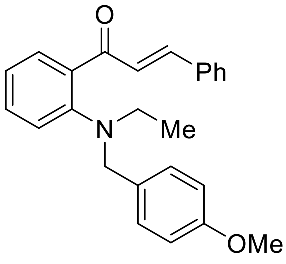

GP 5 starting from **S3h** (368 mg, 1.30 mmol, 1.00 equiv.) and benzaldehyde, room temperature, CH/EA 25:1, yellow oil (436 mg, 1.17 mmol, 90%)

^{1}H-NMR (700 MHz, 298 K, $CDCl_3$): δ = 7.65 (d, $^3J_{HH}$ = 15.9 Hz, 1H, COCHC*H*), 7.61 – 7.54 (m, 3H, H_{Ar}), 7.45 (d, $^3J_{HH}$ = 15.9 Hz, COC*H*CH), 7.43 – 7.37 (m, 4H, H_{Ar}), 7.12 – 7.05 (m, 4H, H_{Ar}), 6.73 – 6.68 (m, 2H, H_{Ar}), 4.17 (s, 2H, CH_2Ar), 3.74 (s, 3H, OC*H*$_3$), 3.03 (q, $^3J_{HH}$ = 6.9 Hz, 2H, C*H*$_2$CH$_3$), 1.01 (t, $^3J_{HH}$ = 6.9 Hz. 3H, CH$_2$C*H*$_3$); **^{13}C-NMR** (176 MHz, 298 K, $CDCl_3$): δ = 195.6 (C_q), 168.8 (C_q), 150.5 (C_q), 142.1 (CH, COCH*C*H), 135.4 (C_q), 135.2 (C_q), 131.6 (CH), 130.4 (CH), 130.4 (CH), 130.2 (CH), 129.9 (CH), 129.9 (C_q), 129.0 (CH), 128.5 (CH), 126.8 (CH, CO*C*HCH), 122.1 (CH), 121.1 (CH), 113.7 (CH), 56.9 (CH_2, *C*H$_2$Ar), 55.3 (CH_3, O*C*H$_3$), 47.2 (CH_2, *C*H$_2$CH$_3$), 11.5 (CH_3, CH$_2$*C*H$_3$); **^{15}N-NMR** (71 MHz, 298 K, $CDCl_3$): δ = 64.9; **HRMS** (ESI+, MeOH) *m/z* $[M+Na]^+$ ($C_{25}H_{25}NO_2Na$) calc. 394.1783, found 394.1792.

2.1.6.14 (*E*)-1-(2-(isobutyl(4-methoxybenzyl)amino)phenyl)-3-phenylprop-2-en-1-one (**1l**)

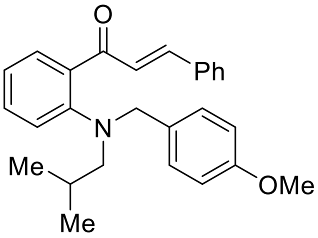

GP 5 starting from **S3i** (249 mg, 0.800 mmol, 1.00 equiv.) and benzaldehyde, room temperature, CH/EA 25:1, yellow oil (280 mg, 0.701 mmol, 88%)

^{1}H-NMR (700 MHz, 298 K, $CDCl_3$): δ = 7.68 (d, $^3J_{HH}$ = 15.9 Hz, 1H, COCHC*H*), 7.58 – 7.54 (m, 2H, H_{Ar}), 7.54 – 7.51 (m, 1H, H_{Ar}), 7.41 (d, $^3J_{HH}$ = 15.9 Hz, COC*H*CH), 7.40 – 7.36 (m, 4H, H_{Ar}), 7.05 – 7.01 (m, 2H, H_{Ar}), 7.01 – 6.97 (m, 2H, H_{Ar}), 6.72 – 6.68 (m, 2H, H_{Ar}), 4.20 (s, 2H, CH_2Ar), 3.74 (s, 3H, OCH_3), 2.77 (d, $^3J_{HH}$ = 6.9 Hz, 2H, $CH_2CH(CH_3)_2$), 1.95 (sept, $^3J_{HH}$ = 6.6 Hz, 1H, $CH_2CH(CH_3)_2$)), 0.83 (d, $^3J_{HH}$ = 6.6 Hz. 6H, $CH_2CH(CH_3)_2$)); **^{13}C-NMR** (176 MHz, 298 K, $CDCl_3$): δ = 195.7 (C_q), 158.8 (C_q), 151.0 (C_q), 142.8 (CH, COCH*C*H), 135.3 (C_q), 13.2 (C_q), 131.5 (CH), 130.5 (CH), 130.3 (CH), 130.0 (CH), 129.5 (C_q), 129.0 (CH), 128.6 (CH), 126.8 (CH, CO*C*HCH), 121.2 (CH), 120.3 (CH), 113.6 (CH), 59.7 (CH_2, $CH_2CH(CH_3)_2$), 58.8 (CH_2, *C*H_2Ar), 55.3 (CH_3, OCH_3), 26.2 (CH, $CH_2CH(CH_3)_2$), 21.1 (CH_3, $CH_2CH(CH_3)_2$); **HRMS** (ESI+, MeOH) *m/z* $[M+Na]^+$ ($C_{25}H_{25}NO_2Na$) calc. 394.1783, found 394.1792.

2.1.6.15 (*E*)-1-(2-((1-(4-chlorophenyl)ethyl)(methyl)amino)phenyl)-3-phenylprop-2-en-1-one (rac-**1m**)

GP 3 starting from (*rac*-**S2b**) (2.45 mmol), overnight, CH/EA 25:1, yellow oil (220 mg, 0.764 mmol, 31%). The product *rac*-1-(2-((1-(4-chlorophenyl)-ethyl)(methyl)amino)phenyl)ethan-1-one was directly used in the next step.

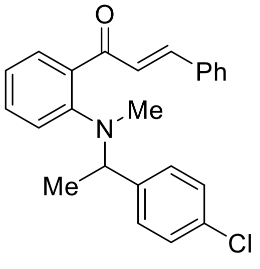

GP 5 starting from *rac*-1-(2-((1-(4-chlorophenyl)ethyl)(methyl)-amino)phenyl)ethan-1-one (220 mg, 0.764 mmol, 1.00 equiv.) and benzaldehyde, room temperature, CH/EA 40:1, yellow oil (262 mg, 0.697 mmol, 91%).

^{1}H-NMR: (700 MHz, 298 K, CD_2Cl_2): δ = 7.64 (d, $^3J_{HH}$ =16.0 Hz, 1H, COCHC*H*), 7.58 – 7.55 (m, 2H, H_{Ar}), 7.55 – 7.52 (m, 1H, H_{Ar}), 7.43 – 7.38 (m, 4H, H_{Ar}), 7.32 (d, $^3J_{HH}$ =16.0 Hz, 1H, COC*H*CH), 7.14 – 7.09 (m, 3H, H_{Ar}), 7.09 – 7.06 (m, 2H, H_{Ar}), 7.03 – 7.00 (m, 1H, H_{Ar}), 4.40 (q, $^3J_{HH}$ =6.8 Hz, 1H, C*H*CH_3), 2.56 (s, 3H, NCH_3), 1.36 (d, $^3J_{HH}$ =6.9 Hz, 3H, CHCH_3); **^{13}C-NMR** (176 MHz, 298 K, $CDCl_3$): δ = 195.8 (C_q), 151.3 (C_q), 142.8 (CH, COCH*C*H), 139.9 (C_q), 135.4 (C_q), 134.1 (C_q), 132.9 (C_q), 131.6 (CH), 130.5 (CH), 130.1 (CH), 129.1 (CH), 128.9 (CH), 128.5 (CH),

128.4 (CH), 127.2 (CH, COC*H*CH), 122.5 (CH), 121.2 (CH), 63.3 (CH, C*H*CH_3), 36.5 (CH_3, NCH_3), 18.0 (CH_3, CHCH_3); **^{15}N-NMR** (71 MHz, 298 K, $CDCl_3$): δ = 58.7; **HRMS** (ESI+, MeOH) *m/z* $[M+Na]^+$ ($C_{24}H_{22}NONaCl$) calc. 398.1288, found 398.1283.

2.1.6.16 (*E*)-1-(2-((1-(4-Bromophenyl)ethyl)(methyl)amino)phenyl)-3-phenylprop-2-en-1-one (*rac*-**1n**)

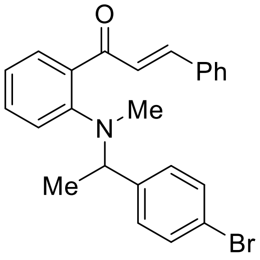

GP 5 starting from *rac*-**S3a** (412 mg, 1.24 mmol, 1.00 equiv.) and benzaldehyde, room temperature, CH/EA 25:1, yellow oil (349 mg, 0.830 mmol, 67%).

^{1}H-NMR: (700 MHz, 298 K, CD_2Cl_2): δ = 7.63 (d, $^3J_{HH}$ =16.0 Hz, 1H, COCHC*H*), 7.58 – 7.55 (m, 2H, H_{Ar}), 7.55 – 7.52 (m, 1H, H_{Ar}), 7.42 – 7.38 (m, 4H, H_{Ar}), 7.32 (d, $^3J_{HH}$ =16.0 Hz, 1H, COC*H*CH), 7.29 – 7.26 (m, 2H, H_{Ar}), 7.13 – 7.09 (m, 1H, H_{Ar}), 7.03 – 7.00 (m, 3H, H_{Ar}), 4.38 (q, $^3J_{HH}$ =6.8 Hz, 1H, C*H*CH_3), 2.56 (s, 3H, NCH_3), 1.35 (d, $^3J_{HH}$ =6.8 Hz, 3H, CHCH_3); **^{13}C-NMR** (176 MHz, 298 K, $CDCl_3$): δ = 195.9 (C_q), 151.3 (C_q), 142.9 (CH, COCH*C*H), 140.5 (C_q), 135.4 (C_q), 135.1 (C_q), 131.6 (CH), ;131.4 (CH), 130.5 (CH), 130.1 (CH), 129.2 (CH), 129.1 (CH), 128.5 (CH), 127.2 (CH), 122.5 (CH), 121.2 (CH), 121.0 (C_q), 63.3 (CH, *C*HCH_3), 36.6 (CH_3, NCH_3), 18.0 (CH_3, *C*HCH_3); **^{15}N-NMR** (71 MHz, 298 K, $CDCl_3$): δ = 58.8; **HRMS** (ESI+, MeOH) *m/z* $[M+Na]^+$ ($C_{24}H_{22}NONaBr$) calc. 442.0782, found 442.0779.

HPLC (IA, 15 °C, heptane/i-PrOH: 98/2, flow rate: 0.7 ml/min, 295 nm).

Chromatogram : GWI-705-5_channel5

System : LC920_2
Method : NOT DEFINED
User : AKP

Acquired : 22.10.2021 13:48:02
Processed : 22.10.2021 14:42:25
Printed : 26.10.2021 12:39:50

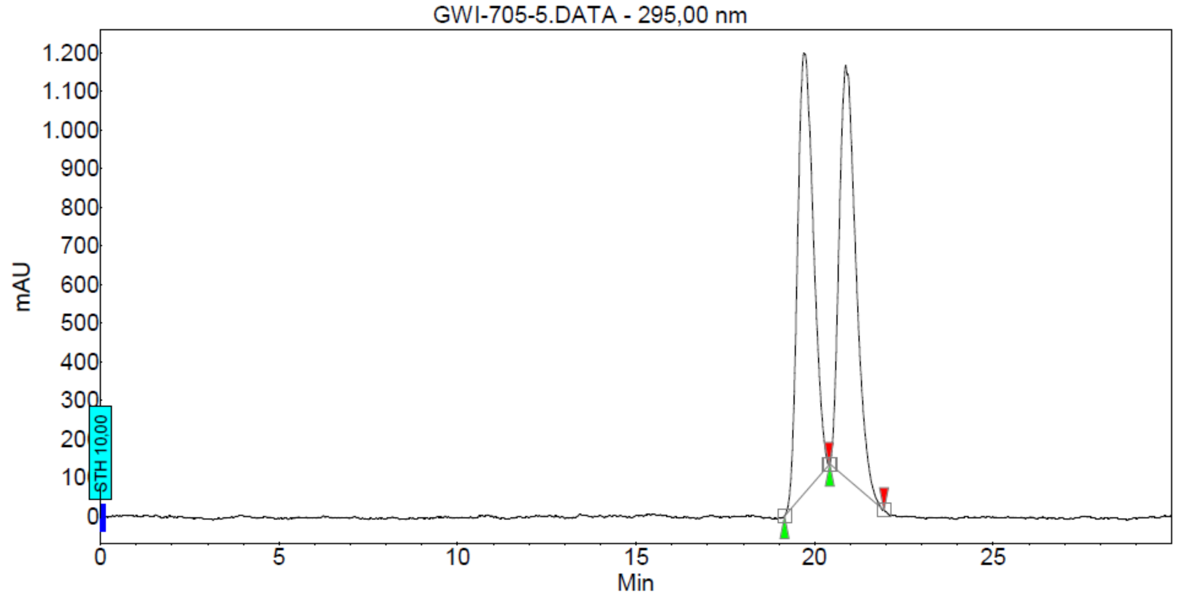

Peak results :

Index	Name	Time [Min]	Quantity [% Area]	Height [mAU]	Area [mAU.Min]	Area % [%]
1	UNKNOWN	19,69	50,36	1140,4	588,9	50,363
2	UNKNOWN	20,87	49,64	1065,6	580,4	49,637
Total			100,00	2206,0	1169,4	100,000

2.1.6.17 (*S,E*)-1-(2-((1-(4-Bromophenyl)ethyl)(methyl)amino)phenyl)-3-phenylprop-2-en-1-one ((*S*)-**1n**)

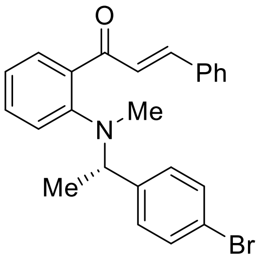

GP 5 starting from (*S*)-**S3a** (412 mg, 1.24 mmol, 1.00 equiv.) and benzaldehyde, room temperature, CH/EA 25:1, yellow oil (349 mg, 0.830 mmol, 67%).

^{1}H-NMR: (700 MHz, 298 K, CD_2Cl_2): δ = 7.63 (d, $^3J_{HH}$ =16.0 Hz, 1H, COCHC*H*), 7.58 – 7.55 (m, 2H, H_{Ar}), 7.55 – 7.52 (m, 1H, H_{Ar}), 7.43 – 7.38 (m, 4H, H_{Ar}), 7.32 (d, $^3J_{HH}$ =16.0 Hz, 1H, COC*H*CH), 7.29 – 7.26 (m, 2H, H_{Ar}), 7.12 – 7.08 (m, 1H, H_{Ar}), 7.03 – 7.00 (m, 3H, H_{Ar}), 4.38 (q, $^3J_{HH}$ =6.8 Hz, 1H, C*H*CH$_3$), 2.56 (s, 3H, NC*H*$_3$), 1.35 (d, $^3J_{HH}$ =6.9 Hz, 3H, CHC*H*$_3$); **^{13}C-NMR** (176 MHz, 298 K, $CDCl_3$): δ = 195.9 (C_q), 151.3 (C_q), 142.9 (CH, COCH*C*H), 140.4 (C_q), 135.4 (C_q), 135.1 (C_q), 131.6 (CH), ;131.3 (CH), 130.5 (CH), 130.1 (CH), 129.2 (CH), 129.1 (CH), 128.4 (CH), 127.2 (CH), 122.5 (CH), 121.2 (CH), 121.0 (C_q), 63.3 (CH, *C*HCH$_3$),

36.5 (CH_3, NCH_3), 18.0 (CH_3, *C*HCH_3); **^{15}N-NMR** (71 MHz, 298 K, $CDCl_3$): δ = 58.6; **HRMS** (ESI+, MeOH) *m/z* $[M+Na]^+$ ($C_{24}H_{22}NONaBr$) calc. 442.0782, found 442.0777.

HPLC (IA, 15 °C, heptane/i-PrOH: 98/2, flow rate: 0.7 ml/min, 295 nm).

Chromatogram : GWI-4832_channel5

System : LC920_2
Method : NOT DEFINED
User : AKP

Acquired : 22.10.2021 14:23:19
Processed : 26.10.2021 12:39:19
Printed : 26.10.2021 12:39:36

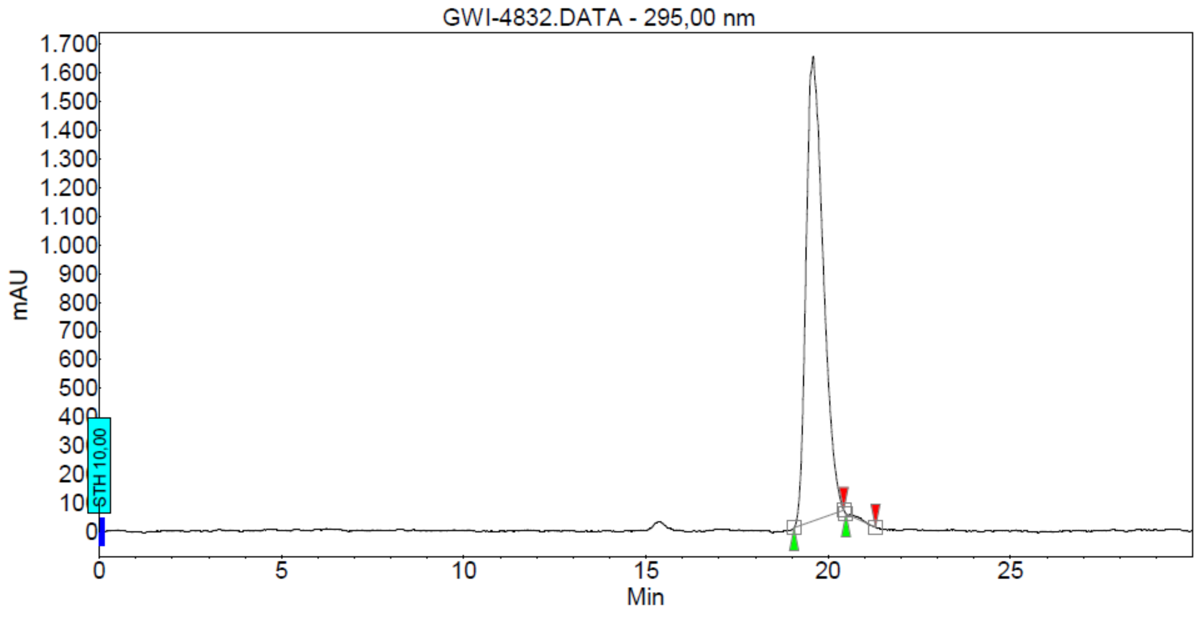

Peak results :

Index	Name	Time [Min]	Quantity [% Area]	Height [mAU]	Area [mAU.Min]	Area % [%]
1	UNKNOWN	19,59	99,76	1619,9	882,1	99,763
2	UNKNOWN	20,80	0,24	8,2	2,1	0,237
Total			100,00	1628,1	884,2	100,000

2.1.6.18 (*E*)-1-(2-(methyl(1-(4-(trifluoromethyl)phenyl)ethyl)amino)phenyl)-3-phenylprop-2-en-1-one (*rac*-**1o**)

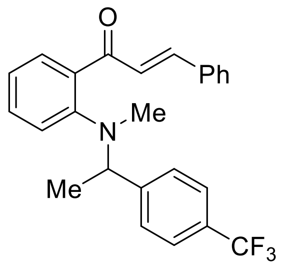

GP 5 starting from rac-**S3b** (160 mg, 0.500 mmol, 1.00 equiv.) and benzaldehyde, room temperature, CH/EA 40:1, yellow oil (95 mg, 0.232 mmol, 46%).

^{1}H-NMR: (700 MHz, 298 K, $CDCl_3$): δ = 7.62 (d, $^3J_{HH}$ =16.0 Hz, 1H, COCHC*H*), 7.58 – 7.55 (m, 2H, H_{Ar}), 7.55 – 7.52 (m, 1H, H_{Ar}), 7.43 – 7.39 (m, 6H, H_{Ar}), 7.31 (d, $^3J_{HH}$ =16.0 Hz, 1H, COC*H*CH), 7.29 – 7.26 (m, 2H, H_{Ar}), 7.14 – 7.11 (m, 1H, H_{Ar}), 7.05 – 7.03 (m, 1H, H_{Ar}), 4.46 (q, $^3J_{HH}$ =6.8 Hz, 1H,

$CHCH_3$), 2.57 (s, 3H, NCH_3), 1.38 (d, $^3J_{HH}$ =6.9 Hz, 3H, $CHCH_3$); **^{13}C-NMR** (176 MHz, 298 K, $CDCl_3$): δ = 196.0 (C_q), 151.1 (C_q), 145.7 (C_q), 143.1 (CH, COCH*C*H), 135.6 (C_q), 135.1 (C_q), 131.6 (CH), 130.5 (CH), 130.0 (CH), 129.1 (CH), 128.5 (CH), 127.8 (CH), 127.2 (CH, CO*C*HCH), 125.2 (q, $^3J_{CF}$ =3.7 Hz, CH), 122.8 (CH), 121.3 (CH), 63.5 (CH, *C*HCH_3), 36.9 (CH_3, NCH_3), 18.2 (CH_3, CHCH_3), two C_q signals were not observed; **^{15}N-NMR** (71 MHz, 298 K, $CDCl_3$): δ = 58.7; **HRMS** (ESI+, MeOH) *m/z* $[M+H]^+$ ($C_{25}H_{23}NOF_3$) calc. 410.1732, found 410.1731.

2.1.6.19 (*E*)-1-(2-((4-Fluorobenzyl)(methyl)amino)phenyl)-3-phenylprop-2-en-1-one (**1p**)

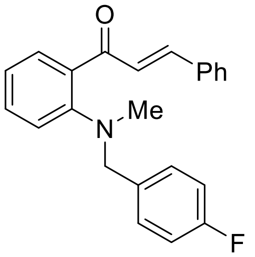

GP 5 starting from **S3e** (386 mg, 1.50 mmol, 1.00 equiv.) and benzaldehyde, 60 °C, CH/EA 25:1, yellow oil (431 mg, 1.25 mmol, 83%).

^{1}H-NMR (700 MHz, 298 K, $CDCl_3$): δ = 7.66 (d, $^3J_{HH}$ = 16.0 Hz, 1H, COCHC*H*), 7.57 – 7.54 (m, 3H, H_{Ar}), 7.43 – 7.40 (m, 4H, H_{Ar}), 7.37 (d, $^3J_{HH}$ = 15.9 Hz, COC*H*CH), 7.17 – 7.13 (m, 2H, H_{Ar}), 7.08 – 7.05 (m, 2H, H_{Ar}), 6.88 – 6.84 (m, 1H, H_{Ar}), 4.19 (s, 2H, CH_2), 2.67 (s, 3H, CH_3); **^{13}C-NMR** (176 MHz, 298 K, $CDCl_3$): δ = 195.5 (C_q), 162.2 (d, $^1J_{CF}$ = 245.4 Hz, C_q), 151.7 (C_q), 143.0 (CH, COCH*C*H), 135.2 (C_q), 133.4 (C_q), 133.3 (d, $^4J_{CF}$ = 3.0 Hz, C_q), 132.0 (CH), 130.4 (2CH), 130.0 (d, $^3J_{CF}$ = 8.0 Hz, CH), 129.1 (CH), 128.5 (CH), 126.7 (CH, CO*C*HCH), 121.6 (CH), 119.1 (CH), 115.3 (d, $^2J_{CF}$ = 21.4 Hz, CH), 60.2 (CH_2), 41.7 (CH_3); **^{19}F-NMR** (282 MHz, 298 K, $CDCl_3$): δ = −115.4 – 115.5 (m, 1F); **HRMS** (ESI+, MeCN) *m/z* $[M+Na]^+$ ($C_{23}H_{20}NOFNa$) calc. 368.1427, found 368.1417.

2.1.6.20 (*E*)-1-(2-((4-Fluorobenzyl)(methyl)amino)phenyl)-3-(4-fluorophenyl)prop-2-en-1-one (**1q**)

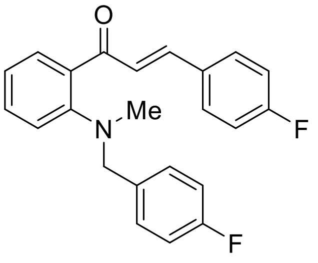

GP 5 starting from **S3e** (386 mg, 1.50 mmol, 1.00 equiv.) and 4-fluorobenzaldehyde, 60 °C, CH/EA 20:1, yellow oil (266 mg, 0.732 mmol, 49%).

^{1}H-NMR (700 MHz, 298 K, $CDCl_3$): δ = 7.62 (d, $^3J_{HH}$ = 15.9 Hz, 1H, COCHC*H*), 7.56 – 7.52 (m, 3H, H_{Ar}), 7.43 – 7.40 (m, 1H, H_{Ar}), 7.29 (d, $^3J_{HH}$ = 15.9 Hz, COC*H*CH), 7.18 – 7.12 (m, 2H, H_{Ar}), 7.12 – 7.08 (m, 2H, H_{Ar}), 7.08 – 7.04 (m, 2H, H_{Ar}), 6.89 – 6.85 (m, 2H, H_{Ar}), 4.19 (s, 2H, CH_2), 2.67 (s, 3H, CH_3); **^{13}C-NMR** (176 MHz, 298 K, $CDCl_3$): δ = 195.2 (C_q), 164.0 (d, $^1J_{CF}$ = 251.6 Hz, C_q), 162.2 (d, $^1J_{CF}$ = 245.5 Hz, C_q), 151.7 (C_q), 141.6 (CH,

COCHCH), 133.3 (C_q), 133.3 (d, $^4J_{CF}$ = 3.1 Hz, C_q), 132.0 (CH), 131.4 (d, $^4J_{CF}$ = 3.2 Hz, C_q), 130.4 (CH), 130.3 (d, $^3J_{CF}$ = 8.4 Hz, CH), 130.0 (d, $^3J_{CF}$ = 8.0 Hz, CH), 126.5 (d, $^5J_{CF}$ = 2.2 Hz, CH, CO*C*HCH), 121.6 (CH), 119.1 (CH), 116.2 (d, $^2J_{CF}$ = 21.9 Hz, CH), 115.3 (d, $^2J_{CF}$ = 21.9 Hz, CH), 60.3 (CH_2), 41.6 (CH_3); **^{19}F-NMR** (282 MHz, 298 K, $CDCl_3$): δ = −109.4 – 109.5 (m, 1F), −115.2 – 115.4 (m, 1F); **^{15}N-NMR** (71 MHz, 298 K, $CDCl_3$): δ = 53.0; **HRMS** (ESI+, MeOH) *m/z* $[M+Na]^+$ ($C_{23}H_{19}NOF_2Na$) calc. 386.1332, found 386.1318.

2.1.6.21 (*E*)-1-(2-(cinnamyl(methyl)amino)phenyl)-3-phenylprop-2-en-1-one(**1r**)

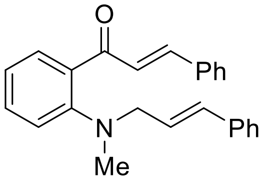

GP 5 starting from **S3c** (210 mg, 0.791 mmol, 1.00 equiv.) and benzaldehyde, room temperature, CH/EA 25:1, yellow oil (206 mg, 0.583 mmol, 74%).

^{1}H-NMR (700 MHz, 298 K, $CDCl_3$): δ = 7.72 (d, $^3J_{HH}$ = 15.9 Hz, 1H, COCHC*H*), 7.62 – 7.57 (m, 3H, H_{Ar}), 7.46 – 7.37 (m, 5H, 4H_{Ar}, COC*H*CH), 7.23 – 7.16 (m, 3H, H_{Ar}), 7.16 – 7.13 (m, 2H, H_{Ar}), 7.08 – 7.02 (m, 1H, H_{Ar}), 6.47 (d, $^3J_{HH}$ = 15.9 Hz, 1H NCH_2CHCH), 6.21 – 6.15 (m, 1H, NCH_2CHCH), 3.81 (bs, 2H, CH_2), 2.83 (bs, 3H, CH_3); **^{13}C-NMR** (176 MHz, 298 K, $CDCl_3$): δ = 195.1 (C_q), 142.6 (C_q), 136.7 (C_q), 135.4 (C_q), 133.1 (C_q), 132.2 (CH), 130.8 (CH), 130.4 (CH), 129.2 (CH), 128.7 (CH), 128.6 (CH), 127.8 (CH), 126.7 (CH), 126.5 (CH), 60.4 (CH_2), 40.8 (CH_3), not all carbon signals were observed; **^{15}N-NMR** (71 MHz, 298 K, $CDCl_3$): δ = 89.0;**HRMS** (ESI+, MeOH) *m/z* $[M+Na]^+$ ($C_{25}H_{23}NONa$) calc. 376.1677, found 376.1682.

2.1.6.22 (*E*)-1-(2-(methyl(3-phenylprop-2-yn-1-yl)amino)phenyl)-3-phenylprop-2-en-1-one (**1s**)

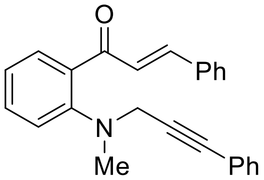

GP 5 starting from **S3d** (236 mg, 0.896 mmol, 1.00 equiv.) and benzaldehyde, room temperature, CH/EA 25:1, orange oil (258 mg, 0.734 mmol, 82%).

^{1}H-NMR (700 MHz, 298 K, $CDCl_3$): δ = 7.72 (d, $^3J_{HH}$ = 15.9 Hz, 1H, COCHC*H*), 7.60 – 7.58 (m, 3H, H_{Ar}), 7.49 – 7.4 (m, 1H, 4H_{Ar}), 7.46 (d, $^3J_{HH}$ = 15.9 Hz, 1H, COC*H*), 7.36 – 7.32 (m, 3H, H_{Ar}), 7.29 – 7.26 (m, 4H, H_{Ar}), 7.25 – 7.22 (m, 2h, H_{Ar}), 7.11 – 7.07 (m, 1H, H_{Ar}), 4.06 (s, 2H, CH_2), 2.96 (s, 3H, CH_3); **^{13}C-NMR** (176 MHz, 298 K, $CDCl_3$): δ = 195.2 (C_q), 150.6 (C_q), 142.8 (CH), 135.3 (C_q), 133.6 (C_q), 132.0 (CH), 131.8 (CH), 130.5 (CH), 130.0 (CH), 129.0 (CH), 128.5 (CH), 128.31 (CH), 128.27 (CH), 126.3 (CH), 123.0 (C_q), 122.2 (CH), 119.5 (CH), 85.7 (C_q), 84.6 (C_q), 47.3 (CH_2), 41.4 (CH_3);

^{15}N-NMR (71 MHz, 298 K, $CDCl_3$): δ = 47.8; **HRMS** (ESI+, MeOH) *m/z* $[M+Na]^+$ ($C_{25}H_{21}NONa$) calc. 374.1521, found 374.1503.

2.1.6.23 *E*)-1-(2-(dimethylamino)phenyl)-3-phenylprop-2-en-1-one (**4**)

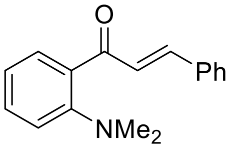

GP 5 starting from **S3f** (780 mg, 4.77 mmol, 1.00 equiv.) and benzaldehyde, room temperature, CH/EA 25:1, orange oil (924 mg, 3.68 mmol, 77%).

^{1}H-NMR (700 MHz, 298 K, $CDCl_3$): δ = 7.72 (d, $^3J_{HH}$ = 16.0 Hz, 1H, COC*H*), 7.60 -7.57 (m, 2H, H_{Ph}), 7.55 – 7.53 (m, 1H, H_{Ar}), 7.42 – 7.36 (m, 5H, 3H_{Ph}, H_{Ar}, COCHC*H*), 7.04 – 7.02 (m, 1H, H_{Ar}), 6.98 – 6.95 (m, 1H, H_{Ar}), 2.83 (s, 6H, 2CH_3); **^{13}C-NMR** (176 MHz, 298 K, $CDCl_3$): δ = 195.20 (C_q, CO), 152.40 (C_q), 142.60 (CH, CO*C*H), 135.41 (C_q), 132.05 (CH), 131.72 (C_q), 130.65 (CH), 130.26 (CH), 129.03 (CH), 128.43 (CH), 126.28 (CH), 120.21 (CH), 117.00 (CH), 44.51 (CH_3); **^{15}N-NMR** (71 MHz, 298 K, $CDCl_3$): δ = 43.8; **HRMS** (ESI+, MeOH) *m/z* $[M+H]^+$ ($C_{17}H_{18}NO$) calc. 252.1388, found 252.1367.

NMR spectroscopic and mass spectrometric data is in good agreement with previously reported chemical shifts and signal patterns.[SI13]

2.1.6.24 Synthesis of (E)-1-(4-fluoro-2-((4-methoxybenzyl)(methyl)amino)phenyl)-3-phenylprop-2-en-1-one (**2t**)

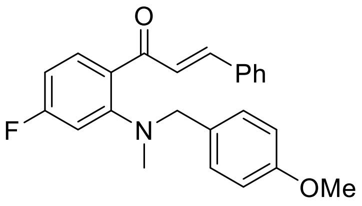

According to a modified literature procedure[SI10], benzaldehyde (1.10 equiv., 1.10 mmol, 117 mg), the ketone **S3l** (1.00 equiv., 1.00 mmol, 287 mg) and NaOH (2.00 equiv., 2.00 mmol, 80.0 mg) were dissolved in 3 ml MeOH and stirred at 60 °C overnight. Then water was added, and the resulting mixture was extracted thrice with EA. The combined organic layers were dried over Na_2SO_4, and the solvent was removed under reduced pressure. The crude product was purified by flash chromatography (SiO_2, CH/EA 20/1). The product was obtained as a yellow oil (219 mg, 0.584 mmol, 58%).

^{1}H-NMR (500 MHz, 303 K, $CDCl_3$): δ = 7.46 (d, $^3J_{HH}$ = 15.9 Hz, 1H, COCHC*H*), 7.60-7.54 (m, 3H, H_{Ar}), 7.42-7.40 (m, 3H, H_{Ar}), 7.38 (d, $^3J_{HH}$ = 16.0 Hz, 1H, COC*H*CH), 7.11-7.06 (m, 2H, H_{Ar}), 6.77-6.67 (m, 4H, H_{Ar}), 4.19 (s, 2H, NCH_2), 3.75 (s, 3H, OCH_3), 2.69 (s, 3H, NCH_3).

^{13}C-NMR (176 MHz, 298 K, $CDCl_3$): δ = 193.7 (C_q), 166.1-164.7 (d, $^1J_{CF}$ '= 250.8 Hz, C_q), 159.1 (C_q), 154.1 (d, $^3J_{CF}$ = 9.6 Hz, C_q), 143.1 (CH), 135.3 (C_q), 132.9 (d, $^3J_{CH}$ = 10.8 Hz, CH), 130.5 (CH), 129.7 (CH), 129.1 (CH), 129.1 (C_q), 128.4 (d, $^4J_{CF}$ = 2.3 Hz, C_q), 126.3 (CH), 114.0 (CH), 107.7 (d, $^2J_{CF}$ = 22 Hz, CH), 105.7 (d, $^2J_{CF}$ = 23.5 Hz, CH), 60.1 (CH_2, NCH_2), 55.4 (CH_2, OCH_3), 41.6 (CH_3, NCH_3).

HRMS (ESI+, MeCN) exact mass for $[MH]^+$ ($C_{24}H_{23}FNO_2$) calc m/z 376.1713, found 376.1708.

2.1.6.25 Synthesis of (E)-1-(5-fluoro-2-((4-methoxybenzyl)(methyl)amino)phenyl)-3-phenylprop-2-en-1-one (**1u**)

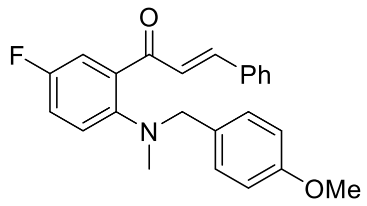

According to a modified literature procedure[SI10] benzaldehyde (1.10 equiv., 1.10 mmol, 117 mg), the ketone **S3m** (1.00 equiv., 1.00 mmol, 287 mg) and NaOH (2.00 equiv., 2.00 mmol, 80.0 mg) were dissolved in 3 ml MeOH and stirred at 60 °C overnight. Then water was added, and the resulting mixture was extracted thrice with EA. The combined organic layers were dried over Na_2SO_4, and the solvent was removed under reduced pressure. The crude product was purified by flash chromatography (SiO_2, CH/EA 20/1). The product was obtained as an orange oil (283 mg, 0.753 mmol, 75%).

^{1}H-NMR (500 MHz, 303 K, $CDCl_3$): δ = 7.66 (d, $^3J_{HH}$ = 16.0 Hz, 1H, COCHC*H*), 7.59-7.54 (m, 2H, H_{Ar}), 7.42 (d, $^3J_{HH}$ = 16.0 Hz, 1H, COC*H*CH), 7.42-7.38 (m, 3H, H_{Ar}), 7.28-7.25 (m, 1H, H_{Ar}), 7.15-7.04 (m, 4H, H_{Ar}), 6.73-6.69 (m, 2H, H_{Ar}), 4.08 (s, 2H, NCH_2), 3.74 (s, 3H, OCH_3), 2.62 (s, 3H, NCH_3).

^{13}C-NMR (176 MHz, 298 K, $CDCl_3$): δ = 194.1 (C_q), 159.0 (C_q), 158.8-157.4 (d, $^1J_{CF}$ '= 242.6 Hz, C_q), 148.5 (d, $^4J_{CF}$ = 2.2 Hz, C_q), 143.3 (CH), 135.6 (d, $^3J_{CF}$ = 6.1 Hz, C_q), 135.2 (C_q), 130.6 (CH), 130.0 (CH), 129.5 (C_q), 129.1 (CH), 128.7 (CH), 126.3 (CH), 121.3 (d, $^3J_{CF}$ = 7.5 Hz, CH), 118.5 (d, $^2J_{CF}$ = 22.2 Hz, CH), 116.6 (d, $^2J_{CF}$ = 23.4 Hz, CH), 113.9 (CH), 61.0 (CH_2, NCH_2), 55.4 (CH_3, OCH_3), 42.3 (CH_3, NCH_3).

HRMS (EI) exact mass for $[M]^+$ ($C_{24}H_{22}FNO_2$) calc m/z 375.1630, found 375.1628.

2.1.6.26 Synthesis of (E)-1-(2-fluoro-6-((4-methoxybenzyl)(methyl)amino)phenyl)-3-phenylprop-2-en-1-one (**1v**)

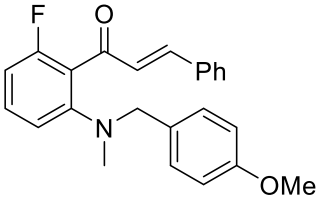

According to a modified literature procedure[SI10] benzaldehyde (1.10 equiv., 1.10 mmol, 117 mg), the ketone **S3k** (1.00 equiv., 1.00 mmol, 287 mg) and NaOH (2.00 equiv., 2.00 mmol, 80.0 mg) were dissolved in 3 ml MeOH and stirred at 60 °C overnight. Then water was added, and the resulting mixture was extracted thrice with DCM. The combined organic layers were dried over Na_2SO_4, and the solvent was removed under reduced pressure. The crude product was purified by flash chromatography (SiO_2, CH/EA 20/1). The product was obtained as an orange oil (276 mg, 0.734 mmol, 73%).

^{1}H-NMR (700 MHz, 298 K, $CDCl_3$): δ = 7.57-7.52 (m, 2H, H_{Ar}), 7.46 (d, $^3J_{HH}$ = 16.2 Hz; 1H, COCHC*H*), 7.43-7.38 (m, 2H, H_{Ar}), 7.32-7.28 (m, 1H, H_{Ar}), 7.11-7.09 (m, 2H, H_{Ar}), 7.09-7.04 (d, $^3J_{HH}$ = 16.1 Hz, 1H, (COC*H*CH), 6.88-6.83 (m, 1H, H_{Ar}),6.77-6.69 (m, 3H, H_{Ar}), 4.15 (s, 2H, NCH_2), 3.74 (s, 3H, OCH_3), 2.66 (s, 3H, NCH_3).

^{13}C-NMR (176 MHz, 298 K, $CDCl_3$): δ = 193.4 (C_q), 161.1-159.7 (d, $^1J_{CF}$ '= 248.3 Hz, C_q), 159.0 (C_q), 152.8 (d, $^3J_{CF}$ = 6.1 Hz, C_q), 144.9 (CH), 134.9 (C_q), 131.3 (d, $^3J_{CH}$ = 10.4 Hz, CH), 130.8 (CH), 129.8 (C_q), 129.6 (CH), 129.1 (CH), 128.7 (CH), 128.2 (CH), 121.7 (d, $^2J_{CF}$ = 17.2 Hz, C_q), 115.0 (d, $^4J_{CF}$ = 2.6 Hz, CH), 113.9 (CH), 118.7 (d, $^2J_{CF}$ = 22.2 Hz, CH), 60.0 (CH_2, NCH_2), 55.4 (CH_3, OCH_3), 41.6 (CH_3, NCH_3).

HRMS (ESI+, MeCN) exact mass for $[M+H]^+$ ($C_{24}H_{23}F_3NO_2$) calc m/z 376.1713, found 376.1707.

2.1.6.27 Synthesis of (E)-1-(2-((4-methoxybenzyl)(methyl)amino)-6-(trifluoromethyl)phenyl)-3-phenylprop-2-en-1-one (**1w**)

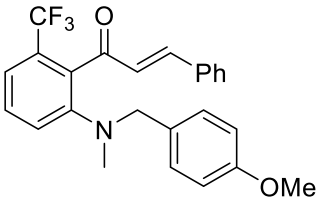

According to a modified literature procedure[SI10] benzaldehyde (1.10 equiv., 1.10 mmol, 117 mg.), the ketone **S3n** (1.00 equiv., 1.00 mmol, 337 mg) and NaOH (2.00 equiv., 2.00 mmol, 80.0 mg) were dissolved in 5 ml MeOH and stirred at 60 °C overnight. The resulting solid was filtered off and purified by recrystallization from methanol. The product was obtained as a yellow solid (115 mg, 0.270 mmol, 38%).

^{1}H-NMR (500 MHz, 303 K, $CDCl_3$): δ = 7.54-7.46 (m, 4H, H_{Ar}), 7.45-7.42 (m, 1H, H_{Ar}), 7.41.7.37 (m, 3H, H_{Ar}), 7.25 (d, $^3J_{HH}$ = 16.2 Hz, 1H, COCHC*H*), 7.12-7.07 (m, 2H, H_{Ar}), 7.00 (d, $^3J_{HH}$ = 16.2 Hz, 1H, COCC*H*CH), 6.69-6.64 (m, 2H, H_{Ar}), 3.99 (s, 2H, NCH_2), 3.72 (s, 3H, OCH_3), 2.56 (s, 3H, NCH_3).

^{13}C-NMR (176 MHz, 298 K, $CDCl_3$): δ = 195.9 (C_q), 159.0 (C_q), 152.5 (C_q), 144.2 (CH), 135.9 (q, $^4J_{CF}$ = 1.8 Hz, C_q), 134.8 (C_q), 130.7 (CH), 130.2 (CH), 130.1 (CH), 129.8 (C_q), 129.2-128.7 (q, $^2J_{CF}$ = 31.7 Hz, C_q), 129.1 (CH), 128.6 (CH), 128.4 (CH), 126.0-121.4 (q, $^1J_{CF}$ = 276.9 Hz, C_q), 125.8 (CH), 121.8 (q, $^3J_{CF}$ = 5.0 Hz, CH), 113.8 (CH), 60.9 (CH_2, NCH_2), 55.4 (CH_3, OCH_3), 42.6 (CH_3, NCH_3).

HRMS (ESI+, MeCN) exact mass for $[M+Na]^+$ ($C_{24}H_{22}FNO_2$) calc m/z 448.1500, found 448.1498.

2.1.6.28 Synthesis of (E)-1-(2-((4-methoxybenzyl)(methyl)amino)-5-(trifluoromethyl)phenyl)-3-phenylprop-2-en-1-one (**1x**)

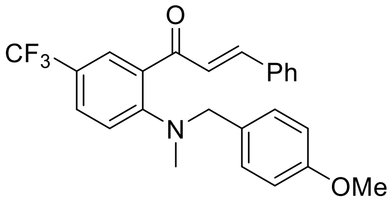

According to a modified literature procedure[SI10] benzaldehyde (1.10 equiv., 1.10 mmol, 117 mg), the ketone **S3o** (1.00 equiv., 1.00 mmol, 337 mg) and NaOH (2.00 equiv., 2.00 mmol, 80.0 mg) were dissolved in 7 ml MeOH and stirred at 60 °C overnight. Then water was added, and the resulting mixture was extracted thrice with EA. The combined organic layers were dried over Na_2SO_4, and the solvent was removed under reduced pressure. The crude product was purified by flash chromatography (SiO_2, CH/EA 20/1). Further purification was achieved by recrystallization from methanol and the product was obtained as a yellow solid (191 mg, 0.449 mmol, 45%).

^{1}H-NMR (500 MHz, 303 K, $CDCl_3$): δ = 7.80-7.77 (m, 1H, H_{Ar}), 7.70 (d, $^3J_{HH}$ = 15.9 Hz, 1H, COCCHC*H*), 7.61-7.54 (m, 3H, H_{Ar}), 7.45-7.39 (m. 3H, H_{Ar}), 7.28 (d, $^3J_{HH}$ = 16.0 Hz, 1H, COC*H*CH), 7.11-7.05 (m, 3H, H_{Ar}), 6.79-6.75 (m, 2H, H_{Ar}), 4.32 (s, 2H, NCH_2), 3.76 (s, 3H, OCH_3), 2.77 (s, 3H, NCH_3).

^{13}C-NMR (176 MHz, 298 K, $CDCl_3$): δ = 194.0 (C_q), 159.2 (C_q), 153.8 (C_q), 144.4 (CH), 134.9 (C_q), 130.8 (CH), 130.5 (C_q), 129.4 (CH), 129.2 (CH), 128.9 (C_q), 128.7 (CH), 128.2 (q, $^3J_{CF}$ = 3.7 Hz, CH), 126.8-122.2 (q, $^1J_{CF}$ = 271.2 Hz, C_q), 125.7 (CH), 121.8-121.2 (q, $^2J_{CF}$ = 33.2 Hz, C_q), 117.9 (CH), 114.2 (CH), 59.3 (CH_2, NCH_2), 55.4 (CH_3, OCH_3), 41.6 (CH_3, NCH_3).

HRMS (EI) exact mass for $[M]^+$ ($C_{25}H_{22}F_3NO_2$) calc m/z 425.1598, found 425.1598.

2.1.6.29 Synthesis of (E)-1-(5-bromo-2-((4-methoxybenzyl)(methyl)amino)phenyl)-3-phenylprop-2-en-1-one (**1y**)

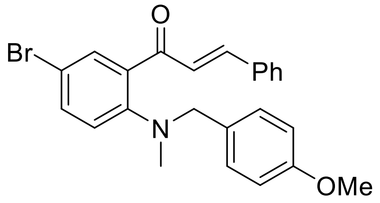

According to a modified literature procedure[SI2] benzaldehyde (1.10 equiv., 1.10 mmol, 117 mg), the ketone **S3q** (1.00 equiv., 1.00 mmol, 348 mg) and NaOH (2.00 equiv., 2.00 mmol, 80.0 mg) were dissolved in 7 ml MeOH and stirred at 60 °C overnight. Then water was added, and the resulting mixture was extracted thrice with EA. The combined organic layers were dried over Na_2SO_4, and the solvent was removed under reduced pressure. The crude product was purified by flash chromatography (SiO_2, CH/EA 20/1).). The product was obtained as a yellow solid (249 mg, 0.571 mmol, 57%).

¹H-NMR (500 MHz, 303 K, $CDCl_3$): δ = 7.68 (d, $^3J_{HH}$ = 15.9 Hz, 1H, COCHC*H*), 7.65-7.64 (m, 1H, H_{Ar}), 7.58-7.64 (m, 2H, H_{Ar}), 7.49-7.46 (m, 1H, H_{Ar}), 7.42-7.40 (m, 3H, H_{Ar}), 7.34 (d, $^3J_{HH}$ = 15.9 Hz, 1H, COC*H*CH), 7.09-7.05 (m, 2H, H_{Ar}),6.94-6.91 (m, 1H, H_{Ar}), 6.75-6.71 (m, 2H, H_{Ar}), 4.15 (s, 2H, NCH_2), 3.75 (s, 3H, OCH_3), 2.66 (s, 3H, NCH_3).

¹³C-NMR (176 MHz, 298 K, $CDCl_3$): δ = 193.8 (C_q), 159.2 (C_q), 159.1 (C_q), 150.9 (C_q), 143.7 (CH), 135.1(C_q), 134.6 (CH), 134.4 (C_q), 133.0 (CH), 130.6 (CH), 129.7 (CH), 129.2 (C_q), 129.1 (CH), 128.7 (CH), 126.4 (CH), 120.8 (CH), 113.9 (CH), 113.6 (C_q), 60.2 (CH_2, N*C*H_2), 55.4 (CH_3, O*C*H_3), 41.8 (CH_3, N*C*H_3).

HRMS (ESI+, MeCN) exact mass for $[M+Na]^+$ ($C_{24}H_{22}NO_2NaBr$) calc m/z 458.0732, found 458.0725

2.1.6.30 Synthesis of (E)-1-(2-((4-methoxybenzyl)(methyl)amino)-4-methylphenyl)-3-phenylprop-2-en-1-one (**1z**)

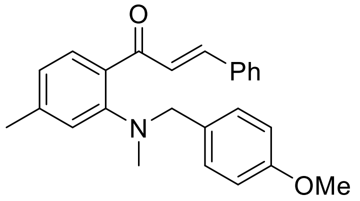

According to a modified literature procedure[SI2] benzaldehyde (1.10 equiv., 1.10 mmol, 117 mg), the ketone **S3r** (1.00 equiv., 1.00 mmol, 283 mg) and NaOH (2.00 equiv., 2.00 mmol, 80.0 mg) were dissolved in 7 ml MeOH and stirred at 60 °C overnight. Then water was added, and the resulting mixture was extracted thrice with EA. The combined organic layers were dried over Na_2SO_4, and the solvent was removed under reduced pressure. The crude product was purified

by flash chromatography (SiO_2, CH/EA 20/1). The product was obtained as a yellow oil (323 mg, 0.870 mmol, 87%).

^{1}H-NMR (500 MHz, 303 K, $CDCl_3$): δ = 7.69 (d, $^3J_{HH}$ = 15.9 Hz, 1H, COCCHC*H*), 7.58-7.54 (m, 2H, H_{Ar}), 7.52-7.49 (m, 1H, H_{Ar}), 7.46 (d, $^3J_{HH}$ = 15.9 Hz, 1H, COC*H*CH), 7.41-7.37 (m. 3H, H_{Ar}), 7.13-7.09 (m, 2H, H_{Ar}), 6.90-6.84 (m, 2H, H_{Ar}), 6.74-6.71 (m, 2H, H_{Ar}), 4.17 (s, 2H, NCH_2), 3.75 (s, 3H, OCH_3), 2.65 (s, 3H, NCH_3), 2.38 (s, 3H, CCH_3).

^{13}C-NMR (176 MHz, 298 K, $CDCl_3$): δ = 194.9 (C_q), 158.9 (C_q), 152.4 (C_q), 142.7 (C_q), 142.3 (CH), 135.6 (C_q), 130.9 (CH), 130.5 (C_q), 130.2 (CH), 129.8 (CH), 129.0 (CH), 128.5 (CH), 126.9 (CH), 122.2 (CH), 119.6 (CH), 113.9 (CH), 60.3 (CH_2, N*C*H_2), 55.4 (CH_3, O*C*H_3), 41.8 (CH_3, N*C*H_3), 22.0 (CH_3, C*C*H_3).

HRMS (ESI+, MeCN) exact mass for $[M+H]^+$ ($C_{25}H_{26}NO_2$) calc m/z 372.1964, found 372.1960.

2.1.6.31 Synthesis of (E)-1-(2-((4-methoxybenzyl)(methyl)amino)-5-nitrophenyl)-3-phenylprop-2-en-1-one (**1aa**)

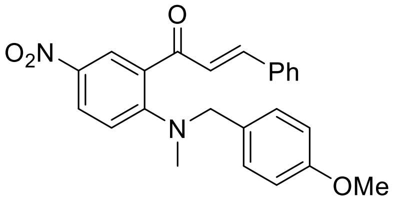

According to a modified literature procedure[SI2] benzaldehyde (1.10 equiv., 1.10 mmol, 117 mg), the ketone **S3s** (1.00 equiv., 1.00 mmol, 314 mg) and NaOH (2.00 equiv., 2.00 mmol, 80.0 mg) were dissolved in 7 ml MeOH and stirred at 60 °C overnight. The resulting solid was filtered off, washed with methanol, dried under reduced pressure, and used without further purification. The product was obtained as a yellow solid (347 mg, 0.863 mmol, 86%).

^{1}H-NMR (700 MHz, 298 K, $CDCl_3$): δ = 8.43-8.41 (m, 1H, H_{Ar}), 8.20-8.16 (m, 1H, H_{Ar}), 7.68 (d, $^3J_{HH}$ = 15.9 Hz, 1H, COCCHC*H*), 7.60-7.56 (m, 2H, H_{Ar}), 7.46-7.30 (m. 3H, H_{Ar}), 7.17 (d, $^3J_{HH}$ = 16.0 Hz, 1H, COC*H*CH), 7.09-7.06 (m, 2H, H_{Ar}), 6.96-6.94 (m, 1H, H_{Ar}), 6.84-6.81 (m, 2H, H_{Ar}), 4.49 (s, 2H, NCH_2), 3.77 (s, 3H, OCH_3), 2.90 (s, 3H, NCH_3).

^{13}C-NMR (176 MHz, 298 K, $CDCl_3$): δ = 192.8 (C_q), 159.4 (C_q), 155.0 (C_q), 146.0 (CH), 138.2 (C_q), 134.5(C_q), 131.2 (CH), 129.3 (CH), 128.8 (CH), 127.9 (C_q), 127.5 (CH), 127.3 (CH), 127.1 (C_q), 125.3 (CH), 116.2 (CH), 114.5 (CH), 58.4 (CH_2, N*C*H_2), 55.5 (CH_3, O*C*H_3), 42.0 (CH_3, N*C*H_3).

HRMS (ESI+, MeCN) exact mass for $[M+Na]^+$ ($C_{24}H_{22}N_2O_4Na$) calc m/z 425.1477, found 425.1481.

2.1.6.32 Synthesis of (E)-1-(4-methoxy-2-((4-methoxybenzyl)(methyl)amino)phenyl)-3-phenylprop-2-en-1-one (**1ab**)

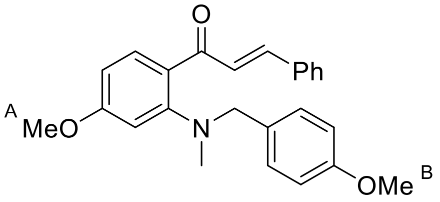

According to a modified literature procedure[SI2] benzaldehyde (1.10 equiv., 1.10 mmol, 117 mg), the ketone **S3t** (1.00 equiv., 1.00 mmol, 299 mg) and NaOH (2.00 equiv., 2.00 mmol, 80.0 mg) were dissolved in 7 ml MeOH and stirred at 60 °C overnight. Then water was added, and the resulting mixture was extracted thrice with EA. The combined organic layers were dried over Na_2SO_4, and the solvent was removed under reduced pressure. The crude product was purified by flash chromatography (SiO_2, CH/EA 10/1). Further purification was achieved by recrystallization from methanol and the product was obtained as a yellow solid (212 mg, 0.547 mmol, 55%).

^{1}H-NMR (700 MHz, 298 K, $CDCl_3$): δ = 7.71 (d, $^3J_{HH}$ = 15.9 Hz, 1H, COCCHC*H*), 7.65-7.62 (m, 1H, H_{Ar}), 7.58-7.54 (m, 2H, H_{Ar}), 7.52 (d, $^3J_{HH}$ = 15.9 Hz, 1H, COC*H*CH), 7.46-7.30 (m. 3H, H_{Ar}), 7.17 (d, $^3J_{HH}$ = 16.0 Hz, 1H, COC*H*CH), 7.42-7.37 (m, 3H, H_{Ar}), 7.13-7.09 (m, 2H, H_{Ar}), 6.76-6.72 (m, 2H, H_{Ar}), 6.59-6.56 (m, 1H, H_{Ar}), 6.55-6.53 (m, 1H, H_{Ar}), 4.18 (s, 2H, NCH_2), 3.84 (s, 3H, OCH_3^B), 3.75 (s, 3H, OCH_3^A), 2.67 (s, 3H, NCH_3).

^{13}C-NMR (176 MHz, 298 K, $CDCl_3$): δ = 193.3 (C_q), 163.2 (C_q), 159.0 (C_q), 154.3 (C_q), 142.0 (CH), 135.6 (C_q), 133.0 (CH), 130.1 (CH), 129.8 (CH), 129.6 (C_q), 129.0 (CH), 128.5 (CH), 126.8 (CH), 125.9 (C_q), 113.9 (CH), 106.0 (CH), 104.9 (CH), 60.3 (CH_2, NCH_2), 55.6 (CH_3, OCH_3^A), 55.4 (CH_3, OCH_3^B), 41.9 (CH_3, NCH_3).

HRMS (EI) exact mass for $[M]^+$ ($C_{25}H_{25}NO_3$) calc m/z 387.1834, found 387.1834.

2.2 Cyclization experiments

2.2.1 General Procedure for NMR-scale experiments (GP 6)

In a glove box $B(C_6F_5)_3$ (2.6 mg, 5.00 µmol, 0.100 equiv.) and the starting material (50.0 µmol, 1.00 equiv.) were dissolved in $CDCl_3$ (0.1 M) and transferred to a J. YOUNG NMR tube. The reaction mixture was kept at room temperature or heated and analyzed by NMR spectroscopy.

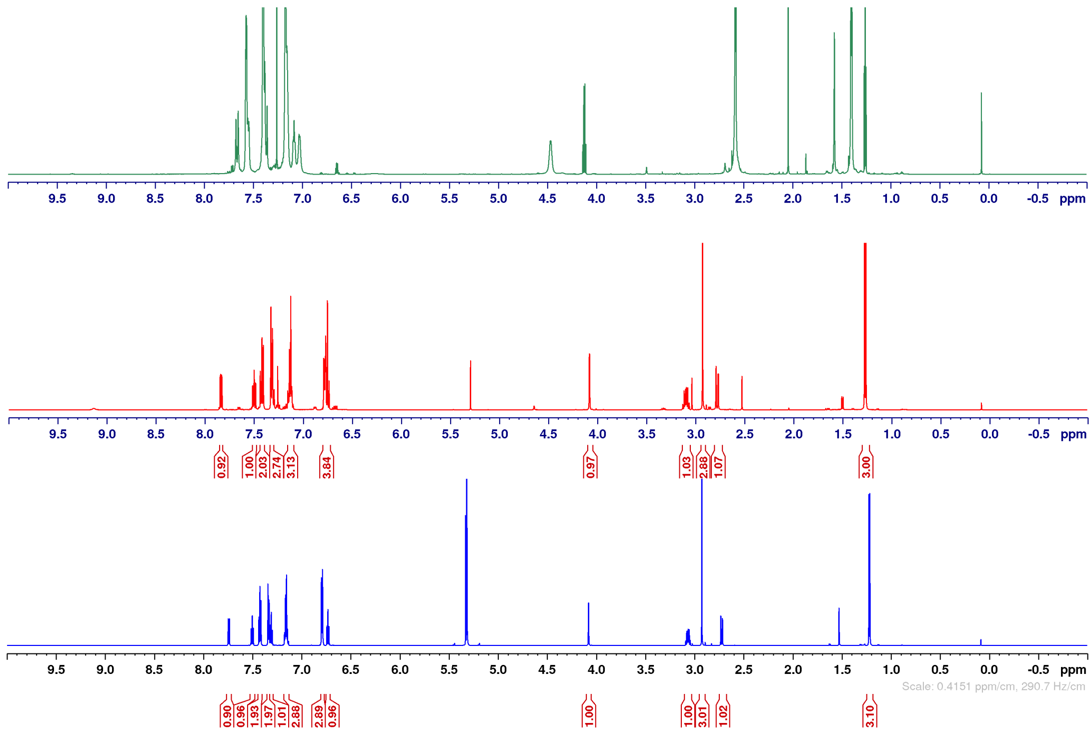

Figure S1: ^{1}H-NMR spectra of the reaction of rac-***1a*** *(green), after mixing with $B(C_6F_5)_3$ (red) and* ra

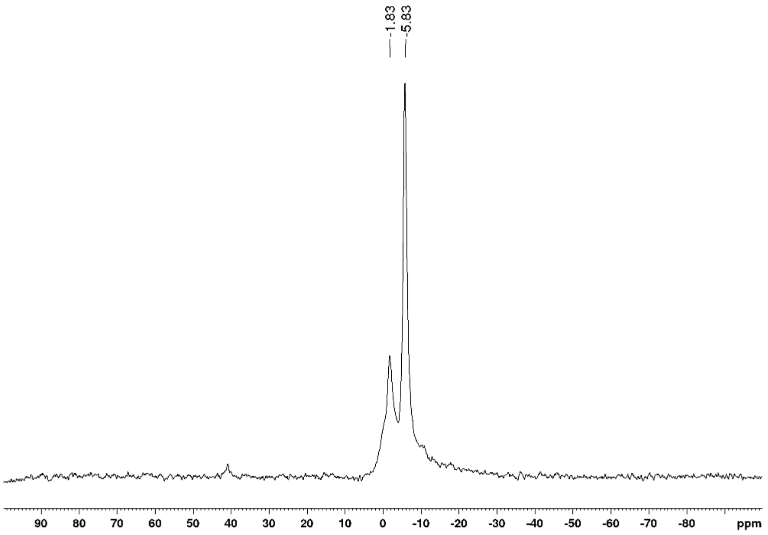

Figure S2: ^{11}B-NMR spectrum of the reaction mixture of rac-***2a*** *with $B(C_6F_5)_3$.*

2.2.2 Lewis acid screening

Substrate **1b** (17.9 mg, 50.0 µmol, 1.00 equiv.) and a Lewis acid (5.00 µmol, 0.100 equiv.) were dissolved in $CDCl_3$ (0.1 M) and transferred to a J. YOUNG NMR tube. The reaction mixture was kept at room temperature or heated to 60 °C for the indicated time and analyzed by NMR spectroscopy. Yields were determined by integration of starting material and product signals. The integral of the product signal was divided by the sum of both integrals.

Table S1: Lewis acid screening.

Entry	Lewis Acid	conversion [%] (determined by NMR spectroscopy)
1	MgI_2 and PyBox	n.r.
2	$Mg(OTf)_2$ and PyBox	n.r.
3	$Yb(OTF)_3$ and PyBox	>99 conversion but not the product (60 C, 54 h)
4	Proline	n.r.
5	TiCl4·2THF	>99 (30 min)
6	*p*TSA in Tol-d_8	25 (18 h)
7	*p*TSA in $CDCl_3$	n.r. at room temperature, 45 (60°C, 18 h)
7	$ZnCl_2$	n.r.
8	$FeCl_2$ and PyBox in THF	n.r.
9	$FeBr_2$ and PyBox in THF	n.r.
10	$BF_3 \cdot OEt_2$	>99 (30 min) with side reactions
11	B(2,3,5,6-F_4-C_6H)$_3$	>99 (80 min)
12	B(2,3,6-F_3-C_6H_2)$_3$	>99 (2 h)
13	B(2,4,6-F_3-C_6H_2)$_3$	34 (80 min), 73 (4h)
14	B(2,6-F_2-C_6H_3)$_3$	30 (18 h)
15	$B(C_6F_5)_3$ (10%)	>99 (30 min)
16	$B(C_6F_5)_3$ (5%)	>99 (120 min)
17	$B(C_6F_5)_3$ (2%)	>99 (360 min)

2.2.3 General procedure for table run experiments (GP 7)

The substrate (250 µmol, 1.00 equiv.) and $B(C_6F_5)_3$ (2.6 mg, 25.0 µmol, 0.100 equiv.) were dissolved in $CHCl_3$ (0.1 M) and placed in a fresh crimp seal glass vial. The reaction mixture was stirred at room temperature or 60 °C for the indicated time. The resulting mixture was diluted with 10 ml DCM, washed with 10 ml H_2O, and extracted twice with 10 ml DCM. The combined organic layers were dried over Na_2SO_4, and volatiles were removed under reduced pressure. Subsequent flash chromatography (mixtures of CH/EA, SiO_2) yielded the desired compound.

2.2.3.1 *rac*-*trans*-2,3-1-methyl-2-phenyl-3-(*cis*-1-phenylethyl)-2,3-dihydroquinolin-4(1H)-one (*rac*-**2a**)

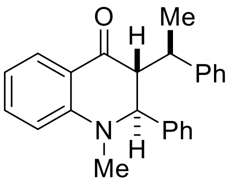

GP 7 starting from *rac*-**1a**, room temperature, 30 minutes, CH/EA 40:1, light yellow solid (76 mg, 0.234 mmol, 89%)

^{1}H-NMR (700 MHz, 298 K, CD_2Cl_2): δ = 7.76 – 7.73 (m, 1H, H_{Ar}), 7.52 – 7.49 (m, 1H, H_{Ar}), 7.45 – 7.41 (m, 2H, H_{Ar}), 7.35 – 7.33 (m, 2H, H_{Ar}), 7.33 – 7.30 (m, 2H, H_{Ar}), 7.18 – 7.13 (m, 3H, H_{Ar}), 6.80 – 6.78 (m, 3H, H_{Ar}), 6.75 – 6.72 (m, 1H, H_{Ar}), 4.08 (d, $^3J_{HH}$ = 1.4 Hz, 1H, NC*H*), 3.07 (dq, $^3J_{HH}$ = 11.3, 7.0 Hz, 1H, COCHC*H*(CH_3)), 2.93 (s, 3H, NC*H*$_3$), 2.73 (dd, $^3J_{HH}$ = 11.2, 1.8 Hz, 1H, COCH), 1.22 (d, $^3J_{HH}$ = 7.1 Hz, 3H, COCHCH(C*H*$_3$)); **^{13}C-NMR** (176 MHz, 298 K, CD_2Cl_2): δ = 195.0 (C_q), 150.3 (C_q), 145.1 (C_q), 139.4 (C_q), 136.3 (CH), 129.3 (CH), 128.2 (CH), 128.0 (CH9, 127.6 (CH), 127.3 (CH), 126.3 (CH), 118.8 (C_q), 116.4 (CH), 112.1 (CH), 67.0 (CH, N*C*H), 61.3 (CH, CO*C*H), 40.7 (CH, COCH*C*H(CH_3)), 38.5 (CH_3, N*C*H$_3$), 20.2 (CH_3, COCHCH(*C*H$_3$)); **^{15}N-NMR** (71 MHz, 298 K, CD_2Cl_2): δ = 60.7; **HRMS** (ESI+, MeOH) *m/z* $[M+Na]^+$ ($C_{24}H_{23}NONa$) calc. 364.1677, found 364.1677.

HPLC (IA, 15 °C, heptane/i-PrOH: 95/5, flow rate: 0.7 ml/min, 238 nm).

Chromatogram : GWI-427-2_channel5

System : LC920_2
Method : NOT DEFINED
User : AKP

Acquired : 25.10.2021 14:14:43
Processed : 25.10.2021 15:05:34
Printed : 26.10.2021 12:37:43

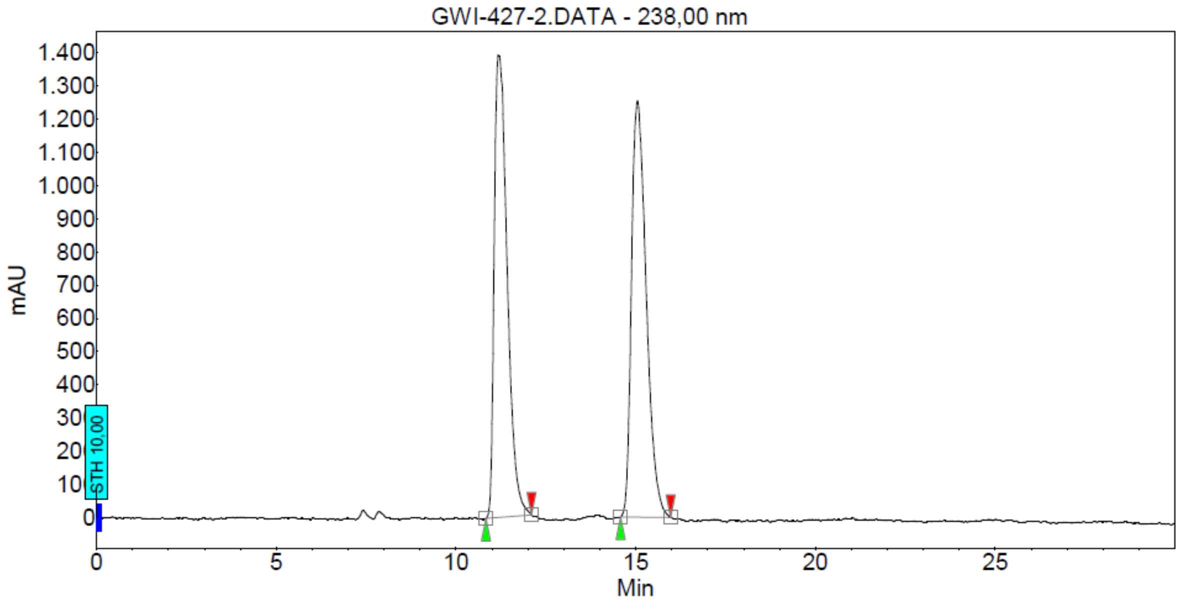

Peak results :

Index	Name	Time [Min]	Quantity [% Area]	Height [mAU]	Area [mAU.Min]	Area % [%]
1	UNKNOWN	11,17	49,41	1391,5	581,7	49,405
2	UNKNOWN	15,04	50,59	1251,5	595,7	50,595
Total			100,00	2643,0	1177,3	100,000

2.2.3.2 *rac-trans*-2,3-1-methyl-2-phenyl-3-(*cis*-1-phenylethyl-1-d)-2,3-dihydroquinolin-4(1H)-one (d_1-*rac*-**2a**)

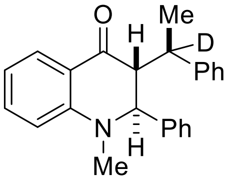

GP 7 starting from d_1-*rac*-**1a**, room temperature, 30 minutes, CH/EA 40:1, light yellow solid (77 mg, 0.225 mmol, 90%)

^{1}H-NMR (700 MHz, 298 K, CD_2Cl_2): δ = 7.78 – 7.74 (m, 1H, H_{Ar}), 7.54 – 7.49 (m, 1H, H_{Ar}), 7.46 – 7.41 (m, 2H, H_{Ar}), 7.36 – 7.29 (m, 3H, H_{Ar}), 7.20 – 7.14 (m, 3H, H_{Ar}), 6.82 – 6.78 (m, 3H, H_{Ar}), 6.76 – 6.72 (m, 1H, H_{Ar}), 4.09 (d, $^{3}J_{HH}$ = 1.7 Hz, 1H, NC*H*), 2.93 (s, 3H, NCH_3), 2.73 (d, $^{3}J_{HH}$ = 1.0 Hz, 1H, COCH), 1.22 (s, 3H, COCHCD(CH_3)); **^{13}C-NMR** (176 MHz, 298 K, CD_2Cl_2): δ = 197.1 (C_q), 152.3 (C_q), 147.0 (C_q), 141.4 (C_q), 138.3 (CH), 131.3 (CH), 131.1 (CH), 130.2 (CH), 130.0 (CH), 129.6 (CH), 129.3 (CH), 128.3 (CH), 120.8 (C_q), 118.4 (CH), 114.1 (CH), 70.0 (CH, N*C*H) 63.2 (CH, CO*C*H), 42.3 (t, $^{1}J_{CD}$ = 19.8 Hz, C_q, COCH*C*DCH$_3$), 40.5 (CH$_3$, N*C*H$_3$),

22.1 (CH_3, COCHCD*C*H_3); **^{15}N-NMR** (71 MHz, 298 K, CD2Cl2): δ = 63.6; **HRMS** (ESI+, MeOH) *m/z* $[M+Na]^+$ ($C_{24}H_{22}DNONa$) calc. 365.1740, found 365.1759.

2.2.3.3 (2*S*,3*R*)-1-methyl-2-phenyl-3-((*R*)-1-phenylethyl)-2,3-dihydroquinolin-4(1H)-one ((*2S,3R*)-**2a**)

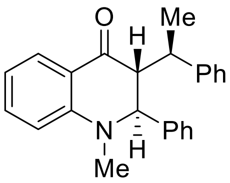

GP 7 starting from(*S*)-**1a**, room temperature, 30 minutes, CH/EA 40:1, light yellow solid (71 mg, 0.208 mmol, 83%). Suitable crystals for X-ray single crystal structure analysis were grown by condensation of *n*-pentane into the dichloromethane solution.

^{1}H-NMR (700 MHz, 298 K, CD_2Cl_2): δ = 7.77 – 7.73 (m, 1H, H_{Ar}), 7.53 – 7.49 (m, 1H, H_{Ar}), 7.45 – 7.41 (m, 2H, H_{Ar}), 7.36 – 7.33 (m, 2H, H_{Ar}), 7.33 – 7.30 (m, 1H, H_{Ar}), 7.19 – 7.14 (m, 3H, H_{Ar}), 6.81 – 6.78 (m, 3H, H_{Ar}), 6.75 – 6.72 (m, 1H, H_{Ar}), 4.08 (d, $^3J_{HH}$ = 1.6 Hz, 1H, NC*H*), 3.07 (dq, $^3J_{HH}$ = 11.2, 7.0 Hz, 1H, COCHC*H*(CH_3)), 2.93 (s, 3H, NC*H*$_3$), 2.73 (dd, $^3J_{HH}$ = 11.1, 1.8 Hz, 1H, COCH), 1.22 (d, $^3J_{HH}$ = 7.1 Hz, 3H, COCHCH(C*H*$_3$)); **^{13}C-NMR** (176 MHz, 298 K, CD_2Cl_2): δ = 195.0 (C_q), 150.3 (C_q), 145.1 (C_q), 139.4 (C_q), 136.3 (CH), 129.3 (CH), 129.1 (CH), 128.2 (CH), 128.0 (CH), 127.6 (CH), 127.3 (CH), 126.3 (CH), 118.8 (C_q), 116.4 (CH), 112.1 (CH), 67.0 (CH, N*C*H), 61.3 (CH, CO*C*H), 40.7 (CH, COCH*C*H(CH_3)), 38.5 (CH_3, N*C*H_3), 20.2 (CH_3, NCHCH(C*H*$_3$)); **^{15}N-NMR** (71 MHz, 298 K, CD_2Cl_2): δ = 61.4; **HRMS** (ESI+, MeOH) *m/z* $[M+Na]^+$ ($C_{24}H_{23}NONa$) calc. 364.1677, found 364.1681.

HPLC (IA, 15 °C, heptane/i-PrOH: 95/5, flow rate: 0.7 ml/min, 238 nm).

Chromatogram : GWI-449-3_channel5

System : LC920_2
Method : NOT DEFINED
User : AKP

Acquired : 25.10.2021 14:47:29
Processed : 26.10.2021 12:37:10
Printed : 26.10.2021 12:37:29

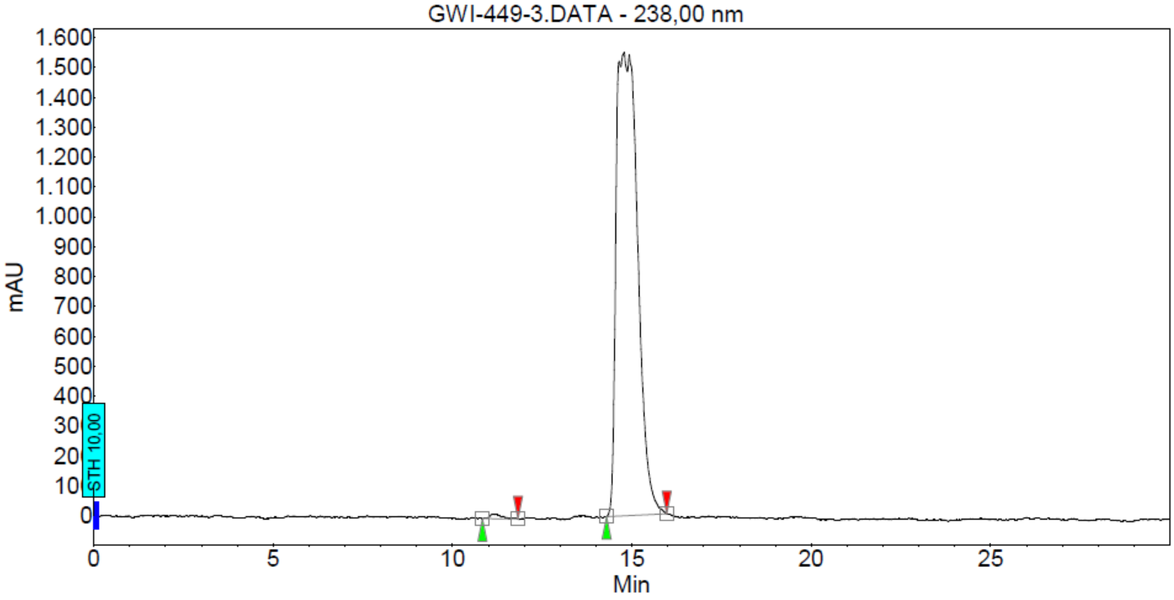

Peak results :

Index	Name	Time [Min]	Quantity [% Area]	Height [mAU]	Area [mAU.Min]	Area % [%]
2	UNKNOWN	11,11	0,59	15,4	6,3	0,586
1	UNKNOWN	14,79	99,41	1551,1	1073,4	99,414
Total			100,00	1566,4	1079,7	100,000

2.2.3.4 *rac-trans*-2,3-3-(4-methoxybenzyl)-1-methyl-2-phenyl-2,3-dihydroquinolin-4(1H)-one (**2b**)

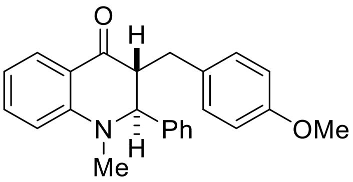

GP 7 starting from **1b**, room temperature, 30 minutes, CH/EA 10:1, yellow solid (85 mg, 0.238 mmol, 95%).

^{1}H-NMR (700 MHz, 298 K, $CDCl_3$): δ = 7.87 – 7.84 (m, 1H, H_{Ar}), 7.52 – 7.48 (m, 1H, H_{Ar}), 7.22 – 7.18 (m, 2H, H_{Ar}), 7.17 – 7.13 (m, 3H, H_{Ar}), 6.93 – 6.90 (m, 2H, H_{Ar}), 6.84 – 6.81 (m, 2H, H_{Ar}), 6.79 – 6.77 (m, 1H, H_{Ar}), 6.77 – 6.74 (m, 1H, H_{Ar}), 4.26 (d, $^3J_{HH}$ = 1.8 Hz, 1H, NC*H*), 3.83 (s, 3H, OCH_3), 3.04 (s, 3H, NCH_3), 3.00 (dd, $^2J_{HH}$ = 13.4 Hz, $^3J_{HH}$ = 4.8 Hz, 1H, COCHCH_2^A), 2.92 (ddd, $^3J_{HH}$ = 11.3, 4.9, 1.9 Hz, 1H, COC*H*), 2.81 (dd, $^2J_{HH}$ = 13.4 Hz, $^3J_{HH}$ = 11.3 Hz, 1H, COCHCH_2^B); **^{13}C-NMR** (176 MHz, 298 K, $CDCl_3$): δ = 195.1 (C_q), 158.6 (C_q), 150.3 (C_q), 139.2 (C_q), 136.3 (CH), 130.6 (C_q), 130.3 (CH), 128.9 (CH), 128.5 (CH), 127.5 (CH), 126.3 (CH),

118.4 (C_q), 116.6 (CH), 114.3 (CH), 112.1 (CH), 66.1 (CH, N*C*H), 56.3 (CH, CO*C*H) 55.4 (CH_3, O*C*H_3), 38.5 (CH_3, N*C*H_3), 36.4 (CH_2); **HRMS** (ESI+, MeOH) *m/z* $[M+Na]^+$ ($C_{24}H_{23}NO_2Na$) calc. 380.1626, found 380.1636.

2.2.3.5 *rac-trans*-2,3-3-(4-methoxybenzyl)-2-(4-methoxyphenyl)-1-methyl-2,3-dihydroquinolin-4(1H)-one (**2c**)

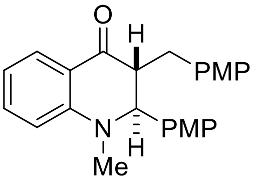

GP 7 starting from **1c**, room temperature, 18 hours, CH/EA 20:1, yellow solid (91 mg, 2.35 mmol, 96%)

^{1}H-NMR (700 MHz, 298 K, $CDCl_3$): δ = 7.88 – 7.85 (m, 1H, H_{Ar}), 7.51 – 7.48 (m, 1H, H_{Ar}), 7.20 – 7.17 (m, 2H, H_{Ar}), 6.93 – 6.90 (m, 2H, H_{Ar}), 6.78 – 6.74 (m, 4H, H_{Ar}), 6.71 – 6.67 (m, 2H, H_{Ar}), 4.21 (d, $^3J_{HH}$ = 1.8 Hz, 1H, NC*H*), 3.83 (s, 3H, OC*H_3*) 3.70 (s, 3H, OC*H_3*), 3.02 (s, 3H, NC*H_3*), 2.98 (dd, $^2J_{HH}$ = 13.4 Hz, $^3J_{HH}$ = 4.8 Hz, 1H, COCHC*H_2^A*), 2.89 (ddd, $^3J_{HH}$ = 11.1, 4.8, 2.0 Hz, 1H COC*H*), 2.80 (dd, $^2J_{HH}$ = 13.4 Hz, $^3J_{HH}$ = 11.2 Hz, 1H, COCHC*H_2^B*); **^{13}C-NMR** (176 MHz, 298 K, $CDCl_3$): δ = 195.4 (C_q),158.9 (C_q), 158.5 (C_q), 150.2 (C_q), 136.3 (CH), 131.0 (C_q), 130.6 (C_q), 130.3 (CH), 128.5 (CH), 127.5 (CH), 118.4 (C_q), 116.5 (CH), 114.3 (CH), 112.1 (CH), 65.6 (CH, N*C*H), 56.3 (CH, CO*C*H), 55.4 (CH_3, O*C*H_3), 55.3 (CH_3, O*C*H_3), 38.4 (CH_3, N*C*H_3), 36.3 (CH_2); **^{15}N-NMR** (71 MHz, 298 K, $CDCl_3$): δ = 62.4; **HRMS** (ESI+, MeCN) *m/z* $[M+Na]^+$ ($C_{25}H_{25}NO_3Na$) calc. 410.1732, found 410.1725.

2.2.3.6 *rac-trans*-2,3-2-(4-fluorophenyl)-3-(4-methoxybenzyl)-1-methyl-2,3-dihydroquinolin-4(1H)-one (**2d**)

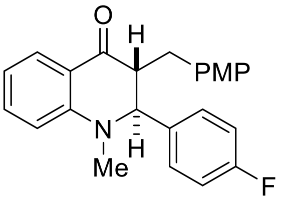

GP 7 starting from **1d**, room temperature, 30 minutes, CH/EA 20:1, yellow solid (91 mg, 0.242 mmol, 97%).

^{1}H-NMR (700 MHz, 298 K, $CDCl_3$): δ = 7.88 – 7.85 (m, 1H, H_{Ar}), 7.53 – 7.49 (m, 1H, H_{Ar}), 7.21 – 7.17 (m, 2H, H_{Ar}), 6.94 – 6.91 (m, 2H, H_{Ar}), 6.87 – 6.83 (m, 2H, H_{Ar}), 6.81 – 6.76 (m, 4H, H_{Ar}), 4.24 (d, $^3J_{HH}$ = 1.3 Hz, 1H, NC*H*), 3.83 (s, 3H, OC*H_3*), 3.03 (s, 3H, NC*H_3*), 2.99 (dd, $^2J_{HH}$ = 13.3 Hz, $^3J_{HH}$ = 4.7 Hz, 1H, COCHC*H_2^A*), 2.90 (ddd, $^3J_{HH}$ = 11.3, 4.6, 1.8 Hz, 1H COC*H*), 2.85 (dd, $^2J_{HH}$ = 13.3 Hz, $^3J_{HH}$ = 11.4 Hz, 1H, COCHC*H_2^B*); **^{13}C-NMR** (176 MHz, 298 K, $CDCl_3$): δ = 195.0 (C_q), 162.8 (d, $^1J_{CF}$ = 245.5 Hz, C_q), 158.6 (C_q), 150.0 (C_q), 136.5 (CH), 134.9 (d, $^4J_{CF}$ = 2.4 Hz, C_q), 130.4 (C_q), 130.3 (CH), 128.5 (CH), 128.0 (d, $^3J_{CF}$ = 8.5 Hz CH), 118.3 (C_q), 116.8 (CH), 115.8 (d, $^2J_{CF}$ = 21.6 Hz, CH), 114.4 (CH), 112.1 (CH), 65.5 (CH, N*C*H), 56.4 (CH, CO*C*H), 55.4 (CH_3, OCH_3), 38.4 (CH_3, N*C*H_3), 36.3 (CH_2); **^{19}F-NMR** (282 MHz, 298 K, $CDCl_3$): δ = −115.1 – 115.2 (m, 1F); **^{15}N-NMR**

(71 MHz, 298 K, $CDCl_3$): δ = 61.2; **HRMS** (ESI+, MeOH) *m/z* $[M+Na]^+$ ($C_{24}H_{22}NO_2FNa$) calc. 398.1525, found 398.1532.

2.2.3.7 *rac-trans*-2,3-3-(4-methoxybenzyl)-1,3-dimethyl-2-phenyl-2,3-dihydroquinolin-4(1H)-one (**2e**)

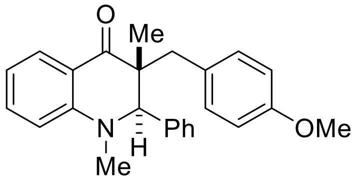

GP 7 starting from **1e**, room temperature, 30 minutes, CH/EA 25:1, yellow solid (82 mg, 0.221 mmol, 88%).

^{1}H-NMR (700 MHz, 298 K, $CDCl_3$): δ = 7.98 – 7.95 (m, 1H, H_{Ar}), 7.49 – 7.45 (m, 1H, H_{Ar}), 7.20 – 7.16 (m, 1H, H_{Ar}), 7.14 – 7.10 (m, 2H, H_{Ar}), 7.09 – 7.06 (m, 2H, H_{Ar}), 6.95 – 6.92 (m, 2H, H_{Ar}), 6.83 – 6,79 (m, 1H, H_{Ar}), 6.72 – 6.68 (m, 1H, H_{Ar}), 3.98 (s, 1H, NC*H*), 3.83 (s, 3H, OC*H*3), 3.06 (d, $^2J_{HH}$ = 13.4 Hz, 1H, $COC(CH_3)CH_2^A$), 2.93 (s, 3H, NC*H*3), 2.83 (d, $^2J_{HH}$ = 13.4 Hz, 1H, $COC(CH_3)CH_2^B$), 0.86 (s, 3H, COC(C*H*3)); **^{13}C-NMR** (176 MHz, 298 K, $CDCl_3$): δ = 198.3 (C_q), 158.7 (C_q), 150.3 (C_q), 137.8 (C_q), 135.8 (CH), 131.8 (CH), 128.6 (CH), 128.43 (C_q), 128.41 (CH), 128.1 (CH), 127.6 (CH), 118.4 (C_q), 116.7 (CH), 113.7 (CH), 112.2 (CH), 73.2 (CH, N*C*H), 55.4 (CH_3, O*C*H3), 50.5 (C_q, CO*C*(CH3)), 43.5 (CH_2, $COC(CH_3)CH_2$), 37.9 (CH_3, N*C*H3), 18.2 (CH_3, COC(*C*H3)); **^{15}N-NMR** (71 MHz, 298 K, $CDCl_3$): δ = 68.4; **HRMS** (ESI+, MeOH) *m/z* $[M+Na]^+$ ($C_{25}H_{25}NO_2Na$) calc. 394.1783, found 394.1791.

2.2.3.8 *rac*-3-(4-methoxybenzyl)-1,2,2-trimethyl-2,3-dihydroquinolin-4(1H)-one (**2f**)

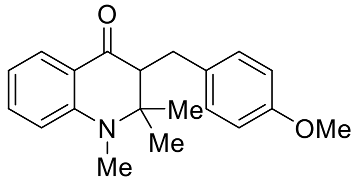

GP 7 starting from **1f** (81 mg, 0.262 mmol, 1.00 equiv.), room temperature, 18 hours, CH/EA 10:1, yellow oil (53 mg, 0.171 mmol, 65%).

^{1}H-NMR (700 MHz, 298 K, $CDCl_3$): δ = 7.81 – 7.78 (m, 1H, H_{Ar}), 7.43 – 7.39 (m, 1H, H_{Ar}), 6.96 – 6.94 (m, 2H, H_{Ar}), 6.78 – 6.75 (m, 3H, H_{Ar}), 6.75 – 6.72 (m, 1H, H_{Ar}), 3.76 (s, 3H, OC*H*3), 2.97 (dd, $^2J_{HH}$ = 14.3 Hz, $^3J_{HH}$ = 4.9 Hz, 1H, $COCHCH_2^A$), 2.94 (s, 3H, NC*H*3), 2.76 (dd, $^2J_{HH}$ = 14.2 Hz, $^3J_{HH}$ = 9.7 Hz, 1H, $COCHCH_2^B$), 2.48 (dd, $^3J_{HH}$ = 9.7, 4.8, 1H, COCH), 1.44 (s, 3H, $C(CH_3)_2^A$), 1.18 (s, 3H, $C(CH_3)_2^B$); **^{13}C-NMR** (176 MHz, 298 K, $CDCl_3$): δ = 196.5 (C_q), 158.1 (C_q), 150.2 (C_q), 135.4 (CH), 131.5 (C_q), 130.1 (CH), 128.0 (CH), 119.3 (C_q), 116.6 (CH), 113.8 (CH), 113.4 (CH), 60.3 (C_q, *C*(CH3)2), 60.1 (CH), 55.3 (CH_3, O*C*H3), 32.1 (CH_2), 31.3 (CH_3, N*C*H3), 25.1 (CH_3, $C(CH_3)_2^A$), 22.1 (CH_3, $C(CH_3)_2^B$); **^{15}N-NMR** (71 MHz, 298 K, $CDCl_3$): δ = 74.3; **HRMS** (ESI+, MeCN) *m/z* $[M+Na]^+$ ($C_{22}H_{27}NO_2Na$) calc. 332.1626, found 332.1616.

2.2.3.9 *rac-trans*-2,3-2-(tert-butyl)-3-(4-methoxybenzyl)-1-methyl-2,3-dihydroquinolin-4(1H)-one (**2g**)

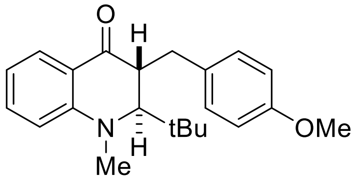

GP 7 starting from **1g**, room temperature, 30 minutes, CH/EA 25:1, yellow solid (78 mg, 0.231 mmol, 92%).

^{1}H-NMR (700 MHz, 298 K, $CDCl_3$): δ = 7.84 – 7.81 (m, 1H, H_{Ar}), 7.42 – 7.38 (m, 1H, H_{Ar}), 7.11 – 7.08 (m, 2H, H_{Ar}), 6.87 – 6.85 (m, 2H, H_{Ar}), 6.67 – 6.64 (m, 1H, H_{Ar}), 6.63 – 6.61 (m, 1H, H_{Ar}), 3.81 (s, 3H, OCH_3), 3.15 (s, 3H, NCH_3), 2.93 (dd, $^{2}J_{HH}$ = 13.4 Hz, $^{3}J_{HH}$ = 4.7 Hz, 1H, $COCHCH_2{}^{A}$), 2.89 (ddd, $^{3}J_{HH}$ = 11.2, 4.7, 0.9 Hz, 1H, COCH), 2.74 (d, $^{3}J_{HH}$ = 1.1 Hz, 1H, NC*H*), 2.55 (dd, $^{2}J_{HH}$ = 13.4 Hz, $^{3}J_{HH}$ = 11.1 Hz, $COCHCH_2{}^{B}$), 0.78 (s, 9H, $NCHC(CH_3)_3$); **^{13}C-NMR** (176 MHz, 298 K, $CDCl_3$): δ = 197.1 (C_q), 158.5 (C_q), 150.4 (C_q), 136.0 (CH), 130.6 (C_q), 130.3 (CH), 127.8 (CH), 118.0 (C_q), 115.8 (CH), 114.2 (CH), 112.5 (CH), 77.8 (CH, N*C*H), 55.4 (CH_3, OCH_3), 49.1 (CH, CO*C*H), 43.2 (CH_3, NCH_3), 38.4 (C_q, $C(CH_3)_3$), 37.5 (CH_2), 28.0 (CH_3, $C(CH_3)_3$); **^{15}N-NMR** (71 MHz, 298 K, $CDCl_3$): δ = 59.5; **HRMS** (ESI+, MeCN) *m/z* $[M+Na]^+$ ($C_{22}H_{27}NO_2Na$) calc. 360.1939, found 360.1943.

2.2.3.10 *rac-trans*-2,3-2-(furan-2-yl)-3-(4-methoxybenzyl)-1-methyl-2,3-dihydroquinolin-4(1H)-one (**2h**)

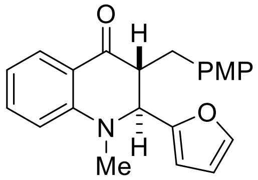

GP 7 starting from **1h**, room temperature, 18 hours, CH/EA 20:1, yellow solid (73 mg, 0.210 mmol, 84%).

^{1}H-NMR (700 MHz, 298 K, $CDCl_3$): δ = 7.92 – 7.83 (m, 1H, H_{Ar}), 7.75 – 7.74 (m, 1H, H_{Ar}), 7.22 – 7.20 (m, 1H, H_{Ar}), 7.19 – 7.17 (m, 2H, H_{Ar}), 6.91 – 6.87 (m, 2H, H_{Ar}), 6.78 – 6.74 (m, 1H, H_{Ar}), 6.71 – 6.68 (m, 1H, H_{Ar}), 6.15 – 6.13 (m, 1H, H_{Ar}), 5.93 – 5.91 (m, 1H, H_{Ar}), 4.22 (bs, 1H, NC*H*), 3.81 (s, 3H, OCH_3), 3.12 (s, 3H, NCH_3), 3.03 – 3.00 (m, 1H, COC*H*), 2.97 (dd, $^{2}J_{HH}$ = 13.7 Hz, $^{3}J_{HH}$ = 4.8 Hz, 1H, $COCHCH_2{}^{A}$), 2.82 (dd, $^{2}J_{HH}$ = 13.6 Hz, $^{3}J_{HH}$ = 11.7 Hz, 1H, $COCHCH_2{}^{B}$); **^{13}C-NMR** (176 MHz, 298 K, $CDCl_3$): δ = 195.3 (C_q), 158.6 (C_q), 152.6 (C_q), 149.3 (C_q), 142.3 (CH), 136.0 (CH), 130.34 (CH), 130.32 (C_q), 128.4 (CH), 118.3 (C_q), 117.3 (CH), 114.3 (CH), 113.0 (CH), 110.2 (CH), 117.8 (CH), 60.3 (CH, N*C*H), 55.4 (CH_3, OCH_3), 52.3 (CH, CO*C*H), 38.8 (CH_3, NCH_3), 34.9 (CH_2); **^{15}N-NMR** (71 MHz, 298 K, $CDCl_3$): δ = 61.4; **HRMS** (ESI+, MeOH) *m/z* $[M+Na]^+$ ($C_{22}H_{21}NO_3Na$) calc. 370.1419, found 370.1441.

2.2.3.11 *rac-trans*-2,3-3-(4-methoxybenzyl)-1-methyl-2-(thiophen-2-yl)-2,3-dihydroquinolin-4(1H)-one (**2i**)

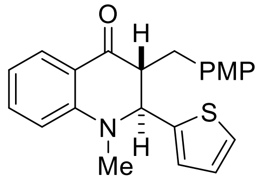

GP 7 starting from **1i**, room temperature, 18 hours, CH/EA 20:1, yellow solid (76 mg, 0.209 mmol, 84%).

^{1}H-NMR (700 MHz, 298 K, $CDCl_3$): δ = 7.94 – 87.91 (m, 1H, H_{Ar}), 7.49 – 7.46 (m, 1H, H_{Ar}), 7.20 – 7.17 (m, 2H, H_{Ar}), 7.05 – 7.03 (m, 1H, H_{Ar}), 6.92 – 6.89 (m, 2H, H_{Ar}), 6.81 – 6.77 (m, 2H, H_{Ar}), 6.73 – 6.71 (m, 1H, H_{Ar}), 6.66 – 6.63 (m, 1H, H_{Ar}), 4.43 (dd, $^3J_{HH}$ = 1.8 Hz, $^4J_{HH}$ = 0.8 Hz, 1H, NC*H*), 3.82 (s, 3H, OC*H*$_3$), 3.07 (s, 3H, NC*H*$_3$), 3.02 (ddd, $^3J_{HH}$ = 11.1, 5.0, 1.9 Hz, 1H, COC*H*), 2.99 (dd, $^2J_{HH}$ = 13.3 Hz, $^3J_{HH}$ = 5.0 Hz, 1H, COCHC*H*$_2^A$), 2.83 (dd, $^2J_{HH}$ = 13.3 Hz, $^3J_{HH}$ = 11.1 Hz, 1H, COCHC*H*$_2^B$); **^{13}C-NMR** (176 MHz, 298 K, $CDCl_3$): δ = 195.1 (C_q), 158.6 (C_q), 149.3 (C_q), 142.1 (C_q), 136.2 (CH), 130.33 (C_q), 130.28 (CH), 128.5 (CH), 126.7 (CH), 125.4 (CH), 124.7 (CH) 118.6 (C_q), 117.4 (CH), 114.4 (CH), 113.1 (CH), 62.7 (CH, N*C*H), 56.0 (CH, CO*C*H), 55.4 (CH_3, O*C*H$_3$), 38.5 (CH_3, N*C*H$_3$), 35.6 (CH_2); **^{15}N-NMR** (71 MHz, 298 K, $CDCl_3$): δ = 64.5; **HRMS** (ESI+, MeOH) *m/z* $[M+Na]^+$ ($C_{22}H_{21}NO_2SNa$) calc. 386.1191, found 386.1206.

2.2.3.12 *rac-trans*-2,3-3-(4-methoxybenzyl)-1-methyl-2-(pyridin-2-yl)-2,3-dihydroquinolin-4(1H)-one (**2j**)

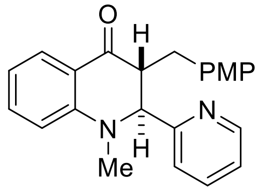

GP 7 starting from **1j**, room temperature, 1 hour, CH/EA 5:1, yellow solid (86 mg, 0.240 mmol, 96%). Suitable crystals for X-ray single crystal structure analysis were grown by condensation of *n*-pentane into the chloroform solution.

^{1}H-NMR (700 MHz, 298 K, $CDCl_3$): δ = 8.49 – 8.46 (m, 1H, H_{Ar}), 7.86 – 7.83 (m, 1H, H_{Ar}), 7.52 – 7.47 (m, 1H, H_{Ar}), 7.44 – 7.40 (m, 1H, H_{Ar}), 7.22 – 7.19 (m, 2H, H_{Ar}), 7.07 – 7.03 (m, 1H, H_{Ar}), 6.91 – 6.87 (m, 2H, H_{Ar}), 6.80 – 6.78 (m, 2H, H_{Ar}), 6.77 – 6.73 (m, 1H, H_{Ar}), 4.22 (bs, 1H, NC*H*), 3.82 – 3.79 (m, 3H, OC*H*$_3$), 3.28 – 3.24 (m, 1H, COC*H*), 3.12 – 3.10 (m, 3H, NC*H*$_3$), 3.03 (dd, $^2J_{HH}$ = 13.8 Hz, $^3J_{HH}$ = 5.7 Hz, 1H, COCHC*H*$_2^A$), 2.87 (dd, $^2J_{HH}$ = 13.5 Hz, $^3J_{HH}$ = 11.1 Hz, 1H, COCHC*H*$_2^B$); **^{13}C-NMR** (176 MHz, 298 K, $CDCl_3$): δ = 195.3 (C_q), 158.8 (C_q), 158.6 (C_q), 150.3 (CH), 149.8 (C_q), 136.6 (CH), 136.2 (CH), 130.4 (CH), 130.3 (C_q), 128.5 (CH), 122.3 (CH), 120.5 (CH), 118.6 (C_q), 116.8 (CH), 114.3 (CH), 112.2 (CH), 68.1 (CH, N*C*H), 55.4 (CH_3, O*C*H$_3$), 53.7 (CH, CO*C*H), 38.7 (CH_3, N*C*H$_3$), 36.2 (CH_2); **^{15}N-NMR** (71 MHz, 298 K,

CDCl$_3$): δ = 59.5, 311.9 (N-Pyr); **HRMS** (ESI+, MeOH) *m/z* [M+Na]$^+$ ($C_{23}H_{22}N_2O_2Na$) calc. 381.1579, found 381.1577.

2.2.3.13 *rac-trans*-2,3-1-ethyl-3-(4-methoxybenzyl)-2-phenyl-2,3-dihydroquinolin-4(1H)-one (**2k**)

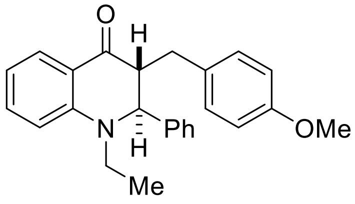

GP 7 starting from **1k**, room temperature, 30 minutes, CH/EA 25:1, yellow solid (82 mg, 0.221 mg, 88%)

^{1}H-NMR (700 MHz, 298 K, CDCl$_3$): δ = 78.87 – 7.83 (m, 1H, H$_{Ar}$), 7.50 – 7.36 (m, 1H, H$_{Ar}$), 7.23 – 7.20 (m, 2H, H$_{Ar}$), 7.17 – 7.13 (m, 3H, H$_{Ar}$), 6.94 – 6.90 (m, 2H, H$_{Ar}$), 6.88 – 6.86 (m, 2H, H$_{Ar}$), 6.84 – 6.81 (m, 1H, H$_{Ar}$), 6.74 – 6.70 (m, 1H, H$_{Ar}$), 4.33 (s, 1H, NC*H*), 3.83 (s, 3H, OC*H*$_3$), 3.58 (dd, $^2J_{HH}$ = 14.3, $^3J_{HH}$ = 7.4 Hz,1H, NC*H*$_2$^{A}), 3.13 (dd, $^2J_{HH}$ = 14.6, $^3J_{HH}$ = 7.4 Hz, 1H, NC*H*$_2$^{B}), 2.98 (dd, $^2J_{HH}$ = 13.6 Hz, $^3J_{HH}$ = 4.7 Hz, 1H, COCHC*H*$_2$^{A}), 2.94 (dd, $^3J_{HH}$ = 10.9, 4.8 Hz, 1H, COC*H*), 2.82 – 2.77 (m, 1H, COCHC*H*$_2$^{B}), 1.23 (t, $^3J_{HH}$ = 7.1, 3H, NCH$_2$C*H*$_3$); **^{13}C-NMR** (176 MHz, 298 K, CDCl$_3$): δ = 195.0 (C$_q$), 158.6 (C$_q$), 149.5 (C$_q$), 140.2 (C$_q$), 136.3 (CH), 130.4 (C$_q$), 130.3 (CH), 128.9 (CH), 128.7 (CH), 127.5 (CH), 126.4 (CH), 118.1 (C$_q$), 116.1 (CH), 114.3 (CH), 111.6 (CH), 64.01 (CH, N*C*H), 55.7 (CH, CO*C*H), 55.4 (CH$_3$, OCH$_3$), 45.5 (CH$_2$, N*C*H$_2$), 32.4 (CH$_2$, COCH*C*H$_2$), 13.2 (CH$_3$, NCH$_2$*C*H$_3$); **^{15}N-NMR** (71 MHz, 298 K, CDCl$_3$): δ = 76.6; **HRMS** (ESI+, MeOH) *m/z* [M+Na]$^+$ ($C_{25}H_{25}NO_2Na$) calc. 394.1783, found 394.1770.

2.2.3.14 *rac-trans*-2,3-1-isobutyl-3-(4-methoxybenzyl)-2-phenyl-2,3-dihydroquinolin-4(1H)-one (**2l**)

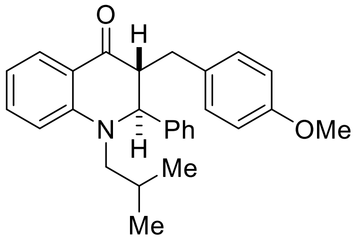

GP 7 starting from **1l**, room temperature, 30 minutes, CH/EA 40:1, then 25:1, yellow solid (61 mg, 1.53 mmol, 61%).

^{1}H-NMR (700 MHz, 298 K, CDCl$_3$): δ = 7.86 – 7.83 (m, 1H, H$_{Ar}$), 7.48 – 7.44 (m, 1H, H$_{Ar}$), 7.21 – 7.14 (m, 5H, H$_{Ar}$), 6.93 – 6.88 (m, 4H, H$_{Ar}$), 6.88 – 6.84 (m, 1H, H$_{Ar}$), 6.74 – 7.70 (m, 1H, H$_{Ar}$), 4.46 (s, 1H, NC*H*), 3.83 (s, 3H, OC*H*$_3$), 3.41 (dd, $^2J_{HH}$ = 15.0 Hz, $^3J_{HH}$ = 7.5 Hz, COCHC*H*$_2$^{A}), 3.06 – 3.01 (m, 2H, COC*H*, NC*H*$_2$^{A}), 2.97 – 2.93 (m, 1H, NC*H*$_2$^{B}), 2.70 (dd, $^2J_{HH}$ = 15.0 Hz, $^3J_{HH}$ = 7.2 Hz, 1H, COCHC*H*$_2$^{B}), 2.08 – 2.01 (m, 1H, NCH$_2$C*H*) 1.04 (d, $^3J_{HH}$ = 6.7 Hz, 3H, NCH$_2$CH(C*H*$_3$)$_2$^{A}), 0.99 (d, $^3J_{HH}$ = 6.5 Hz, 3H, NCH$_2$CH(C*H*$_3$)$_2$^{B}); **^{13}C-NMR** (176 MHz, 298 K, CDCl$_3$): δ = 194.6 (C$_q$), 158.5 (C$_q$), 150.7 (C$_q$), 139.9 (C$_q$), 136.1 (CH), 130.3 (C$_q$), 130.1 (CH), 128.9 (CH), 128.6 (CH), 127.5 (CH), 126.5 (CH),

118.3 (C_q), 116.2 (CH), 114.2 (CH), 112.1 (CH), 65.3 (CH, N*C*H), 58.2 (CH_2, COCH*C*H_2), 55.8 (CH, CO*C*H), 55.4 (CH, O*C*H_3), 36.6 (CH_2, N*C*H_2), 28.1 (CH, NCH_2*C*H), 20.79 (CH_3, $NCH_2CH(CH_3)_2^A$), 20.75 (CH_3, $NCH_2CH(CH_3)_2^B$); **^{15}N-NMR** (71 MHz, 298 K, $CDCl_3$): δ = 75.3; **HRMS** (ESI+, MeOH) *m/z* $[M+Na]^+$ ($C_{27}H_{29}NO_2Na$) calc. 422.2096, found 422.2113.

2.2.3.15 *rac-trans*-2,3-3-(*cis*-1-(4-chlorophenyl)ethyl)-1-methyl-2-phenyl-2,3-dihydroquinolin-4(1H)-one (*rac*-**2m**)

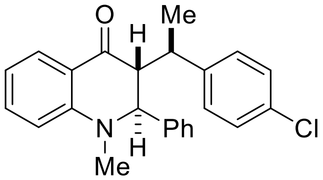

GP 7 starting from *rac*-**2m**, room temperature, 30 minutes, CH/EA 40:1, light yellow solid (84 mg, 0.223 mmol, 89%).

^{1}H-NMR (700 MHz, 298 K, CD_2Cl_2): δ = 7.77 – 7.72 (m, 1H, H_{Ar}), 7.53 – 7.49 (m, 1H, H_{Ar}), 7.43 – 7.39 (m, 2H, H_{Ar}), 7.31 – 7.29 (m, 2H, H_{Ar}), 7.19 – 7.15 (m, 3H, H_{Ar}), 7.19 – 7.14 (m, 3H, H_{Ar}), 6.82 – 6.78 (m, 1H, H_{Ar}), 4.06 (d, $^3J_{HH}$ = 1.7 Hz, 1H, NC*H*), 3.07 (dq, $^3J_{HH}$ = 11.2, 7.0 Hz, 1H, COCHC*H*(CH_3)), 2.93 (s, 3H, NC*H*$_3$), 2.68 (dd, $^3J_{HH}$ = 11.2, 1.9 Hz, 1H, COCH), 1.22 (d, $^3J_{HH}$ = 7.1 Hz, 3H, COCHCH(C*H*$_3$)); **^{13}C-NMR** (176 MHz, 298 K, CD_2Cl_2): δ = 194.7 (C_q), 150.3 (C_q), 143.7 (C_q), 139.2 (C_q), 136.4 (CH), 132.9 (C_q) 129.5 (CH), 129.4 (CH), 129.1 (CH), 128.2 (CH), 127.7 (CH), 126.3 (CH), 118.8 (C_q), 116.6 (CH), 112.2 (CH), 67.0 (CH, N*C*H), 61.2 (CH, CO*C*H), 40.1 (CH, COCH*C*H(CH_3)), 38.5 (CH_3, N*C*H_3), 20.1 (CH_3, NCHCH(C*H*$_3$)); **^{15}N-NMR** (71 MHz, 298 K, CD_2Cl_2): δ = 61.4; **HRMS** (ESI+, MeOH) *m/z* $[M+Na]^+$ ($C_{24}H_{22}NONaCl$) calc. 398.1288, found 398.1284.

2.2.3.16 *rac-trans*-2,3-3-(cis-1-(4-bromophenyl)ethyl)-1-methyl-2-phenyl-2,3-dihydroquinolin-4(1H)-one (*rac*-**2n**)

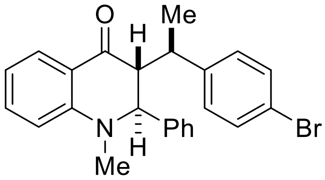

GP 7 starting from *rac*-**1n**, room temperature, 30 minutes, CH/EA 40:1, light yellow solid (91 mg, 0.216 mmol, 87%).

^{1}H-NMR (700 MHz, 298 K, $CDCl_3$): δ = 7.84 – 7.80 (m, 1H, H_{Ar}), 7.57 – 7.54 (m, 2H, H_{Ar}), 7.52 – 7.49 (m, 1H, H_{Ar}), 7.22 – 7.19 (m, 2H, H_{Ar}), 7.17 – 7.12 (m, 3H, H_{Ar}), 6.78 – 6.74 (m, 4H, H_{Ar}), 4.04 (d, $^3J_{HH}$ = 1.7 Hz, 1H, NC*H*), 3.07 (dq, $^3J_{HH}$ = 11.2, 7.0 Hz, 1H, COCHC*H*(CH_3)), 2.93 (s, 3H, NC*H*$_3$), 2.72 (dd, $^3J_{HH}$ = 11.2, 1.8 Hz, 1H, COCH), 1.23 (d, $^3J_{HH}$ = 7.0 Hz, 3H, COCHCH(C*H*$_3$)); **^{13}C-NMR** (176 MHz, 298 K, $CDCl_3$): δ = 194.7 (C_q), 150.0 (C_q), 143.9 (C_q), 138.6 (C_q), 136.3 (CH), 132.2 (CH) 129.4 (CH), 129.0 (CH), 128.4 (CH), 127.6 (CH), 126.0 (CH), 120.9 (C_q), 118.5 (C_q), 116.5 (CH), 111.8 (CH), 66.9 (CH, N*C*H), 60.8 (CH, CO*C*H), 40.0 (CH, COCH*C*H(CH_3)), 38.4 (CH_3, NCH_3), 20.1 (CH_3, COCHCH(*C*H_3)); **^{15}N-NMR** (71 MHz,

298 K, $CDCl_3$): δ = 61.4; **HRMS** (ESI+, MeOH) *m/z* $[M+Na]^+$ ($C_{24}H_{22}NOBrNa$) calc. 442.0782, found 442.0768.

HPLC (IA, 15 °C, heptane/i-PrOH: 95/5, flow rate: 0.7 ml/min, 234 nm).

Chromatogram : GWI-709b-1_channel5

System : LC920_2
Method : NOT DEFINED
User : AKP

Acquired : 27.10.2021 13:05:10
Processed : 27.10.2021 15:12:26
Printed : 28.10.2021 12:51:53

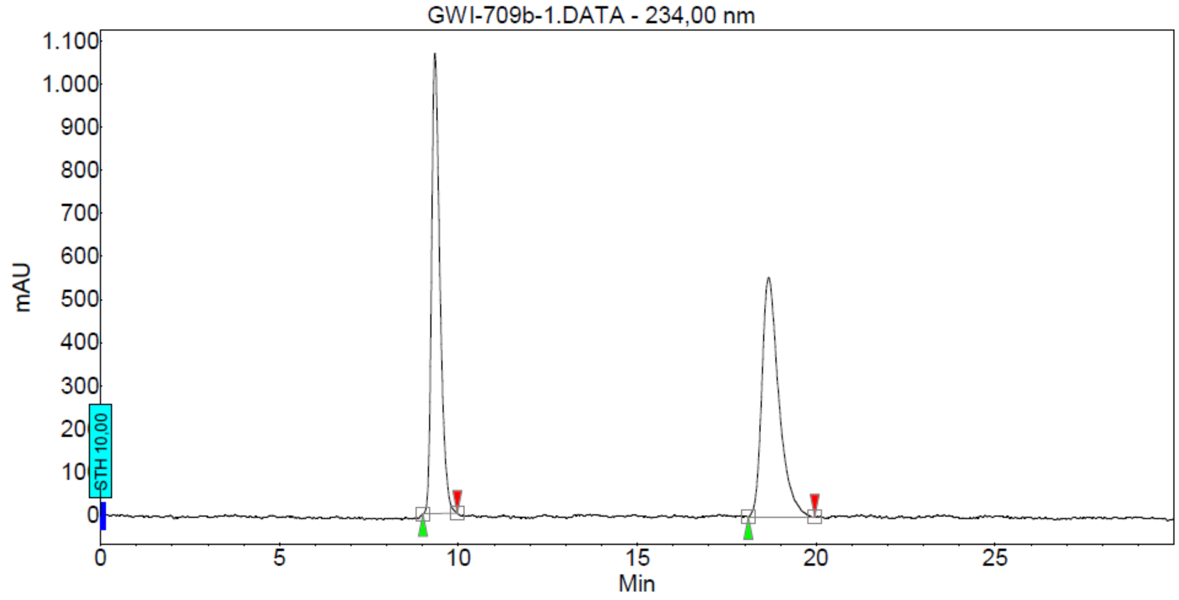

Peak results :

Index	Name	Time [Min]	Quantity [% Area]	Height [mAU]	Area [mAU.Min]	Area % [%]
1	UNKNOWN	9,35	48,78	1069,2	290,4	48,777
2	UNKNOWN	18,67	51,22	556,1	304,9	51,223
Total			100,00	1625,2	595,3	100,000

2.2.3.17 (*2S,3R*)-3-((*R*)-1-(4-bromophenyl)ethyl)-1-methyl-2-phenyl-2,3-dihydroquinolin-4(1H)-one ((*2S,3R*)-**2n**)

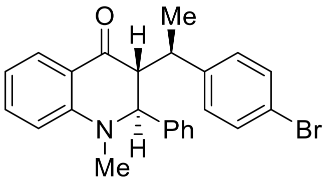

GP 7 starting from (*S*)-**1n**, room temperature, 30 minutes, CH/EA 40:1, light yellow solid (86 mg, 0.205 mmol, 82%).

^{1}H-NMR (700 MHz, 298 K, $CDCl_3$): δ = 7.84 – 7.80 (m, 1H, H_{Ar}), 7.56 – 7.53 (m, 2H, H_{Ar}), 7.52 – 7.49 (m, 1H, H_{Ar}), 7.22 – 7.19 (m, 2H, H_{Ar}), 7.16 – 7.11 (m, 3H, H_{Ar}), 6.79 – 6.74 (m, 4H, H_{Ar}), 4.04 (d, $^3J_{HH}$ = 1.3 Hz, 1H, NC*H*), 3.07 (dq, $^3J_{HH}$ = 11.2, 7.0 Hz, 1H, COCHC*H*(CH_3)), 2.93 (s, 3H, NC*H_3*), 2.72 (dd, $^3J_{HH}$ = 11.2, 1.8 Hz, 1H, COCH), 1.22 (d, $^3J_{HH}$ = 7.0 Hz, 3H, COCHCH(C*H_3*)); **^{13}C-NMR**

(176 MHz, 298 K, $CDCl_3$): δ = 194.7 (C_q), 150.0 (C_q), 143.9 (C_q), 138.6 (C_q), 136.3 (CH), 132.2 (CH) 129.4 (CH), 129.0 (CH), 128.4 (CH), 127.6 (CH), 126.0 (CH), 120.9 (C_q), 118.5 (C_q), 116.6 (CH), 111.8 (CH), 66.9 (CH, N*C*H), 60.8 (CH, CO*C*H), 40.0 (CH, COCH*C*H(CH_3)), 38.4 (CH_3, NCH_3), 20.1 (CH_3, COCHCH($\mathit{C}H_3$)); **^{15}N-NMR** (71 MHz, 298 K, $CDCl_3$): δ = 61.8; **HRMS** (ESI+, MeCN) *m/z* $[M]^+$ ($C_{24}H_{22}NOBr$) calc. 419.0884, found 419.0885.

HPLC (IA, 15 °C, heptane/i-PrOH: 95/5, flow rate: 0.7 ml/min, 234 nm).

Chromatogram : GWI-488b-1_channel5

System : LC920_2
Method : NOT DEFINED
User : AKP

Acquired : 27.10.2021 14:18:19
Processed : 27.10.2021 15:12:55
Printed : 28.10.2021 12:52:10

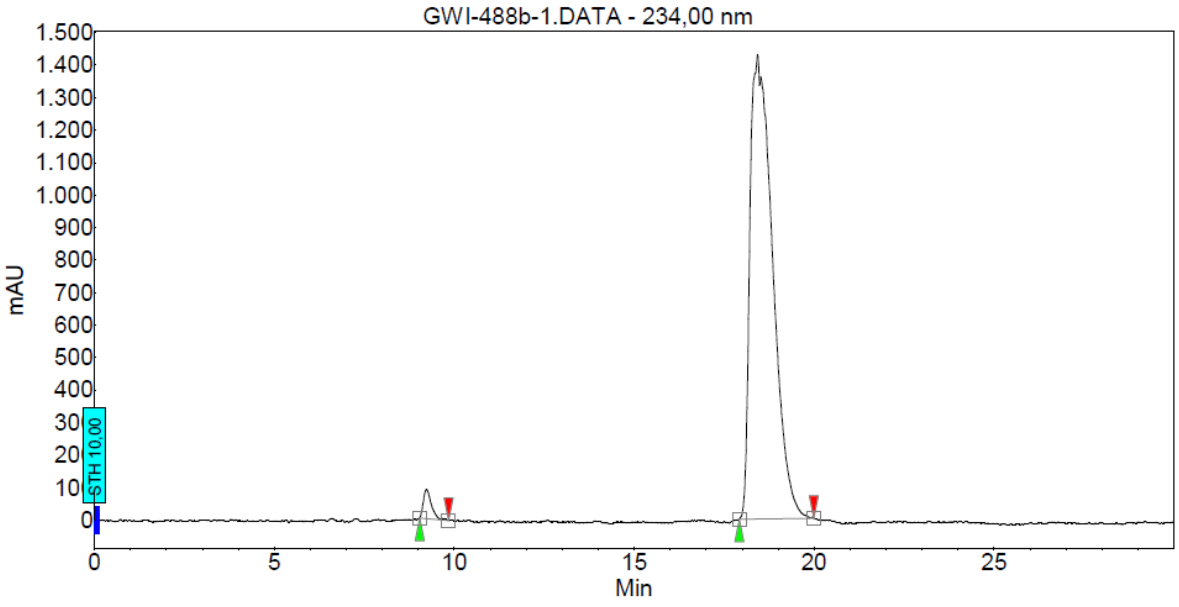

Peak results :

Index	Name	Time [Min]	Quantity [% Area]	Height [mAU]	Area [mAU.Min]	Area % [%]
1	UNKNOWN	9,23	2,22	89,6	23,6	2,218
2	UNKNOWN	18,43	97,78	1429,2	1039,8	97,782
Total			100,00	1518,8	1063,4	100,000

2.2.3.18 *rac-trans*-2,3-1-methyl-2-phenyl-3-(*cis*-1-(4-(trifluoromethyl)phenyl)ethyl)-2,3-dihydroquinolin-4(1H)-one (*rac*-**2o**)

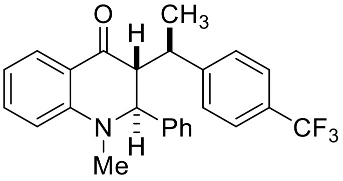

GP 7 starting from *rac*-**1o**, room temperature, 1 hour, CH/EA 40:1, light yellow solid (70 mg, 0.171 mmol, 78%)

^{1}H-NMR (700 MHz, 298 K, $CDCl_3$): δ = 7.85 – 7.82 (m, 1H, H_{Ar}), 7.70 – 7.68 (m, 2H, H_{Ar}), 7.53 – 7.50 (m, 1H, H_{Ar}), 7.46 – 7.43 (m, 2H, H_{Ar}), 7.17 – 7.12 (m, 3H, H_{Ar}), 6.79 – 6.76 (m, 4H, H_{Ar}), 4.01 (d, $^3J_{HH}$ = 1.4 Hz, 1H, NC*H*), 3.19 (dq, $^3J_{HH}$ = 11.2, 7.0 Hz, 1H, COCHC*H*(CH_3)), 2.93 (s, 3H, NCH_3), 2.80 (dd, $^3J_{HH}$ = 11.2, 1.8 Hz, 1H, COCH), 1.27 (d, $^3J_{HH}$ = 7.1 Hz, 3H, COCHCH(CH_3)); **^{13}C-NMR** (176 MHz, 298 K, $CDCl_3$): δ = 194.5 (C_q), 150.0 (C_q), 149.1 (C_q), 138.4 (C_q), 136.4 (CH), 139.5 (q, $^2J_{CF}$ = 32.5 Hz, C_q). 129.0 (CH), 128.4 (CH), 128.0 (CH), 127.7 (CH), 126.1 (q, $^3J_{CF}$ = 3.7 Hz, CH), 124.3 (q, $^1J_{CF}$ = 272.0 Hz, C_q), 118.6 (C_q), 116.7 (CH), 111.8 (CH), 66.9 (CH, N*C*H), 60.5 (CH, CO*C*H), 40.3 (CH, COCH*C*H(CH_3)), 38.4 (CH_3, NCH_3), 20.1 (CH_3, COCHCH(*C*H_3)); **^{19}F-NMR** (282 MHz, 298 K, $CDCl_3$): δ = −62.37 – 62.39 (m, 3F); **^{15}N-NMR** (71 MHz, 298 K, $CDCl_3$): δ = 61.5; **HRMS** (ESI+, MeOH) *m/z* $[M+Na]^+$ ($C_{24}H_{22}NONaCl$) calc. 398.1288, found 398.1284.

2.2.3.19 *rac-trans*-2,3-3-(4-fluorobenzyl)-1-methyl-2-phenyl-2,3-dihydroquinolin-4(1H)-one (**2p**)

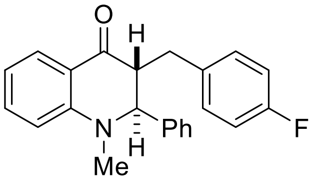

GP 7 starting from **1p**, 60 °C, 18 hours, CH/EA 40:1, yellow solid (76 mg, 0.220 mmol, 87%).

^{1}H-NMR (700 MHz, 298 K, $CDCl_3$): δ = 7.87 – 7.84 (m, 1H, H_{Ar}), 7.53 – 7.49 (m, 1H, H_{Ar}), 7.25 – 7.21 (m, 2H, H_{Ar}), 7.18 – 7.14 (m, 3H, H_{Ar}), 7.08 – 7.04 (m, 2H, H_{Ar}), 6.85 – 6.82 (m, 2H, H_{Ar}), 6.80 – 6.75 (m, 2H, H_{Ar}), 4.24 (bs, 1H, NC*H*), 3.04 – 3.01 (m, 4H, NCH_3, COCHCH_2^A) 3.03 (s, 3H, NCH_3), 2.95 – 2.92 (m, 1H, COC*H*), 2.87 (dd, $^2J_{HH}$ = 13.1 Hz, $^3J_{HH}$ = 11.0 Hz, 1H, COCHCH_2^B); **^{13}C-NMR** (176 MHz, 298 K, $CDCl_3$): δ = 194.8 (C_q), 162.9 (d, $^1J_{CF}$ = 245.1 Hz, C_q), 150.3 (C_q), 18.9 (C_q), 136.4 (CH), 134.3 (d, $^4J_{CF}$ = 3.2 Hz, C_q), 130.8 (d, $^3J_{CF}$ = 7.9 Hz, CH), 129.0 (CH), 128.5 (CH), 127.7 (CH), 126.4 (CH), 118.4 (C_q), 116.8 (CH), 115.8 (CH), 115.7 (CH), 112.2 (CH), 66.3 (CH, CO*C*H), 56.0 (CH, N*C*H), 38.5 (CH_3), 36.4 (CH);**^{19}F-NMR** (282 MHz, 298 K, $CDCl_3$): δ = −115.97 – 116.05 (m, 1F); **^{15}N-NMR** (71 MHz, 298 K, $CDCl_3$): δ = 61.4; **HRMS** (ESI+, MeOH) *m/z* $[M+Na]^+$ ($C_{23}H_{20}NOFNa$) calc. 368.1427, found 368.1417.

2.2.3.20 *rac-trans*-2,3-3-(4-fluorobenzyl)-2-(4-fluorophenyl)-1-methyl-2,3-dihydroquinolin-4(1H)-one (**2q**)

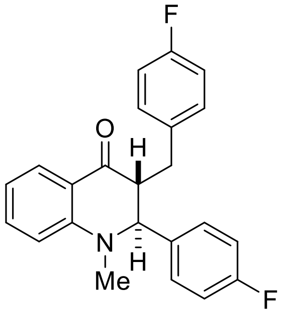

GP 7 starting from **1q**, 60 °C, 18 hours, CH/EA 40:1, yellow solid (81 mg, 0.223 mmol, 89%).

^{1}H-NMR (700 MHz, 298 K, $CDCl_3$): δ = 7.87 – 7.85 (m, 1H, H_{Ar}), 7.53 – 7.50 (m, 1H, H_{Ar}), 7.24 – 7.20 (m, 2H, H_{Ar}), 7.08 – 7.04 (m, 3H, H_{Ar}), 6.87 – 6.83 (m, 2H, H_{Ar}), 6.82 – 6.76 (m, 4H, H_{Ar}), 4.22 (bs, 1H, NC*H*), 3.04 – 3.01 (m, 4H, NCH3, COCHCH_2^A), 2.90 – 2.83 (m, 2H, COCH, COCHCH_2^B); **^{13}C-NMR** (176 MHz, 298 K, $CDCl_3$): δ = 194.6 (C_q), 162.2 (d, $^1J_{CF}$ = 245.2 Hz, C_q), 162.0 (d, $^1J_{CF}$ = 245.2 Hz, C_q), 136.6 (CH), 134.6 (d, $^4J_{CF}$ = 3.1 Hz, C_q), 134.2 (d, $^4J_{CF}$ = 3.2 Hz, C_q), 130.7 (d, $^3J_{CF}$ = 7.9 Hz, CH), 128.0 (d, $^3J_{CF}$ = 8.1 Hz, CH), 118.4 (C_q), 117.0 (CH), 115.9 (d, $^2J_{CF}$ = 21.5 Hz, CH), 115.8 (d, $^2J_{CF}$ = 21.3 Hz, CH), 112.2 (CH), 65.8 (CH, CO*C*H), 56.1 (CH, CO*C*H), 38.4 (CH_3, NCH_3), 36.3 (CH_2);**^{19}F-NMR** (282 MHz, 298 K, $CDCl_3$): δ = −114.8 – 114.9 (m, 1F), −115.8 – 115.9 (m, 1F); **^{15}N-NMR** (71 MHz, 298 K, $CDCl_3$): δ = 61.4; **HRMS** (ESI+, MeOH) *m/z* [M+Na]$^+$ ($C_{23}H_{19}NOFNa$) calc. 386.1332, found 386.1330.

2.2.3.21 *rac-trans*-2,3-3-cinnamyl-1-methyl-2-phenyl-2,3-dihydroquinolin-4(1H)-one (**2r**)

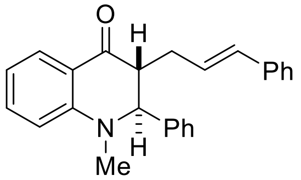

GP 7 starting from **1r**, room temperature, 30 minutes, CH/EA 25:1, yellow solid (76 mg, 0.215 mmol, 86%).

^{1}H-NMR (700 MHz, 298 K, $CDCl_3$): δ = 7.87 – 7.84 (m, 1H, H_{Ar}), 7.52 – 7.47 (m, 1H, H_{Ar}), 7.40 – 7.36 (m, 2H, H_{Ar}), 7.35 – 7.31 (m, 2H, H_{Ar}), 7.26 – 7.18 (m, 4H, H_{Ar}), 7.05 – 7.02 (m, 2H, H_{Ar}), 6.79 – 6.74 (m, 2H, H_{Ar}), 6.47 (d, $^3J_{HH}$ = 15.8 Hz, 1H, C*H*Ph), 6.27 (ddd, $^3J_{HH}$ = 15.4, 8.7, 6.5 Hz, 1H, C*H*CHPh), 4.55 (d, $^3J_{HH}$ = 2.9 Hz, 1H, NC*H*), 3.04 (s, 3H, NCH_3), 2.88 – 2.84 (m, 1H, COC*H*), 2.68 – 2.62 (m, 1H, COCHCH_2^A), 2.58 – 2.54 (m, 1H, COCHCH_2^B); **^{13}C-NMR** (176 MHz, 298 K, $CDCl_3$): δ = 194.9 (C_q), 150.5 (C_q), 139.4 (C_q), 137.2 (C_q), 136.3 (CH), 133.4 (CH, C*H*Ph), 129.0 (CH), 128.7 (CH), 128.4 (CH), 127.7 (CH), 127.6 (CH), 126.8 (CH, C*H*CHPh), 126.7 (CH), 126.4 (CH), 118.5 (C_q), 116.7 (CH), 112.3 (CH), 67.4 (CH, N*C*H), 53.8 (CH, CO*C*H), 38.4 (CH_3, NCH_3), 34.6 (CH_2); **^{15}N-NMR** (71 MHz, 298 K, $CDCl_3$): δ = 62.6; **HRMS** (ESI+, MeOH) *m/z* [M+Na]$^+$ ($C_{25}H_{23}NONa$) calc. 376.1677, found 376.1681.

2.2.3.22 *rac-trans*-2,3-1-methyl-2-phenyl-3-(3-phenylprop-2-yn-1-yl)-2,3-dihydroquinolin-4(1H)-one (**2s**)

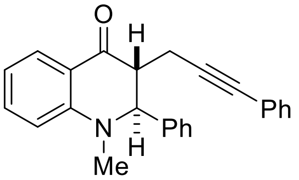

GP 7 starting from **1s**, 60°C, 18 hours, CH/EA 40:1, yellow solid (66 mg, 0.188 mmol, 75%).

^{1}H-NMR (700 MHz, 298 K, $CDCl_3$): δ = 7.88 – 7.85 (m, 1H, H_{Ar}), 7.51 – 7.48 (m, 1H, H_{Ar}), 7.46 – 7.43 (m, 2H, H_{Ar}), 7.33 – 7.30 (m, 3H, H_{Ar}), 7.29 – 7.26 (m, 2H, H_{Ar}), 7.25 – 7.23 (m, 1H, H_{Ar}), 7.15 – 7.13 (m, 2H, H_{Ar}), 6.80 – 6.78 (m, 1H, H_{Ar}), 6.77 – 6.74 (m, 1H, H_{Ar}), 4.93 (d, $^3J_{HH}$ = 3.9 Hz, 1H, NC*H*), 3.06 (s, 3H, NCH_3), 3.00 – 2.97 (m, 1H, COC*H*), 2.88 (dd, $^2J_{HH}$ = 16.7 Hz, $^3J_{HH}$ = 9.8 Hz, 1H, COCHCH_2^A), 2.65 (dd, $^2J_{HH}$ = 16.7 Hz, $^3J_{HH}$ = 4.6 Hz, 1H, COCHCH_2^B); **^{13}C-NMR** (176 MHz, 298 K, $CDCl_3$): δ = 193.5 (C_q), 150.8 (C_q), 139.2 (C_q), 136.5 (CH), 131.8 (CH), 129.1 (CH), 128.5 (CH), 128.4 (CH), 128.2 (CH), 127.9 (CH), 123.5 (C_q), 118.3 (C_q), 116.9 (CH), 112.6 (CH), 86.6 (C_q), 83.4 (C_q), 67.4 (CH, N*C*H), 52.9 (CH, CO*C*H), 38.5 (CH_3, NCH_3), 21.2 (CH_2, COCHCH_2); **HRMS** (ESI+, MeOH) *m/z* $[M+Na]^+$ ($C_{25}H_{21}NONa$) calc. 374.1521, found 374.1513.

2.2.3.23 rac-trans-2,3-7-fluoro-3-(4-methoxybenzyl)-1-methyl-2-phenyl-2,3-dihydroquinolin-4(1H)-one (**2t**)

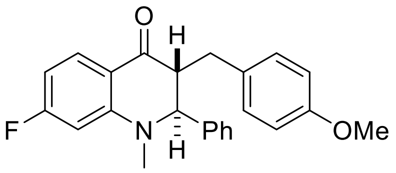

GP 1 starting from **1t**, room temperature, 30 minutes, CH/EA 10:1, yellow oil (56 mg, 0.149 µmol, 60%).

^{1}H-NMR (500 MHz, 303 K, $CDCl_3$): δ = 7.90-7.85 (m, 1H, H_{Ar}), 7.21-7.15 (m, 5H, H_{Ar}), 6.94-6.90 (m, 2H, H_{Ar}), 6.84-6.79 (m, 2H, H_{Ar}), 6.48-6.41 (m, 2H, H_{Ar}), 4.27 (d, $^3J_{HH}$ = 1.8 Hz, NC*H*Ph), 3.83 (s, 3H, OCH_3), 3.03-2.97 (m, 1H, COCHCH_2^A), 3.00 (s, 3H, NCH_3), 2.94-2.89 (ddd, $^3J_{HH}$ = 11.0, 4.8, 1.8 Hz, 1H, COC*H*CH$_2$), 2.82-2.75 (dd, $^2J_{HH}$ = 13.6 Hz, $^3J_{HH}$ = 11.2 Hz, 1H, COCHCH_2^B).

^{13}C-NMR (176 MHz, 298 K, $CDCl_3$): δ = 193.7 (C_q), 169.4-168.0 (d, $^1J_{CF}$ = 253.5 Hz, C_q), 158.7 (C_q), 152.3 (d, $^3J_{CF}$ = 12.2 Hz, C_q), 138.9 (C_q), 131.5 (d, $^3J_{CF}$ = 12.0 Hz, CH), 130.4 (CH), 129.1 (CH), 127.8 (CH), 126.3 (CH), 115.4 (Cq), 114.5 (CH), 104.8 (d, $^2J_{CF}$ = 23.0 Hz, CH), 98.6 (d, $^2J_{CF}$ = 26.4 Hz, CH), 66.3 (CH, N*C*HPh), 56.1 (CH, CO*C*HCH$_2$), 55.5 (CH_3, OCH_3), 38.7 (CH_3, NCH_3), 36.5 (CH_2, NCH_2).

HRMS (ESI+, MeCN) *m/z* $[M+H]^+$ ($C_{24}H_{23}NO_2F$) calc. 376.1713, found 376.1711.

2.2.3.24 rac-trans-2,3-6-fluoro-3-(4-methoxybenzyl)-1-methyl-2-phenyl-2,3-dihydroquinolin-4(1H)-one (**2u**)

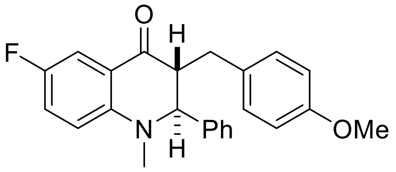

GP 1 starting from **1u**, room temperature, 30 minutes, CH/EA 10:1, yellow solid (56 mg, 0.149 µmol, 60%).

^{1}H-NMR (500 MHz, 303 K, $CDCl_3$): δ = 7.56-7.52 (m, 1H, H_{Ar}), 7.28-7.22 (m, 1H, H_{Ar}), 7.21-7.14 (m, 5H, H_{Ar}), 6.94-6.90 (m, 2H, H_{Ar}), 6.83-6.80 (m, 2H, H_{Ar}), 6.75-6.71 (m, 1H, H_{Ar}), 4.25 (d, $^3J_{HH}$ = 1.7 Hz, NC*H*Ph), 3.83 (s, 3H, OC*H*3), 3.00 (s, 3H, NC*H*3), 3.00-2.97 (dd, $^2J_{HH}$ = 12.0 Hz, $^3J_{HH}$ = 5.0 Hz, 1H, COCHCH_2^A), 2.97-2.93 (ddd, $^3J_{HH}$ = 13.7, 4.8, 2.0 Hz, 1H, COC*H*CH2), 2.84-2.78 (dd, $^2J_{HH}$ = 12.1 Hz, $^3J_{HH}$ = 13.7 Hz, 1H, COCHCH_2^B).

^{13}C-NMR (176 MHz, 298 K, $CDCl_3$): δ = 194.5 (d, $^4J_{CF}$ = 1.7 Hz, C_q), 158.7 (C_q), 155.8 (C_q), 154.4 (C_q), 147.1 (C_q), 138.9 (C_q), 130.4 (CH), 129.0 (CH), 127.6 (CH), 126.4 (CH), 123.9 (d, $^2J_{CF}$ = 23.8 Hz, CH), 118.5 (d, $^3J_{CF}$ = 5.6 Hz, C_q), 114.4 (CH), 113.5 (d, $^3J_{CF}$ = 11.8 Hz, CH), 113.4 (d, $^4J_{CF}$ = 3.8 Hz, CH), 66.2 (CH, N*C*HPh), 56.3 (CH, CO*C*HCH2), 55.5 (CH_3, O*C*H3), 38.8 (CH_3, N*C*H3), 36.3 (CH_2, N*C*H2)

HRMS (ESI+, MeCN) *m/z* $[M+Na]^+$ ($C_{24}H_{22}NO_2FNa$) calc. 398.1532, found 398.1532.

2.2.3.25 rac-trans-2,3-5-fluoro-3-(4-methoxybenzyl)-1-methyl-2-phenyl-2,3-dihydroquinolin-4(1H)-one (**2v**)

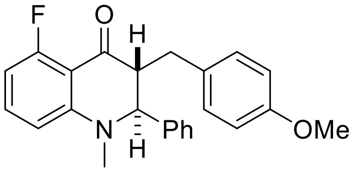

GP 2 starting from **1v**, room temperature, 30 minutes, CH/EA 10:1, orange solid (76 mg, 0.203 µmol, 81%).

^{1}H-NMR (500 MHz, 303 K, $CDCl_3$): δ = 7.44-7.37 (m, 1H, H_{Ar}), 7.20-7.14 (m, 5H, H_{Ar}), 6.93-6.89 (m, 2H, H_{Ar}), 6.86-6.81 (m, 2H, H_{Ar}), 6.57-6.52 (m, 1H, H_{Ar}), 6.45-6.39 (m, 1H, H_{Ar}), 4.26 (d, $^3J_{HH}$ = 1.8 Hz, NC*H*Ph), 3.83 (s, 3H, OC*H*3), 3.07-3.02 (m, 1H, COCHCH_2^A), 3.05 (s, 3H, NC*H*3), 2.89-2.84 (ddd, $^3J_{HH}$ = 11.0, 4.6, 1.8 Hz, 1H, COC*H*CH2), 2.82-2.75 (dd, $^2J_{HH}$ = 13.3 Hz, $^3J_{HH}$ = 11.0 Hz, 1H, COCHCH_2^B).

^{13}C-NMR (176 MHz, 298 K, $CDCl_3$): δ = 193.3 (C_q), 164.3-162.8 (d, $^1J_{CF}$ = 263.0 Hz, C_q), 158.7 (C_q), 151.6 (d, $^3J_{CF}$ = 4.0 Hz, C_q), 138.7 (C_q), 136.6 (d, $^3J_{CF}$ = 12.6 Hz, CH), 130.4 (CH), 130.2 (C_q), 129.1 (CH), 127.8 (CH), 126.2 (CH), 114.5 (CH), 107.7 (d, $^4J_{CF}$ = 3.3 Hz, CH), 104.1 (d, $^2J_{CF}$ = 22.0 Hz, CH), 66.2 (CH, N*C*HPh), 57.0 (CH, CO*C*HCH2),55.5(CH_3, O*C*H3), 39.4 (CH_3, N*C*H3), 36.4(CH_2, N*C*H2).

HRMS (ESI+, MeCN) *m/z* $[M+Na]^+$ ($C_{24}H_{22}NO_2FNa$) calc. 398.1532, found 398.1533.

2.2.3.26 rac-trans-2,3-3-(4-methoxybenzyl)-1-methyl-2-phenyl-5-(trifluoromethyl)-2,3-dihydroquinolin-4(1H)-one (**2w**)

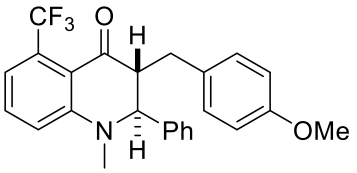

GP 1 starting from **1w**, room temperature, overnight, CH/EA 10:1, orange oil (22 mg, 0.0527 µmol, 21%).

^{1}H-NMR (700 MHz, 298 K, $CDCl_3$): δ = 7.53-7.49 (m, 1H, H_{Ar}), 7.22-7.16 (m, 3H, H_{Ar}), 7.16-7.11 (m, 3H, H_{Ar}), 7.04-6.99 (m, 1H, H_{Ar}), 6.91-6.88 (m, 2H, H_{Ar}), 6.86-6.82 (m, 2H, H_{Ar}), 4.34 (d, $^3J_{HH}$ = 2.1 Hz, 1H, NC*H*Ph), 3.82 (s, 3H, OCH_3), 3.05 (s, 3H, NCH_3), 3.05-3.02 (dd, $^2J_{HH}$ = 13.7 Hz, $^3J_{HH}$ = 5.4 Hz, 1H, COCHCH_2^A), 2.91 (ddd, $^3J_{HH}$ = 10.5, 5.3, 2.1 Hz, 1H, COC*H*CH_2), 2.77 (dd, $^2J_{HH}$ = 13.7 Hz, $^3J_{HH}$ = 10.5 Hz, 1H, COCHCH_2^B).

^{13}C-NMR (176 MHz, 298 K, $CDCl_3$): δ = 193.8 (C_q), 158.7 (C_q), 151.4 (C_q), 138.9 (C_q), 134.5 (CH), 130.7-130.1 (q, $^2J_{CF}$ =32.2 Hz, C_q), 130.3 (CH), 129.9 (CH), 129.2 (CH), 128.0 (CH), 126.1-121.4 (q, $^1J_{CF}$ = 274.1 Hz, C_q), 126.0 (CH), 116.5 (CH), 116.0 (q, $^3J_{CF}$ = 7.3 Hz, CH), 115.1 (C_q), 114.4 (CH), 66.8 (CH, N*C*HPh), 56.9 (CH, CO*C*HCH_2), 55.5 (CH_3, OCH_3), 39.3 (CH_3, NCH_3), 36.1 (CH_2, NCH_2).

HRMS (ESI+, MeCN) *m/z* $[M+Na]^+$ ($C_{25}H_{22}NO_2F_3Na$) calc. 448.1500, found 448.1506.

2.2.3.27 rac-trans-2,3-3-(4-methoxybenzyl)-1-methyl-2-phenyl-6-(trifluoromethyl)-2,3-dihydroquinolin-4(1H)-one (**2x**)

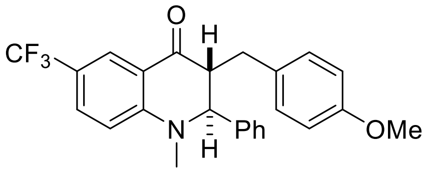

GP 1 starting from **1x**, room temperature, 1 hour, CH/EA 10:1, yellow solid (95 mg, 0.224 µmol, 90%).

^{1}H-NMR (500 MHz, 303 K, $CDCl_3$): δ = 8.14-8.11 (m, 1H, H_{Ar}), 7.71-7.66 (m, 1H, H_{Ar}), 7.20-7.15 (m, 5H, H_{Ar}), 6.95-6.90 (m, 2H, H_{Ar}), 6.89-6.79 (m, 3H, H_{Ar}), 4.32 (s, 1H, NC*H*Ph), 3.83 (s, 3H, OCH_3), 3.08 (s, 3H, NCH_3), 3.04-2.99 (dd, $^2J_{HH}$ = 12.7 Hz, $^3J_{HH}$ = 5.0 Hz, 1H, COCHCH_2^A), 2.99-2.96 (ddd, $^3J_{HH}$ = 10.4, 5.0, 1.8 Hz, 1H, COC*H*CH_2), 2.81-2.73 (dd, $^2J_{HH}$ = 12.6 Hz, $^3J_{HH}$ = 10.6 Hz, 1H, COCHCH_2^B).

^{13}C-NMR (176 MHz, 298 K, $CDCl_3$): δ = 194.2 (C_q), 158.8 (C_q), 151.9 (C_q), 138.4 (C_q), 132.6 (q, $^3J_{CF}$ = 3.2 Hz, CH), 130.3 (CH), 130.0 (C_q), 129.2 (CH), 128.0 (CH), 127.0-122.4 (q, $^1J_{CF}$ = 270.5 Hz, C_q), 126.3 (q, $^3J_{CF}$ = 4.0 Hz, CH), 126.1 (CH), 119.0-118.5 (q, $^2J_{CF}$ = 33.7 Hz, C_q), 117.6 (CH), 114.5 (CH), 112.4 (CH), 66.2 (CH, N*C*HPh), 56.0 (CH, CO*C*HCH_2), 55.5 (CH_3, O*C*H3), 38.8 (CH_3, NCH_3), 36.5 (CH_2, NCH_2).

HRMS (ESI+, MeCN) *m/z* $[M+Na]^+$ ($C_{25}H_{22}NO_2F_3Na$) calc. 448.1500, found 448.1505.

2.2.3.28 rac-trans-2,3-6-bromo-3-(4-methoxybenzyl)-1-methyl-2-phenyl-2,3-dihydroquinolin-4(1H)-one (**2y**)

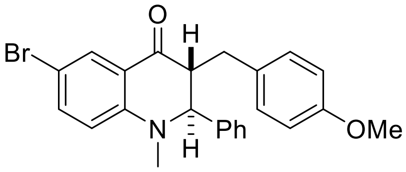

GP 1 starting from **1y**, room temperature, 30 minutes, without further purification, yellow solid (107 mg, 0.245 µmol, 98%).

^{1}H-NMR (700 MHz, 298 K, $CDCl_3$): δ = 7.94-7.92 (m, 1H, H_{Ar}), 7.57-7.54 (m, 1H, H_{Ar}), 7.20-7.15 (m, 5H, H_{Ar}), 6.93-6.90 (m, 2H, H_{Ar}), 6.81-6.78 (m, 2H, H_{Ar}), 6.70-6.67 (m, 1H, H_{Ar}), 4.27 (d, $^3J_{HH}$ = 1.8 Hz, 1H, NC*H*Ph), 3.83 (s, 3H, OCH_3), 3.02 (s, 3H, NCH_3), 2.99-2.95 (dd, $^2J_{HH}$ = 13.2 Hz, $^3J_{HH}$ = 5.1 Hz, 1H, COCHCH_2^A), 2.94-2.90 (ddd, $^3J_{HH}$ = 10.9, 5.2, 1.8 Hz, 1H, COC*H*CH_2), 2.80-2.76 (dd, $^2J_{HH}$ = 13.2 Hz, $^3J_{HH}$ = 10.9 Hz, 1H, COCHCH_2^B).

^{13}C-NMR (176 MHz, 298 K, $CDCl_3$): δ = 194.3 (C_q), 158.8 (C_q), 149.2 (C_q), 138.9 (CH), 138.5 (C_q), 130.8 (CH), 130.3 (CH), 130.1 (C_q), 129.1 (CH), 127.8 (CH), 126.2 (CH), 119.5 (C_q), 114.5 (CH), 114.2 (CH), 109.2 (C_q), 66.2 (CH, N*C*HPh), 56.1 (CH, CO*C*HCH_2), 55.5 (CH_3, O*C*H3), 38.6 (CH_3, NCH_3), 36.5 (CH_2, NCH_2).

HRMS (ESI+, MeCN) exact mass for $[M+Na]^+$ ($C_{24}H_{22}NO_2BrNa$) calc. m/z 458.0732, found 458.0732.

2.2.3.29 rac-trans-2,3-3-(4-methoxybenzyl)-1,7-dimethyl-2-phenyl-2,3-dihydroquinolin-4(1H)-one (**2z**)

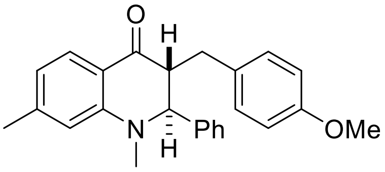

GP 1 starting from **1z**, room temperature, 50 minutes, without further purification, yellow solid (91 mg, 0.245 µmol, 98%).

^{1}H-NMR (700 MHz, 298 K, $CDCl_3$): δ = 7.77-7.74 (m, 1H, H_{Ar}), 7.21-7.18 (m, 2H, H_{Ar}), 7.16-7.11 (m, 3H, H_{Ar}), 6.94-6.89 (m, 2H, H_{Ar}), 6.83-6.80 (m, 2H, H_{Ar}), 6.60-6.56 (m, 2H, H_{Ar}), 4.24 (d, $^3J_{HH}$ = 1.8 Hz, 1H, NC*H*Ph), 3.83 (s, 3H, OCH_3), 3.03 (s, 3H, NCH_3), 3.01-2.97 (dd, $^2J_{HH}$ = 13.4 Hz, $^3J_{HH}$ = 4.7 Hz, 1H, COCHCH_2^A), 2.90-2.87 (ddd, $^3J_{HH}$ = 11.3, 4.8, 1.8 Hz, 1H, COC*H*CH_2), 2.82-2.77 (dd, $^2J_{HH}$ = 13.4 Hz, $^3J_{HH}$ = 11.3 Hz, 1H, COCHCH_2^B), 2.41 (s, 3H, CCH_3).

^{13}C-NMR (176 MHz, 298 K, $CDCl_3$): δ = 194.8 (C_q), 158.6 (C_q), 154.4 (C_q), 147.5 (C_q), 139.3 (C_q), 130.7 (C_q), 130.3 (CH), 128.9 (CH), 128.6 (CH), 127.5 (CH), 126.4 (CH), 118.2 (CH), 116.3 (C_q), 114.4 (CH), 112.3 (CH), 66.1 (CH, N*C*HPh), 56.3 (CH,

CO*C*HCH$_2$), 55.5 (CH$_3$, O*C*H3), 38.6 (CH$_3$, N*C*H$_3$), 36.5 (CH$_2$, N*C*H$_2$), 22.7 (CH$_3$, C*C*H$_3$).

HRMS (ESI+, MeCN) exact mass for $[M+Na]^+$ ($C_{25}H_{25}NO_2Na$) calc. m/z 394.1783, found 394.1782.

2.2.3.30 rac-trans-2,3-3-(4-methoxybenzyl)-1-methyl-6-nitro-2-phenyl-2,3-dihydroquinolin-4(1H)-one (**2aa**)

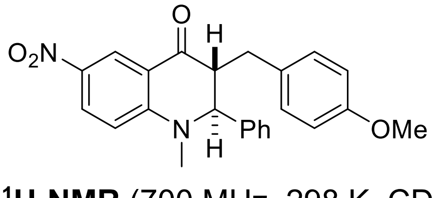

GP 1 starting from **1aa**, room temperature, 30 minutes, without further purification, yellow solid (98 mg, 0.243 µmol, 97%).

^{1}H-NMR (700 MHz, 298 K, CDCl$_3$): δ = 8.76-8.74 (m, 1H, H$_{Ar}$), 8.35-8.33 (m, 1H, H$_{Ar}$), 7.21-7.19 (m, 3H, H$_{Ar}$), 7.17-7.14 (m, 2H, H$_{Ar}$), 6.94-6.91 (m, 2H, H$_{Ar}$), 6.85-6.83 (m, 1H, H$_{Ar}$), 6.82-6.78 (m, 2H, H$_{Ar}$), 4.40 (s, 1H, NC*H*Ph), 3.83 (s, 3H, OC*H*$_3$), 3.16 (s, 3H, NC*H*$_3$), 3.05-3.03 (ddd, $^3J_{HH}$ = 11.6, 5.4, 1.8 Hz, 1H, COC*H*CH$_2$), 3.03-2.99 (dd, $^2J_{HH}$ = 14.4 Hz, $^3J_{HH}$ = 5.4 Hz, 1H, COCHC*H$_2$*^{A}), 2.77-2.74 (dd, $^2J_{HH}$ = 14.0 Hz, $^3J_{HH}$ = 11.9 Hz, 1H, COCHC*H$_2$*^{B}).

^{13}C-NMR (176 MHz, 298 K, CDCl$_3$): δ = 193.4 (C$_q$), 158.9 (C$_q$), 153.6 (C$_q$), 138.3 (C$_q$), 137.7 (C$_q$), 131.1 (CH), 130.3 (CH), 129.4 (CH), 129.3 (C$_q$), 128.4 (CH), 125.8 (CH), 125.5 (CH), 117.1 (C$_q$), 114.6 (CH), 112.3 (CH), 66.7 (CH, N*C*HPh), 55.9 (CH, CO*C*HCH$_2$), 55.5 (CH$_3$, O*C*H3), 39.2 (CH$_3$, N*C*H$_3$), 36.6 (CH$_2$, N*C*H$_2$).

HRMS (ESI+, MeCN) exact mass for $[M+Na]^+$ ($C_{24}H_{22}N_2O_4Na$) calc. m/z 425.1477, found 425.1477.

2.2.3.31 rac-trans-2,3-7-methoxy-3-(4-methoxybenzyl)-1-methyl-2-phenyl-2,3-dihydroquinolin-4(1H)-one (**2ab**)

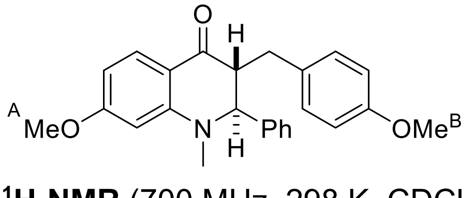

GP 1 starting from **1ab**, room temperature, 30 minutes, without further purification, yellow solid (93 mg, 0.240 µmol, 96%).

^{1}H-NMR (700 MHz, 298 K, CDCl$_3$): δ = 7.83-7.80 (m, 1H, H$_{Ar}$), 7.21-7.17 (m, 2H, H$_{Ar}$), 7.17-7.12 (m, 3H, H$_{Ar}$), 6.93-6.89 (m, 2H, H$_{Ar}$), 6.83-6.79 (m, 2H, H$_{Ar}$), 6.37-6.33 (m, 1H, H$_{Ar}$), 6.20-6.18 (m, 1H, H$_{Ar}$), 4.24 (d, $^3J_{HH}$ = 1.5 Hz, 1H, NC*H*Ph), 3.91 (s, 3H, OC*H$_3$*^{A}), 3.83 (s, 3H, OC*H$_3$*^{B}), 3.02 (s, 3H, NC*H*$_3$), 3.01-2.98 (dd, $^2J_{HH}$ = 13.2 Hz, $^3J_{HH}$ = 4.7 Hz, 1H, COCHC*H$_2$*^{A}), 2.88-2.84 (ddd, $^3J_{HH}$ = 11.2, 4.6, 1.6 ‘Hz, 1H, COC*H*CH$_2$), 2.82-2.76 (dd, $^2J_{HH}$ = 13.1 Hz, $^3J_{HH}$ = 11.3 Hz, 1H, COCHC*H$_2$*^{B}).

^{13}C-NMR (176 MHz, 298 K, $CDCl_3$): δ = 193.9 (C_q), 166.7 (C_q), 158.6 (C_q), 152.3 (C_q), 139.3 (C_q), 131.0 (CH), 130.7 (C_q), 130.4 (CH), 128.9 (CH), 127.5 (CH), 126.3 (CH), 114.4 (CH), 112.9 (C_q), 104.1 (CH), 96.2 (CH), 66.3 (CH, N*C*HPh), 56.1 (CH, CO*C*HCH$_2$), 55.6 (CH_3, O*C*H$_3^A$), 55.5 (CH_3, O*C*H$_3^B$), 38.7 (CH_3, N*C*H$_3$), 36.7 (CH_2, N*C*H$_2$).

HRMS (EI) exact mass for $[M]^+$ ($C_{25}H_{25}NO_3$) calc m/z 387.1834, found 387.1850

2.2.4 Representative procedure for 1 mmol scale

Substrate **1b** (1.00 mmol, 358 mg, 1.00 equiv.) and $B(C_6F_5)_3$ (51 mg, 100 µmol, 0.100 equiv.) were dissolved in $CHCl_3$ (0.1 M) and placed in a crimp seal glass vial. The reaction mixture was stirred at room temperature for 18h. The resulting mixture was diluted with 40 ml DCM, washed with 40 ml H_2O, and extracted twice with 40 ml DCM. The combined organic layers were dried over Na_2SO_4, and volatiles were removed under reduced pressure. Subsequent flash chromatography (CH/EA 40:1, SiO_2) yielded **2b** yellow solid (326 mg, 0.912 mmol, 91%).

2.3 References

[SI1] a) G. M. Sheldrick, SHELXT - integrated space-group and crystal-structure determination, *Acta Crystallogr. A* **2015**, *71*, 3; b) G. M. Sheldrick, Crystal structure refinement with SHELXL, *Acta Crystallogr. C* **2015**, *71*, 3.

[SI2] K. L. Walker, L. M. Dornan, R. N. Zare, R. M. Waymouth, M. J. Muldoon, Mechanism of Catalytic Oxidation of Styrenes with Hydrogen Peroxide in the Presence of Cationic Palladium(II) Complexes, *Journal of the American Chemical Society* **2017**, *139*, 12495.

[SI3] J. Holz, C. Pfeffer, H. Zuo, D. Beierlein, G. Richter, E. Klemm, R. Peters, In Situ Generated Gold Nanoparticles on Active Carbon as Reusable Highly Efficient Catalysts for a C sp3 -C sp3 Stille Coupling, *Angew. Chem. Int. Ed. Engl.* **2019**, *58*, 10330.

[SI4] C. Li, Y. Zhang, Q. Sun, T. Gu, H. Peng, W. Tang, Transition-Metal-Free Stereospecific Cross-Coupling with Alkenylboronic Acids as Nucleophiles, *Journal of the American Chemical Society* **2016**, *138*, 10774.

[SI5] K. J. Emery, T. Tuttle, A. R. Kennedy, J. A. Murphy, C–C bond-forming reactions of ground-state aryl halides under reductive activation, *Tetrahedron* **2016**, *72*, 7875.

[SI6] J. F. Guastavino, R. A. Rossi, Synthesis of benzo-fused heterocycles by intramolecular α-arylation of ketone enolate anions, *J. Org. Chem.* **2012**, *77*, 460.

[SI7] W. Verboom, M. Hamzink, D. N. Reinhoudt, R. Visser, Novel applications of the "t-amino effect" in heterocyclic chemistry. Synthesis of a pyrrolo[1,2-a]quinazoline and 5H-pyrrolo[1,2-a][3,1]benzothiazines, *Tetrahedron Lett.* **1984**, *25*, 4309.

[SI8] J. Zhou, L. Li, S. Wang, M. Yan, W. Wei, Catalyst-free photodecarbonylation of ortho -amino benzaldehyde, *Green Chem.* **2020**, *22*, 3421.

[SI9] R. A. Bunce, T. Nago, S. Abuskhuna, Steric and Electronic Requirements in the Synthesis of 2,3-Dihydro-4(1 H)-quinolinones by the Tandem Michael-S N Ar Reaction, *J. Heterocyclic Chem.* **2015**, *52*, 1143.

[SI10] M.-Y. Chang, C.-Y. Tsai, M.-H. Wu, *Tetrahedron* **2013**, *69*, 6364

3 NMR Spectra

3.1 NMR spectra of precursors

^{1}H-NMR (700 MHz, 298 K, $CDCl_3$) (*rac*-**S2a**)

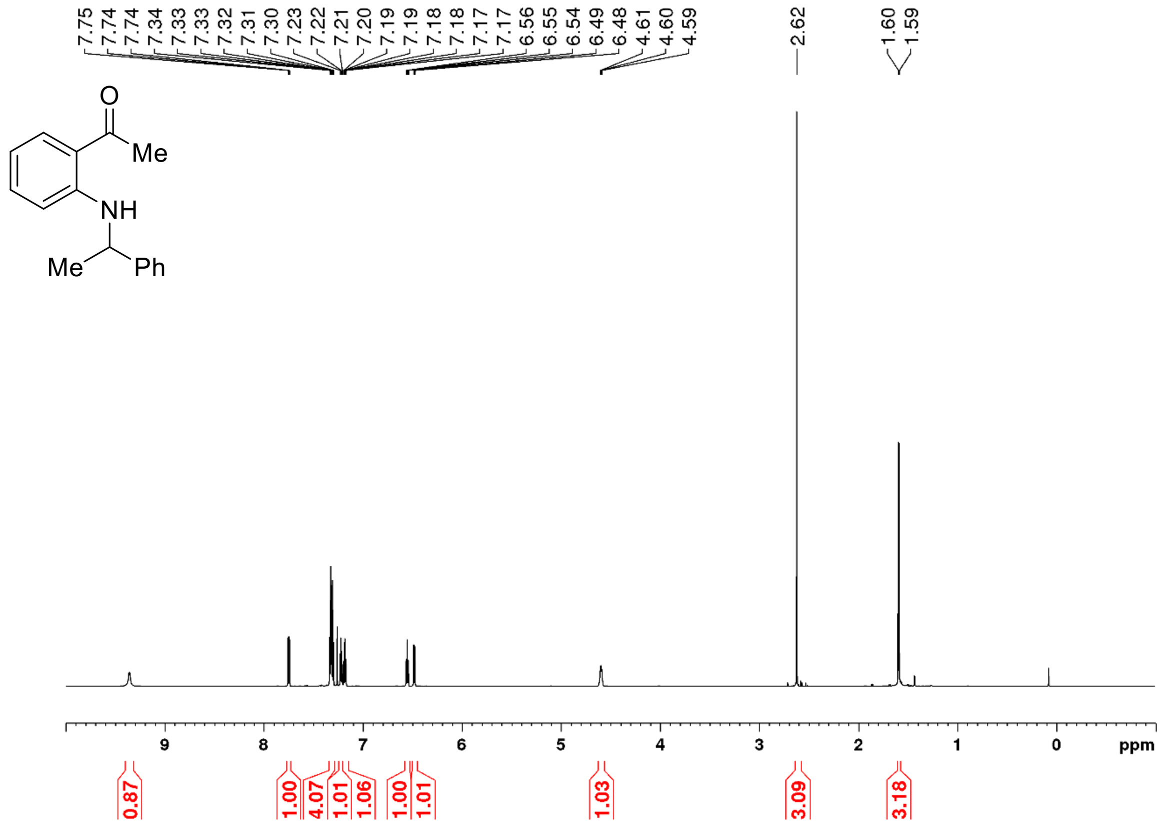

^{13}C-NMR (176 MHz, 298 K, $CDCl_3$)

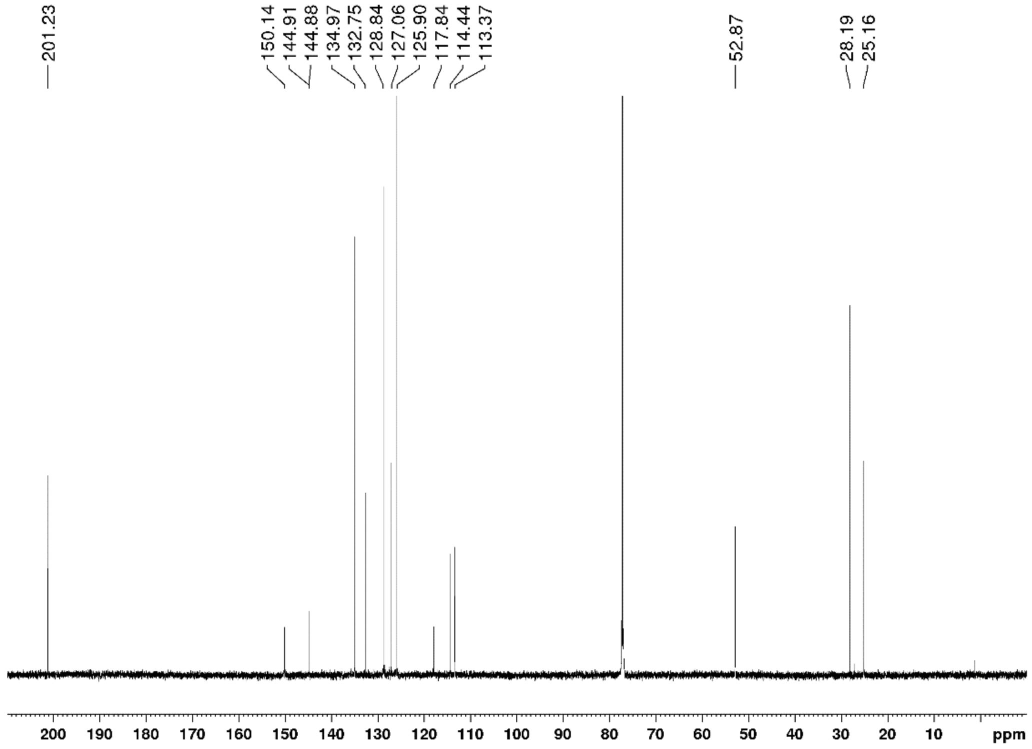

^{1}H-NMR (500 MHz, 303 K, $CDCl_3$) (d_1-*rac*-**S2a**)

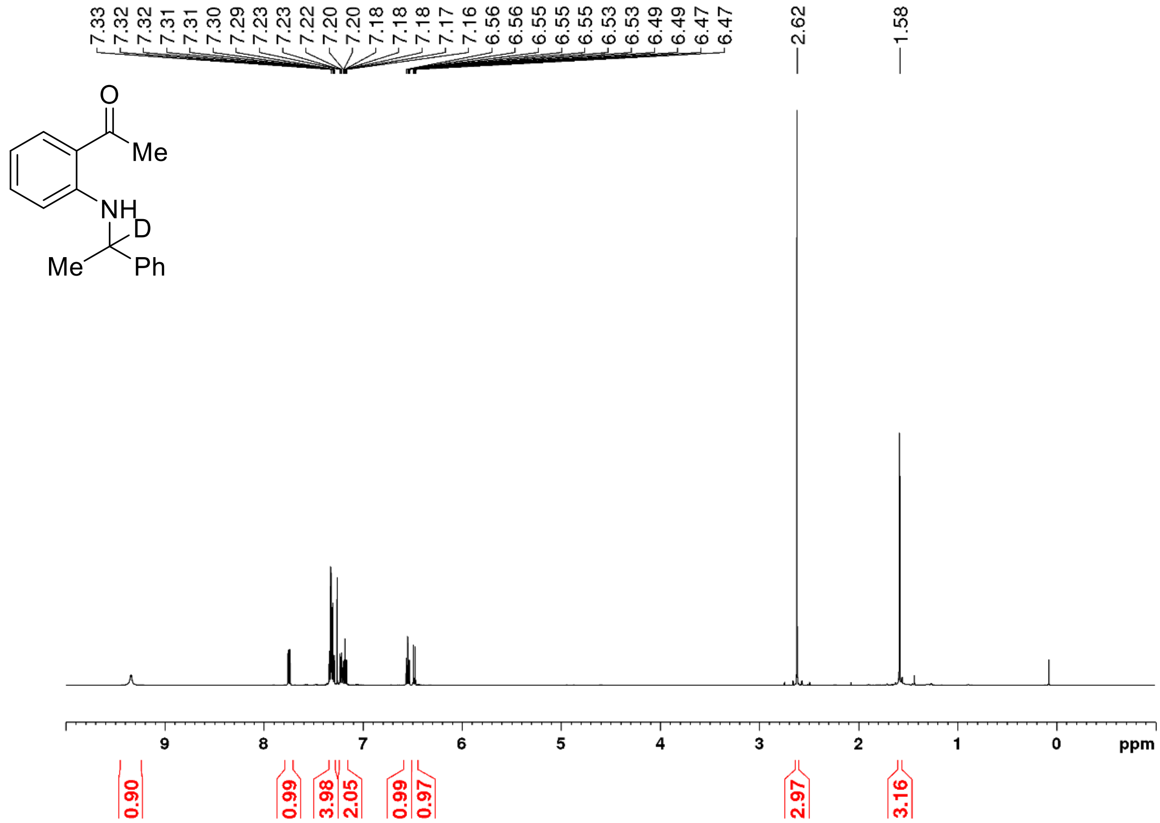

^{1}H-NMR (700 MHz, 298 K, $CDCl_3$) (*rac*-**S2b**)

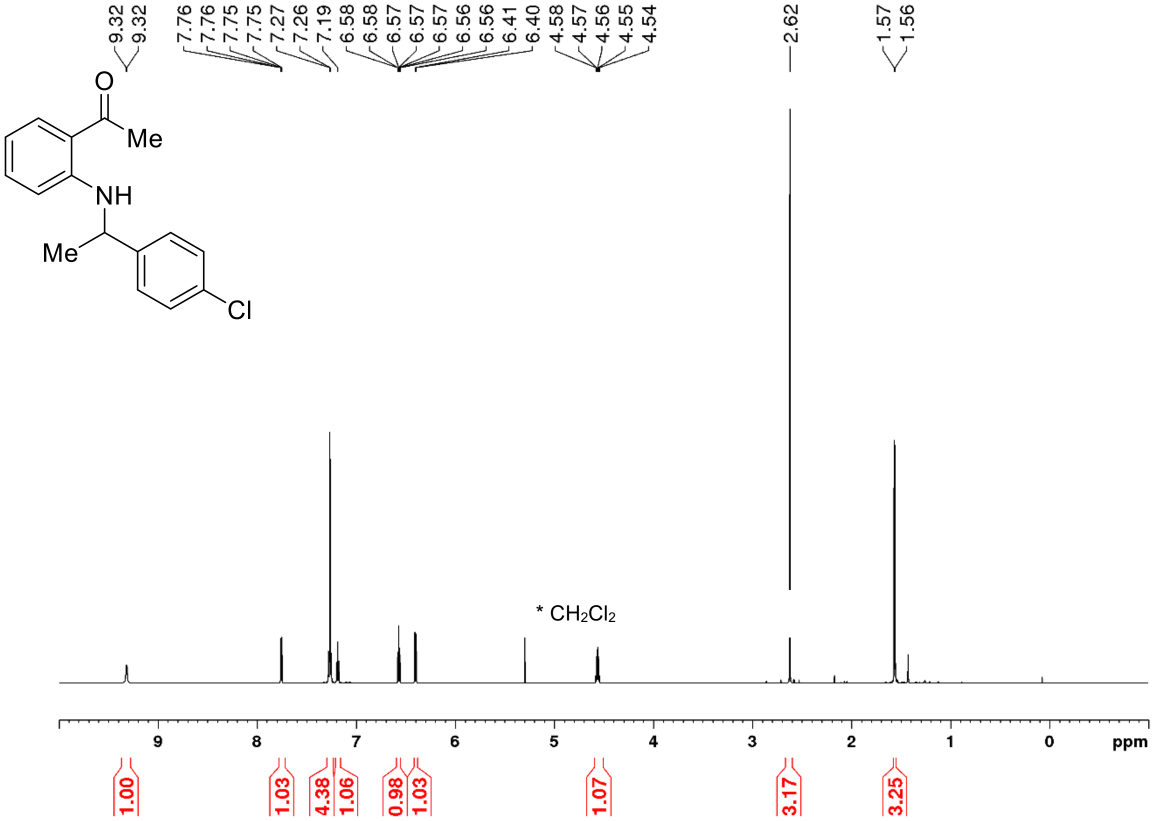

^{13}C-NMR (176 MHz, 298 K, $CDCl_3$)

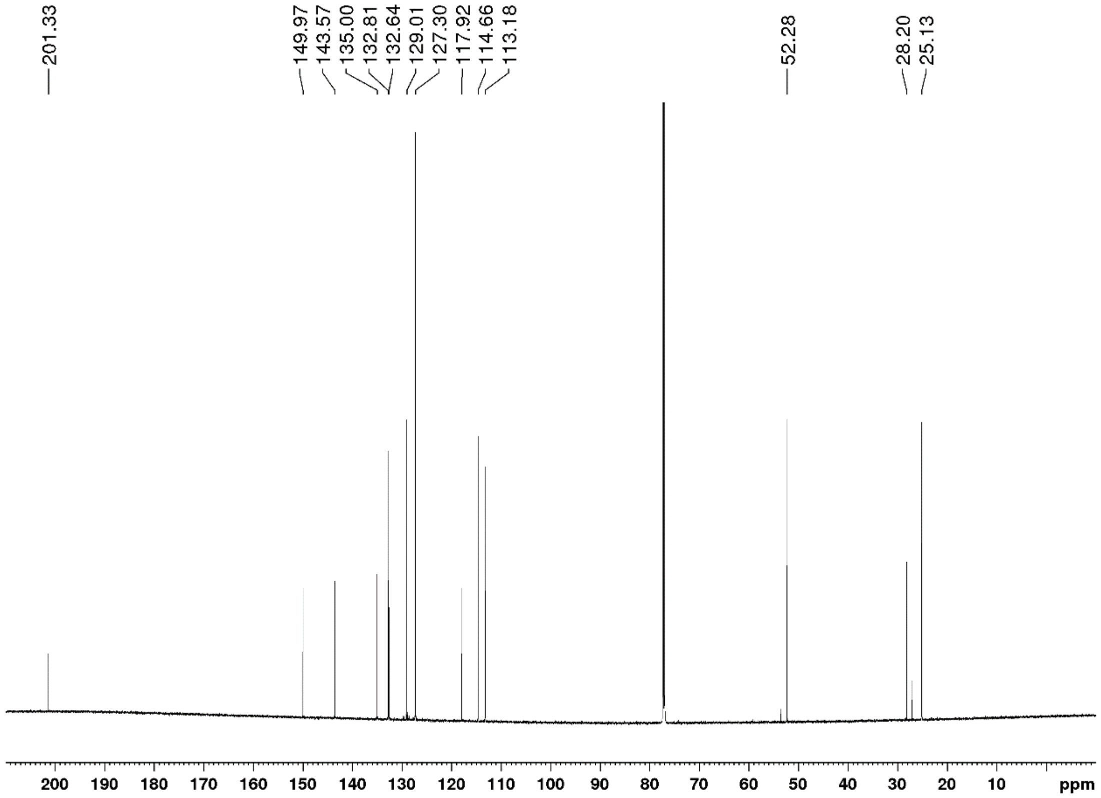

^{1}H-NMR (700 MHz, 298 K, $CDCl_3$) (*rac*-**S2c**)

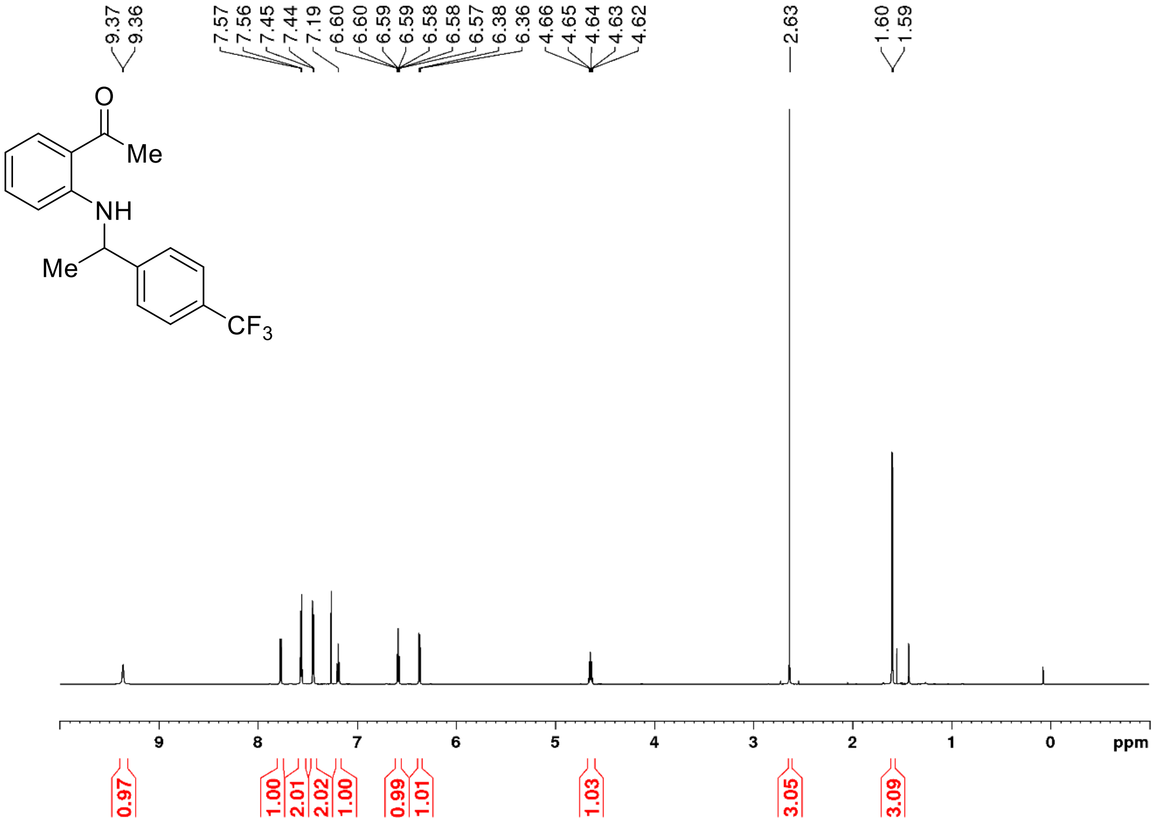

^{13}C-NMR (176 MHz, 298 K, $CDCl_3$)

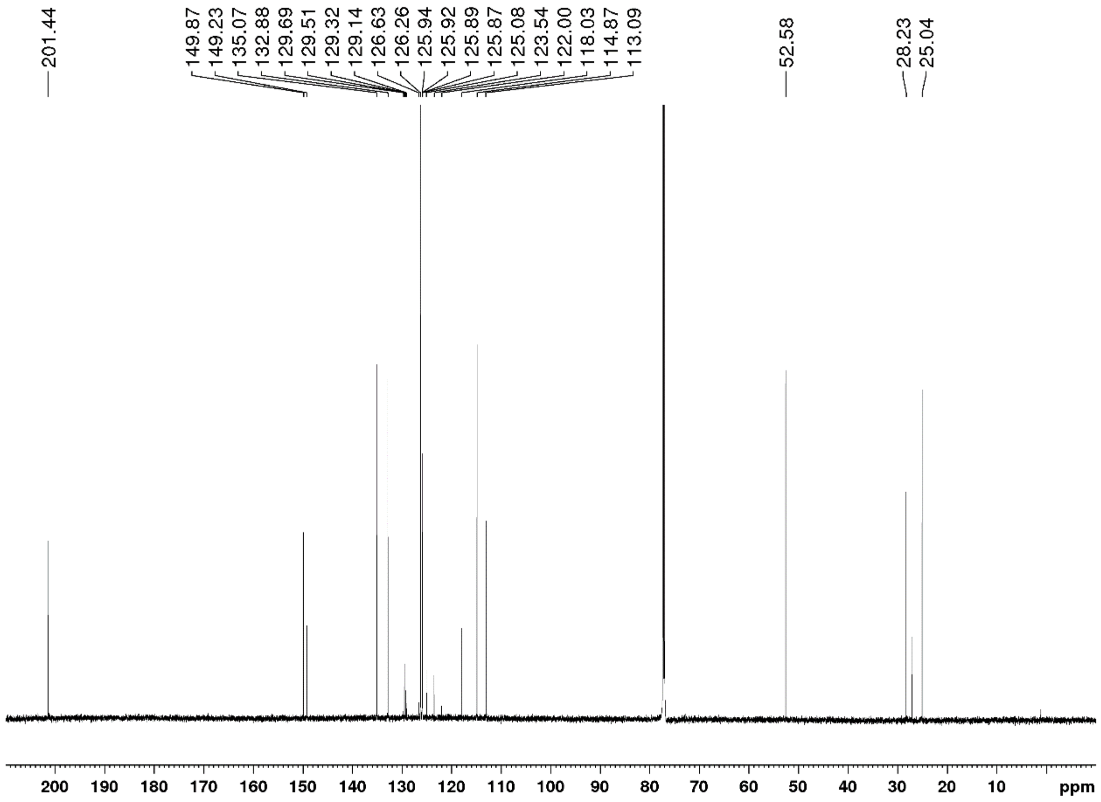

^{1}H-NMR (700 MHz, 298 K, $CDCl_3$) ((*S*)-**2a**)

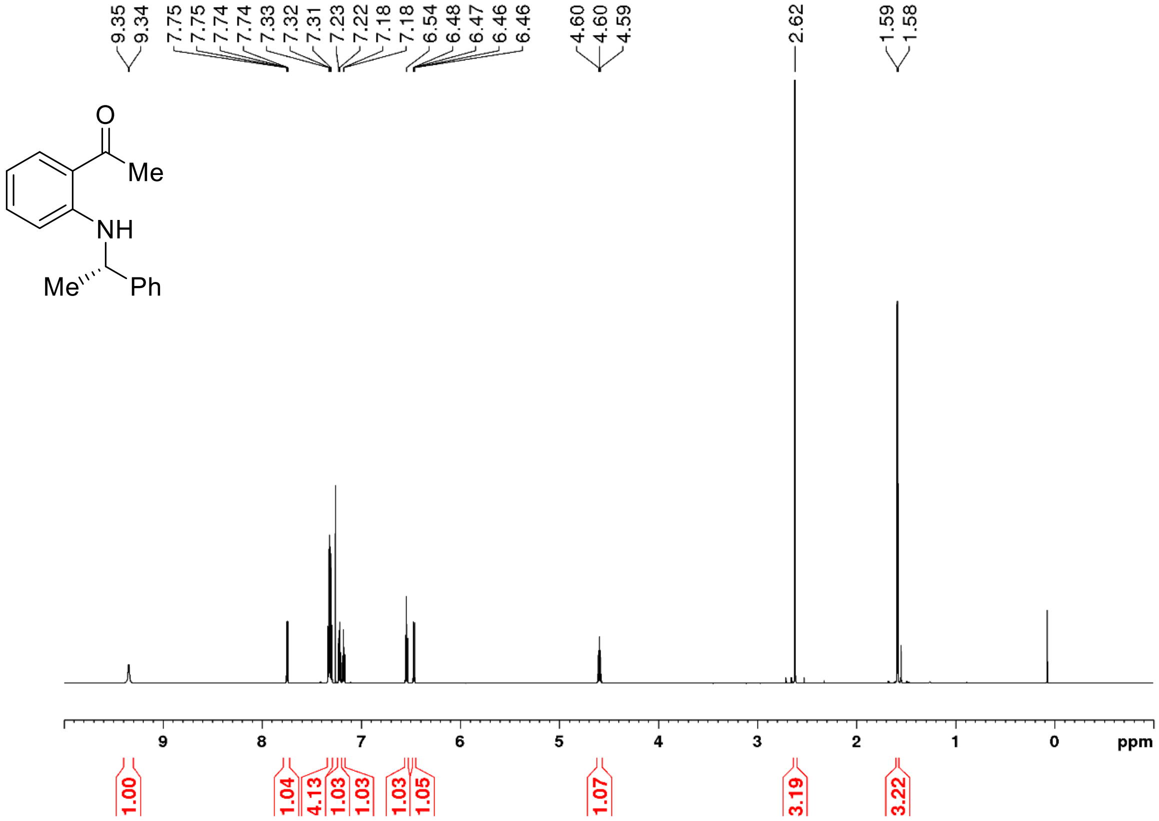

^{13}C-NMR (176 MHz, 298 K, $CDCl_3$)

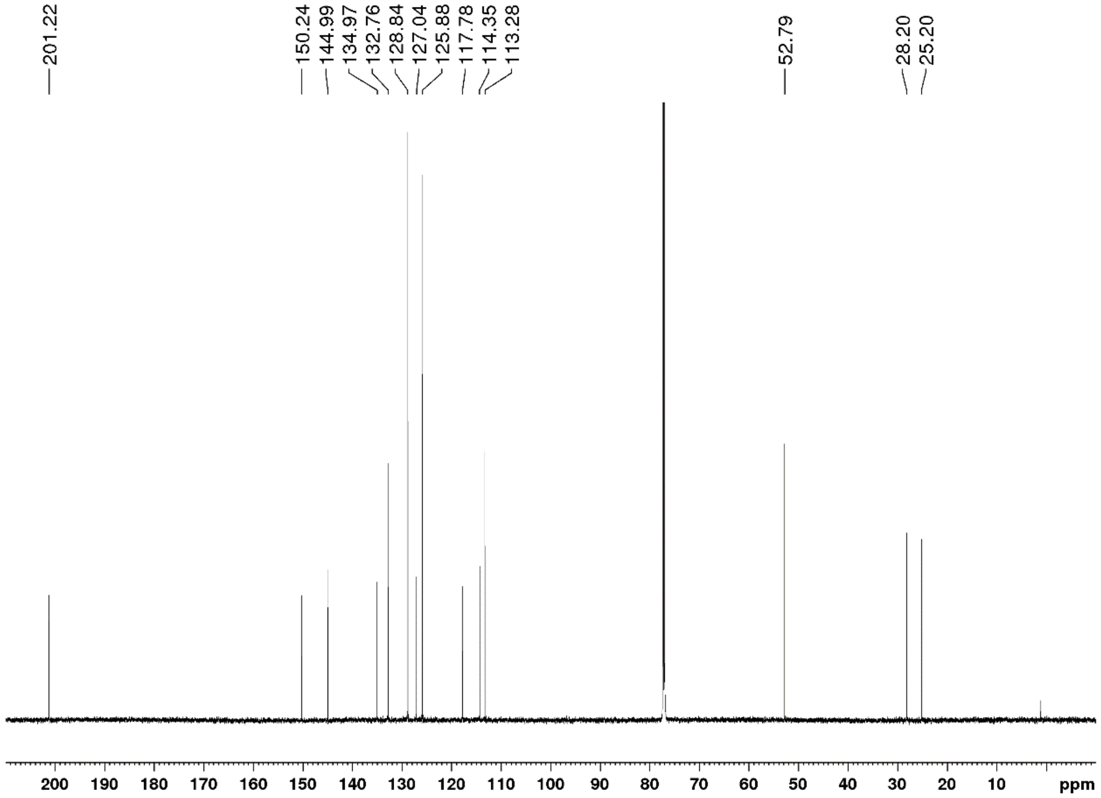

^{1}**H-NMR** (700 MHz, 298 K, $CDCl_3$) (*rac*-**S3a**)

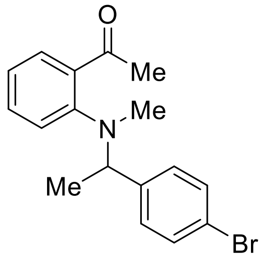

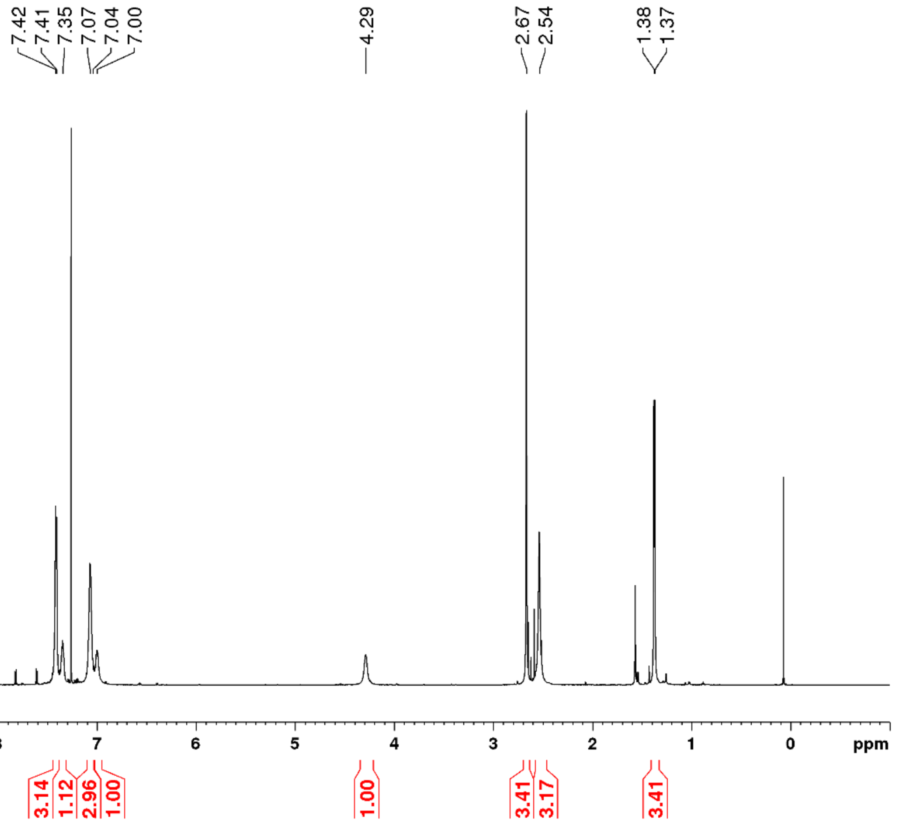

^{13}**C-NMR** (176 MHz, 298 K, $CDCl_3$)

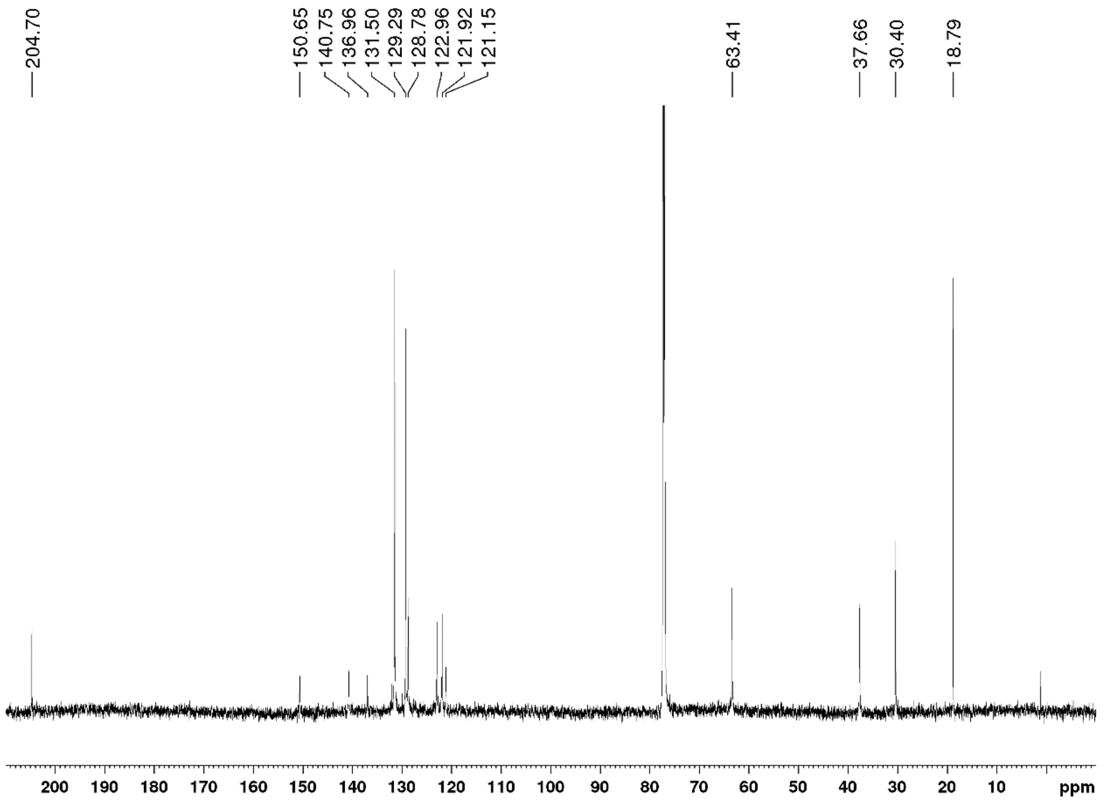

^{1}H-NMR (700 MHz, 298 K, $CDCl_3$) ((*S*)-**S3a**)

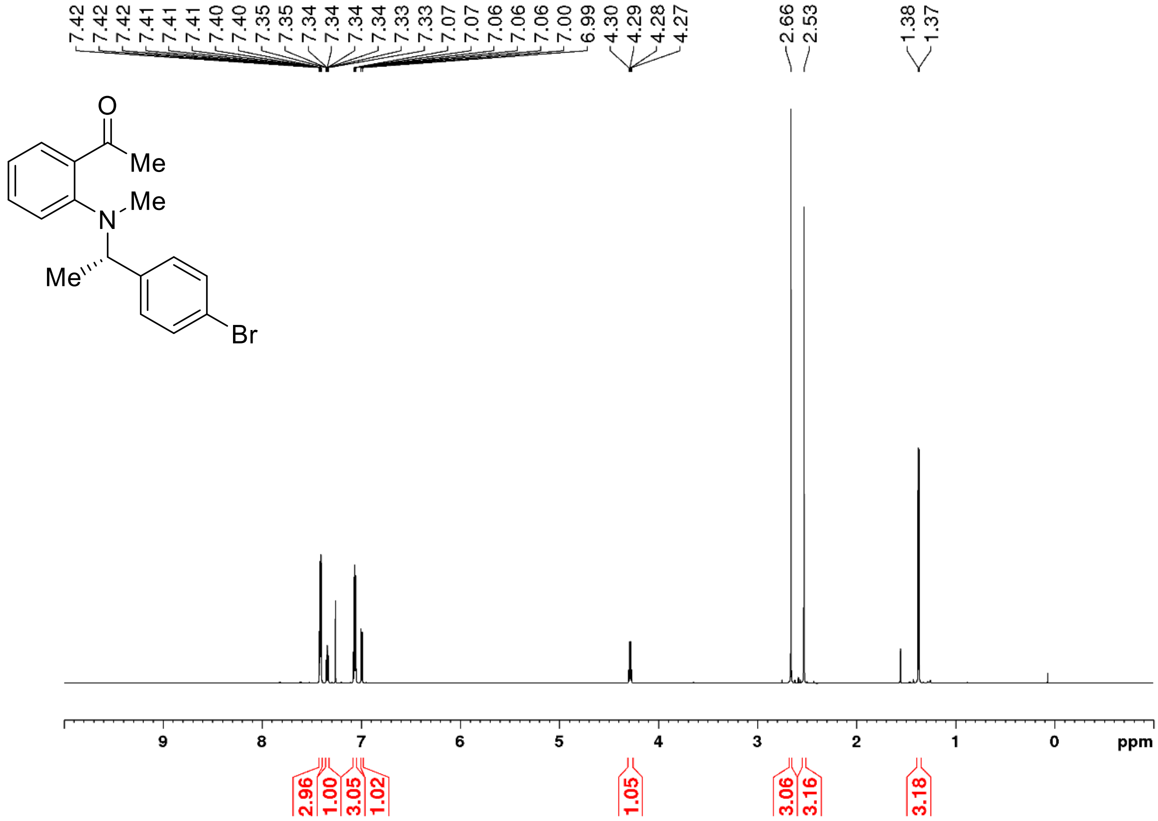

^{13}C-NMR (176 MHz, 298 K, $CDCl_3$)

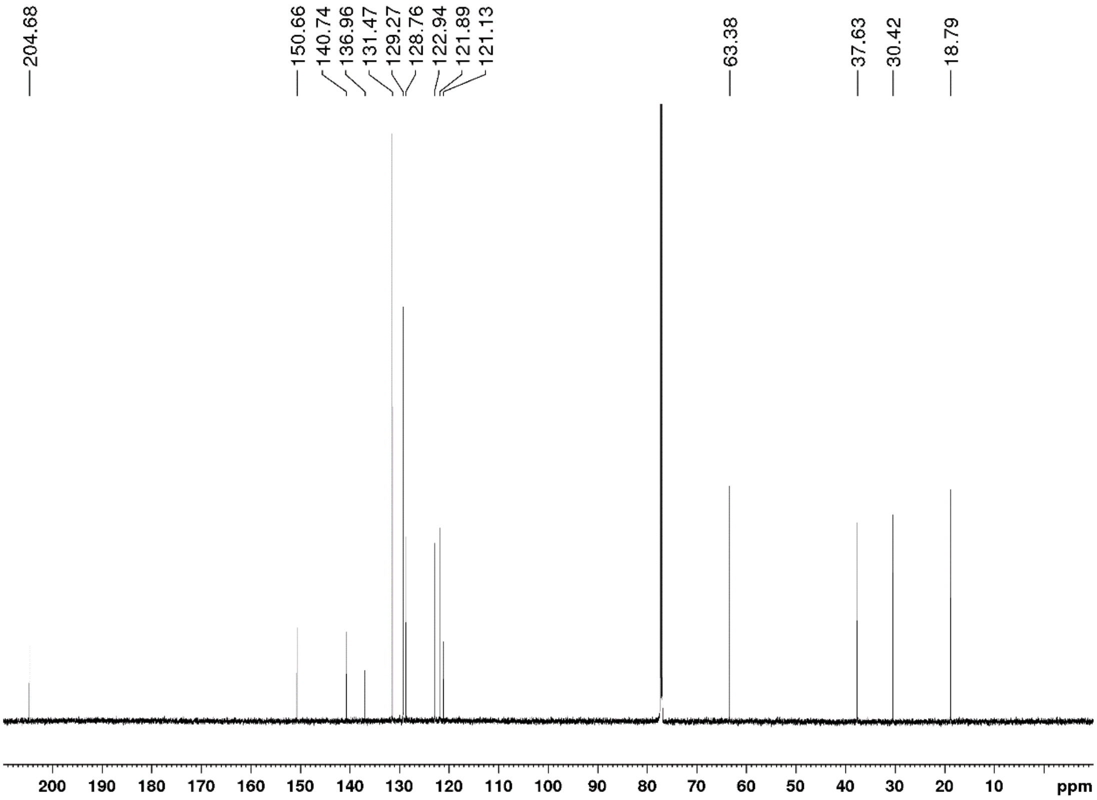

^{1}**H-NMR** (700 MHz, 298 K, $CDCl_3$) (**S3b**)

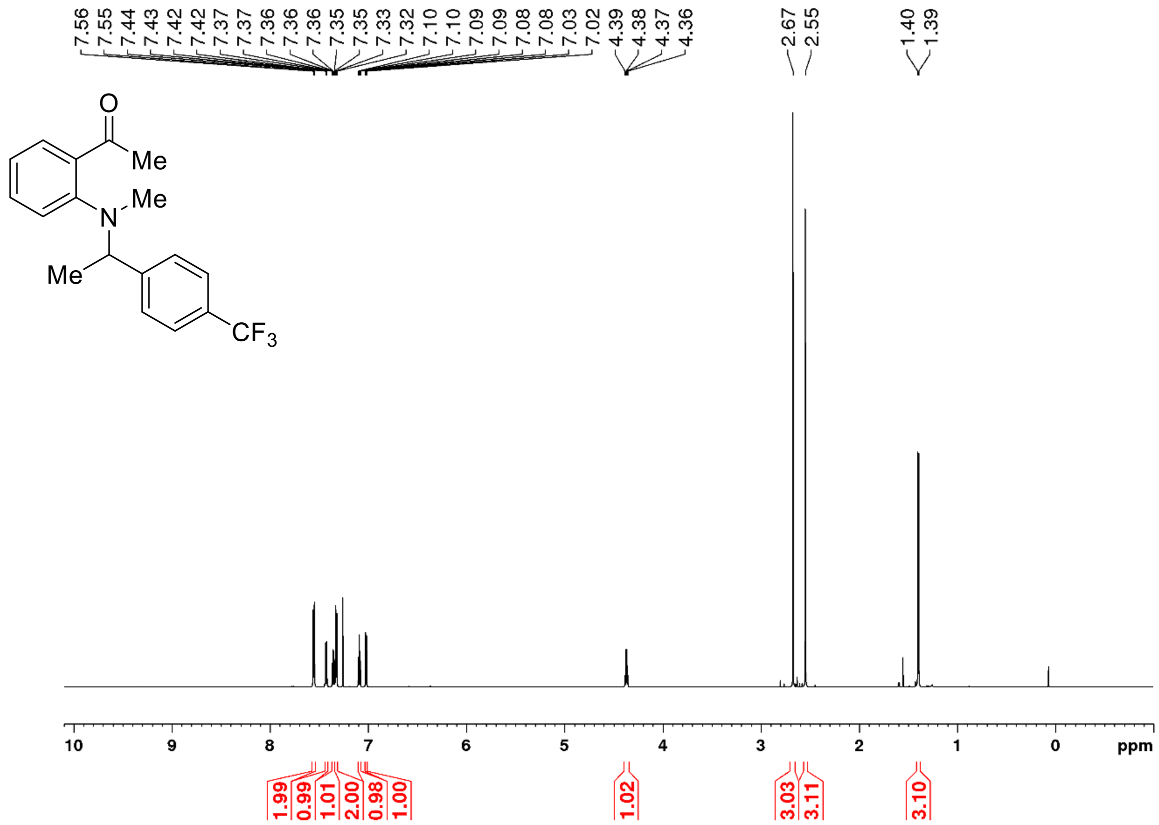

^{13}**C-NMR** (176 MHz, 298 K, $CDCl_3$)

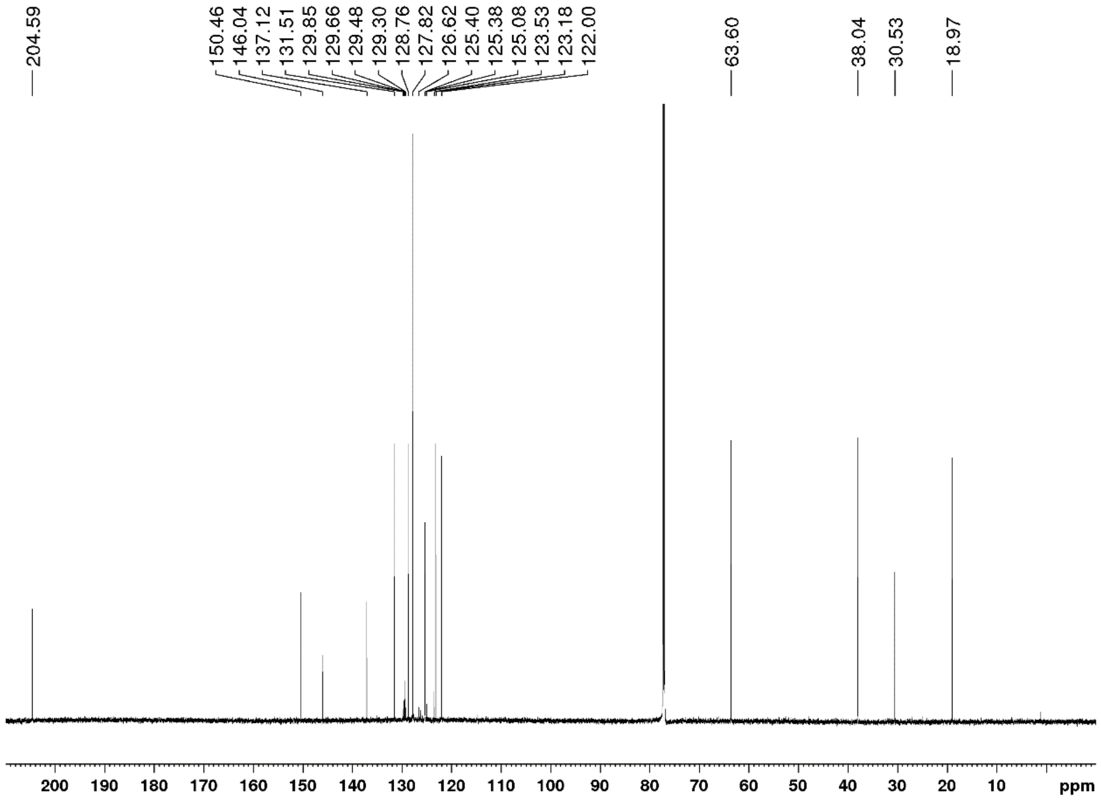

^{1}H-NMR (700 MHz, 298 K, $CDCl_3$) (**S3c**)

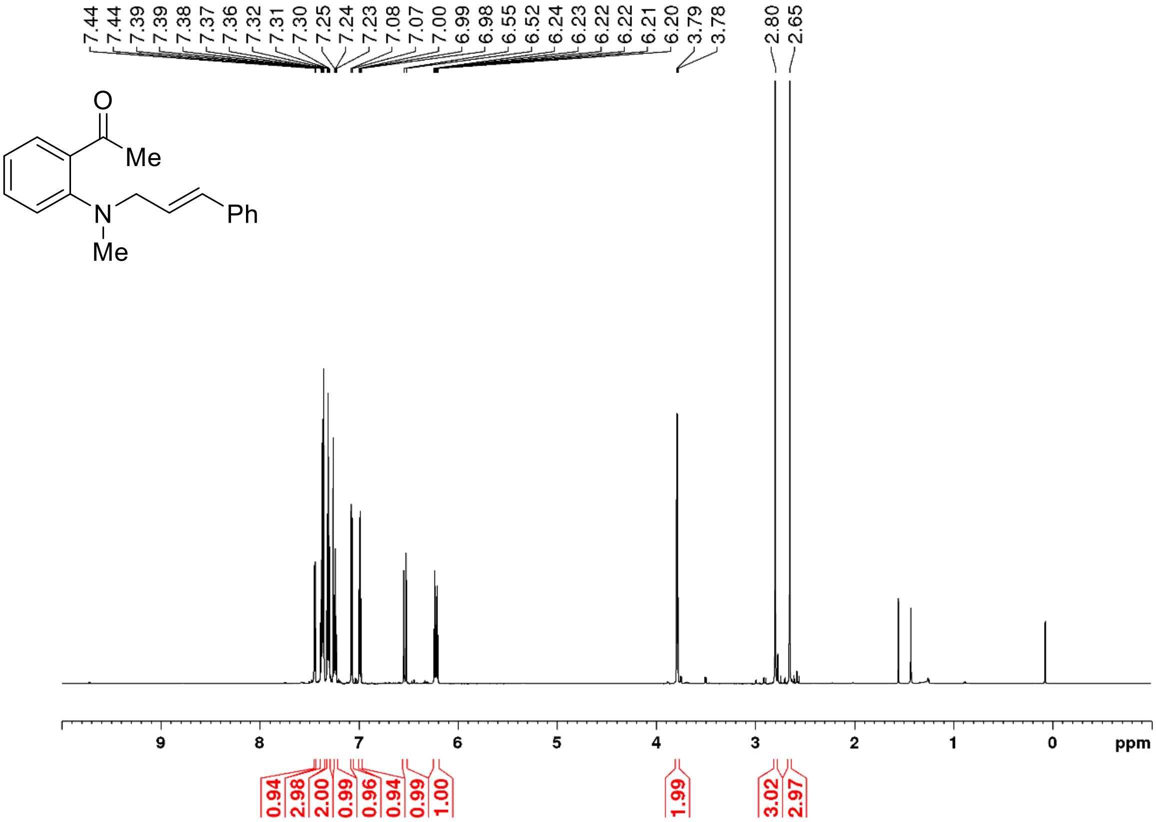

^{13}C-NMR (176 MHz, 298 K, $CDCl_3$)

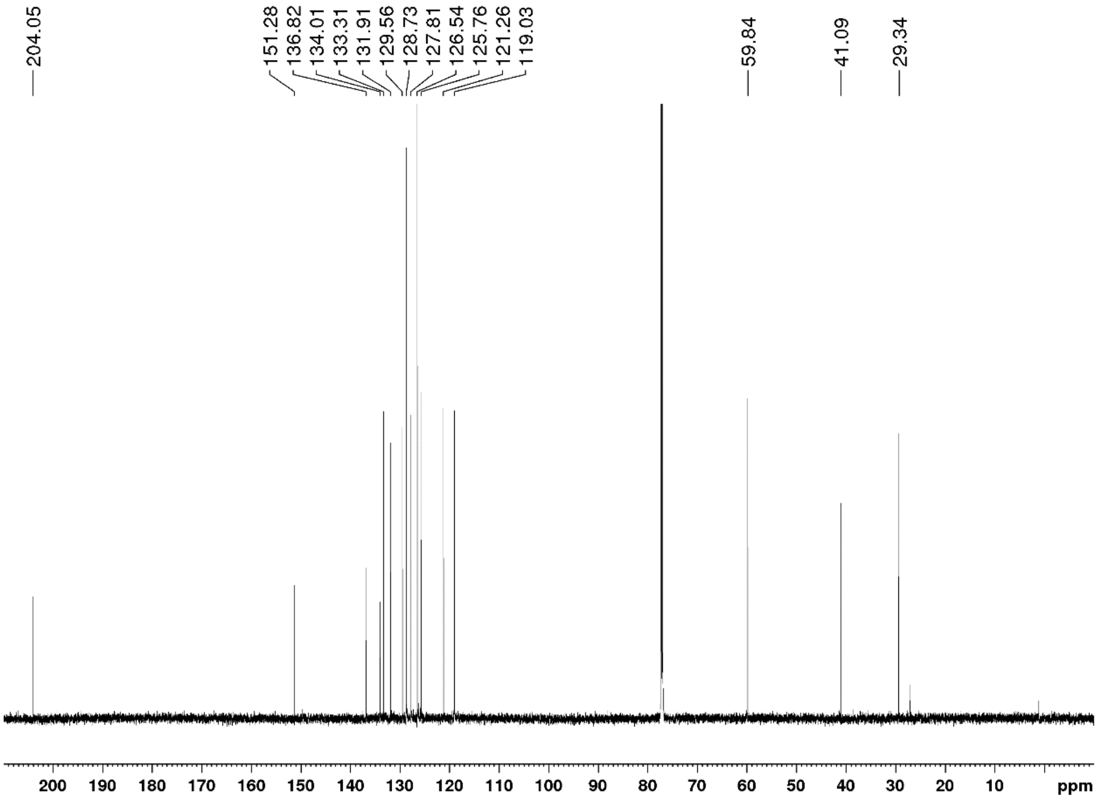

^{1}H-NMR (700 MHz, 298 K, $CDCl_3$) (**S3d**)

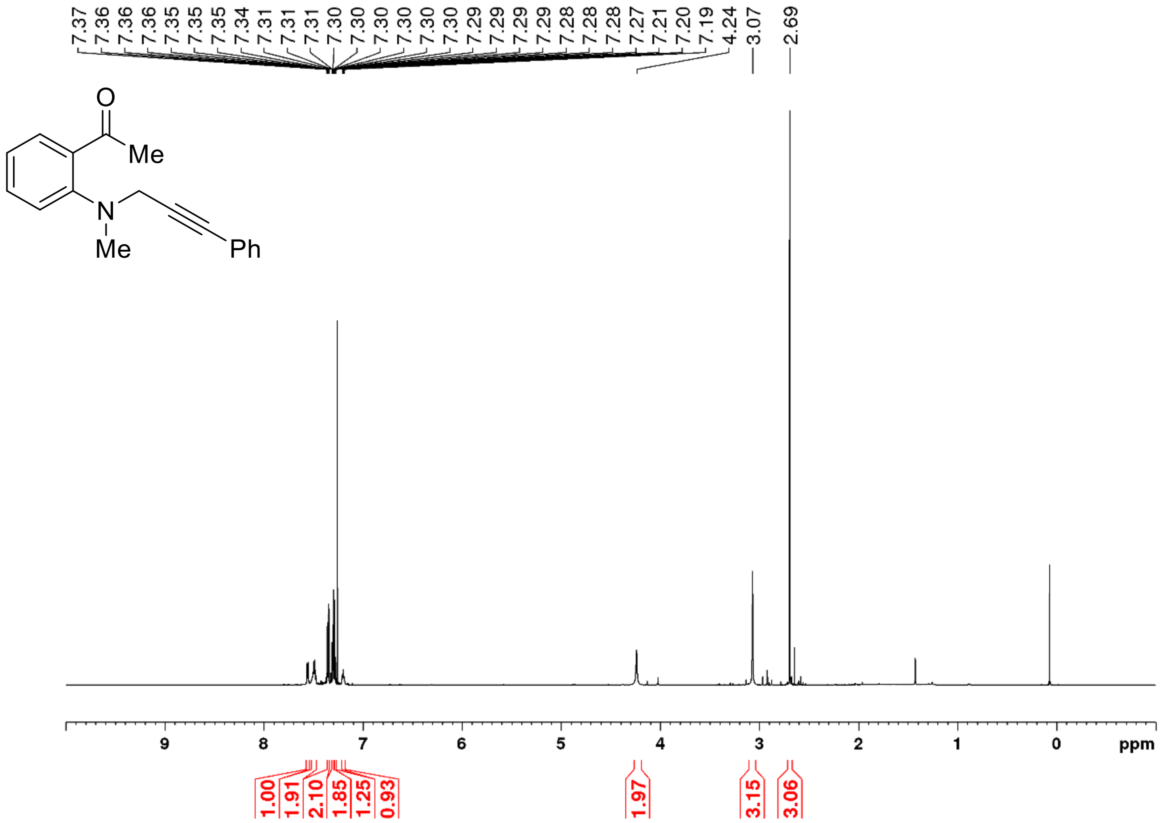

^{13}C-NMR (176 MHz, 298 K, $CDCl_3$)

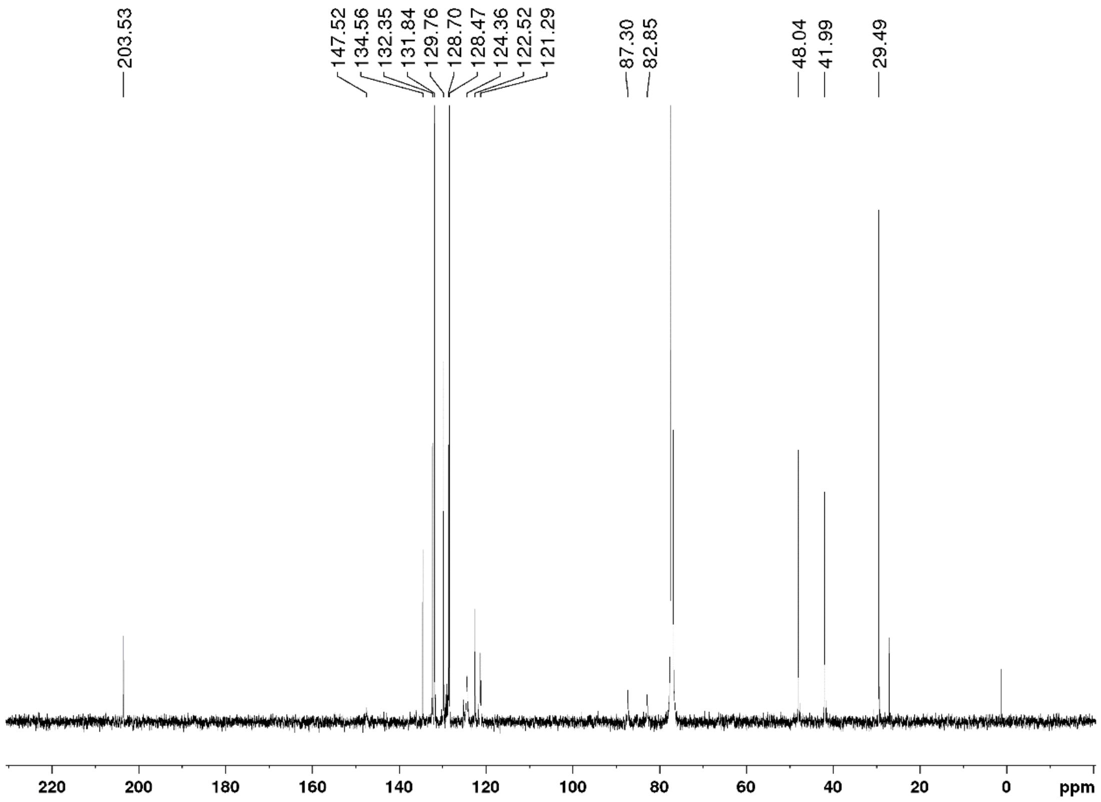

^{1}H-NMR (700 MHz, 298 K, $CDCl_3$) (**S3f**)

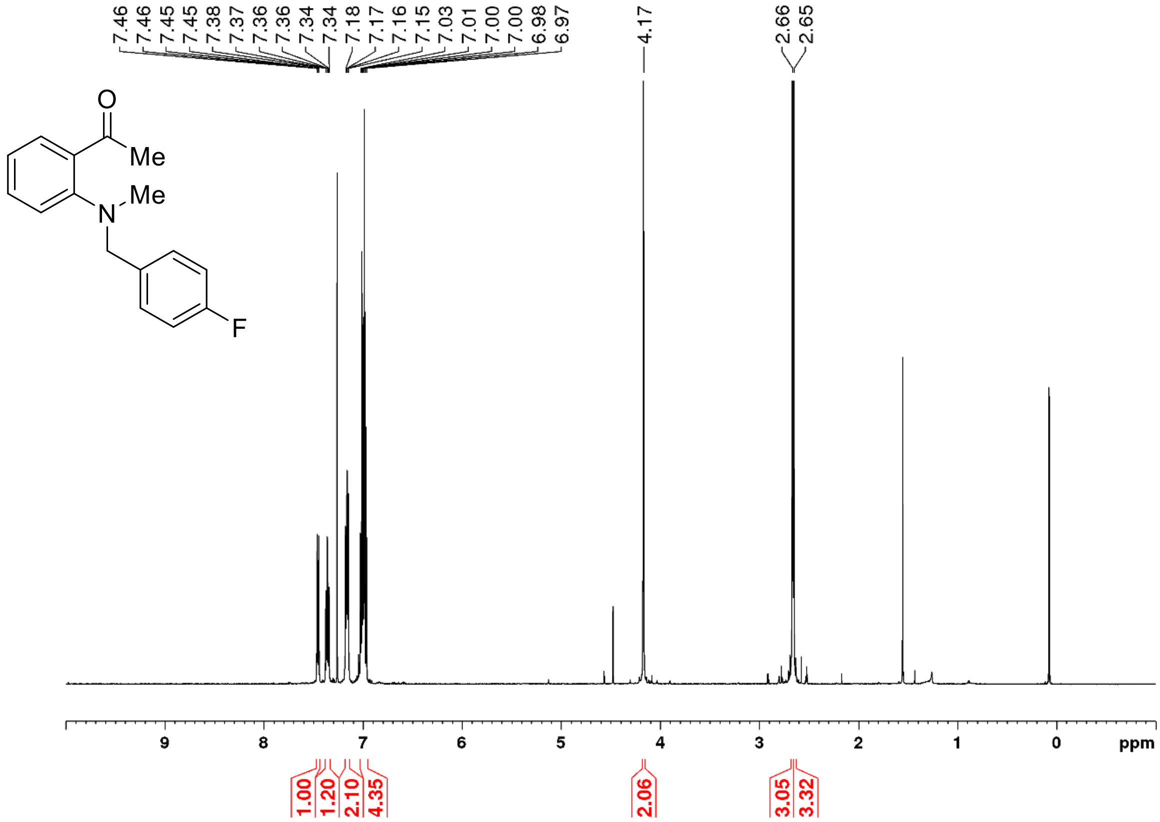

^{13}C-NMR (176 MHz, 298 K, $CDCl_3$)

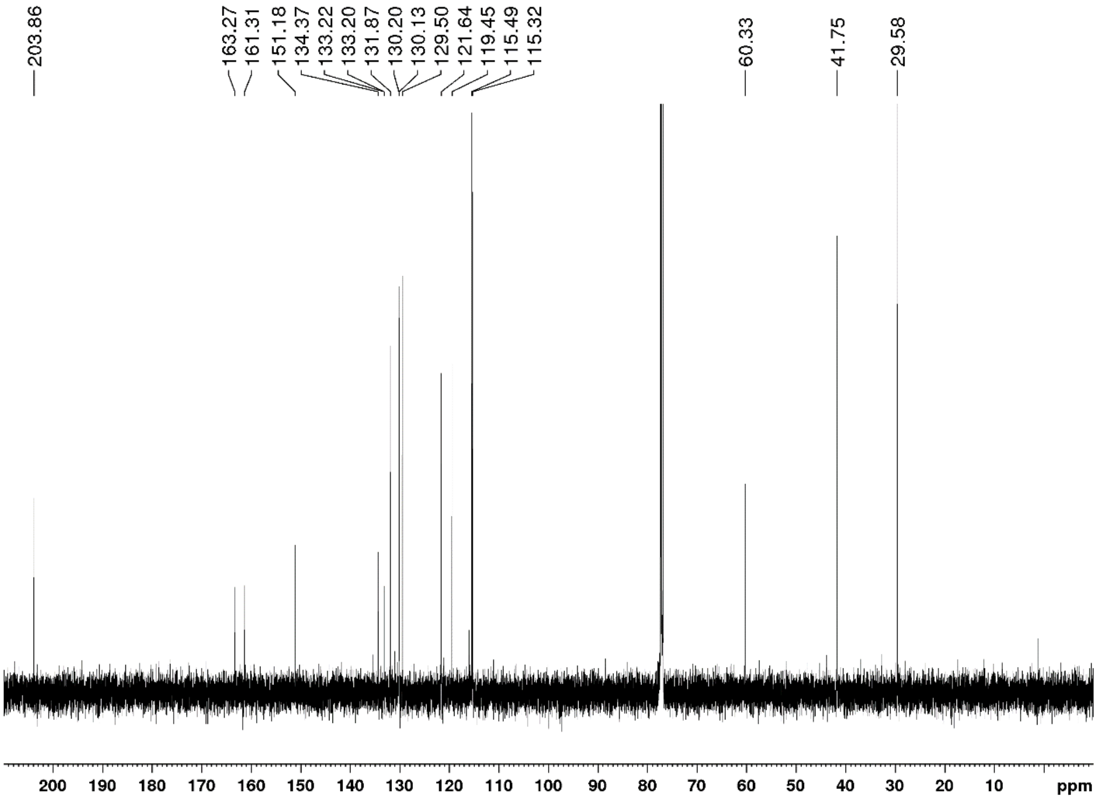

^{1}H-NMR (700 MHz, 298 K, $CDCl_3$) (**S3g**)

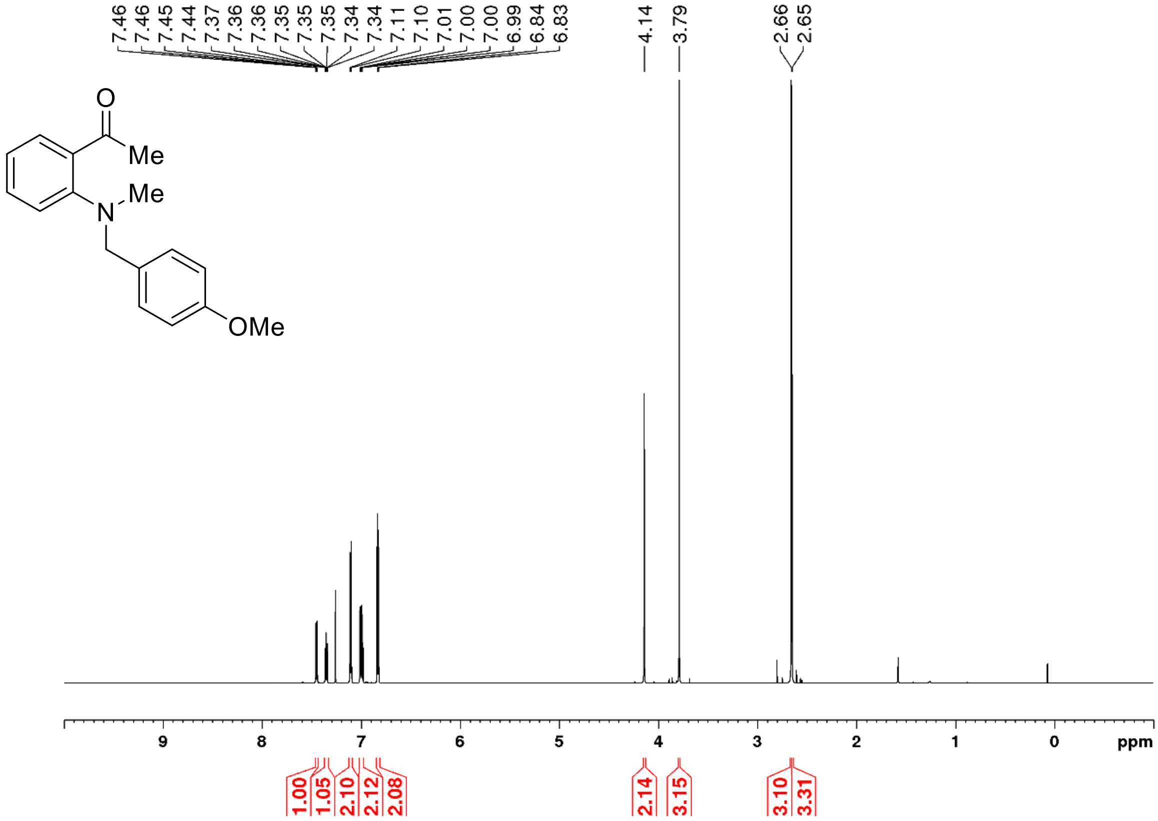

^{13}C-NMR (176 MHz, 298 K, $CDCl_3$)

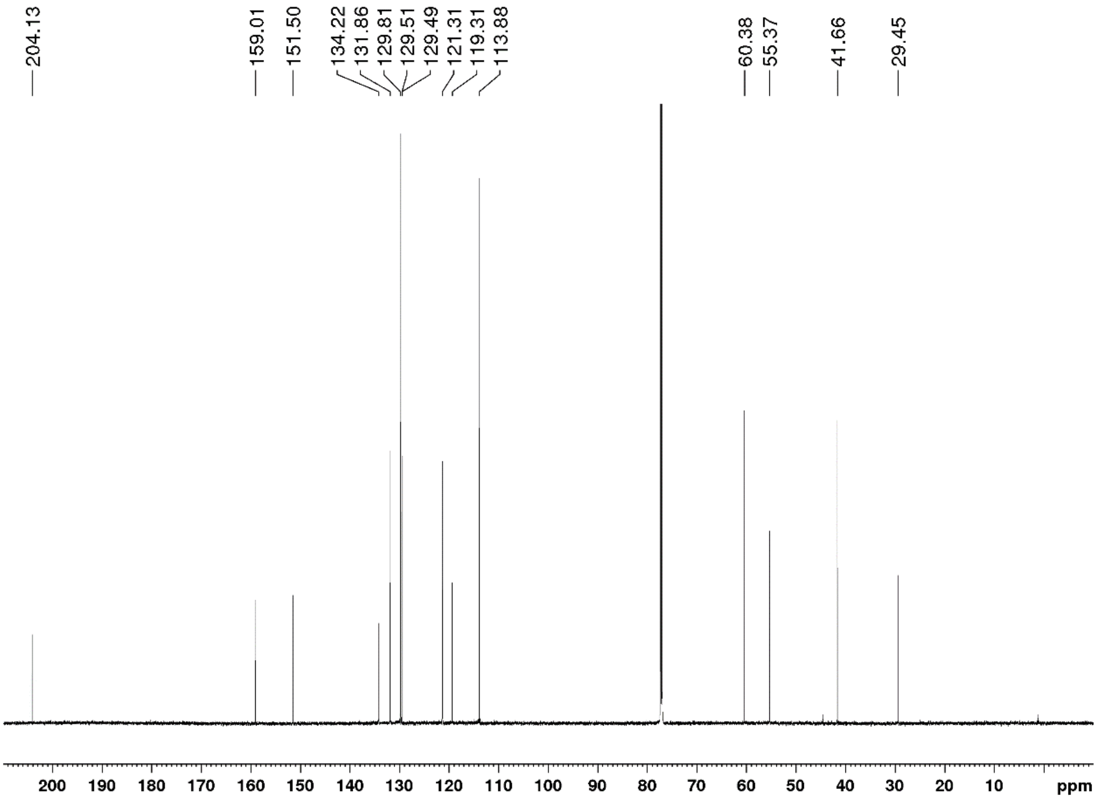

^{1}H-NMR (700 MHz, 298 K, $CDCl_3$) (**S3h**)

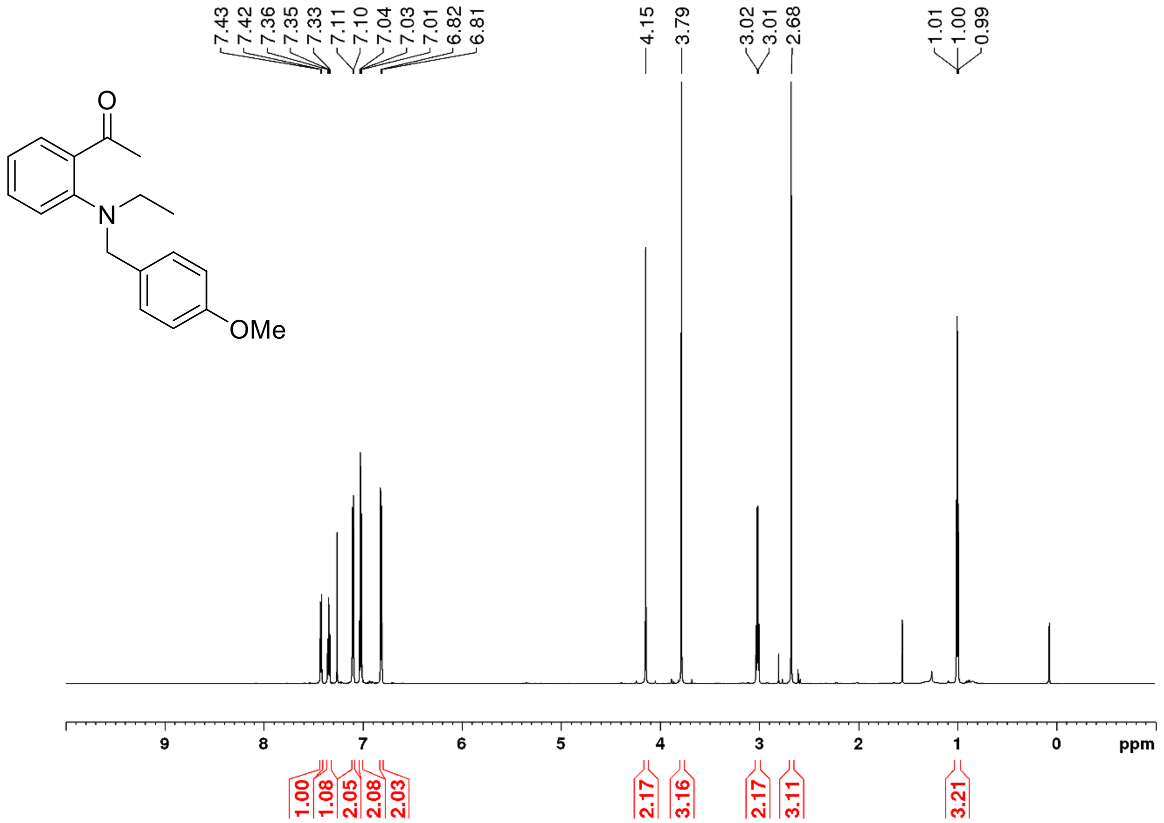

^{13}C-NMR (176 MHz, 298 K, $CDCl_3$)

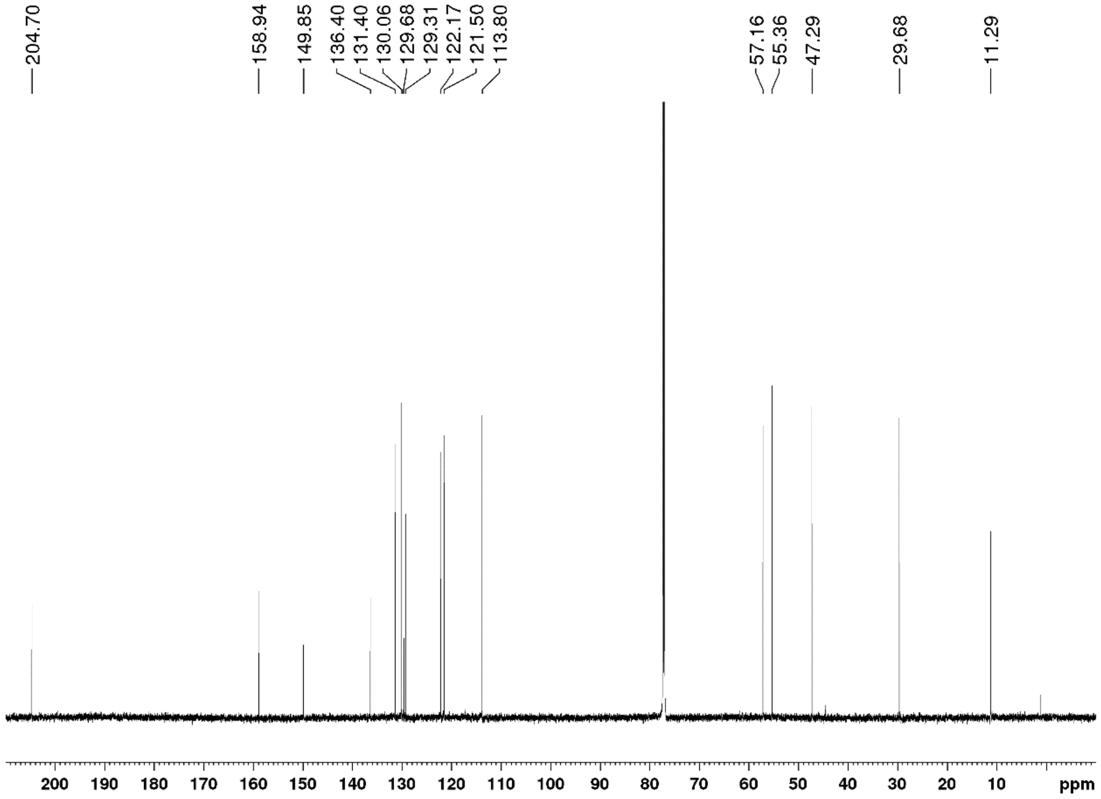

¹H-NMR (700 MHz, 298 K, $CDCl_3$) (**S3i**)

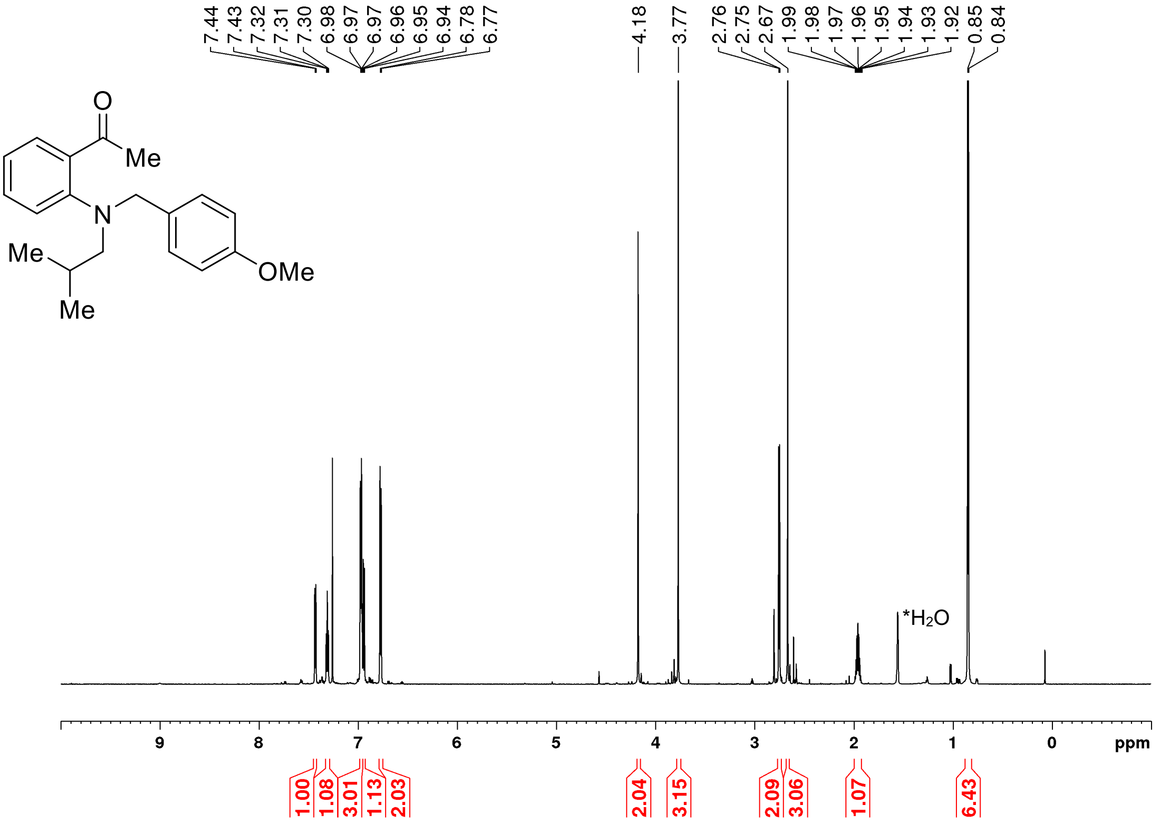

¹³C-NMR (176 MHz, 298 K, $CDCl_3$)

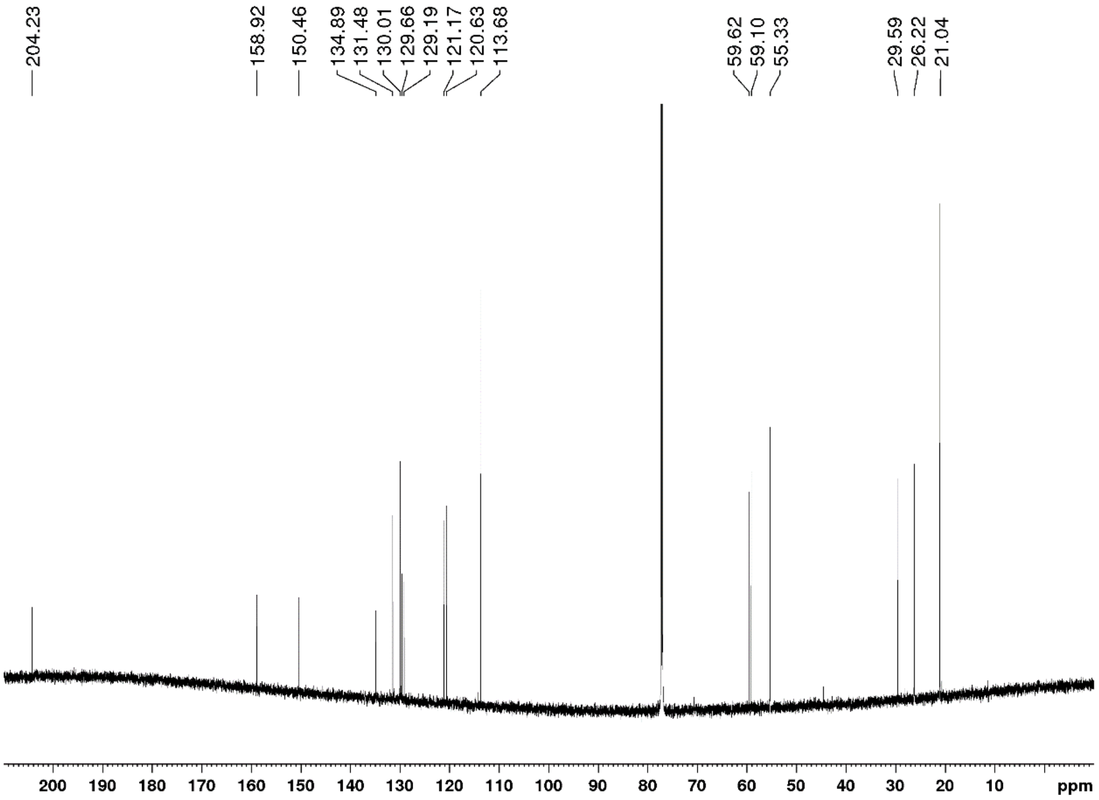

^{1}H-NMR (700 MHz, 298 K, $CDCl_3$) (**S3j**)

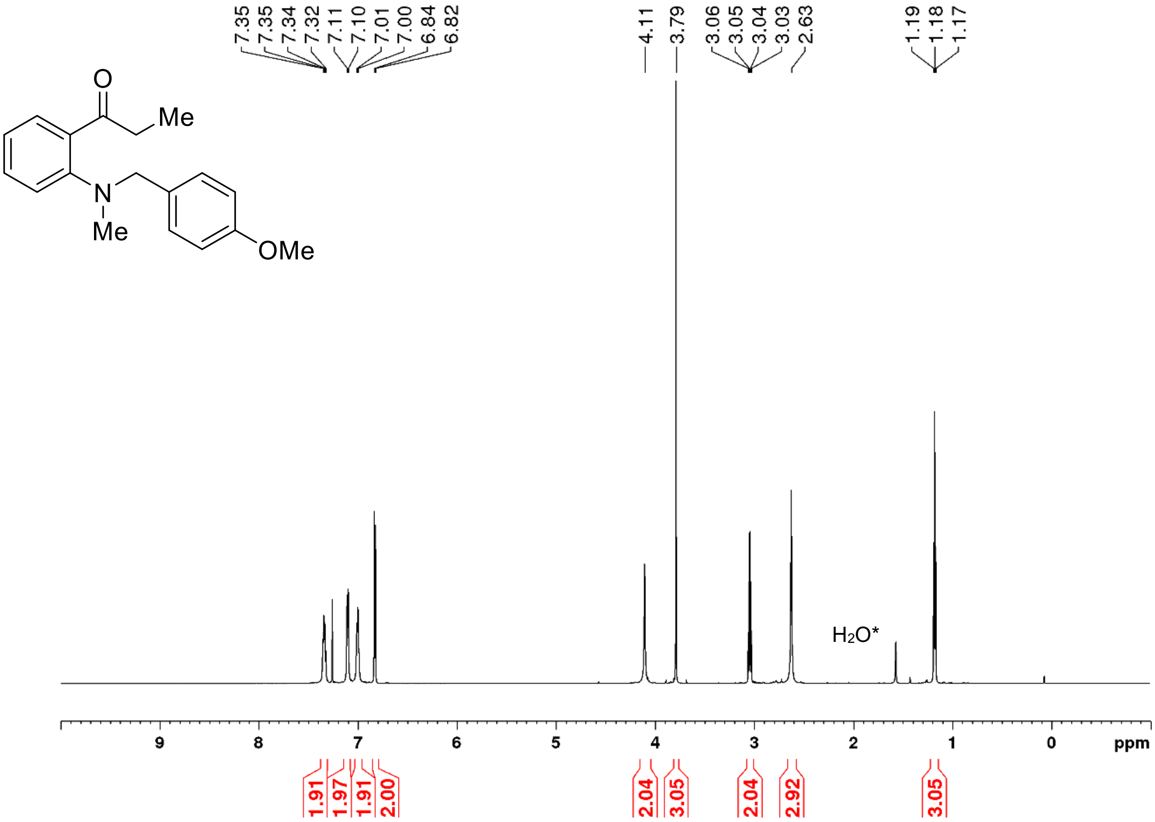

^{13}C-NMR (176 MHz, 298 K, $CDCl_3$)

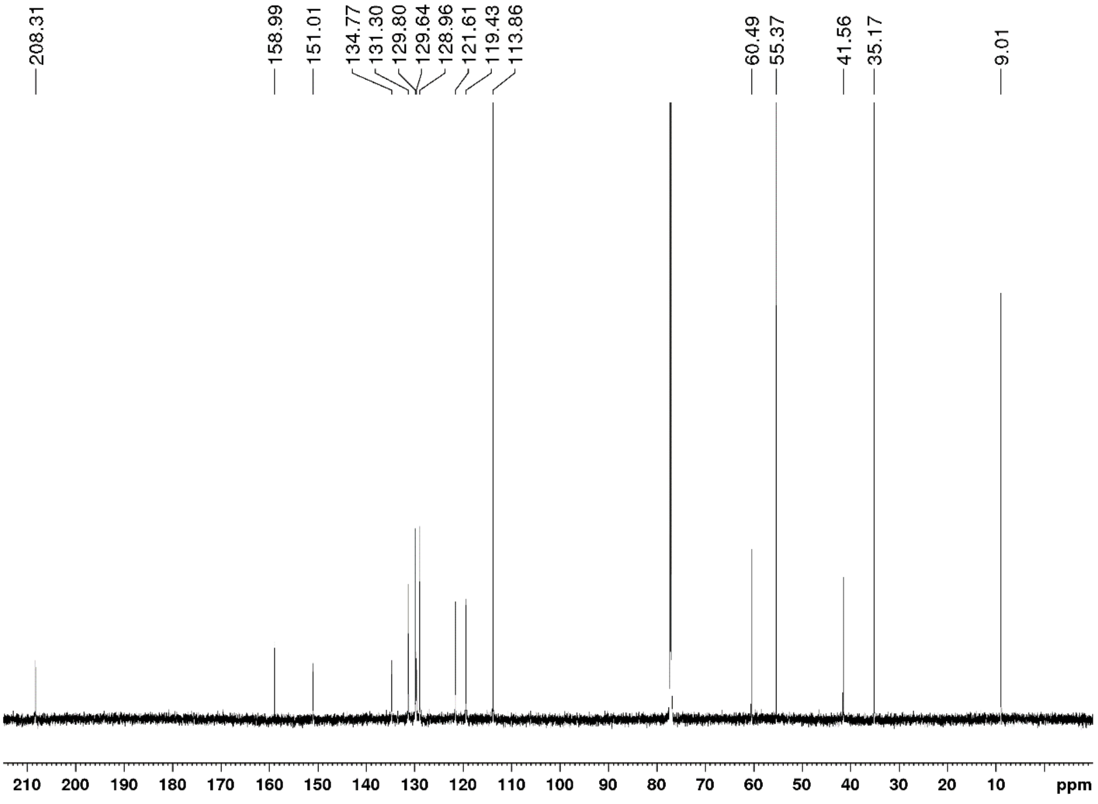

^{1}H-NMR (500 MHz, 303 K, $CDCl_3$) (**S3k**)

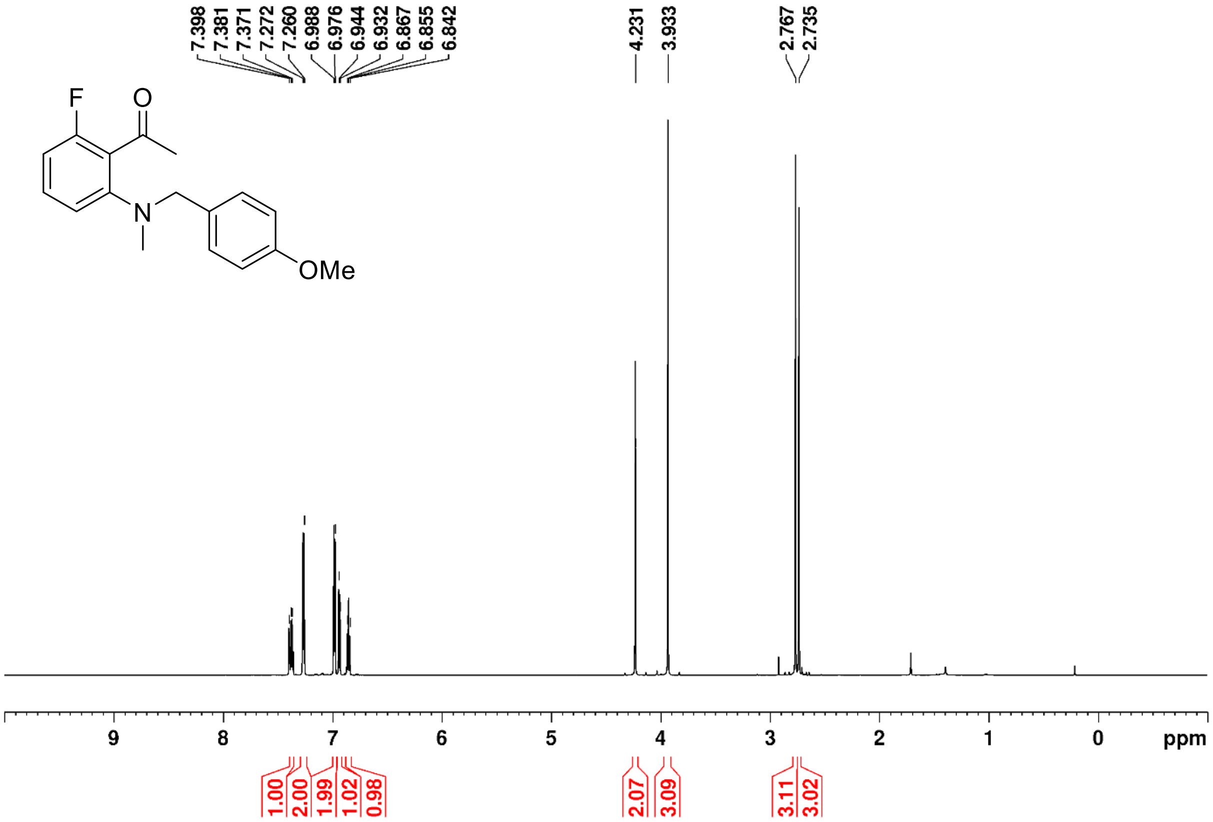

^{13}C-NMR (176 MHz, 298 K, $CDCl_3$) (**S3k**)

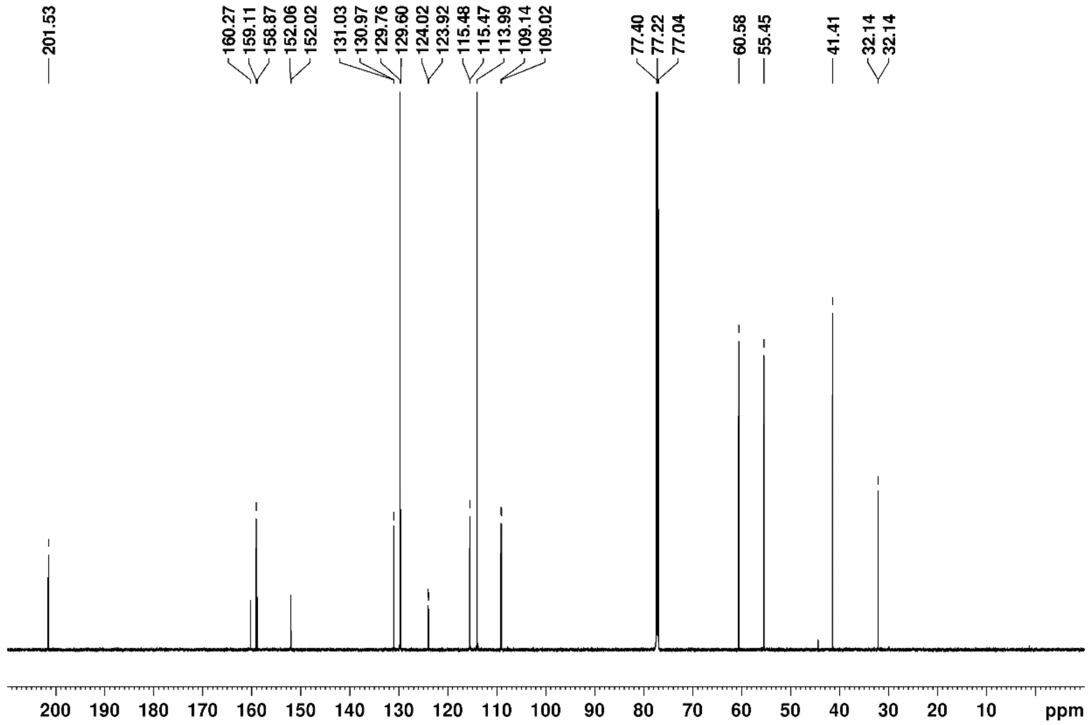

^{1}H-NMR (700 MHz, 298 K, $CDCl_3$) (S3I)

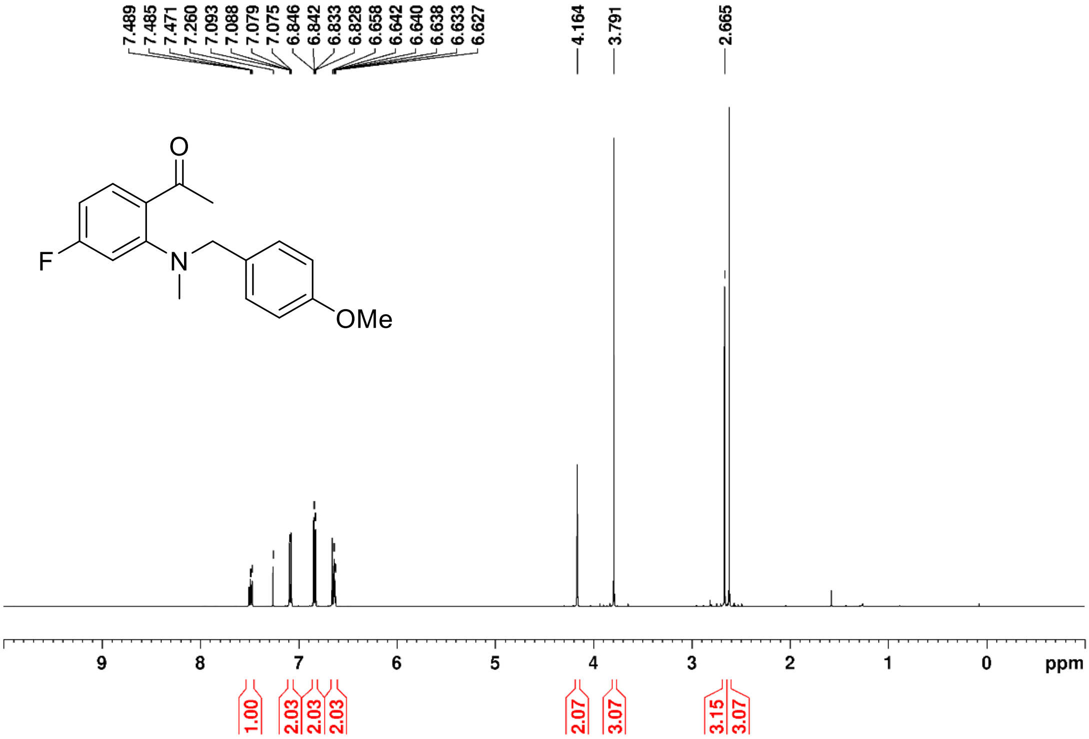

^{13}C-NMR (176 MHz, 298 K, $CDCl_3$) (S3I)

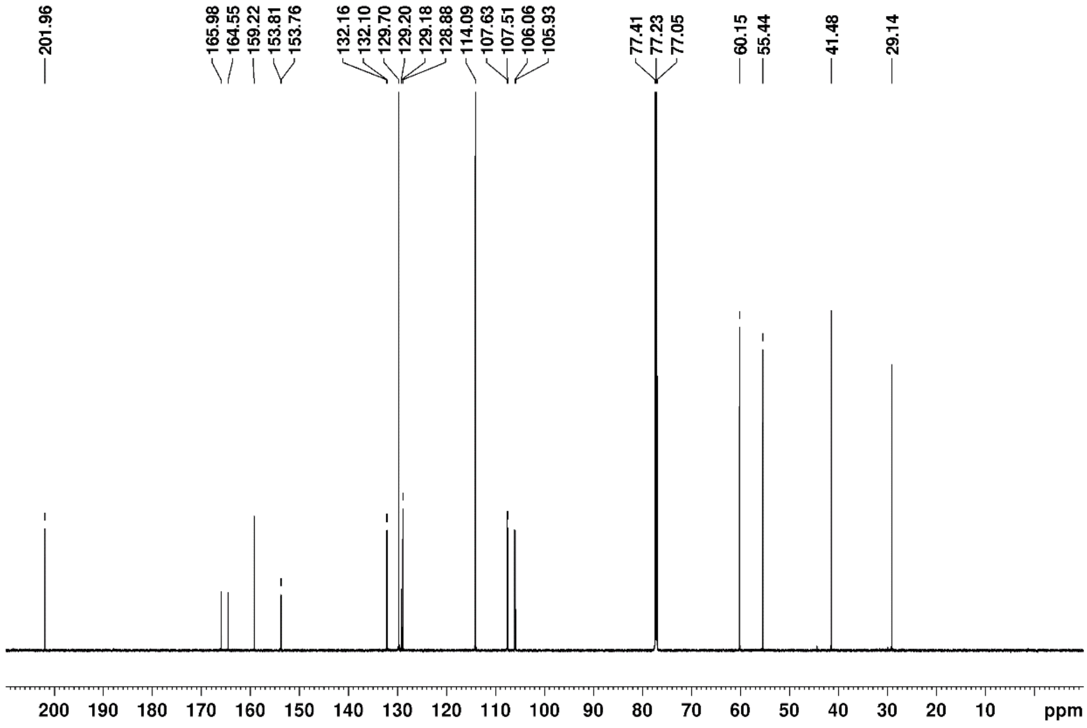

^{1}H-NMR (700 MHz, 298 K, $CDCl_3$) (**S3m**)

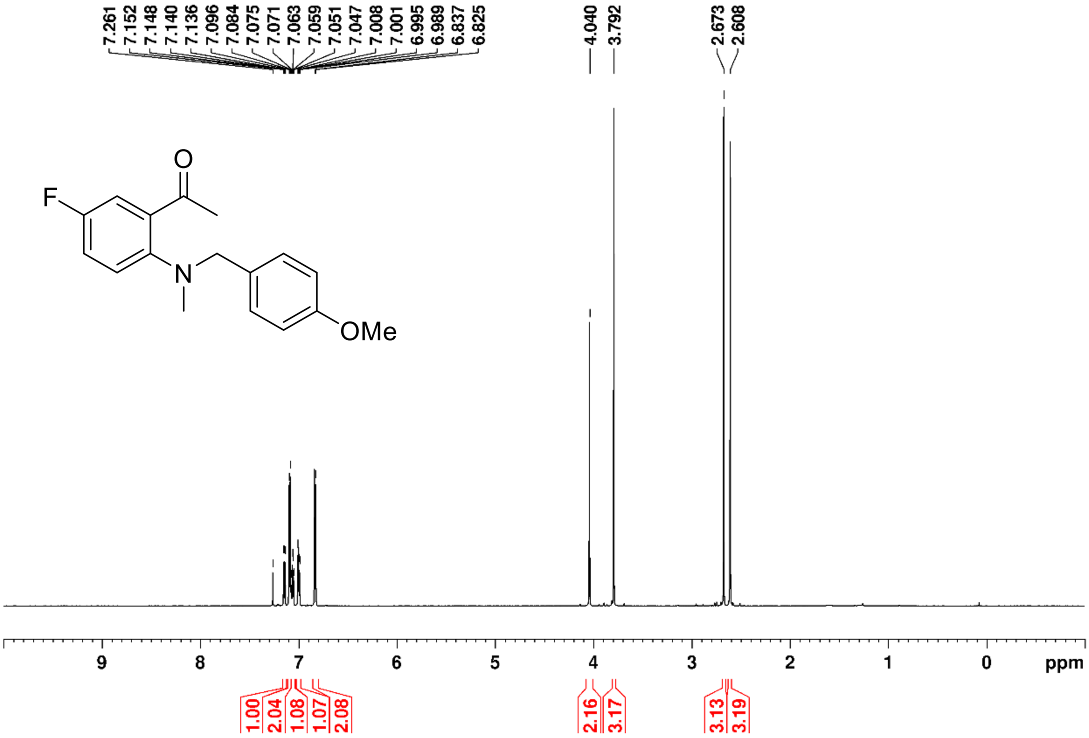

^{13}C-NMR (176 MHz, 298 K, $CDCl_3$) (**S3m**)

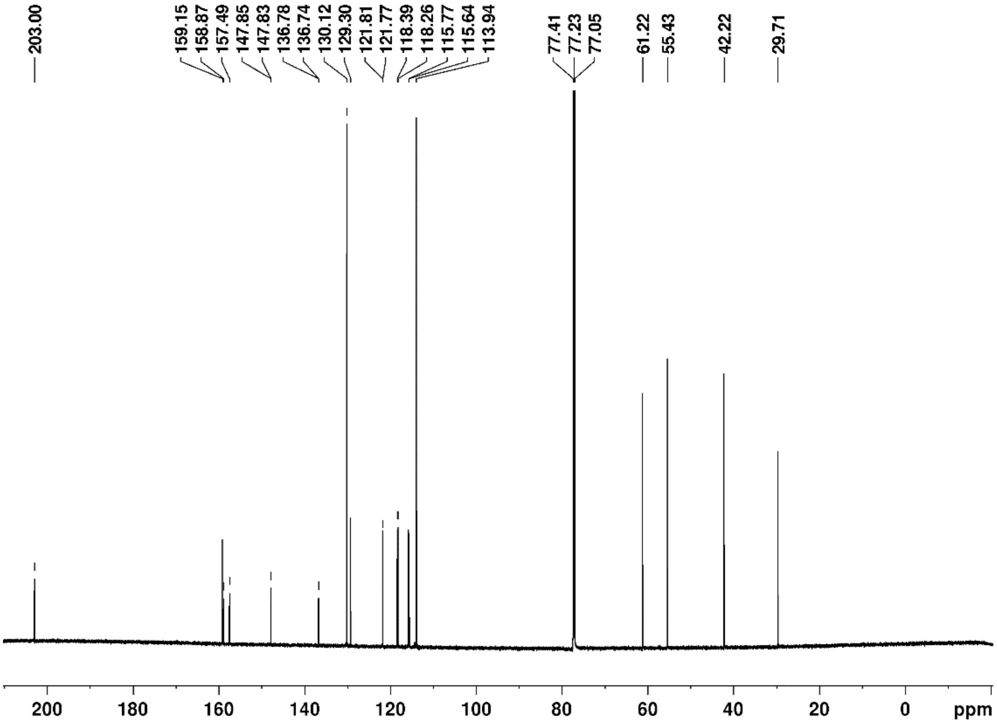

[1]**H-NMR** (700 MHz, 298 K, CDCl3) (**S3n**)

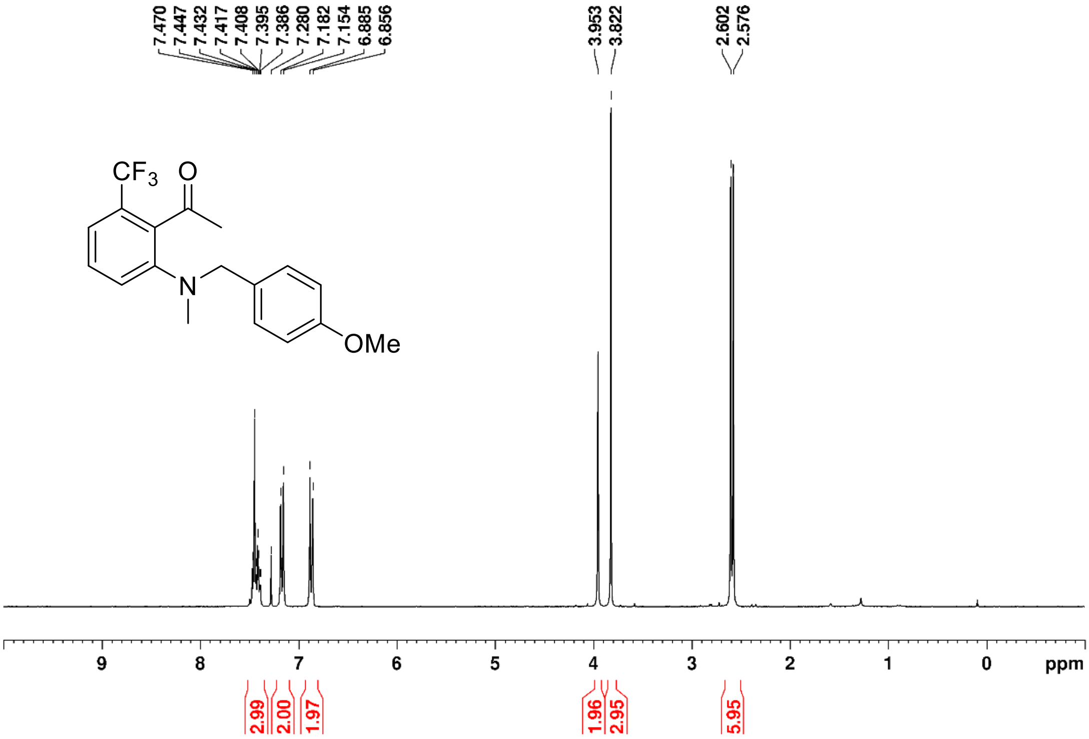

[13]**C-NMR** (176 MHz, 298 K, CDCl3) (**S3n**)

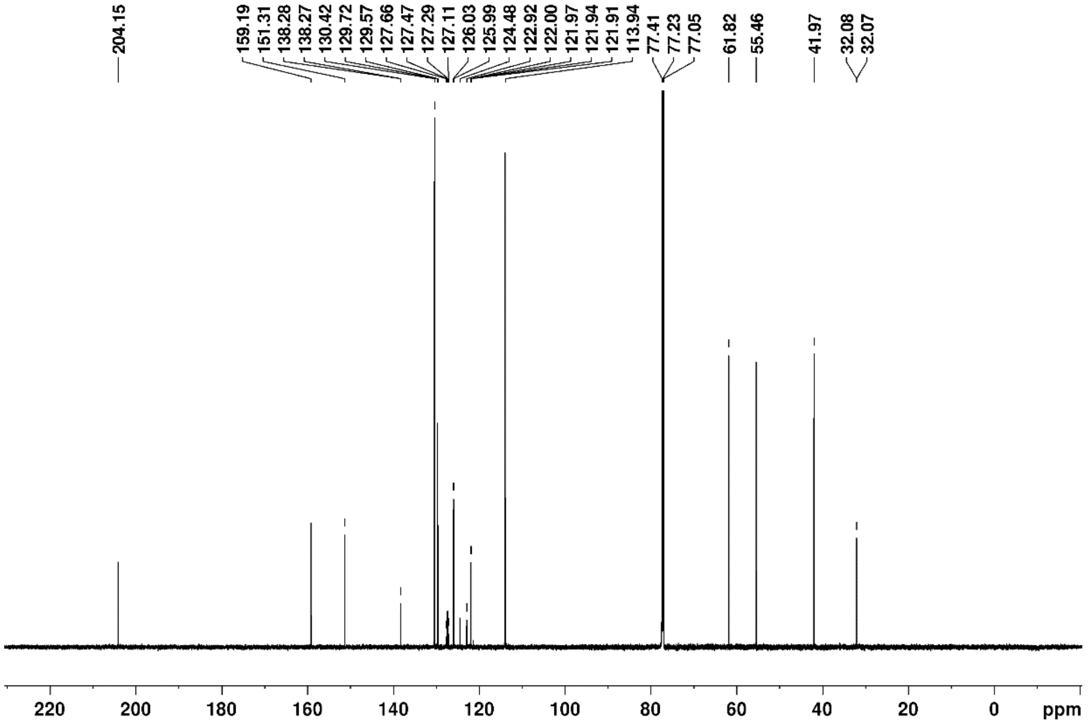

^{1}H-NMR (500 MHz, 303 K, $CDCl_3$) (**S3o**)

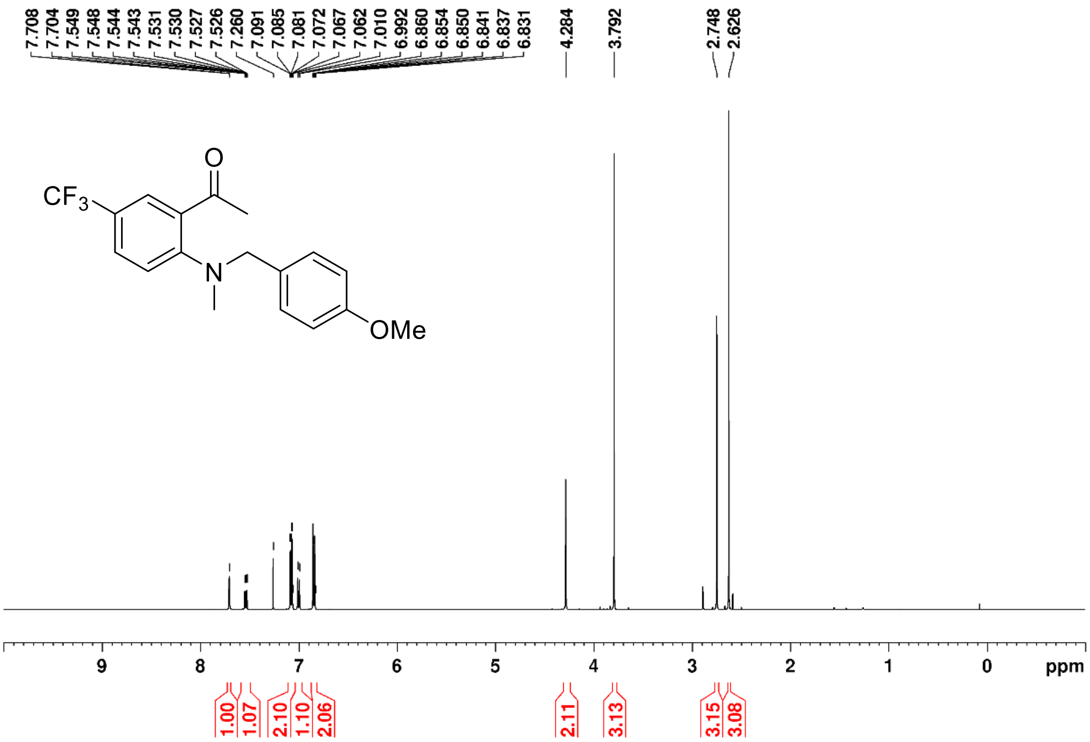

^{13}C-NMR (176 MHz, 298 K, $CDCl_3$) (**S3o**)

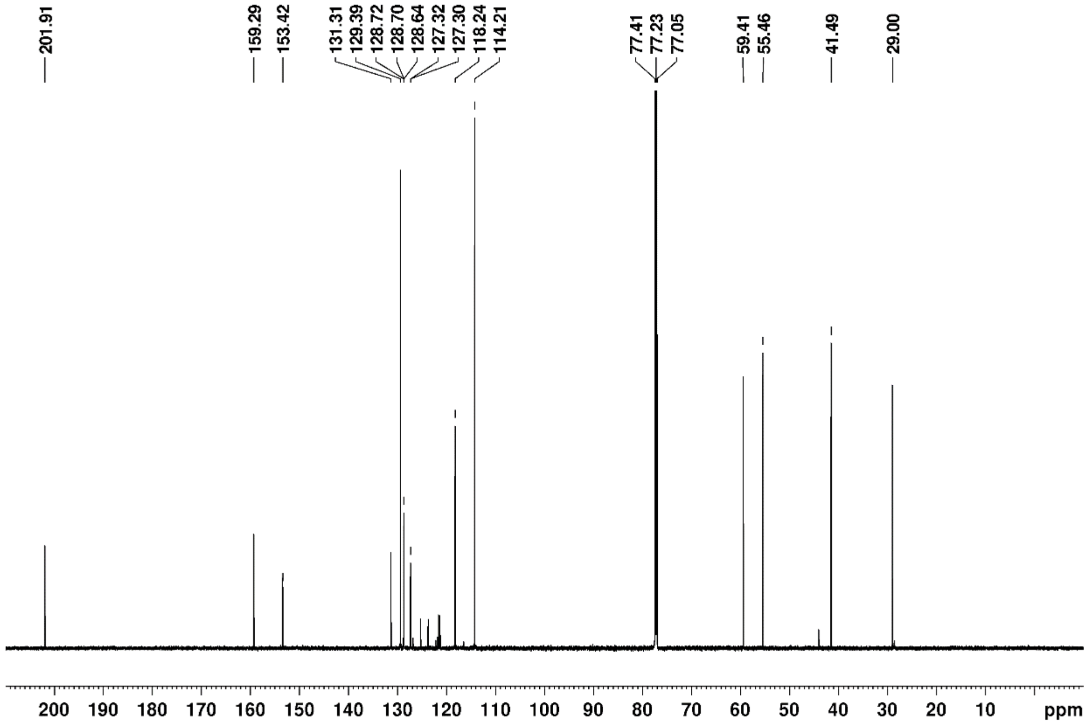

^{1}**H-NMR** (500 MHz, 303 K, $CDCl_3$) (**S3q**)

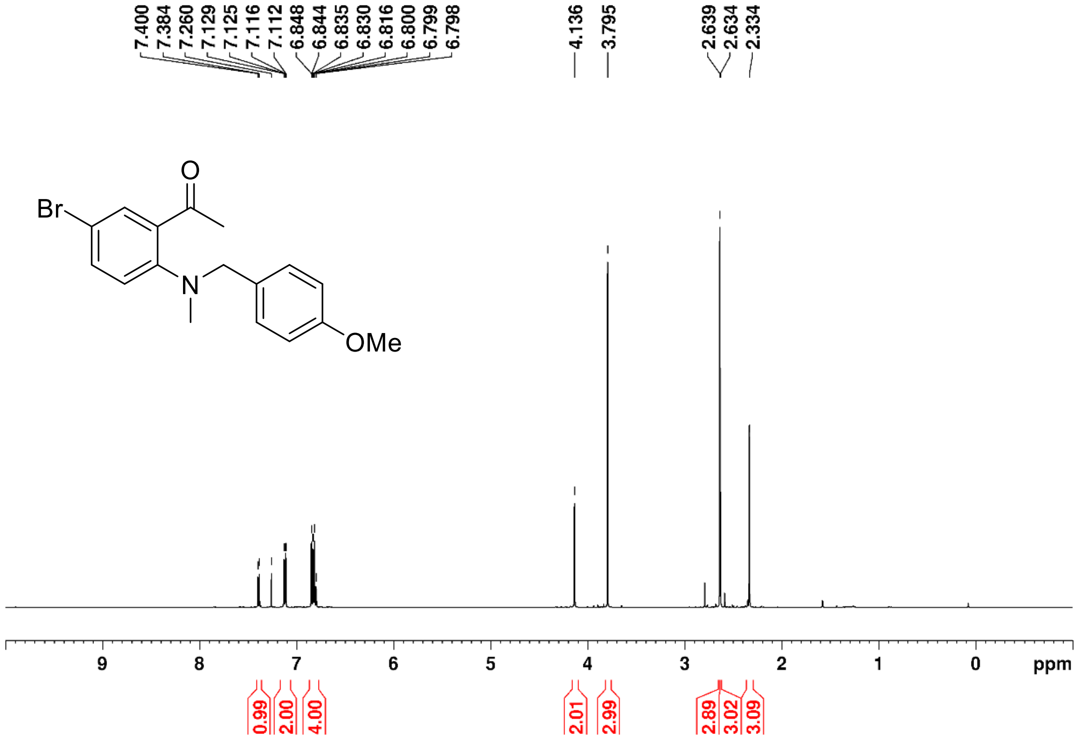

^{13}**C-NMR** (176 MHz, 298 K, $CDCl_3$) (**S3q**)

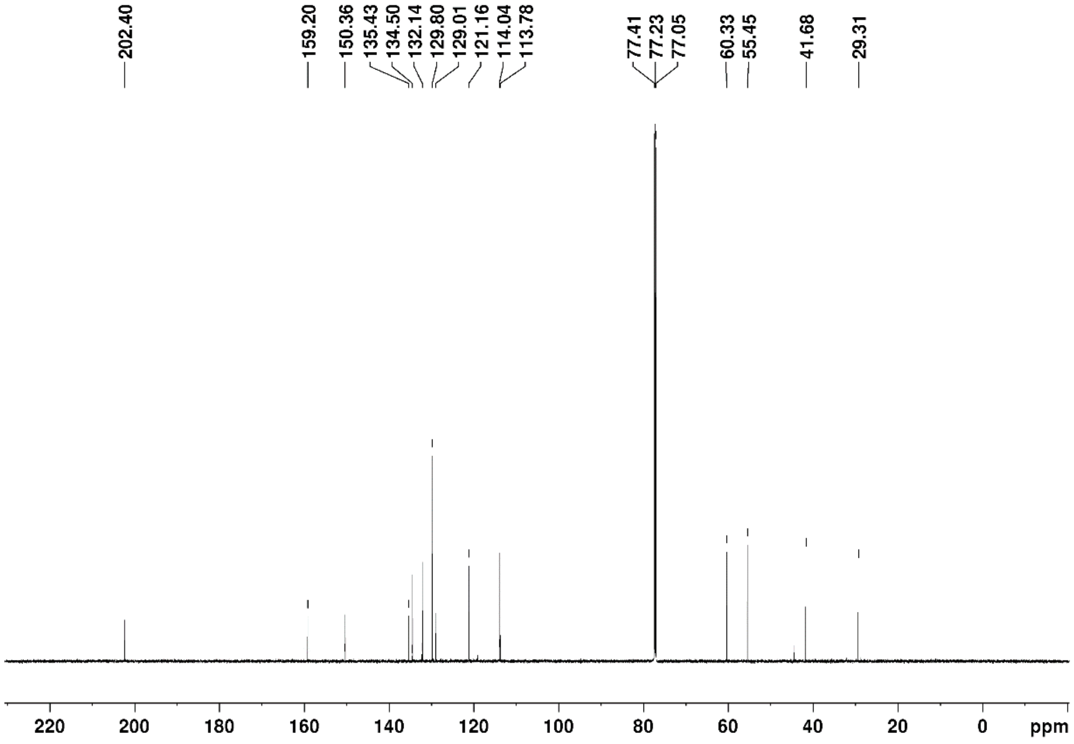

¹H-NMR (500 MHz, 303 K, $CDCl_3$) (**S3r**)

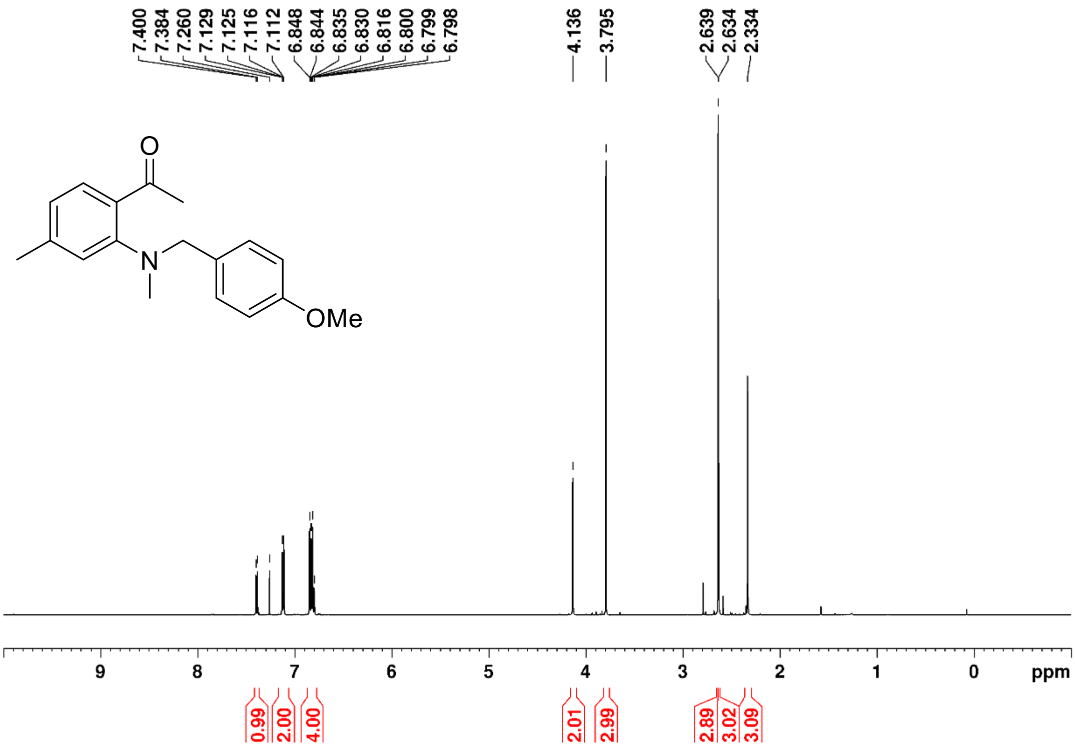

¹³C-NMR (176 MHz, 298 K, $CDCl_3$) (**S3r**)

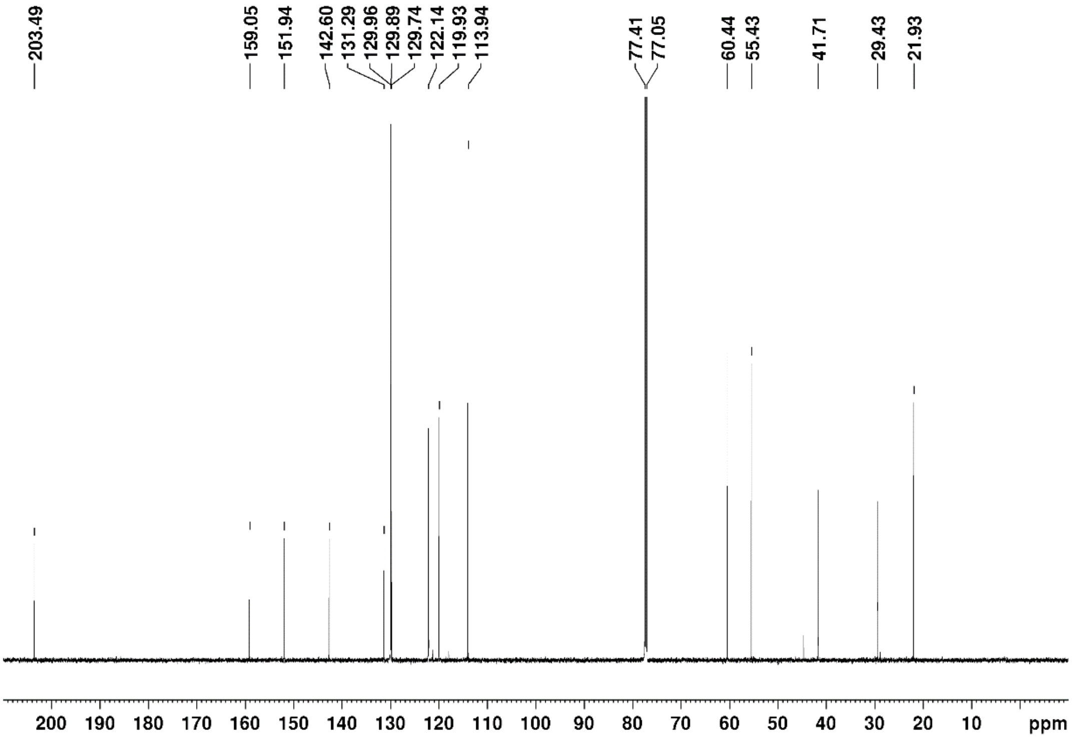

^{1}**H-NMR** (700 MHz, 298 K, $CDCl_3$) (**S3s**)

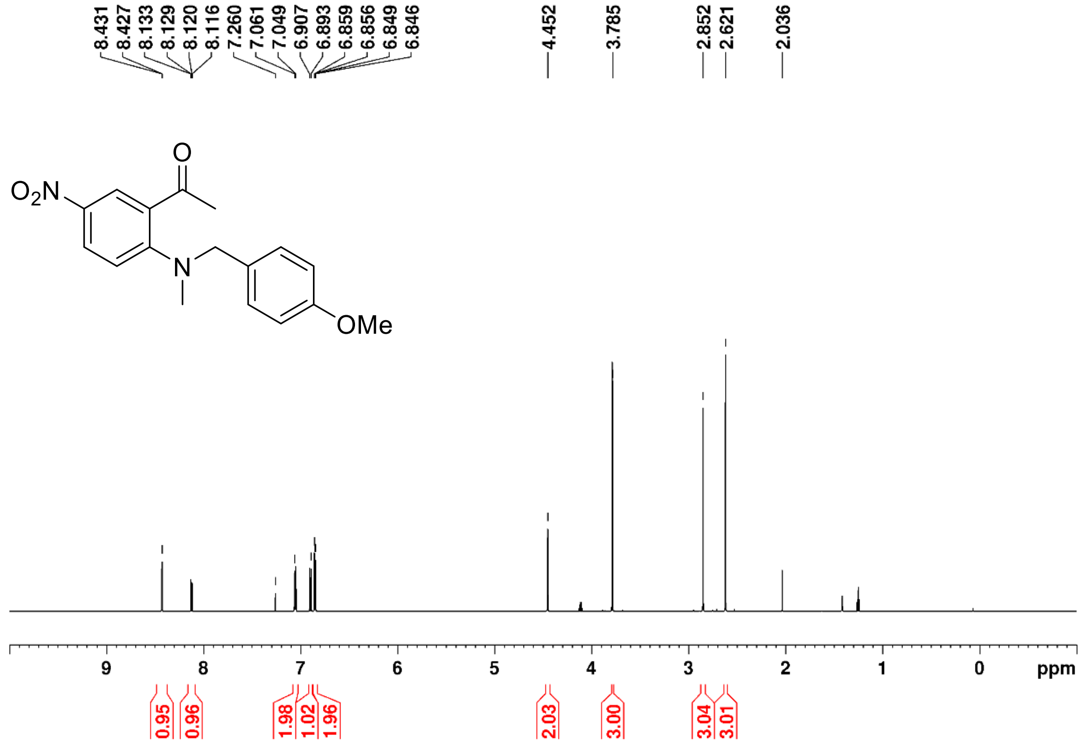

^{13}**C-NMR** (176 MHz, 298 K, $CDCl_3$) (**S3s**)

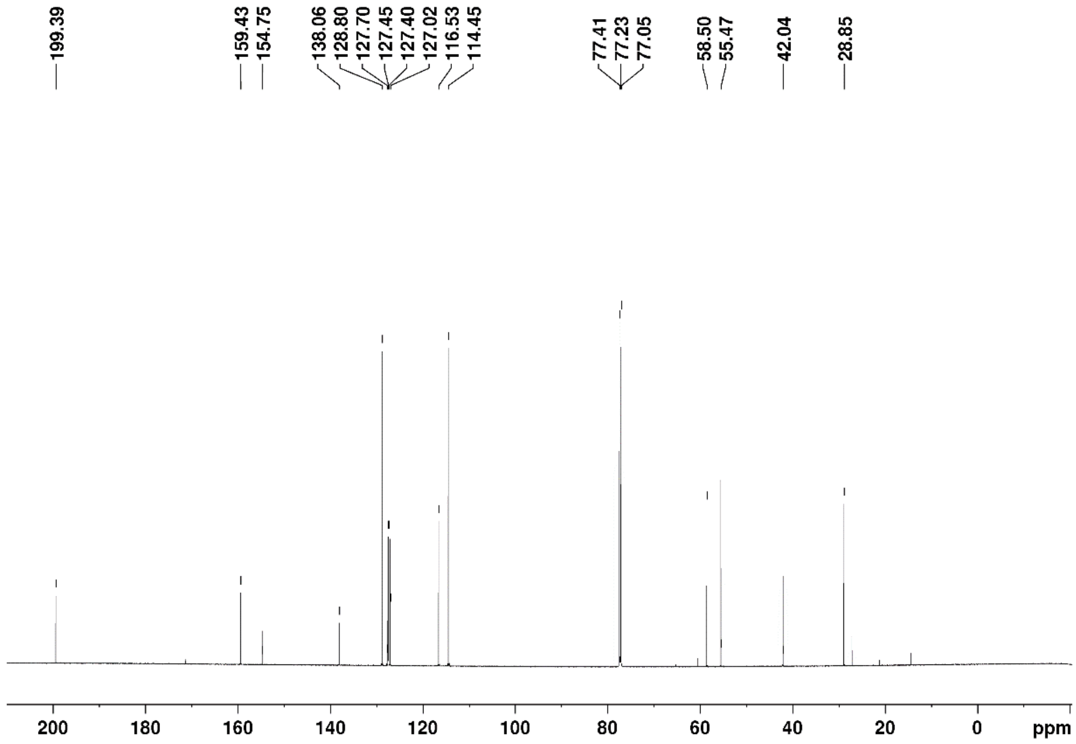

^{1}H-NMR (700 MHz, 298 K, $CDCl_3$) (**S3t**)

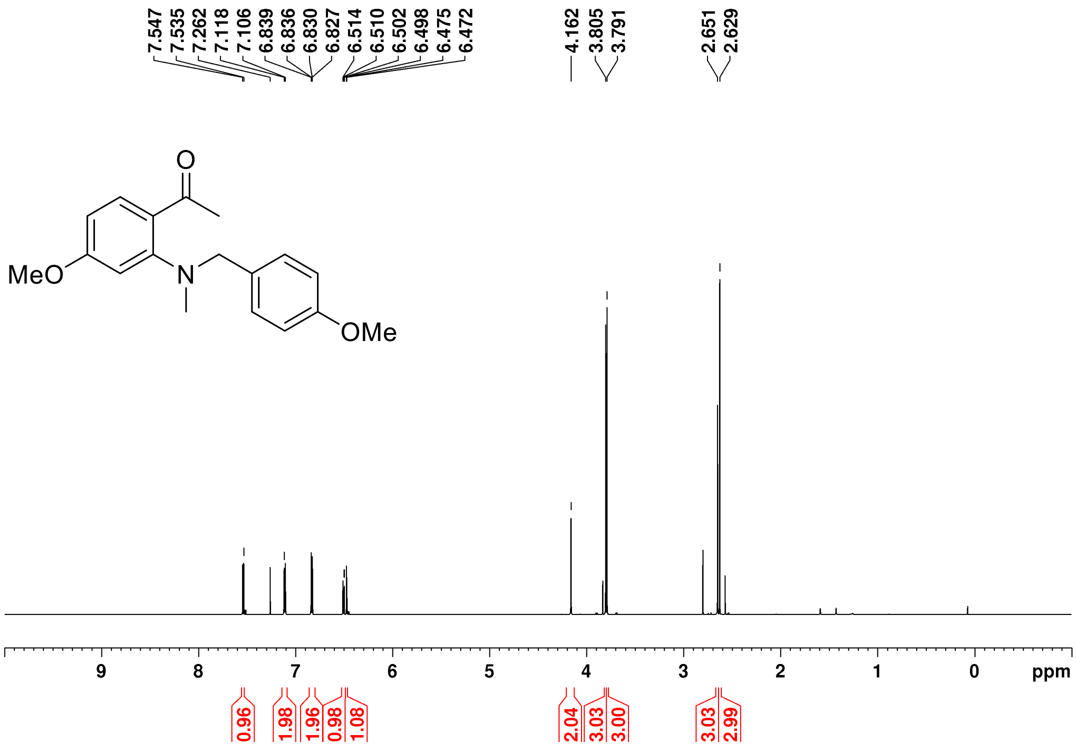

^{13}C-NMR (176 MHz, 298 K, $CDCl_3$) (**S3t**)

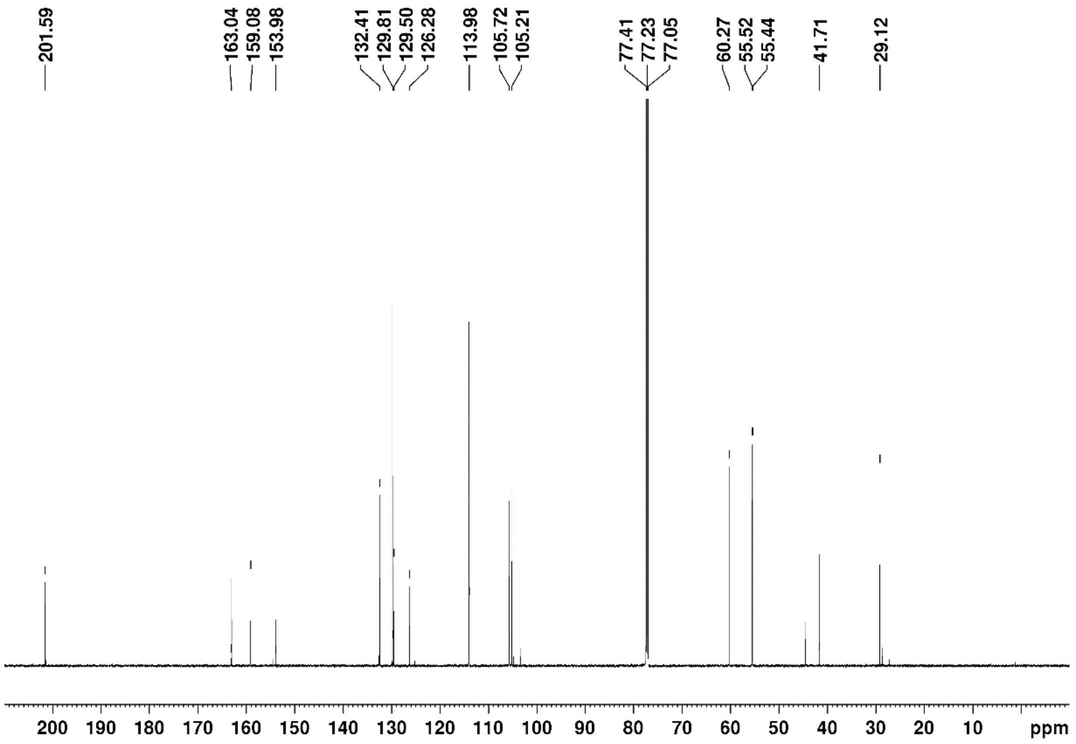

3.2 NMR spectra of starting materials

^{1}**H-NMR** (500 MHz, 303 K, CD_2Cl_2) (*rac*-**1a**)

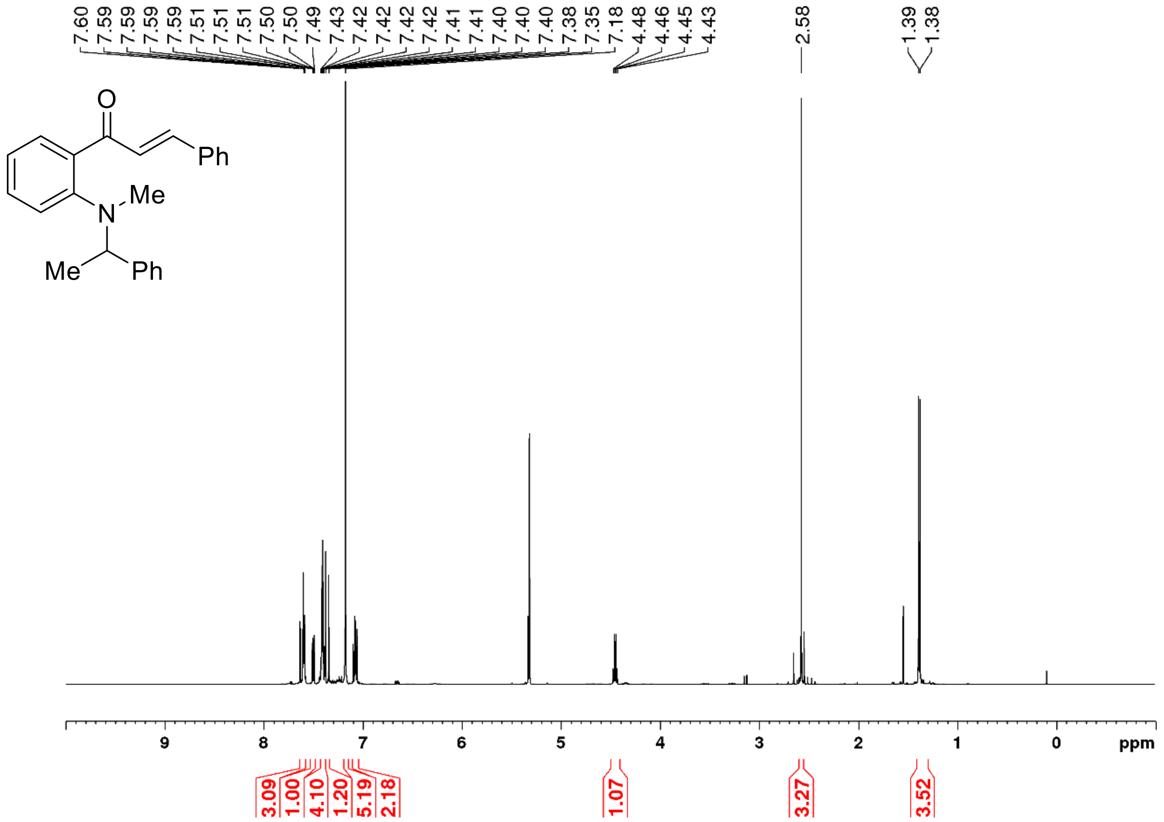

^{13}**C-NMR** (176 MHz, 298 K, $CDCl_3$)

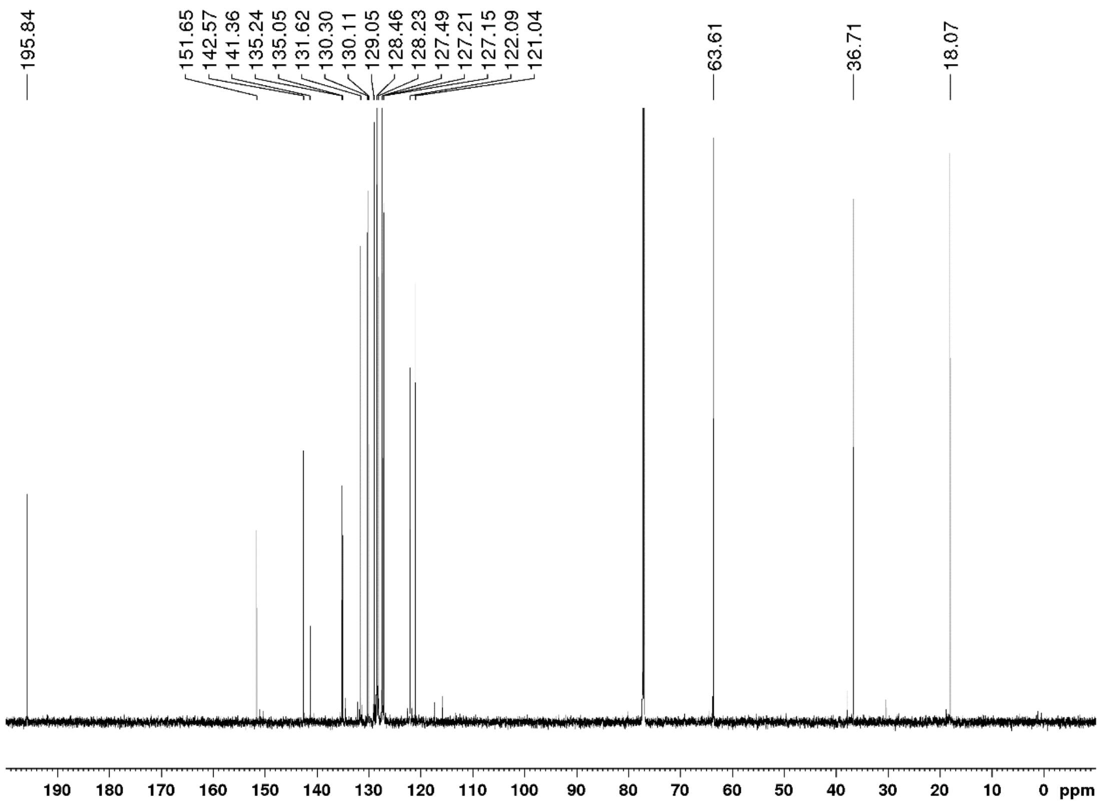

^{1}H-NMR (700 MHz, 298 K, CD_2Cl_2) (d_1-*rac*-**1a**)

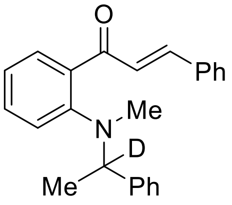

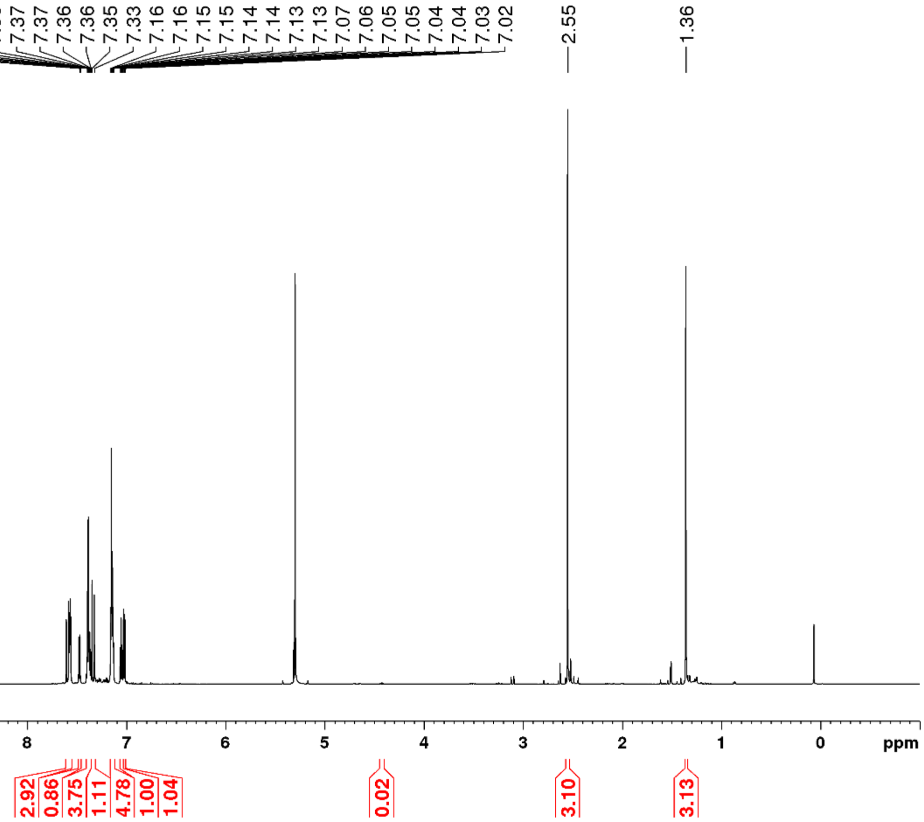

^{13}C-NMR (176 MHz, 298 K, CD_2Cl_2)

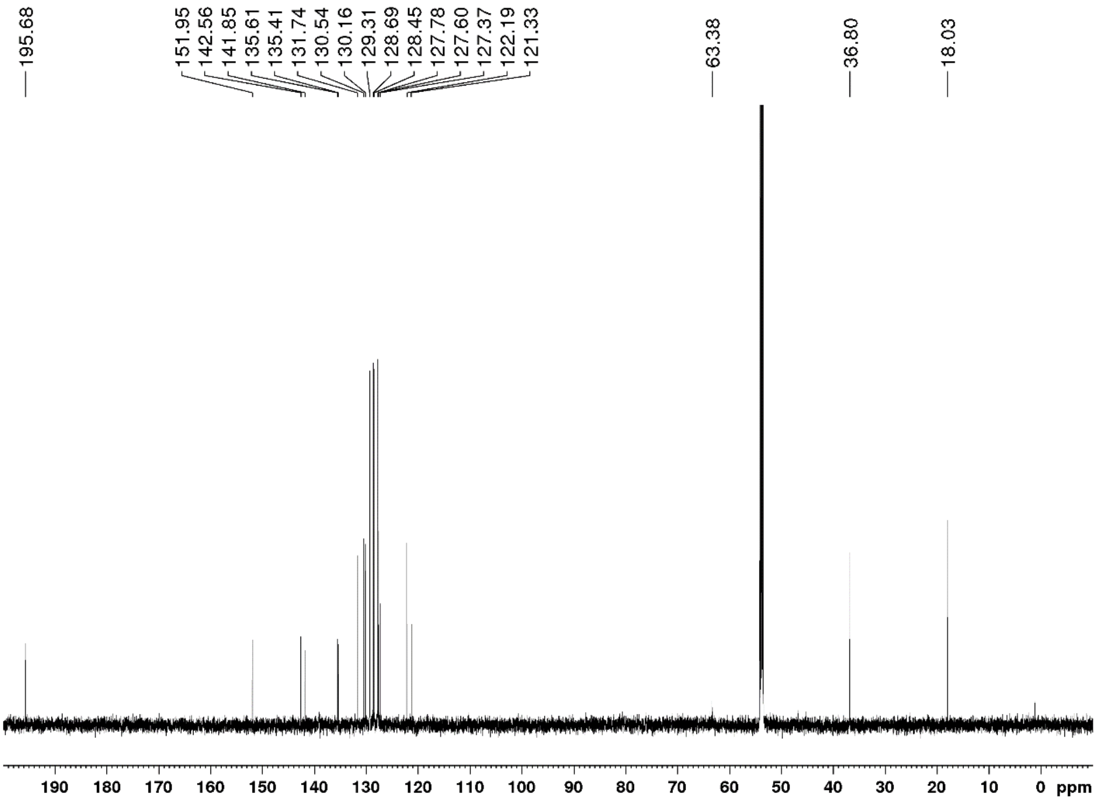

^{1}H-NMR (700 MHz, 298 K, CD_2Cl_2) ((*S*)-**1a**)

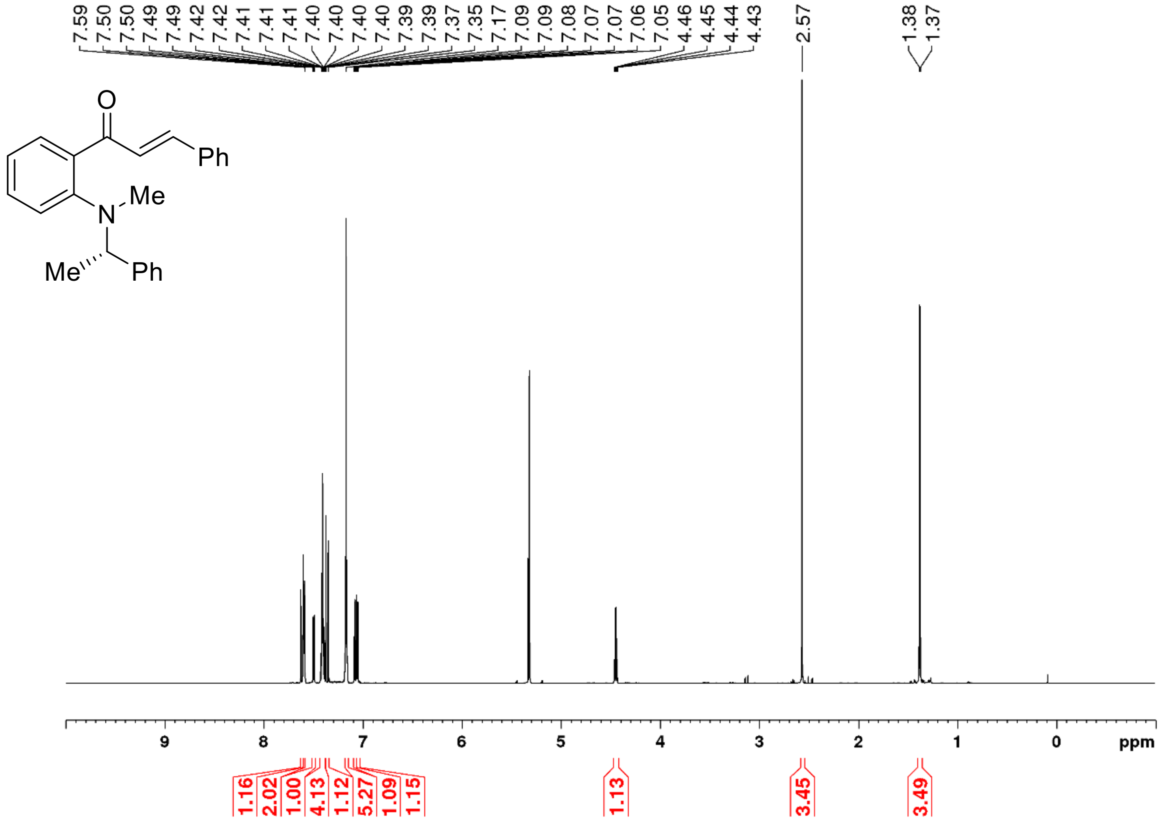

^{13}C-NMR (176 MHz, 298 K, CD_2Cl_2)

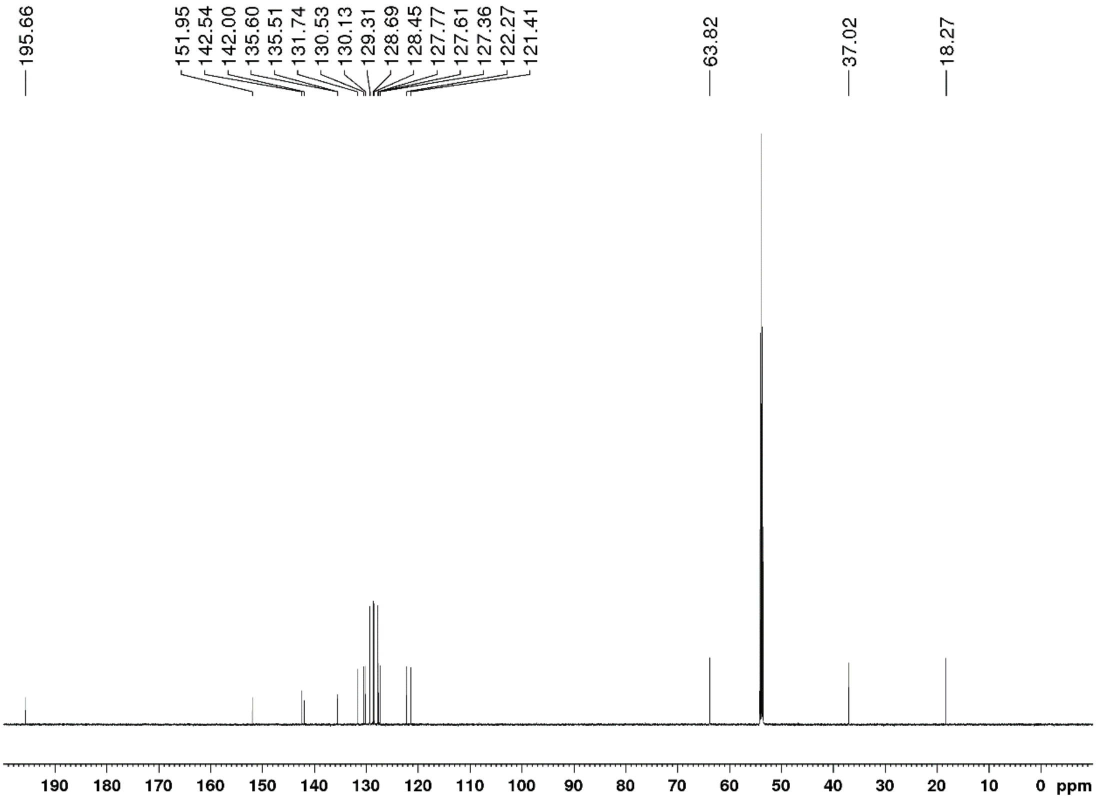

^{1}**H-NMR** (700 MHz, 298 K, $CDCl_3$) (**1b**)

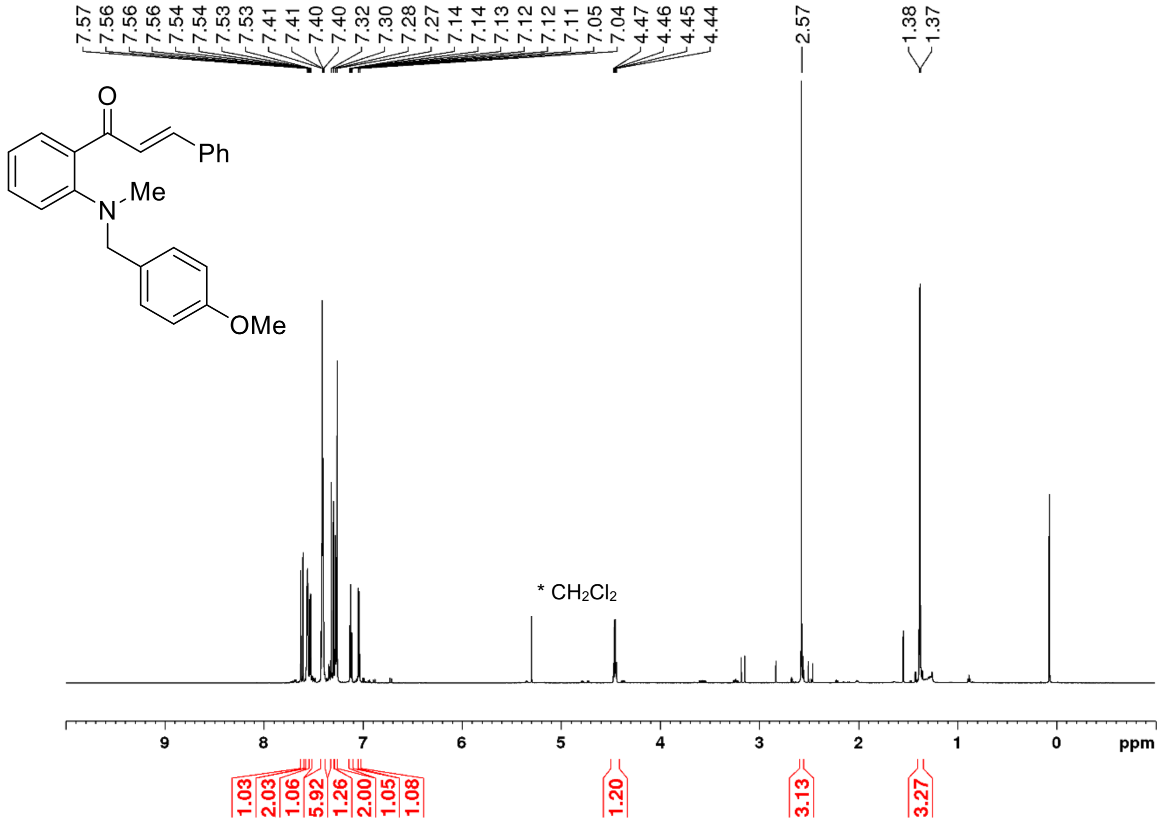

^{13}**C-NMR** (176 MHz, 298 K, $CDCl_3$)

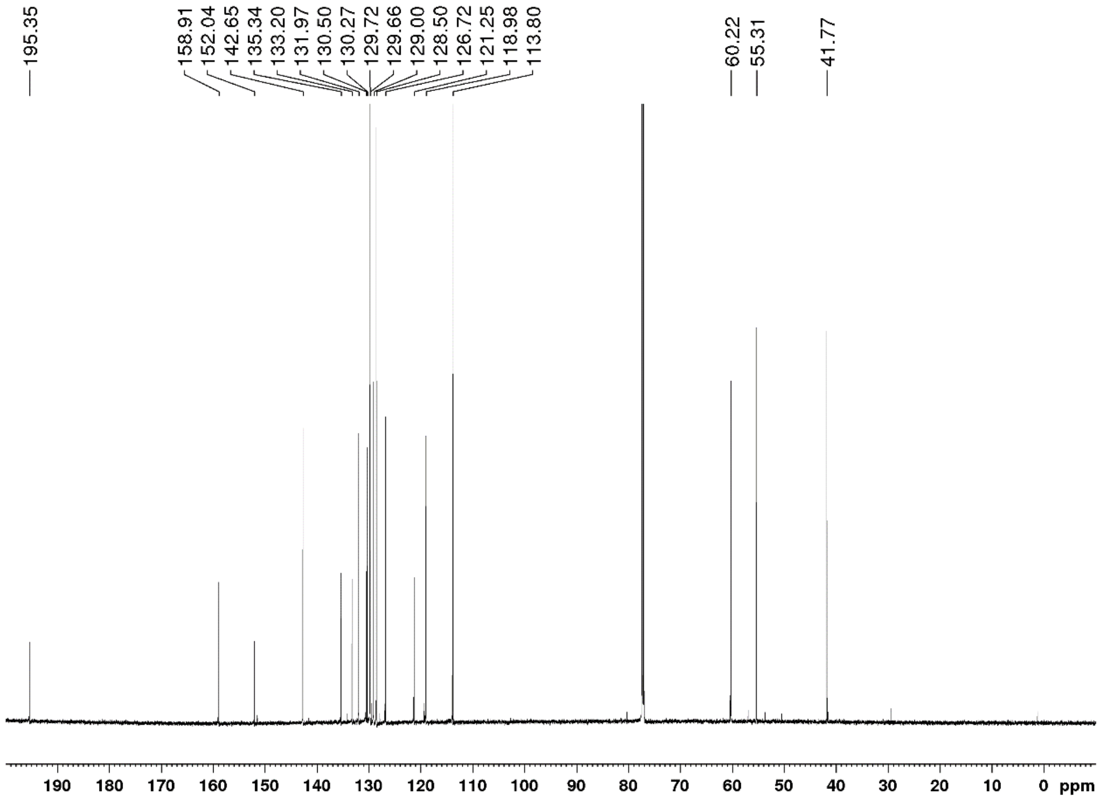

[1]**H-NMR** (700 MHz, 298 K, $CDCl_3$) (**1c**)

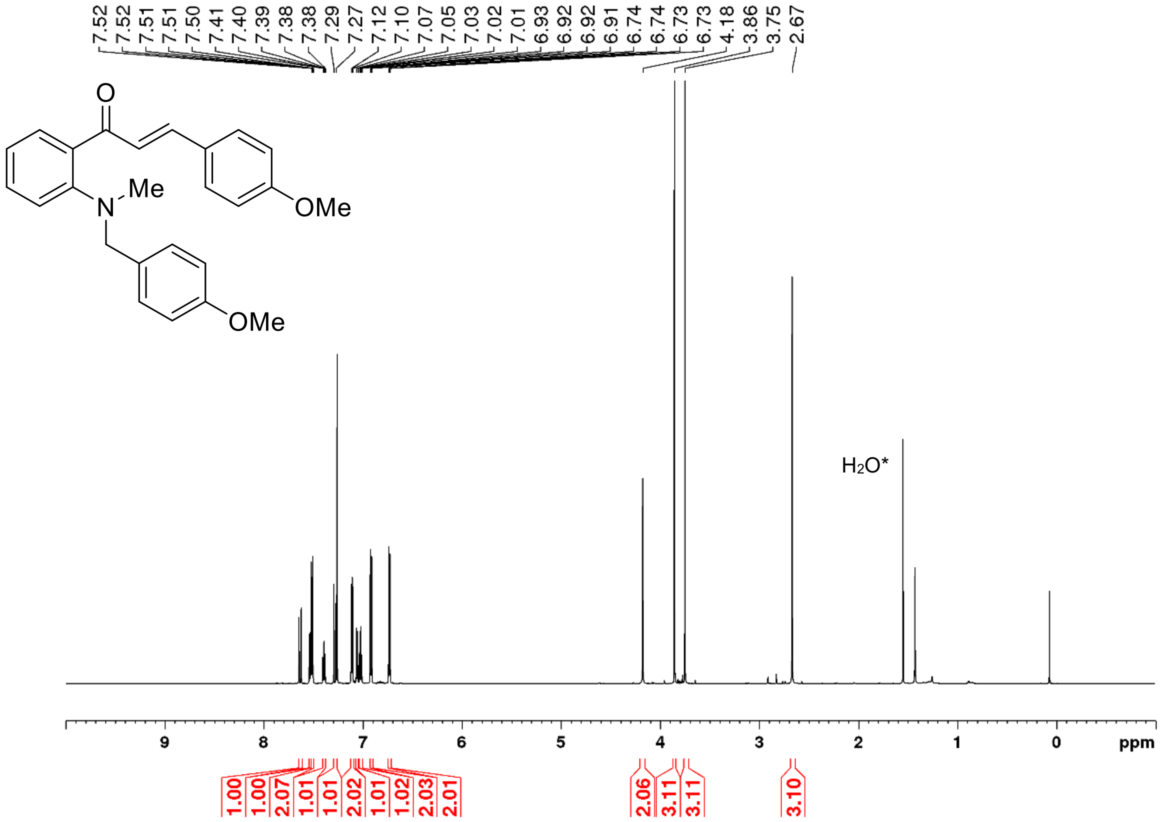

[13]**C-NMR** (176 MHz, 298 K, $CDCl_3$)

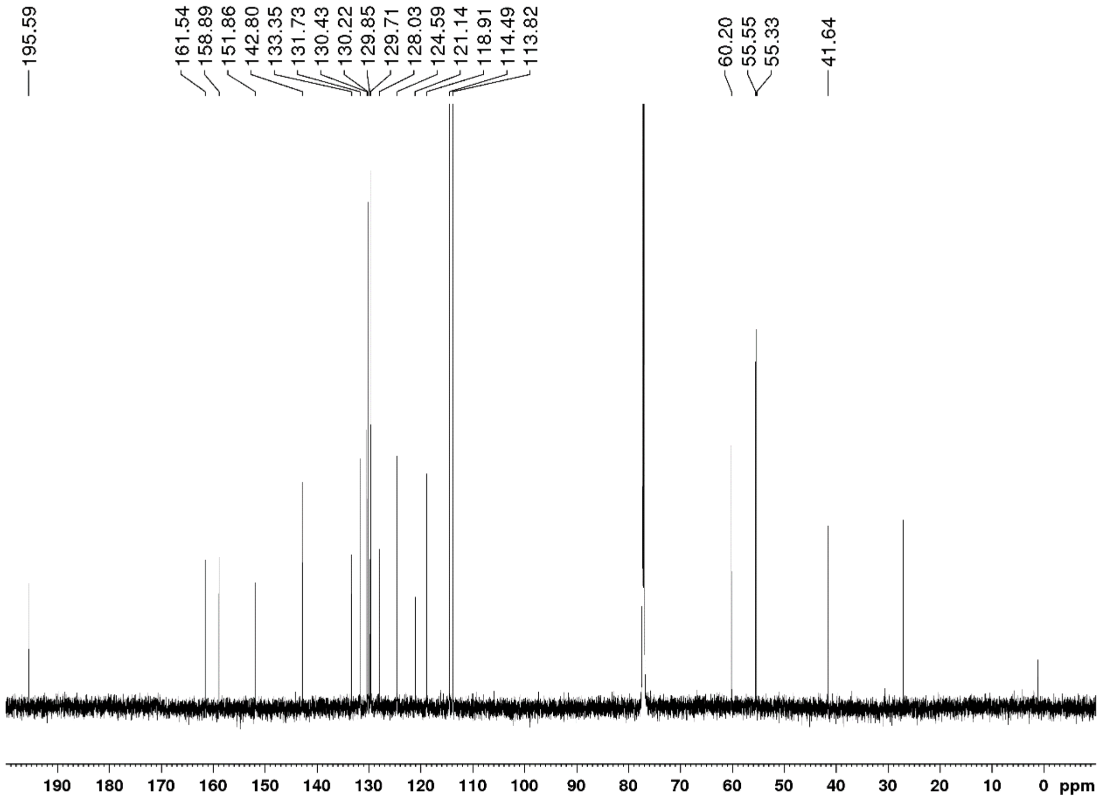

^{1}H-NMR (700 MHz, 298 K, $CDCl_3$) (**1d**)

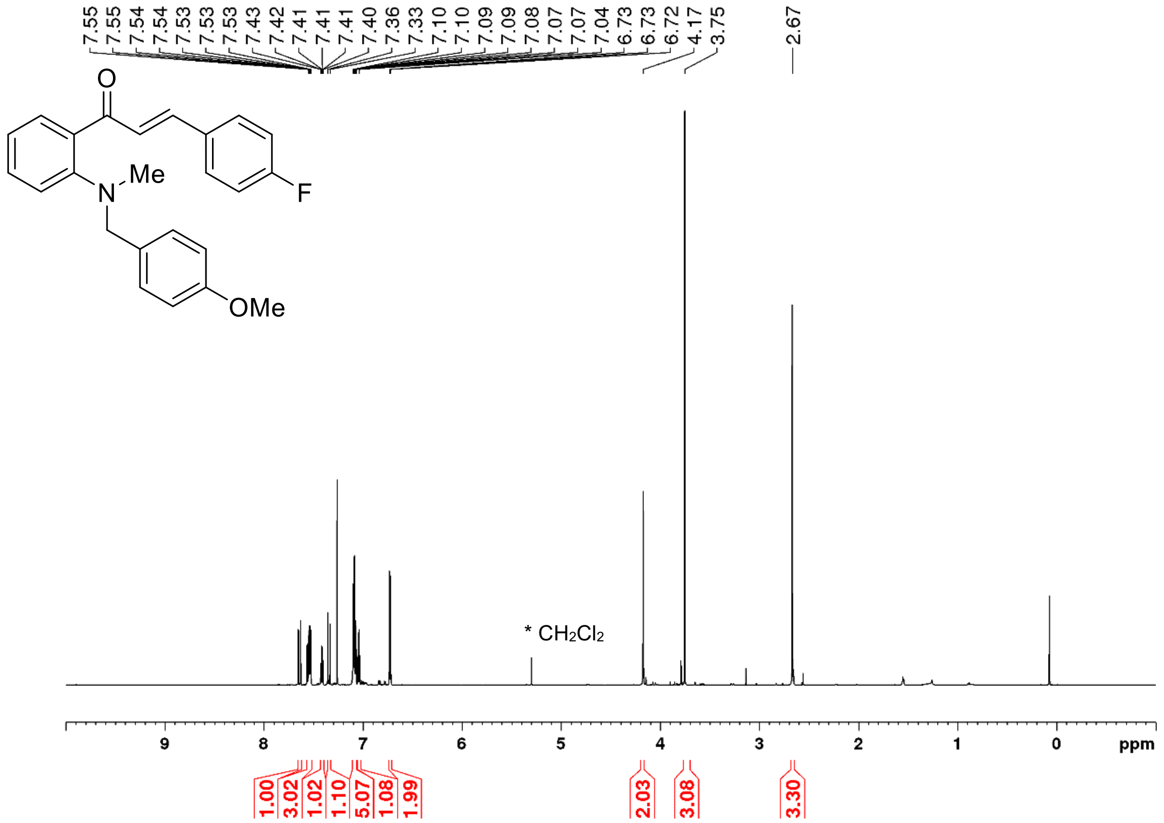

^{13}C-NMR (176 MHz, 298 K, $CDCl_3$)

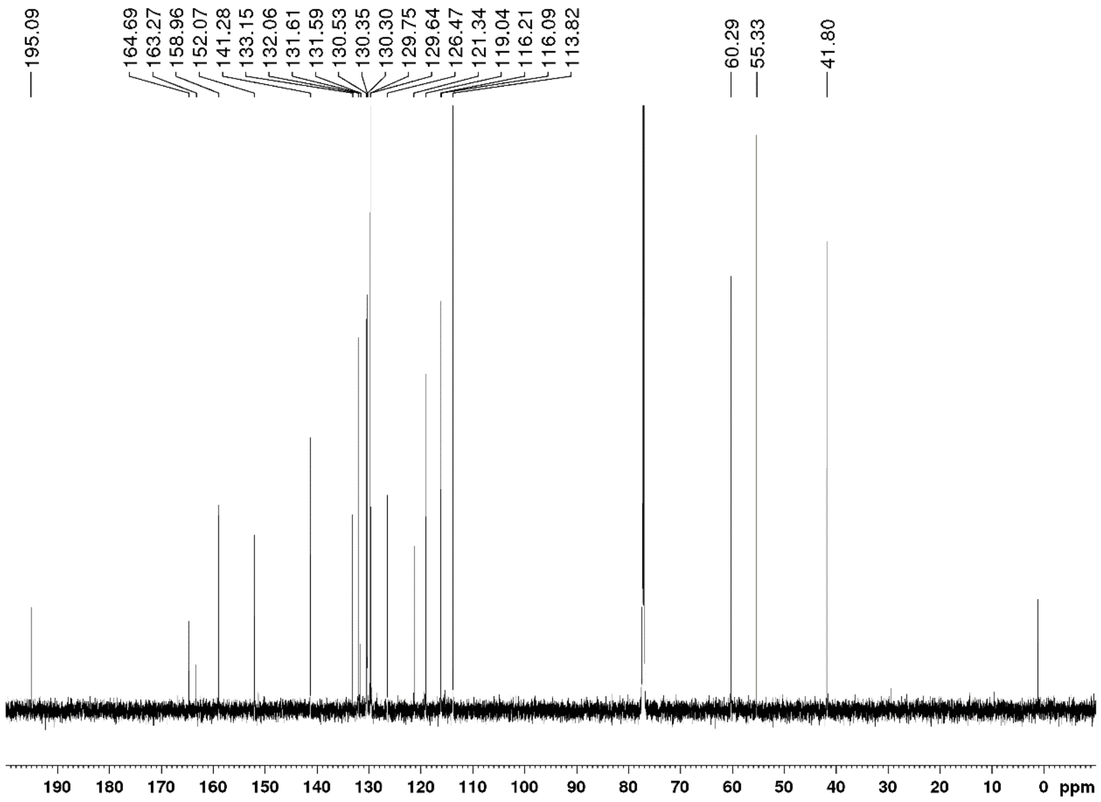

^{1}H-NMR (700 MHz, 298 K, $CDCl_3$) (**1e**)

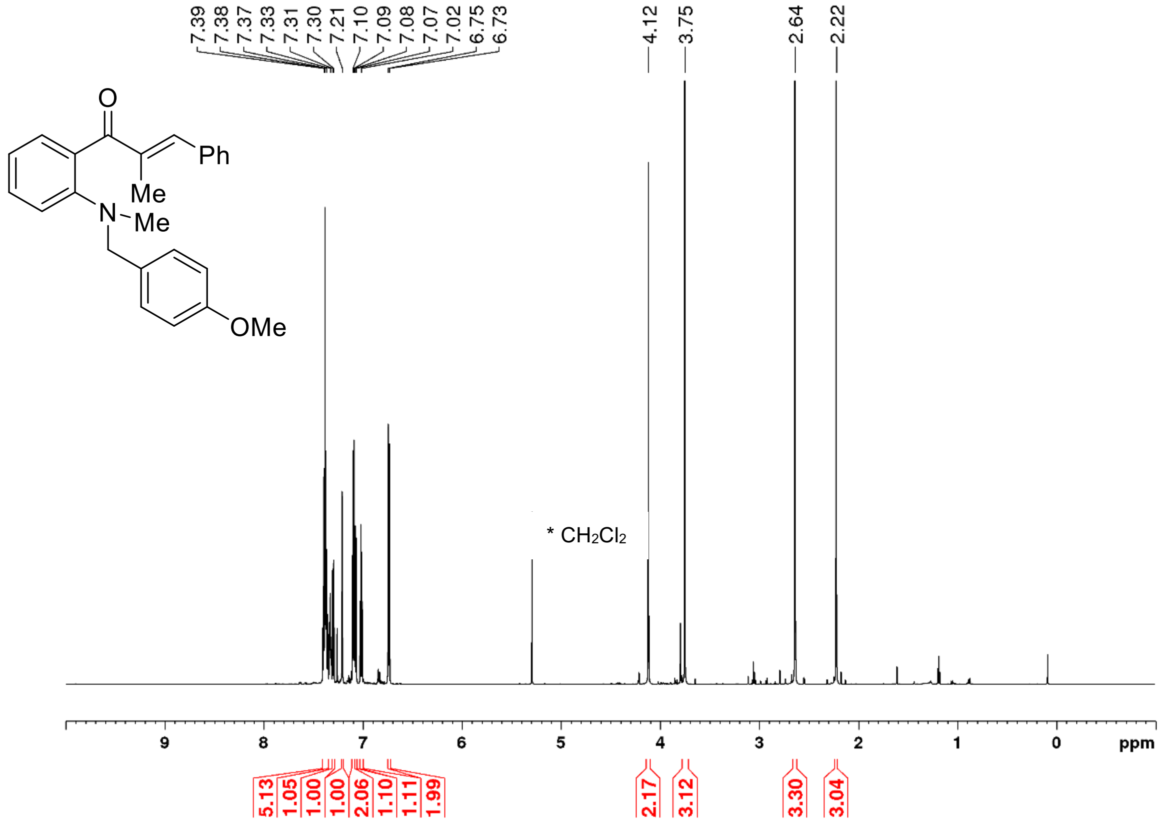

^{13}C-NMR (176 MHz, 298 K, $CDCl_3$)

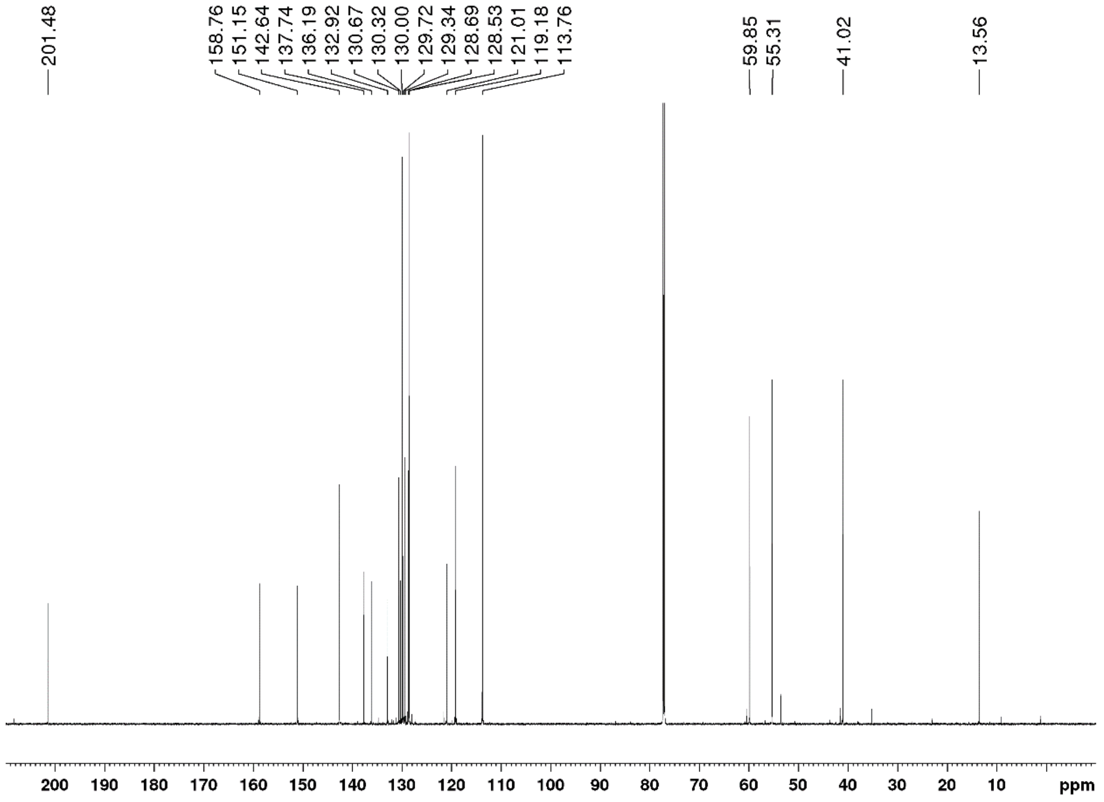

^{1}H-NMR (700 MHz, 298 K, $CDCl_3$) (**1f**)

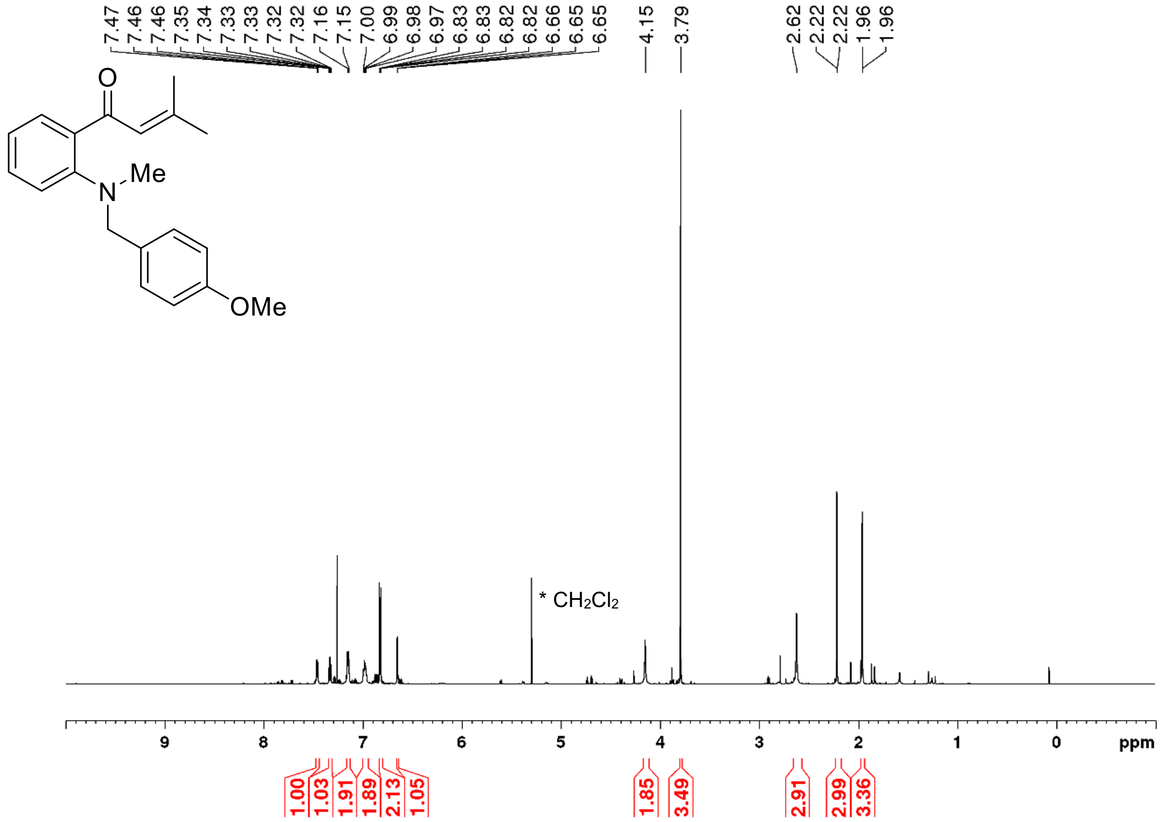

^{13}C-NMR (176 MHz, 298 K, $CDCl_3$)

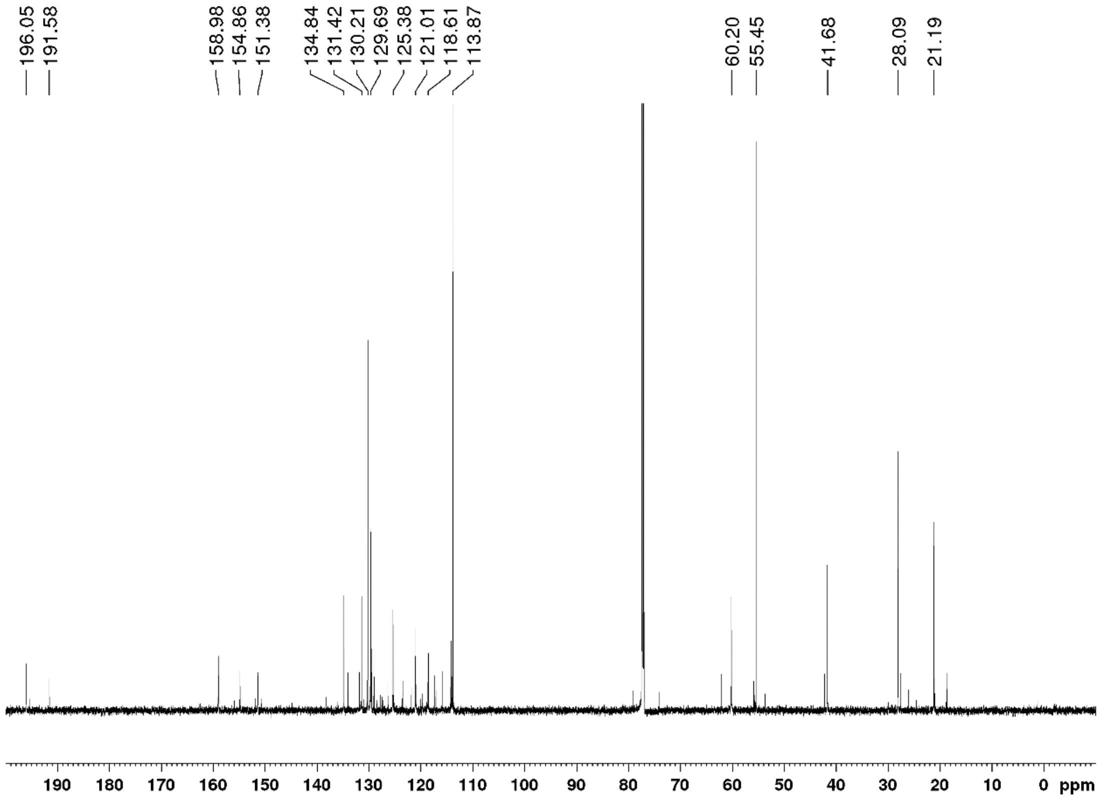

^{1}H-NMR (700 MHz, 273 K, $CDCl_3$) (**1g**)

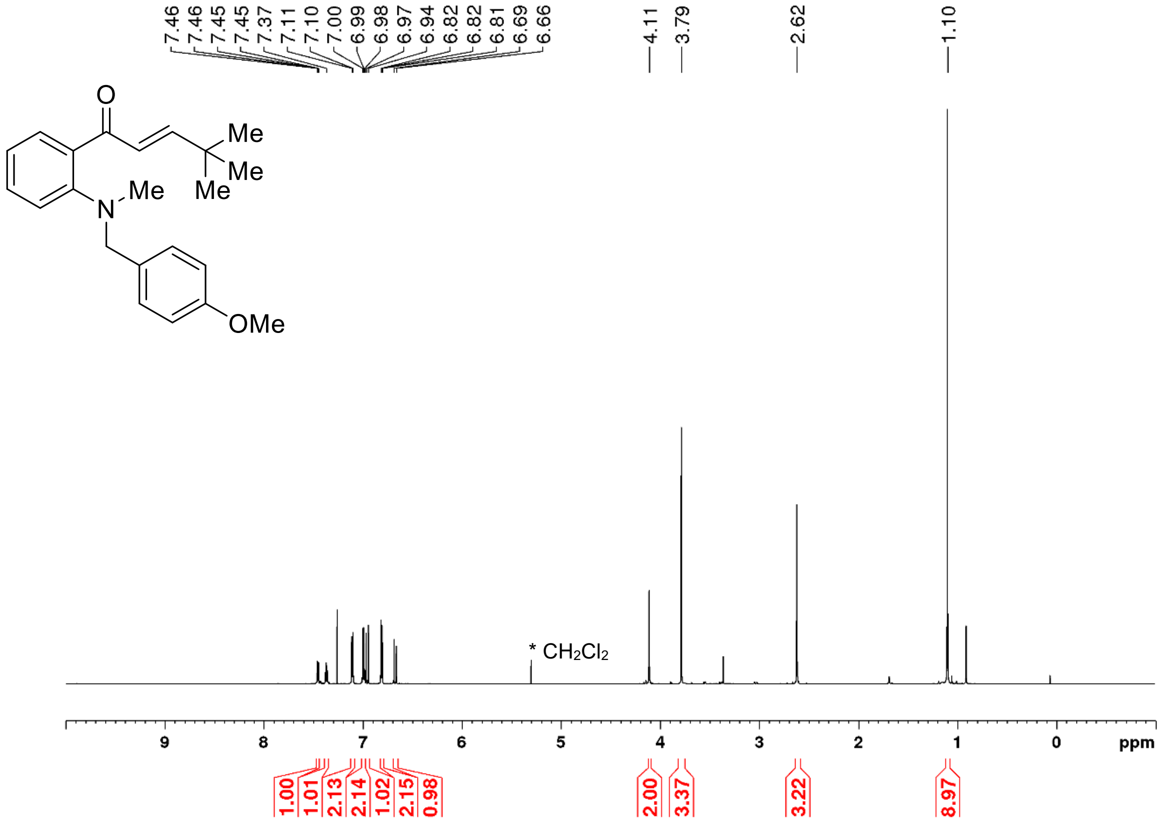

^{13}C-NMR (176 MHz, 273 K, $CDCl_3$)

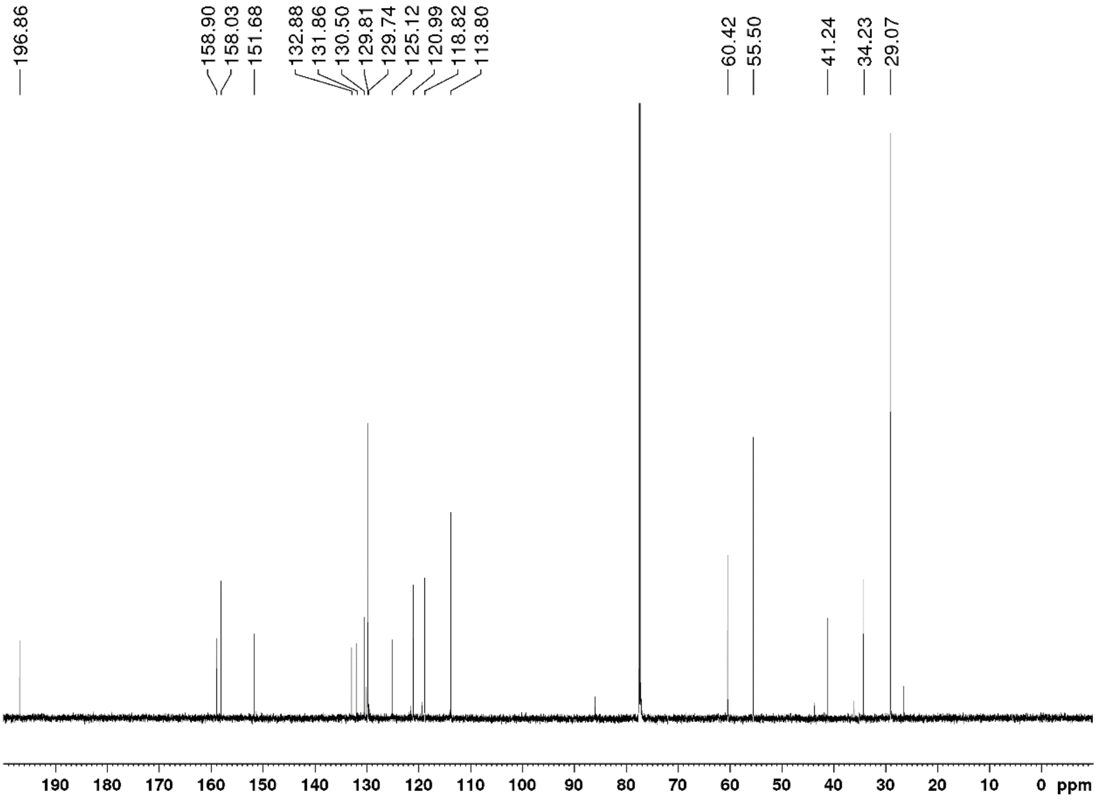

^{1}H-NMR (700 MHz, 298 K, $CDCl_3$) (**1h**)

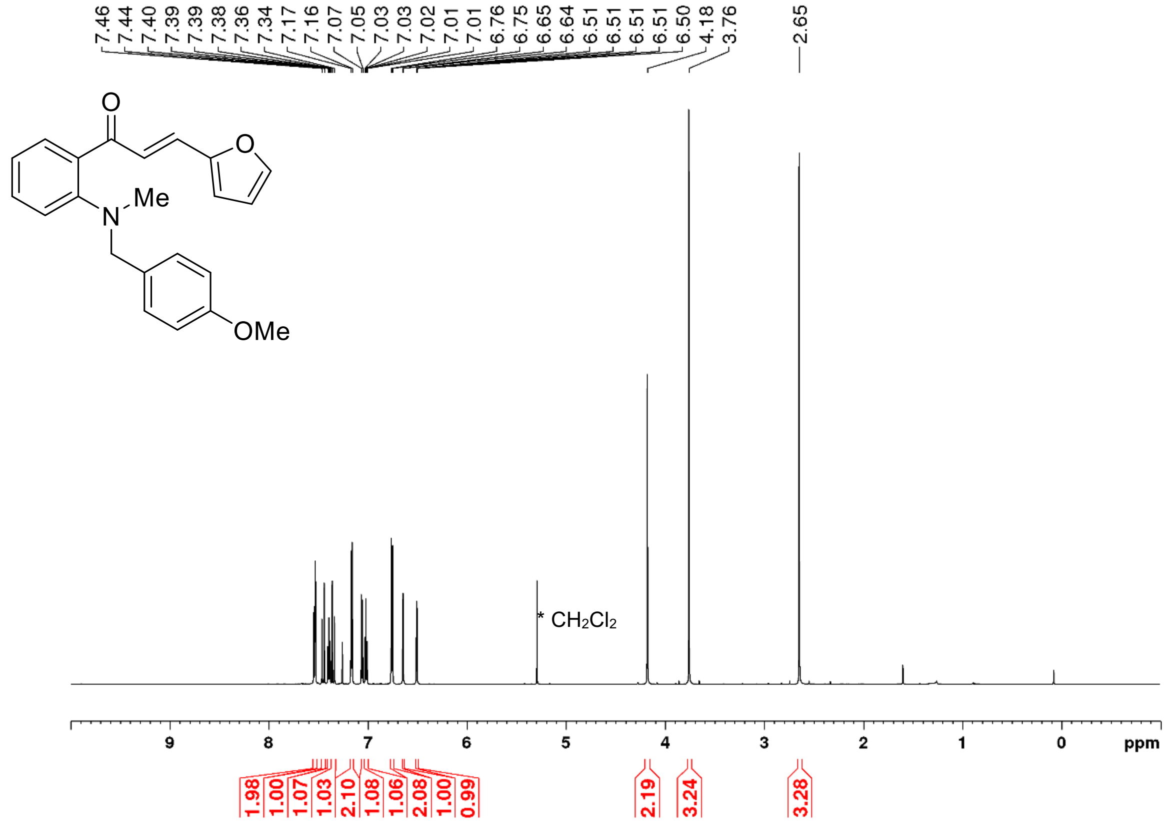

^{13}C-NMR (176 MHz, 298 K, $CDCl_3$)

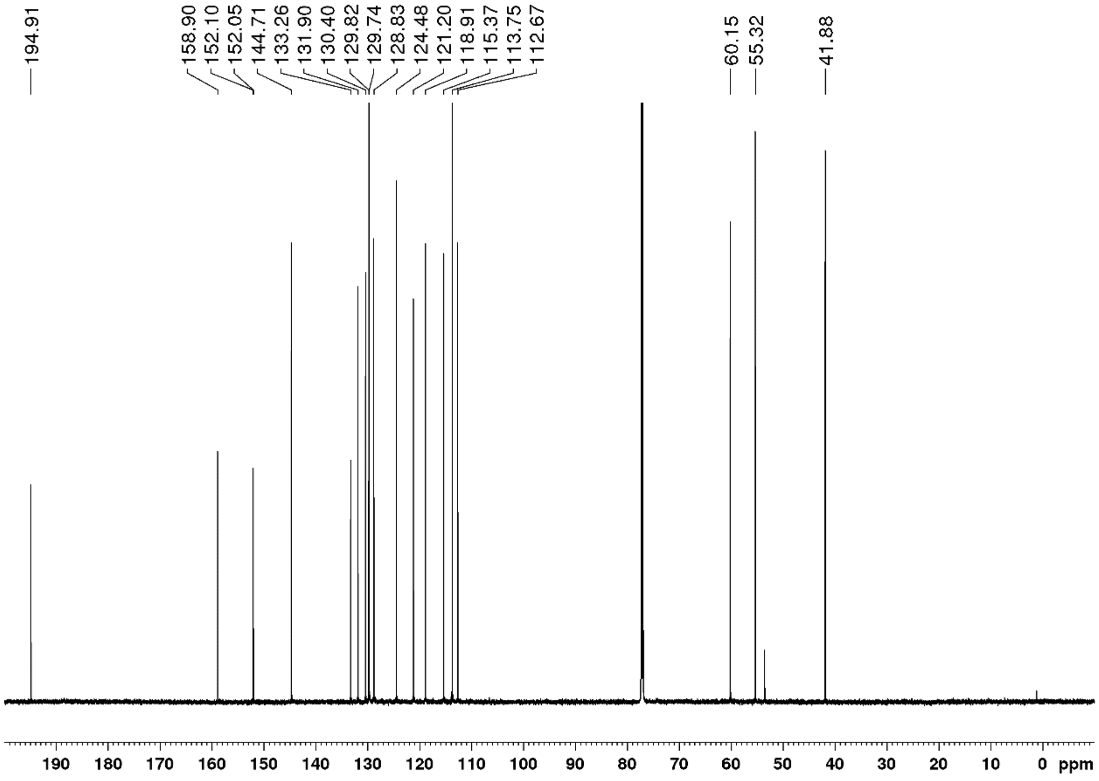

^{1}H-NMR (700 MHz, 298 K, $CDCl_3$) (**1i**)

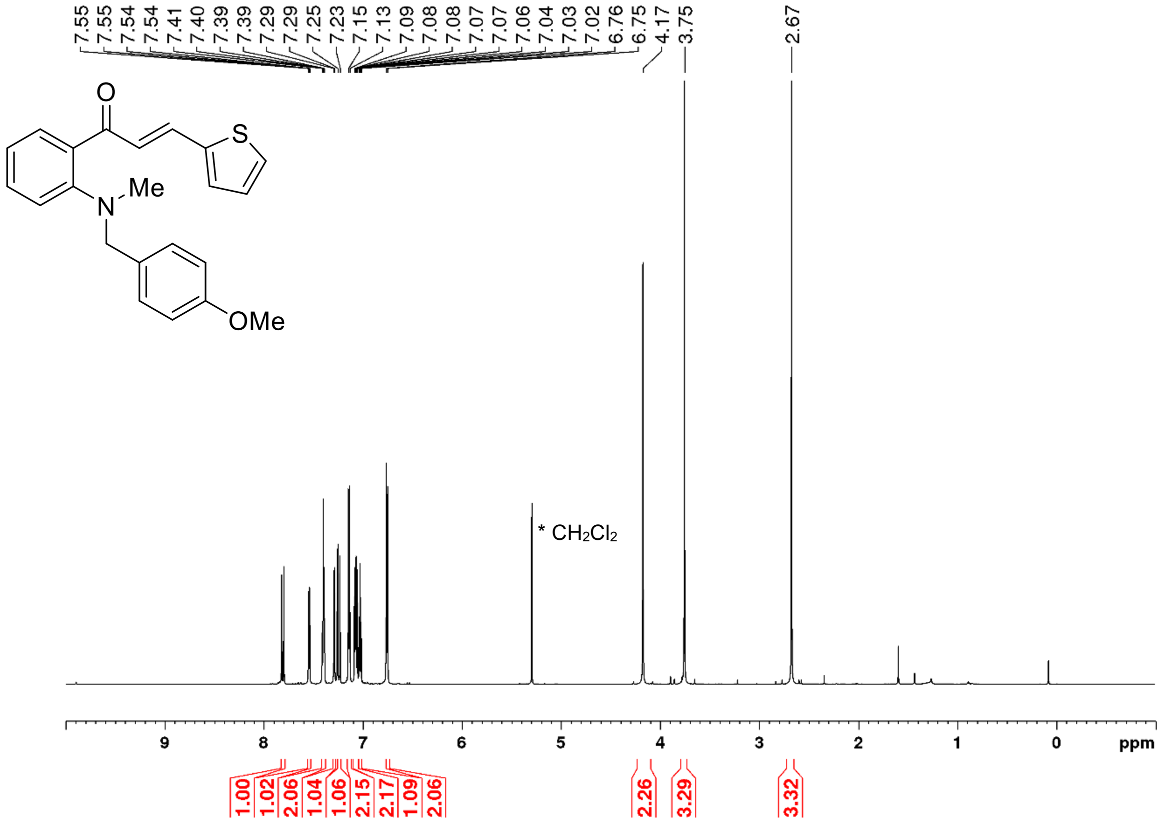

^{13}C-NMR (176 MHz, 298 K, $CDCl_3$)

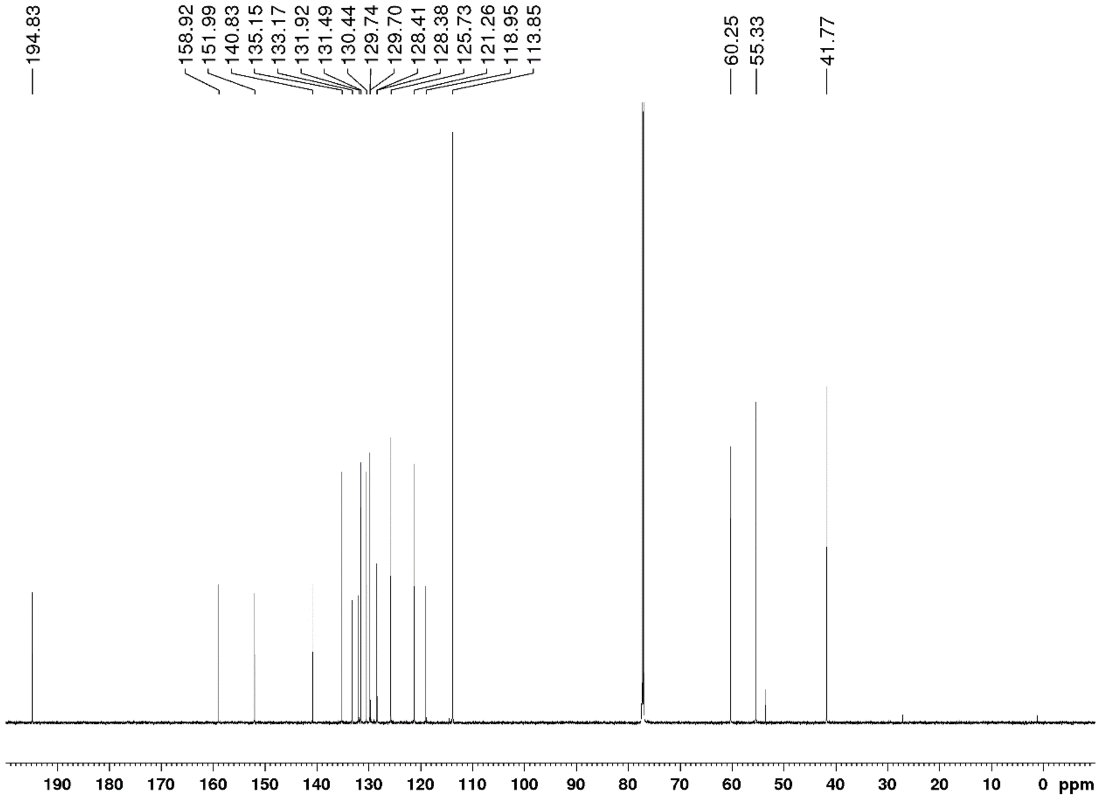

^{1}H-NMR (700 MHz, 298 K, $CDCl_3$) (**1j**)

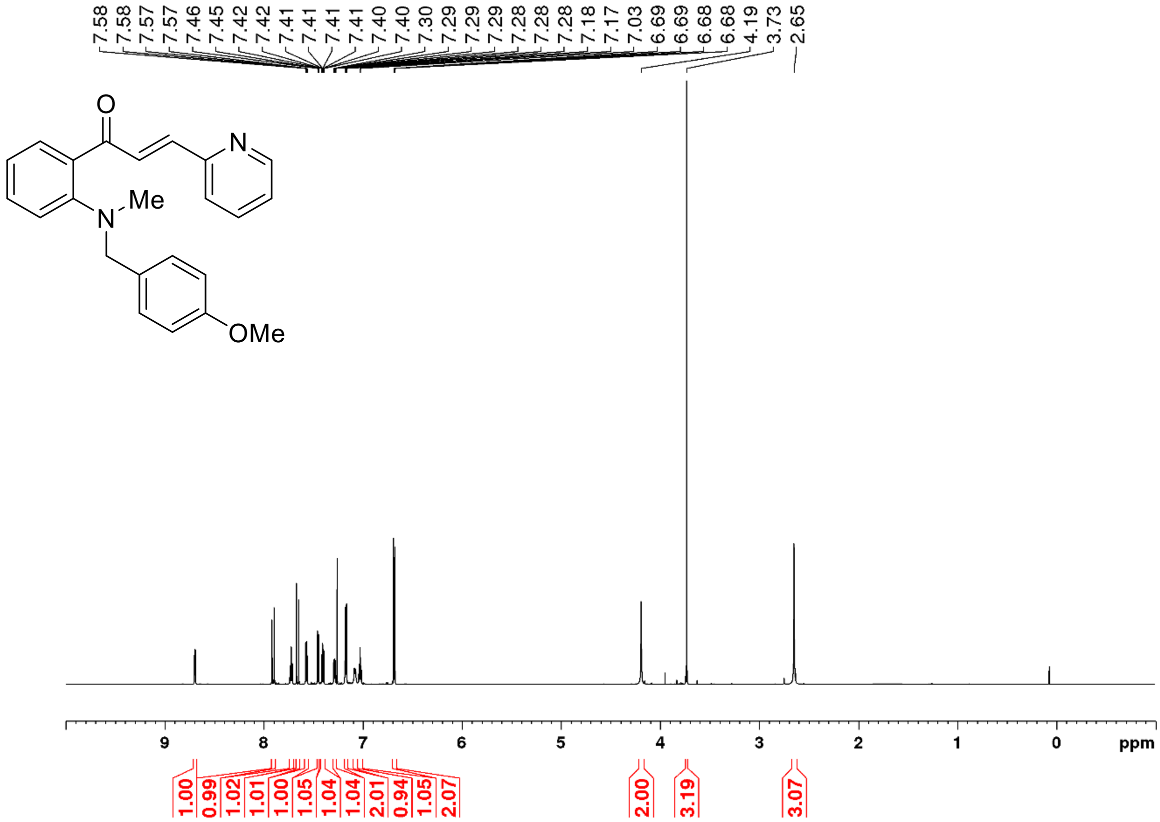

^{13}C-NMR (176 MHz, 298 K, $CDCl_3$)

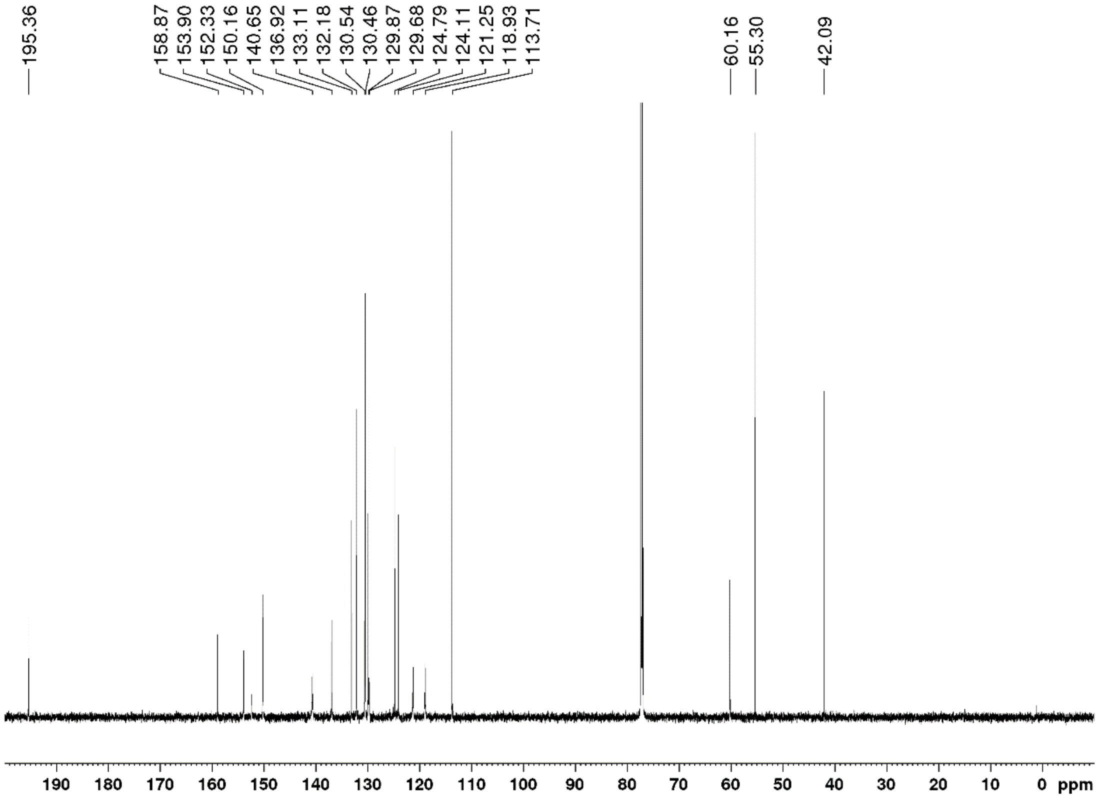

[1]**H-NMR** (700 MHz, 298 K, CDCl3) (**1k**)

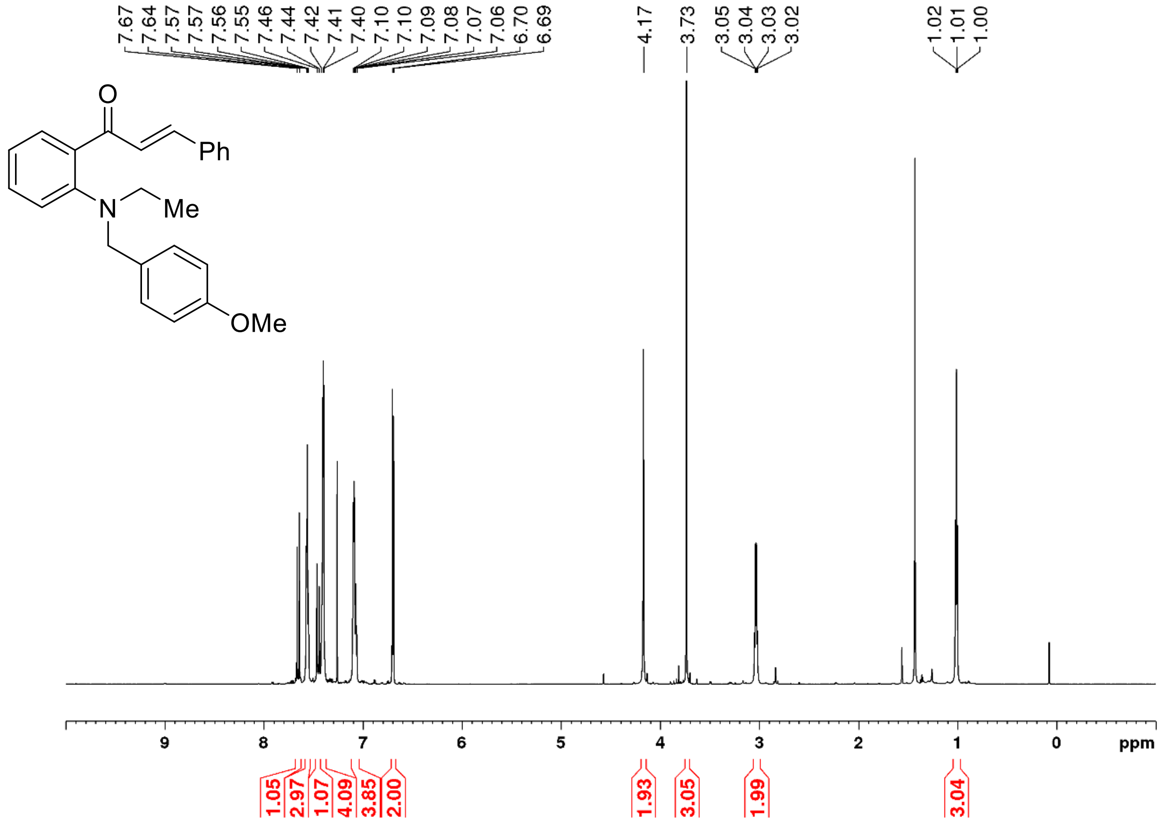

[13]**C-NMR** (176 MHz, 298 K, CDCl3)

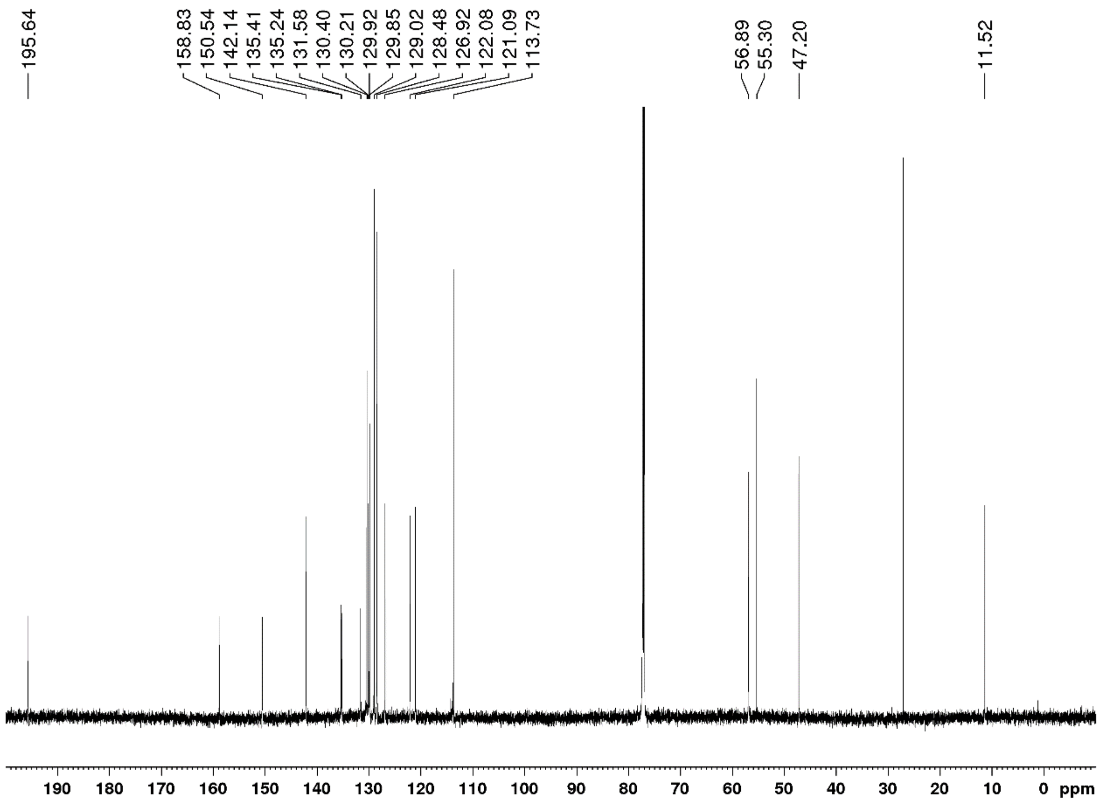

^{1}H-NMR (700 MHz, 298 K, $CDCl_3$) (**1l**)

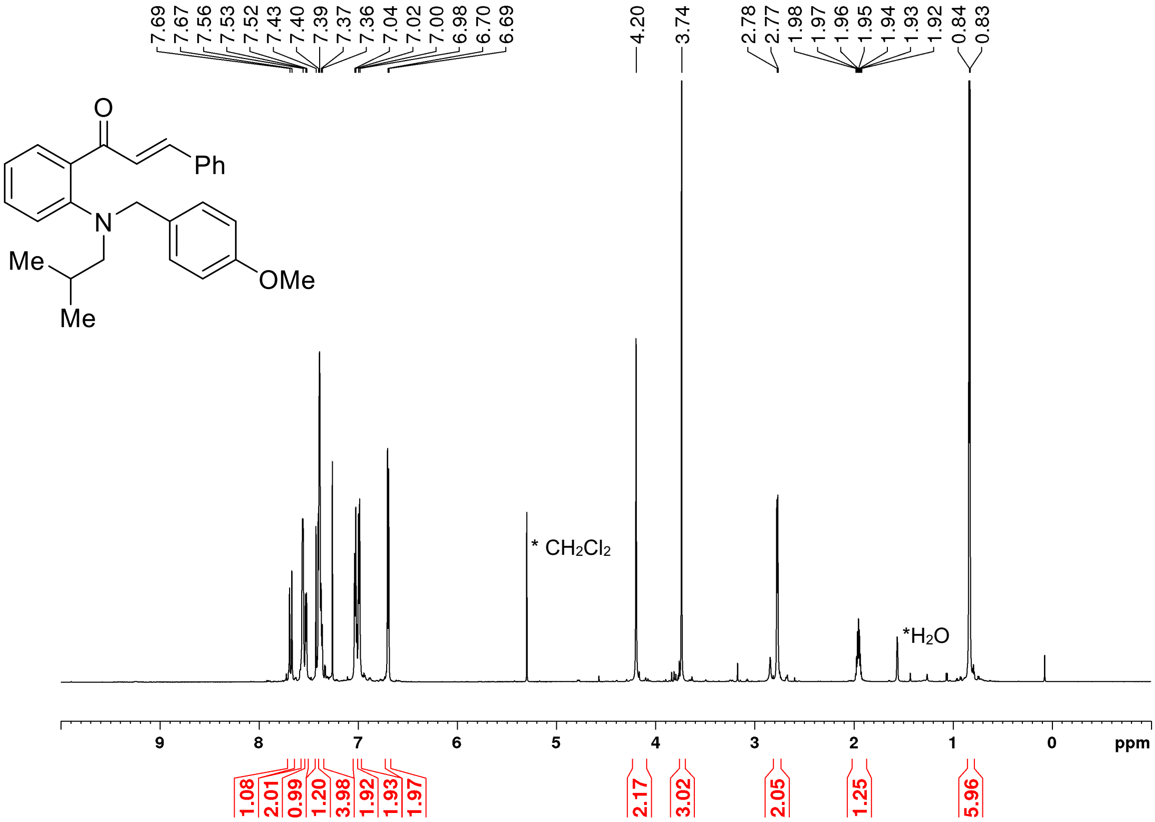

^{13}C-NMR (176 MHz, 298 K, $CDCl_3$)

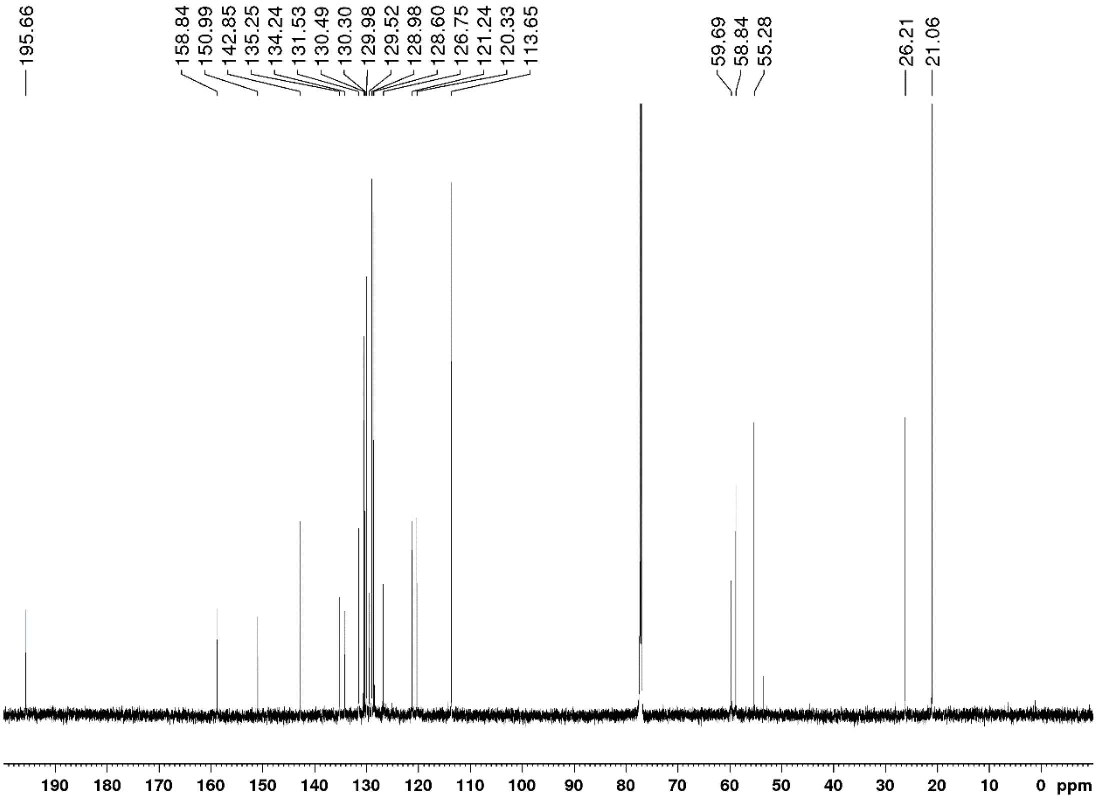

^{1}H-NMR (700 MHz, 298 K, $CDCl_3$) (*rac*-**1m**)

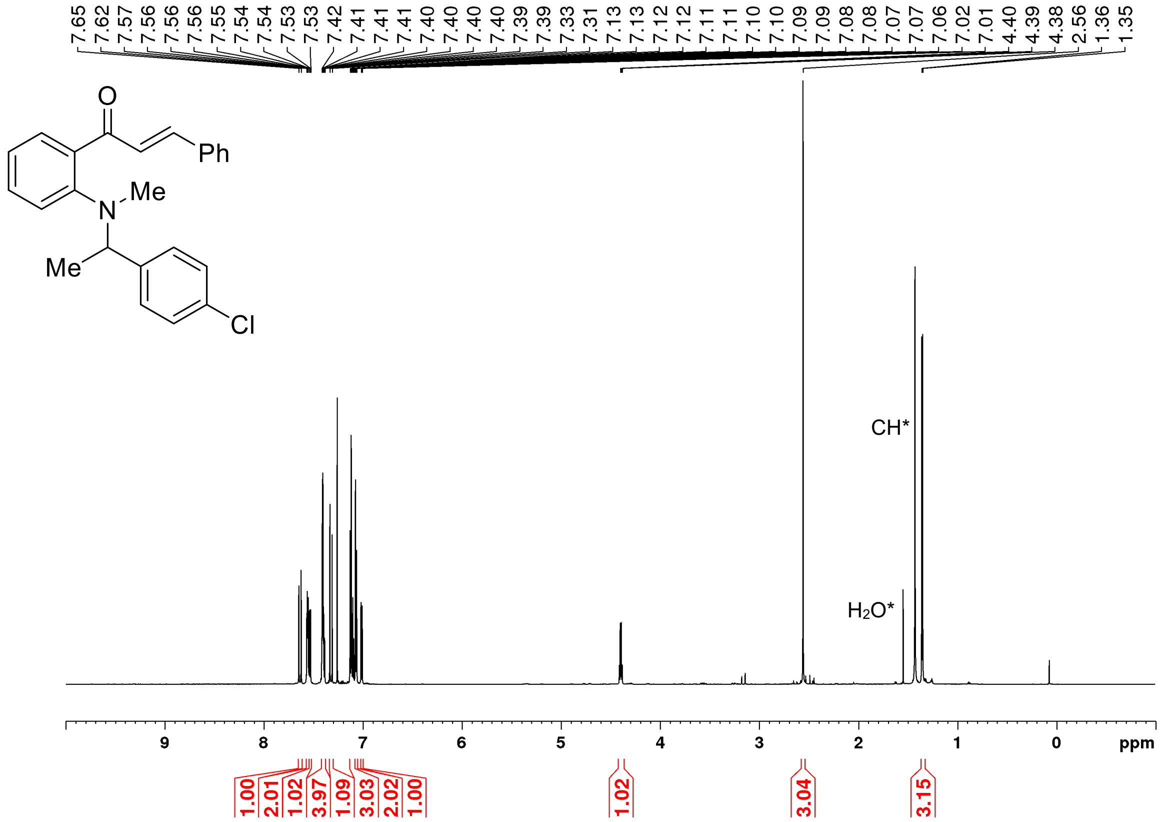

^{13}C-NMR (176 MHz, 298 K, $CDCl_3$)

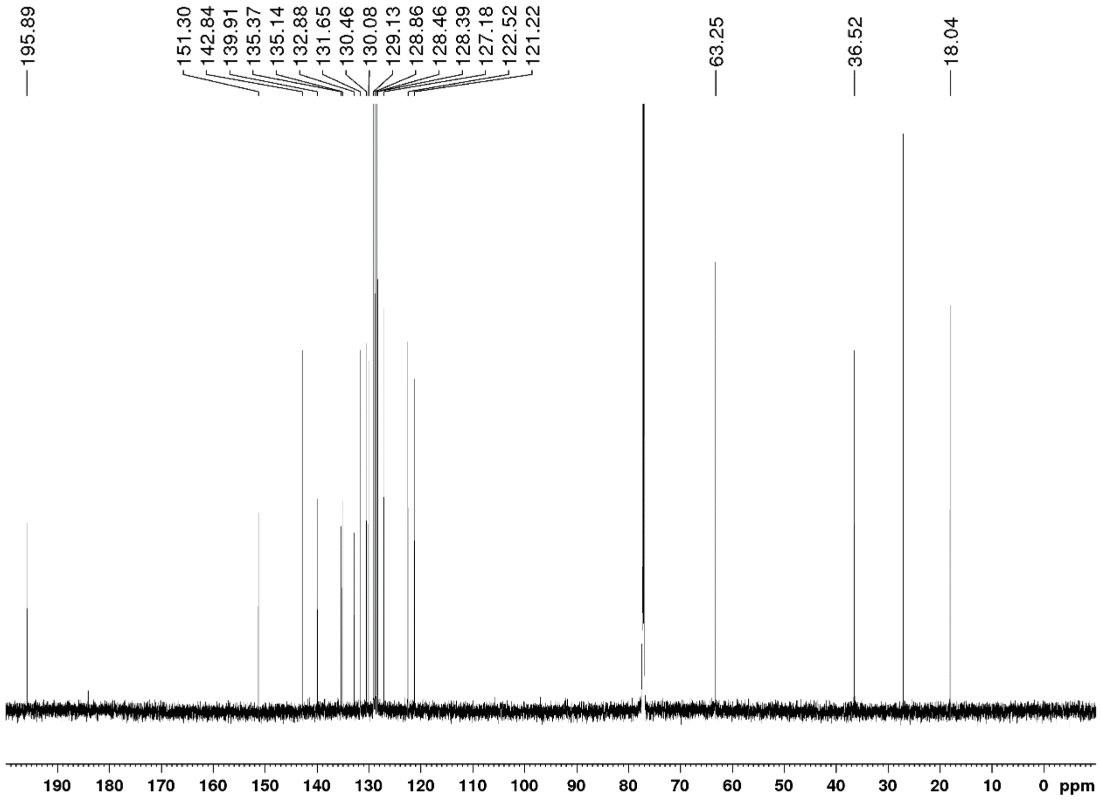

^{1}H-NMR (700 MHz, 298 K, $CDCl_3$) (*rac*-**1n**)

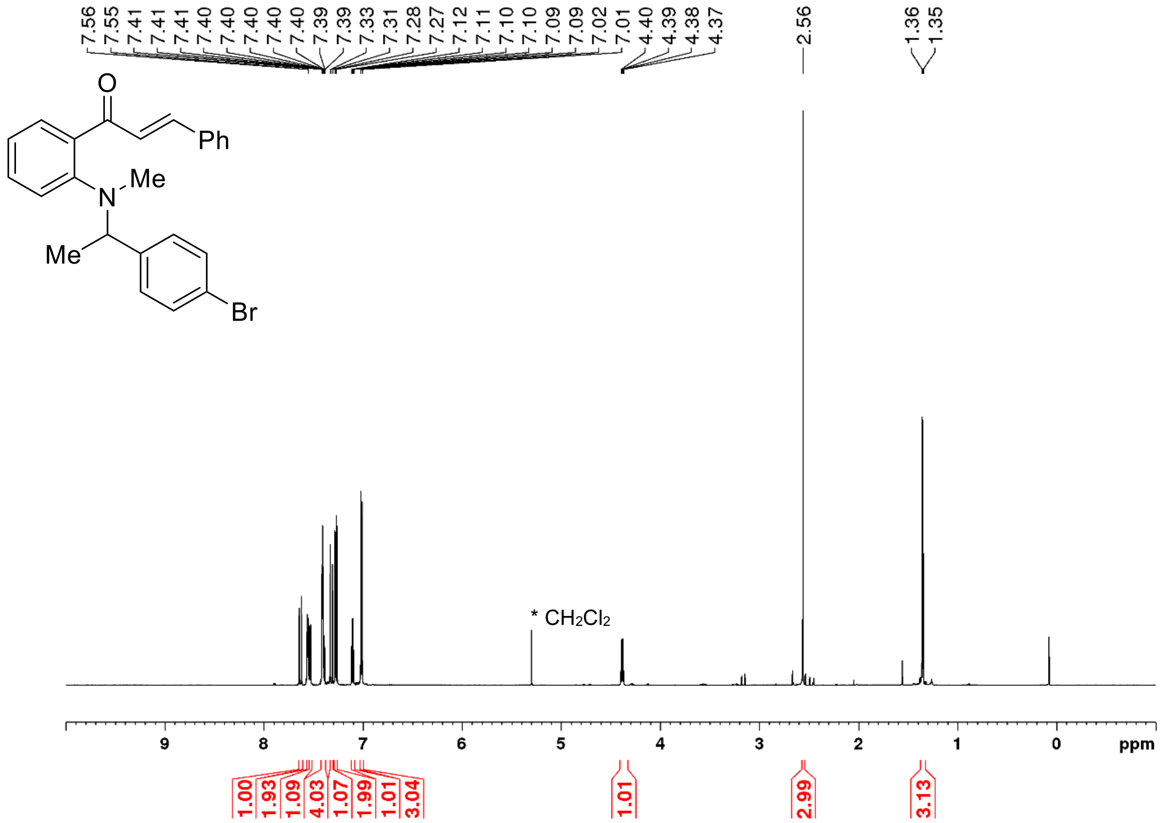

^{13}C-NMR (176 MHz, 298 K, $CDCl_3$)

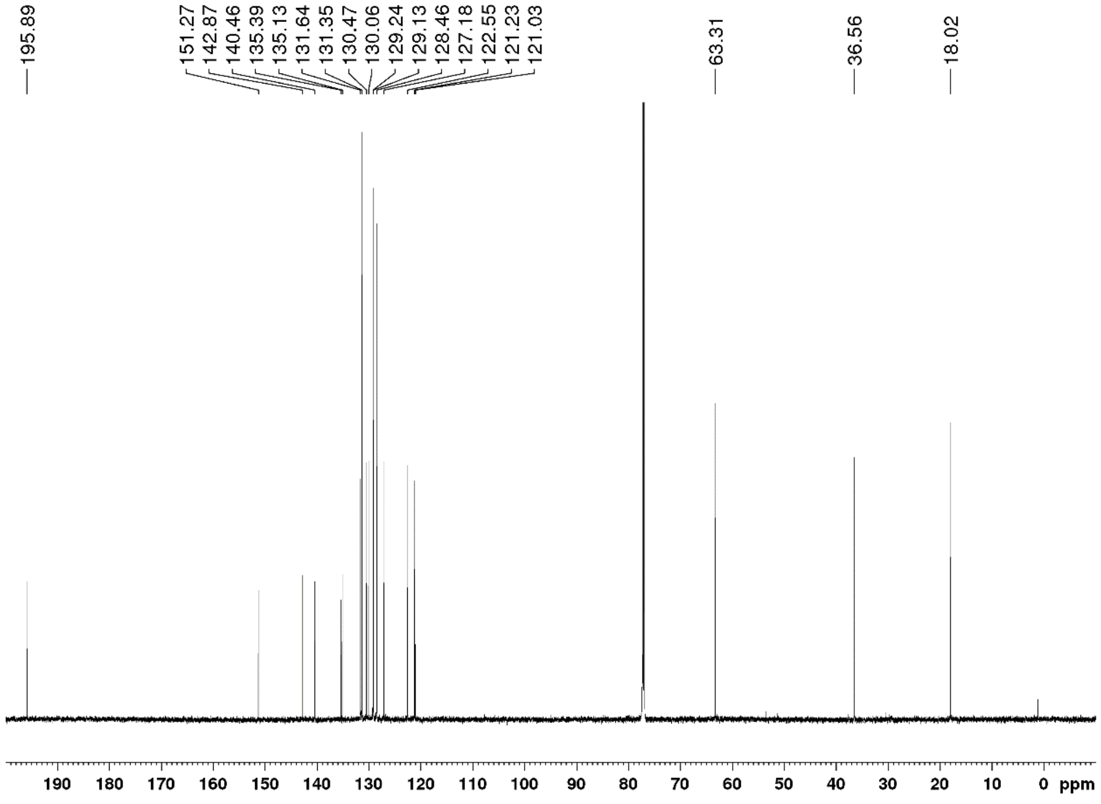

^{1}H-NMR (700 MHz, 298 K, $CDCl_3$) ((*S*)-**1n**)

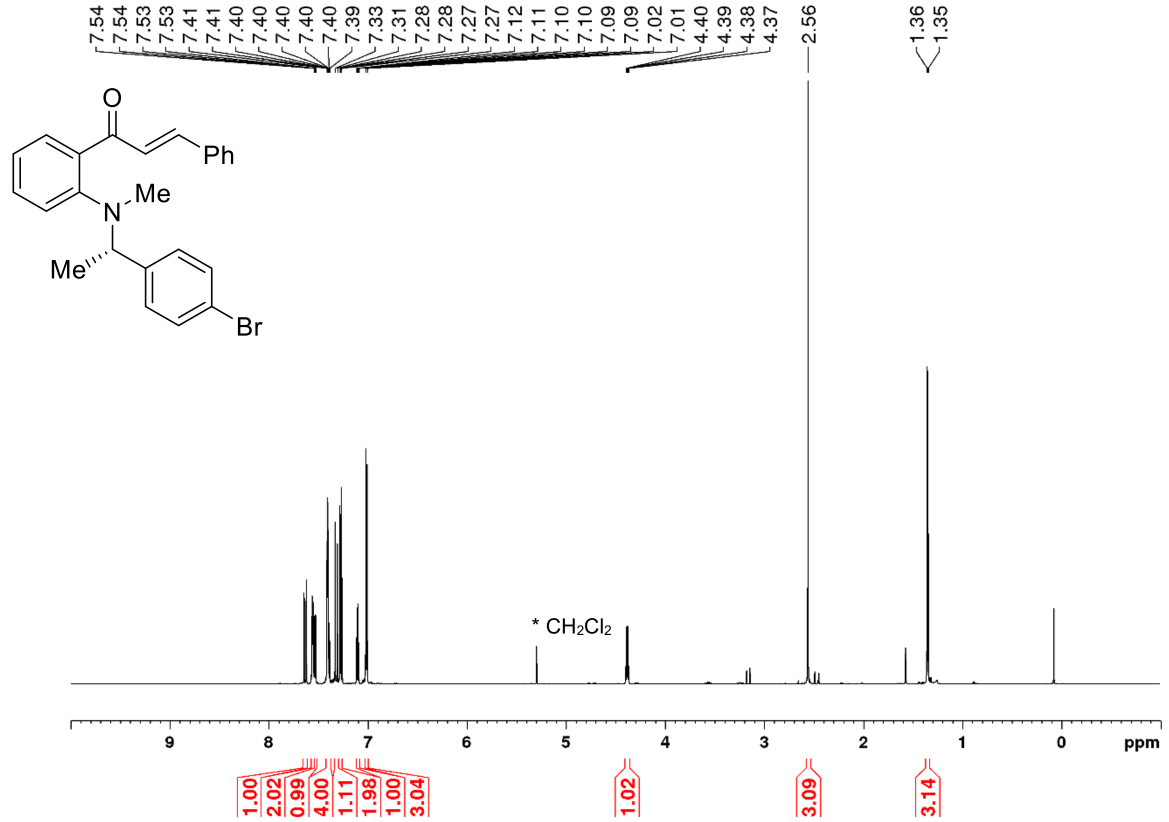

^{13}C-NMR (176 MHz, 298 K, $CDCl_3$)

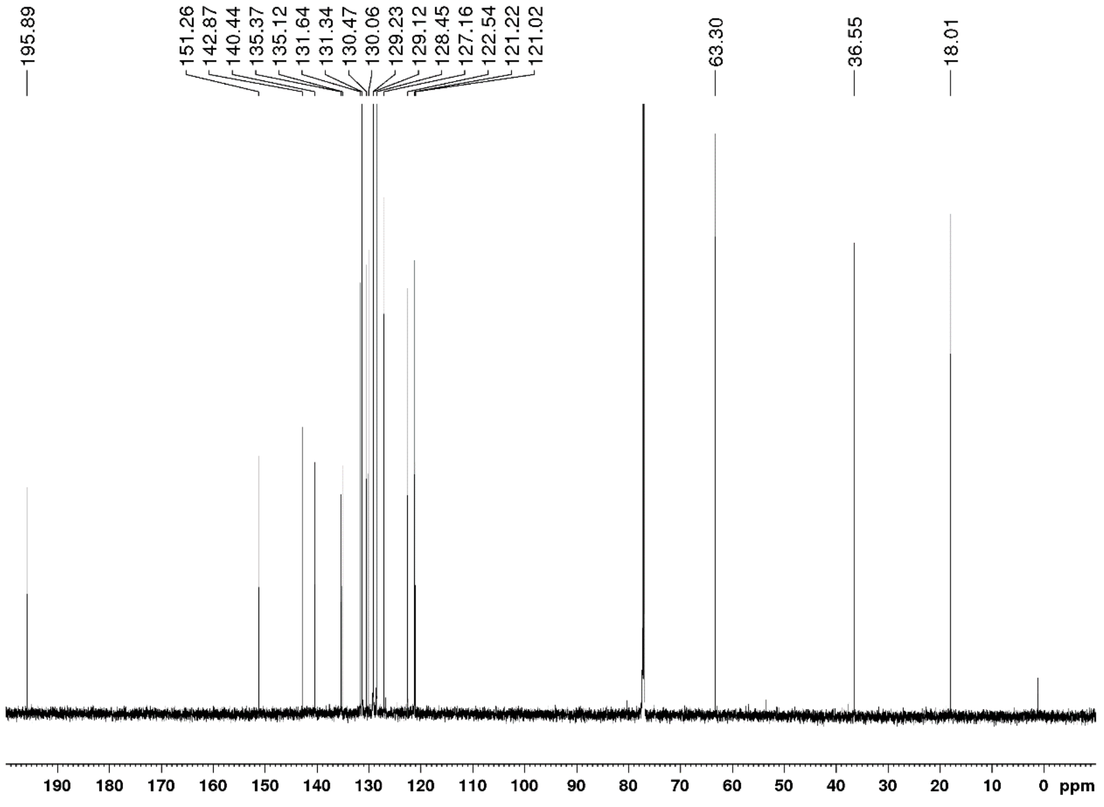

^{1}H-NMR (700 MHz, 298 K, $CDCl_3$) (*rac*-**1m**)

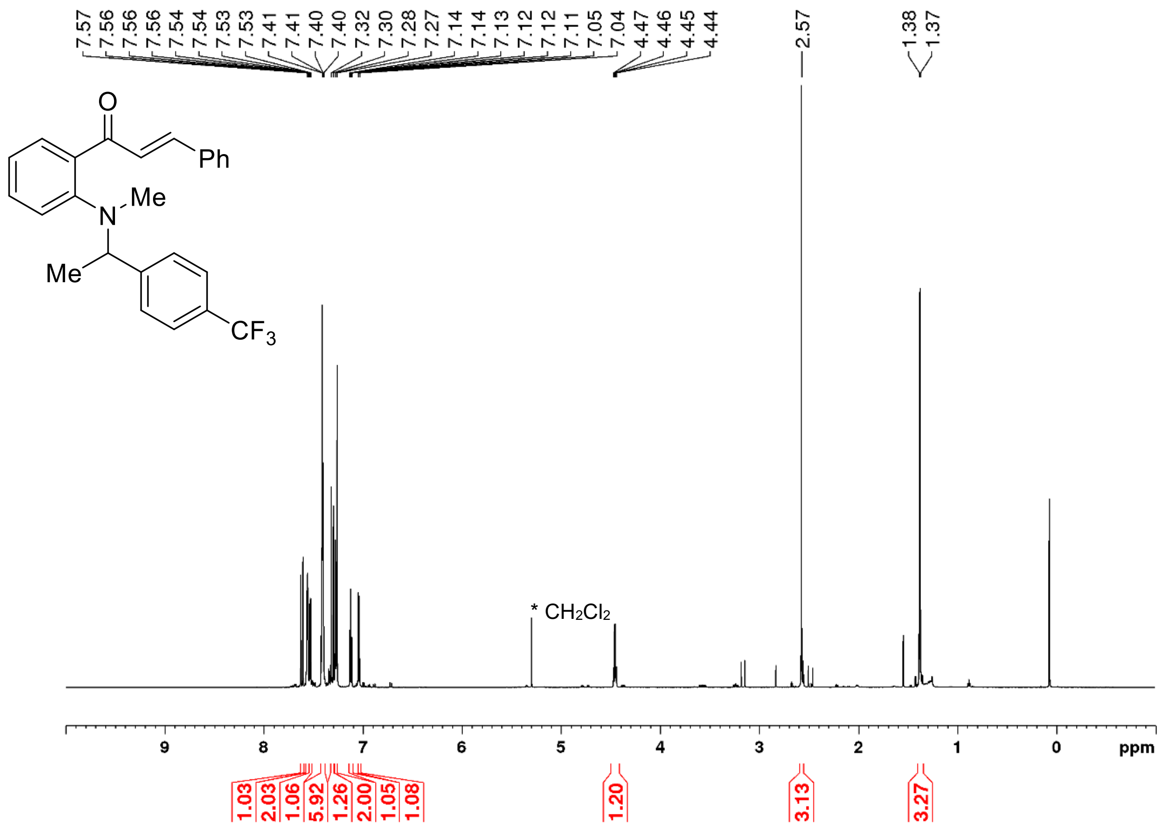

^{13}C-NMR (176 MHz, 298 K, $CDCl_3$)

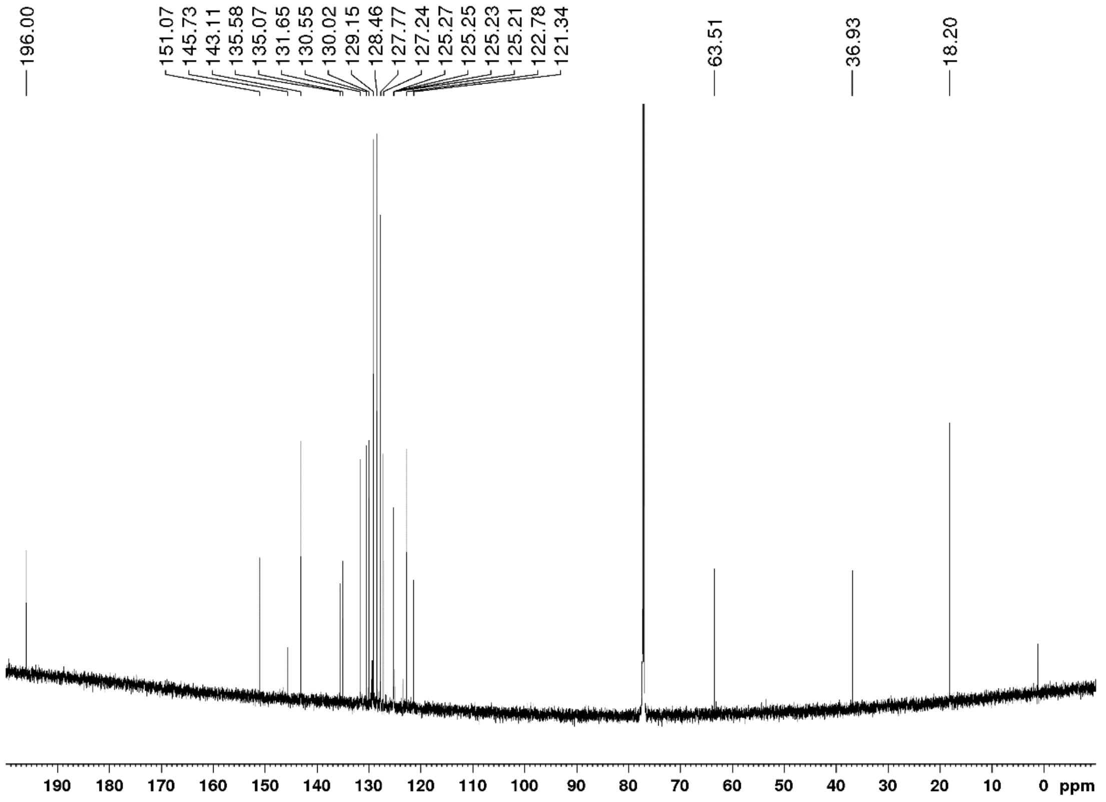

[1]**H-NMR** (700 MHz, 298 K, $CDCl_3$) (**1p**)

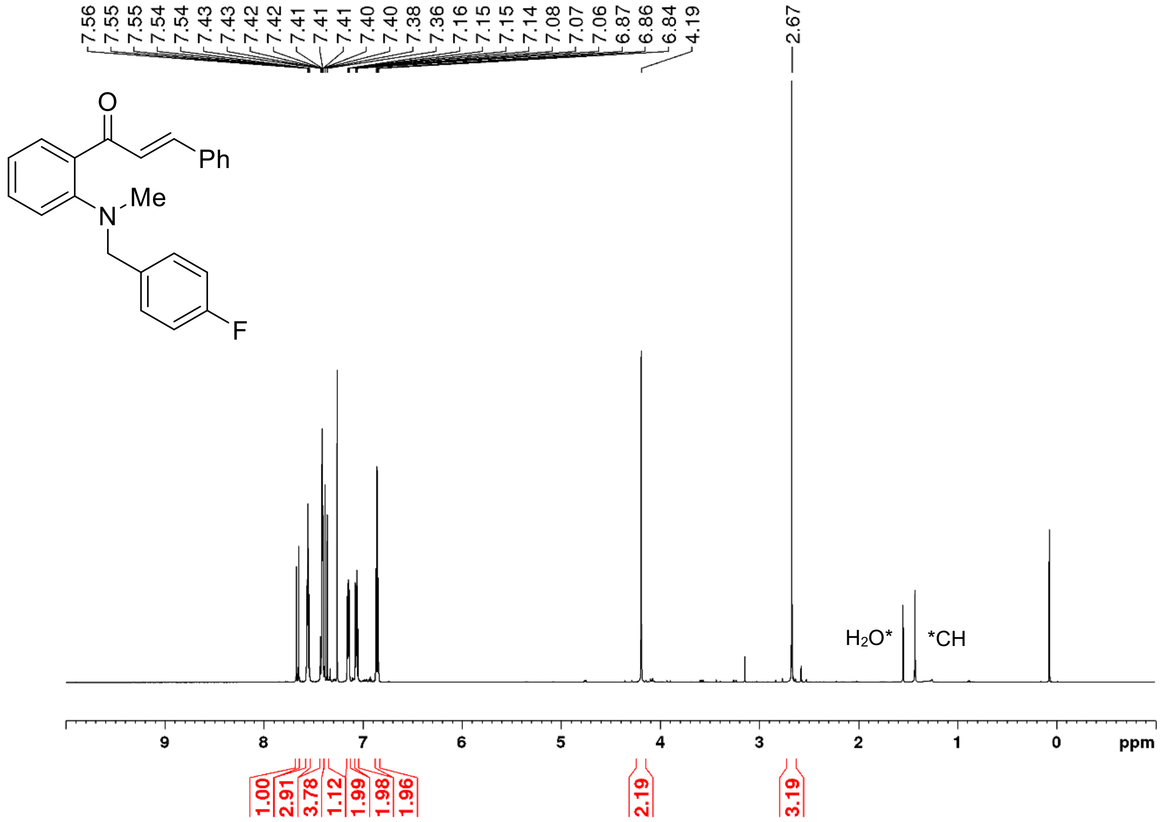

[13]**C-NMR** (176 MHz, 298 K, $CDCl_3$)

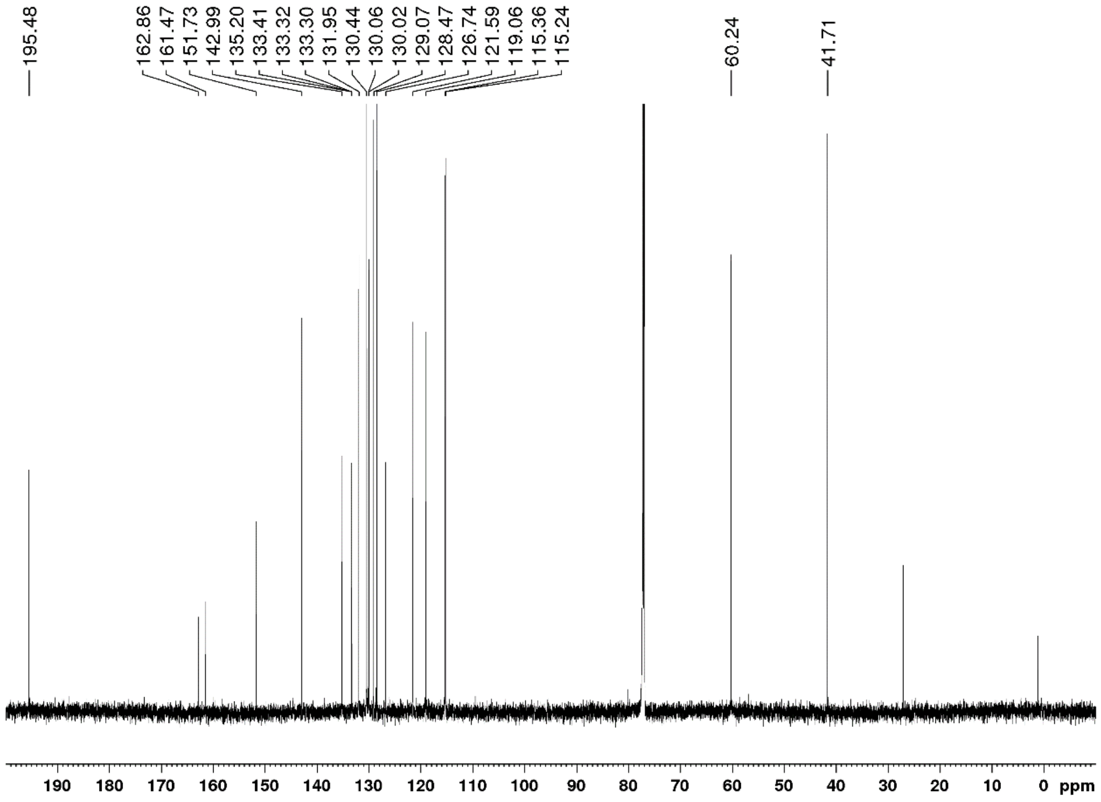

^{1}**H-NMR** (700 MHz, 298 K, $CDCl_3$) (**1q**)

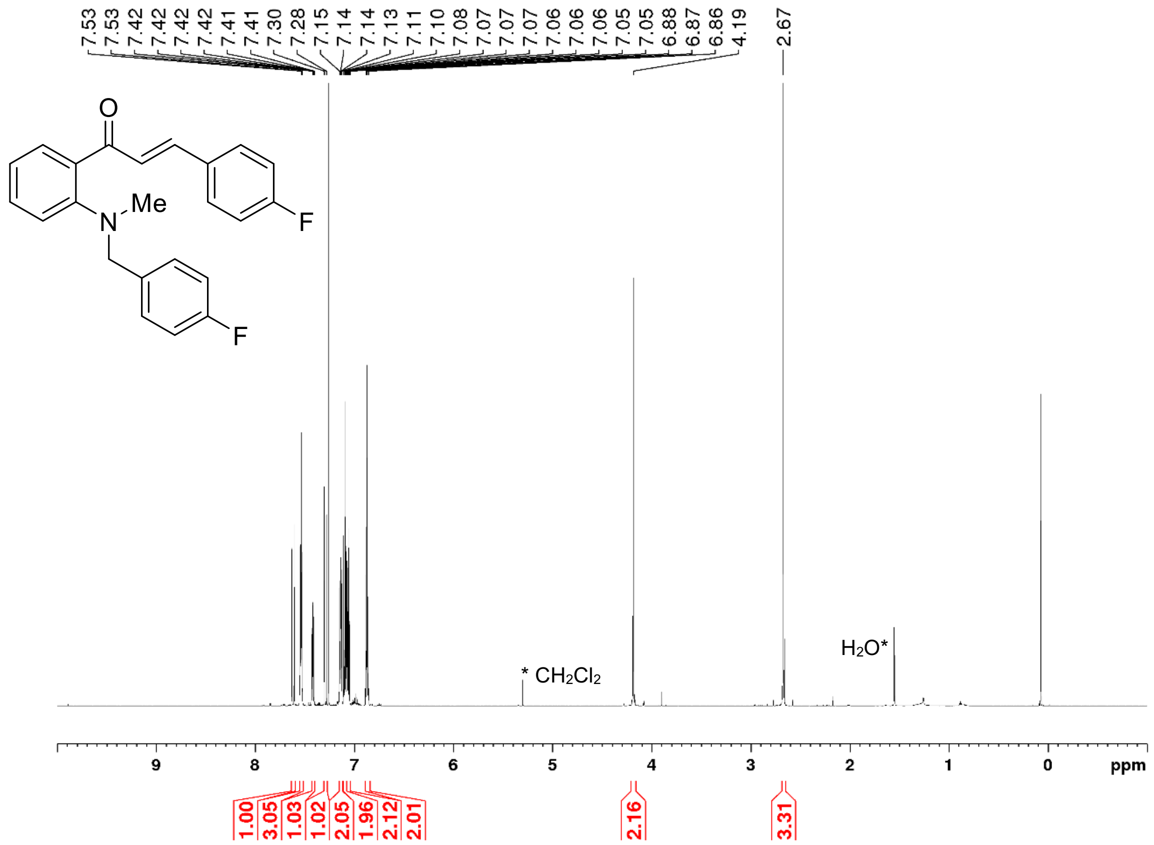

^{13}**C-NMR** (176 MHz, 298 K, $CDCl_3$)

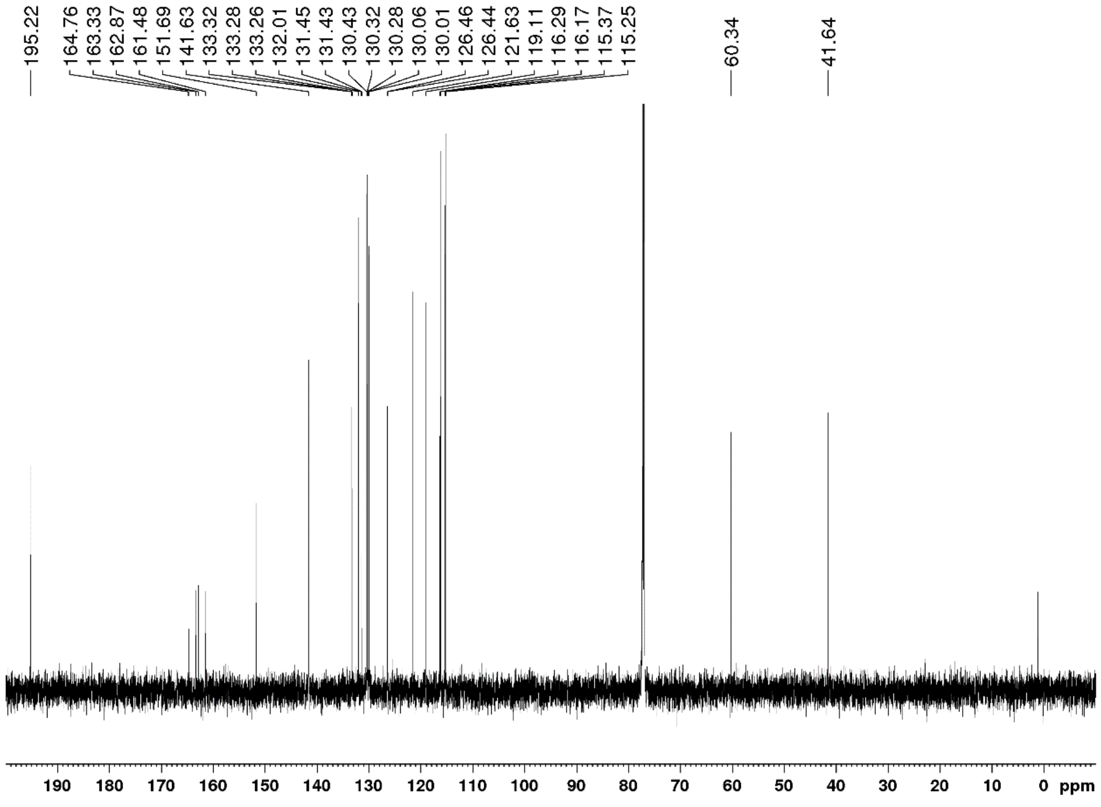

^{1}H-NMR (700 MHz, 298 K, $CDCl_3$) (**1r**)

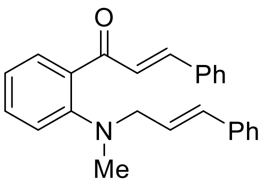

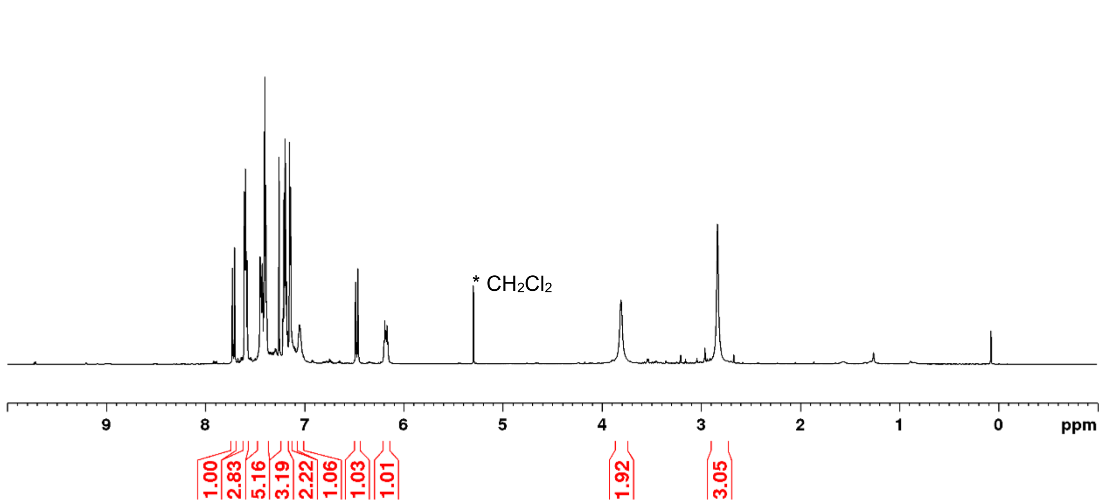

^{13}C-NMR (176 MHz, 298 K, $CDCl_3$)

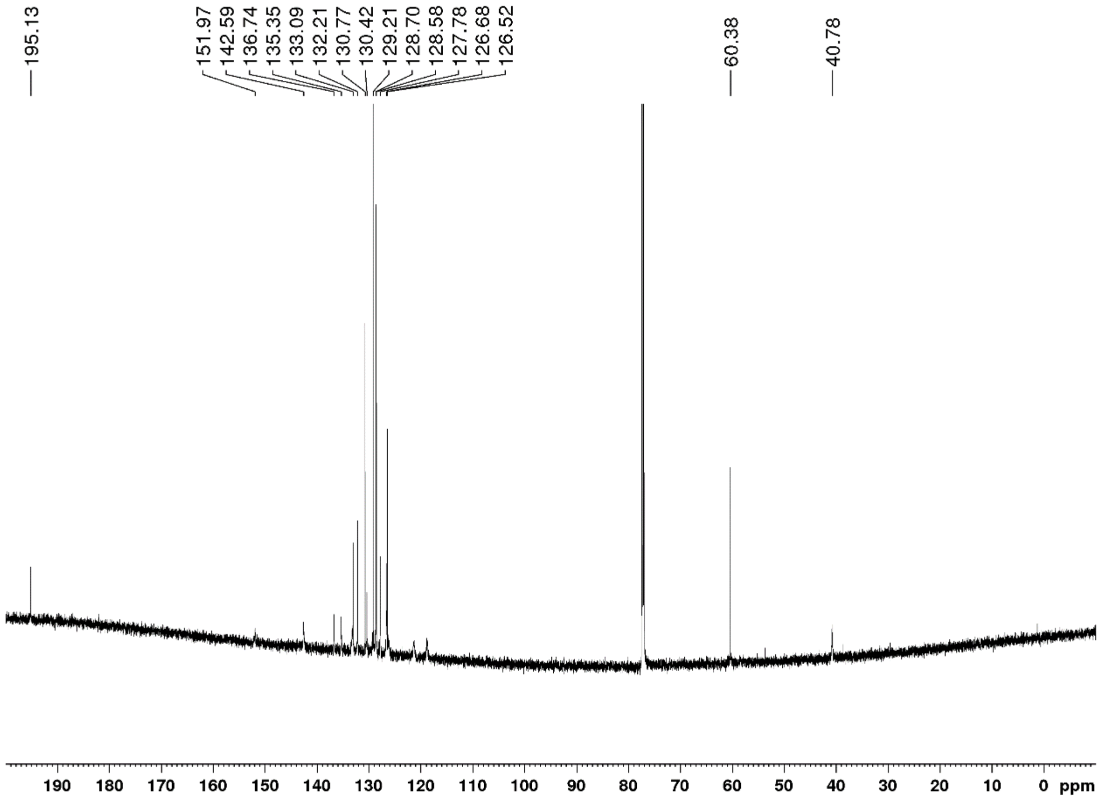

^{1}**H-NMR** (700 MHz, 298 K, $CDCl_3$) (**1s**)

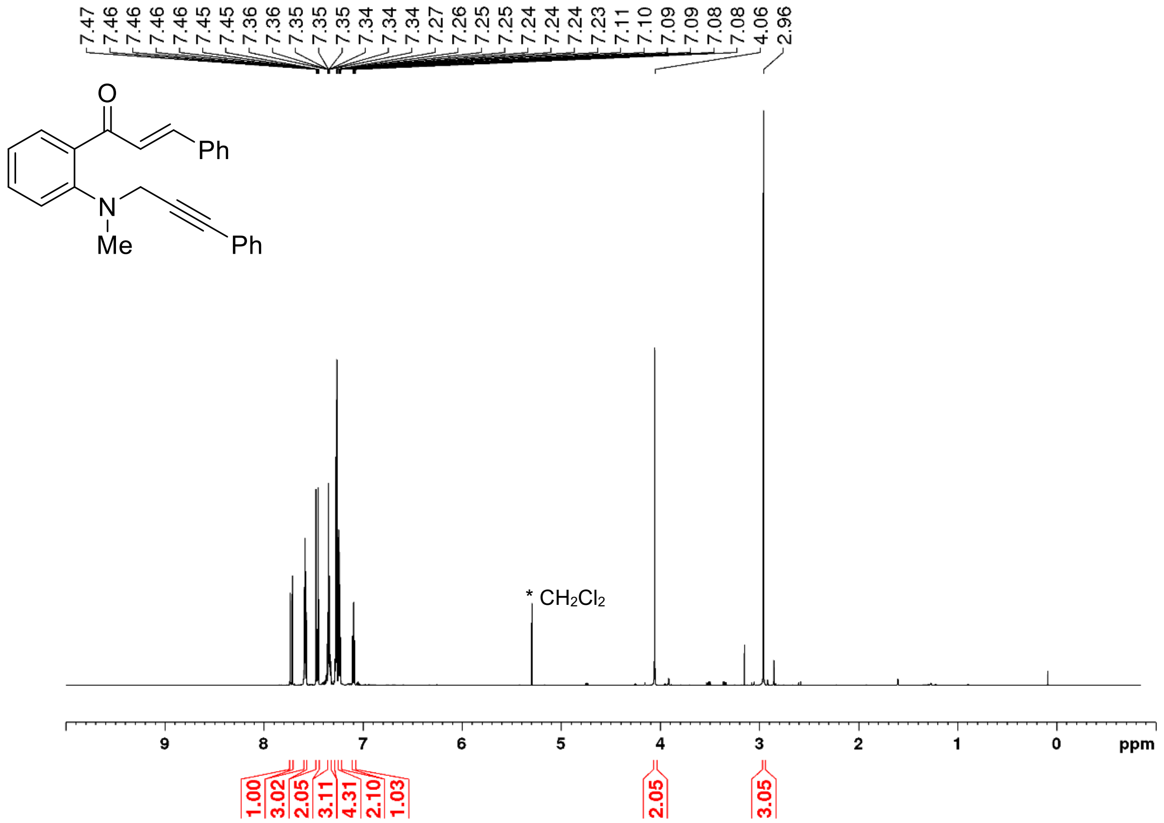

^{13}**C-NMR** (176 MHz, 298 K, $CDCl_3$)

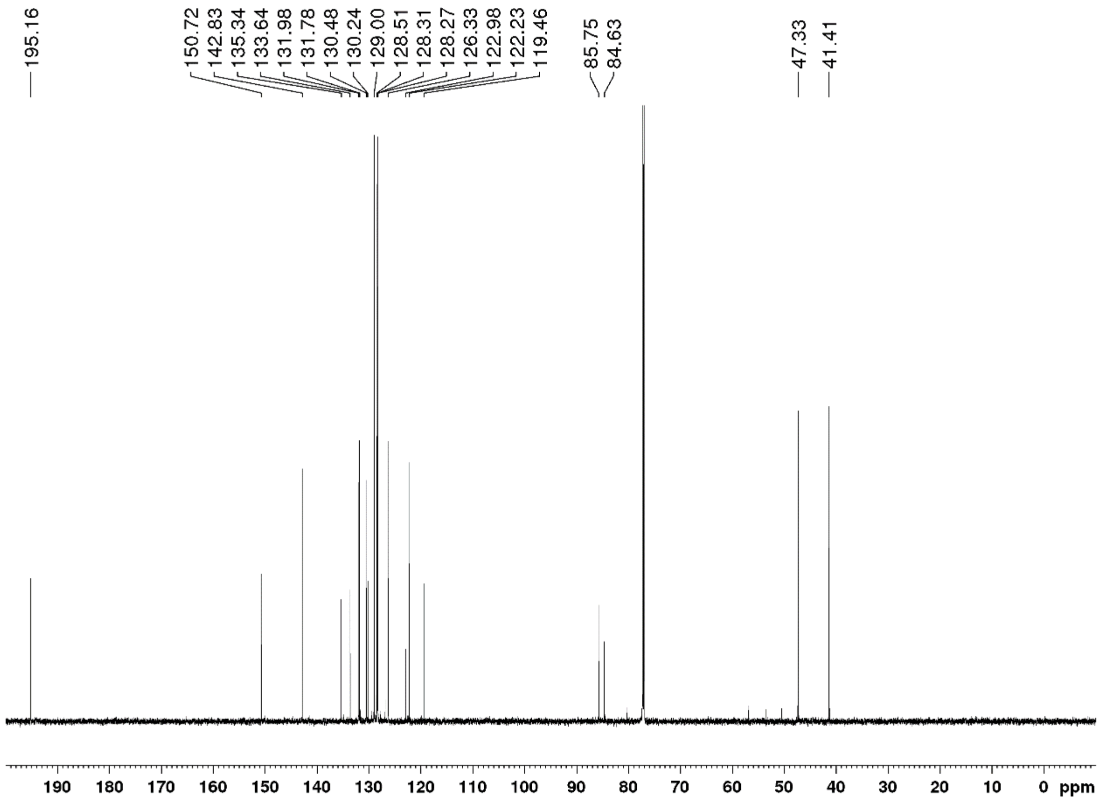

^{1}H-NMR (500 MHz, 303 K, $CDCl_3$) (**1t**)

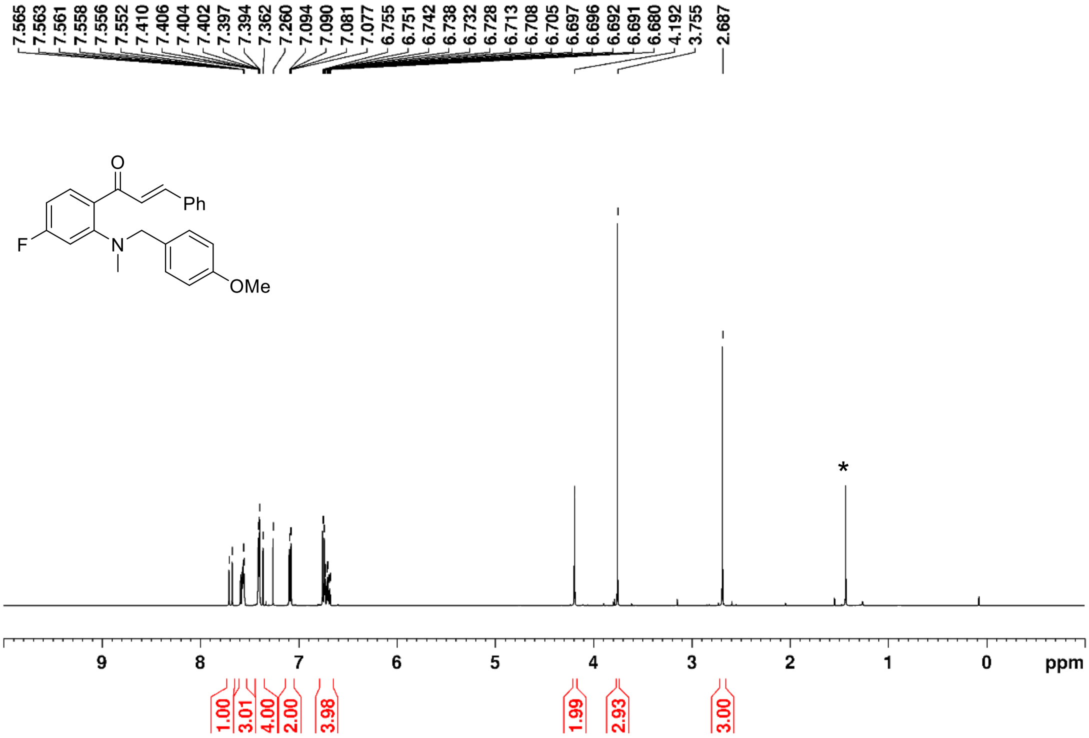

* residual hexanes resonance

^{13}C-NMR (176 MHz, 298 K, $CDCl_3$) (**1t**)

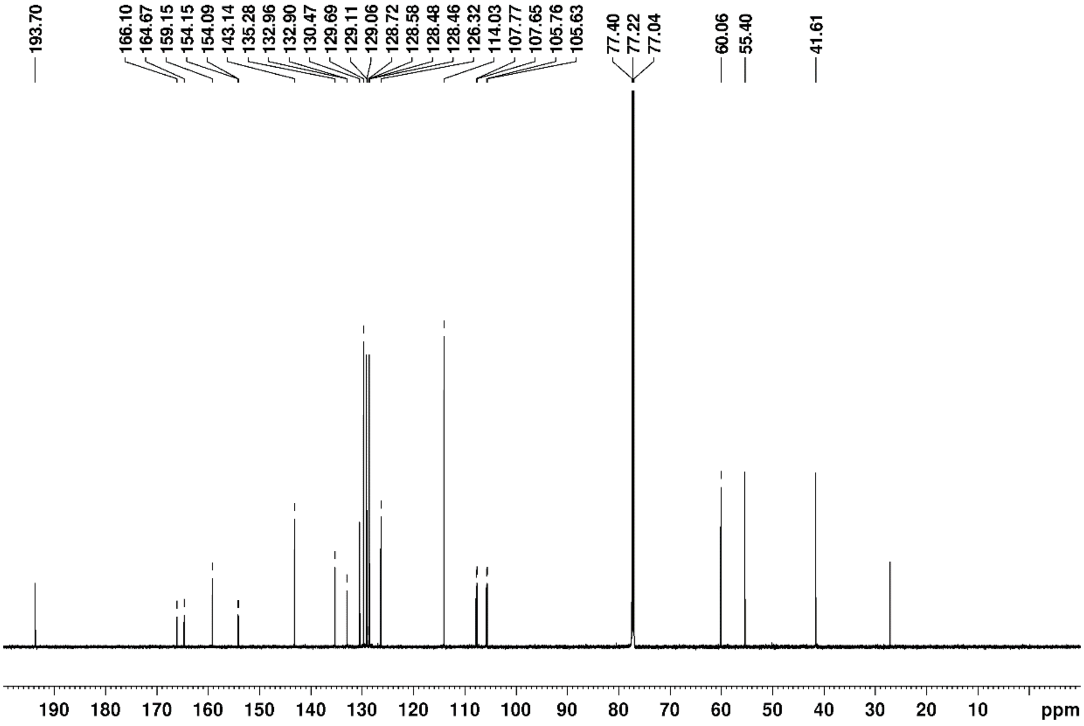

^{1}H-NMR (500 MHz, 303 K, $CDCl_3$) (**1u**)

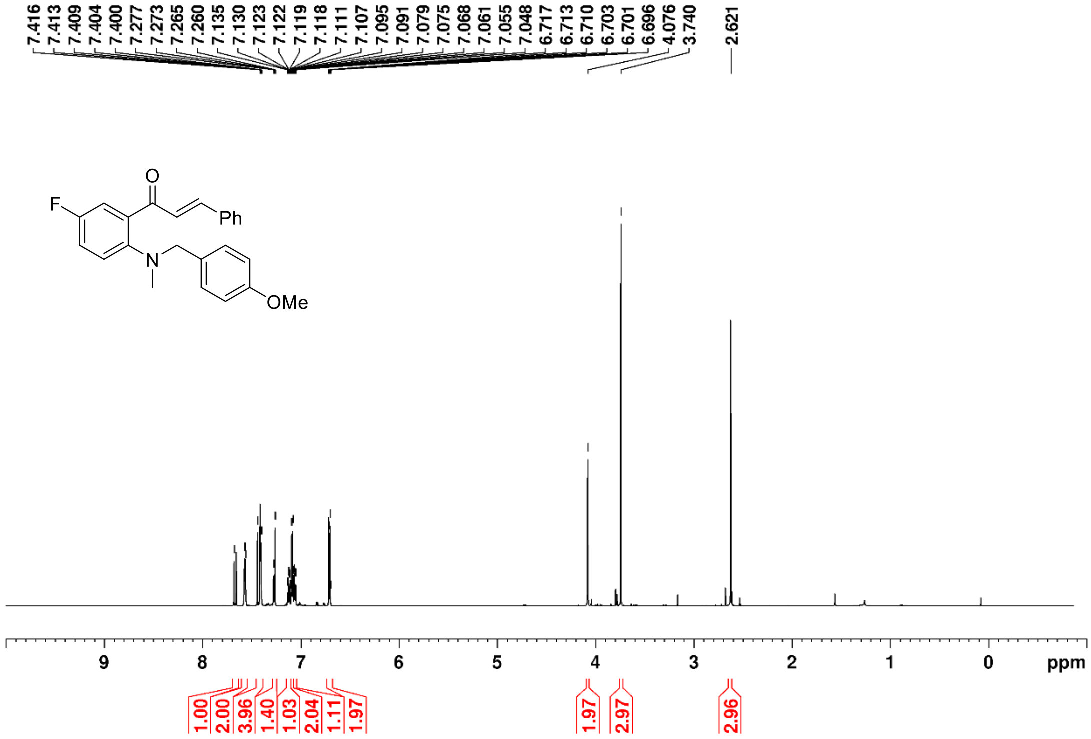

^{13}C-NMR (176 MHz, 298 K, $CDCl_3$) (**1u**)

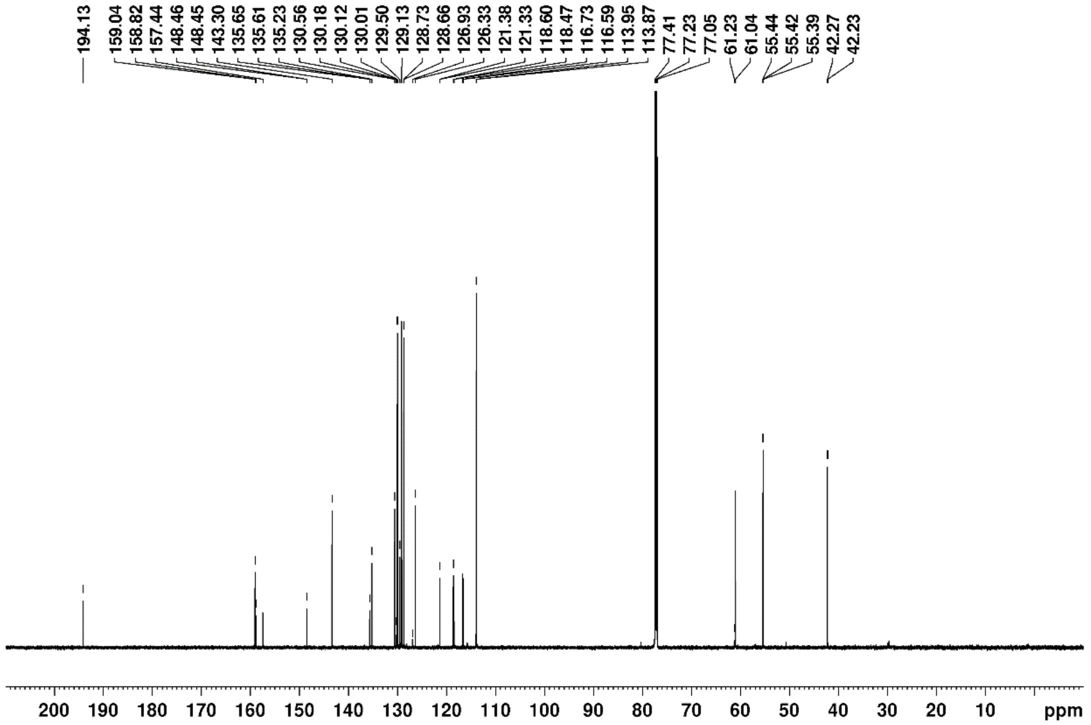

^{1}H-NMR (700 MHz, 298 K, $CDCl_3$) (**1v**)

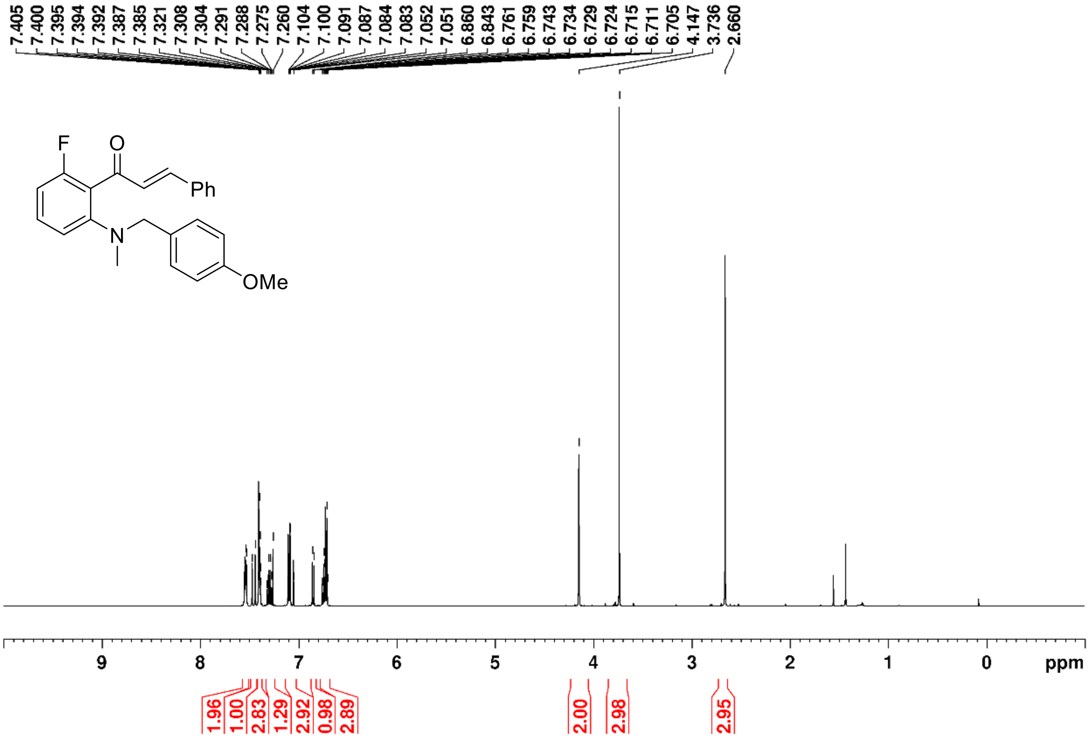

^{13}C-NMR (176 MHz, 298 K, $CDCl_3$) (**1v**)

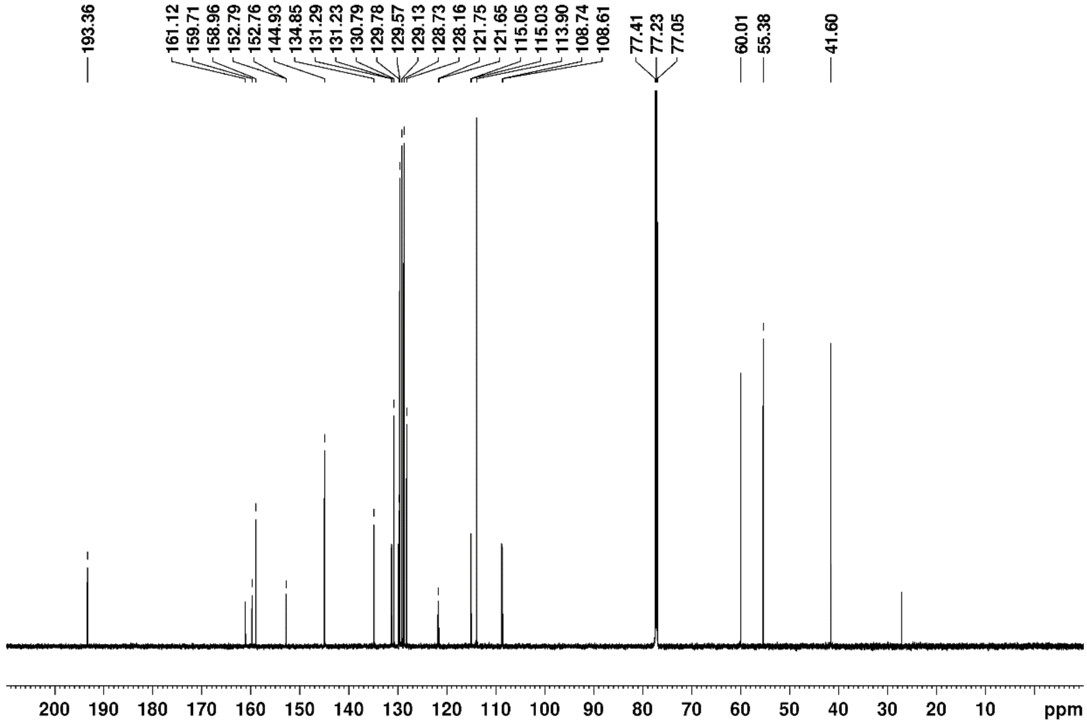

^{1}**H-NMR** (500 MHz, 303 K, $CDCl_3$) (**1w**)

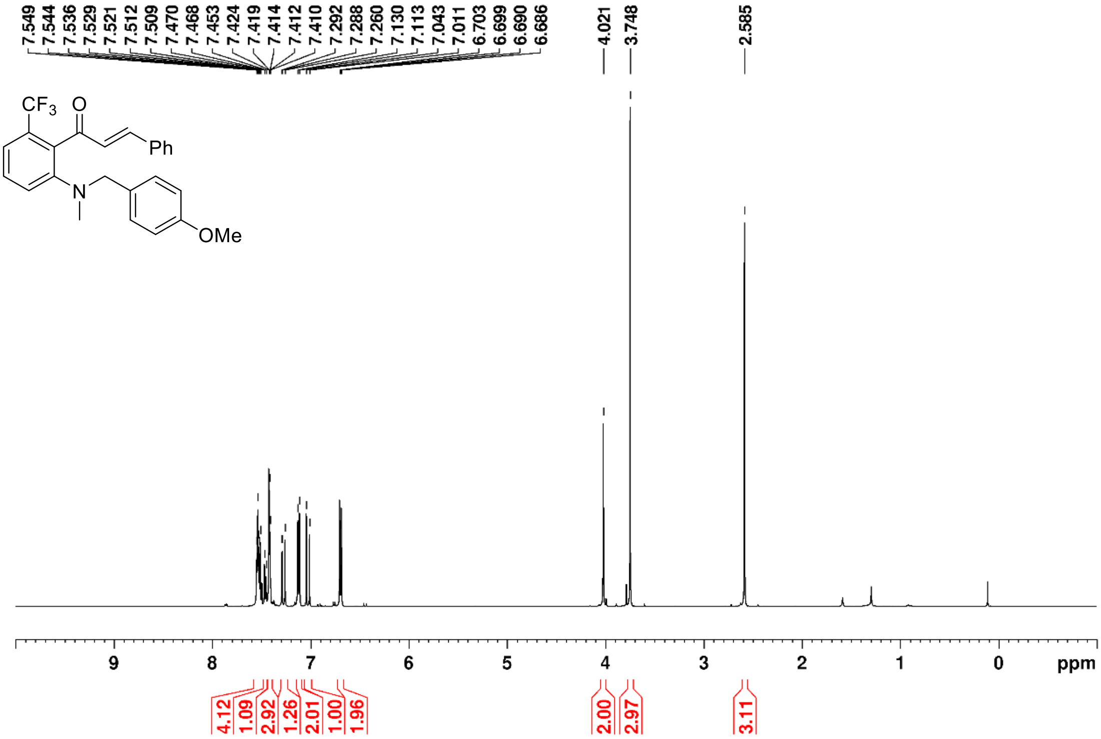

^{13}**C-NMR** (176 MHz, 298 K, $CDCl_3$) (**1w**)

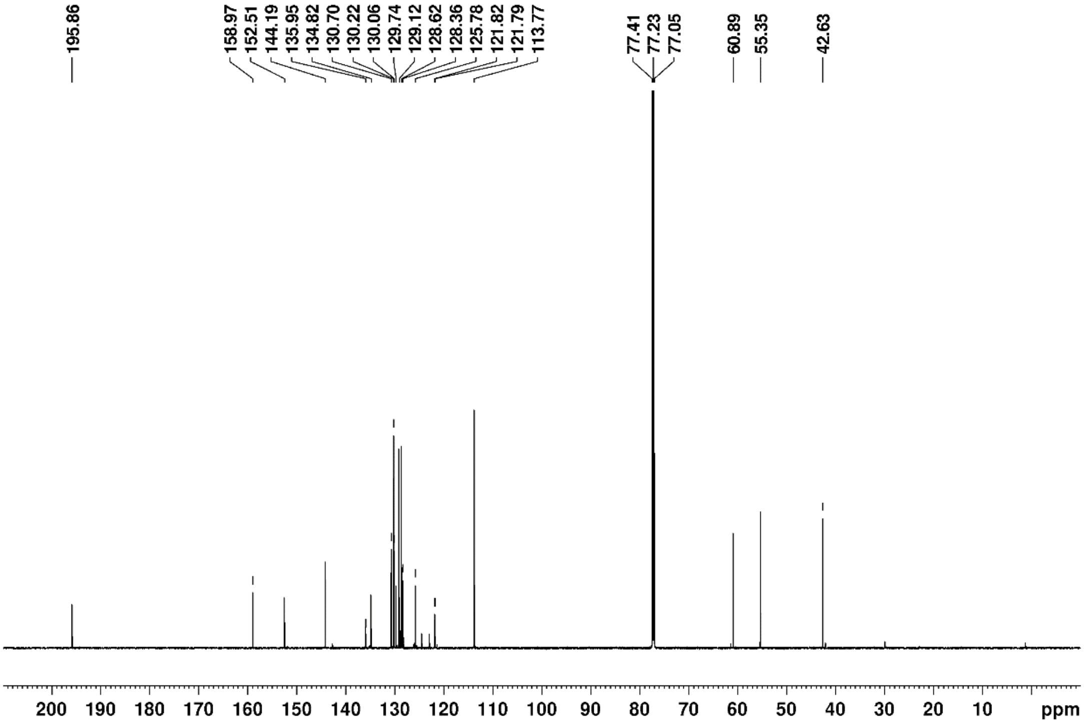

[1]**H-NMR** (500 MHz, 303 K, $CDCl_3$) (**1x**)

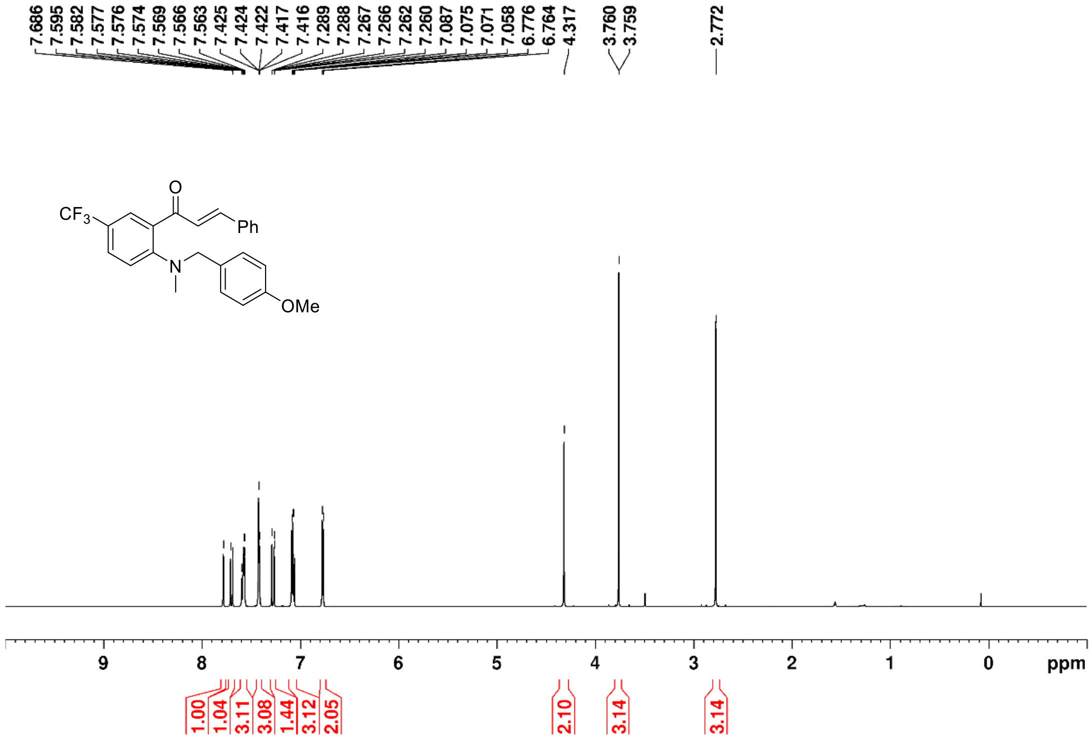

[13]**C-NMR** (176 MHz, 298 K, $CDCl_3$) (**1x**)

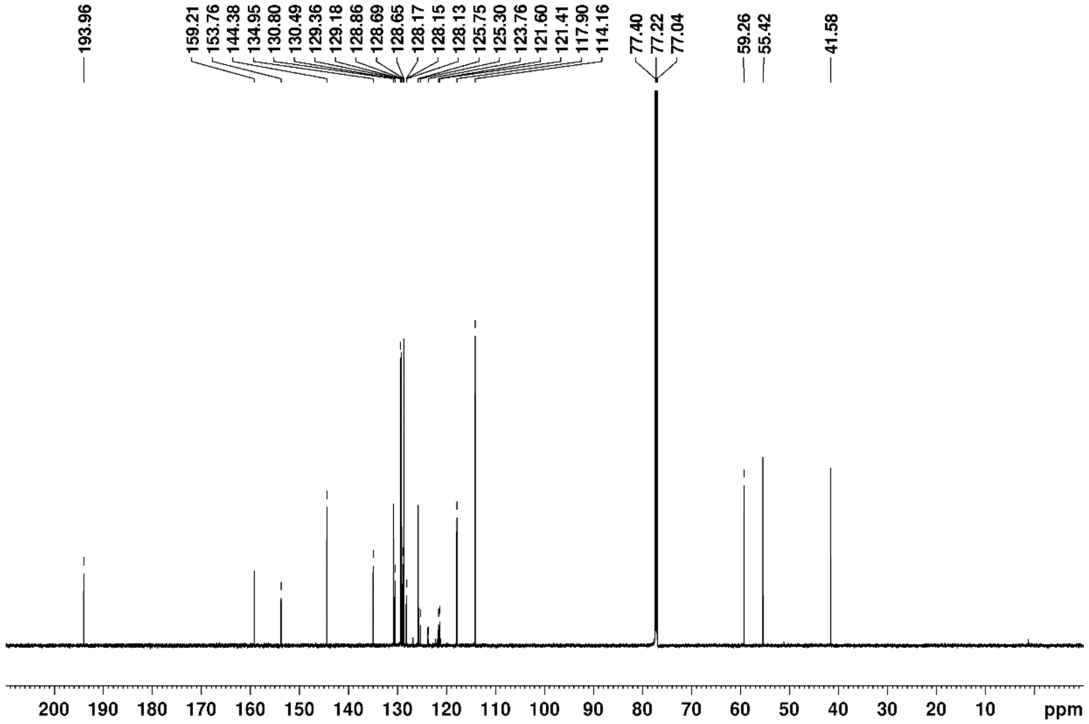

^{1}H-NMR (700 MHz, 298 K, $CDCl_3$) (**1y**)

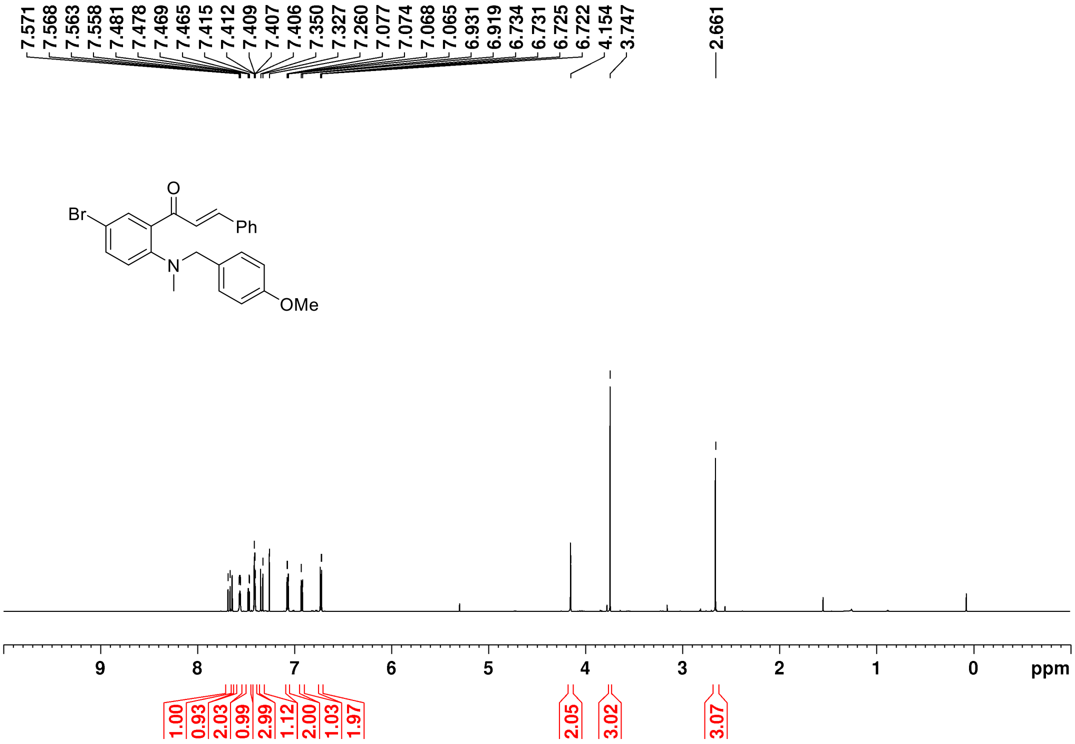

^{13}C-NMR (176 MHz, 298 K, $CDCl_3$) (**1y**)

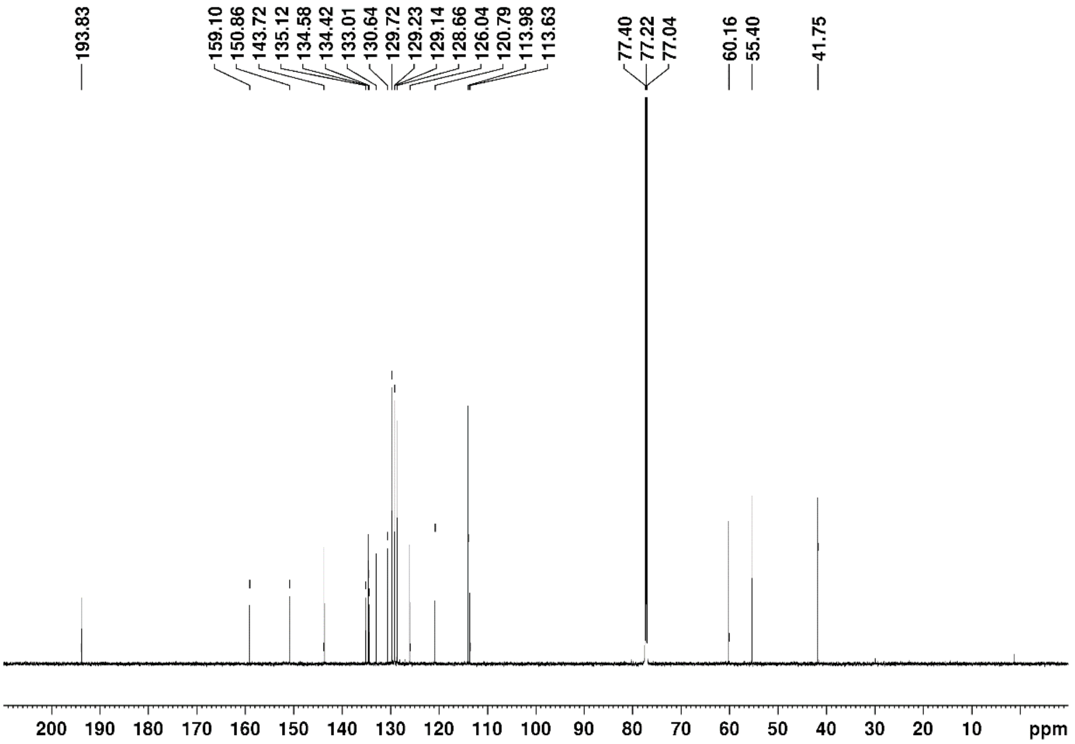

^{1}H-NMR (700 MHz, 298 K, $CDCl_3$) (**1z**)

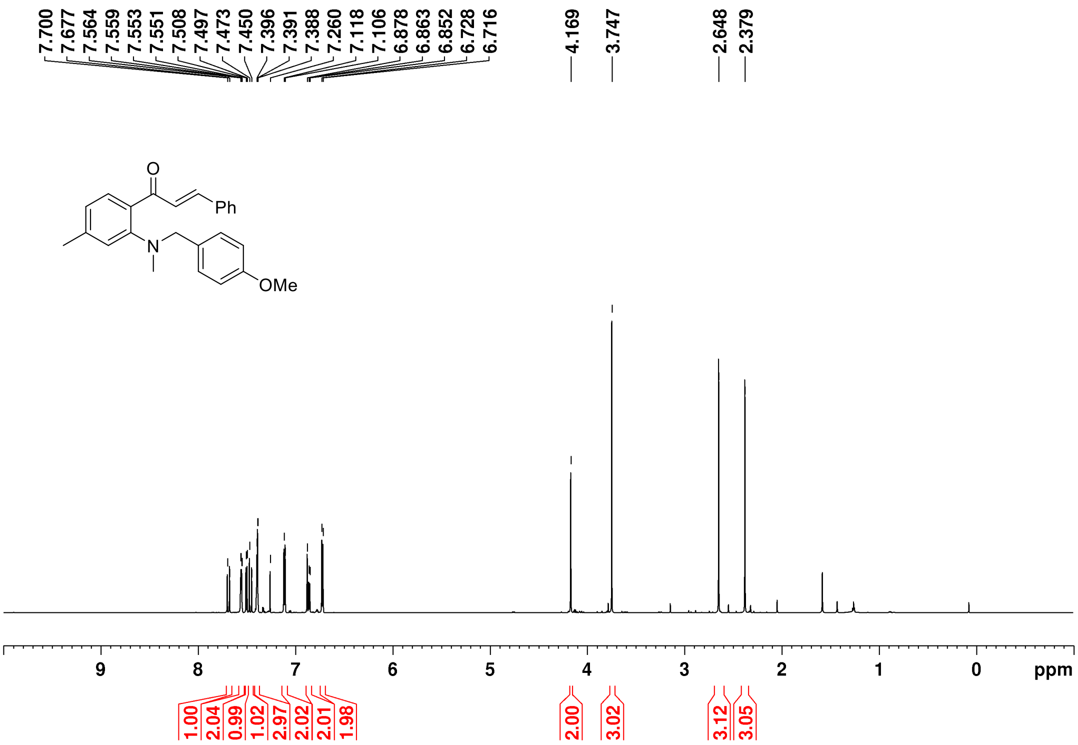

^{13}C-NMR (176 MHz, 298 K, $CDCl_3$) (**1z**)

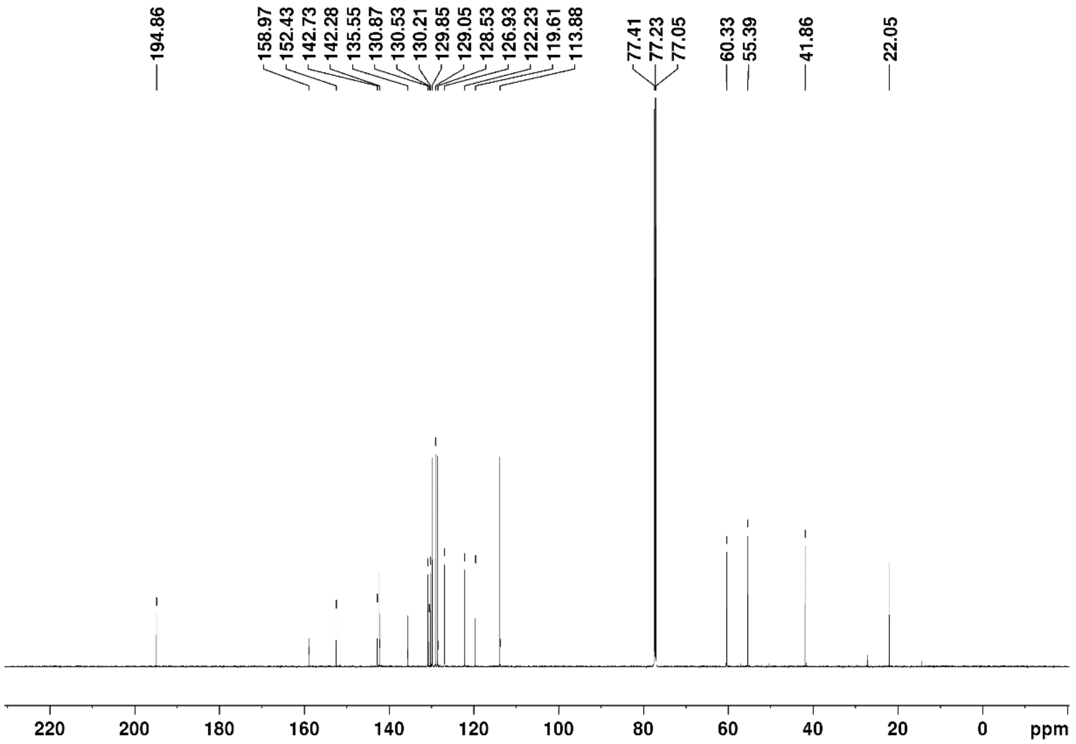

^{1}**H-NMR** (700 MHz, 298 K, $CDCl_3$) (**1aa**)

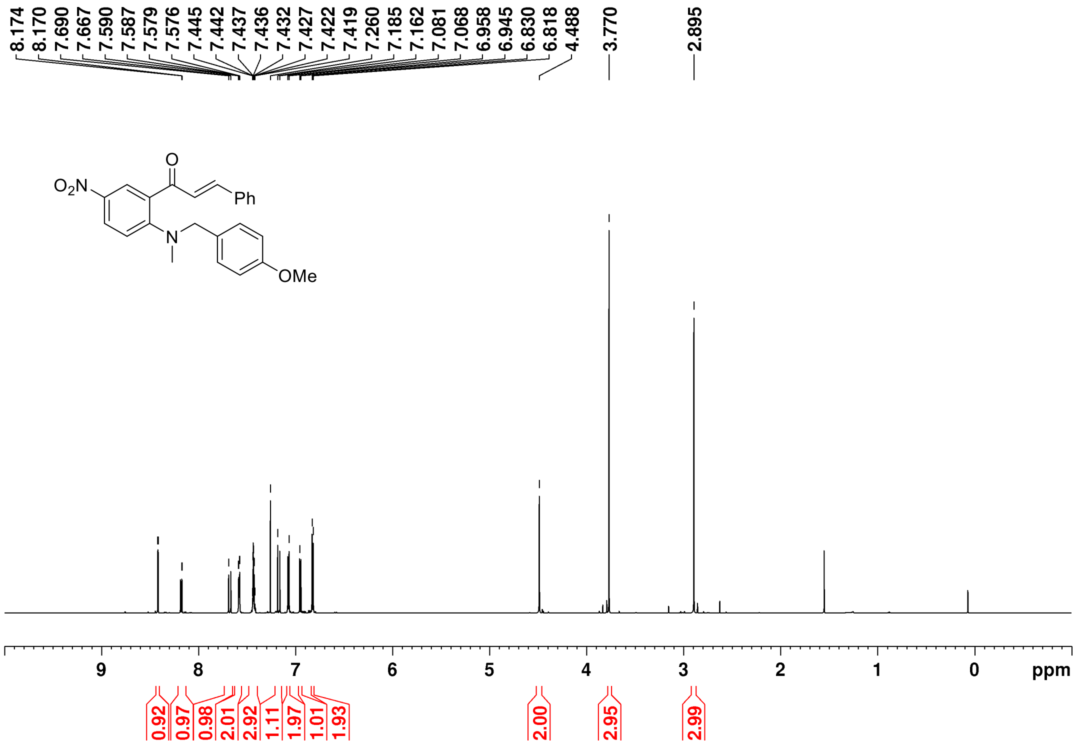

^{13}**C-NMR** (176 MHz, 298 K, $CDCl_3$) (**1aa**)

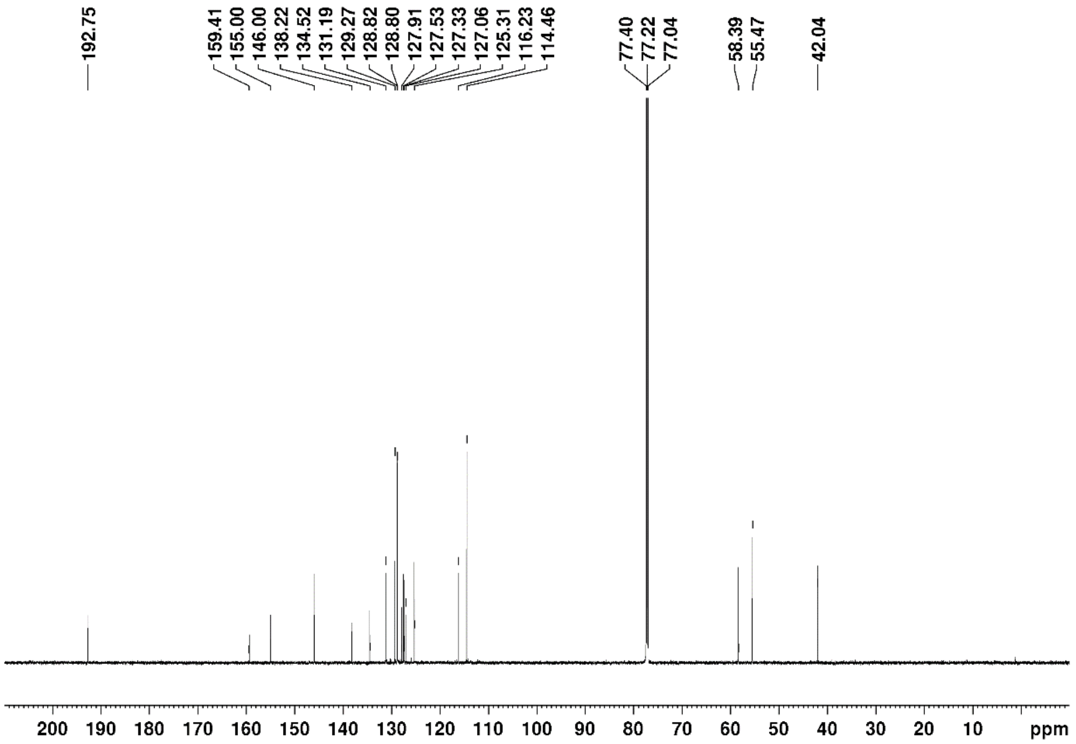

^{1}H-NMR (700 MHz, 298 K, $CDCl_3$) (**1ab**)

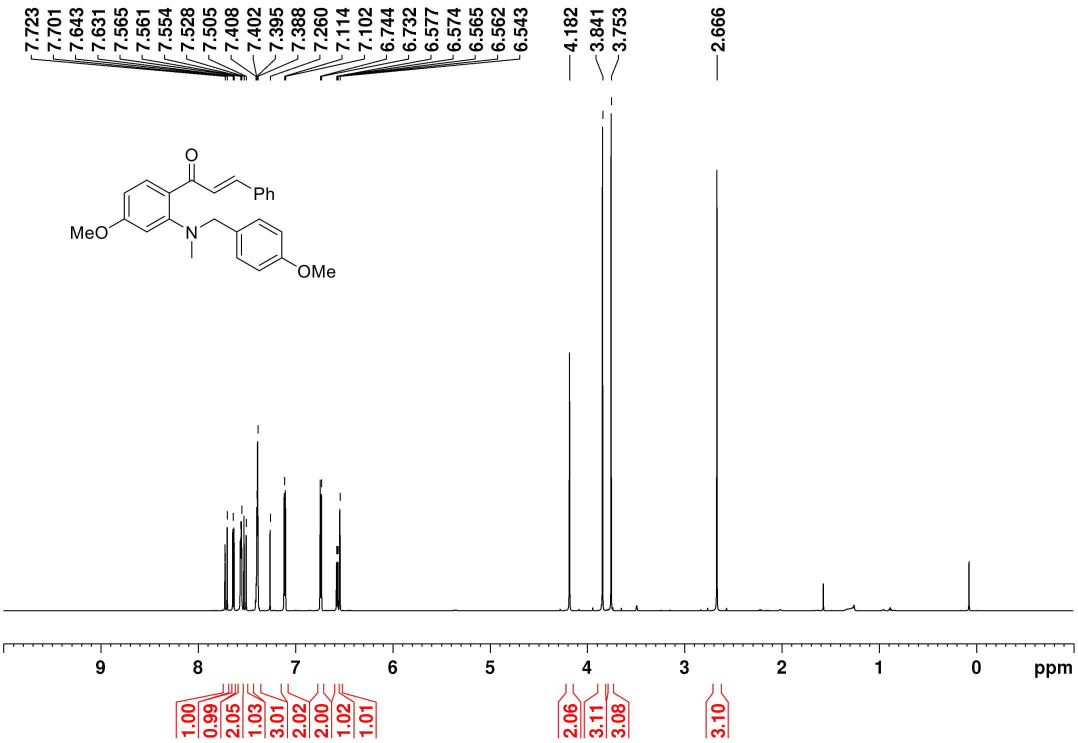

^{13}C-NMR (176 MHz, 298 K, $CDCl_3$) (**1ab**)

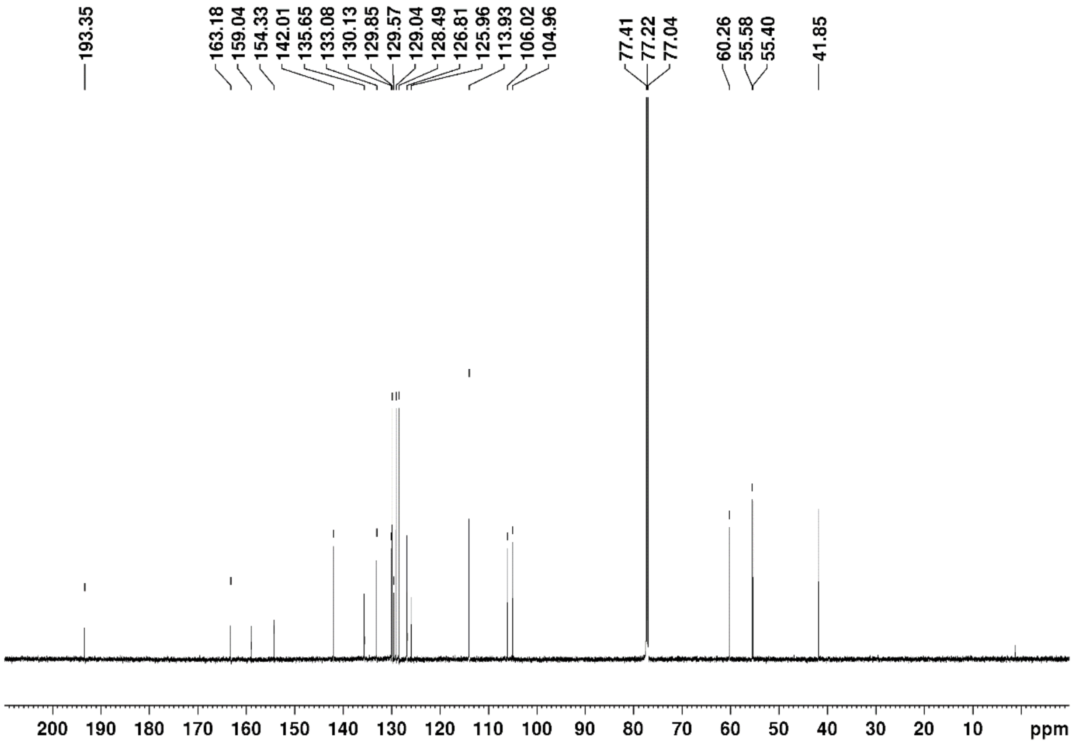

3.3 NMR spectra of cyclization products

^{1}H-NMR (700 MHz, 298 K, CD_2Cl_2) (*rac*-**2a**)

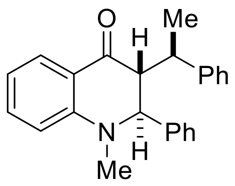

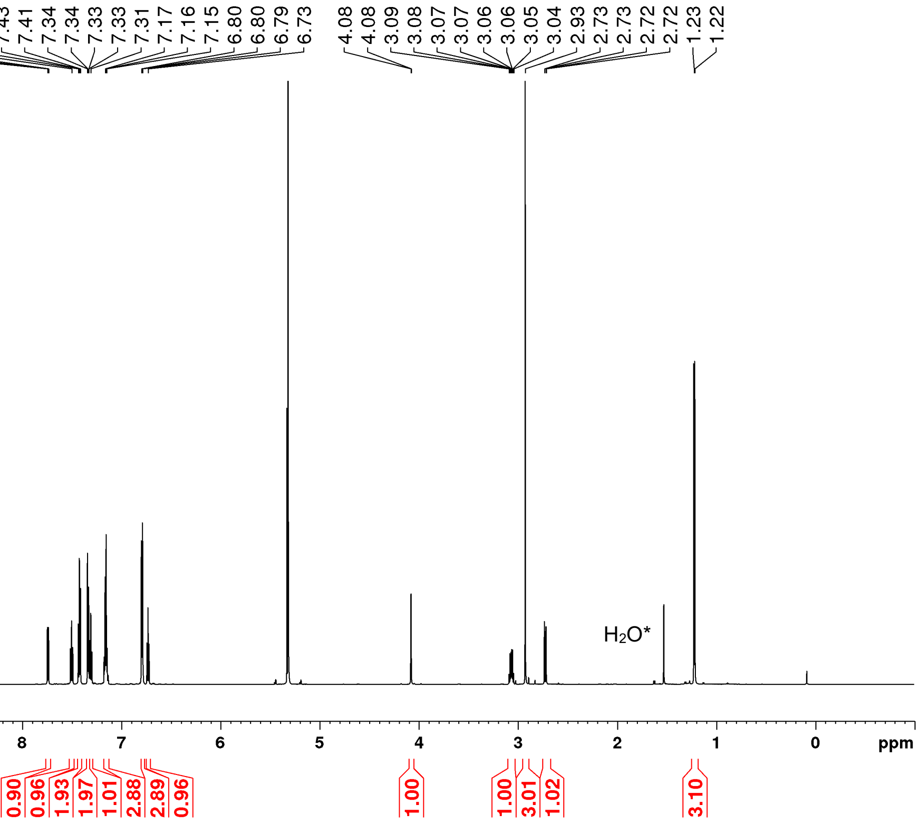

^{13}C-NMR (176 MHz, 298 K, CD_2Cl_2)

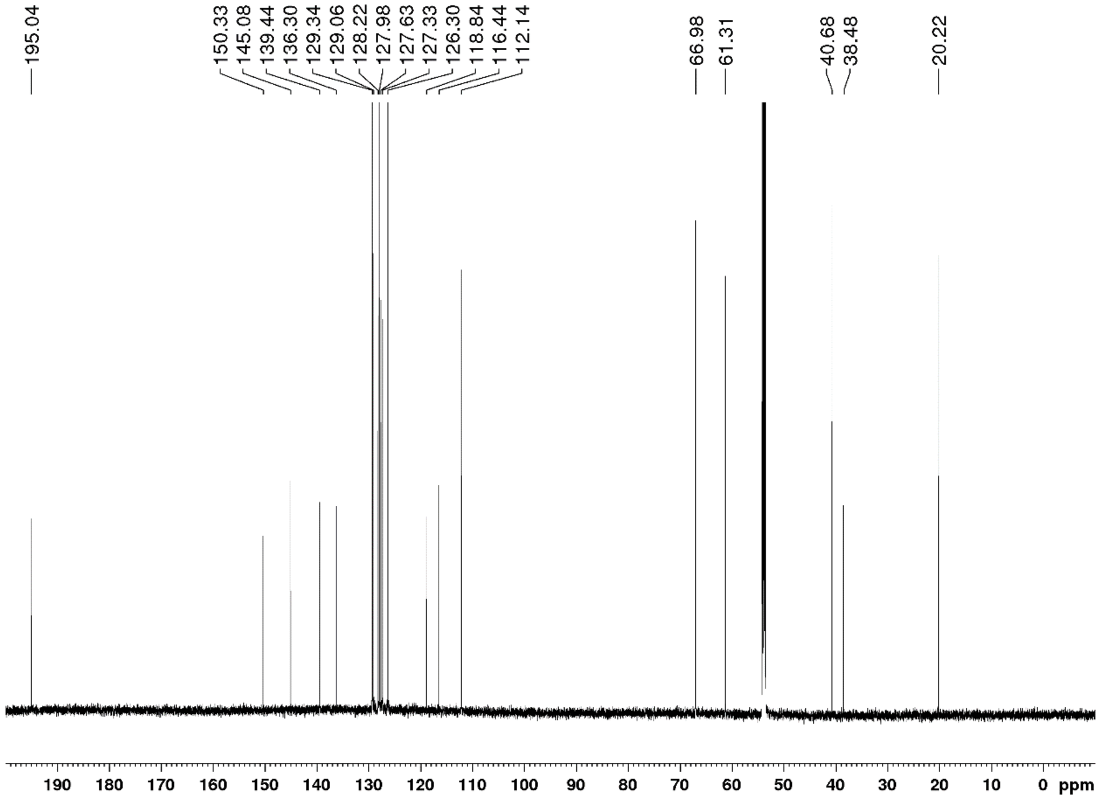

^{1}H-NMR (700 MHz, 298 K, CD_2Cl_2) (d_1-*rac*-**2a**)

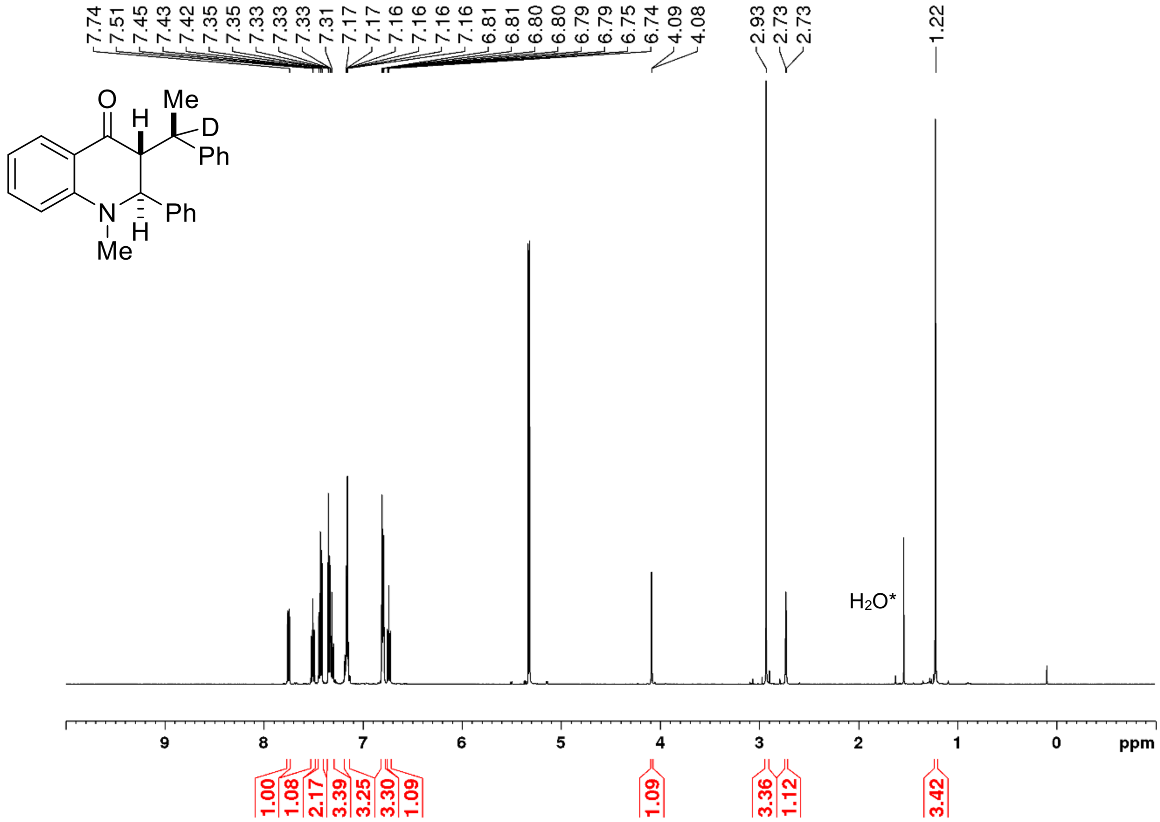

^{13}C-NMR (176 MHz, 298 K, CD_2Cl_2)

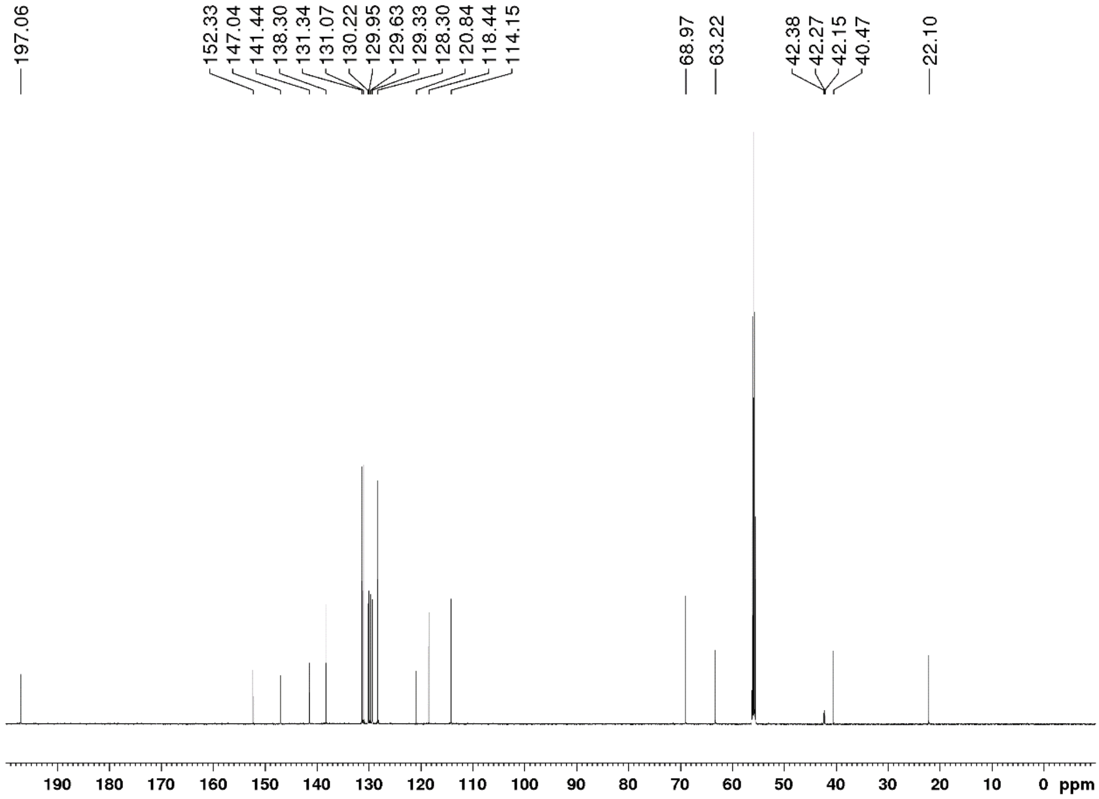

^{1}H-NMR (700 MHz, 298 K, CD_2Cl_2) ((*2S,3R*)-**2a**)

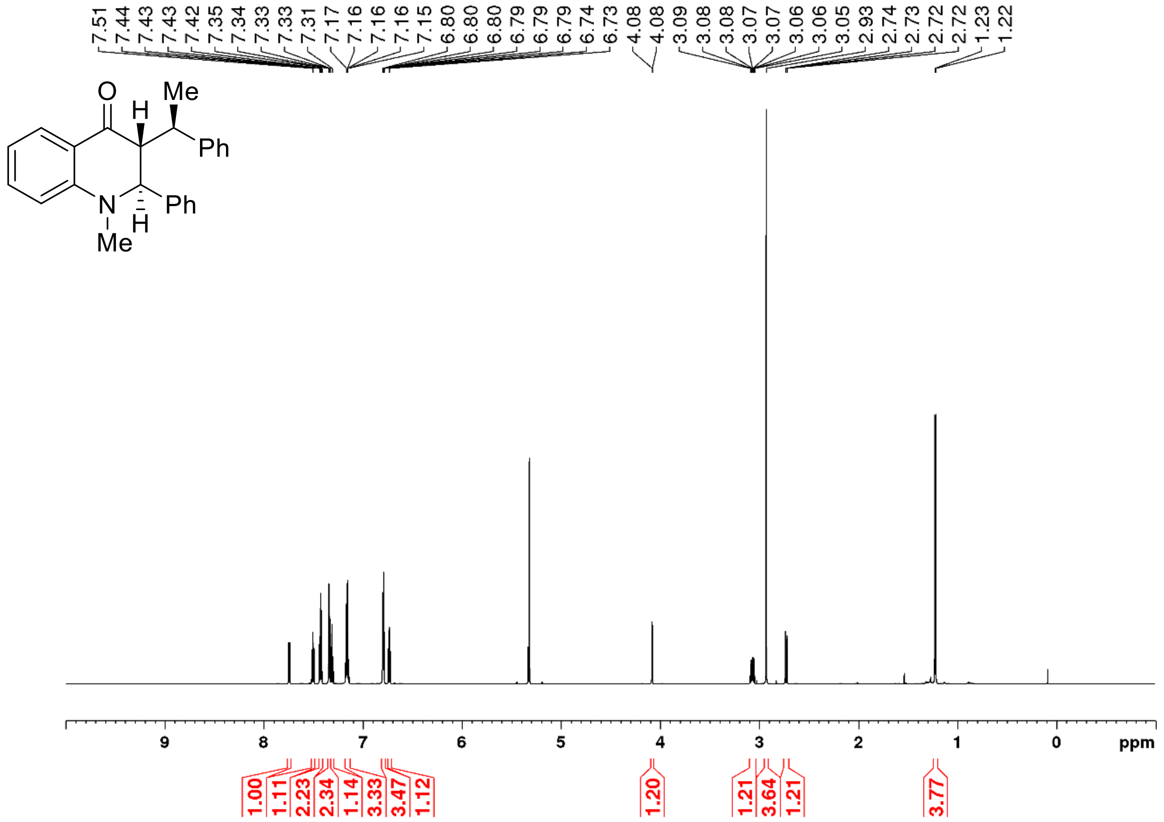

^{13}C-NMR (176 MHz, 298 K, CD_2Cl_2)

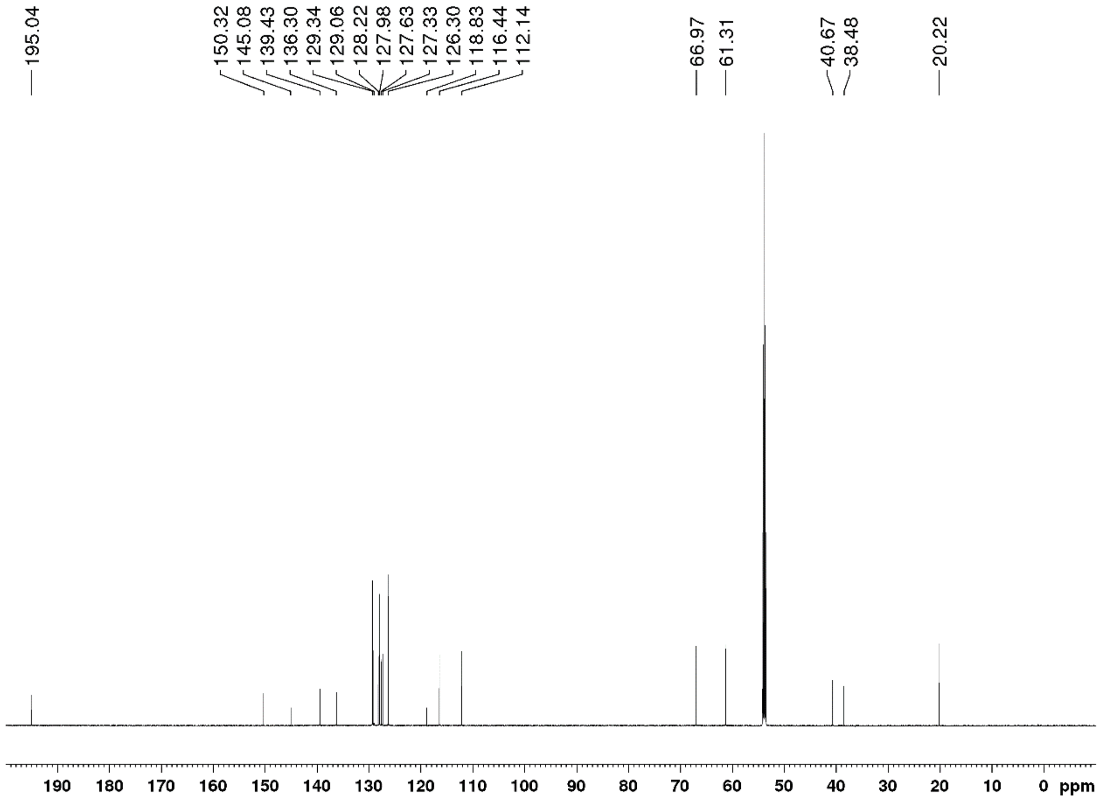

^{1}H-NMR (700 MHz, 298 K, $CDCl_3$) (**2b**)

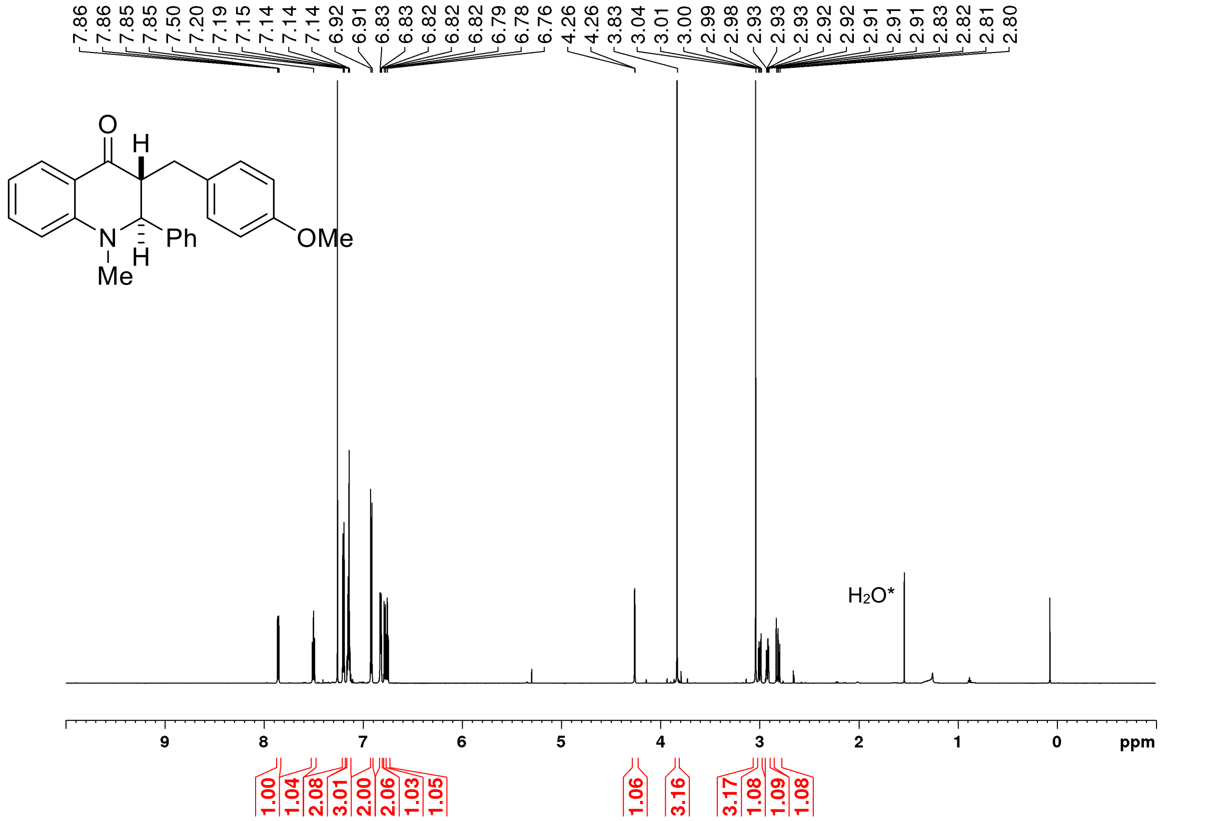

^{13}C-NMR (176 MHz, 298 K, $CDCl_3$)

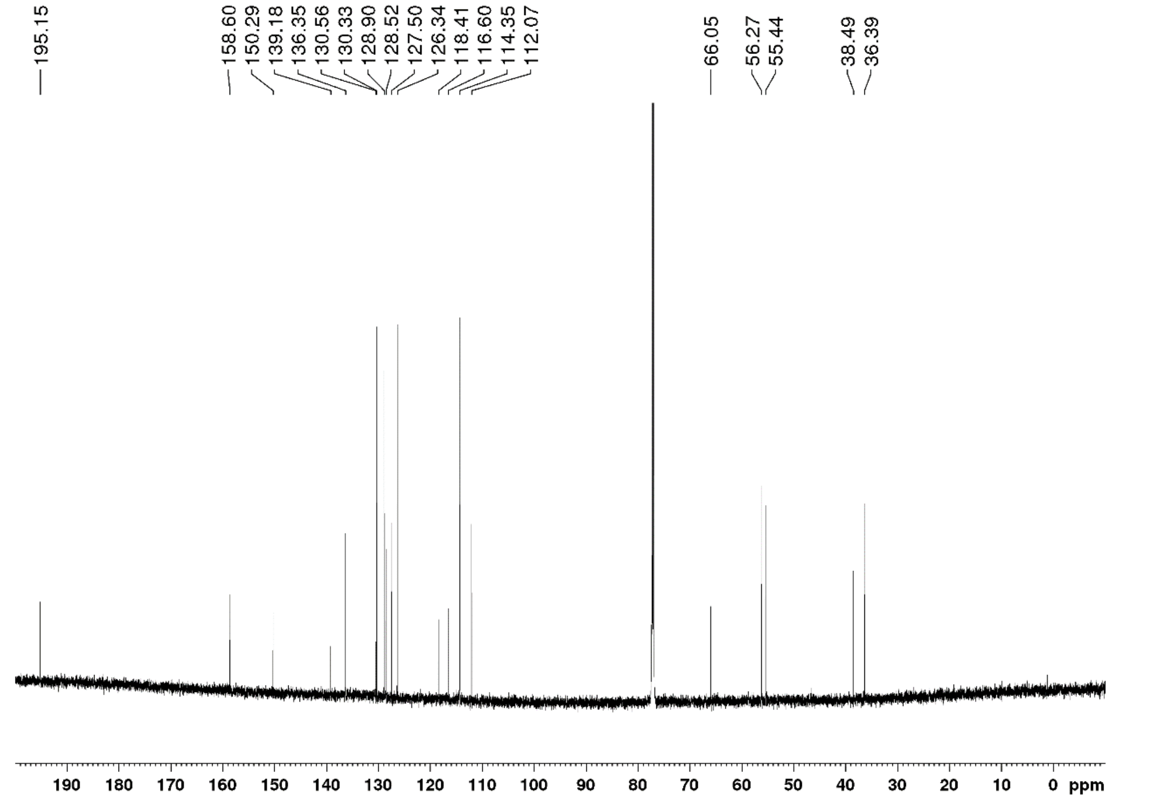

^{1}H-NMR (700 MHz, 298 K, $CDCl_3$) (**2c**)

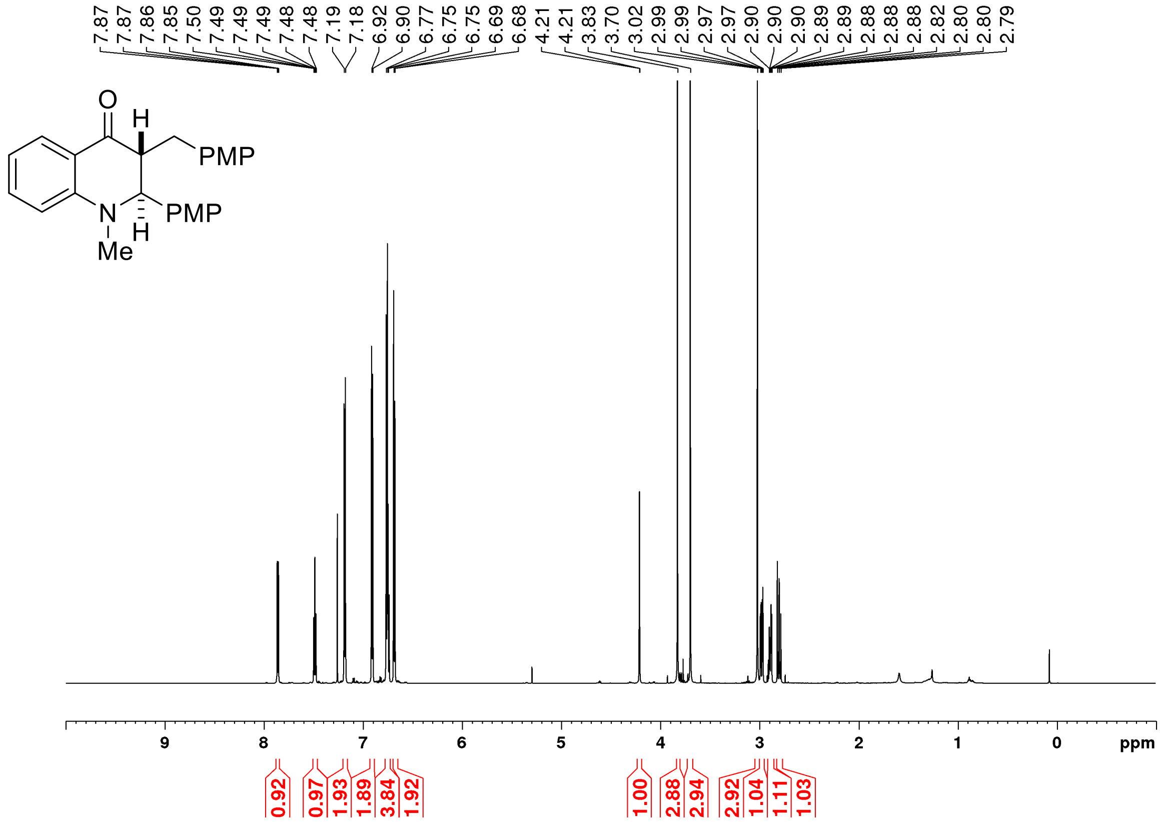

^{13}C-NMR (176 MHz, 298 K, $CDCl_3$)

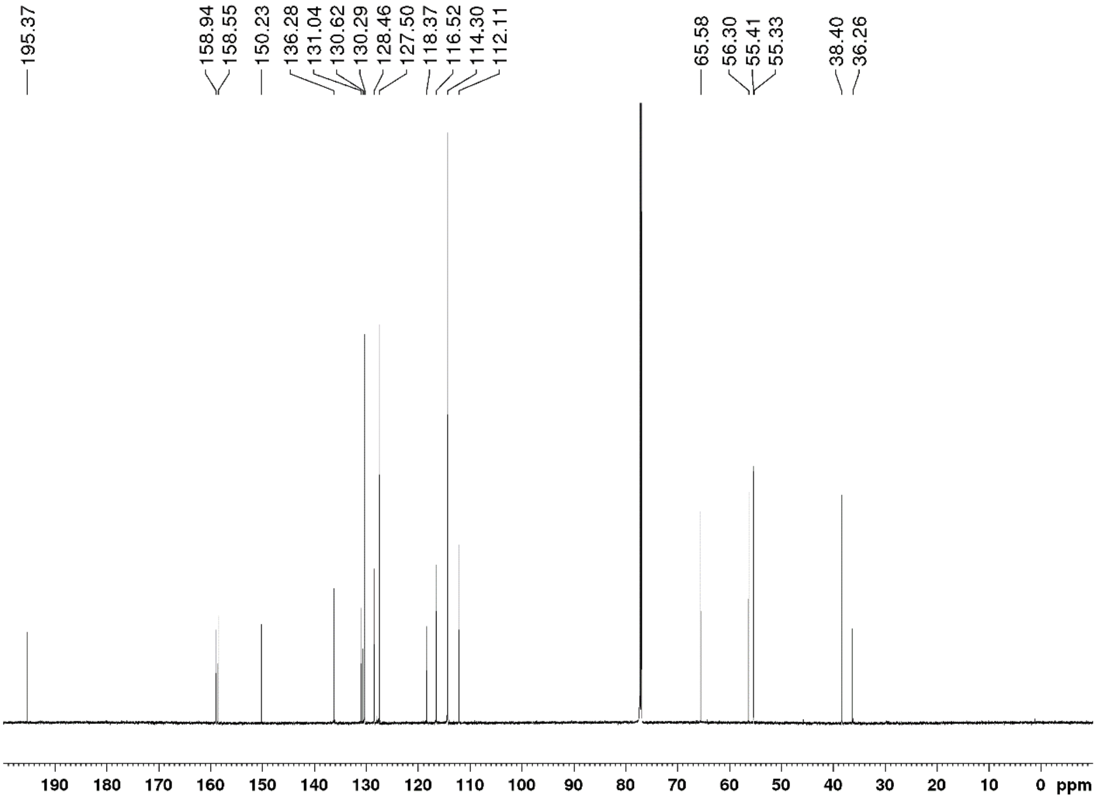

^{1}H-NMR (700 MHz, 298 K, $CDCl_3$) (2d)

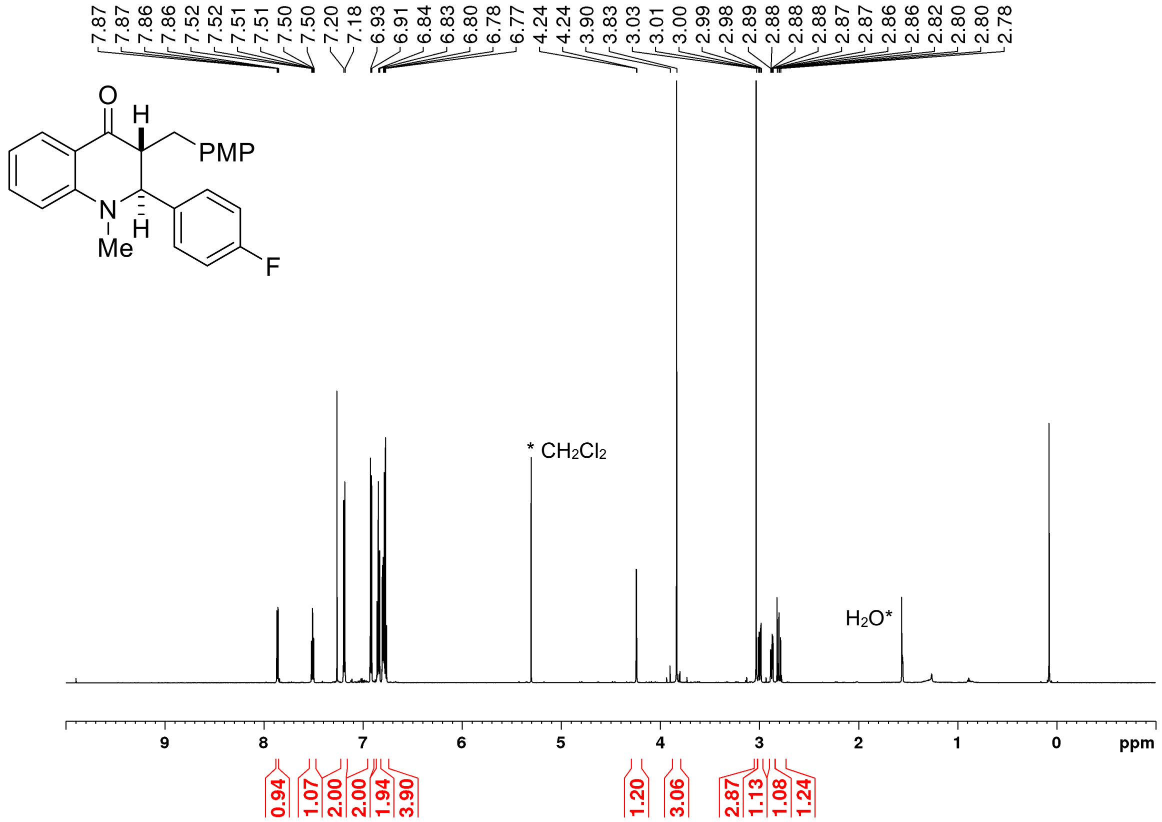

^{13}C-NMR (176 MHz, 298 K, $CDCl_3$)

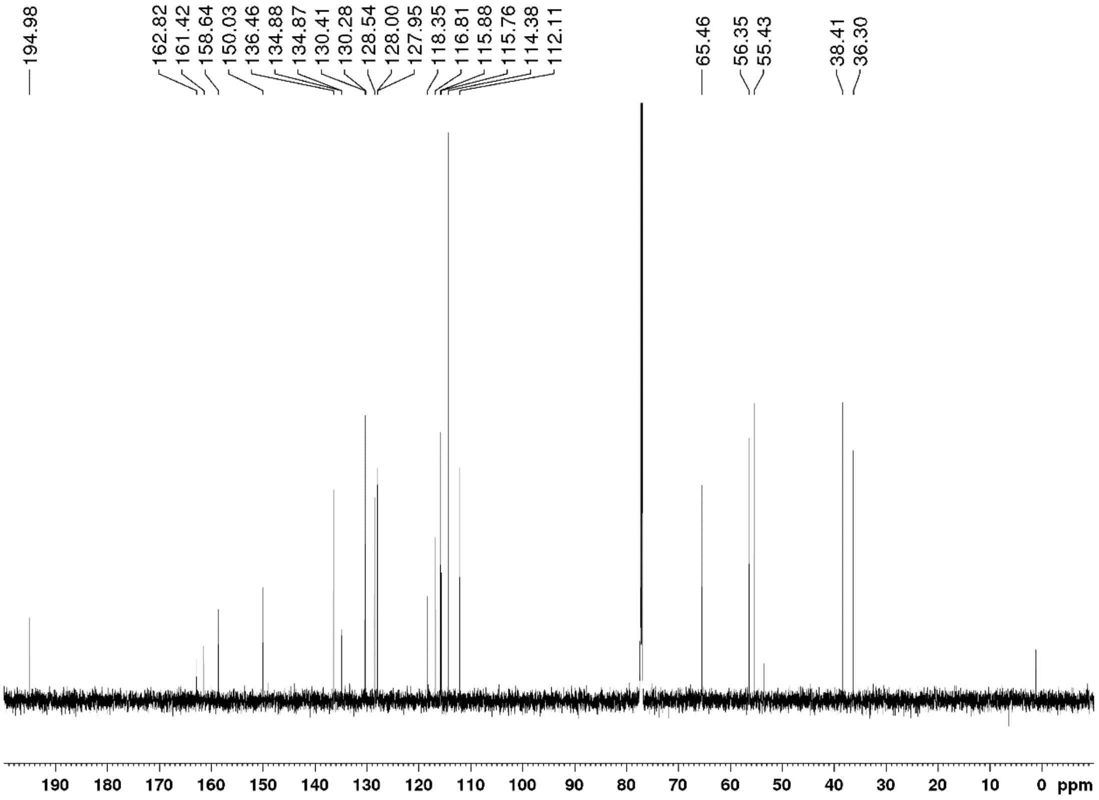

^{1}H-NMR (700 MHz, 298 K, $CDCl_3$) (**2e**)

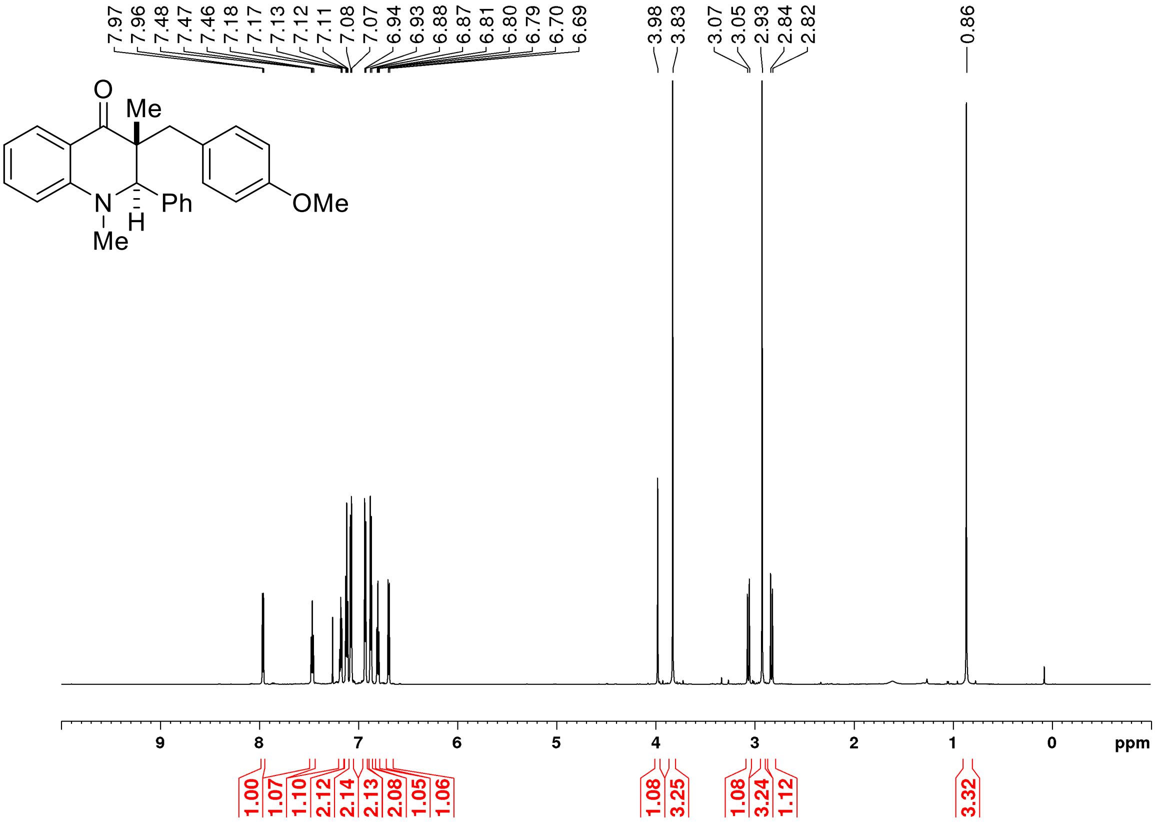

^{13}C-NMR (176 MHz, 298 K, $CDCl_3$)

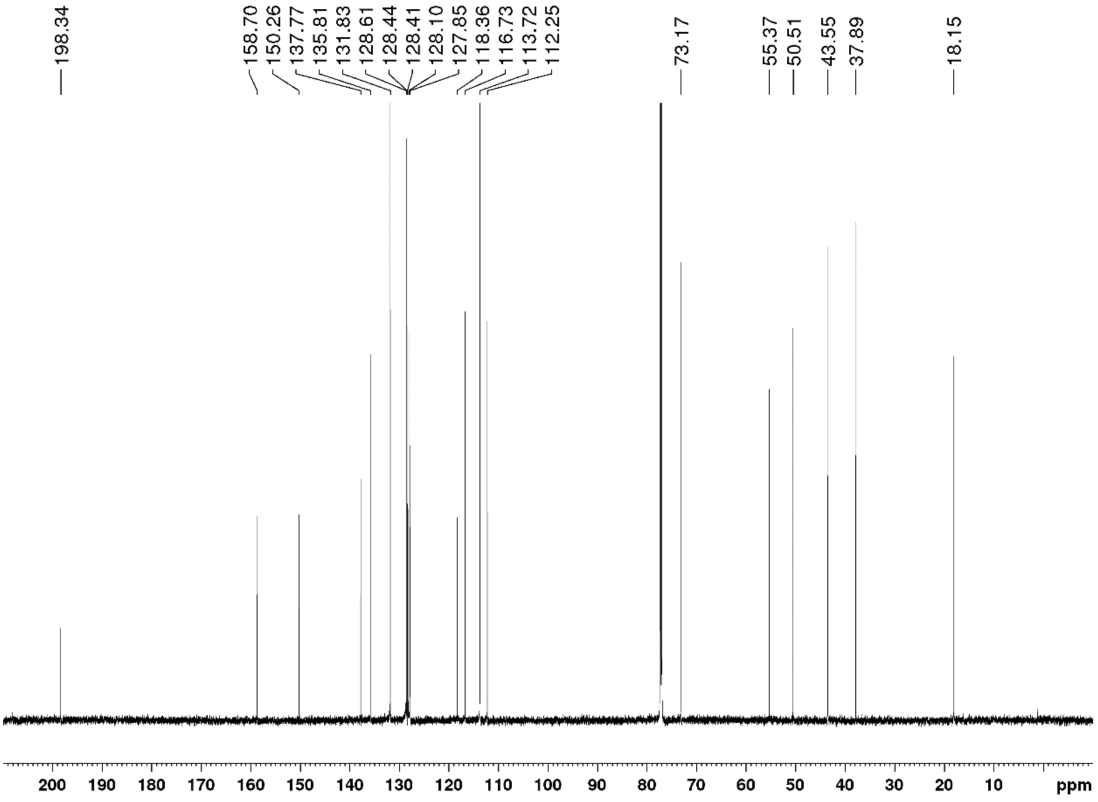

[1]**H-NMR** (700 MHz, 298 K, $CDCl_3$) (**2f**)

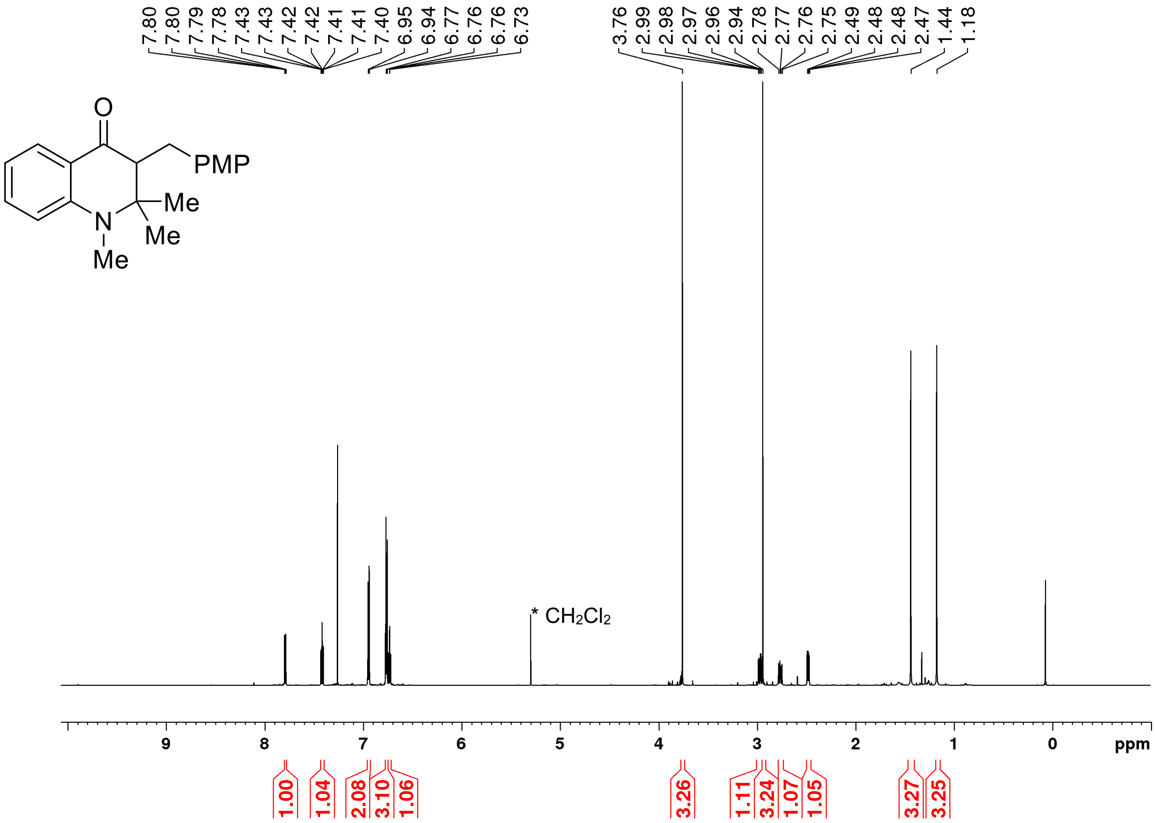

[13]**C-NMR** (176 MHz, 298 K, $CDCl_3$)

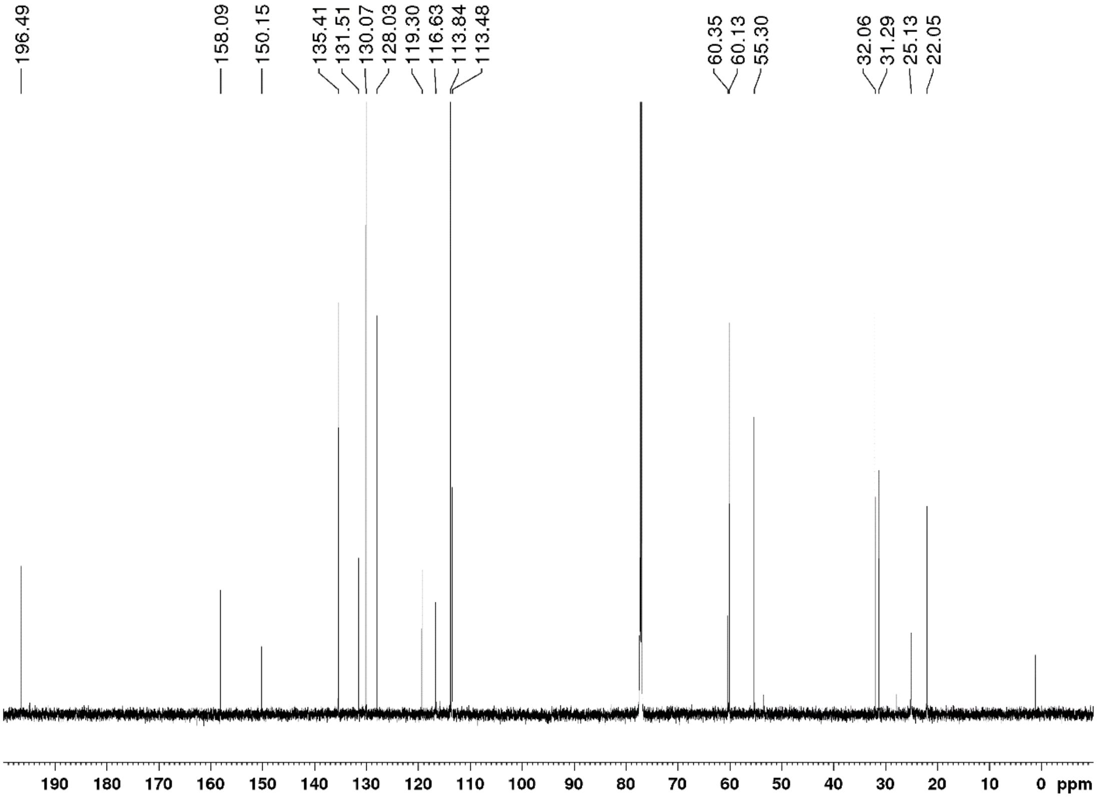

^{1}H-NMR (700 MHz, 298 K, $CDCl_3$) (2g)

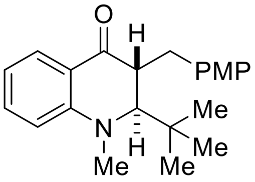

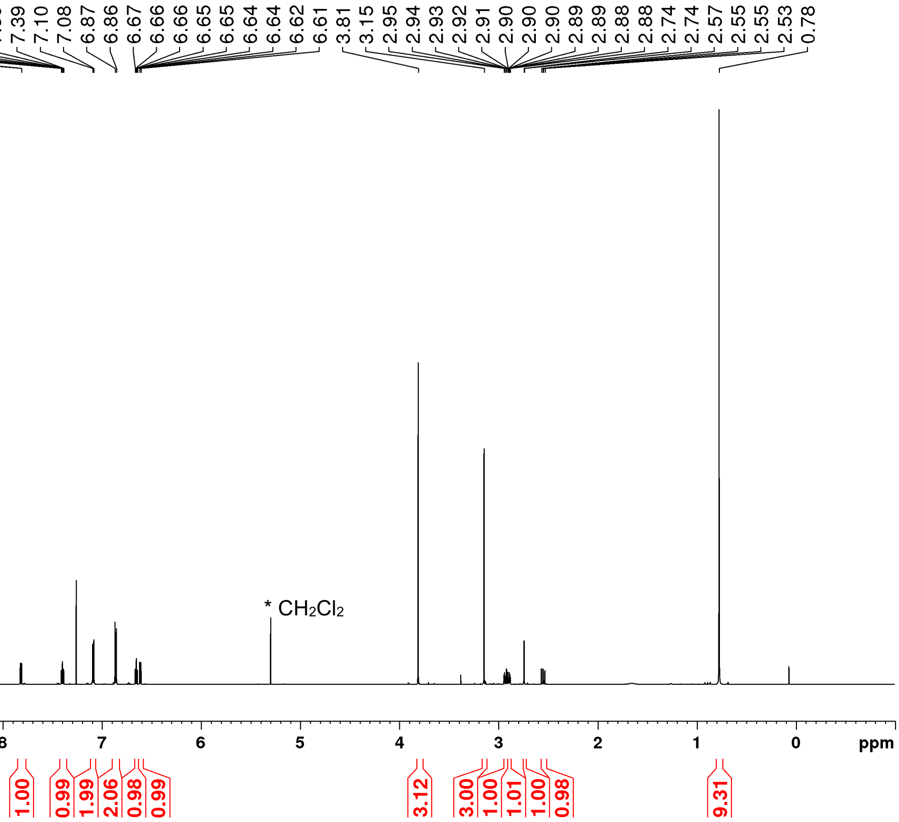

^{13}C-NMR (176 MHz, 298 K, $CDCl_3$)

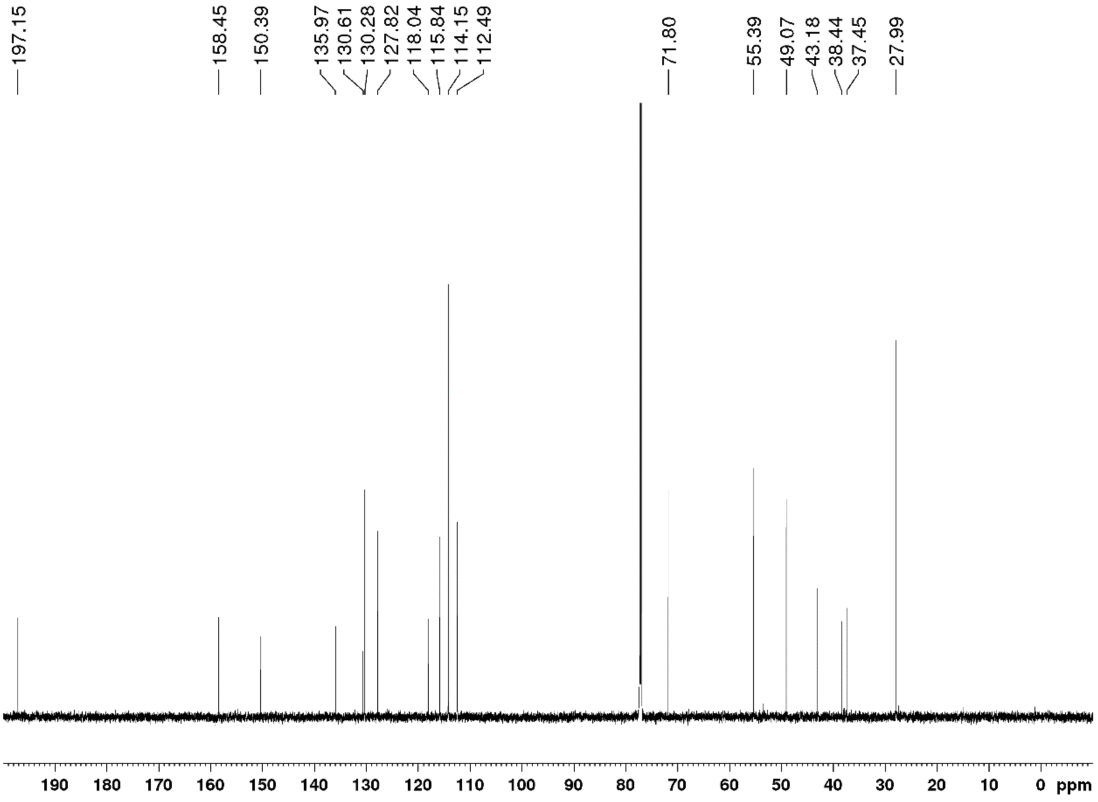

^{1}H-NMR (700 MHz, 298 K, $CDCl_3$) (**2h**)

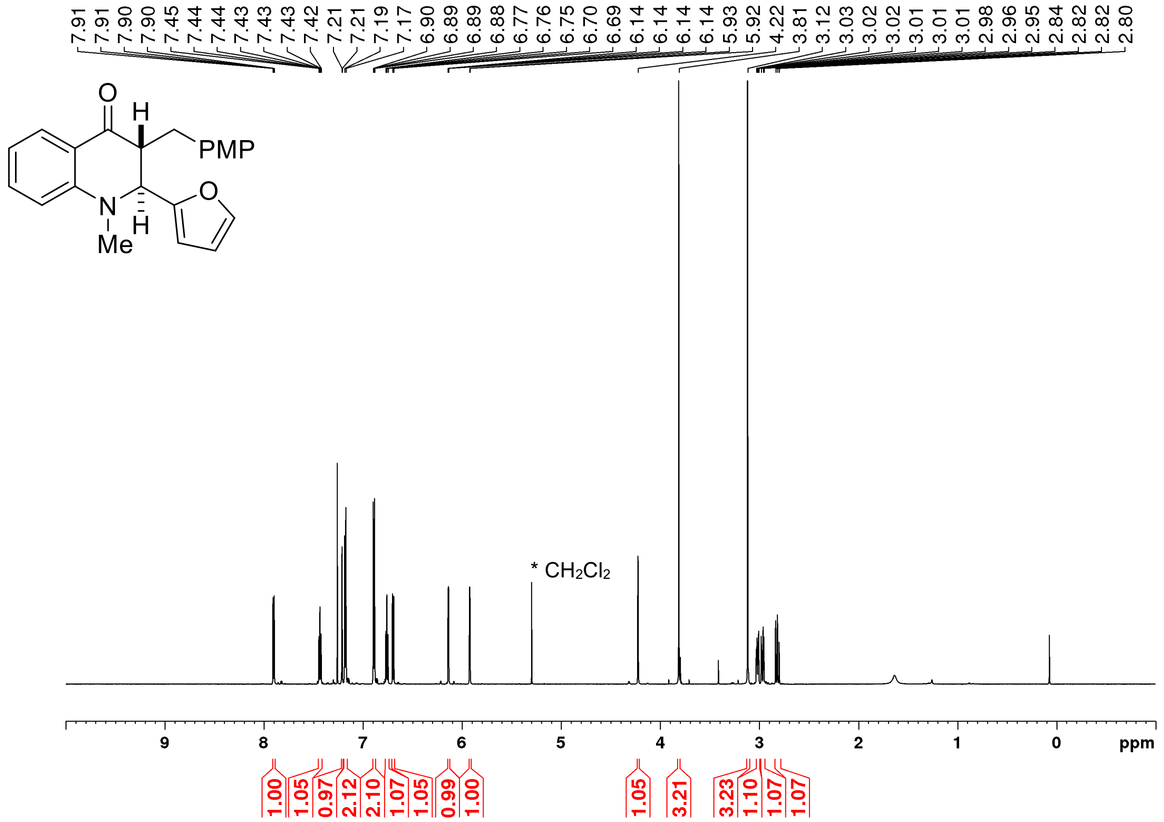

^{13}C-NMR (176 MHz, 298 K, $CDCl_3$)

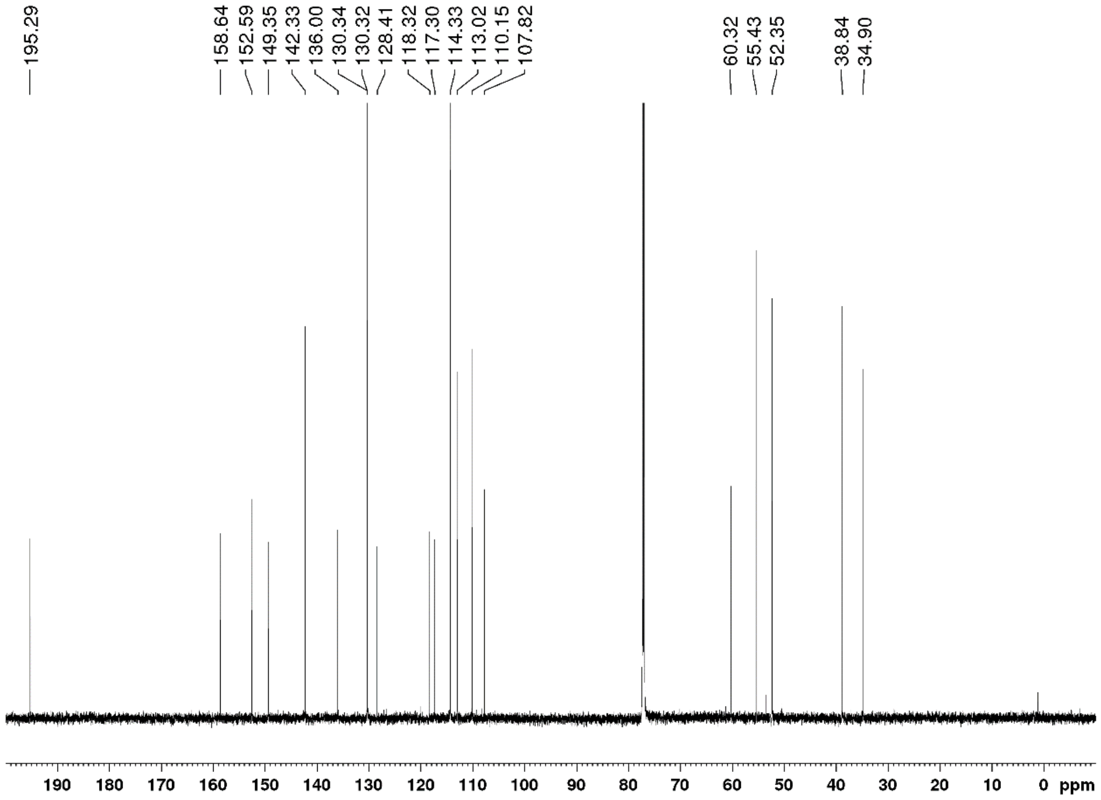

^{1}H-NMR (700 MHz, 298 K, $CDCl_3$) (**2i**)

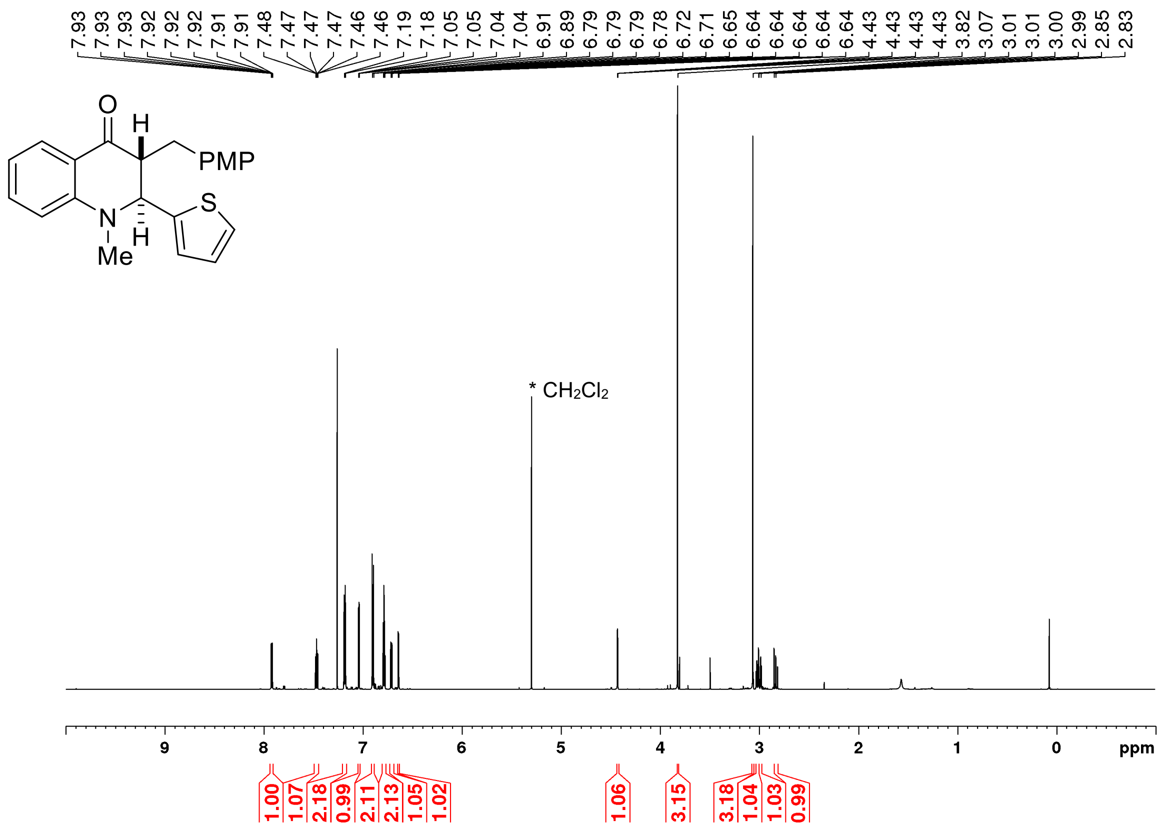

^{13}C-NMR (176 MHz, 298 K, $CDCl_3$)

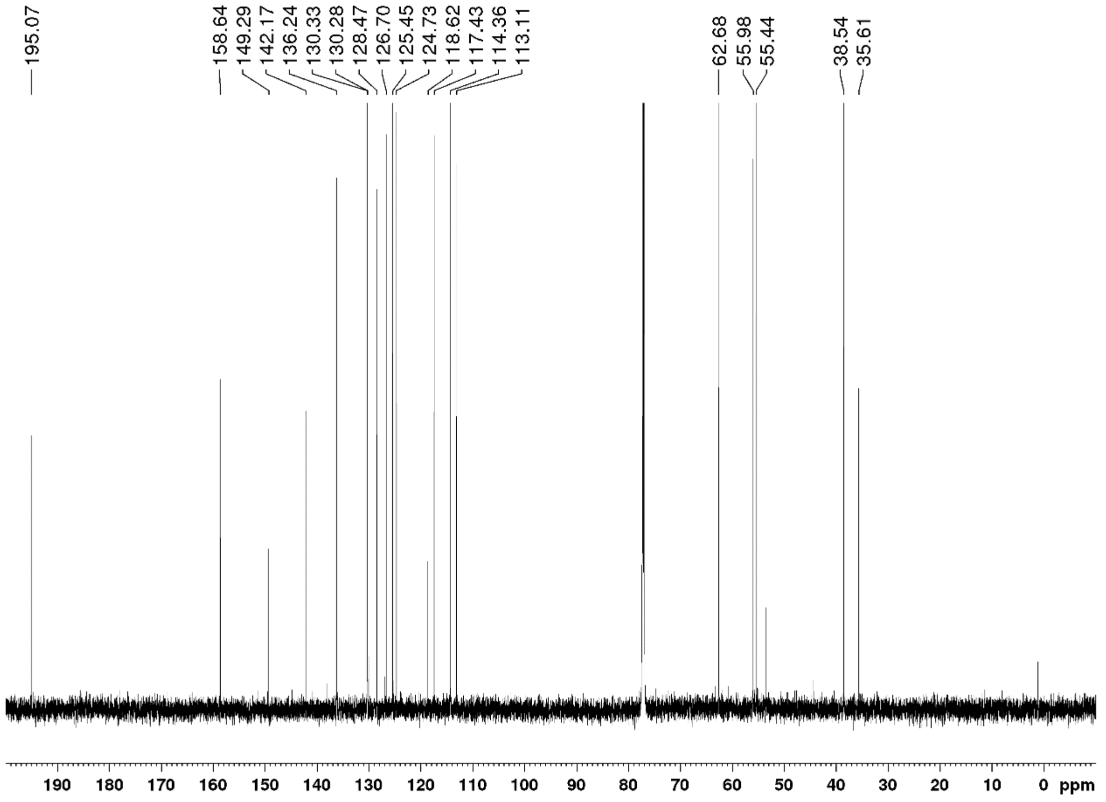

^{1}H-NMR (700 MHz, 298 K, $CDCl_3$) (**2j**)

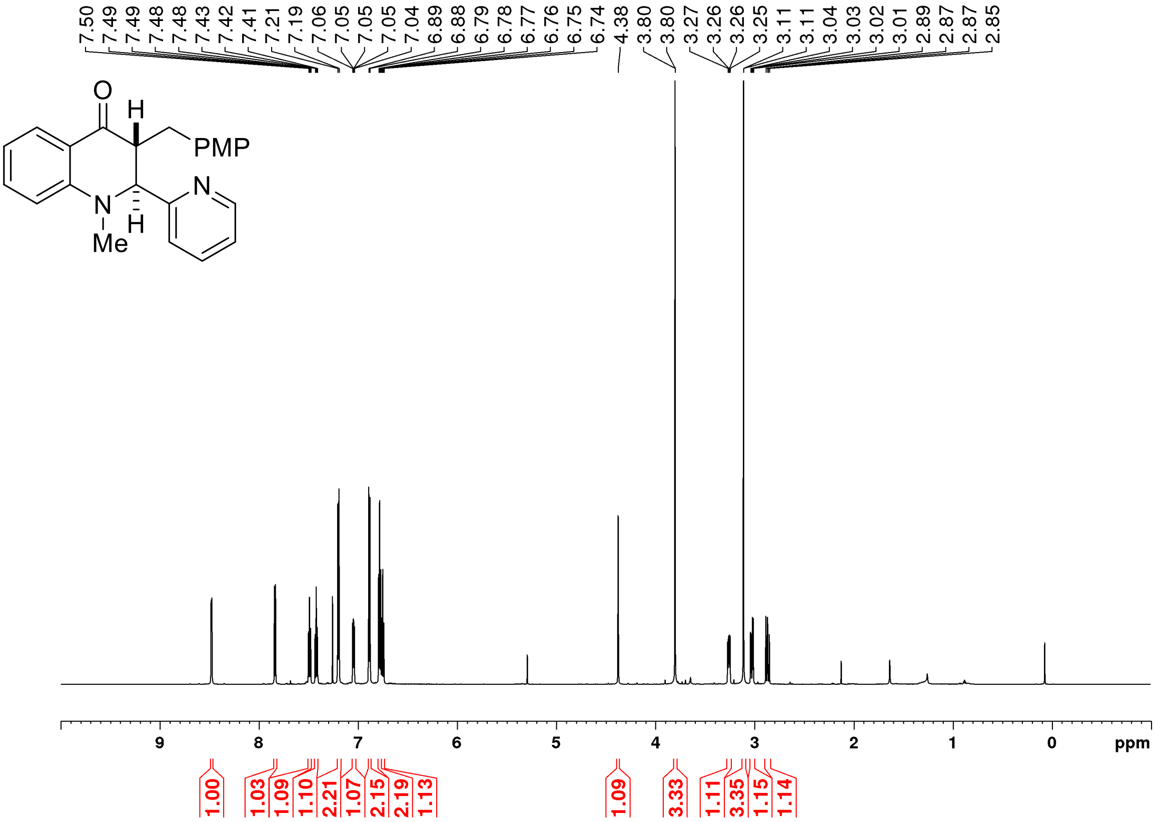

^{13}C-NMR (176 MHz, 298 K, $CDCl_3$)

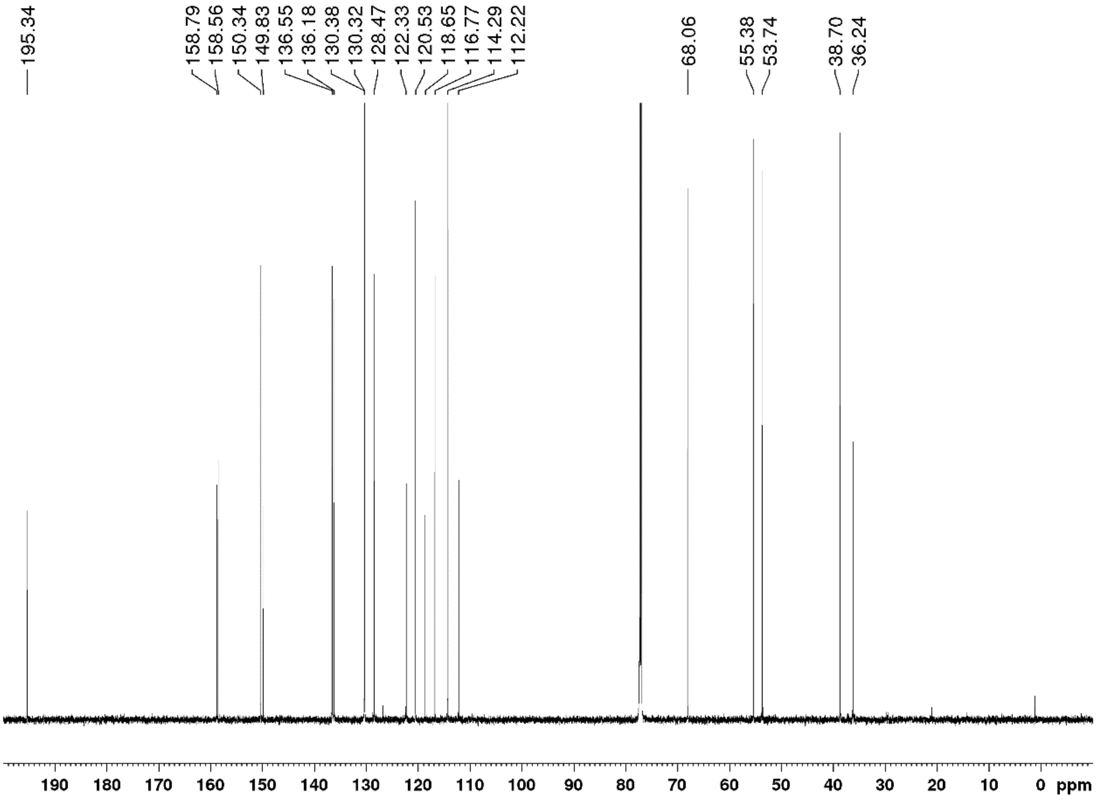

^{1}H-NMR (700 MHz, 298 K, $CDCl_3$) (**2k**)

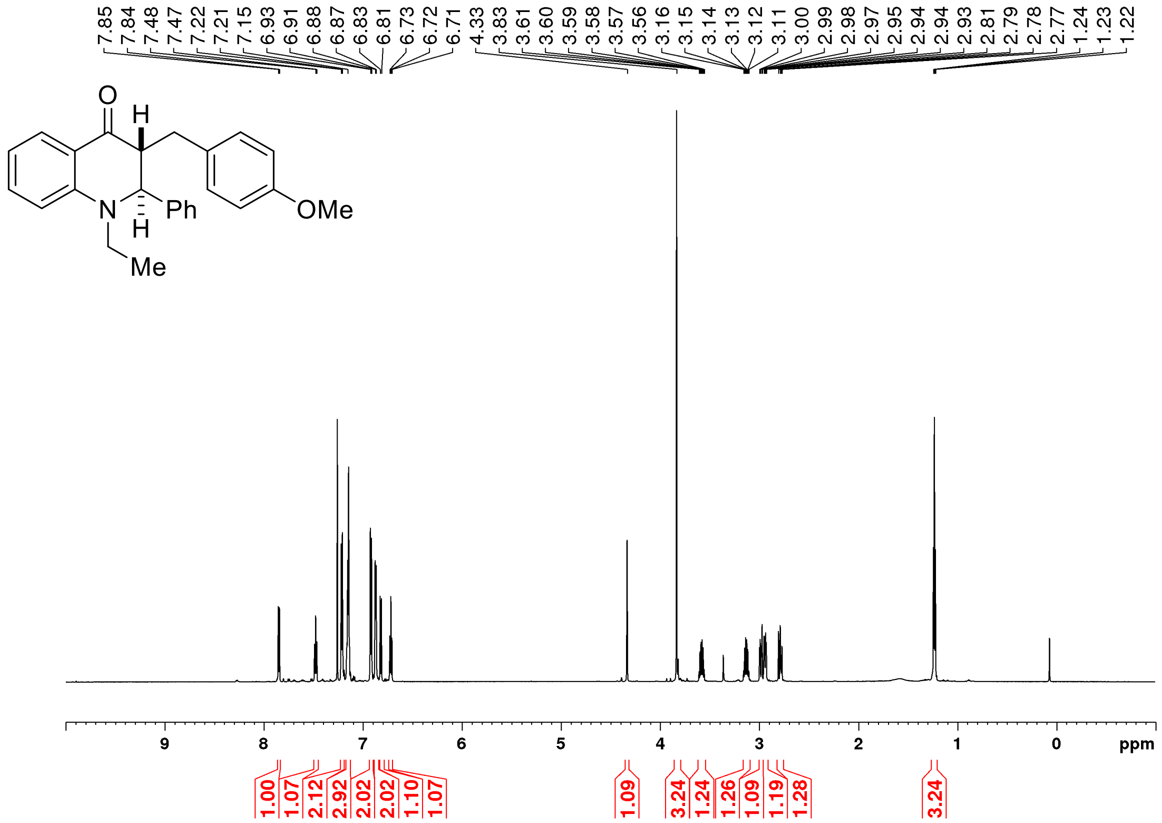

^{13}C-NMR (176 MHz, 298 K, $CDCl_3$)

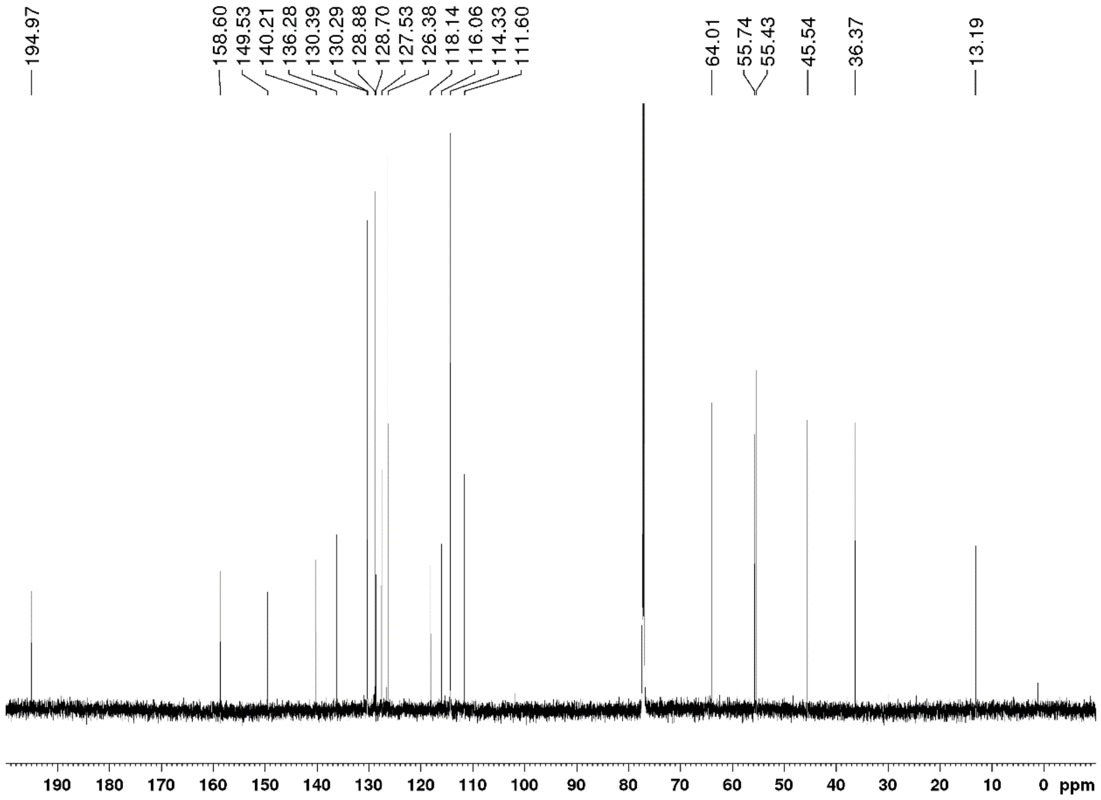

^{1}H-NMR (700 MHz, 298 K, $CDCl_3$) (2l)

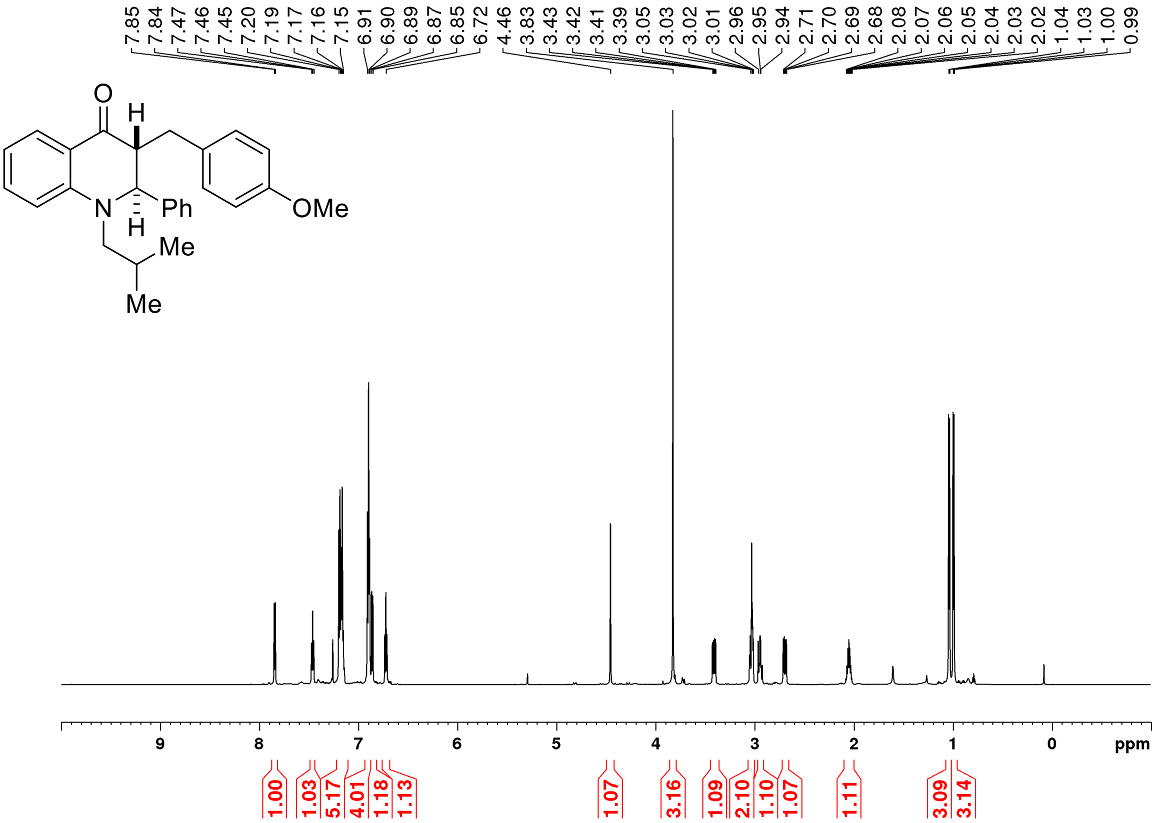

^{13}C-NMR (176 MHz, 298 K, $CDCl_3$)

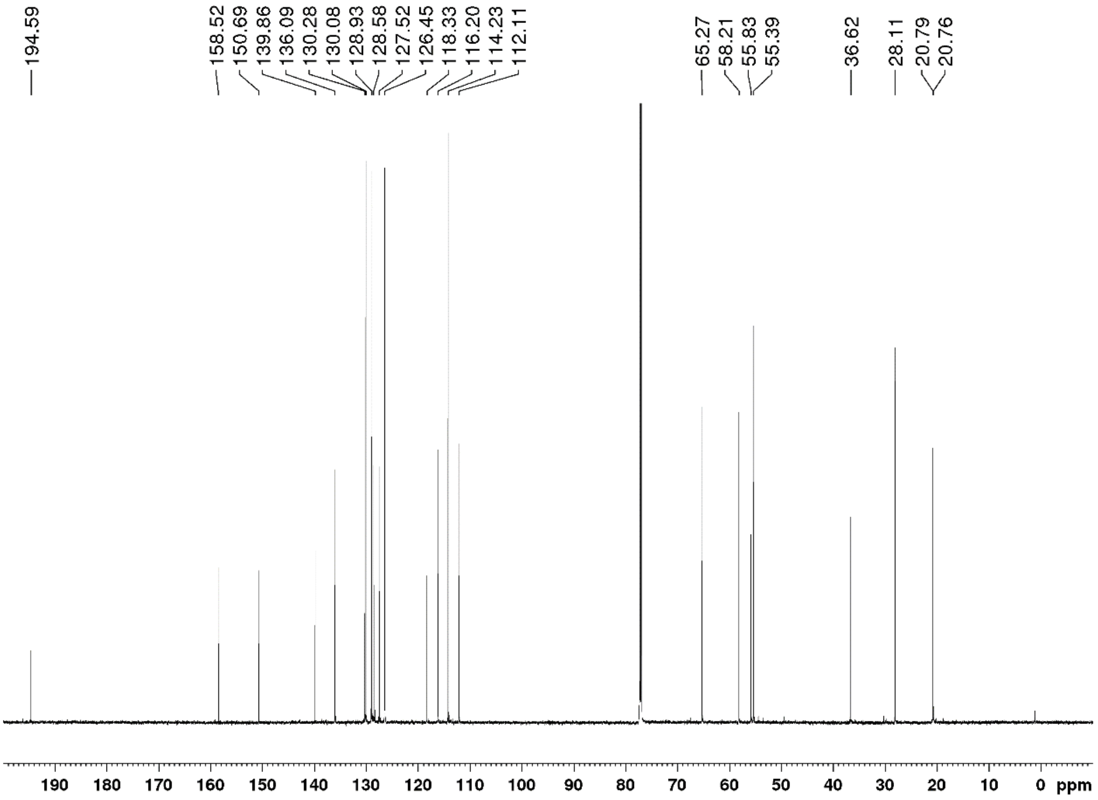

^{1}**H-NMR** (700 MHz, 298 K, CD_2Cl_2) (*rac*-**2m**)

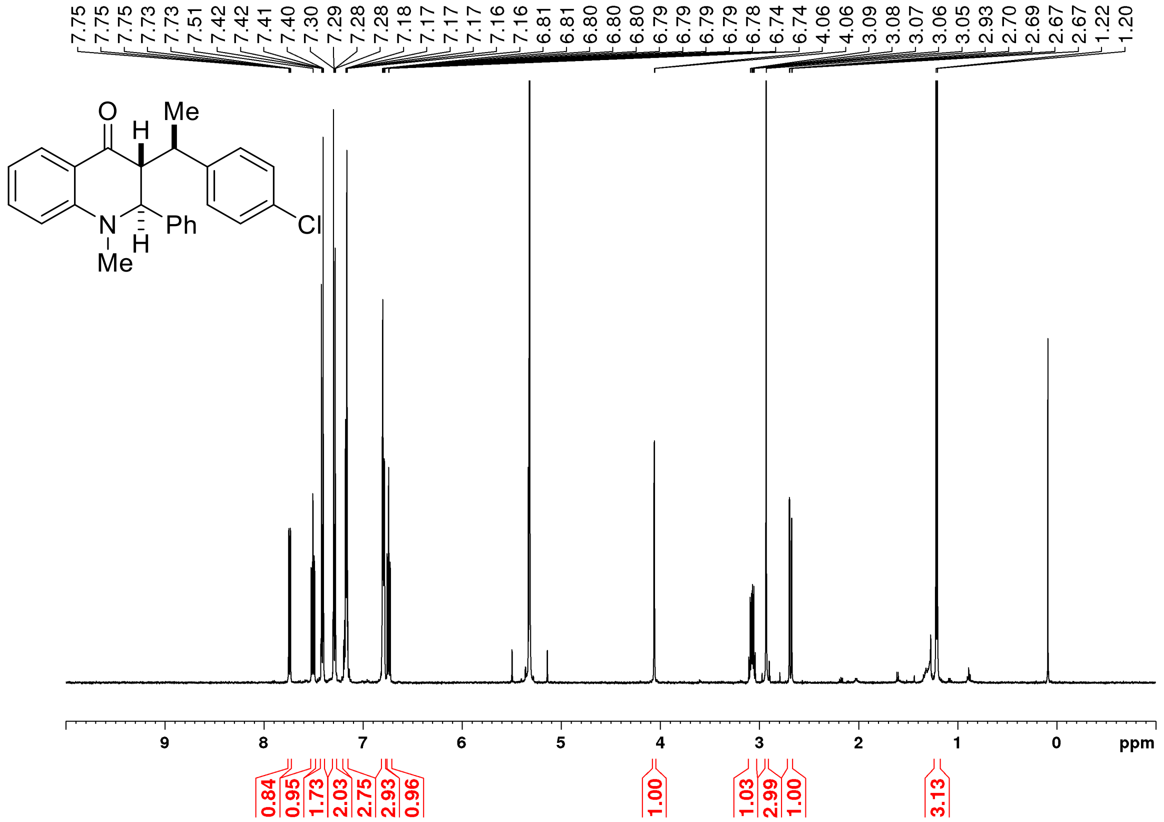

^{13}**C-NMR** (176 MHz, 298 K, CD_2Cl_2)

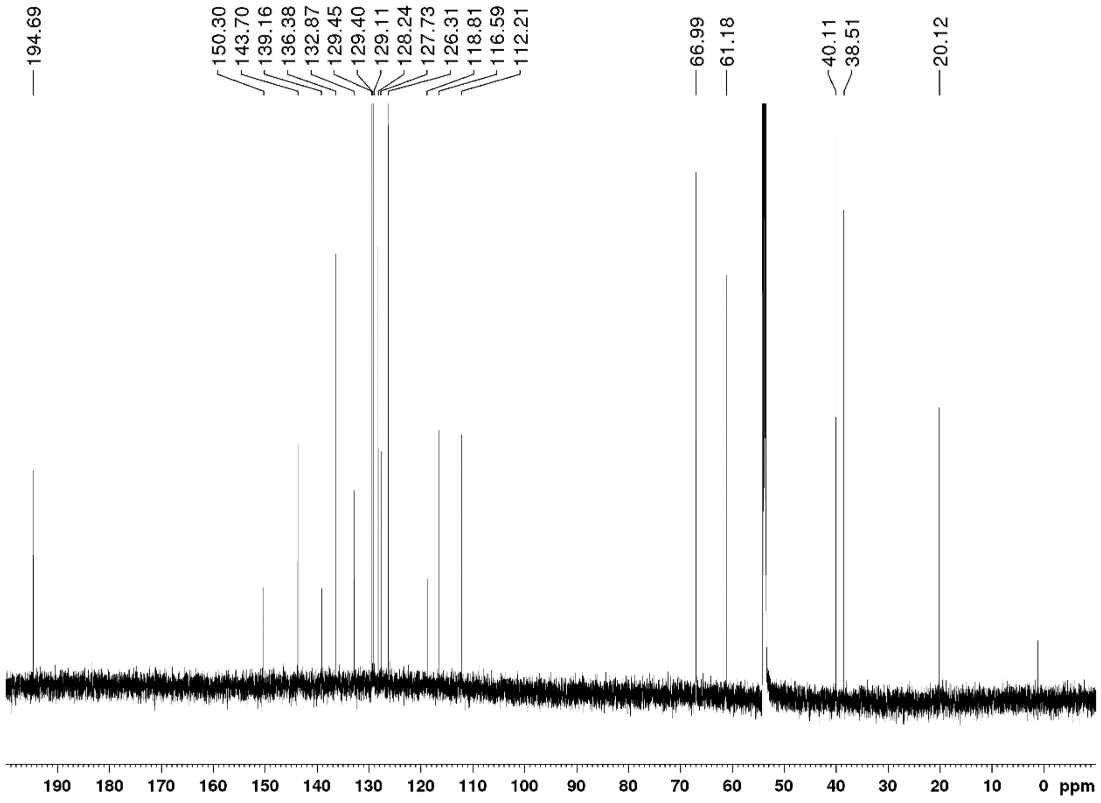

^{1}H-NMR (700 MHz, 298 K, $CDCl_3$) (*rac*-**2n**)

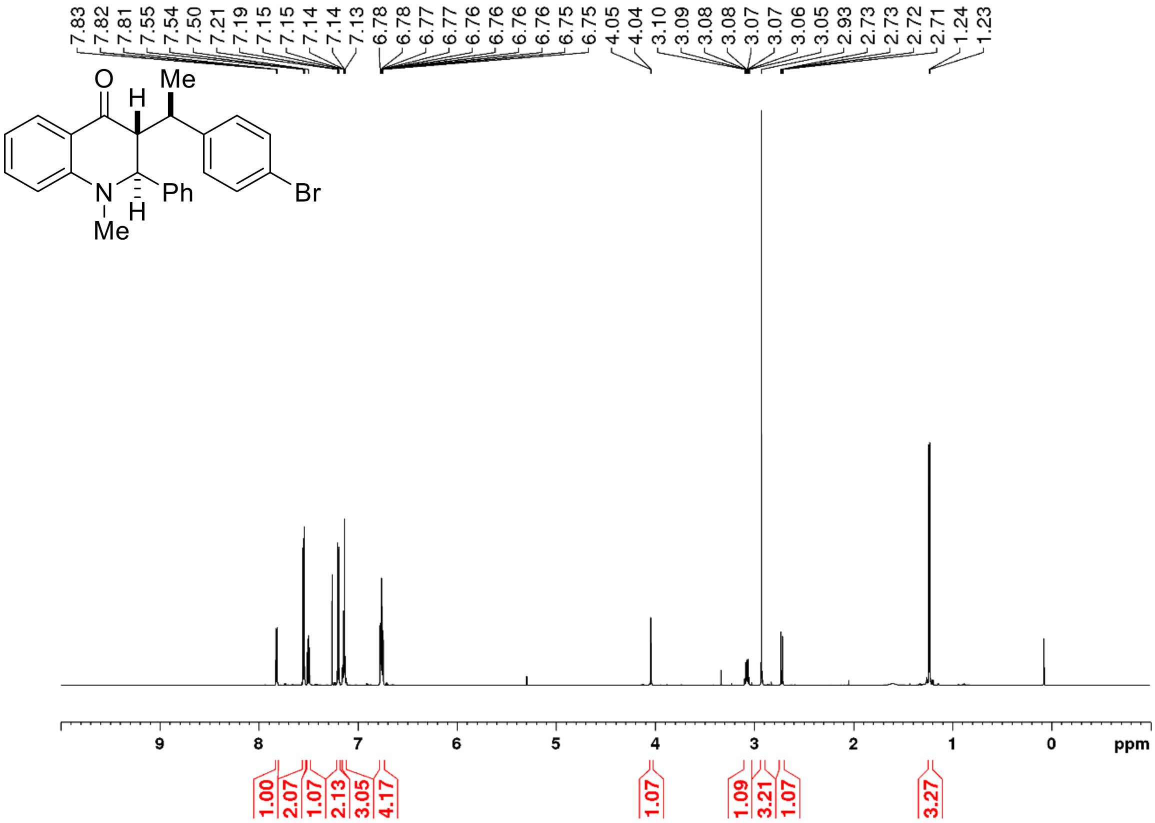

^{13}C-NMR (176 MHz, 298 K, $CDCl_3$)

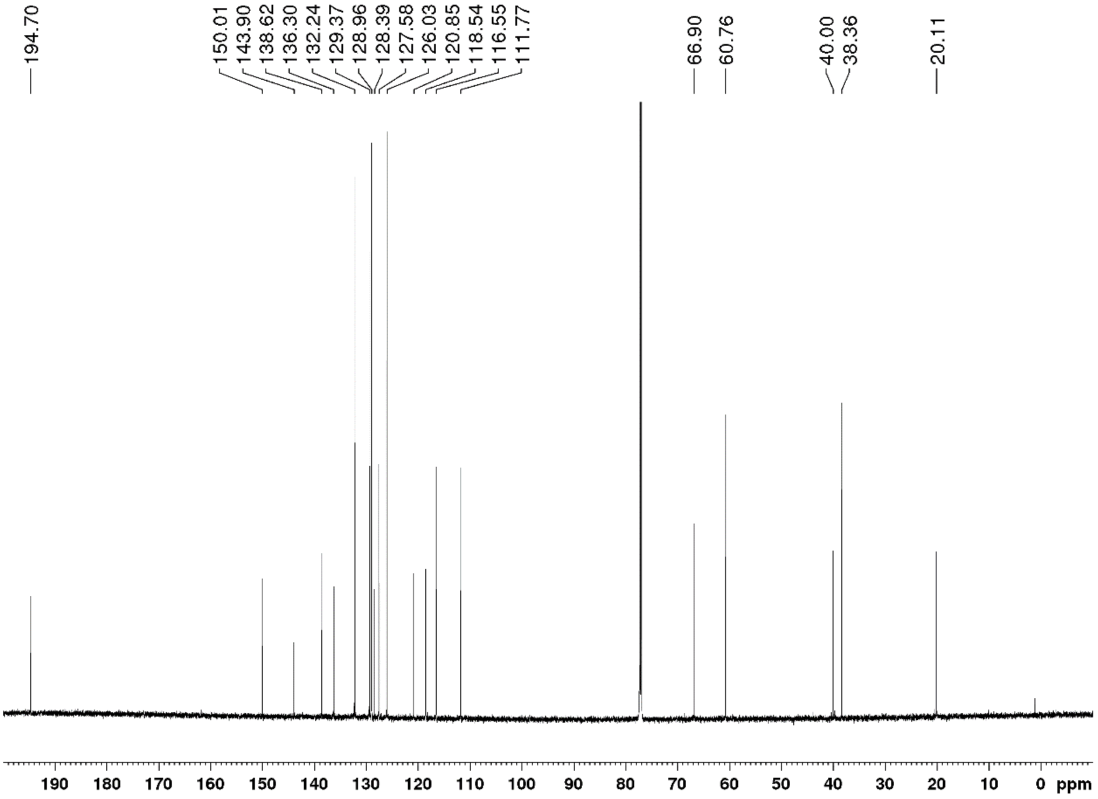

^{1}H-NMR (700 MHz, 298 K, $CDCl_3$) ((*2S,3R*)-**2n**)

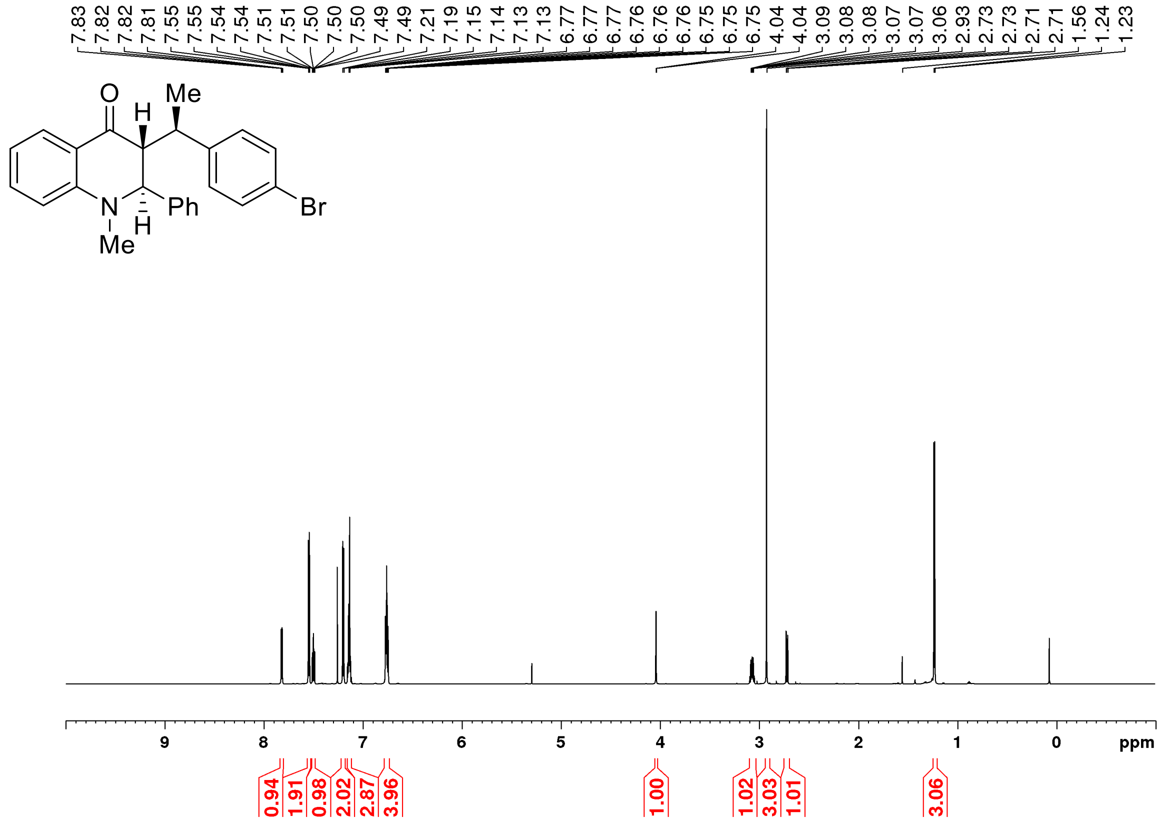

^{13}C-NMR (176 MHz, 298 K, $CDCl_3$)

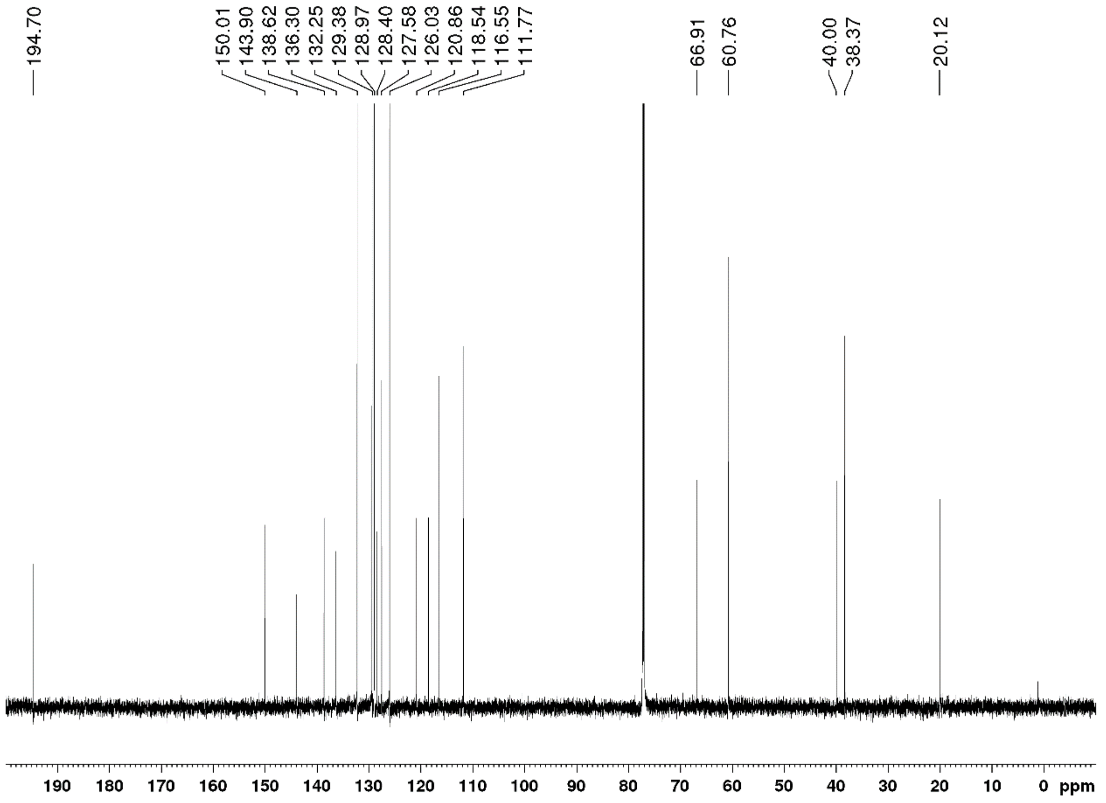

^{1}H-NMR (700 MHz, 298 K, $CDCl_3$) (*rac*-**2o**)

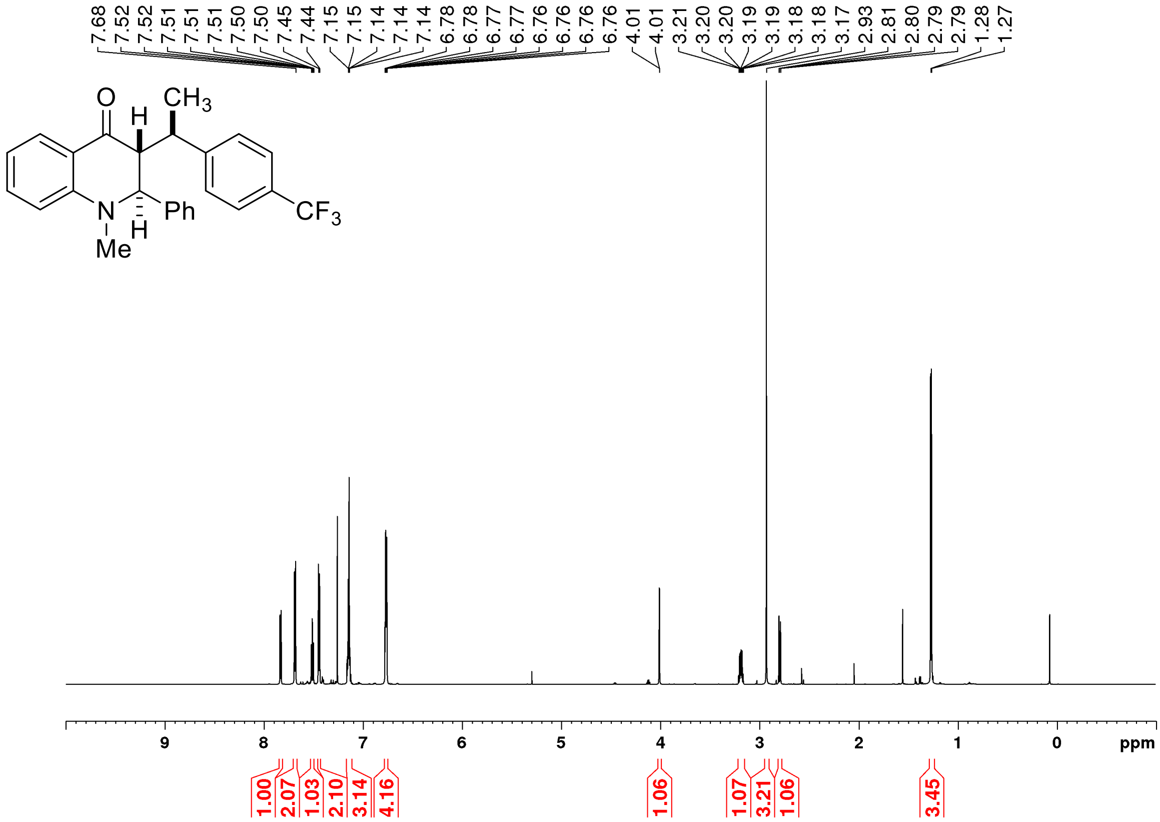

^{13}C-NMR (176 MHz, 298 K, $CDCl_3$)

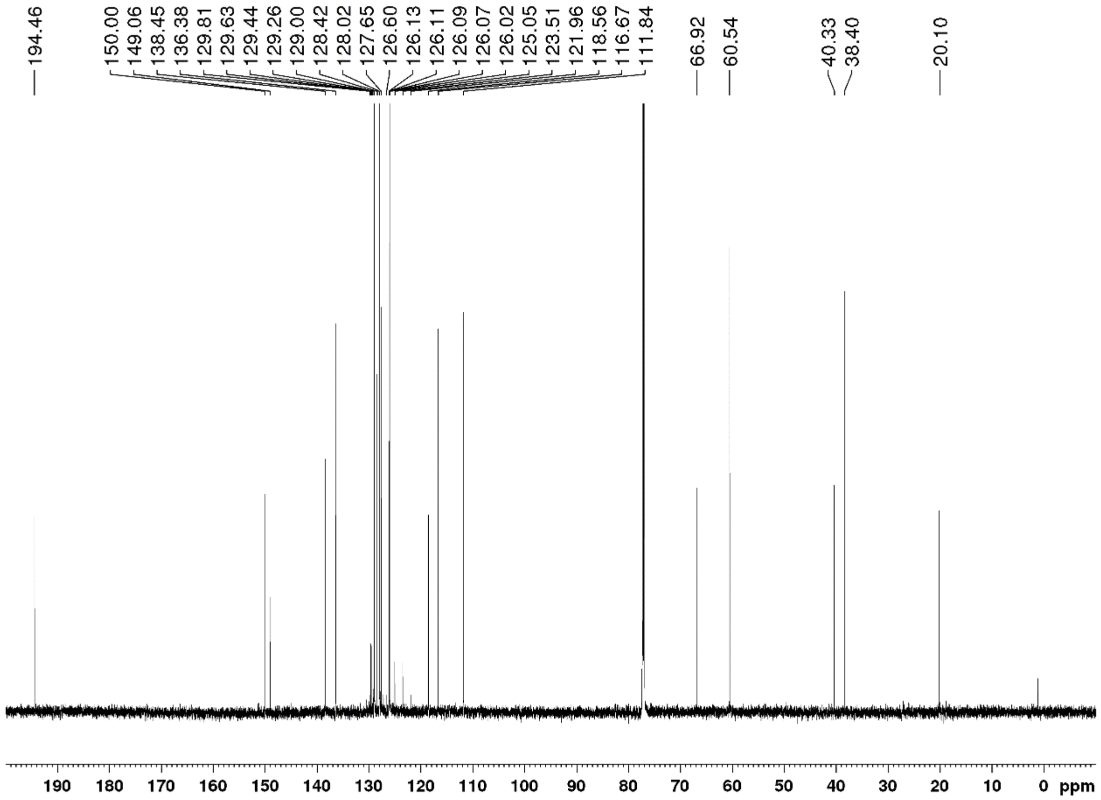

[1]**H-NMR** (700 MHz, 298 K, $CDCl_3$) (**2p**)

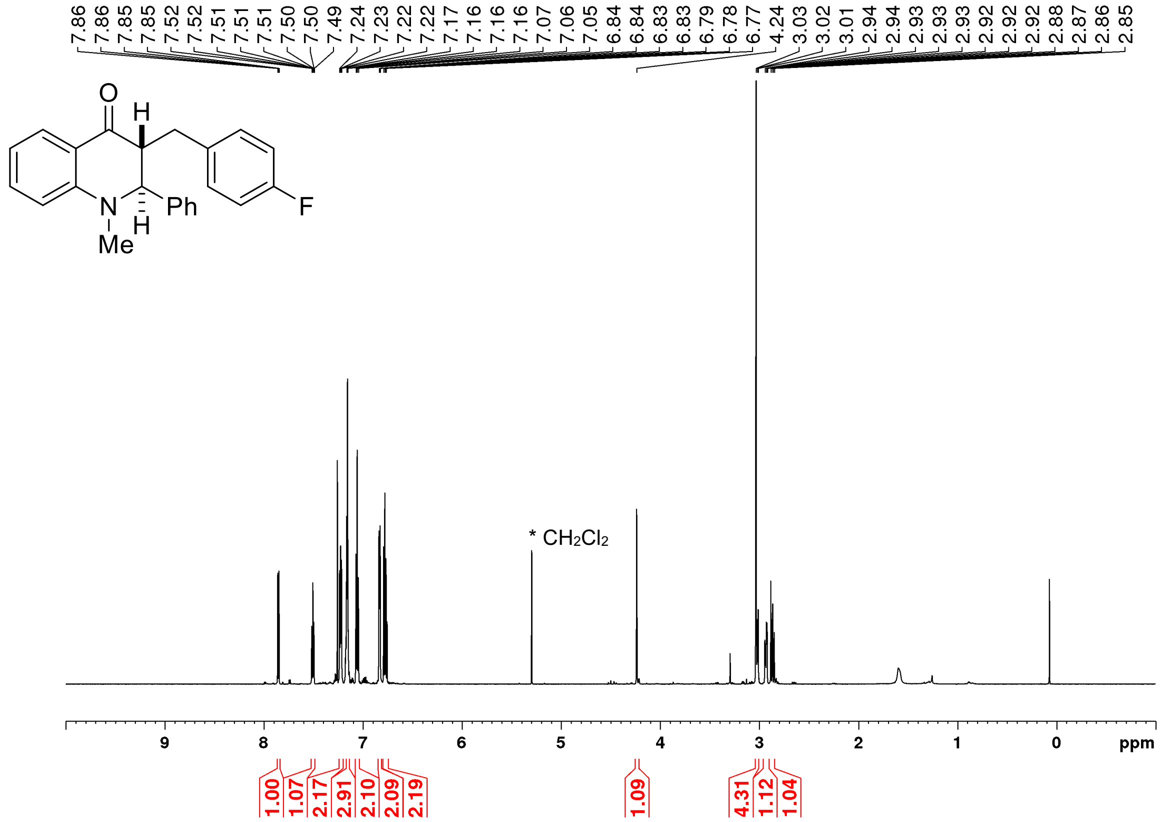

[13]**C-NMR** (176 MHz, 298 K, $CDCl_3$)

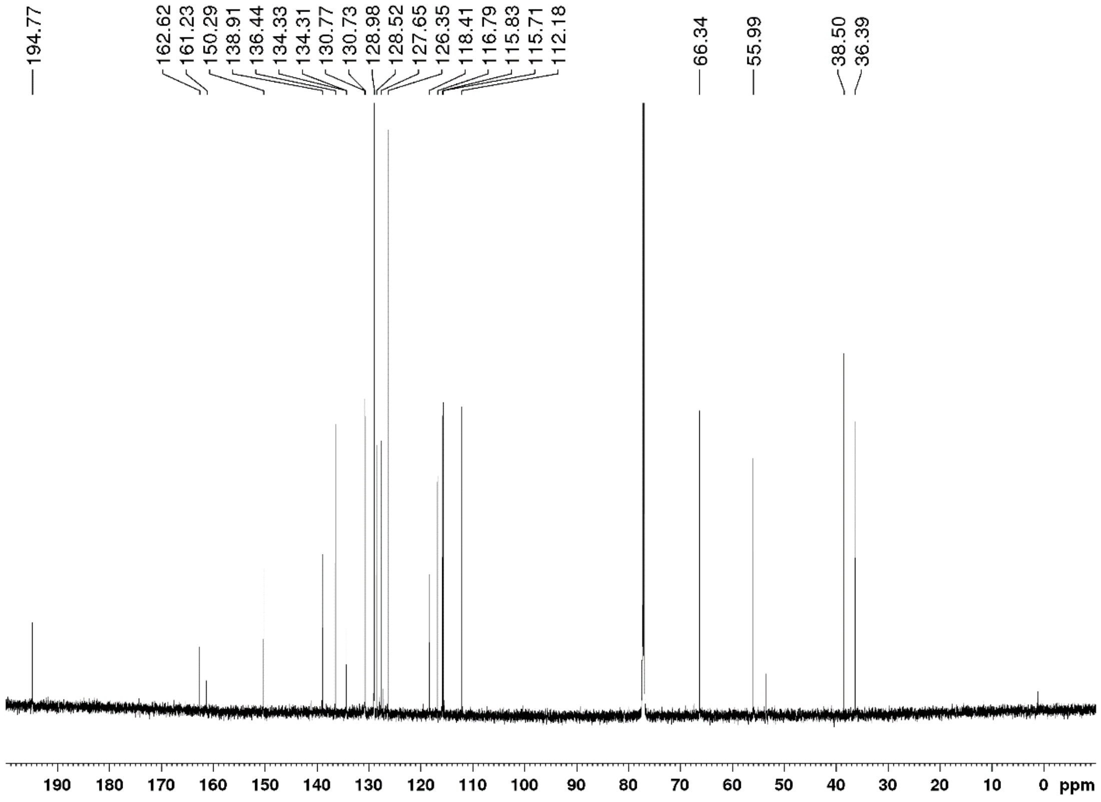

^{1}H-NMR (700 MHz, 298 K, $CDCl_3$) (**2q**)

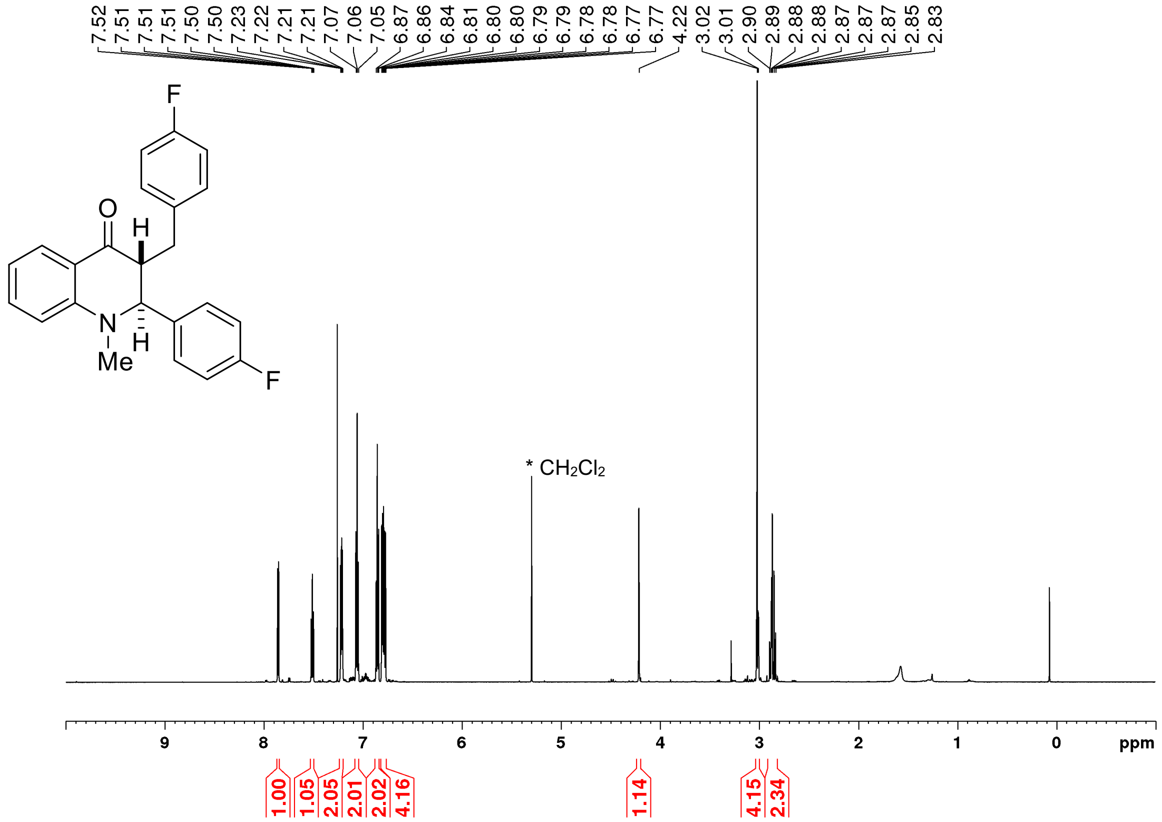

^{13}C-NMR (176 MHz, 298 K, $CDCl_3$)

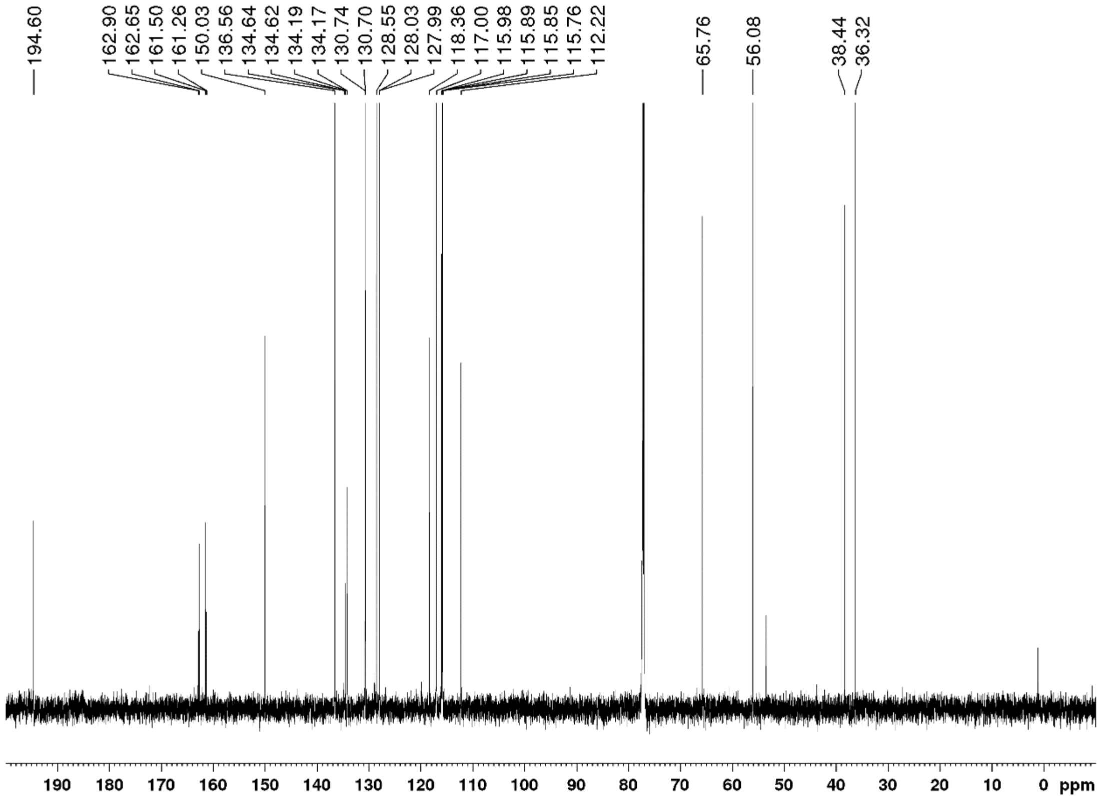

^{1}H-NMR (700 MHz, 298 K, $CDCl_3$) (**2r**)

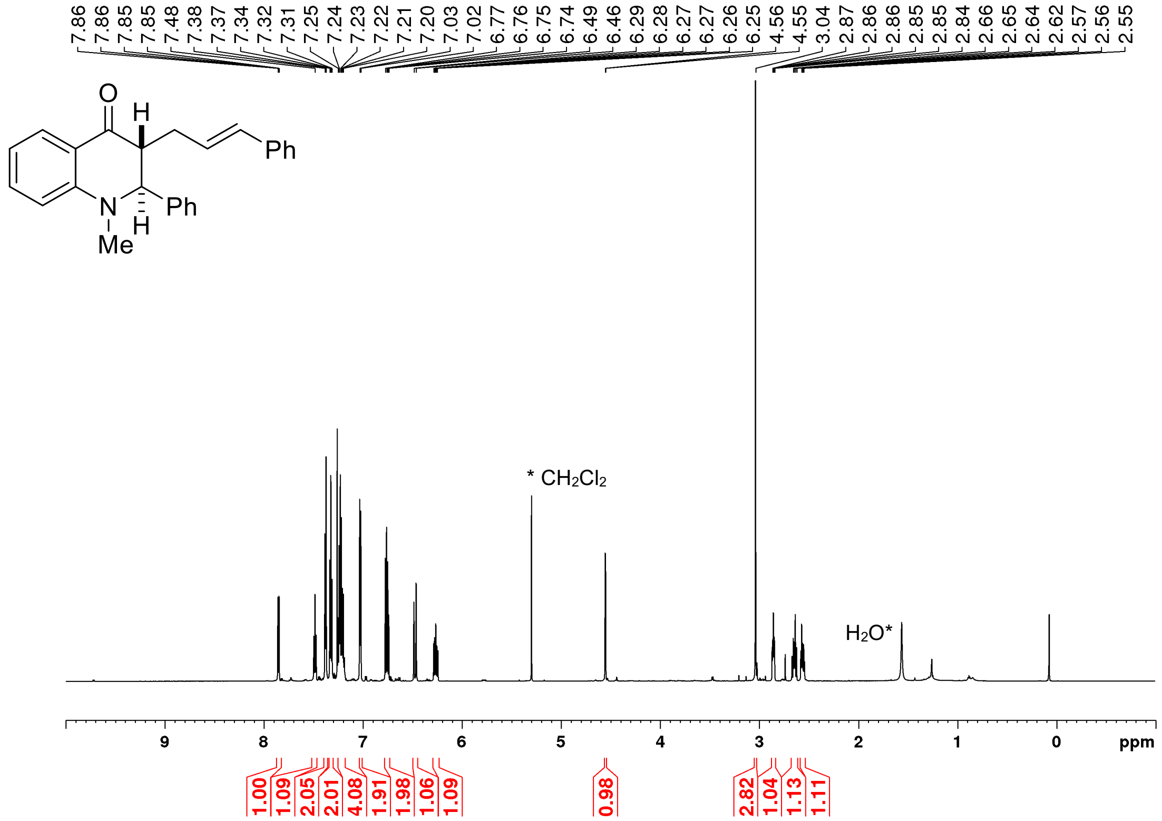

^{13}C-NMR (176 MHz, 298 K, $CDCl_3$)

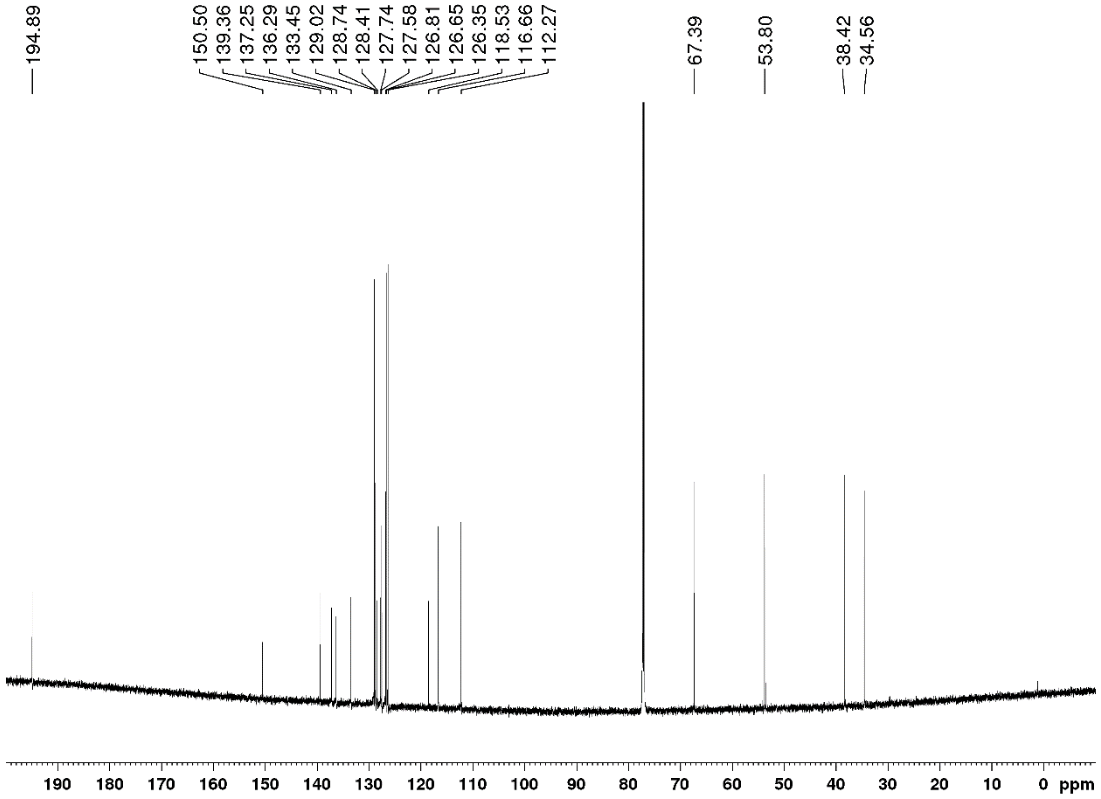

^{1}**H-NMR** (700 MHz, 298 K, $CDCl_3$) (**2s**)

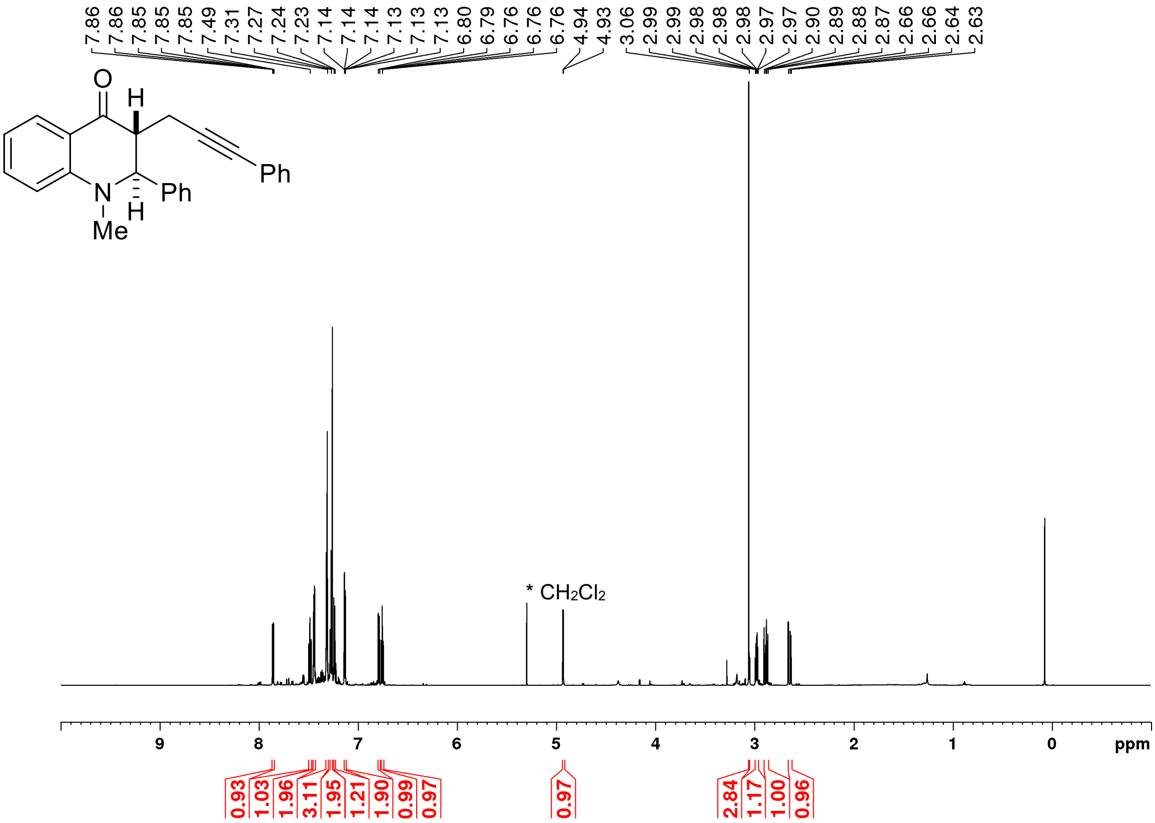

^{13}**C-NMR** (176 MHz, 298 K, $CDCl_3$)

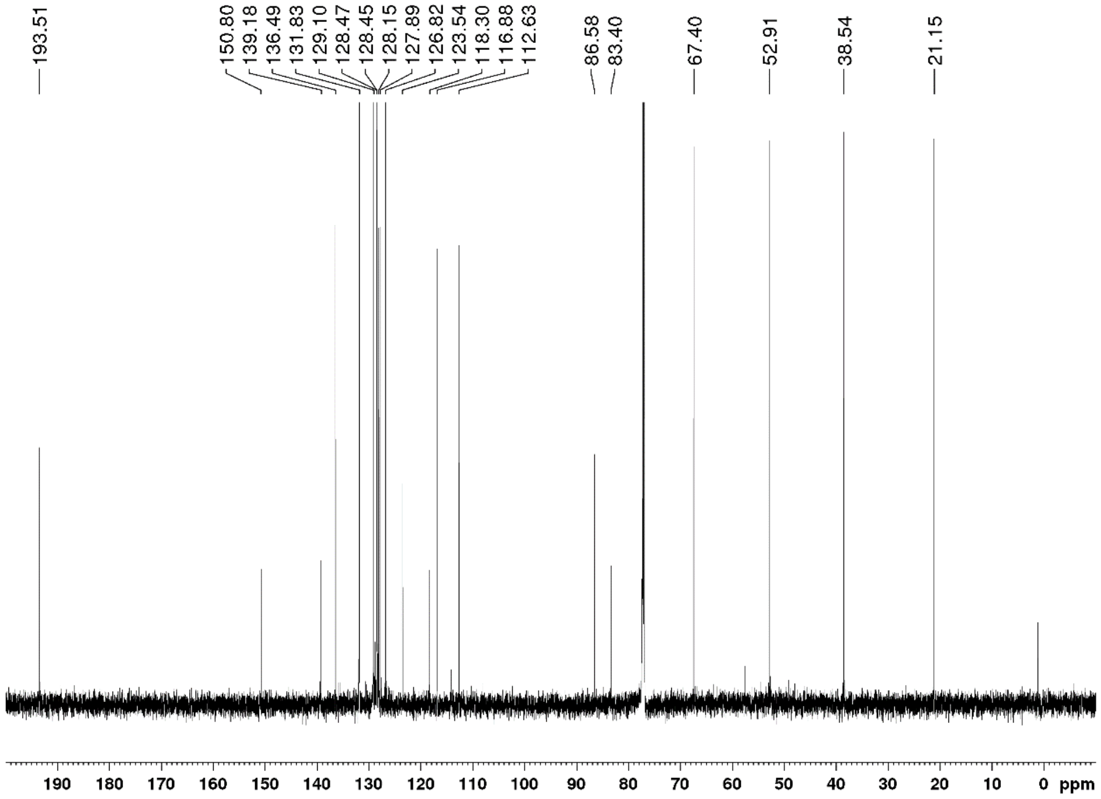

4 UV/Vis and luminescence spectra

rac-trans-2,3-1-methyl-2-phenyl-3-(*cis*-1-phenylethyl)-2,3-dihydroquinolin-4(1H)-one (*rac*-**2a)**

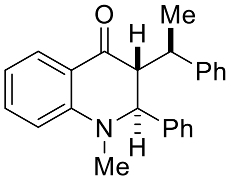

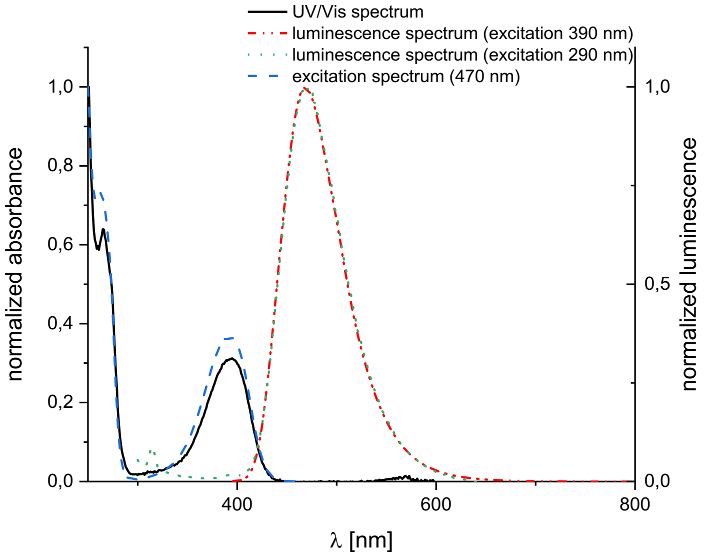

rac-trans-2,3-1-methyl-2-phenyl-3-(*cis*-1-phenylethyl-1-d)-2,3-dihydroquinolin-4(1H)-one (d_1-*rac*-**2a**)

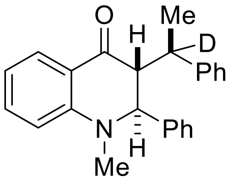

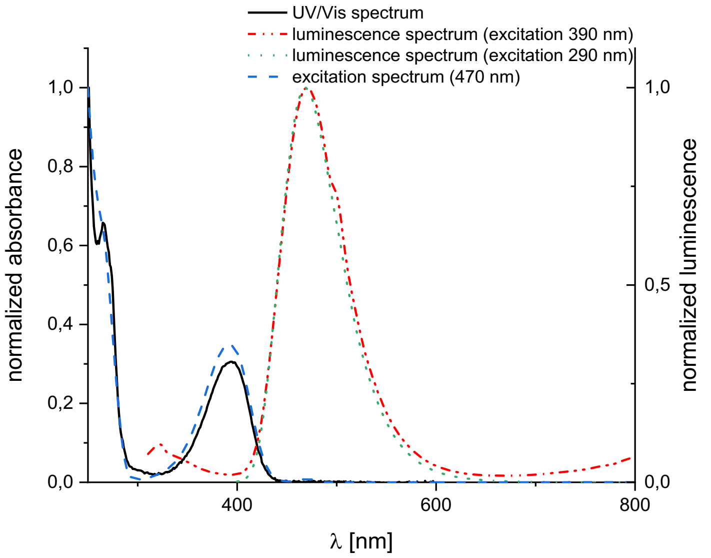

(2*S*,3*R*)-1-methyl-2-phenyl-3-((*R*)-1-phenylethyl)-2,3-dihydroquinolin-4(1H)-one (*S*)-**2a**

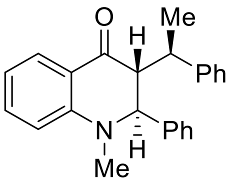

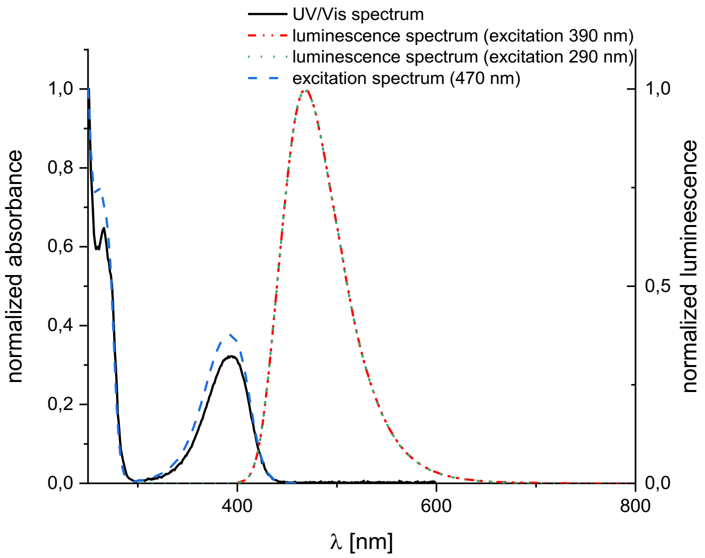

rac-trans-2,3-3-(4-methoxybenzyl)-1-methyl-2-phenyl-2,3-dihydroquinolin-4(1H)-one (**2b**)

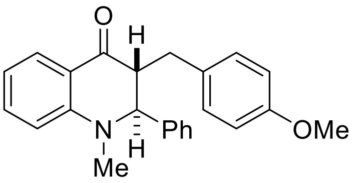

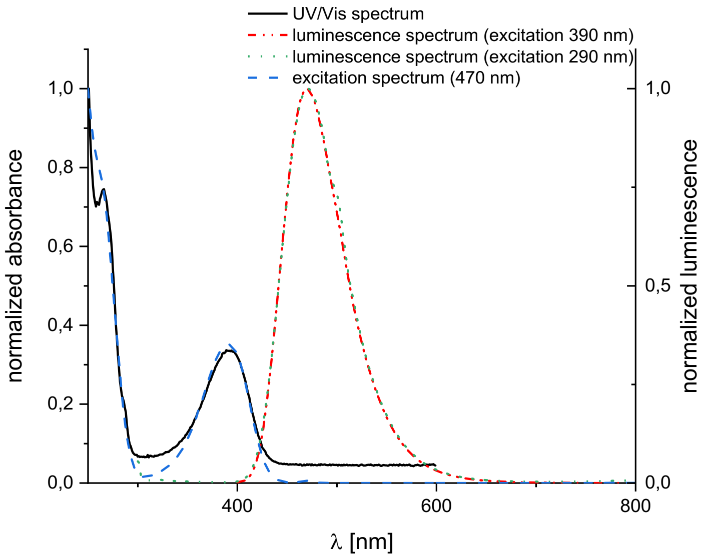

rac-trans-2,3-3-(4-methoxybenzyl)-2-(4-methoxyphenyl)-1-methyl-2,3-dihydroquinolin-4(1H)-one (**2c**)

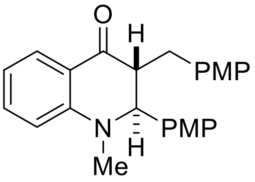

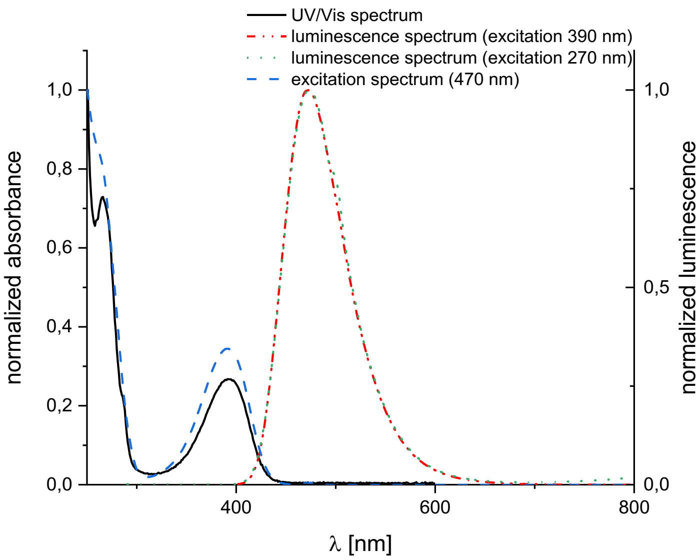

rac-trans-2,3-2-(4-fluorophenyl)-3-(4-methoxybenzyl)-1-methyl-2,3-dihydroquinolin-4(1H)-one (**2d**)

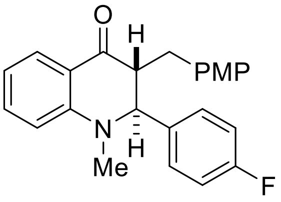

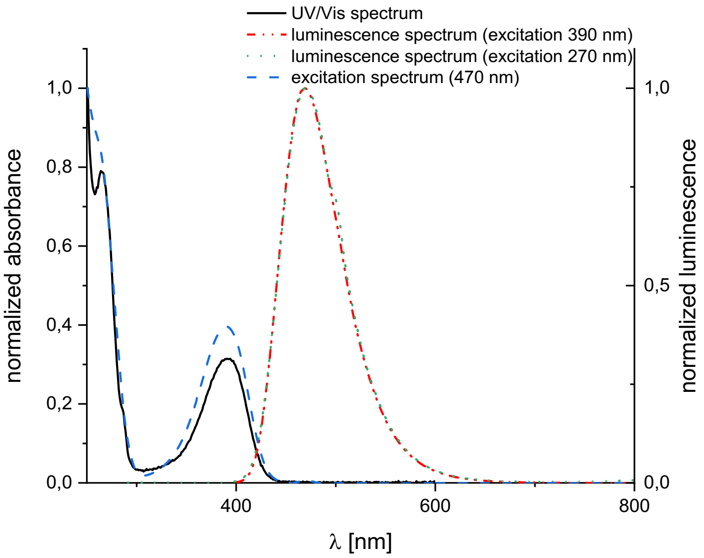

rac-trans-2,3-3-(4-methoxybenzyl)-1,3-dimethyl-2-phenyl-2,3-dihydroquinolin-4(1H)-one (**2e**)

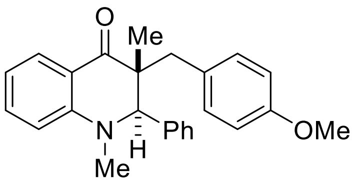

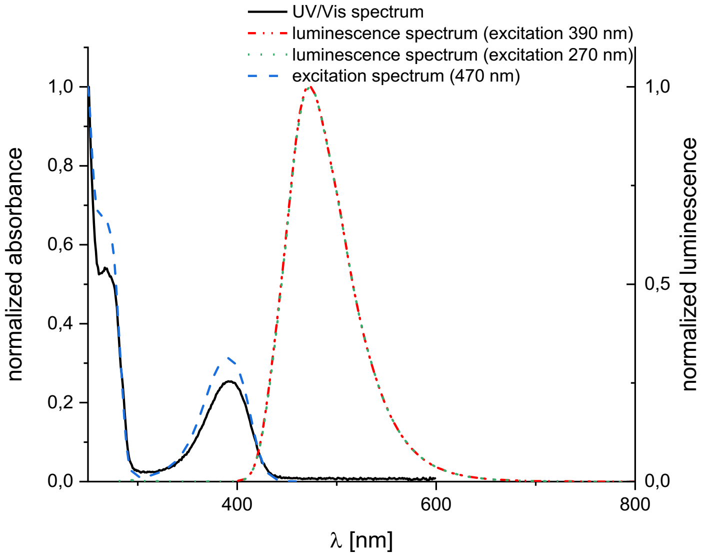

rac-3-(4-methoxybenzyl)-1,2,2-trimethyl-2,3-dihydroquinolin-4(1H)-one (**2f**)

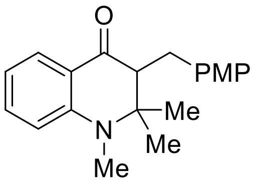

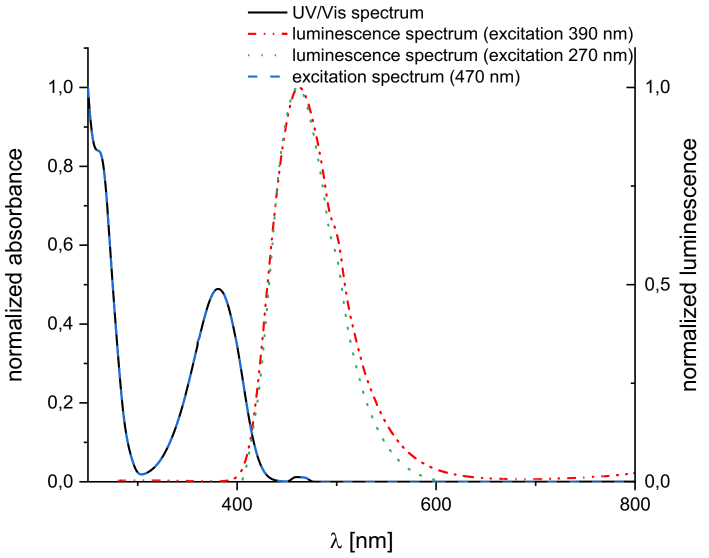

rac-trans-2,3-2-(tert-butyl)-3-(4-methoxybenzyl)-1-methyl-2,3-dihydroquinolin-4(1H)-one (**2e**)

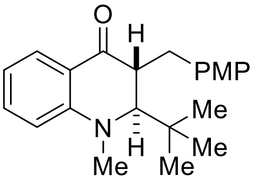

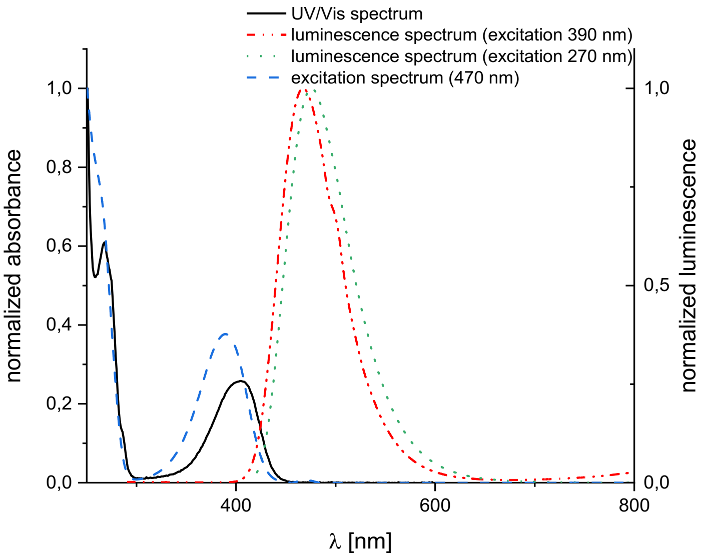

rac-trans-2,3-2-(furan-2-yl)-3-(4-methoxybenzyl)-1-methyl-2,3-dihydroquinolin-4(1H)-one (**2h**)

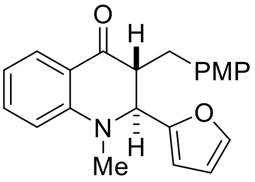

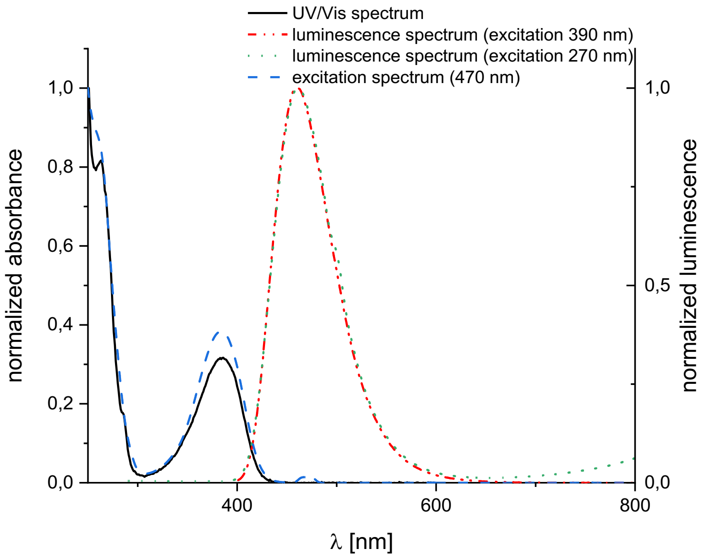

rac-trans-2,3-3-(4-methoxybenzyl)-1-methyl-2-(thiophen-2-yl)-2,3-dihydroquinolin-4(1H)-one (**2i**)

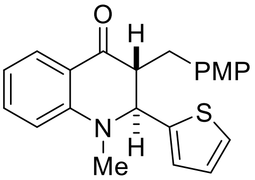

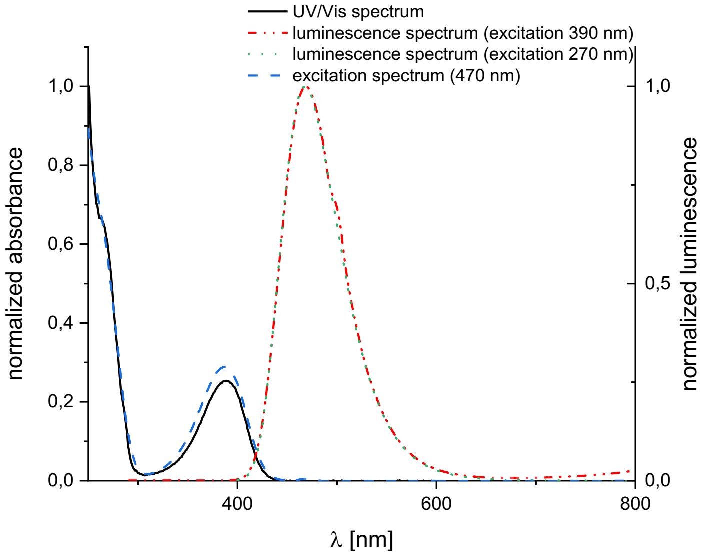

rac-trans-2,3-3-(4-methoxybenzyl)-1-methyl-2-(pyridin-2-yl)-2,3-dihydroquinolin-4(1H)-one (**2j**)

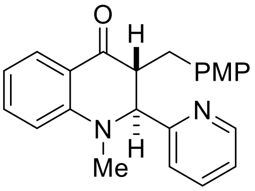

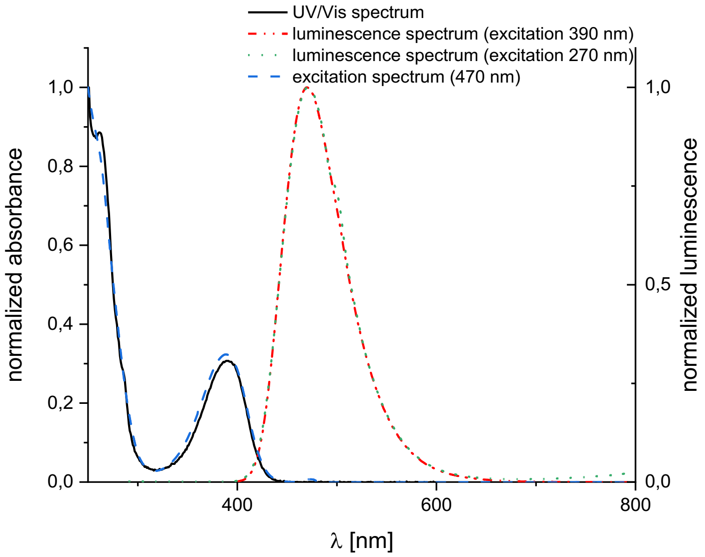

rac-trans-2,3-1-ethyl-3-(4-methoxybenzyl)-2-phenyl-2,3-dihydroquinolin-4(1H)-one (**2k**)

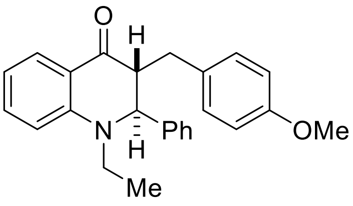

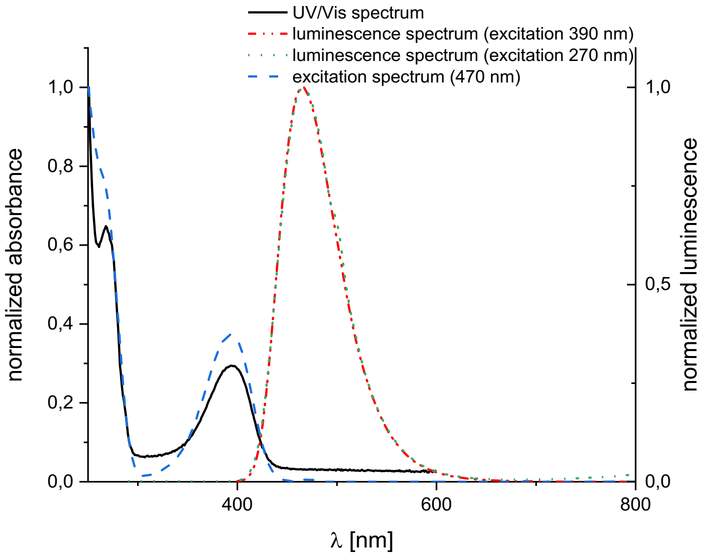

rac-trans-2,3-1-isobutyl-3-(4-methoxybenzyl)-2-phenyl-2,3-dihydroquinolin-4(1H)-one (**2l**)

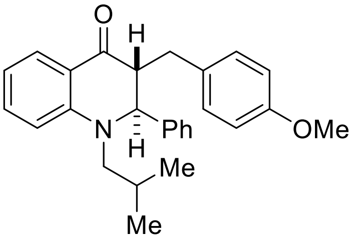

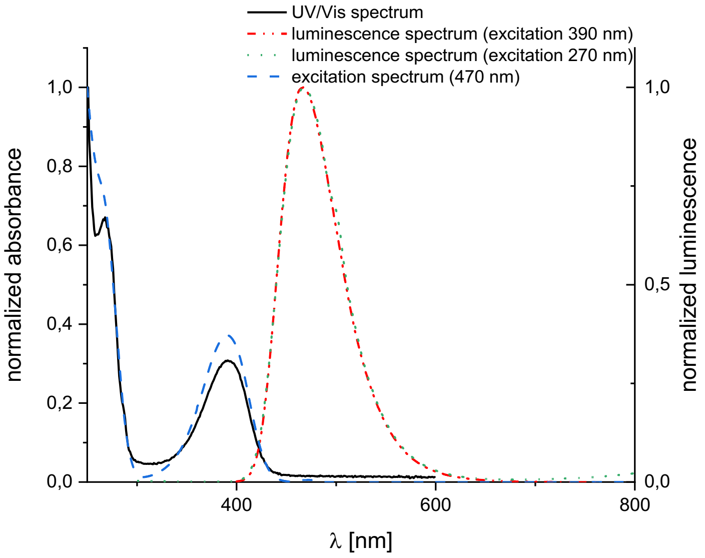

rac-trans-2,3-3-(*cis*-1-(4-chlorophenyl)ethyl)-1-methyl-2-phenyl-2,3-dihydroquinolin-4(1H)-one (*rac*-**2m**)

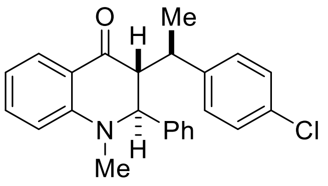

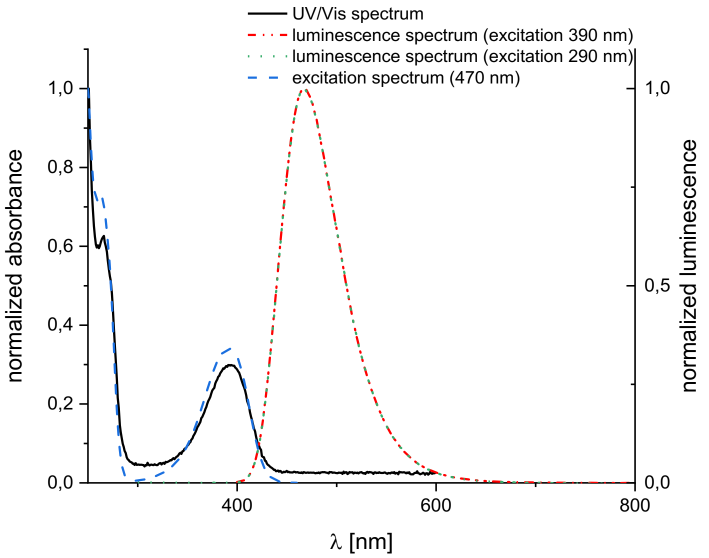

rac-trans-2,3-3-(cis-1-(4-bromophenyl)ethyl)-1-methyl-2-phenyl-2,3-dihydroquinolin-4(1H)-one (*rac*-**2n**)

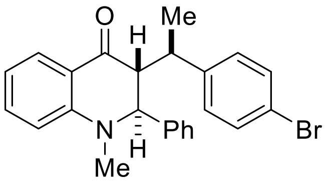

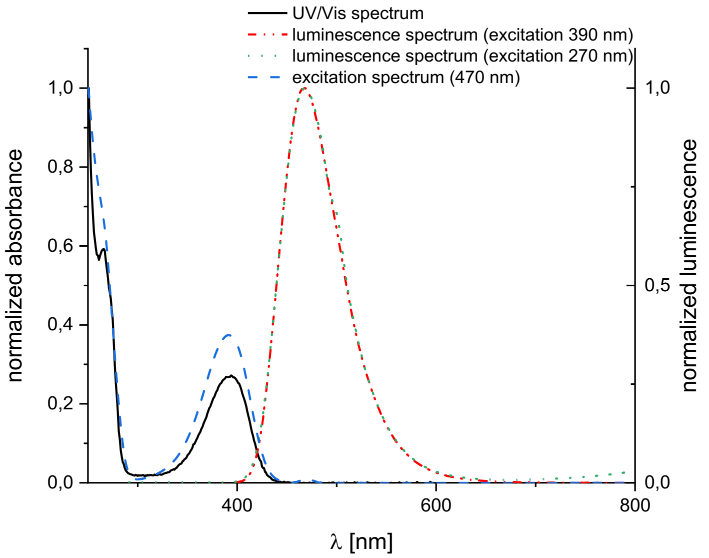

(*2S,3R*)-3-((*R*)-1-(4-bromophenyl)ethyl)-1-methyl-2-phenyl-2,3-dihydroquinolin-4(1H)-one ((*2S,3R*)-**2n**)

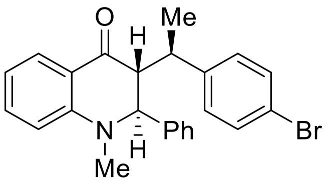

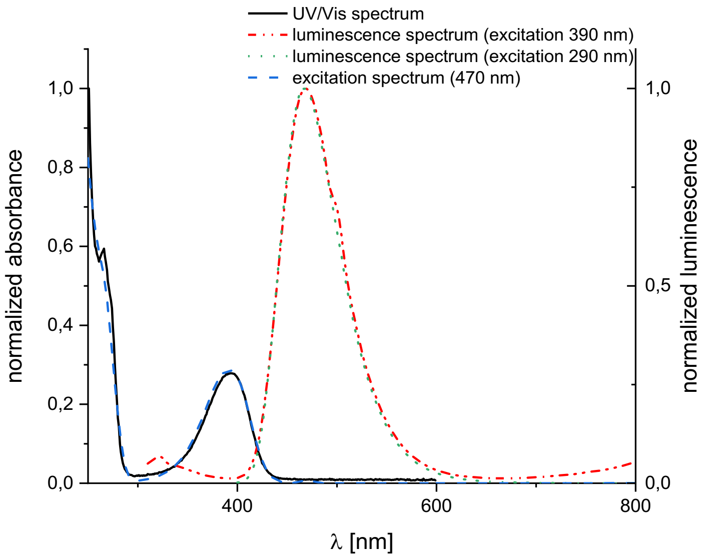

rac-*trans*-2,3-1-methyl-2-phenyl-3-(*cis*-1-(4-(trifluoromethyl)phenyl)ethyl)-2,3-dihydroquinolin-4(1H)-one (*rac*-**2o**)

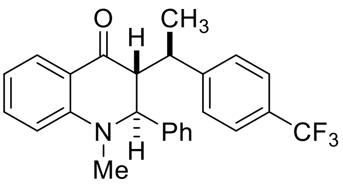

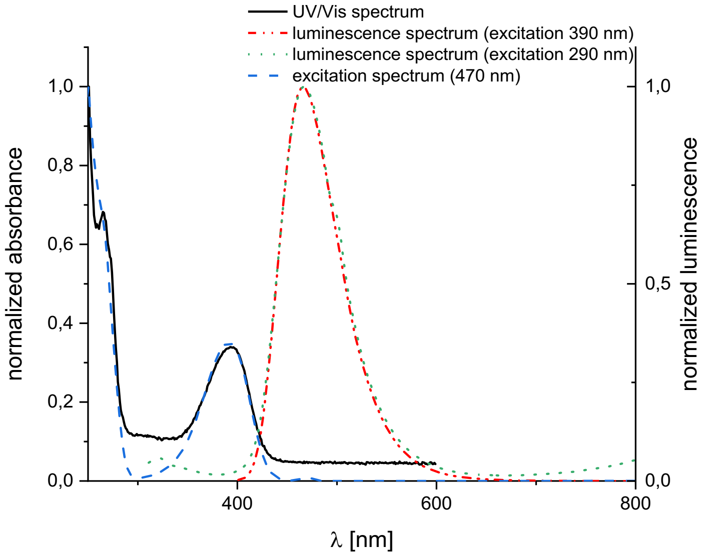

rac-trans-2,3-3-(4-fluorobenzyl)-1-methyl-2-phenyl-2,3-dihydroquinolin-4(1H)-one (**2p**)

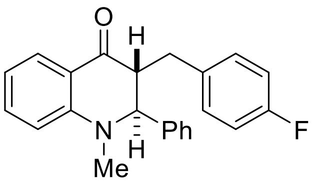

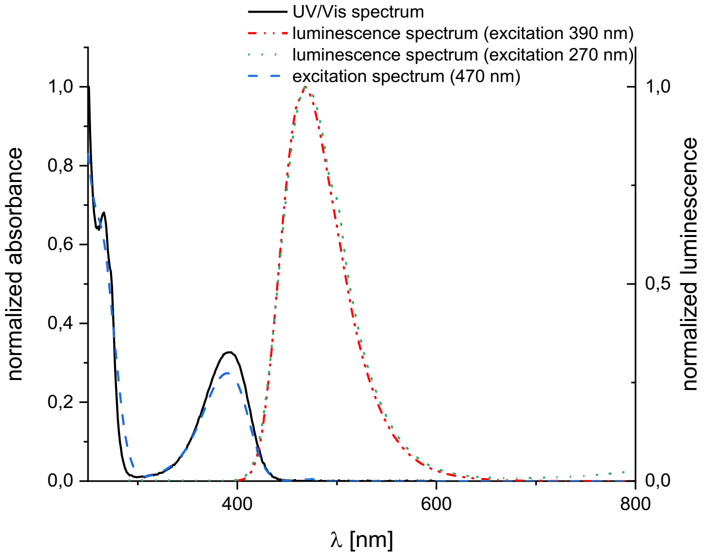

rac-*trans*-2,3-3-(4-fluorobenzyl)-2-(4-fluorophenyl)-1-methyl-2,3-dihydroquinolin-4(1H)-one (**2q**)

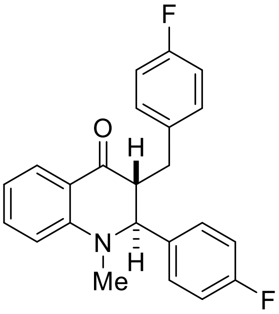

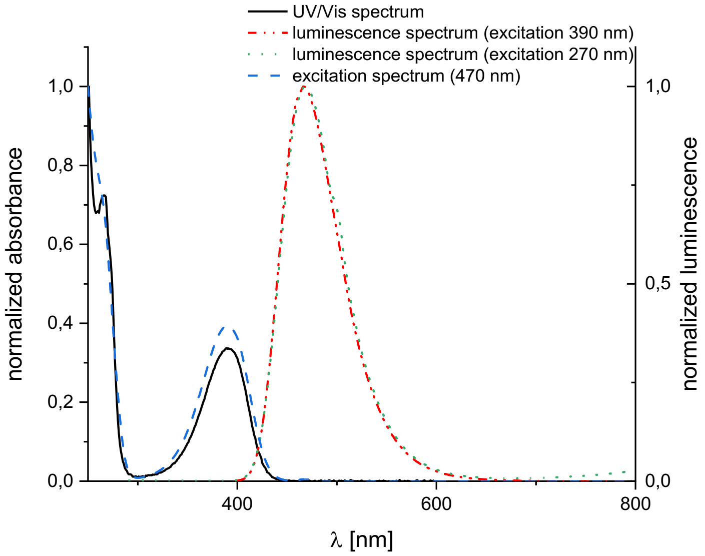

rac-trans-2,3-3-cinnamyl-1-methyl-2-phenyl-2,3-dihydroquinolin-4(1H)-one (**2r**)

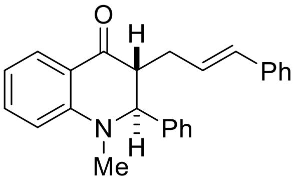

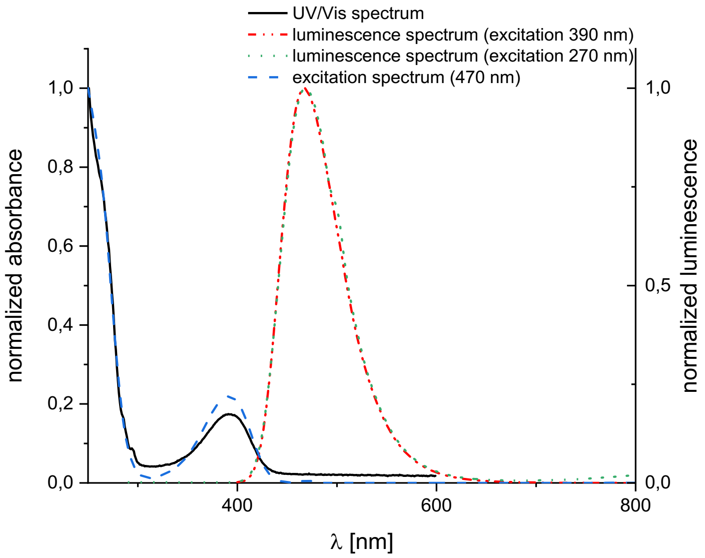

rac-trans-2,3-1-methyl-2-phenyl-3-(3-phenylprop-2-yn-1-yl)-2,3-dihydroquinolin-4(1H)-one (**2s**)

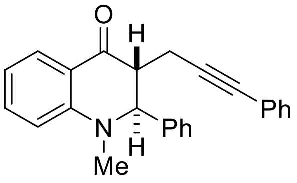

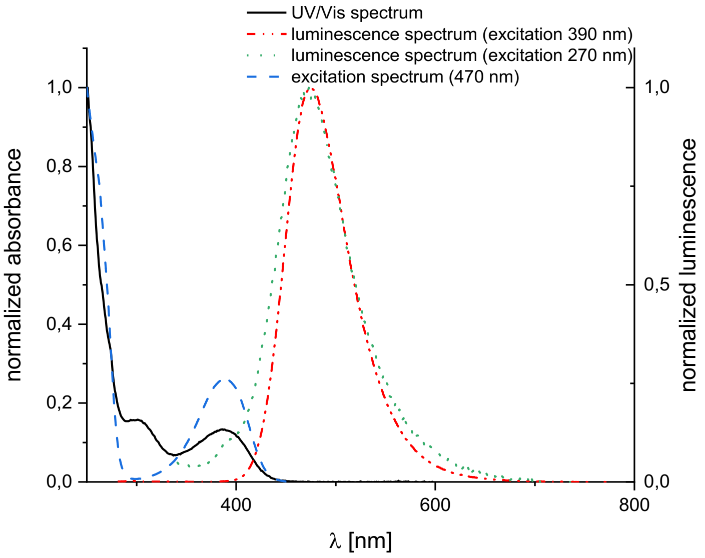

^{1}H-NMR (500 MHz, 303 K, $CDCl_3$) (**2t**)

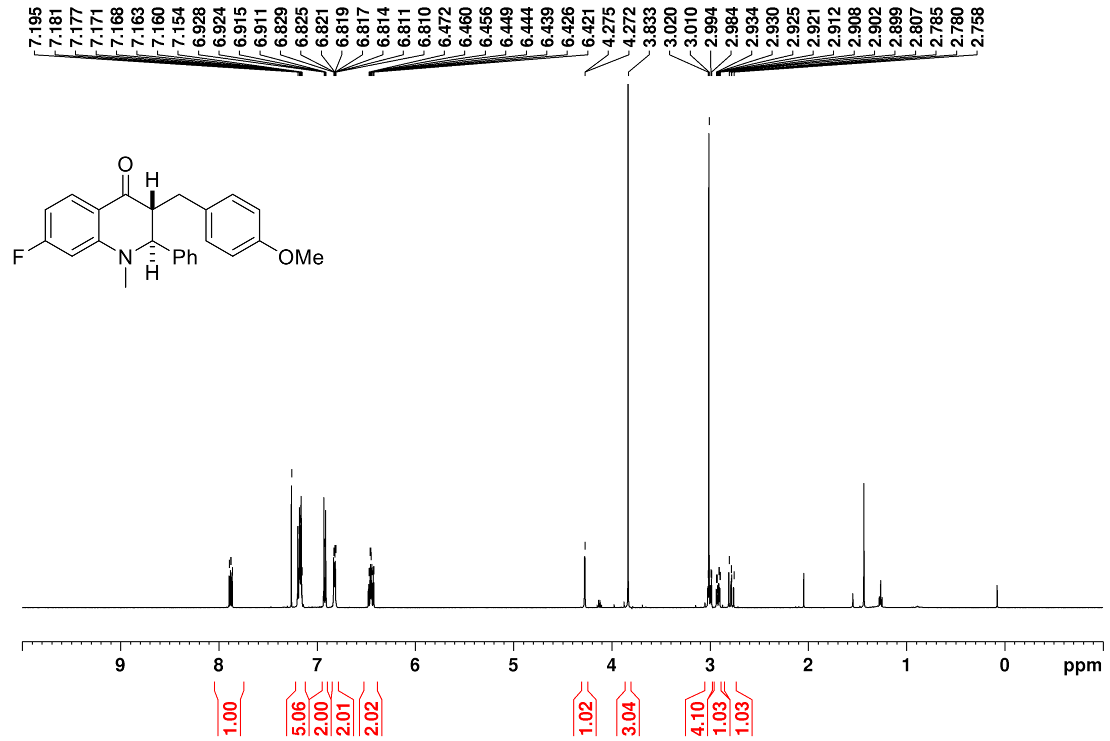

^{13}C-NMR (176 MHz, 298 K, $CDCl_3$) (**2t**)

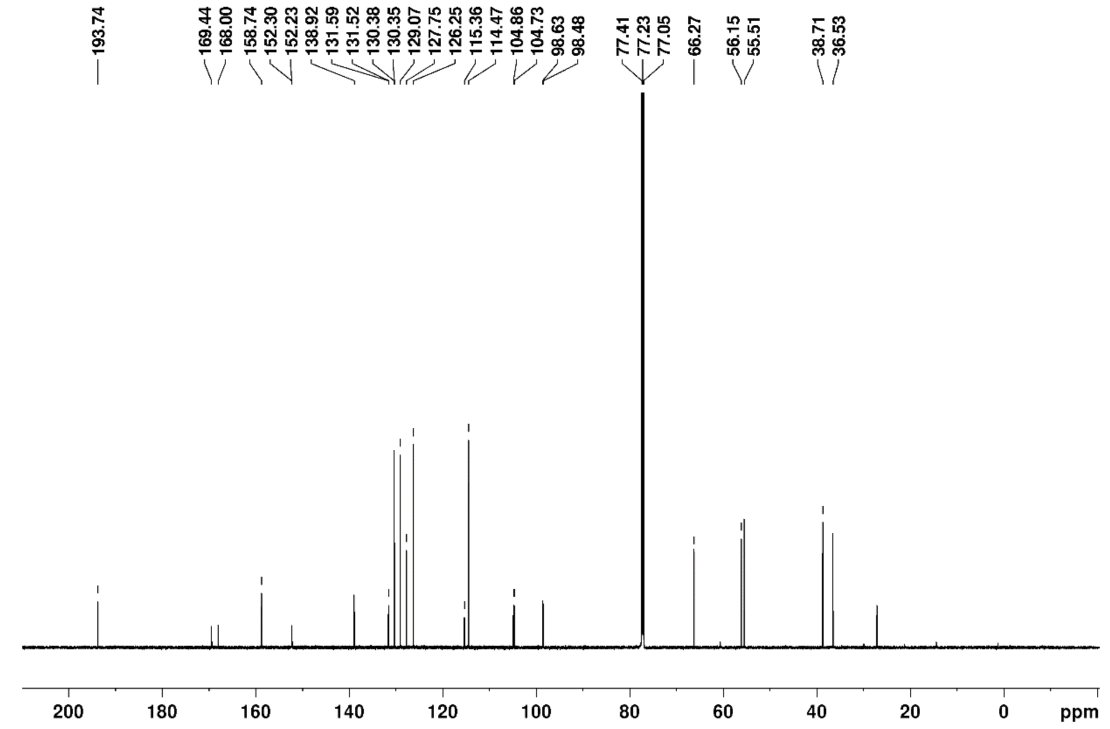

^{1}**H-NMR** (500 MHz, 303 K, $CDCl_3$) (**2u**)

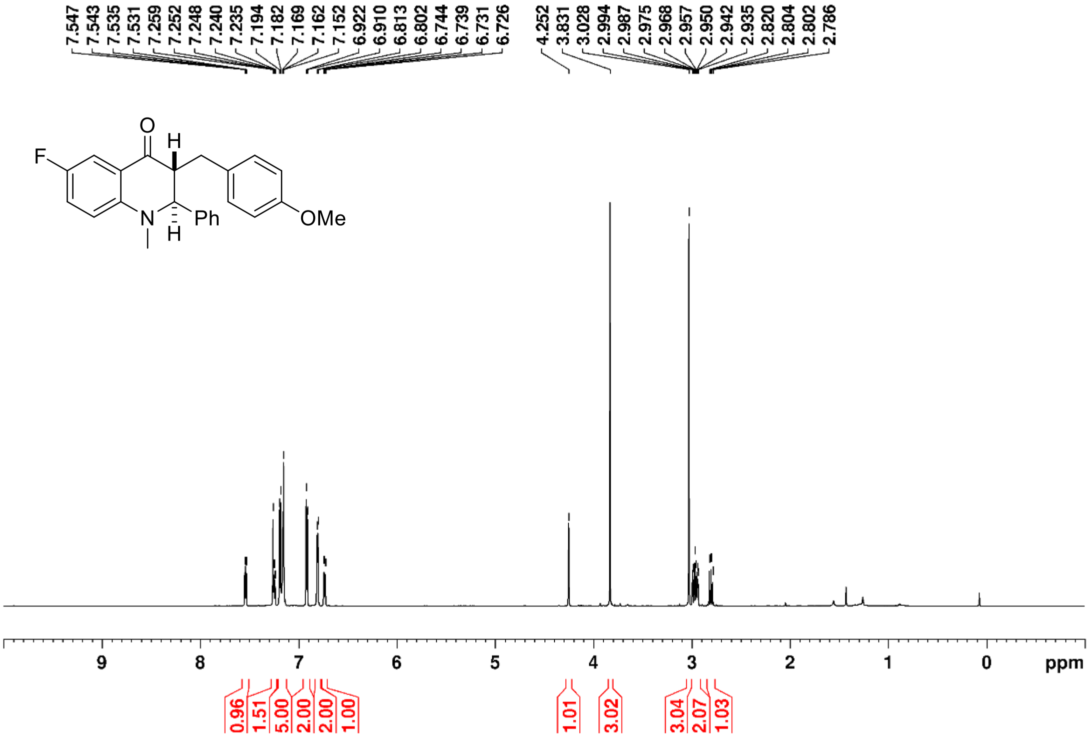

^{13}**C-NMR** (176 MHz, 298 K, $CDCl_3$) (**2u**)

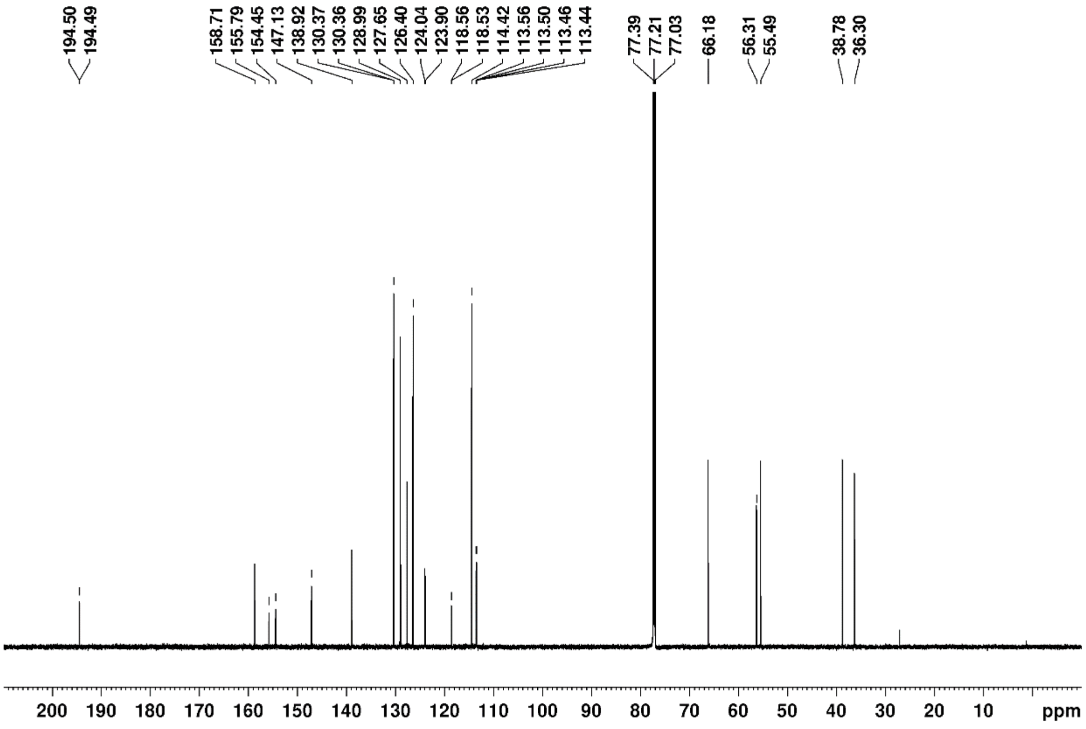

¹H-NMR (500 MHz, 303 K, $CDCl_3$) (**2v**)

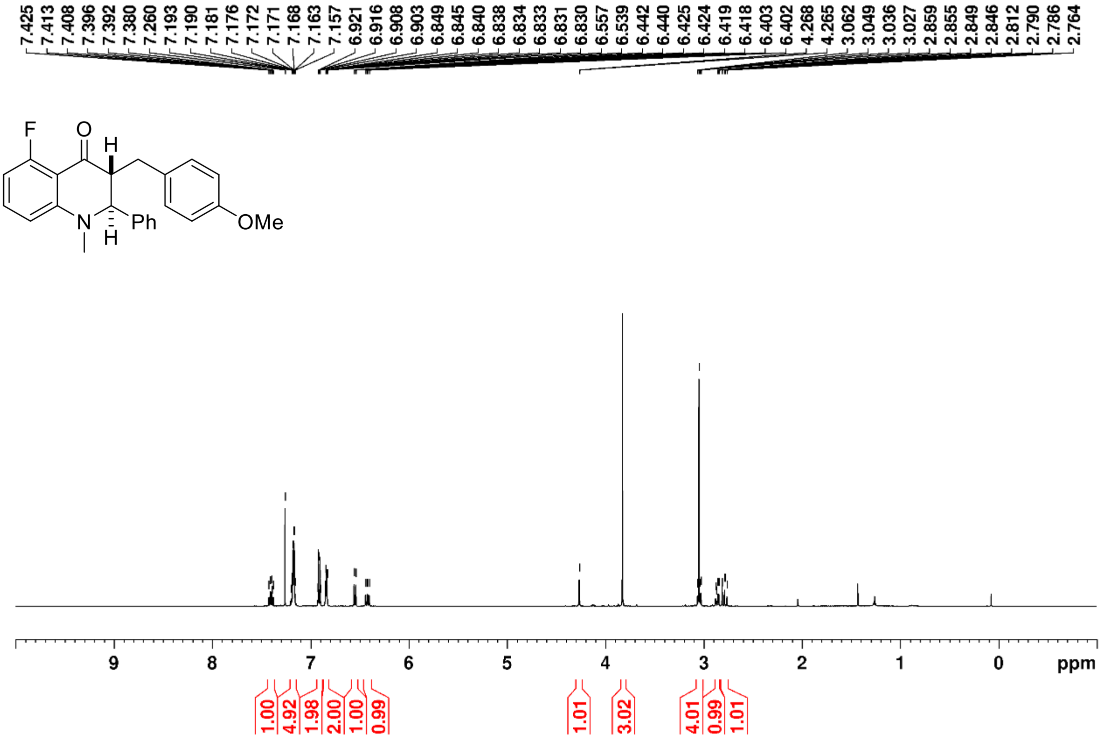

¹³C-NMR (176 MHz, 298 K, $CDCl_3$) (**2v**)

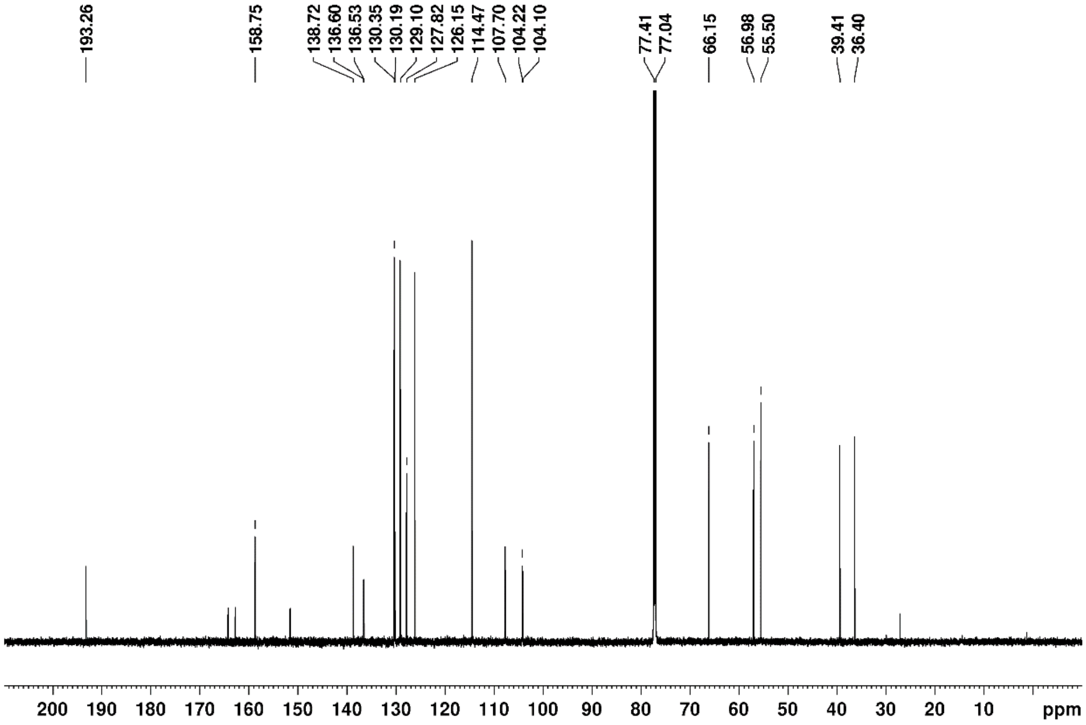

^{1}H-NMR (700 MHz, 298 K, $CDCl_3$) (**2w**)

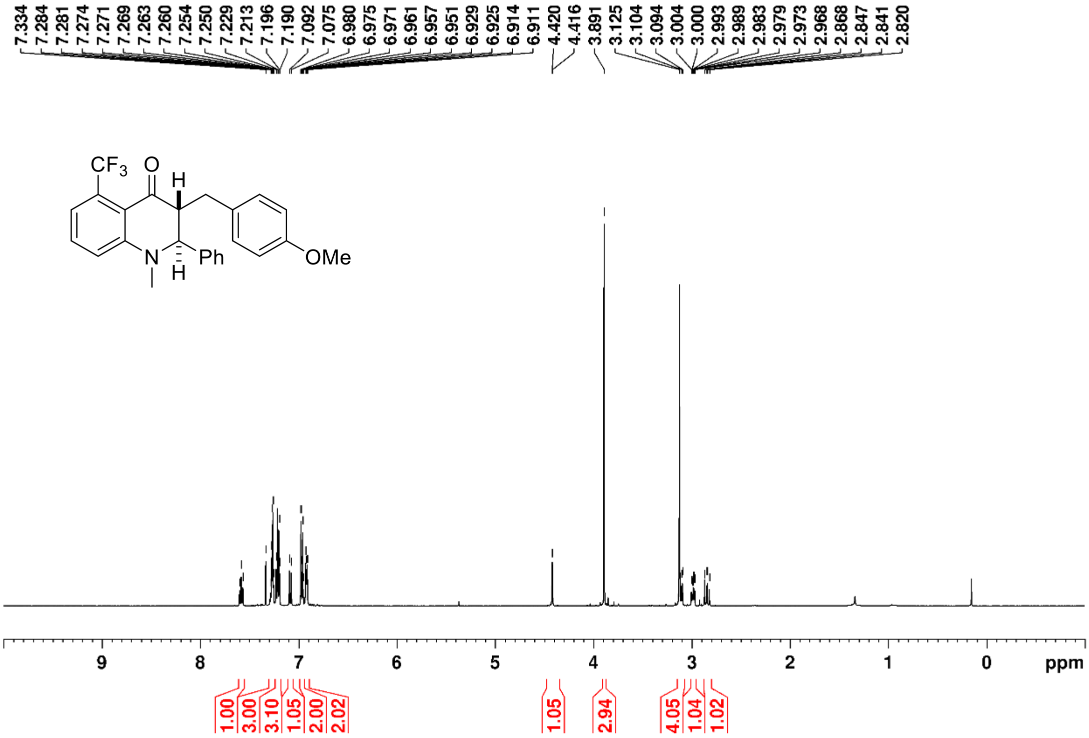

^{13}C-NMR (176 MHz, 298 K, $CDCl_3$) (**2w**)

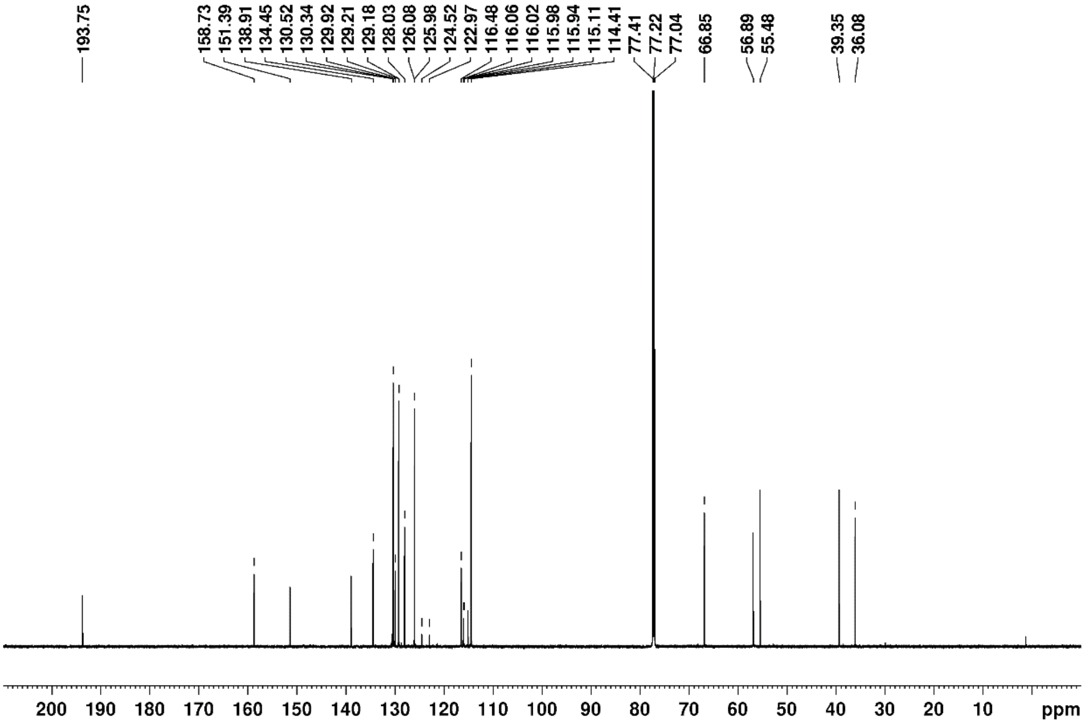

^{1}H-NMR (500 MHz, 303 K, $CDCl_3$) (**2x**)

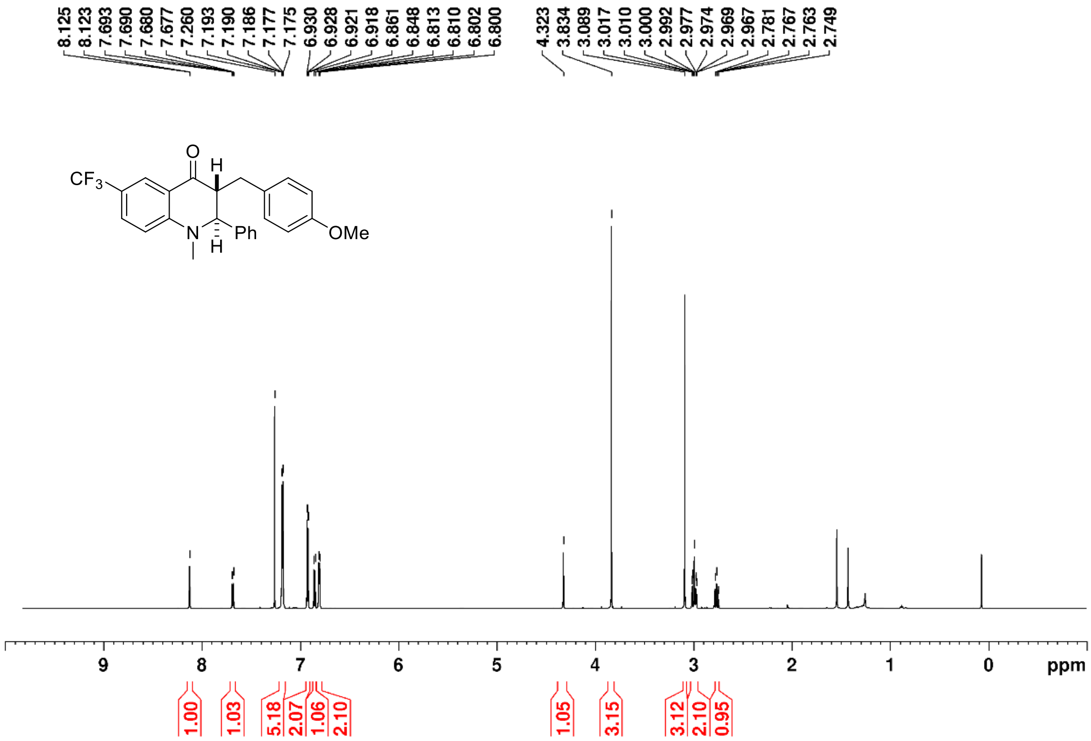

^{13}C-NMR (176 MHz, 298 K, $CDCl_3$) (**2x**)

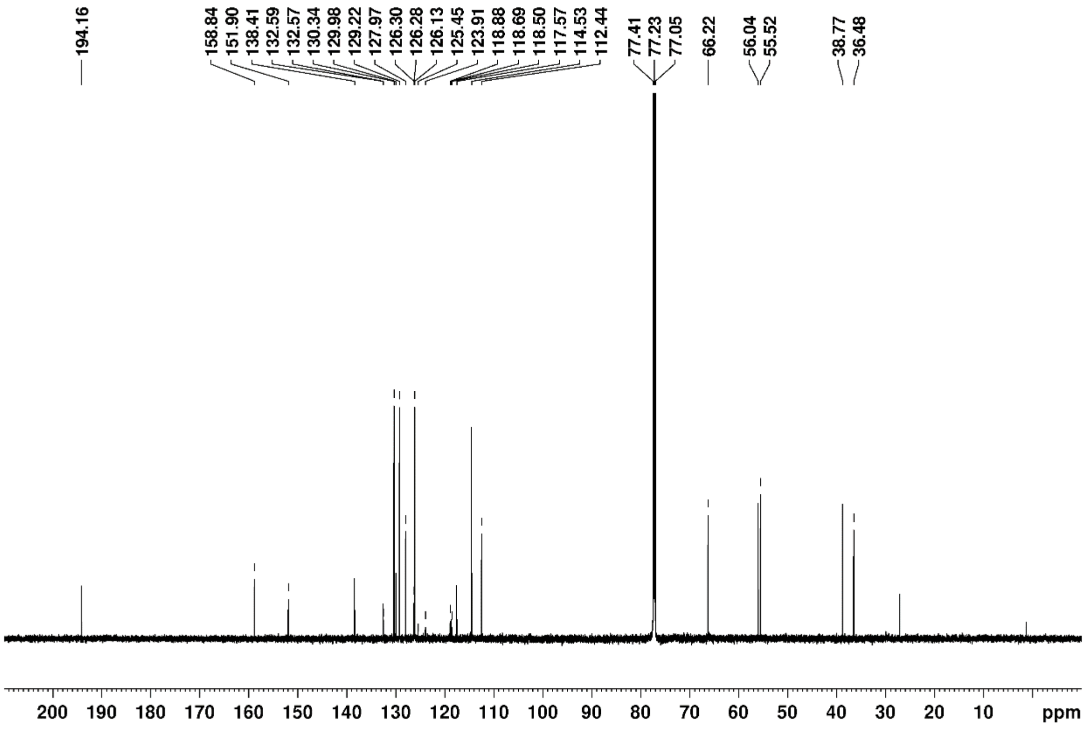

^{1}H-NMR (700 MHz, 298 K, $CDCl_3$) (2y)

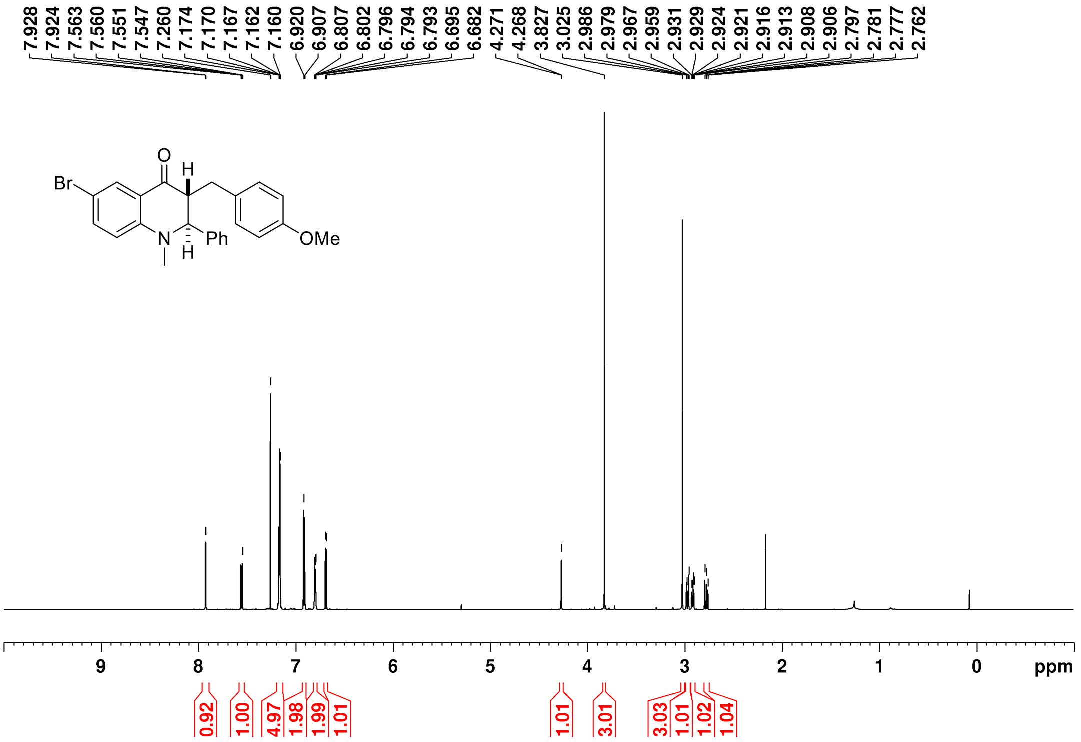

^{13}C-NMR (176 MHz, 298 K, $CDCl_3$) (2y)

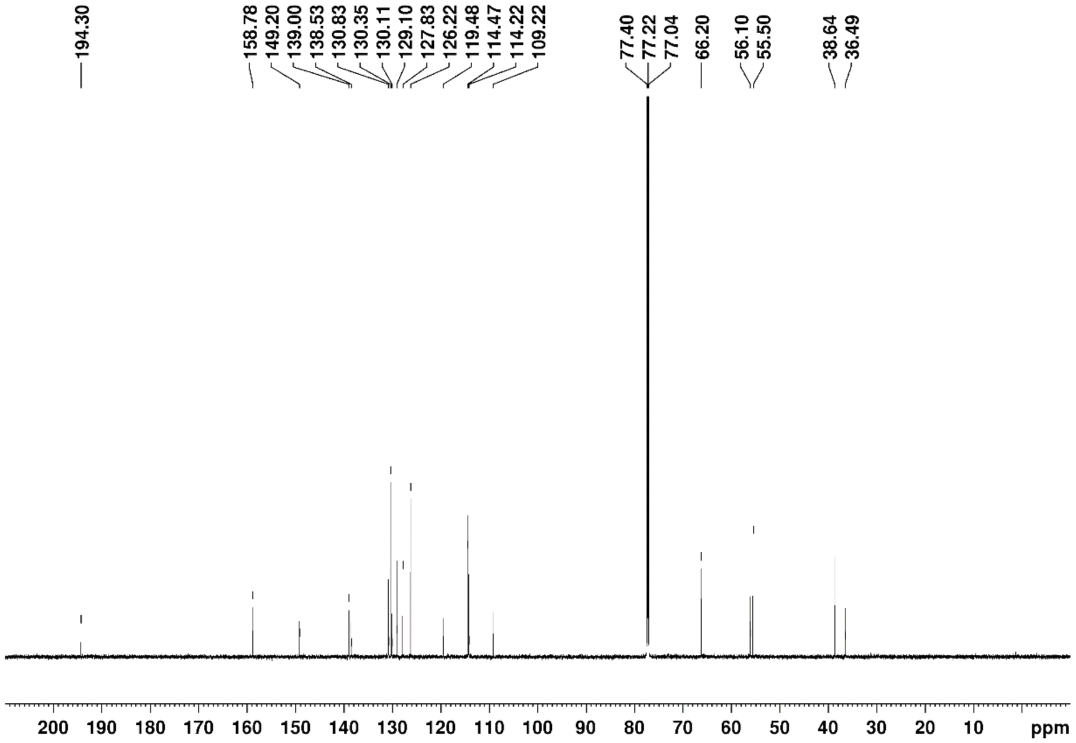

^{1}H-NMR (700 MHz, 298 K, $CDCl_3$) (**2z**)

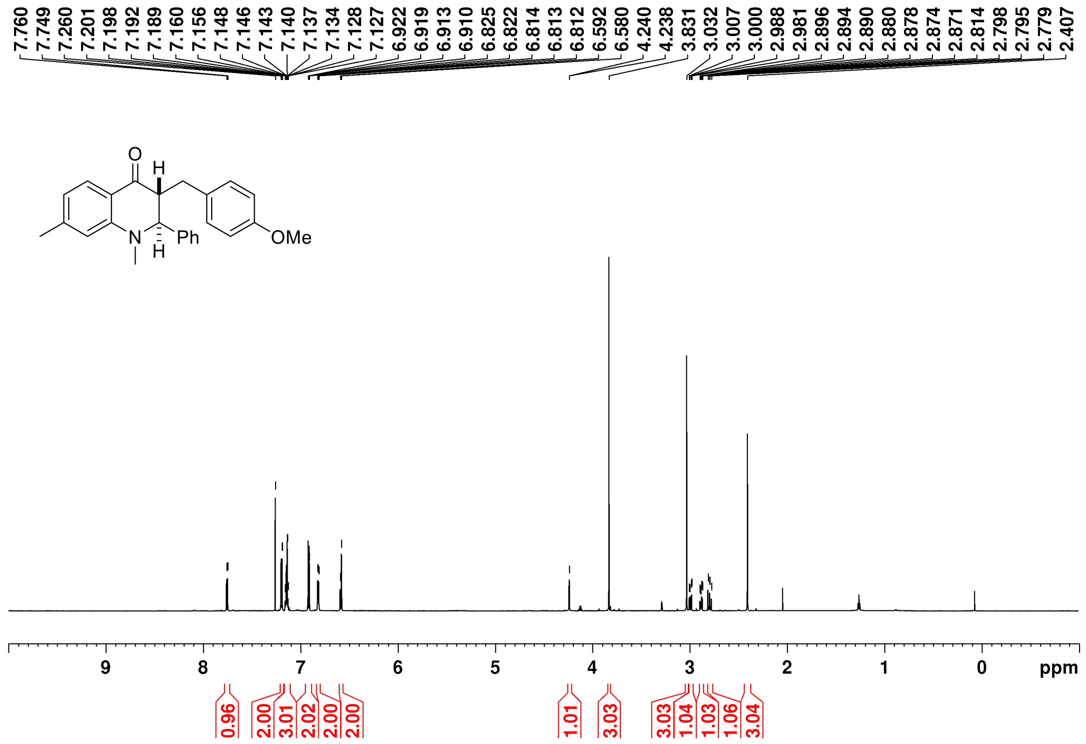

^{13}C-NMR (176 MHz, 298 K, $CDCl_3$) (**2z**)

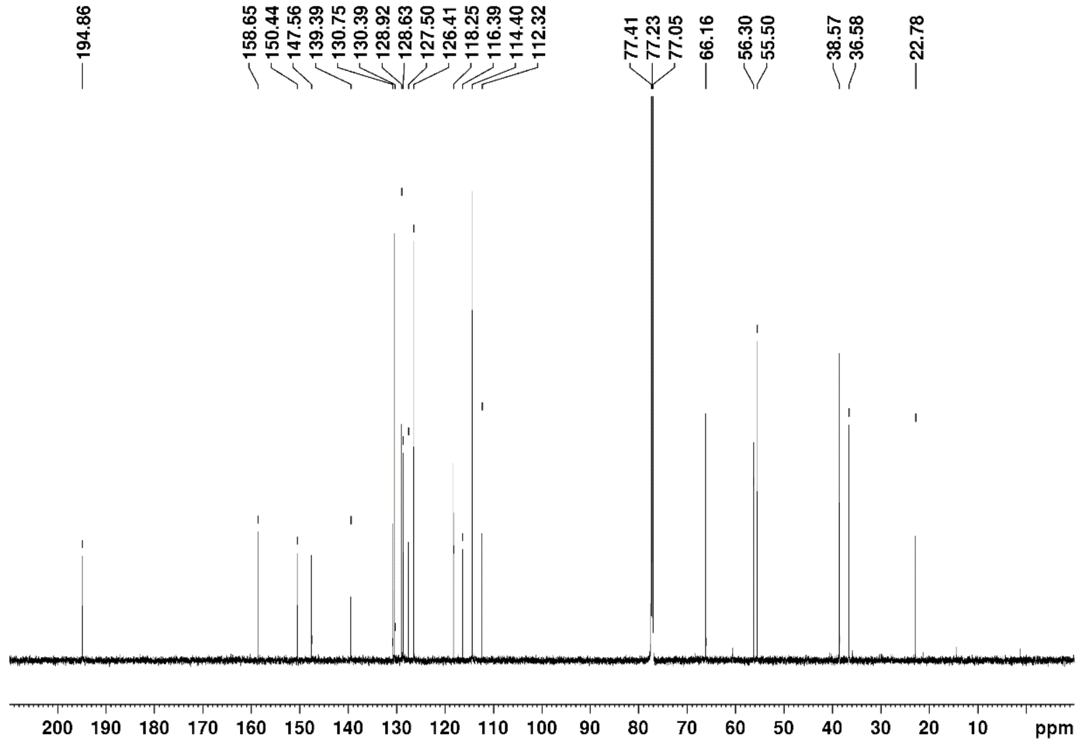

^{1}H-NMR (700 MHz, 298 K, $CDCl_3$) (2aa)

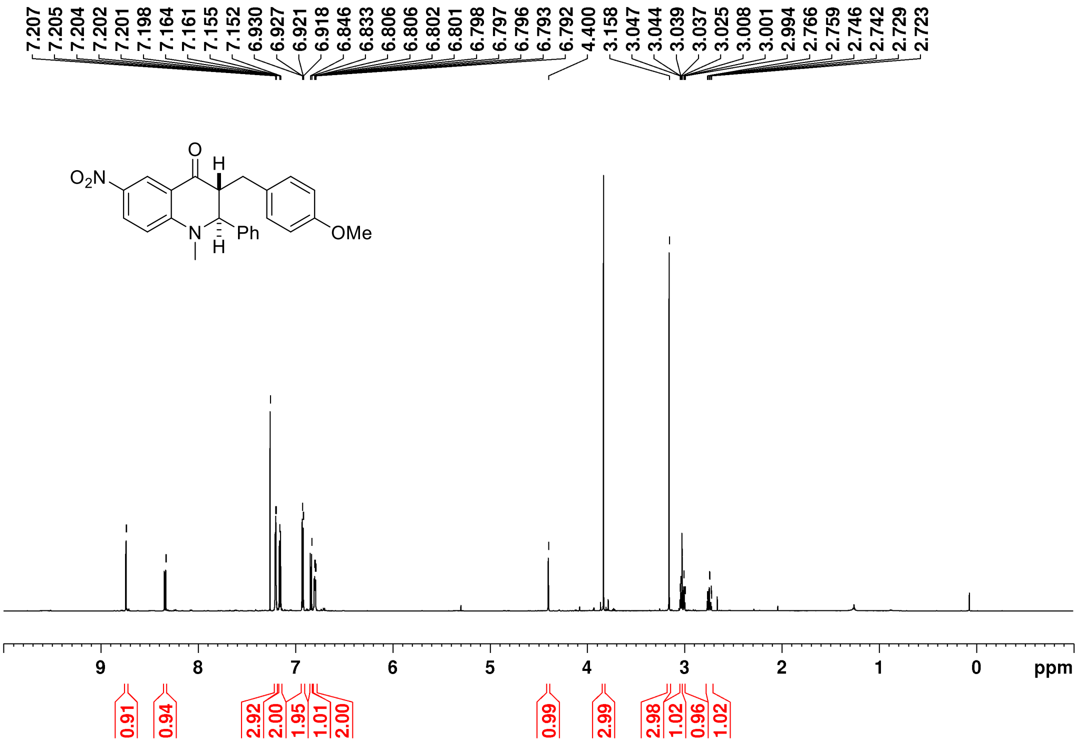

^{13}C-NMR (176 MHz, 298 K, $CDCl_3$) (2aa)

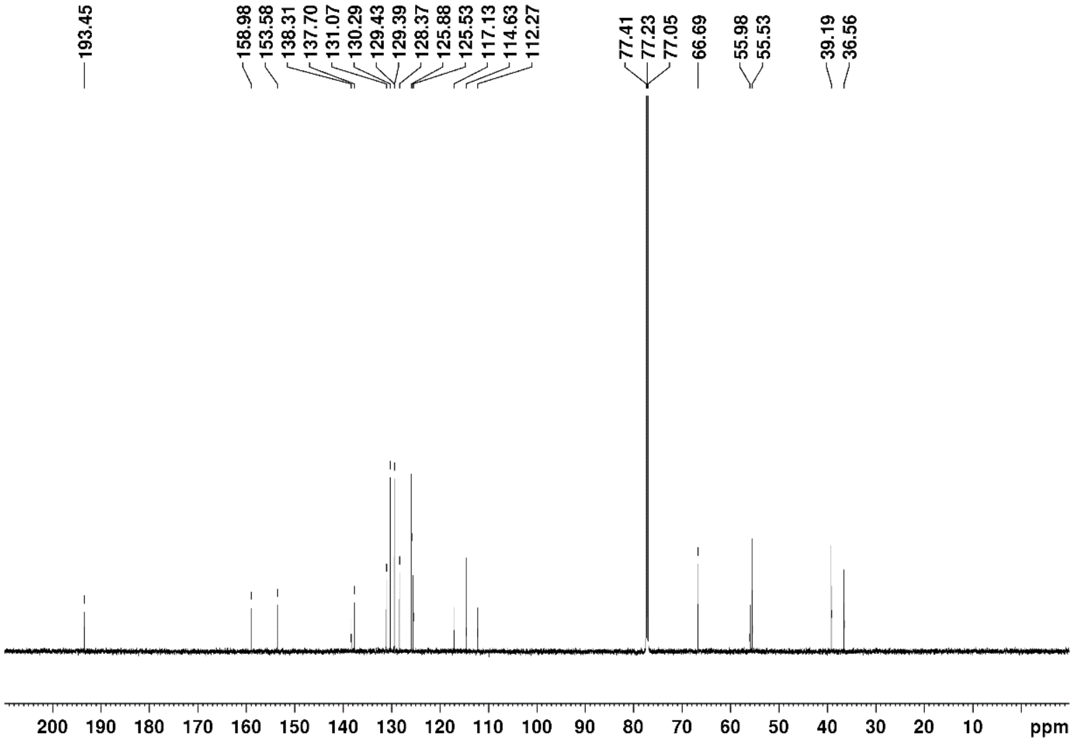

^{1}**H-NMR** (700 MHz, 298 K, $CDCl_3$) (**2ab**)

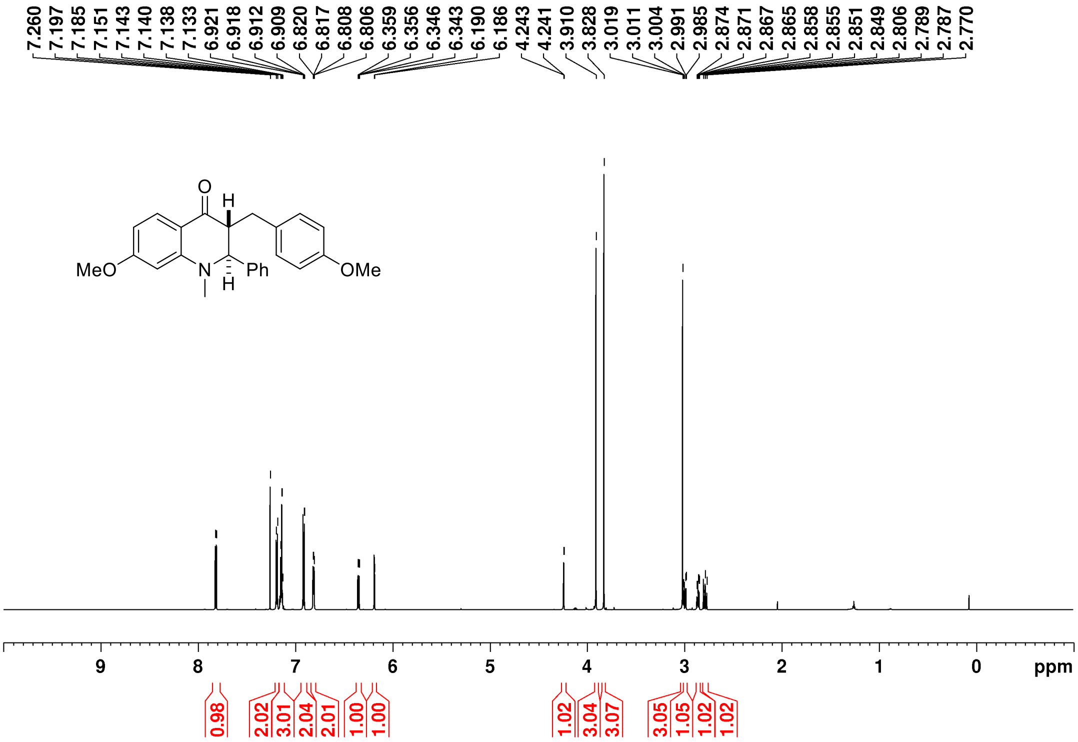

^{13}**C-NMR** (176 MHz, 298 K, $CDCl_3$) (**2ab**)

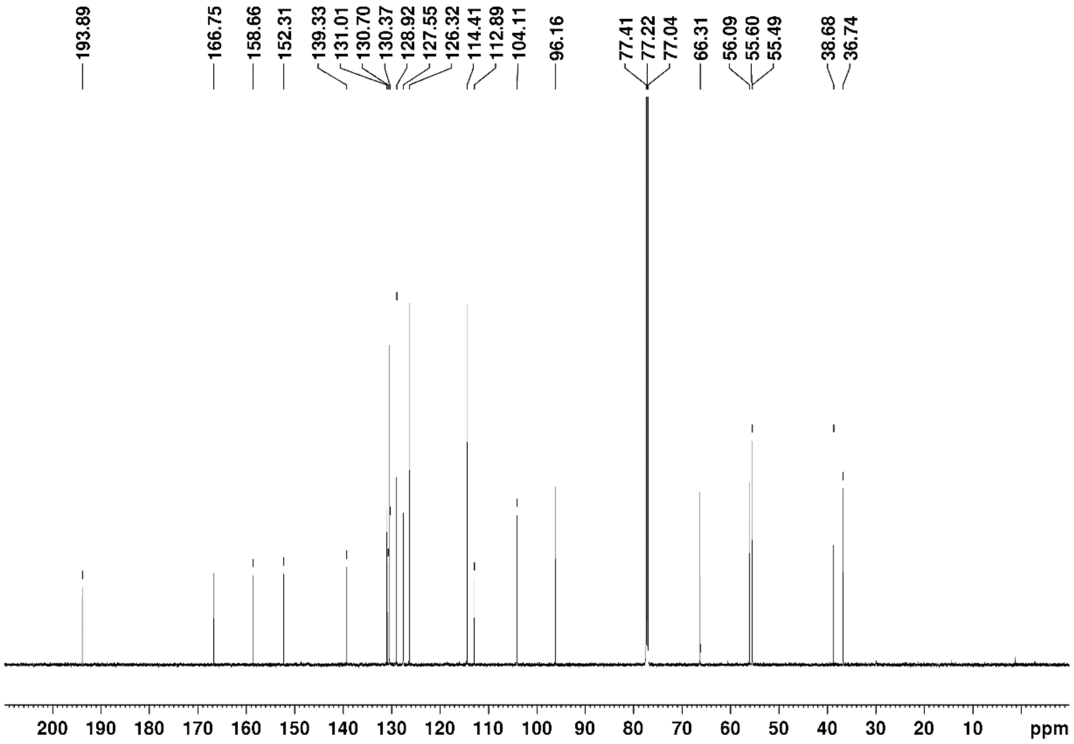

5 Mechanistic studies and kinetic analysis

5.1.1 Isolation of ((1,1-dimethyl-2-phenyl-1,2,3,4-tetrahydroquinolin-1-ium-4-yl)oxy)tris(perfluorophenyl)borate (3)

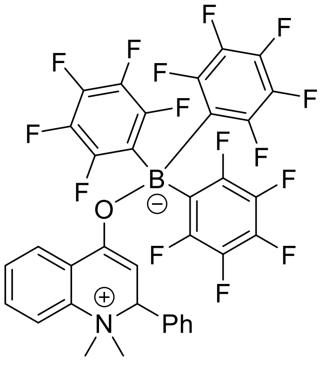

4 (25.1 mg, 0.100 mmol, 1.00 equiv.) and $B(C_6F_5)_3$ (51.2 mg, 0.100 mmol, 1.00 equiv.) were dissolved in $CHCl_3$ and stirred at room temperature overnight leading to the precipitation of a colorless solid. The solvent was removed under reduced pressure and the solid was analyzed by NMR spectroscopy. Suitable crystals for X-ray single crystal structure analysis were grown by condensation of *n*-pentane into the chloroform solution.

^{1}H-NMR (700 MHz, 298 K, CD_3CN): δ = 8.02 – 7.99 (m, 1H, H_{Ar}), 7.63 – 7.59 (m, 2H, H_{Ar}), 7.50 – 7.46 (m, 1H, H_{Ar}), 7.41 – 7.39 (m,1H, H_{Ar}), 7.37 – 7.34 (m, 1H, H_{Ar}), 7.23 – 7.17 (m, 2H, H_{Ar}), 6.87 (bs, 2H, H_{Ar}), 5.13 (d, $^3J_{HH}$ = 5.7 Hz, 1H, NC*H*), 4.68 (d, $^3J_{HH}$ = 6.7 Hz, 1H, COC*H*), 3.54 (s, 3H, CH_3^A), 3.11 (s, 3H, CH_3^B); **^{13}C-NMR** (176 MHz, 298 K, $CDCl_3$): δ =149.2 (C_q), 148.9 (dm, $^1J_{CF}$ = 239.5 Hz, CF), 141.0 (C_q), 139.7 (dm, $^1J_{CF}$ = 245.7 Hz, CF), 137.6 (dm, $^1J_{CF}$ = 245.5 Hz, CF), 132.8 (C_q), 131.8 (CH), 131.4 (CH), 131.2 (CH), 130.8 (C_q, C-O), 130.0 (CH), 129.6 (CH), 123.4 (bs, C_q, C-B), 120.1 (CH), 96.8 (CH, CO*C*H), 77.5 (CH, N*C*H), 56.7 (CH_3, CH_3^A), 50.1 (CH_3, CH_3^B); **^{11}B-NMR** (225 MHz, 298 K, CD_3CN): δ = −3.6 (s); **^{1}H/^{15}N HMBC** (700/71 MHz, 298 K, CD_3CN): δ = 4.68/65.1, 7.41/65.1; **^{19}F-NMR** (659 MHz, 298 K, CD_2Cl_2): δ = −135.9 – −136.2 (m, 2F, F_{ortho}), −160.2 – −160.4 (m, 1H, H_{para}), −165.5 – −165.8 (m, 2F, F_{meta}).

5.1.2 General procedure for kinetic measurements

In a glove box the reaction substrate **1b**, **1c** or **1d** (50.0 µmol, 1.00 equiv.) and B(2,3,6-F_3-C_6H_2)$_3$ (2.0 mg, 5.00 µmol, 0.100 equiv.) were dissolved in $CDCl_3$ (0.50 ml, 0.100 M). The solution was directly transferred into a sealable NMR tube equipped with a J. YOUNG Teflon tap. The sample was subjected to ^{1}H-NMR spectroscopy at 30 °C representing the t_0 measurement. The sample was kept in the spectrometer to maintain a constant temperature and measurements were performed each 15 (**1b and 1d**) or 30 minutes (**1c**). For integration suitable aromatic ppm regions for starting material and product were used, referenced to $CDCl_3$ (**1b** and **1d**) or the signal of residual silicon

grease (0.07 ppm, **1c**) (as the solvent signal overlaps with the starting material and the product). The conversion of the reaction was determined by signal integration using the DCM impurity signal as an internal standard as the signals of customary internal standards would overlap with either product or starting material signals. All calculations and data analysis were performed with MICROSOFT EXCEL, linear regression with ORIGINLAB OriginPro 2018.

Table S2: Mean rate of reaction and calculated reaction constant.

X	k (mol/l·h) Value	Error	σ	$\log(k_{rel}/k_0)$	Error
F	0,02576333	0,00066308	0,06	0,06818474	0,00358988
H	0,02202	0,00048769	0	0	0
OMe	0,00635667	7,5942E-05	-0,27	-0,53958788	-0,01020063

	Value	Error
ρ	1,88967	0,13047

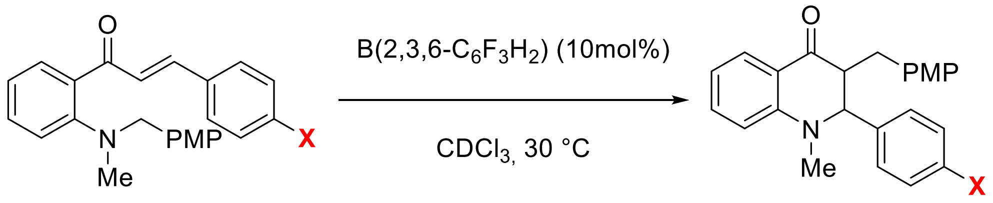

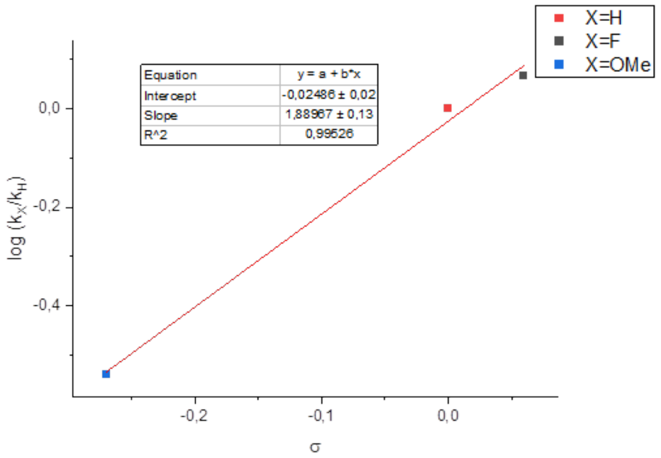

Complete kinetic data for the conversion of 1b

GWI-636a, BCF3, GWI-493, CDCl3, 10 mol%, 30°C

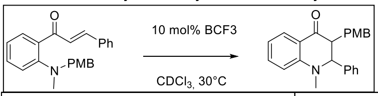

	Value	Error		Value	Error	Regression (Origin)	Value	Error
Time interval [h]	0,25	0,0042	n [mmol]	0,05	0,00012391	(intercept)	2,4700E-03	1,4107E-04
NMR-error	0,02		Vprobe (ml)	0,5	0,01	slope	3,9050E-02	9,3818E-04
						k (mol/l*h)	3,9050E-02	9,3818E-04

Exp. Nr.	Time h	dt [h]	DCM	SM (1H)	dSM	Prod(1H)	dP	conv. [%]	c SM [mol/l]	dc SM	c P [mol/l]	dc P
1	0	0	1	31,2231	0,62446134	0,696906	0,013938111	2,18328994	0,09781671	0,00231541	0,00218329	0,000135199
2	0,25	0,00105	1	29,9907	0,59981339	2,568333	0,051366668	7,888243315	0,09211176	0,00239057	0,00788824	0,000470473
3	0,5	0,0021	1	28,2802	0,56560335	4,334970	0,08669941	13,29128366	0,08670872	0,00243774	0,01329128	0,000763997
4	0,75	0,00315	1	26,5003	0,53000647	5,995878	0,119917562	18,45101202	0,08154899	0,00246099	0,01845101	0,001022503
5	1	0,0042	1	24,7856	0,49571117	7,582005	0,151640104	23,42470155	0,0765753	0,00246323	0,0234247	0,001251528
6	1,25	0,00525	1	22,9982	0,45996323	9,018104	0,180362073	28,16725677	0,07183274	0,00244695	0,02816726	0,001451478
7	1,5	0,0063	1	21,7598	0,43519608	10,549810	0,210996194	32,65223078	0,06734777	0,00241499	0,03265223	0,001624014
8	1,75	0,00735	1	20,3402	0,40680421	11,930844	0,238616873	36,97072795	0,06302927	0,00236901	0,03697073	0,001774939
9	2	0,0084	1	19,0084	0,38016753	13,227479	0,264549581	41,03343583	0,05896656	0,00231214	0,04103344	0,001903304
10	2,25	0,00945	1	17,6174	0,35234732	14,348456	0,286969117	44,88686639	0,05511313	0,00224599	0,04488687	0,002012855
11	2,5	0,0105	1	16,3904	0,32780764	15,493864	0,309877287	48,59410594	0,05140589	0,00217114	0,04859411	0,002107038
12	2,75	0,01155	1	15,1312	0,30262439	16,462760	0,329255207	52,10726995	0,04789273	0,00209006	0,05210727	0,002186144
13	3	0,0126	1	14,0960	0,28191919	17,446034	0,348920673	55,3104987	0,0446895	0,00200753	0,0553105	0,002249666
14	3,25	0,01365	1	13,1257	0,26251316	18,384514	0,367690272	58,34469556	0,0416553	0,00192179	0,0583447	0,002302265
15	3,5	0,0147	1	12,2146	0,24429114	19,298633	0,385972664	61,23985925	0,03876014	0,0018331	0,06123986	0,002345588
16	3,75	0,01575	1	11,2570	0,22513934	19,991241	0,399824826	63,97563983	0,03602436	0,00174314	0,06397564	0,002380363
17	4	0,0168	1	10,5601	0,21120211	21,009341	0,420186826	66,54960233	0,0334504	0,00165303	0,0665496	0,002407615
18	4,25	0,01785	1	9,8026	0,1960528	21,765842	0,435316834	68,94801536	0,03105198	0,0015643	0,06894802	0,002428238
19	4,5	0,0189	1	9,0749	0,18149724	22,387738	0,447754762	71,15666897	0,02884333	0,00147852	0,07115667	0,002443159
20	4,75	0,01995	1	8,5328	0,17065612	23,312589	0,466251789	73,20552665	0,02679447	0,00139545	0,07320553	0,002453511
21	5	0,021	1	7,7561	0,15512254	23,619407	0,472388133	75,27969683	0,0247203	0,00130794	0,0752797	0,002460571

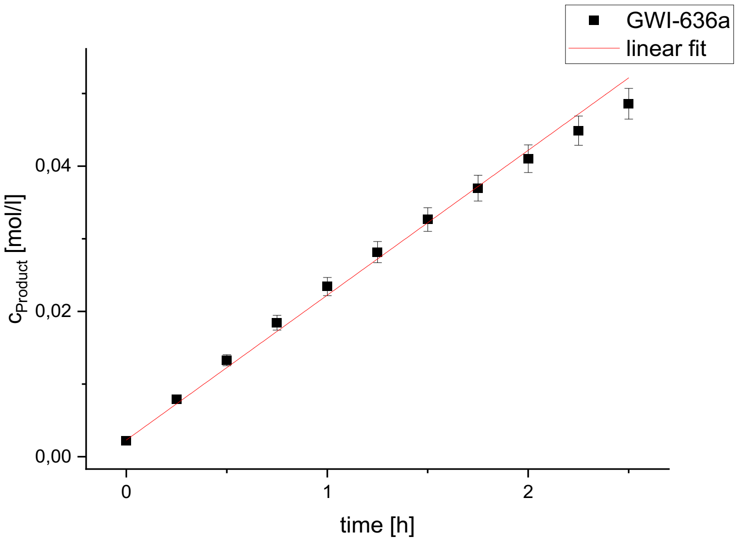

GWI-636b, BCF3, GWI-493, CDCl3, 10 mol%, 30°C

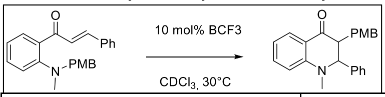

Regression (Origin)

	Value	Error		Value	Error		Value	Error
Time interva	0,25	0,0042	n [mmol]	0,05	0,00012391	(intercept)	1,5558E-04	2,2000E-02
NMR-error	0,02		Vprobe (ml)	0,5	0,01	slope	2,2000E-02	4,9381E-04
						k (mol/l*h)	2,2000E-02	4,9381E-04

Exp. Nr.	Time h	dt [h]	DCM	SM (1H)	dSM	Prod(1H)	dP	conv. [%]	c SM [mol/l]	dc SM	c P [mol/l]	dc P
1	0	0	1	37,6251	0,75250226	0,767415	0,015348296	1,998865056	0,098001135	0,00231255	0,00199887	0,00012393
2	0,25	0,00105	1	35,5160	0,71032001	3,230876	0,064617527	8,338417451	0,091661583	0,00239539	0,00833842	0,00049582
3	0,5	0,0021	1	33,0702	0,66140374	5,551682	0,11103364	14,37445206	0,085625548	0,00244438	0,01437445	0,00082003
4	0,75	0,00315	1	30,8329	0,61665814	7,736483	0,154729652	20,05860774	0,079941392	0,00246388	0,02005861	0,00109869
5	1	0,0042	1	28,3469	0,56693731	9,754080	0,195081609	25,60062526	0,074399375	0,00245799	0,02560063	0,0013455
6	1,25	0,00525	1	26,2939	0,52587763	11,683925	0,23367851	30,76514008	0,06923486	0,0024304	0,03076514	0,00155338
7	1,5	0,0063	1	24,2206	0,48441118	13,468656	0,269373126	35,73610153	0,064263898	0,00238368	0,0357361	0,00173331
8	1,75	0,00735	1	22,5591	0,45118197	15,251801	0,305036026	40,33704925	0,059662951	0,00232282	0,04033705	0,00188224
9	2	0,0084	1	20,9434	0,4188683	16,942946	0,338858912	44,7204361	0,055279564	0,00224909	0,04472044	0,00200837
10	2,25	0,00945	1	19,2448	0,38489594	18,413629	0,368272571	48,89643736	0,051103563	0,00216455	0,04889644	0,00211423
11	2,5	0,0105	1	17,9593	0,35918585	20,058850	0,401177009	52,76125777	0,047238742	0,00207388	0,05276126	0,00219978
12	2,75	0,01155	1	16,4908	0,32981653	21,195371	0,42390743	56,24173462	0,043758265	0,001982	0,05624173	0,00226659
13	3	0,0126	1	15,3649	0,30729828	22,677253	0,453545054	59,61083339	0,040389167	0,00188383	0,05961083	0,00232204
14	3,25	0,01365	1	13,9776	0,27955263	23,649642	0,472992833	62,85239302	0,037147607	0,0017808	0,06285239	0,00236681
15	3,5	0,0147	1	12,9278	0,25855669	24,873245	0,497464908	65,80035653	0,034199643	0,00167981	0,06580036	0,00240023
16	3,75	0,01575	1	11,7403	0,23480531	25,556169	0,511123378	68,52174817	0,031478252	0,00158041	0,06852175	0,00242491
17	4	0,0168	1	10,7753	0,21550521	26,534702	0,530694032	71,11961558	0,028880384	0,00147999	0,07111962	0,00244294
18	4,25	0,01785	1	9,9350	0,19870033	27,469178	0,549383559	73,43876325	0,026561237	0,00138578	0,07343876	0,00245448
19	4,5	0,0189	1	9,0619	0,18123894	28,209744	0,564194883	75,68678334	0,024313217	0,00129036	0,07568678	0,00246155
20	4,75	0,01995	1	8,3009	0,16601877	28,955312	0,579106247	77,71934028	0,02228066	0,0012006	0,07771934	0,00246447
21	5	0,021	1	7,6926	0,15385183	29,729996	0,594599922	79,44398877	0,020556011	0,00112185	0,07944399	0,00246435

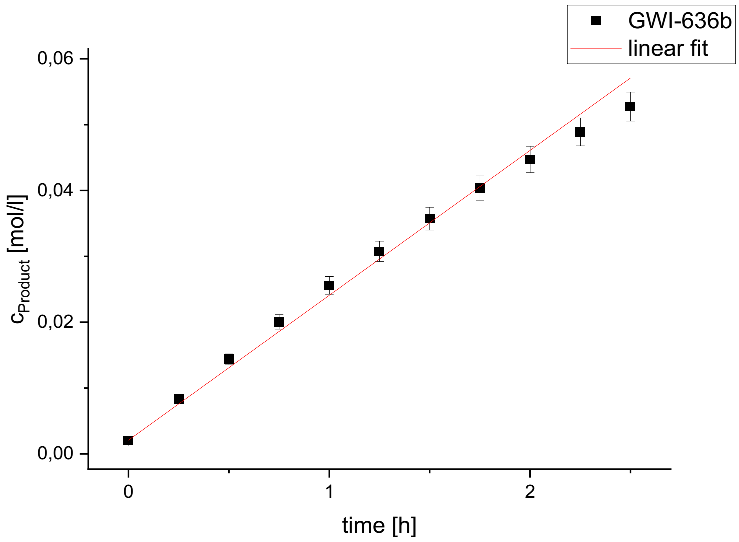

GWI-636d, BCF3, GWI-493, CDCl3, 10 mol%, 30°C

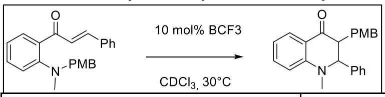

Regression (Origin)

	Value	Error		Value	Error		Value	Error
Time interva	0,25	0,0042	n [mmol]	0,05	0,00012391	(intercept)	1,9300E-03	1,4430E-04
NMR-error	0,02		Vprobe (ml)	0,5	0,01	slope	2,4110E-02	5,5823E-04
						k (mol/l*h)	2,4110E-02	5,5823E-04

Exp. Nr.	Time h	dt [h]	DCM	SM (1H)	dSM	Prod(1H)	dP	conv. [%]	c SM [mol/l]	dc SM	c P [mol/l]	dc P
1	0	0	1	68,5612	1,37122324	1,299994	0,025999879	1,860825171	0,098139175	0,00231039	0,00186083	0,00011547
2	0,25	0,00105	1	63,1841	1,26368153	6,124595	0,122491899	8,836693625	0,091163306	0,00240054	0,00883669	0,00052369
3	0,5	0,0021	1	59,5434	1,190868	10,788276	0,215765525	15,33914282	0,084660857	0,00244951	0,01533914	0,00086915
4	0,75	0,00315	1	55,4573	1,10914583	15,142285	0,302845708	21,44812483	0,078551875	0,00246471	0,02144812	0,00116288
5	1	0,0042	1	52,1432	1,0428646	19,615698	0,392313954	27,33555015	0,07266445	0,0024511	0,02733555	0,00141771
6	1,25	0,00525	1	46,9719	0,9394375	22,974672	0,459493433	32,8460414	0,067153959	0,00241325	0,03284604	0,00163111
7	1,5	0,0063	1	43,4936	0,86987239	26,694602	0,533892041	38,03287996	0,06196712	0,00235542	0,03803288	0,00180977
8	1,75	0,00735	1	39,9622	0,79924337	30,168752	0,603375037	43,01776128	0,056982239	0,00227956	0,04301776	0,0019612
9	2	0,0084	1	36,4448	0,7288966	33,145502	0,662910048	47,62946419	0,052370536	0,00219167	0,04762946	0,00208359
10	2,25	0,00945	1	32,9719	0,65943723	36,226859	0,724537182	52,35192031	0,04764808	0,00208405	0,05235192	0,00219129
11	2,5	0,0105	1	30,9012	0,61802444	38,848473	0,776969455	55,69697891	0,044303021	0,00199702	0,05569698	0,00225677
12	2,75	0,01155	1	27,1728	0,54345683	40,555497	0,811109942	59,8796575	0,040120342	0,0018756	0,05987966	0,00232607
13	3	0,0126	1	25,3008	0,50601568	42,953099	0,859061982	62,93136305	0,037068637	0,00177819	0,06293136	0,0023678
14	3,25	0,01365	1	22,3866	0,44773126	44,322354	0,886447083	66,44142336	0,033558577	0,00165693	0,06644142	0,00240658
15	3,5	0,0147	1	20,4686	0,40937147	46,480393	0,929607853	69,42660233	0,030573398	0,00154604	0,0694266	0,0024318
16	3,75	0,01575	1	21,2760	0,4255198	62,162616	1,243252323	74,50102417	0,025498976	0,0013412	0,07450102	0,00245832
17	4	0,0168	1	17,4001	0,34800234	50,375193	1,00750386	74,32676133	0,025673239	0,00134857	0,07432676	0,00245775
18	4,25	0,01785	1	15,3766	0,30753254	51,481109	1,029622171	77,00097559	0,022999024	0,0012327	0,07700098	0,00246382
19	4,5	0,0189	1	13,9770	0,27954067	52,810019	1,056200378	79,0722406	0,020927759	0,00113902	0,07907224	0,00246458
20	4,75	0,01995	1	12,9153	0,25830605	54,344699	1,086893974	80,79794472	0,019202055	0,00105836	0,08079794	0,00246259
21	5	0,021	1	12,0465	0,24093066	56,352603	1,127052067	82,38788743	0,017612113	0,00098192	0,08238789	0,00245866

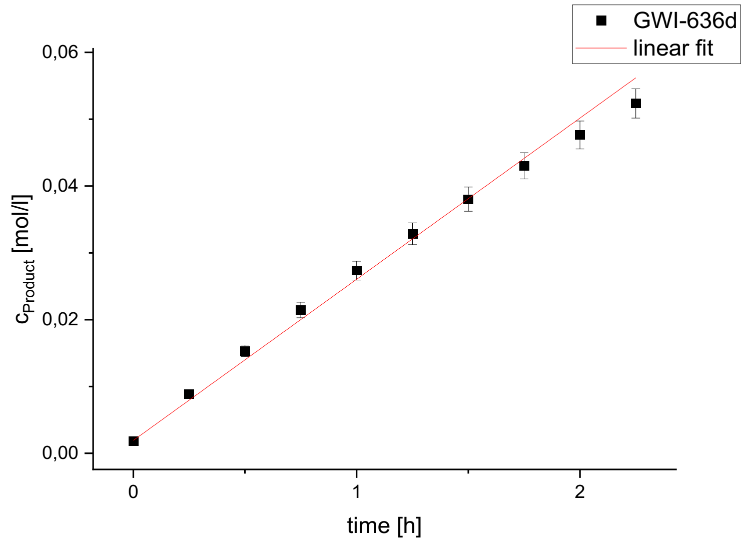

Complete kinetic data for the conversion of 1c

GWI-636g, BCF3, GWI-638, CDCl3, 10 mol%, 30°C

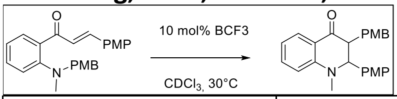

Regression (Origin)

	Value	Error		Value	Error		Value	Error
Time interval [	0,25	0,0042	n [mmol]	0,05	0,00012391	(intercept)	8,6578E-04	5,0371E-05
NMR-error	0,02		Vprobe (ml)	0,5	0,01	slope	6,6500E-03	8,6878E-05
						k (mol/l*h)	6,6500E-03	8,6878E-05

Exp. Nr.	Time h	dt [h]	DCM	SM (1H)	dSM	Prod(1H)	dP	conv. [%]	c SM [mol/l]	dc SM	c P [mol/l]	dc P
1	0	0	1	79,5060	1,59011974	0,670964	0,013419282	0,836854108	0,09916315	0,00227238	0,00083685	5,20909E-05
2	0,5	0,0021	1	44,3796	0,88759262	2,131846	0,042636923	4,583484133	0,09541652	0,00232952	0,00458348	0,000278435
3	1	0,0042	1	42,1566	0,84313289	3,712621	0,074252418	8,093918333	0,09190608	0,00237286	0,00809392	0,000480319
4	1,5	0,0063	1	40,3965	0,80792947	5,319020	0,106380395	11,63504845	0,08836495	0,0024066	0,01163505	0,000673981
5	2	0,0084	1	38,5511	0,77102178	6,825634	0,136512683	15,04214864	0,08495785	0,00242959	0,01504215	0,000850843
6	2,5	0,0105	1	37,4586	0,74917148	8,465420	0,169308407	18,43354534	0,08156645	0,00244326	0,01843355	0,001017667
7	3	0,0126	1	35,6220	0,7124407	9,897486	0,197949729	21,74338866	0,07825661	0,00244772	0,02174339	0,001171608
8	3,5	0,0147	1	33,7646	0,67529186	11,241639	0,224832771	24,97795998	0,07502204	0,00244362	0,02497796	0,001313581
9	4	0,0168	1	32,3311	0,64662173	12,655129	0,253102581	28,1311261	0,07186887	0,00243156	0,02813113	0,001443924
10	4,5	0,0189	1	31,3206	0,62641233	14,165061	0,283301223	31,14180522	0,06885819	0,00241262	0,03114181	0,001560954
11	5	0,021	1	29,6179	0,59235832	15,411463	0,308229258	34,22535087	0,06577465	0,00238571	0,03422535	0,001673299
12	5,5	0,0231	1	28,3765	0,56752918	16,760694	0,335213883	37,13281181	0,06286719	0,00235336	0,03713281	0,001772262
13	6	0,0252	1	27,0218	0,54043642	17,971169	0,359423389	39,9421539	0,06005785	0,00231569	0,03994215	0,001861461
14	6,5	0,0273	1	26,3003	0,52600669	19,623173	0,392463451	42,73012616	0,05726987	0,00227206	0,04273013	0,001943739
15	7	0,0294	1	24,6970	0,49393949	20,493666	0,409873329	45,34936001	0,05465064	0,0022254	0,04534936	0,002015372
16	7,5	0,0315	1	23,6545	0,47308925	21,827691	0,436553819	47,99177094	0,05200823	0,00217277	0,04799177	0,002082078
17	8	0,0336	1	22,5766	0,45153139	23,060983	0,461219659	50,53071781	0,04946928	0,00211694	0,05053072	0,00214091
18	8,5	0,0357	1	21,2853	0,42570536	23,916203	0,478324061	52,91023187	0,04708977	0,00205994	0,05291023	0,002191366
19	9	0,0378	1	20,2823	0,40564678	25,101865	0,502037297	55,3096952	0,0446903	0,00199786	0,0553097	0,002237659
20	9,5	0,0399	1	19,3307	0,3866135	26,281292	0,525625834	57,61929059	0,04238071	0,00193377	0,05761929	0,002277867
21	10	0,042	1	18,1976	0,36395101	27,196528	0,543930569	59,91206117	0,04008794	0,00186592	0,05991206	0,002313561
22	10,5	0,0441	1	17,4913426	08:23:45	28,56095955	0,571219191	62,01852725	0,03798147	0,00179987	0,062019	0,002342649
23	11	0,0462	1	16,3553475	07:51:02	29,29206512	0,585841302	64,17026392	0,03582974	0,00172874	0,064170	0,002368696
24	11,5	0,0483	1	15,3515235	07:22:07	30,02395562	0,600479112	66,16779856	0,0338322	0,0016594	0,066168	0,002389561
25	12	0,0504	1	14,6053926	07:00:38	31,03337693	0,620667539	67,99783875	0,03200216	0,00159306	0,067998	0,002405875
26	12,5	0,0525	1	13,6982038	06:34:30	31,78784494	0,635756899	69,88482367	0,03011518	0,00152186	0,069885	0,002419891

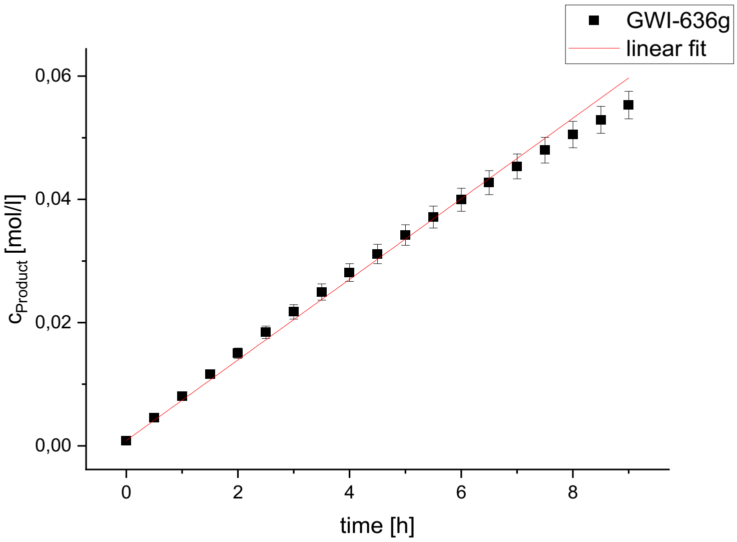

GWI-636h, BCF3, GWI-638, CDCl3, 10 mol%, 30°C

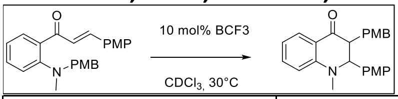

	Value	Error
Time interval [	0,25	0,0042
NMR-error	0,02	

	Value	Error
n [mmol]	0,05	0,00012391
Vprobe (ml)	0,5	0,01

Regression (Origin)

	Value	Error
(intercept)	9,0614E-04	4,8294E-05
slope	6,2000E-03	7,2964E-05
k (mol/l*h)	6,2000E-03	7,2964E-05

Exp. Nr.	Time h	dt [h]	DCM	SM (1H)	dSM	Prod(1H)	dP	conv. [%]	c SM [mol/l]	dc SM	c P [mol/l]	dc P
1	0	0	1	38,2825	0,76565025	0,338447	0,00676895	0,876331118	0,09912367	0,00227304	0,00087633	5,45343E-05
2	0,5	0,0021	1	37,0081	0,74016115	1,656529	0,033130579	4,284357073	0,09571564	0,00232537	0,00428436	0,000260776
3	1	0,0042	1	35,8068	0,71613537	2,952034	0,059040686	7,616422795	0,09238358	0,00236755	0,00761642	0,000453438
4	1,5	0,0063	1	34,4162	0,68832448	4,226972	0,084539438	10,93846351	0,08906154	0,00240076	0,01093846	0,000636678
5	2	0,0084	1	32,5393	0,65078537	5,351053	0,107021053	14,12247904	0,08587752	0,0024243	0,01412248	0,000804018
6	2,5	0,0105	1	31,7277	0,63455305	6,618722	0,13237444	17,26035932	0,08273964	0,00243957	0,01726036	0,000960999
7	3	0,0126	1	30,8208	0,61641511	7,898154	0,15796308	20,39869936	0,0796013	0,00244696	0,0203987	0,001110124
8	3,5	0,0147	1	29,1502	0,58300363	8,936713	0,178734254	23,46401015	0,07653599	0,00244658	0,02346401	0,001248172
9	4	0,0168	1	27,7760	0,55552031	10,003965	0,200079294	26,47953917	0,07352046	0,00243886	0,02647954	0,001376644
10	4,5	0,0189	1	26,6788	0,53357513	11,125794	0,222515877	29,42977435	0,07057023	0,00242428	0,02942977	0,001495294
11	5	0,021	1	25,7475	0,51495096	12,219158	0,244383165	32,18387737	0,06781612	0,00240438	0,03218388	0,001599771
12	5,5	0,0231	1	24,6324	0,49264852	13,274460	0,265489192	35,01859718	0,0649814	0,00237755	0,0350186	0,00170097
13	6	0,0252	1	23,5092	0,47018494	14,271459	0,285429188	37,77446411	0,06222554	0,00234532	0,03777446	0,001793191
14	6,5	0,0273	1	22,6317	0,45263403	15,352745	0,307054909	40,41850452	0,0595815	0,00230867	0,0404185	0,001875959
15	7	0,0294	1	21,4377	0,4287543	16,158024	0,323160473	42,97833801	0,05702166	0,00226787	0,04297834	0,001950763
16	7,5	0,0315	1	20,5825	0,41164909	17,138812	0,342776241	45,43541003	0,05456459	0,00222378	0,04543541	0,002017633
17	8	0,0336	1	19,9065	0,39812975	18,309189	0,366183786	47,91015319	0,05208985	0,00217448	0,04791015	0,002080102
18	8,5	0,0357	1	19,0202	0,38040314	19,190239	0,383804773	50,22255969	0,04977744	0,00212399	0,05022256	0,002134045
19	9	0,0378	1	18,3068	0,3661351	20,243834	0,404876687	52,51238608	0,04748761	0,00206978	0,05251239	0,002183246
20	9,5	0,0399	1	17,3251	0,34650227	21,069441	0,421388811	54,87611747	0,04512388	0,00200942	0,05487612	0,002229635
21	10	0,042	1	16,3543	0,32708539	21,743033	0,434860658	57,07236905	0,04292763	0,00194933	0,05707237	0,002268731
22	10,5	0,0441	1	15,6841074	07:31:42	22,74202574	0,454840515	59,18374777	0,04081625	0,00188793	0,059184	0,002302679
23	11	0,0462	1	15,1827901	07:17:16	23,91163278	0,478232656	61,16379533	0,0388362	0,0018271	0,061164	0,002331274
24	11,5	0,0483	1	14,2422579	06:50:11	24,42564962	0,488512992	63,1677563	0,03683224	0,00176234	0,063168	0,002357021
25	12	0,0504	1	13,5092323	06:29:04	25,23273965	0,504654793	65,13024091	0,03486976	0,00169582	0,065130	0,002379122
26	12,5	0,0525	1	12,9751823	06:13:41	26,28661624	0,525732325	66,95214483	0,03304786	0,0016313	0,066952	0,002396881

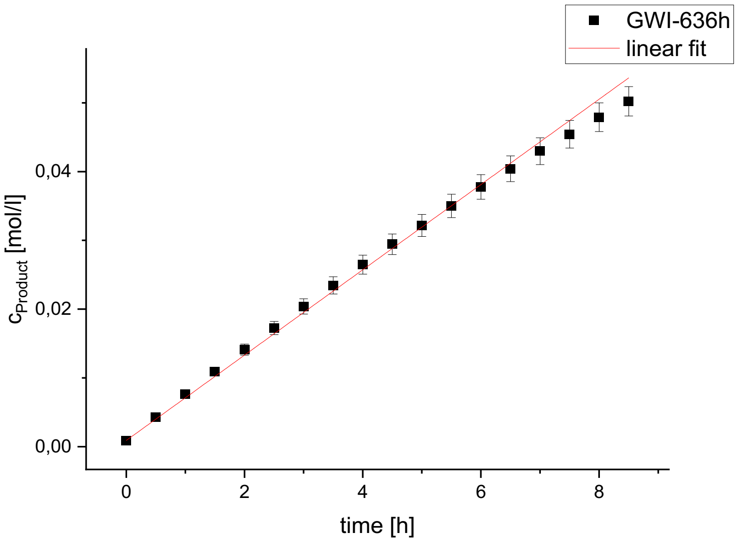

GWI-636i, BCF3, GWI-638, CDCl3, 10 mol%, 30°C

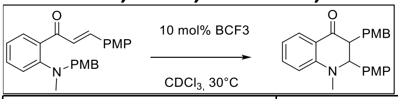

	Value	Error		Value	Error
Time interval [	0,25	0,0042	n [mmol]	0,05	0,00012391
NMR-error	0,02		Vprobe (ml)	0,5	0,01

Regression (Origin)

	Value	Error
(intercept)	7,6274E-04	3,8661E-05
slope	6,2200E-03	6,7983E-05
k (mol/l*h)	6,2200E-03	6,7983E-05

Exp. Nr.	Time h	dt [h]	DCM	SM (1H)	dSM	Prod(1H)	dP	conv. [%]	c SM [mol/l]	dc SM	c P [mol/l]	dc P
1	0	0	1	40,6295	0,81259044	0,303647	0,00607293	0,741810458	0,09925819	0,00227078	0,00074181	4,6203E-05
2	0,5	0,0021	1	38,6184	0,77236709	1,669735	0,033394702	4,144488159	0,09585551	0,0023234	0,00414449	0,000252495
3	1	0,0042	1	36,8490	0,7369795	2,961109	0,059222179	7,438087676	0,09256191	0,00236551	0,00743809	0,000443351
4	1,5	0,0063	1	36,3281	0,72656244	4,344394	0,08688788	10,6813998	0,0893186	0,0023985	0,0106814	0,000622813
5	2	0,0084	1	33,7198	0,67439572	5,489599	0,109791979	14,00072709	0,08599927	0,00242355	0,01400073	0,000797768
6	2,5	0,0105	1	32,3886	0,64777259	6,684720	0,133694396	17,10813105	0,08289187	0,00243901	0,01710813	0,000953565
7	3	0,0126	1	32,1006	0,64201256	8,128684	0,162573682	20,20587392	0,07979413	0,00244674	0,02020587	0,001101188
8	3,5	0,0147	1	30,5143	0,61028574	9,243894	0,18487787	23,25029314	0,07674971	0,00244685	0,02325029	0,001238791
9	4	0,0168	1	29,1678	0,5833551	10,400654	0,208013085	26,28524721	0,07371475	0,00243958	0,02628525	0,001368586
10	4,5	0,0189	1	27,5202	0,55040483	11,409412	0,228188238	29,30776643	0,07069223	0,00242502	0,02930777	0,001490525
11	5	0,021	1	26,3307	0,52661499	12,499298	0,249985957	32,18975675	0,06781024	0,00240433	0,03218976	0,001599988
12	5,5	0,0231	1	25,9287	0,518575	13,909369	0,278187378	34,91472332	0,06508528	0,00237865	0,03491472	0,001697375
13	6	0,0252	1	24,7311	0,49462117	14,970491	0,299409828	37,70757411	0,06229243	0,00234617	0,03770757	0,001791025
14	6,5	0,0273	1	22,9512	0,45902454	15,583336	0,311666721	40,43989274	0,05956011	0,00230836	0,04043989	0,001876606
15	7	0,0294	1	22,3900	0,44779998	16,877425	0,337548503	42,98072916	0,05701927	0,00226783	0,04298073	0,00195083
16	7,5	0,0315	1	22,3939	0,44787837	18,720047	0,374400949	45,53208858	0,05446791	0,00222194	0,04553209	0,002020165
17	8	0,0336	1	20,3881	0,40776209	18,847896	0,376957912	48,03725028	0,05196275	0,00217182	0,04803725	0,002083178
18	8,5	0,0357	1	21,5177	0,43035374	21,777268	0,435545365	50,29978243	0,04970022	0,00212223	0,05029978	0,002135772
19	9	0,0378	1	19,3378	0,38675673	21,539301	0,43078603	52,69278282	0,04730722	0,00206533	0,05269278	0,002186944
20	9,5	0,0399	1	18,3536	0,36707148	22,414636	0,448292718	54,98067252	0,04501933	0,00200665	0,05498067	0,002231584
21	10	0,042	1	17,4729	0,34945881	23,361394	0,467227877	57,21017465	0,04278983	0,00194543	0,05721017	0,002271056
22	10,5	0,0441	1	15,6679243	07:31:14	22,98825943	0,459765189	59,46851765	0,04053148	0,00187937	0,059469	0,002306984
23	11	0,0462	1	14,4776194	06:56:57	23,31918857	0,466383771	61,69618501	0,03830381	0,00181021	0,061696	0,002338428
24	11,5	0,0483	1	14,2822344	06:51:20	24,90076743	0,498015349	63,54992286	0,03645008	0,00174963	0,063550	0,002361567
25	12	0,0504	1	13,4854645	06:28:23	25,47084081	0,509416816	65,3831019	0,0346169	0,00168702	0,065383	0,002381745
26	12,5	0,0525	1	12,1239168	05:49:10	25,27466983	0,505493397	67,58188513	0,03241811	0,00160838	0,067582	0,002402402

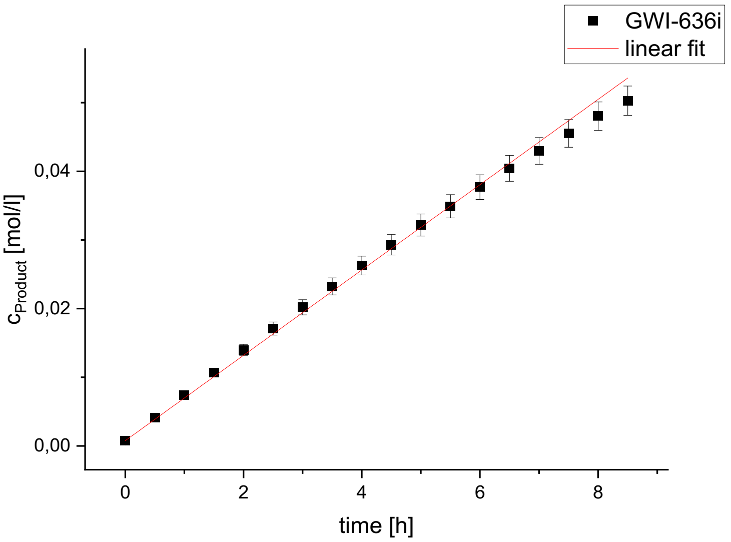

Complete kinetic data for the conversion of 1d

GWI-636j, BCF3, GWI-637b, CDCl3, 10 mol%, 30°C

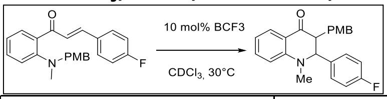

	Value	Error
Time interval [	0,25	0,0042
NMR-error	0,02	

	Value	Error
n [mmol]	0,05	0,00012391
Vprobe (ml)	0,5	0,01

Regression (Origin)

	Value	Error
(intercept)	2,1600E-03	1,7616E-04
slope	2,2430E-02	5,5137E-04
k (mol/l*h)	2,2430E-02	5,5137E-04

Exp. Nr.	Time h	dt [h]	DCM	SM (1H)	dSM	Prod(1H)	dP	conv. [%]	c SM [mol/l]	dc SM	c P [mol/l]	dc P
1	0	0	1	7,2929	0,14585736	0,153017	0,003060346	2,055058358	0,09794494	0,00230029	0,00205506	0,000127088
2	0,25	0,00105	1	7,2058	0,14411656	0,685225	0,01370451	8,683574058	0,09131643	0,00238673	0,00868357	0,000513982
3	0,5	0,0021	1	6,6960	0,13392042	1,164107	0,023282143	14,81028185	0,08518972	0,00243537	0,01481028	0,000840327
4	0,75	0,00315	1	6,2049	0,12409874	1,613246	0,032264922	20,63453928	0,07936546	0,00245377	0,02063454	0,00112272
5	1	0,0042	1	5,7433	0,11486635	2,043903	0,040878069	26,24689231	0,07375311	0,00244582	0,02624689	0,001369163
6	1,25	0,00525	1	5,3257	0,10651467	2,449082	0,048981638	31,50019368	0,06849981	0,00241555	0,03150019	0,001577009
7	1,5	0,0063	1	4,9194	0,09838873	2,823264	0,056465275	36,46355417	0,06353645	0,00236667	0,03646355	0,001753099
8	1,75	0,00735	1	4,5299	0,09059713	3,169658	0,063393163	41,16698547	0,05883301	0,00230216	0,04116699	0,001901781
9	2	0,0084	1	4,2143	0,08428586	3,514319	0,070286375	45,47153962	0,05452846	0,00222761	0,04547154	0,002022343
10	2,25	0,00945	1	3,8629	0,07725779	3,810359	0,076207177	49,65770365	0,0503423	0,00214089	0,0496577	0,002125373
11	2,5	0,0105	1	3,5584	0,07116761	4,101729	0,082034576	53,54660877	0,04645339	0,00204777	0,05354661	0,002208524
12	2,75	0,01155	1	3,3001	0,06600277	4,387885	0,087757699	57,07429388	0,04292571	0,00195283	0,05707429	0,002273487
13	3	0,0126	1	3,0036	0,06007244	4,619824	0,092396475	60,60020482	0,0393998	0,00184799	0,0606002	0,00232847
14	3,25	0,01365	1	2,7770	0,0555399	4,873977	0,097479544	63,70402479	0,03629598	0,00174748	0,06370402	0,002368639
15	3,5	0,0147	1	2,5191	0,05038242	5,062954	0,101259088	66,77531233	0,03322469	0,00164042	0,06677531	0,002400801
16	3,75	0,01575	1	2,3209	0,04641736	5,263588	0,10527176	69,39967574	0,03060032	0,00154297	0,06939968	0,002422303
17	4	0,0168	1	2,0996	0,041992	5,418209	0,108364176	72,07164742	0,02792835	0,00143809	0,07207165	0,002438536
18	4,25	0,01785	1	1,9315	0,03863089	5,616527	0,112330541	74,41009172	0,02558991	0,00134162	0,07441009	0,002448055
19	4,5	0,0189	1	1,7577	0,03515363	5,761531	0,115230623	76,62412584	0,02337587	0,00124624	0,07662413	0,002453036
20	4,75	0,01995	1	1,6086	0,03217115	5,895333	0,11790667	78,56368996	0,02143631	0,00115947	0,07856369	0,002454178
21	5	0,021	1	1,4468	0,02893603	6,016770	0,120335393	80,61515982	0,01938484	0,00106441	0,08061516	0,00245211

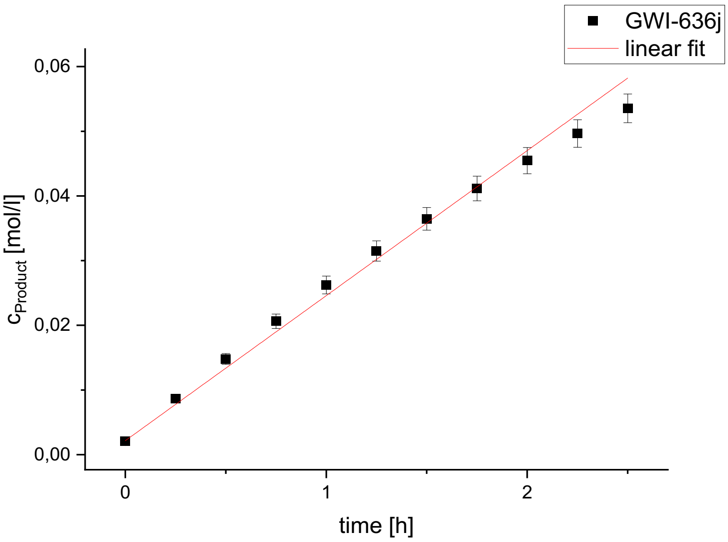

GWI-636k, BCF3, GWI-637b, CDCl3, 10 mol%, 30°C

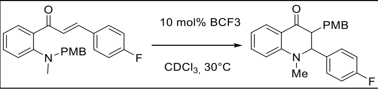

Regression (Origin)

	Value	Error		Value	Error		Value	Error
Time interval [	0,25	0,0042	n [mmol]	0,05	0,00012391	(intercept)	2,4000E-03	1,9074E-04
NMR-error	0,02		Vprobe (ml)	0,5	0,01	slope	2,7270E-02	7,2384E-04
						k (mol/l*h)	2,7270E-02	7,2384E-04

Exp. Nr.	Time h	dt [h]	DCM	SM (1H)	dSM	Prod(1H)	dP	conv. [%]	c SM [mol/l]	dc SM	c P [mol/l]	dc P
1	0	0	1	12,0584	0,24116851	0,285223	0,005704459	2,31068584	0,09768931	0,00230428	0,00231069	0,00014266
2	0,25	0,00105	1	11,3083	0,22616502	1,293370	0,025867393	10,26351842	0,08973648	0,00240215	0,01026352	0,000601012
3	0,5	0,0021	1	10,1803	0,20360568	2,170556	0,043411111	17,57415365	0,08242585	0,00244749	0,01757415	0,000977718
4	0,75	0,00315	1	9,3795	0,1875894	3,037757	0,060755145	24,46405483	0,07553595	0,00245108	0,02446405	0,001293608
5	1	0,0042	1	8,5377	0,17075384	3,810690	0,076213796	30,85983152	0,06914017	0,00242042	0,03085983	0,001552855
6	1,25	0,00525	1	7,7512	0,15502366	4,546062	0,09092124	36,96813445	0,06303187	0,00236059	0,03696813	0,001769897
7	1,5	0,0063	1	6,9949	0,13989778	5,198018	0,10396036	42,63149059	0,05736851	0,00227846	0,04263149	0,001944463
8	1,75	0,00735	1	6,3862	0,12772413	5,851825	0,117036499	47,81671796	0,05218328	0,00218075	0,04781672	0,002081789
9	2	0,0084	1	5,7489	0,11497704	6,420614	0,12841227	52,76002971	0,04723997	0,00206758	0,05276003	0,002192682
10	2,25	0,00945	1	5,2172	0,10434351	6,973947	0,13947893	57,20512463	0,04279488	0,00194912	0,05720512	0,002275705
11	2,5	0,0105	1	4,6657	0,093314	7,433960	0,148679198	61,43941289	0,03856059	0,00182158	0,06143941	0,002340091
12	2,75	0,01155	1	4,1886	0,08377192	7,904983	0,158099658	65,36512537	0,03463487	0,00169051	0,06536513	0,00238697
13	3	0,0126	1	3,7415	0,07482981	8,277584	0,16555169	68,87039493	0,03112961	0,00156307	0,06887039	0,00241841
14	3,25	0,01365	1	3,3575	0,06714996	8,664409	0,173288179	72,07183415	0,02792817	0,00143808	0,07207183	0,002438537
15	3,5	0,0147	1	2,9896	0,05979129	8,962876	0,179257513	74,9878302	0,02501217	0,00131711	0,07498783	0,002449733
16	3,75	0,01575	1	2,7215	0,05443036	9,320094	0,186401882	77,39905743	0,02260094	0,00121193	0,07739906	0,002453853
17	4	0,0168	1	2,3857	0,04771368	9,468707	0,189374141	79,87510327	0,0201249	0,00109909	0,0798751	0,002453244
18	4,25	0,01785	1	2,1511	0,04302212	9,801570	0,196031395	82,00314358	0,01799686	0,00099819	0,08200314	0,002448801
19	4,5	0,0189	1	1,9783	0,03956687	10,071054	0,201421084	83,58138964	0,01641861	0,00092102	0,08358139	0,002443166
20	4,75	0,01995	1	1,7534	0,03506855	10,246169	0,204923376	85,38761189	0,01461239	0,00083026	0,08538761	0,002434272
21	5	0,021	1	1,5804	0,0316079	10,430406	0,20860812	86,8418843	0,01315812	0,00075528	0,08684188	0,002425215

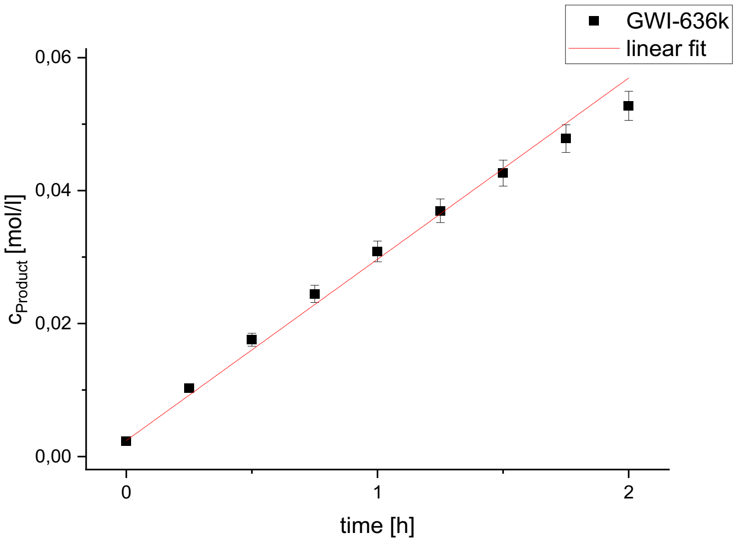

GWI-636l, BCF3, GWI-637b, CDCl3, 10 mol%, 30°C

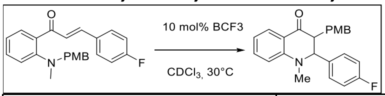

Regression (Origin)

	Value	Error		Value	Error		Value	Error
Time interval [	0,25	0,0042	n [mmol]	0,05	0,00012391	(intercept)	2,8300E-03	2,1357E-04
NMR-error	0,02		Vprobe (ml)	0,5	0,01	slope	2,7590E-02	7,1402E-04
						k (mol/l*h)	2,7590E-02	7,1402E-04

Exp. Nr.	Time h	dt [h]	DCM	SM (1H)	dSM	Prod(1H)	dP	conv. [%]	c SM [mol/l]	dc SM	c P [mol/l]	dc P
1	0	0	1	13,0695	0,26139089	0,364760	0,007295194	2,715136386	0,09728486	0,00231048	0,00271514	0,000167191
2	0,25	0,00105	1	12,2205	0,2444094	1,467329	0,02934657	10,71997458	0,08928003	0,00240623	0,01071997	0,000625784
3	0,5	0,0021	1	11,1698	0,22339697	2,478497	0,049569945	18,15968986	0,08184031	0,00244927	0,01815969	0,001006041
4	0,75	0,00315	1	10,2277	0,20455443	3,429247	0,068584947	25,1098715	0,07489013	0,00244947	0,02510987	0,001321271
5	1	0,0042	1	9,2307	0,18461453	4,287629	0,08575257	31,71708805	0,06828291	0,00241383	0,03171709	0,001585116
6	1,25	0,00525	1	8,4026	0,16805248	5,105513	0,102110264	37,79583409	0,06220417	0,00235019	0,03779583	0,001797011
7	1,5	0,0063	1	7,5502	0,15100456	5,830736	0,116614716	43,57485683	0,05642514	0,00226228	0,04357486	0,001971048
8	1,75	0,00735	1	6,8017	0,13603414	6,520753	0,130415066	48,94556428	0,05105444	0,00215663	0,04894556	0,002108835
9	2	0,0084	1	6,1389	0,12277839	7,177572	0,143551444	53,89987406	0,04610013	0,00203871	0,05389987	0,002215478
10	2,25	0,00945	1	5,5677	0,11135311	7,794174	0,155883489	58,33163914	0,04166836	0,00191659	0,05833164	0,002294235
11	2,5	0,0105	1	4,9745	0,09949019	8,309243	0,166184867	62,55192724	0,03744807	0,00178569	0,06255193	0,002354628
12	2,75	0,01155	1	4,4652	0,08930375	8,799353	0,175987055	66,33741113	0,03366259	0,00165615	0,06633741	0,002396676
13	3	0,0126	1	3,9801	0,07960243	9,245595	0,184911898	69,90619257	0,03009381	0,00152353	0,06990619	0,002425819
14	3,25	0,01365	1	3,5537	0,07107342	9,643864	0,192877278	73,07322131	0,02692678	0,00139731	0,07307322	0,002443149
15	3,5	0,0147	1	3,1528	0,06305675	9,998753	0,199975064	76,02694825	0,02397305	0,00127235	0,07602695	0,002452079
16	3,75	0,01575	1	2,7707	0,0554147	10,286255	0,205725095	78,77968006	0,02122032	0,00114962	0,07877968	0,002454119
17	4	0,0168	1	2,4962	0,04992341	10,618086	0,212361716	80,96597686	0,01903402	0,00104782	0,08096598	0,002451419
18	4,25	0,01785	1	2,1748	0,04349588	10,822647	0,216452941	83,2675214	0,01673248	0,00093653	0,08326752	0,002444446
19	4,5	0,0189	1	1,9332	0,03866311	11,082562	0,221651246	85,14752955	0,01485247	0,00084247	0,08514753	0,002435605
20	4,75	0,01995	1	1,7895	0,0357905	11,374537	0,227490747	86,40598335	0,01359402	0,00077793	0,08640598	0,002428107
21	5	0,021	1	1,4876	0,0297517	11,445023	0,228900464	88,49741072	0,01150259	0,00066787	0,08849741	0,002412844

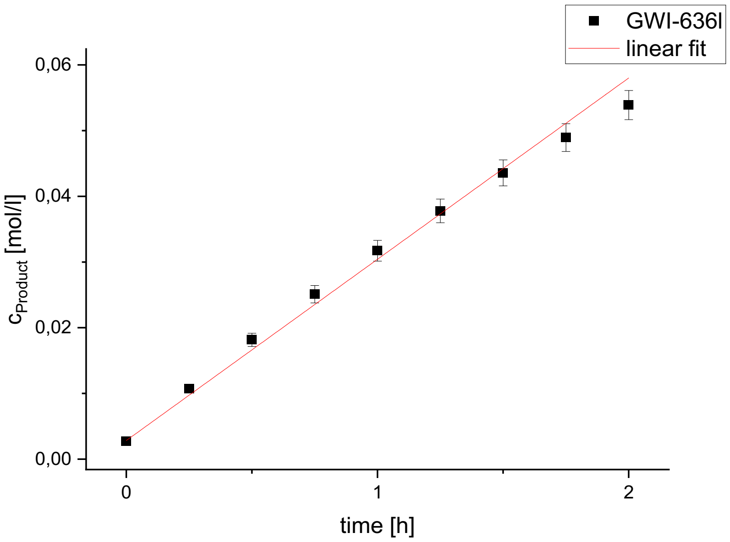

5.2 Control experiments

5.2.1 NMR scale experiment with radical scavenger

Starting material **1b** (17.9 mg, 50.0 µmol, 1.00 equiv.), TEMPO (7.8 mg, 50.0 µmol, 1.00 equiv.) and B(2,3,6-F_3-C_6H_2)$_3$ (2.00 mg, 5.00 µmol, 0.100 equiv.) were dissolved in $CDCl_3$ and the reaction was monitored by ^{1}H-NMR spectroscopy at 30 °C. ^{1}H-NMR spectroscopy showed quantitative conversion of starting material **1b** to **2b**.

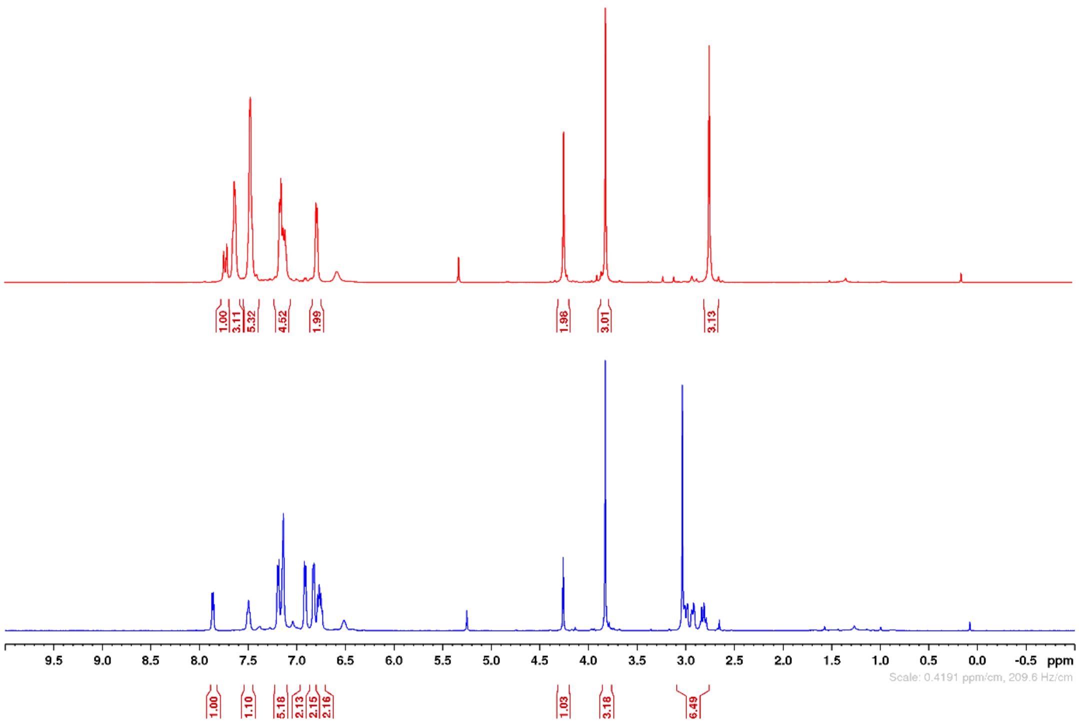

Figure S3: Reaction of 1b and B(2,3,6-F_3-C_6H_2)$_3$ with 1.00 equiv. TEMPO after mixing (red) and after complete conversion (blue).

6 Crystallographic Data

rac-trans-2,3-1-methyl-2-phenyl-3-(cis-1-phenylethyl)-2,3-dihydroquinolin-4(1H)-one (*rac*-**2a)**

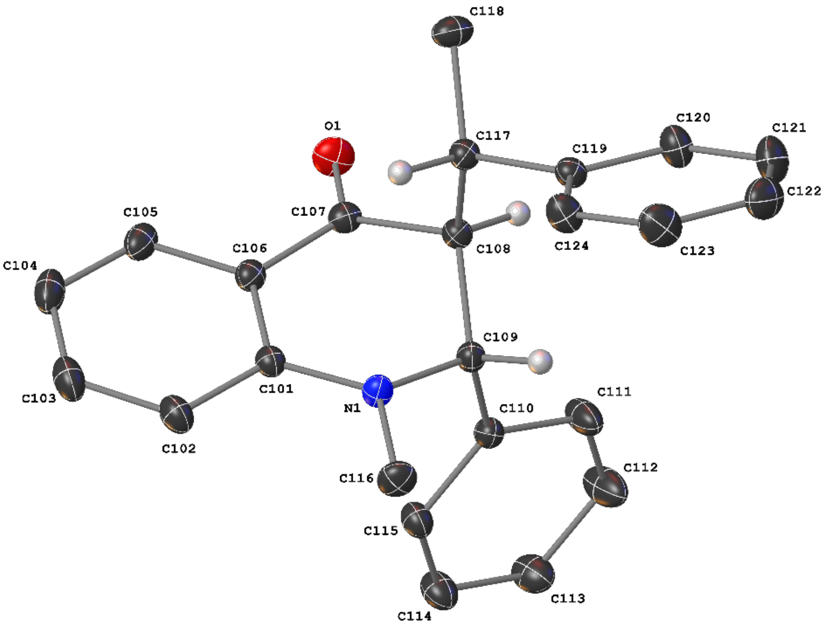

($C_{24}H_{23}NO$), M_r = 341.43 Da, colourless block, size: 0.30 x 0.18 x 0.10 mm³, monoclinic space group $C2/c$ with Z = 8, a = 18.4937(14) Å, b = 10.9801(8) Å, c = 17.9771(13) Å, β = 96.007(2)°, V = 3630.4(5) Å³, D_c = 1.249 mg/m³, μ = 0.075 mm^{-1}, F(000) = 1456, 2.160° ≤ θ ≤ 33.214°, reflections collected: 245465, independent reflections: 6952, R_{int} = 0.0550, refinement converged at R1 = 0.0412 [I>2σ(I)], wR2 = 0.1184 [all data], min./max. ΔF: -0.187 eÅ^{-3} (0.61 Å from C109) / 0.467 eÅ^{-3} (0.69 Å from C110), **CCDC-No.: 2127718.**

CCDC-No.	2127718	
Empirical formula	C24 H23 N O	
Formula weight	341.43	
Temperature	120(2) K	
Wavelength	0.71073 Å	
Crystal system	Monoclinic	
Space group	C2/c	
Unit cell dimensions	a = 18.4937(14) Å	α = 90°
	b = 10.9801(8) Å	β = 96.007(2)°
	c = 17.9771(13) Å	γ = 90°
Volume	3630.4(5) Å^3	
Z	8	
Density (calculated)	1.249 Mg/m^3	
Absorption coefficient	0.075 mm^{-1}	
F(000)	1456	
Crystal size	0.300 x 0.180 x 0.100 mm^3	

Theta range for data collection	2.160 to 33.214°.
Index ranges	-28<=h<=28, -16<=k<=16, -27<=l<=27
Reflections collected	245465
Independent reflections	6952 [R(int) = 0.0550]
Completeness to theta = 25.242°	99.6 %
Absorption correction	Semi-empirical from equivalents
Refinement method	Full-matrix least-squares on F^2
Data / restraints / parameters	6952 / 0 / 237
Goodness-of-fit on F^2	1.082
Final R indices [I>2sigma(I)]	R1 = 0.0412, wR2 = 0.1085
R indices (all data)	R1 = 0.0500, wR2 = 0.1184
Largest diff. peak and hole	0.467 and -0.187 eÅ^{-3}

(2S,3R)-1-methyl-2-phenyl-3-((R)-1-phenylethyl)-2,3-dihydroquinolin-4(1H)-one ((2S,3R)-**2a**)

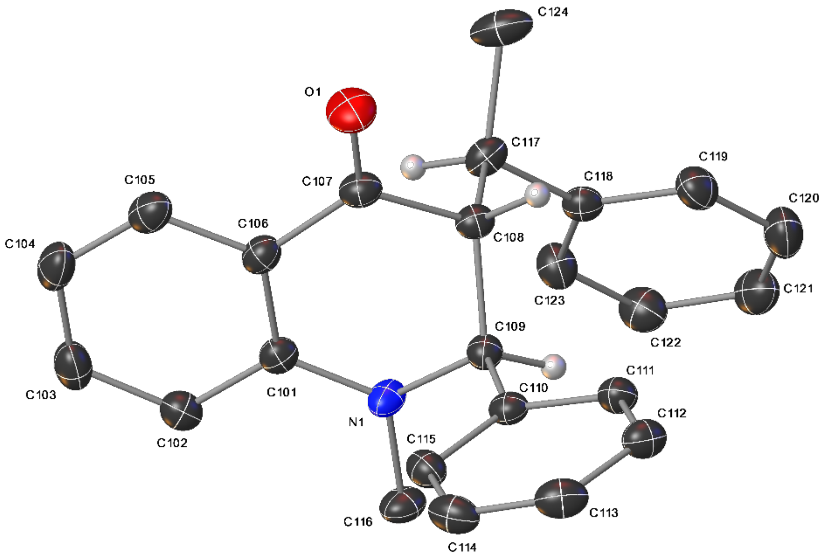

($C_{49}H_{48}Cl_2N_2O_2$), M_r = 767.79 Da, colourless block, size: 0.30 x 0.28 x 0.26 mm³, monoclinic space group $P2_1$ with Z = 2, a = 10.5193(6) Å, b = 9.7849(5) Å, c = 19.7922(11) Å, β = 99.8690(10)°, V = 2007.07(19) Å³, D_c = 1.270 mg/m³, μ = 1.781 mm^{-1}, F(000) = 812, 2.266° ≤ θ ≤ 74.785°, reflections collected: 50277, independent reflections: 8037, R_{int} = 0.0246, refinement converged at R1 = 0.0350 [I>2σ(I)], wR2 = 0.1011 [all data], min./max. ΔF: -0.453 eÅ^{-3} (0.58 Å from Cl12) / 0.592 eÅ^{-3} (1.34 Å from Cl12), **CCDC-No.: 2127715.**

CCDC-No.	2127715	
Empirical formula	C49 H48 Cl2 N2 O2	
Formula weight	767.79	
Temperature	120(2) K	
Wavelength	1.54178 Å	
Crystal system	Monoclinic	
Space group	$P2_1$	
Unit cell dimensions	a = 10.5193(6) Å	α = 90°
	b = 9.7849(5) Å	β = 99.8690(10)°
	c = 19.7922(11) Å	γ = 90°
Volume	2007.07(19) Å^3	
Z	2	
Density (calculated)	1.270 Mg/m^3	
Absorption coefficient	1.781 mm^{-1}	
F(000)	812	
Crystal size	0.30 x 0.28 x 0.26 mm^3	
Theta range for data collection	2.266 to 74.785°.	
Index ranges	-13<=h<=13, -11<=k<=12, -24<=l<=23	
Reflections collected	50227	

Independent reflections	8037 [R(int) = 0.0246]
Completeness to theta = 67.679°	99.0 %
Absorption correction	Semi-empirical from equivalents
Refinement method	Full-matrix least-squares on F^2
Data / restraints / parameters	8037 / 1 / 500
Goodness-of-fit on F^2	1.060
Final R indices [I>2sigma(I)]	R1 = 0.0350, wR2 = 0.1005
R indices (all data)	R1 = 0.0354, wR2 = 0.1011
Absolute structure parameter	0.056(3)
Largest diff. peak and hole	0.592 and -0.453 eÅ^{-3}

rac-*trans*-2,3-3-(4-methoxybenzyl)-1-methyl-2-(pyridin-2-yl)-2,3-dihydroquinolin-4(1H)-one (**2j**)

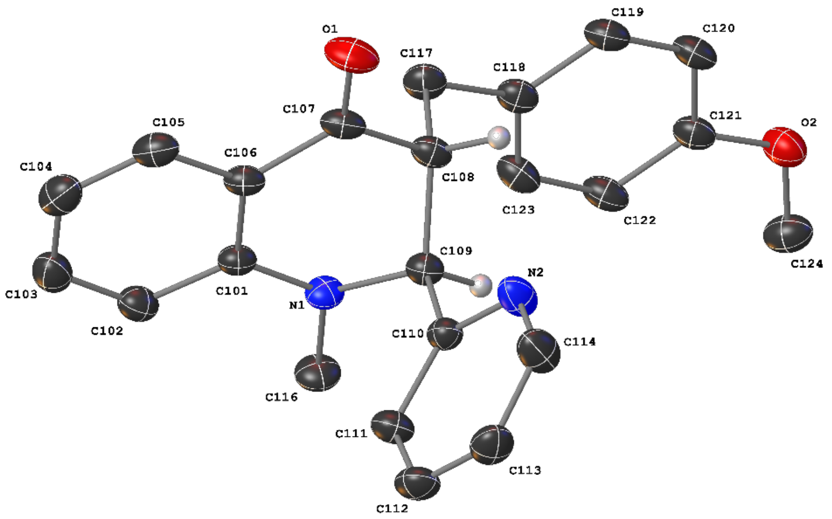

($C_{23}H_{22}N_2O_2$), M_r = 358.42 Da, colourless block, size: 0.30 x 0.24 x 0.10 mm³, monoclinic space group $P2_1/c$ with Z = 4, a = 10.2249(7) Å, b = 21.8948(13) Å, c = 8.4780(5) Å, β = 106.003(2)°, V = 1824.4(2) Å³, D_c = 1.305 mg/m³, μ = 0.084 mm^{-1}, F(000) = 760, 2.072° ≤ θ ≤ 32.049°, reflections collected: 150446, independent reflections: 6358, R_{int} = 0.0518, refinement converged at R1 = 0.0424 [I>2σ(I)], wR2 = 0.1274 [all data], min./max. ΔF: -0.198 eÅ^{-3} (0.66 Å from C121) / 0.381 eÅ^{-3} (0.72 Å from C106), **CCDC-No.: 2127716.**

CCDC-No.	2127716	
Empirical formula	C23 H22 N2 O2	
Formula weight	358.42	
Temperature	124(2) K	
Wavelength	0.71073 Å	
Crystal system	Monoclinic	
Space group	$P2_1/c$	
Unit cell dimensions	a = 10.2249(7) Å	α = 90°
	b = 21.8948(13) Å	β = 106.003(2)°
	c = 8.4780(5) Å	γ = 90°
Volume	1824.4(2) Å^3	
Z	4	
Density (calculated)	1.305 Mg/m^3	
Absorption coefficient	0.084 mm^{-1}	
F(000)	760	
Crystal size	0.300 x 0.240 x 0.100 mm^3	
Theta range for data collection	2.072 to 32.049°.	
Index ranges	-15<=h<=15, -32<=k<=32, -12<=l<=12	
Reflections collected	150446	

Independent reflections	6358 [R(int) = 0.0518]
Completeness to theta = 25.242°	99.7 %
Absorption correction	Semi-empirical from equivalents
Refinement method	Full-matrix least-squares on F^2
Data / restraints / parameters	6358 / 0 / 246
Goodness-of-fit on F^2	1.088
Final R indices [I>2sigma(I)]	R1 = 0.0424, wR2 = 0.1105
R indices (all data)	R1 = 0.0582, wR2 = 0.1274
Largest diff. peak and hole	0.381 and -0.198 eÅ^{-3}

rac-*trans*-2,3-3-(4-fluorobenzyl)-2-(4-fluorophenyl)-1-methyl-2,3-dihydroquinolin-4(1H)-one **(2q)**

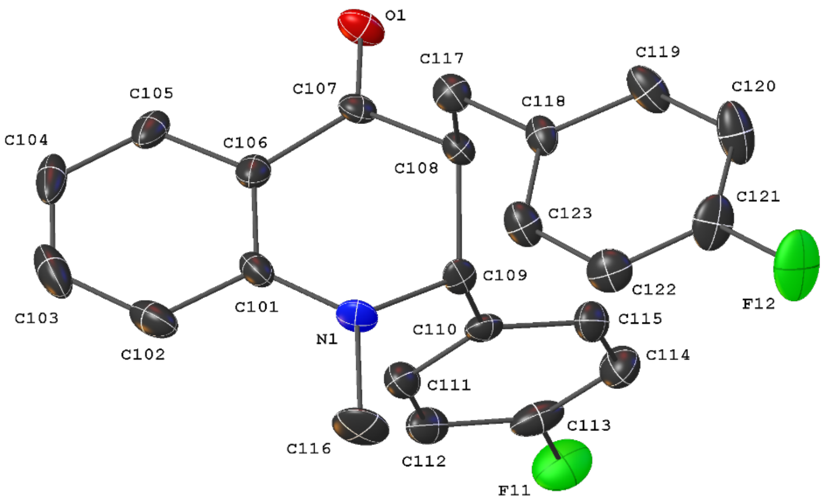

($C_{23}H_{19}F_2NO$), M_r = 363.39 Da, colourless block, size: 0.22 x 0.15 x 0.10 mm³, monoclinic space group $P2_1$ with Z = 8, a = 10.731(12) Å, b = 27.644(3) Å, c = 12.8837(16) Å, β = 90.433(2)°, V = 3623.1(7) Å³, D_c = 1.332 mg/m³, μ = 0.095 mm^{-1}, F(000) = 1520, 2.002° ≤ θ ≤ 27.993°, reflections collected: 92923, independent reflections: 17450, R_{int} = 0.0712, refinement converged at R1 = 0.634 [I>2σ(I)], wR2 = 0.1453 [all data], min./max. ΔF: -0.260 eÅ^{-3} (1.30 Å from C419) / 0.369 eÅ^{-3} (0.90 Å from N4), **CCDC-No.: 2127719.**

CCDC-No.	2127719	
Empirical formula	C23 H19 F2 N O	
Formula weight	363.39	
Temperature	120(2) K	
Wavelength	0.71073 Å	
Crystal system	Monoclinic	
Space group	$P2_1$	
Unit cell dimensions	a = 10.1731(12) Å	α = 90°
	b = 27.644(3) Å	β = 90.433(2)°
	c = 12.8837(16) Å	γ = 90°
Volume	3623.1(7) Å^3	
Z	8	
Density (calculated)	1.332 Mg/m^3	
Absorption coefficient	0.095 mm^{-1}	
F(000)	1520	
Crystal size	0.220 x 0.150 x 0.100 mm^3	
Theta range for data collection	2.002 to 27.993°.	
Index ranges	-13<=h<=13, -36<=k<=36, -16<=l<=17	
Reflections collected	92923	

Independent reflections	17450 [R(int) = 0.0712]
Completeness to theta = 25.242°	99.9 %
Absorption correction	Semi-empirical from equivalents
Refinement method	Full-matrix least-squares on F^2
Data / restraints / parameters	17450 / 1 / 978
Goodness-of-fit on F^2	1.132
Final R indices [I>2sigma(I)]	R1 = 0.0634, wR2 = 0.1411
R indices (all data)	R1 = 0.0712, wR2 = 0.1453
Absolute structure parameter	-0.2(2)
Largest diff. peak and hole	0.369 and -0.260 eÅ^{-3}

((1,1-dimethyl-2-phenyl-1,2,3,4-tetrahydroquinolin-1-ium-4-yl)oxy)tris(perfluorophenyl)borate (**3**)

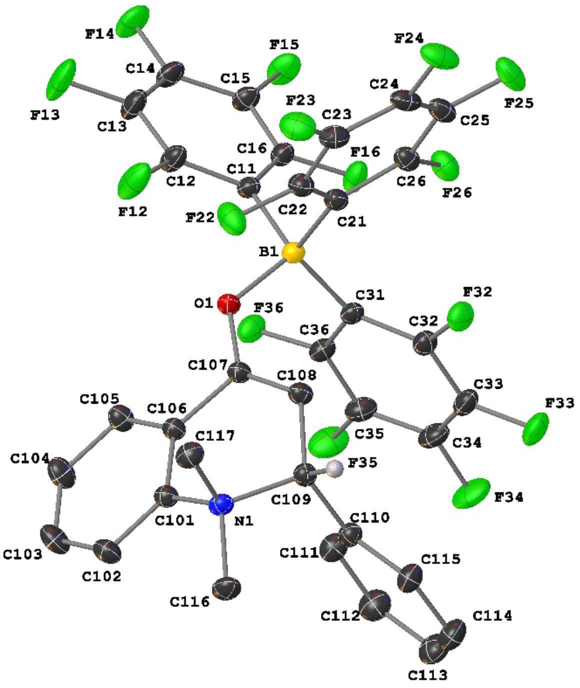

($C_{35}H_{17}BF_{15}NO$), M_r = 763.30 Da, colourless block, size: 0.28 x 0.24 x 0.12 mm³, monoclinic space group $P2_1/n$ with Z = 4, a = 12.8492(8) Å, b = 12.7654(8) Å, c = 19.1721(11) Å, β = 101.983(2)°, V = 3076.2(3) Å³, D_c = 1.648 mg/m³, μ = 0.162 mm^{-1}, F(000) = 1528, 2.130° ≤ θ ≤ 32.113°, reflections collected: 350181, independent reflections: 10743, R_{int} = 0.0364, refinement converged at R1 = 0.0369 [I>2σ(I)], wR2 = 0.1165 [all data], min./max. ΔF: -0.332 eÅ^{-3} (0.76 Å from F13) / 0.454 eÅ^{-3} (0.69 Å from C106), **CCDC-No.: 2127717.**

CCDC-No.	2127717	
Empirical formula	C35 H17 B F15 N O	
Formula weight	763.30	
Temperature	120(2) K	
Wavelength	0.71073 Å	
Crystal system	Monoclinic	
Space group	$P2_1/n$	
Unit cell dimensions	a = 12.8492(8) Å	α = 90°
	b = 12.7654(8) Å	β = 101.983(2)°
	c = 19.1721(11) Å	γ = 90°
Volume	3076.2(3) Å^3	
Z	4	
Density (calculated)	1.648 Mg/m^3	
Absorption coefficient	0.162 mm^{-1}	
F(000)	1528	
Crystal size	0.280 x 0.240 x 0.120 mm^3	
Theta range for data collection	2.130 to 32.113°.	
Index ranges	-19<=h<=19, -19<=k<=19, -28<=l<=28	
Reflections collected	350181	
Independent reflections	10743 [R(int) = 0.0364]	

Completeness to theta = 25.242°	99.8 %
Absorption correction	Semi-empirical from equivalents
Refinement method	Full-matrix least-squares on F^2
Data / restraints / parameters	10743 / 0 / 480
Goodness-of-fit on F^2	1.061
Final R indices [I>2sigma(I)]	R1 = 0.0369, wR2 = 0.1045
R indices (all data)	R1 = 0.0457, wR2 = 0.1165
Largest diff. peak and hole	0.454 and -0.332 eÅ^{-3}

7 Computational chemistry

Density functional theory computations were carried out as implemented with the ORCA program 4.2.1.[QC1] The structures were preoptimized with the tight binding method included in the xtb-gfn2 package version 6.3.3 by S. Grimme.[QC2] Geometries were refined by the PBEh-3c functional with the implemented modified basis set def2-mSVP including dispersion correction D3BJ. Frequency calculations were conducted at the same level of theory providing access to thermodynamic data at 298.15 K. Minimum structures were characterized by the absence of imaginary frequencies, transition states were identified by the presence of one imaginary frequency along the reaction trajectory. Final energy evaluation and solvent contributions (SMD, chloroform)[QC3] were calculated at the PW6B95[QC4] level with the large basis def2-QZVPP for quantitative analysis.[QC5]

The transition structures TS_2 and TS_2' were further optimized by restricted (RKS) and unrestricted (UKS) Kohn-Sham using the wB97X-D3BJ[QC6] functional with def2-TZVP basis set. Energy evaluation was conducted at the same level of theory without solvent correction.

[QC1] Neese, F.; Wennmohs, F.; Becker, U.; Riplinger, C. *J. Chem. Phys.* **2020**, *152*, 224108.

[QC2] C. Bannwarth, E. Caldeweyher, S. Ehlert, A. Hansen, P. Pracht, J. Seibert, S. Spicher, S. Grimme, *WIREs Comput. Mol. Sci.,* **2020**, e01493. DOI: 10.1002/wcms.1493; S. Grimme, C. Bannwarth, P. Shushkov, *J. Chem. Theory Comput.*, **2017**, *13*, 1989-2009; E. Caldeweyher, C. Bannwarth and S. Grimme, *J. Chem. Phys.*, **2017**, *147*, 034112; E. Caldeweyher, S. Ehlert, A. Hansen, H. Neugebauer, S. Spicher, C. Bannwarth and S. Grimme, *J. Chem. Phys.,* **2019**, *150*, 154122; E. Caldeweyher, J.-M. Mewes, S. Ehlert and S. Grimme, *Phys. Chem. Chem. Phys.* **2020**, *22*, 8499-8512.

[QC3] Marenich, A. V.; Cramer, C. J.; Truhlar, D. G. *J. Phys. Chem. B* **2009**, *113*, 6378–6396.

[QC4] Y. Zhao and D. G. Truhlar, *J. Phys. Chem. A*, **2005**, *109*, 5656–5667

[QC5] Hellweg, A.; Hättig, C.; Höfener, S.; Klopper, W. *Theor. Chem. Acc.* **2007**, *117*, 587–597; Grimme, S.; Brandenburg, J. G.; Bannwarth, C.; Hansen, A. *J. Chem. Phys.* **2015**, *143*, 054107; Stoychev, G. L.; Auer, A. A.; Neese, F. *J. Chem. Theory Comput.* **2017**, *13*, 554–562; Kruse, H.; Grimme, S. *J. Chem. Phys.* **2012**, *136*, 154101; Grimme, S.; Ehrlich, S.; Goerigk, L. *J. Comput. Chem.* **2011**, *32*, 1456–1465; Grimme, S.; Antony, J.; Ehrlich, S.; Krieg, H. *J. Chem. Phys.* **2010**, *132*, 154104; Weigend, F. *J. Comput. Chem.* **2008**, *29*, 167–175; Weigend, F. *Phys. Chem. Chem. Phys.* **2006**, *8*, 1057–1065; Weigend, F.; Ahlrichs, R. *Phys. Chem. Chem. Phys.* **2005**, *7*, 3297–3305.

[QC6] J.-D. Chai, M. Head-Gordon, *J. Chem. Phys.* **2008**, *128*, 084106.

Table S3. *Summarized computational details (coordinates can be accessed from submitted coordinates.xyz file).*

	**neg. frequency / cm^{-1}**	**PW6B95-D3BJ def2-QZVPP SMD $CHCl_3$**	**PBEh-3c def2-mSVP**					
		**E / Eh**	**thermo corr / Eh**	**corr ΔH /Eh**	**corr ΔS / Eh**	**ΔG / Eh**	**ΔΔG / Eh**	**ΔΔG / kcal•mol^{-1}**
(*S*)-**1a**		-1060,2508	0,00094421	0,4428	0,0715	-1059,8785		
$B(C_6F_5)_3$		-2211,8864	0,00094421	0,1893	0,0844	-2211,7805		
(*S*)-**1a**•$B(C_6F_5)_3$		-3272,1770	0,00094421	0,6354	0,1280	-3271,6685	-0,0095	-5,9673
**TS$_1$**	-310,3	-3272,1509	0,00094421	0,6348	0,1252	-3271,6404	0,0187	11,7094
**TS$_1$'**	-339,4	-3272,1480	0,00094421	0,6348	0,1242	-3271,6365	0,0226	14,1542
**INT**		-3272,1813	0,00094421	0,6373	0,1240	-3271,6670	-0,0080	-4,9945
**INT'**		-3272,1808	0,00094421	0,6374	0,1237	-3271,6661	-0,0071	-4,4662
**stacked-TS$_2$** (UKS)	-64,0	-3272,1583	0,00094421	0,6330	0,1252	-3271,6496	0,0095	5,9343
**stacked-TS$_2$** (RKS)	-63,8	-3272,1584	0,00094421	0,6330	0,1252	-3271,6496	0,0094	5,9122
**TS$_2$** (RKS)	-99,0	-3272,1475	0,00094421	0,6324	0,1256	-3271,6398	0,0193	12,0815
**TS$_2$'** (UKS)	-101,0	-3272,1520	0,00094421	0,6329	0,1254	-3271,6436	0,0155	9,7043
**TS$_2$'** (RKS)	-100,7	-3272,1519	0,00094421	0,6329	0,1255	-3271,6436	0,0155	9,7112
**stacked-TS$_2$‘** (RKS)	-72,8	-3272.1521	0,00094421	0,6329	0,1245	-3271,6428	0,0162	10,1614
(2*S*,3*R*)-**2a**•$B(C_6F_5)_3$		-3272,2082	0,00094421	0,6372	0,1246	-3271,6947	-0,0357	-22,3779
(2*R*,3*S*)-**2a**•$B(C_6F_5)_3$		-3272,2076	0,00094421	0,6372	0,1247	-3271,6942	-0,0351	-22,0443
(2*S*,3*R*)-**2a**		-1060,2844	0,00094421	0,4444	0,0694	-1059,9084	-0,0299	-18,7564
(2*R*,3*S*)-**2a**		-1060,2848	0,00094421	0,4444	0,0693	-1059,9088	-0,0303	-18,9945
**wB95X-D3BJ/def2-TZVP optimized**								
stacked-TS$_2$ (UKS)	-69,3	-3268,8402	0,00094421	0,6212	0,1267	-3268,3447		0,0000
stacked-TS$_2$ (RKS)	-74,1	-3268,8402	0,00094421	0,6213	0,1269	-3268,3449	-0,0002	-0,1305
TS$_2$' (UKS)	-139,0	-3268,8402	0,00094421	0,6212	0,1267	-3268,3447		0,0000
TS$_2$' (RKS)	-142,4	-3268,8402	0,00094421	0,6213	0,1265	-3268,3445	0,0003	0,1612

Figure S1: *Lowest free energy (kcal/mol) trajectories (PW6B95/def2-QZVPP//PBEh-3c/def2-mSVP) for (S)-**1a** → (2S,3R)-**2a***

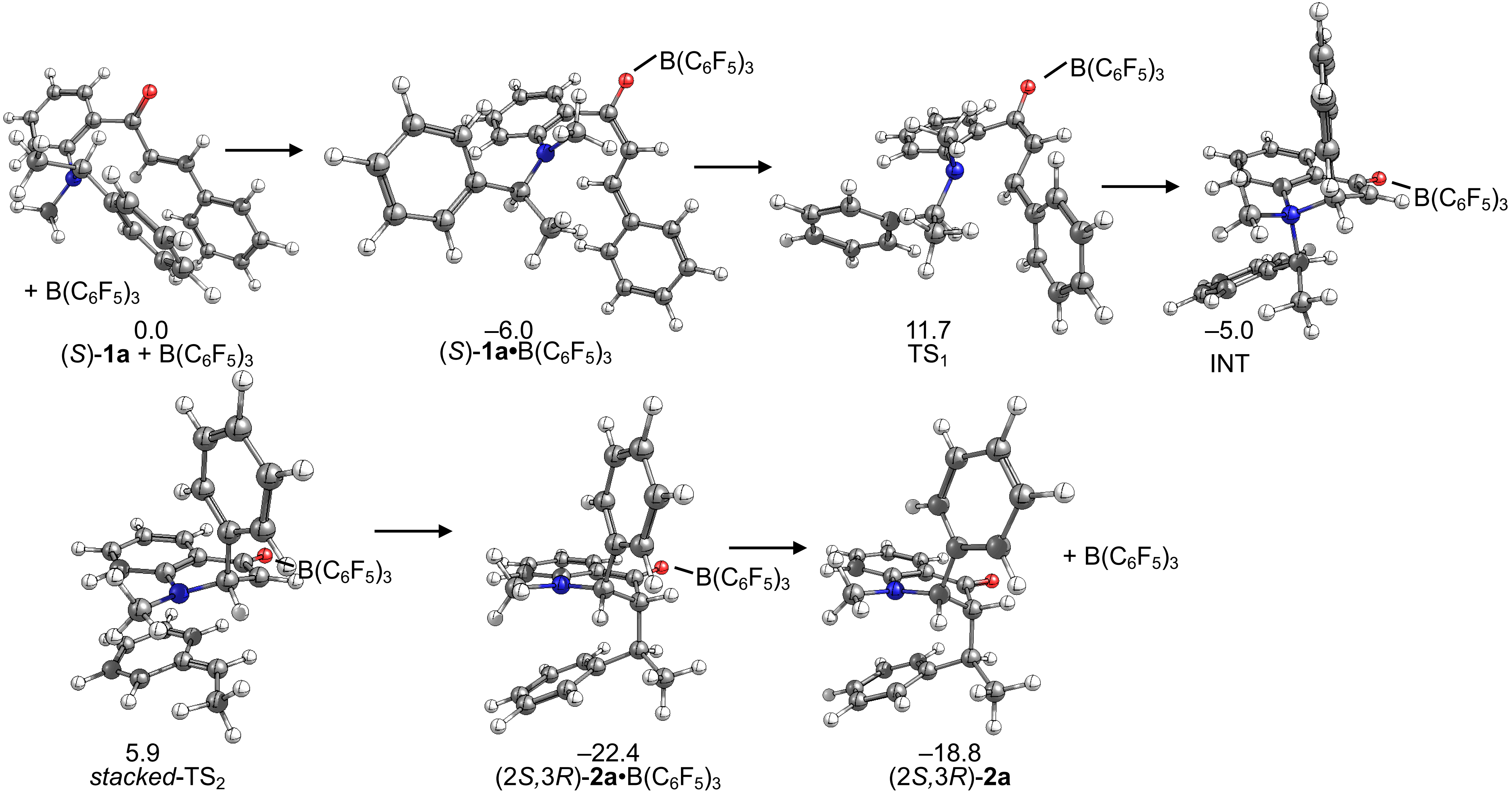

Figure S2: *Lowest free energy (kcal/mol) trajectories (PW6B95/def2-QZVPP//PBEh-3c/def2-mSVP) for (S)-**1a** → (2R,3S)-**2a***

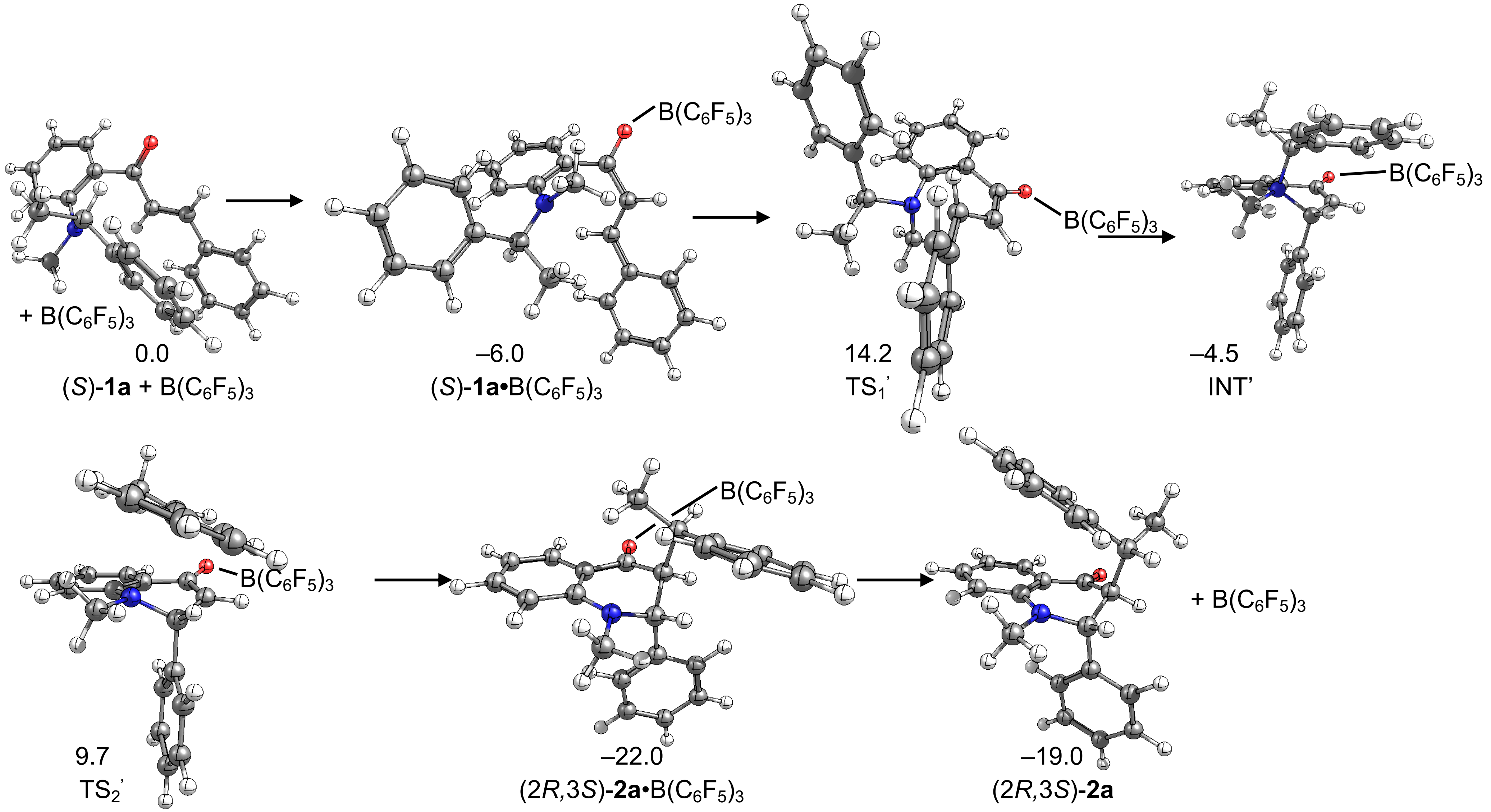

Figure S3: *Geometries and orbital representations of stacked-TS_2 at ωB95X-D3BJ/def2-TZVP level.*

stacked-TS_2 geometry
(UKS ωB95X-D3BJ/def2-TZVP)

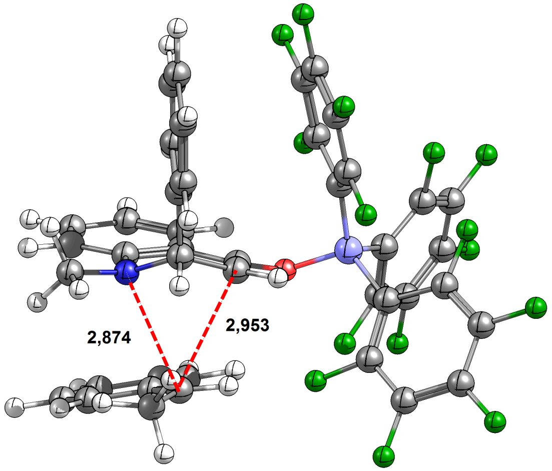

stacked-TS_2 geometry
(RKS ωB95X-D3BJ/def2-TZVP)

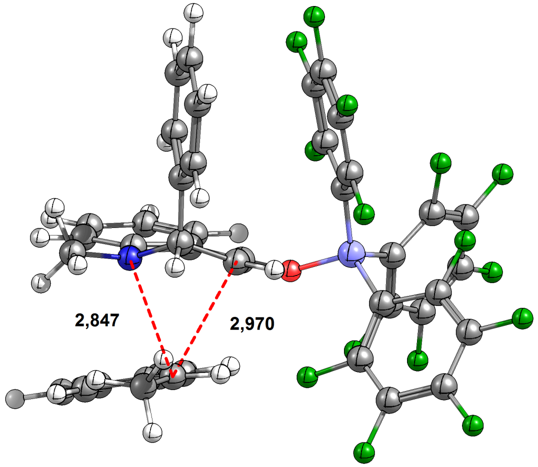

Orbital representations of stacked-TS_2 (UKS, wB95X-D3BJ/def2-TZVP)

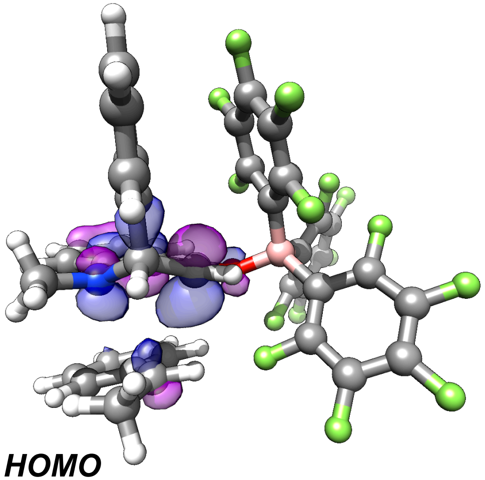

HOMO
(isovalue 0.05 electrons Bohr^{-3})

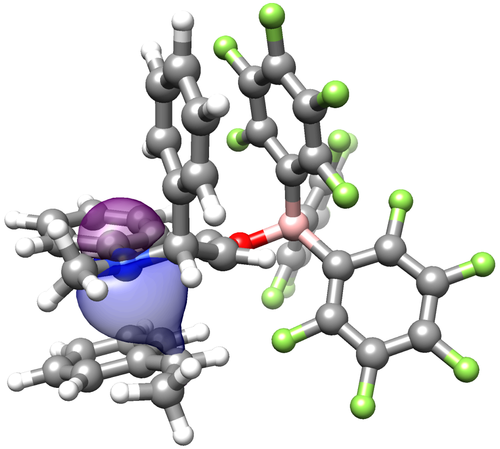

localized orbital N-aryl
(isovalue 0.03 electrons Bohr^{-3})

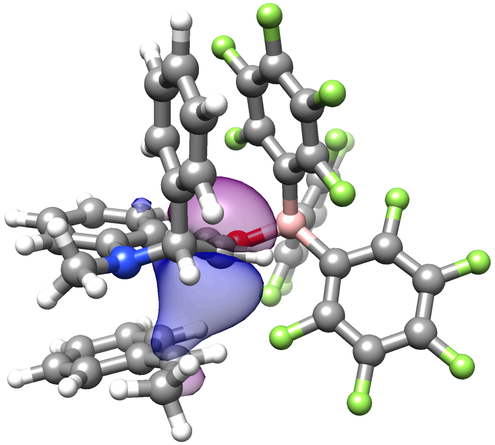

localized orbital enolate
(isovalue 0.03 electrons Bohr^{-3})

Figure S4: *Geometries and orbital representations of TS_2' ωB95X-D3BJ/def2-TZVP level.*

TS_2' geometry (UKS ωB95X-D3BJ/def2-TZVP)

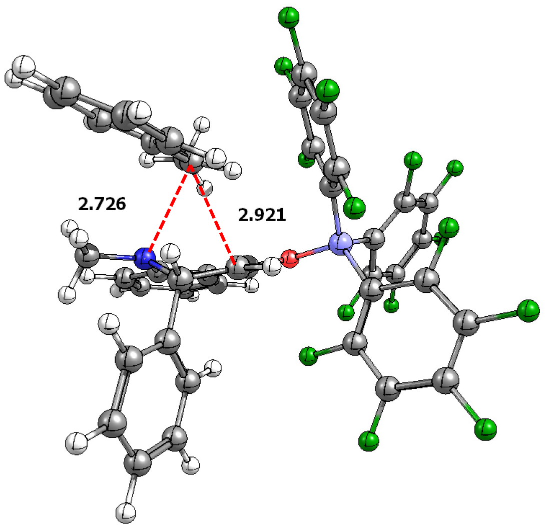

TS_2' geometry (RKS ωB95X-D3BJ/def2-TZVP)

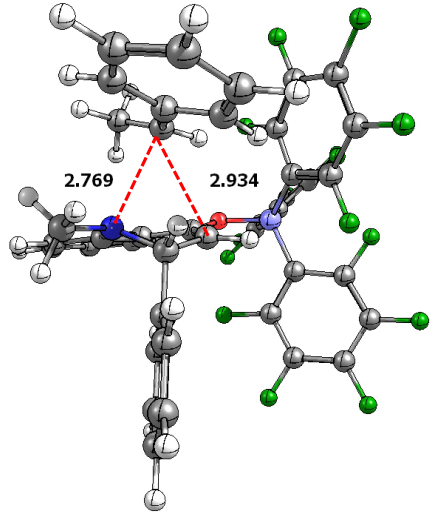

Orbital representations of TS_2' (UKS ωB95X-D3BJ/def2-TZVP)

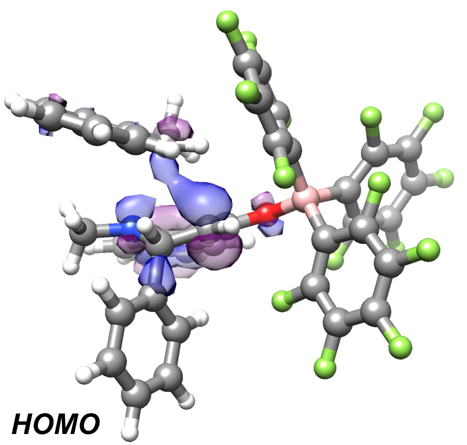

HOMO
(isovalue 0.05 electrons $Bohr^{-3}$)

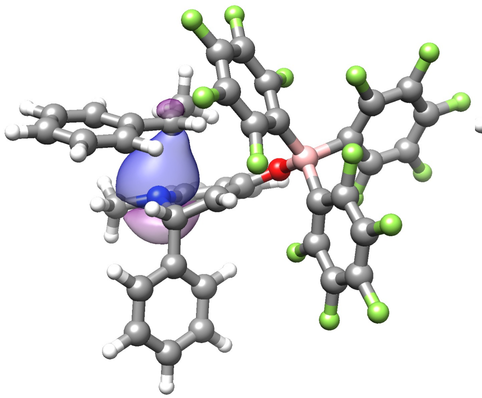

localized orbital N-aryl
(isovalue 0.03 electrons $Bohr^{-3}$)

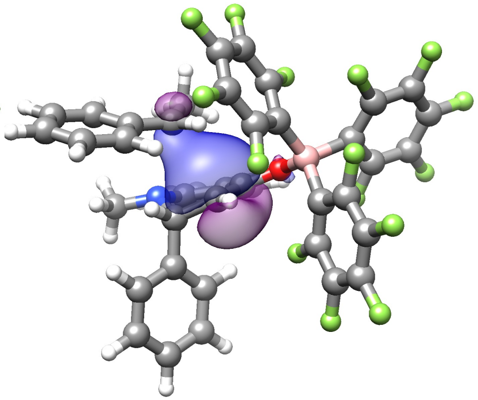

localized orbital enolate
(isovalue 0.03 electrons $Bohr^{-3}$)

Figure S5: *Geometries of stacked-TS$_2$ and TS$_2$ (top) and representation of non-covalent interactions (NCI); isovalue 0.6 electron Bohr^{-3}.*

stacked-TS$_2$ geometry (RKS PBEh-3c/def2-mSVP) ***TS$_2$ geometry (RKS PBEh-3c/def2-mSVP)***

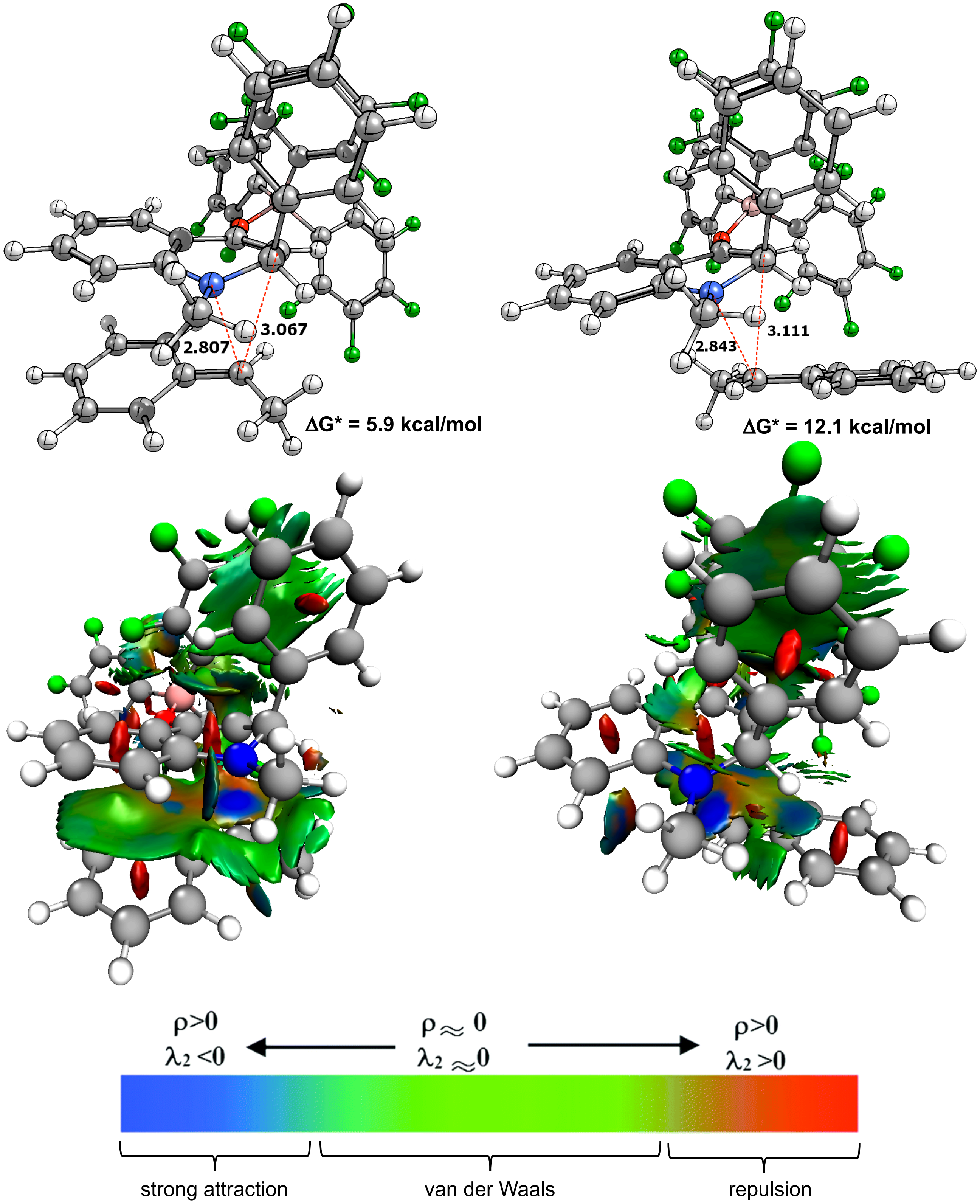

Figure S6: *Geometries of stacked-TS_2' and TS_2' (top) and representation of non-covalent interactions (NCI); isovalue 0.6 electron Bohr^{-3}.*

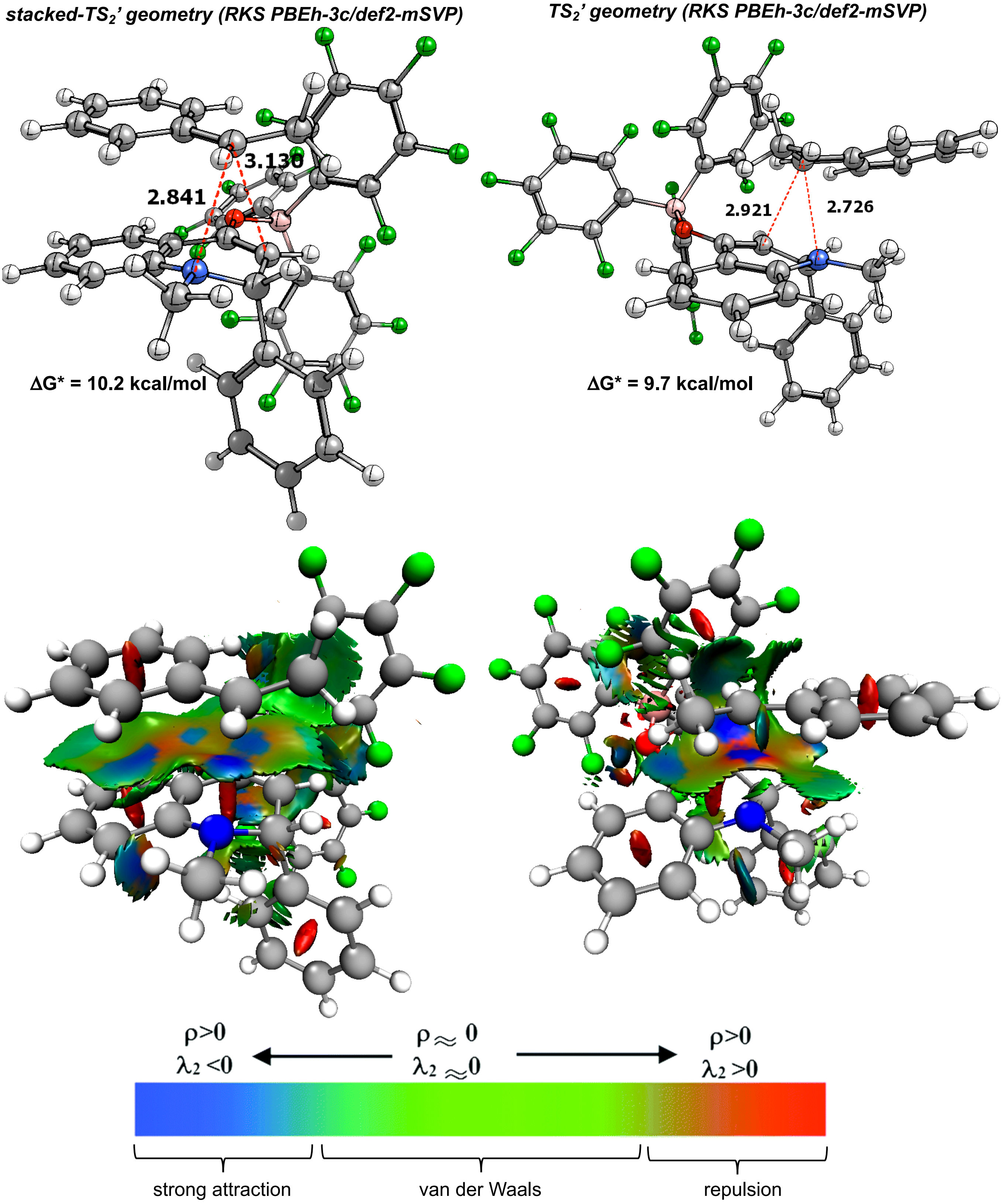

Figure S7: *Trajectory for the conversion of* ***1b*** *into* ***2b*** *(arbitrary enantiomer); free energies in kcal/mol.*

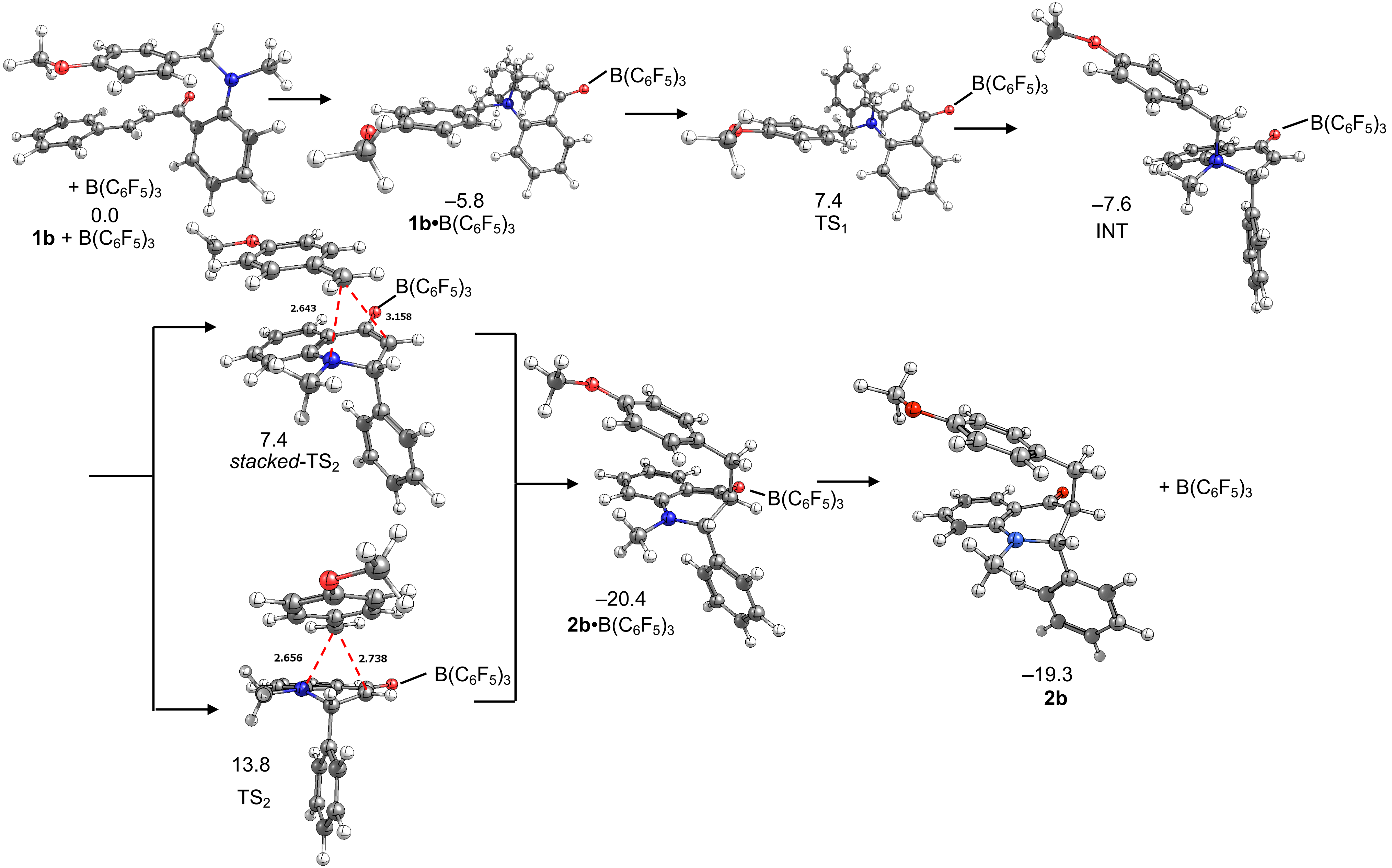

Figure S8: *Geometries of stacked-TS$_2$ and TS$_2$ (top) of substrate* ***1b*** *and representation of non-covalent interactions (NCI); isovalue 0.6 electron Bohrs^{-3}.*

stacked-TS$_2$ geometry for 1b (RKS PBEh-3c/def2-mSVP)

TS$_2$ geometry for 1b (RKS PBEh-3c/def2-mSVP)

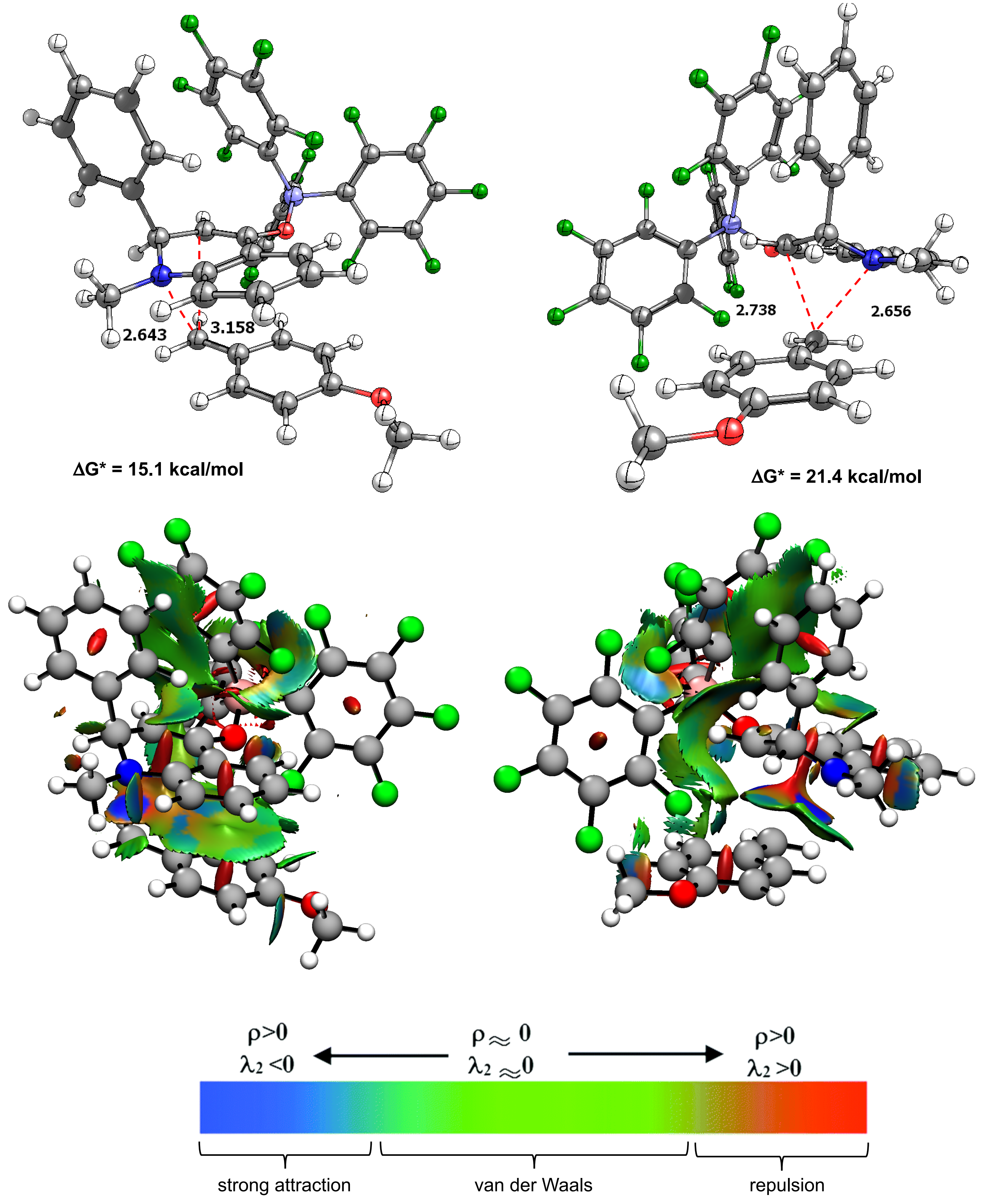

Supplement: Supplementary file 1 — Supporting Information [file ANIE-61-0-s001.pdf]
